# 1,5-Hydrogen Atom Transfer/Surzur–Tanner Rearrangement: A Radical Cascade Approach for the Synthesis of 1,6-Dioxaspiro[4.5]decane and 6,8-Dioxabicyclo[3.2.1]octane Scaffolds in Carbohydrate Systems

**DOI:** 10.1021/acs.joc.1c01376

**Published:** 2021-09-23

**Authors:** Elisa I. León, Ángeles Martín, Adrián S. Montes, Inés Pérez-Martín, María del Sol Rodríguez, Ernesto Suárez

**Affiliations:** † Síntesis de Productos Naturales, Instituto de Productos Naturales y Agrobiología del CSIC, Avda. Astrofísico Francisco Sánchez 3, 38206 La Laguna, Tenerife, Spain; ‡ Doctoral and Postgraduate School, Universidad de La Laguna, Avda. Astrofísico Francisco Sánchez s/n, 38200 La Laguna, Tenerife, Spain

## Abstract

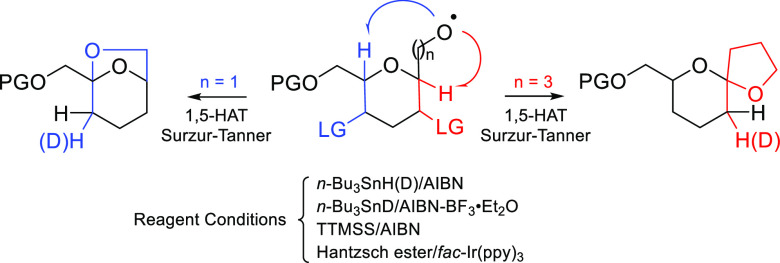

The 1,5-HAT–1,2-(ester)alkyl radical migration (Surzur–Tanner rearrangement) radical/polar sequence triggered by alkoxyl radicals has been studied on a series of *C*-glycosyl substrates with 3-*C*-(α,β-d,l-glycopyranosyl)1-propanol and *C*-(α-d,l-glycopyranosyl)methanol structures prepared from chiral pool d- and l-sugar. The use of acetoxy and diphenoxyphosphatoxy as leaving groups provides an efficient construction of 10-deoxy-1,6-dioxaspiro[4.5]decane and 4-deoxy-6,8-dioxabicyclo[3.2.1]octane frameworks. The alkoxyl radicals were generated by the reaction of the corresponding *N*-alkoxyphthalimides with group 14 hydrides [*n*-Bu_3_SnH(D) and (TMS)_3_SiH], and in comparative terms, the reaction was also initiated by visible light photocatalysis using the Hantzsch ester/*fac*-Ir(ppy)_3_ procedure. Special attention was devoted to the influence of the relative stereochemistry of the centers involved in the radical sequence on the reaction outcome. The addition of BF_3_•Et_2_O as a catalyst to the radical sequence resulted in a significant increase in the yields of the desired bicyclic ketals.

## Introduction

The development of synthetic methodologies for bicyclic 1,6-dioxaspiro[4.5]decane^1^ and 6,8-dioxabicyclo[3.2.1]octane (6,8-DOBCO)^2^ scaffolds is largely stimulated by their occurrence as the structural core of highly active insect pheromones.^3^ They can also be widely found as subunits^4^ in the structure of other more complex and biologically important natural products such as steroids,^5^ polyether ionophores,^6^ and marine toxins.^7^ In some cases, both structural motifs are present in the same natural skeleton, as occurs in pinnatoxins and the related pteriatoxins, potent neurotoxins of a dinoflagellate origin.^8^ Moreover, both bicyclic ketals have attracted much interest from synthetic chemists as versatile building blocks in fine organic synthesis.^9^

In the carbohydrate field, the preparation of spiro-heterocycles has been recently reviewed.^10^ Several naturally occurring 2,7-anhydro-β-d-*glyco*-hept-2-ulopyranose sugars with 6,8-dioxabicyclo[3.2.1]octane structures have been described. The most representative example is sedoheptulosan (2,7-anhydro-β-d-*altro*-hept-2-ulopyranose), although analogous compounds with d-*gluco* and d-*manno* stereochemistry are also known.^11^

**Scheme 1:**
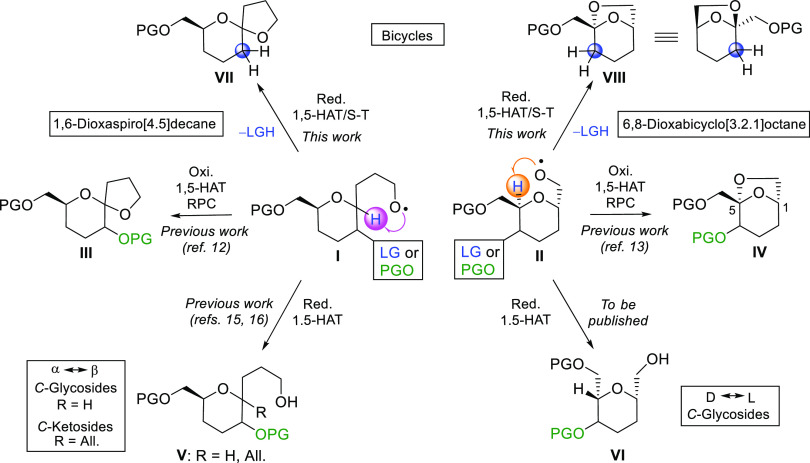
1,5-HAT Reactions of 3-*C*-(Glycopyranosyl)propan-1-*O*-yl and *C*-(Glycopyranosyl)methan-1-*O*-yl Radicals S–T = Surzur–Tanner; RPC = radical polar crossover; HAT = hydrogen atom transfer.

In previous papers, we reported on a new procedure for the stereoselective construction of 1,6-dioxaspiro[4.5]decane^12^ and 6,8-dioxabicyclo[3.2.1]octane^13^ frameworks on carbohydrate models as described in Scheme 1. Under mild oxidative conditions (PhI(OAc)_2_/I_2_), the initially generated alkoxyl radicals (i.e., I and II, PGO) trigger a 1,5-hydrogen atom transfer (1,5-HAT)^14^–radical oxidation–nucleophilic cyclization through a radical/polar crossover sequence that ultimately leads to the desired bicycles (i.e., III and IV, respectively) in a single step. In some cases, [4.5] spiroketal systems with a kinetic nonanomeric unstable configuration at the spiro center can be preferentially obtained using this methodology. Also using this simple procedure, natural *C*-glycosyl compounds of a *C*-(1,6-anhydro-β-d-*glyco*-1-ulopyranosyl)methanol structure (i.e., IV) with *rare* stereochemistries d-*ido*, d-*gulo*, and d-*altro* can be obtained from readily available d-*gluco*, d-*galacto*, and d-*manno* chiral pool sugars, respectively.^13^

Otherwise, the generation of the above-mentioned alkoxyl radicals (i.e., I and II, PGO) under reductive conditions proceeds by a different mechanism that allows the preparation of interesting and highly versatile chiral synthons. Homolytic intermolecular allylmethalation of the intermediate C1 radical may lead to *C*-ketosides (i.e., V, R = All).^15^ The regioselective HAT by alkoxyl radicals of the H5 enables also the C5-allylation and the possibility of preparing *C*-ketosides on both sides of the pyranosyl ring oxygen.^16^ Although, at first glance, the homolytic reduction of the C1- and C5-radical intermediates might seem of little synthetic utility, it allows a diastereoselective interconversion between d- and l-*C*-glycosides (i.e., VI)^17^ and α- and β-*C*-glycosides (i.e., V, R = H), which is difficult to achieve using conventional methods.^18^

Since the discovery by Surzur and Teissier^19^ and by Tanner and Law^20^ in the 1960s that β-(acyloxy)alkyl radicals undergo a 1,2-suprafacial migration of their ester group, this rearrangement has attracted considerable mechanistic and synthetic attention.^21^ The use of β-(phosphatoxy)alkyl radicals^22^ with a better leaving group (LG) and complexation with Lewis acids^23^ notably increase the reaction rate and consequently its importance from a synthetic point of view.

In carbohydrate chemistry, this rearrangement has been exploited for a convenient synthesis of 2-deoxypyranoses from 1-pyranosyl radicals^24^ and in the stereoselective preparation of purine and pyrimidine α-nucleosides.^25^ This rearrangement is also involved in the DNA and RNA strand scission from 2’- and 4’-radicals via the cleavage of the β-phosphate.^26^

It is evident that if we end the above-mentioned 1,5-HAT sequences with a β-(acyloxy)alkyl radical (i.e., I and II, LG), a simple and versatile preparation of 2-deoxy-*C*-glycosides on a 1-ulopyranose ring system (i.e., VII and VIII) could be achieved, where the HAT and the vicinal deoxygenation through an alkene radical-cation intermediate would occur in the same synthetic step. In fact, we would gain access to a series of ketoses with 5-deoxy-non-4-ulopyranose (i.e., VII) and 5-deoxy-hept-6-ulopyranose (also named 3-deoxy-hept-2-ulopyranose) (i.e., VIII) structures by long-range selective oxidation at C1 and C5 ring carbon atoms, respectively. The synthetic interest is apparent; the 3-deoxy-hept-2-ulopyranose framework present in VIII is intimately related to the ring system of octulosonic (Kdo, Kdn) and sialic acids.^27^ Procedures for the preparation of analogous [4.5] spiroketals in 2-deoxy-pyranose systems using different methodologies have been described in previous publications.^28^ In general, deoxy-pyranoses are important targets and are frequently found in bioactive secondary metabolites of microbial origin.^29^

In this paper, the 1,5-HAT–Surzur–Tanner (S–T) radical/polar sequence has been studied principally on a series of *C*-glycosyl substrates with 3-*C*-(glyco)1-propanol (i.e., I, LG) and *C*-(glyco)methanol (i.e., II, LG) structures prepared from chiral pool d- and l-sugar and with α- and β-configurations at the anomeric center. The initial alkoxyl radicals were generated by homolytic cleavage of the corresponding *N*-alkoxyphthalimide derivatives using the *n*-Bu_3_SnH/AIBN protocol under several different conditions.^30^ In most cases, the reaction finishes with an intramolecular nucleophilic 5-cyclization at the *cine* position of the radical-cation–LG anion pair intermediate to give the expected bicyclic acetal (i.e., VII or VIII) with a deoxygenated carbon atom at the vicinal position.^21e^

To unambiguously determine the fate of the radical throughout the cascade sequence, the experiments will also be performed with *n*-Bu_3_SnD/AIBN. This will allow us, among other things, to detect whether in the last step of the sequence the β-elimination of the ester takes place by the expected radical-polar β-(ester)alkyl shift mechanism or by a competitive pure radical β-(ester)alkyl fragmentation.^21^ Additionally, the influence of boron trifluoride as a catalyst on the sequence outcome will be addressed. In comparative terms, the reaction was also initiated by visible light photocatalysis using the Hantzsch ester/*fac*-Ir(ppy)_3_ procedure.^31^ In all cases, the reactions were allowed to proceed until the complete consumption of the radical precursors as indicated by TLC.

Due to the stereochemical requirements for the HAT reaction transition state,^32^ much attention has been paid to the not always apparent conformation of the sugar rings in these *C*-glycosyl compounds. For this purpose, the ^3^*J*_HCCH_ vicinal ring coupling constants were extracted from the experimental 1D ^1^H NMR spectra by iterative simulation^33^ and compared with the values calculated on minimized structures in ^4^*C*_1_ and ^1^*C*_4_ conformations [see Tables S1 and S2 in the Supporting Information (SI)].^34^

Previous examples of the HAT–S–T rearrangement sequence have been reported in the formation of tetrahydrofurans from β-(phosphatoxy)alkyl radicals^35^ and as a key step in the synthesis of cephalosporolide E.^36^ We have also described another example of this sequence during the reaction of methyl **2,3,4-tri-***O***-acetyl-6-deoxy-**α-d-Tal*p*-(1 → 4)-2,3-di-*O*-methyl-α-d-Glc*p*-6-*O*-yl disaccharide radical.^37^ The initial 1,8-HAT(6^I^O^•^ → 5^II^C^•^) between the two sugars generated a 4^II^β-(acetoxy)5^II^-alkyl radical that led finally to the formation of a rare eight-membered 4^II^-deoxy-1,3,5-trioxocane ring system. The use of *n*-Bu_3_SnD confirms that, at least in part, the last step of the sequence involves an S–T rearrangement through a *cine* 8-*exo*-substitution mechanism. On the other hand, unsuccessful attempts to trap the intermediate alkene radical cation intramolecularly by carboxylate anions have been reported.^38^

To obtain a complete picture of the stereochemical influence of the substituents in the course of the radical sequence, we have prepared 3-*C*-(glycopyranosyl)1-propoxyphthalimides with α,β-d-*gluco* (**1**–**4**), α,β-d-*manno* (**5**–**8**), α-l-*fuco* (**9** and **10**), and α,β-d-*arabino* (**11** and **12**) configurations (Scheme 4).^39^ A few examples of 3-*C*-(α-d-ribofuranosyl)1-propoxyphthalimides (**13**–**15**) have been included in this work to study the influence of the greater conformational flexibility of the five-membered ring (Scheme 5). Furthermore, *C*-(glycopyranosyl)*N*-methoxyphthalimides with α-d-*gluco* (**16**–**19**), α-d-*galacto* (**20** and **21**), α-l-*rhamno* (**22**), and α-l-*fuco* (**23** and **24**) configurations (Scheme 6) have also been synthesized (Schemes 4–6 are presented later in this work). In most of these models, it has been possible to investigate the differences between the migratory capabilities of acetoxy and diphenoxyphosphatoxy groups and how they affect the final result of the sequence.^40^

## Results and Discussion

### Synthesis of 10-Deoxy-1,6-dioxaspiro[4.5]decane and 9-Deoxy-1,6-dioxaspiro[4.4]nonane Scaffolds

The results of the study with 3-*C*-(α,β-d-Glc*p*)propan-1-*O*-yl radicals using 2-acetyl and 2-diphenoxyphosphoryl as LGs are summarized in Table 1. Initial experiments with 3-*C*-(2-*O*-acetyl-α-d-Glc*p*)1-propoxyphthalimide precursor **1** employing conditions optimized for the generation of alkoxyl radicals from *N*-alkoxyphthalimides using *n*-Bu_3_SnH (1 equiv) in a dilute solution (0.013 M) of toluene at reflux temperature and AIBN as the initiator gave a mixture of three compounds: **25**, **26β**, and **26α** (Table 1, entry 1). The major product **25** was identified as the expected 1,5-HAT–S–T spiroketal. The minor components of the reaction are **26β**, which is formed by hydrogen abstraction at C-1 and subsequent radical axial quenching with inversion of configuration, and isomeric alcohol **26α**, which could arise either by abstraction and retention of the configuration or simply by premature reduction of the alkoxyl radical. In the latter case, a combination of both mechanisms could be operative and cannot be ruled out at the present stage of the work. The yield of the cyclized product **25** was increased to 50% by lowering the tin hydride concentration with a syringe pump; under these conditions, the *C*-glucosyl compound **26β** could not be detected (Table 1, entry 2). Attempts to improve the yield using (TMS)_3_SiH (TTMSS), a group 14 hydride with a smaller hydrogen donor capacity,^41^ to avoid the reduction of radical intermediates met with no success.

**Table 1:**
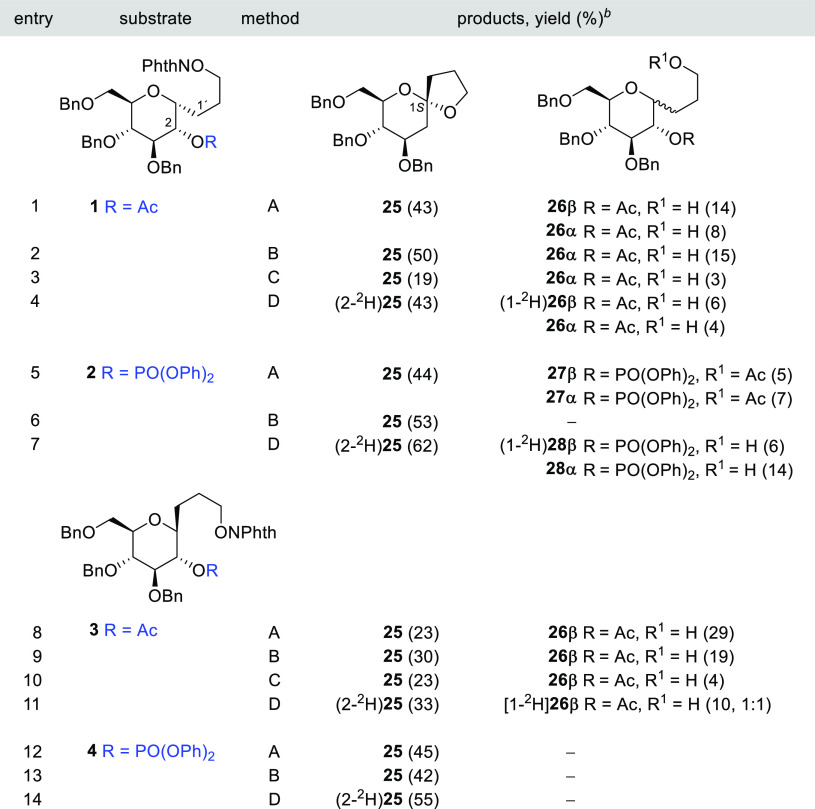
1,5-HAT–S–T Sequence in 3-*C*-(α,β-d-Glc*p*)1-propoxyphthalimides **1**–**4**^a^

a Reagents and conditions: method A: *n*-Bu_3_SnH (1 equiv), AIBN (0.1 equiv), PhCH_3_ (0.013 M), reflux; method B: *n*-Bu_3_SnH (1 equiv/h), AIBN (0.1 equiv), PhCH_3_ (0.013 M), reflux; method C: TTMSS (1 equiv), AIBN (0.1 equiv), PhCH_3_ (0.013 M), reflux; and method D: *n*-Bu_3_SnD (1 equiv), AIBN (0.1 equiv), PhCH_3_ (0.013 M), reflux.

b Values in parentheses are isolate yields; deuterium incorporation (^2^H/^1^H) is included in partially labeled compounds.

Considerable analytical and spectroscopic data were diagnostic of the spiroketal structure of **25**, unambiguously expressing the presence of a quaternary ketal carbon and the additional methylene group as well as the disappearance of the acetyl group. In a minimized structure, the pyranose ring adopts preferentially a ^4^*C*_1_ chair conformation, from which the calculated coupling constants were in agreement with the experimental values (see Table S3 in the SI). The configuration of the spiro center was tentatively assigned as 1*S*, with the anomeric oxygen in an axial position,^42^ according to the downfield displacement observed for the H3 and H5 protons that in this conformation present 1,3-diaxial interactions with the C1–O bond. In addition, the absence of NOE interactions between H1’ and H5 and/or H3 that were present in previously reported analogous [4.5] spiroketals in 2-deoxy-pyranose systems with 1*R* stereochemistry may also support this assignment.^28a^

The use of *n*-Bu_3_SnD showed the quantitative monodeuteration for (2-^2^H)**25** and for the inverted product (1-^2^H)**26β** (Table 1, entry 4). Moreover, the early reduction of the alkoxyl radical was solely responsible for the unlabeled **26α** and no retention at C1 could be detected in this experiment. The diastereoselective ratio (^2^H_ax_/^2^H_eq_, 7:1) of deuterium at (2-^2^H)**25** is mostly attributable to a β-facial preference for the radical quenching due to steric hindrance.

For comparative purposes, we have prepared *C*-(2-*O*-diphenoxyphosphoryl-α-d-Glc*p*)1-propoxyphthalimide **2**. The rate constant of the β-(phosphatoxy)alkyl radical migration should be several orders of magnitude greater than that recorded for comparable acyloxy shifts.^21d^ However, the reaction of **2** with *n*-Bu_3_SnH/AIBN afforded the 1,5-HAT–S–T substitution product **25** in a similar yield (44%) together with a mixture of alcohols that, after acetylation, were identified as **27β** and **27α** (Table 1, entry 5). The slow addition of *n*-Bu_3_SnH generates the bicycle **25** as a sole product in 53% yield (Table 1, entry 6). The reaction with *n*-Bu_3_SnD showed the complete monodeuteration for the spirocompound (2β-^2^H)**25** achieved in a significantly better yield (62%) (Table 1, entry 7). The inseparable mixture of the complete labeling inverted product (1-^2^H)**28β** and the reduced unlabeled alcohol **28α** was also obtained (20%, 1:2.1).

This protocol was also applied to *C*-(2-*O*-acetyl-β-d-Glc*p*)1-propoxyphthalimide **3**, where the pyranose ring adopts preferentially a ^4^*C*_1_ conformation with the three-carbon tether in an equatorial position and, consequently, the abstractable hydrogen atom at C1 is axially oriented (see Table S1 in the SI). Unfortunately, treatment of **3** under the same conditions mentioned above did not increase the yield of **25** (Table 1, compare entries 8–10 with 1–3). Now, the principal compound is *C*-glucosyl compound **26β**, and according to the reaction with *n*-Bu_3_SnD, approximately 50% is formed by prereduction of the alkoxyl radical {[1-^2^H]**26β** (^2^H/^1^H, 1:1)} (Table 1, entry 11). These results were rather unexpected since electrophilic radicals abstract axial hydrogen atoms much faster than the equatorial ones and the initial 1,5-HAT should be favored relative to our previous models **1** and **2**.^43^ The different reactivity between **1** and **3** can be explained by a possible memory of chirality effect of the C1 radical after the 1,5-HAT reaction (Table 1, compare entries 1 and 8).^44^

Moreover, the migration of a phosphatoxy group contributed to a marked improvement in the yield of **25** as shown in model **4** (Table 1, entries 12–14). Under these conditions, no appreciable amounts of *C*-glucosyl compounds resulting from the reduction of intermediate radicals were detected. As observed in previous models, the yield of the spiroketal increased significantly when changing from a hydride donor **25** to a less reactive deuteride donor (2-^2^H)**25** (Table 1, compare entries 12 and 14). These results probably reflect a kinetic isotope effect (KIE) in which a slower process permits the radical to reach the end of the sequence, avoiding prereduction and the formation of uncyclized products.

The reaction of an analogous series of 3-*C*-(α,β-d-Man*p*)propan-1-*O*-yl radicals using also 5-acetyl and 5-diphenoxyphosphatoxy as LGs is summarized in Table 2. The 3-*C*-(2-*O*-acetyl-α-d-Man*p*)1-propoxyphthalimide **5** under the classical tin hydride conditions afforded exclusively uncyclized compounds **29β** and **29α**, as confirmed by deuterium labeling experiments (Table 2, entries 1 and 2). The expected spiroketal **25** could not be detected.

**Table 2:**
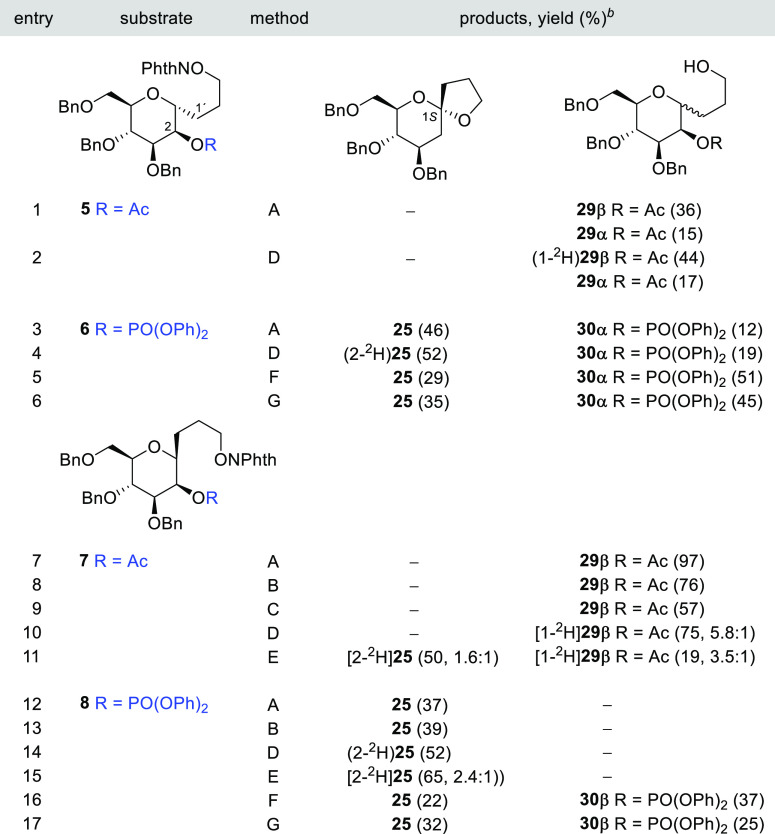
1,5-HAT–S–T Sequence in 3-*C*-(α,β-d-Man*p*)1-propoxyphthalimides **5**–**8**^a^

a Reagents and conditions: method A: *n*-Bu_3_SnH (1 equiv), AIBN (0.1 equiv), PhCH_3_ (0.013 M), reflux; method B: *n*-Bu_3_SnH (1 equiv/h), AIBN (0.1 equiv), PhCH_3_ (0.013 M), reflux; method C: TTMSS (1 equiv), AIBN (0.1 equiv), PhCH_3_ (0.013 M), reflux; method D: *n*-Bu_3_SnD (1 equiv), AIBN (0.1 equiv), PhCH_3_ (0.013 M), reflux; method E: *n*-Bu_3_SnD (1 equiv), BF_3_•Et_2_O (0.2 equiv), AIBN (0.1 equiv), PhCH_3_ (0.013 M), reflux; method F: Hantzsch ester (1.1 equiv), *fac*-Ir(ppy)_3_ (0.01 equiv), THF (0.007 M), rt, blue LED; and method G: Hantzsch ester (0.37 equiv/h), *fac*-Ir(ppy)_3_ (0.01 equiv), THF (0.007 M), rt, blue LED.

b Values in parentheses are isolate yields; deuterium incorporation (^2^H/^1^H) is included in partially labeled compounds.

The isomeric β-phthalimide **7** behaved similarly, with only **29β** (97%) being obtained (Table 2, entry 7). Under tin deuteride conditions, the reaction gave [1-^2^H]**29β** (^2^H/^1^H, 5.8:1); the labeled compound originated by deuterium incorporation with retention after the 1,5-HAT and the unlabeled compound by direct reduction of the alkoxyl radical (Table 2, entry 10). Knowing that aluminum and scandium Lewis acid can efficiently enhance the rate of the S–T rearrangement,^23^ we envisioned an experiment that under the same conditions [*n*-Bu_3_SnD (1 equiv), AIBN (0.1 equiv), and PhCH_3_ (0.013 M) at 110 °C] contains a catalytic amount of BF_3_•Et_2_O (0.2 equiv). To our delight, the radical sequence now proceeded nicely to the end, furnishing the desired product [2-^2^H]**25** in 50% yield along with a small amount of [1-^2^H]**29β** (19%) (Table 2, entry 11). The ^1^H NMR analysis of the deuterium incorporation at [2-^2^H]**25** (^2^H/^1^H, 1.6:1) revealed that, in this case, the β-elimination of the ester could take place not only by the radical-polar β-(acyloxy)alkyl shift mechanism but also by a competitive pure radical β-(acyloxy)alkyl fragmentation.^45^ Alternatively, acid-catalyzed opening and recombination of the spiroketal ring through an unobserved glucal intermediate [3-*C*-(1,5-anhydro-2-deoxy-d-*arabino*-hex-1-enopyranosyl)propan-1-ol] may also account for the loss of deuterium detected.

The 3-*C*-(2-*O*-diphenoxyphosphoryl-α,β-d-Man*p*)1-propoxyphthalimide models **6** and **8** with a faster migratory group gave, under standard tin hydride conditions, spiroketal **25** in moderate yields (Table 2, entries 3 and 12, respectively). The yields of (2-^2^H)**25** improved using tin deuteride (52% in both cases) and increased notably upon Lewis acid catalysis giving [2-^2^H]**25** (65%, ^2^H/^1^H, 2.4:1) by partial labeling (Table 2, entries 4, 14, and 15). The difference in reactivity between 2-acetyl-d-*gluco* (**1** and **3**) and -d-*manno* derivatives (**5** and **7**) has been attributed to the observed lower migration efficiency of axial β-(acetoxy)alkyl radicals.^24b,^^46^

The formation of alkoxyl radicals from *N*-alkoxyphthalimide under photoredox catalysis conditions and their use in selective C(sp^3^)–H functionalization through 1,5-HAT have been recently reported.,^31b^^47^ As far as we know, this type of methodology has never been employed to initiate the 1,5-HAT–S–T sequence described in this paper. The blue LED irradiation of phthalimides **6** and **8** in the presence of a catalytic amount of *fac*-[Ir(ppy)_3_] and Hantzsch ester as the reductant afforded spirocycle **25** in a disappointingly low yield, with the prereduced alcohols **30α** and **30β**, respectively, being the major products (Table 2, entries 5 and 16). Although the yield of **25** increased slightly by slowly adding the Hantzsch ester to the reaction mixture using a syringe pump, it is still clearly inferior to the results obtained with the tin hydrides (Table 2, entries 6 and 17).

Next, this study was extended to the acetyl and diphenoxyphosphoryl 3-*C*-(α-l-Fuc*p*)1-propoxyphthalimides **9** and **10**, respectively, as described in Table 3. When the 2-acetyl precursor **9** was treated with *n*-Bu_3_SnH/AIBN, the main product was the expected spirocycle **31** (46%) together with an inseparable mixture (2:3, 15%) of two minor alcohols: 6-deoxy-d-*altro***32** and l-*fuco***33** (Table 3, entry 1). Although both diastereomers can be tentatively identified by NMR analysis of the mixture, additional support for these structures came from the complete separation and characterization of diphenoxyphosphoryl analogues **34** and **35** achieved during the reaction of phthalimide **10** (Table 3, entry 5).

**Table 3:**
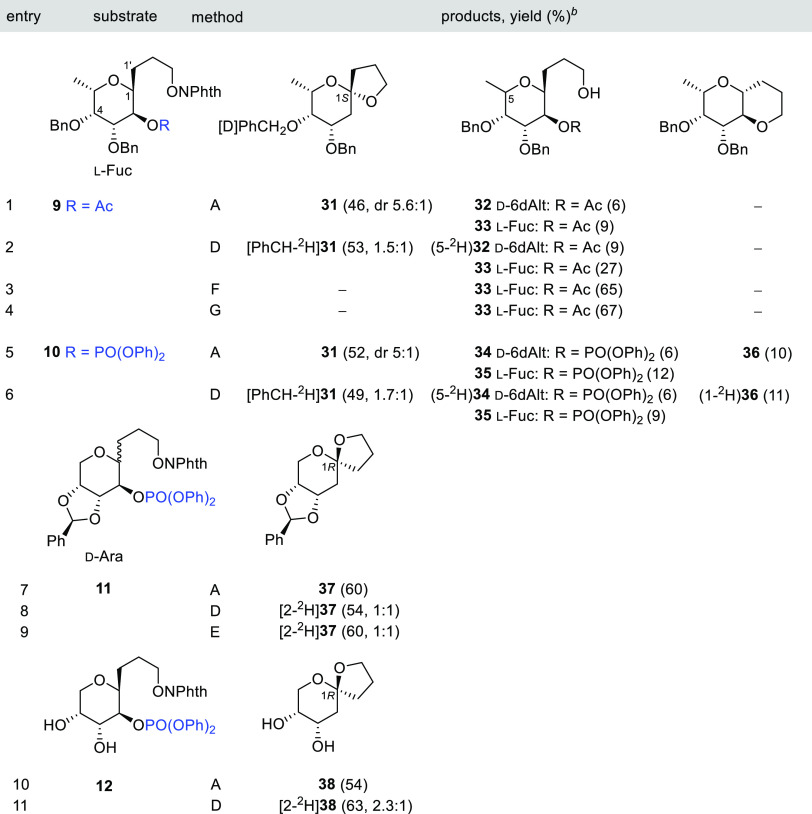
1,5-HAT–S–T Sequence in 3-*C*-(α-l-Fuc*p*)- and 3-*C*-(d-Ara*p*)1-propoxyphthalimides **9**–**12**^a^

a Reagents and conditions: method A: *n*-Bu_3_SnH (1 equiv), AIBN (0.1 equiv), PhCH_3_ (0.013 M), reflux; method D: *n*-Bu_3_SnD (1 equiv), AIBN (0.1 equiv), PhCH_3_ (0.013 M), reflux; method E: *n*-Bu_3_SnD (1 equiv), BF_3_•Et_2_O (0.2 equiv), AIBN (0.1 equiv), PhCH_3_ (0.013 M), reflux; method F: Hantzsch ester (1.1 equiv), *fac*-Ir(ppy)_3_ (0.01 equiv), THF (0.007 M), rt, blue LED; and method G: Hantzsch ester (0.37 equiv/h), *fac*-Ir(ppy)_3_ (0.01 equiv), THF (0.007 M), rt, blue LED.

b Values in parentheses are isolate yields; deuterium incorporation (^2^H/^1^H) is included in partially labeled compounds. dr = diastereomeric ratio; only the major isomer is shown.

The spiroketal **31** with a nonanomeric configuration at the spirocenter was isolated and contaminated with a small amount of the thermodynamic isomer (1*S*/1*R*, 85:15). In both isomers, the pyranosyl ring preferentially adopts a ^1^*C*_4_ chair conformation (see Table S3 in the SI). The ^1^H NMR spectrum of **31** shows a ^4^*J*_w_ coupling (1.3 Hz, calcd 1.3 Hz)^48^ between H2α and H4 equatorial hydrogens, which also supports the mentioned conformation (see Table S4 in the SI). The 1*S* configuration of the major isomer (shown in Table 3) was established based on the NOE interactions observed between H5 and H1’ and H2’. The downfield displacement observed for H3 (0.1 ppm) and H5 (0.3 ppm) in the ^1^H NMR spectrum of the minor 1*R*-isomer lends further evidence to the proposed spiroketal stereochemistry.

Several interesting conclusions can be drawn from the results obtained during the deuteration experiment (Table 3, entry 2). Compound [PhCH-^2^H]**31** (D/H = 1.5:1; dr = 4:1) showed no significant incorporation of deuterium at the C2 site, but instead a partial deuteration of a benzylic proton at C4 could be detected. The quantitative incorporation of deuterium confirmed that the 6-deoxy-d-altrose derivative (5-^2^H)**32** was formed by the reductive inversion of configuration of a 5-radical intermediate. Finally, the undeuterated alcohol **33** was formed exclusively by prereduction of the initial alkoxyl radical. All our attempts to obtain spirocyclic **31** by applying the photoredox conditions mentioned above were unsuccessful, with only prereduced alcohol **33** being isolated instead (Table 3, entries 3 and 4).

The reaction of 3-*C*-(2-diphenoxyphosphoryl-α-l-Fuc*p*)1-propoxyphthalimide **10** provided the desired bicycle **31** in better yield (52%) together with small amounts of the 6-deoxy-d-*altro***34** and l-*fuco***35** derivatives that could now be conveniently characterized. The 6-deoxy-β-d-altropyranosyl ring in **34** exists preferentially in a ^4^*C*_1_ conformation with the two alkyl residues in an equatorial position, with the value of the ^3^*J*_4,5_ = 9.8 Hz (calcd = 8.4 Hz) confirming the inversion of configuration at C5. Furthermore, a new compound **36** with a 2,7-dioxabicyclo[4.4.0]decane skeleton, hitherto undetected in the reaction of previous models, was also isolated in 10% yield (Table 3, entry 5). The structure and stereochemistry of **36**, a constitutional isomer of **31**, were readily established by analytic and spectroscopic means. Most significantly, the ^3^*J* fucopyranosyl ring coupling constants extracted by DAISY from the experimental spectrum and NOE interactions of H1 with H3 and H5, and H2 with H1’ confirmed the *trans*-fused bis(pyran) proposed framework.^49^

**Scheme 2:**
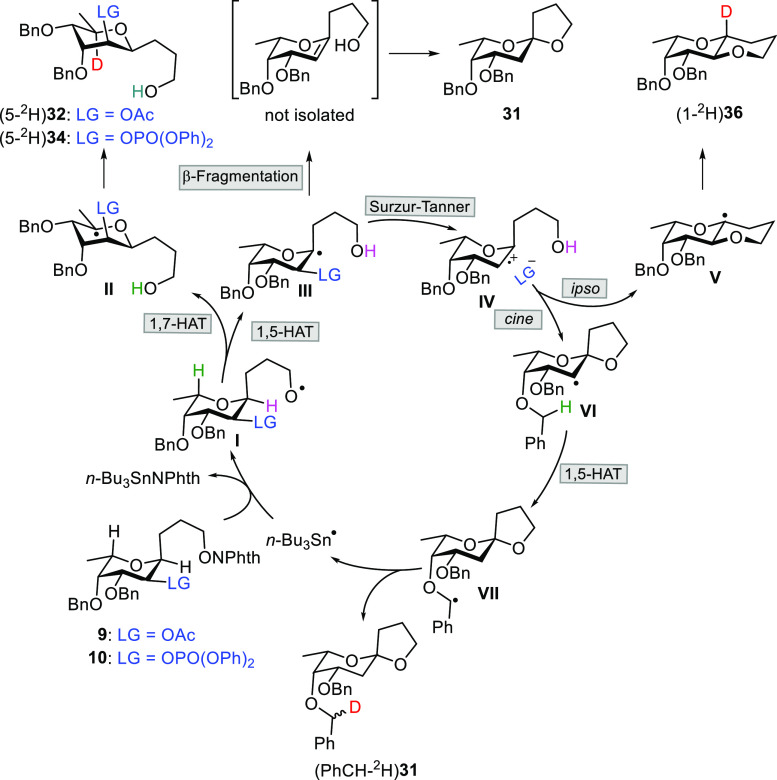
Propagation Cycle of 3-*C*-(α-l-Fuc*p*)propan-1-*O*-yl Radicals The prereduction of the alkoxyl radical and the initiation and termination steps are omitted for clarity.

A possible propagation cycle for the acetyl and diphenoxyphosphoryl 3-*C*-(α-l-Fuc*p*)propan-1-*O*-yl radical chain reactions, employing tin deuteride as reductant, is outlined in Scheme 2. The electrophilic alkoxyl radical (I) triggers two competitive hydrogen atom transfer reactions by abstraction of stereochemically accessible 1H (1,5-HAT) and 5H (1,7-HAT). Many examples of 1,5-hydrogen translocations are known; however, their 1,7-HAT counterparts are comparatively very scarce.^14,50^ The 5-alkyl radical (II) leads finally to 3-*C*-(6-deoxy-β-d-altropyranosyl)1-propanol derivatives (5-^2^H)**32** and (5-^2^H)**34** with inversion of configuration. The 1-alkyl radical (III) continues the cascade sequence by the two mechanisms mentioned before: pure radical β-fragmentation to give unlabeled **31**, through a non-isolated olefin, and S–T rearrangement through the radical-polar intermediate (IV).^45^ When phosphate is used as LG, the reaction is directed toward two competitive pathways: *cine* and *ipso* intramolecular cyclization by the primary alcohol that now acts as a nucleophile. The minor *ipso* cyclization affords the bis(pyran) (1-^2^H)**36** through radical (V). Furthermore, the *cine* substitution provides 2-radical (VI) that regioselectively abstracts a benzylic hydrogen from the 4-OBn protecting group by means of another 1,5-HAT process. Consequently, no deuterium incorporation (within the limits of NMR detection) was observed at C2. Reductive quenching of radical VII leads to the quantitatively deuterated (PhCH-^2^H)**31**, isolated together with the unlabeled **31** formed by the β-fragmentation mechanism.

The ^1^H and ^13^C{^1^H} NMR spectra of isolated [PhCH-^2^H]**31** (D/H, 1.5:1; dr = 4:1) deserve some comments. The deuteration at 4-OBn is highly stereoselective, providing evidence for a steric hindered deuteride addition. Both diastereoisomeric deuterated benzyl ethers seem to adopt two different conformations that affect the chemical shift displacement of surrounding protons and carbons. Thus, for example, the 6-methyl group signal appears as three doublets of approximately the expected intensities (1.5:0.3:1.2): 1.130 ppm (*J* = 6.3 Hz, D major), 1.133 ppm (*J* = 6.6 Hz, D minor), and 1.135 ppm (*J* = 6.3 Hz, unlabeled) (see Table S5 and Figure S1 in the SI for details).

This anomalous behavior that may be attributable to the aromatic ring current effect can also be observed in its ^13^C{^1^H} NMR spectrum; the C4 atom appears as three signals: 74.97 ppm (D major), 75.02 ppm (D minor), and 75.09 ppm (unlabeled), also with intensities in accordance with the relative proportions (see Table S6 and Figure S3 in the SI for details).

This effect has not been detected in analogous monodeuterated 4-OBn compounds with a d-glucose configuration described in the literature.^51^ In an attempt to rationalize this unexpected NMR result, we prepared methyl 4-*O*-benzyl-6-*O*-*tert*-butyldiphenylsilyl-2,3-di-*O*-methyl-α-d-[4-O-PhCH-^2^H]galactopyranoside ([PhCH-^2^H]**97**) by the reaction of alcohol **96** with benzyl α-[^2^H]-4-methylbenzenesulfonate (Scheme 6).^52^ As expected for a d-sugar, [PhCH-^2^H]**97** adopts a ^4^*C*_1_ conformation, while [PhCH-^2^H]**31** exists preferentially in a ^1^*C*_4_ chair. Since the structures of d-galactose and l-fucose are in a pseudoenantiomeric relationship, 4-OBn would have a very similar stereochemical environment in [PhCH-^2^H]**97** to that which it has in the structure of [PhCH-^2^H]**31**. Indeed, in the NMR spectra of labeled [PhCH-^2^H]**97** (D/H, 7:1; dr = 1:1), it is also observed how both diastereoisomeric deuterated benzyl ethers affect the chemical displacement of the surrounding protons and carbons differently. For example, in the ^13^C{^1^H} NMR spectrum, the C4 atom analogously appears as three signals at 73.56 ppm (D_1_), 73.59 ppm (D_2_), and 73.64 ppm (unlabeled) with the expected intensities (see Table S6 and Figure S4 in the SI for details).

The effectiveness of this methodology was also tested on a d-pentose structure. Thus, the reaction with *n*-Bu_3_SnH/AIBN of 3-*C*-(2-*O*-diphenoxyphosphoryl-α,β-d-arabinopyranosyl)1-propoxyphthalimide derivatives **11** as a mixture of anomers and its deprotected diastereoisomeric pure β-diol **12** afforded exclusively the desired spirocycles **37** and **38**, respectively (Table 3, entries 7 and 10). The 2-deoxy-arabinopyranosyl ring adopted preferentially a ^1^*C*_4_ conformation (see Table S3 in the SI). Compound **38** was previously described by an alternative glycosylation method using thermodynamic conditions, and a 1*R* anomeric stabilized configuration was assigned.^28c^ Consequently, we have not found NOE interaction between H1’ and H3 and/or H5 as in previous thermodynamic spiroketals prepared in this work.

The analysis of the isotopic distribution in [2-^2^H]**37** (^2^H/^1^H, 1:1) and [2-^2^H]**38** (^2^H/^1^H, 2.3:1), obtained by reductive *n*-Bu_3_SnD/AIBN with or without the BF_3_•Et_2_O catalyst, showed a partial monodeuteration at C2, with the major isotopomer occupying the β-equatorial position (Table 3, entries 8, 9, and 11).

For the sake of completeness, this methodology was also extended to a series of furanosyl models derived from 3-*C*-(α-d-ribofuranosyl)1-propanol as described in Table 4. When the reaction of acetyl phthalimide **13** was carried out under the *n*-Bu_3_SnH(D)/AIBN conditions, no traces of any compound with a 1,6-dioxaspiro[4.4]nonane skeleton were detected. Only the alcohol **40** was obtained (Table 4, entries 1 and 2). The deuterium composition of [1-^2^H]**40** (^2^H/^1^H, 2:1) indicates that a significant 1,5-HAT reaction has taken place, but the C1-radical intermediate is reduced before the S–T rearrangement occurs. An equimolecular mixture of spirocycles [2-^2^H]**39** (^2^H/^1^H, 1.3:1) was achieved in moderate yield by adding a catalytic amount of BF_3_•Et_2_O to the reaction medium (Table 4, entry 3). Also in this case, a substantial loss of deuterium at C2 indicated the possibility of competitive mechanisms with the 1,2-β-(acyloxy)alkyl radical migration. A change to a better LG such as triflate **14** increased the rate of S–T rearrangement, and the sequence could now be completed under standard tin hydride conditions (Table 4, entries 5 and 6). However, adding BF_3_•Et_2_O to the reaction resulted in a very complex mixture containing alcohol **41** as the sole identifiable product. The initiation of the reaction under photoredox catalysis conditions on both phthalimides **13** and **14** afforded poorer results (Table 4, entries 4 and 8).

**Table 4:**
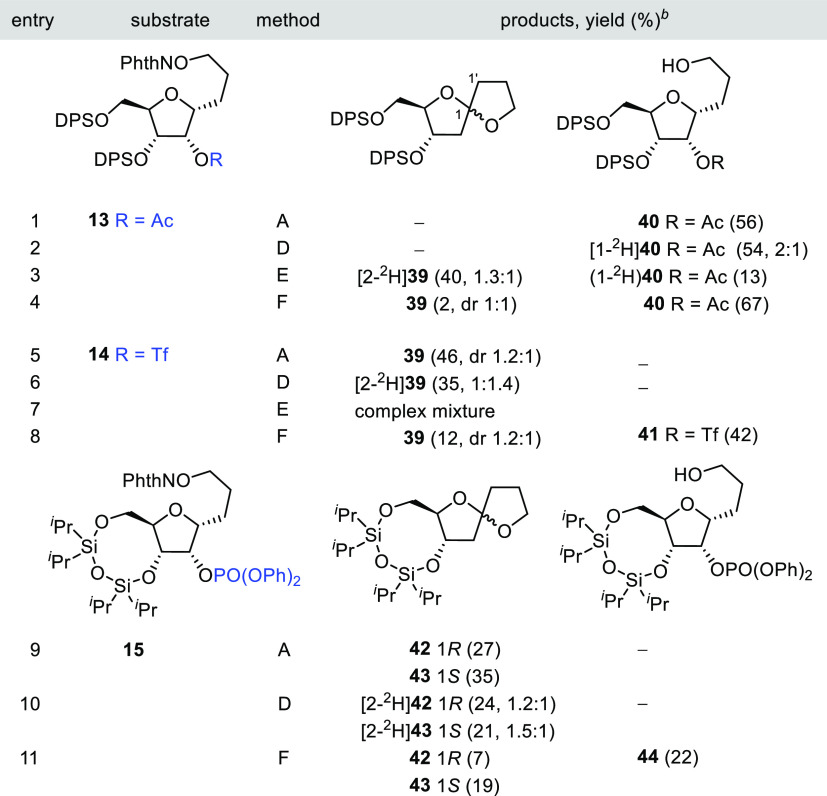
1,5-HAT–S–T Sequence in 3-*C*-(α-d-Rib*f*)1-propoxyphthalimides **13**–**15**^a^

a Reagents and conditions: method A: *n*-Bu_3_SnH (1 equiv), AIBN (0.1 equiv), PhCH_3_ (0.013 M), reflux; method D: *n*-Bu_3_SnD (1 equiv), AIBN (0.1 equiv), PhCH_3_ (0.013 M), reflux; method E: *n*-Bu_3_SnD (1 equiv), BF_3_•Et_2_O (0.2 equiv), AIBN (0.1 equiv), PhCH_3_ (0.013 M), reflux; and method F: Hantzsch ester (1.1 equiv), *fac*-Ir(ppy)_3_ (0.01 equiv), THF (0.007 M), rt, blue LED.

b Values in parentheses are isolate yields; deuterium incorporation (^2^H/^1^H) is included in partially labeled compounds. dr = diastereomeric ratio.

The use of diphenylphosphate as in **15** gave access exclusively to spirocycles **42** and **43**, isolated as a separable mixture of anomers in 62% overall yield (Table 4, entries 9 and 10). Again, under photoredox conditions, lower yields of the spirocyclic compounds and significant amounts of prematurely reduced alcohol **44** were obtained (Table 4, entry 11).

### Synthesis of 4-Deoxy-6,8-dioxabicyclo[3.2.1]octane Scaffolds

The objective of this section is the preparation of 4-deoxy carbohydrates with a 6,8-dioxabicyclo[3.2.1]octane skeleton by applying this 1,5-HAT–S–T rearrangement sequence to *C*-glycosyl compounds of a *C*-(α-d,l-glycopyranosyl)methanol general structure, and the results are included in Tables 5 and 6. The sequence was first attempted on the *C*-(4-*O*-acetyl-6-*O*-*tert*-butyldiphenylsilyl-2,3-di-*O*-methyl-α-d-glucopyranosyl)*N*-methoxyphthalimide (**16**) model. In this compound, the glucopyranosyl ring adopts preferentially a ^4^*C*_1_ chair conformation, and thus, the initial 1,5-HAT reaction should be favored (see Table S2 in the SI). However, the tin hydride conditions led to a mixture of four compounds, in which the desired bicycle **45** was isolated as a minor product. The other compounds were the unstable olefin **46** formed presumably by β-(acyloxy)alkyl fragmentation, and **47** and **48** generated by the premature reduction of intermediate radicals (Table 5, entry 1).

**Table 5:**
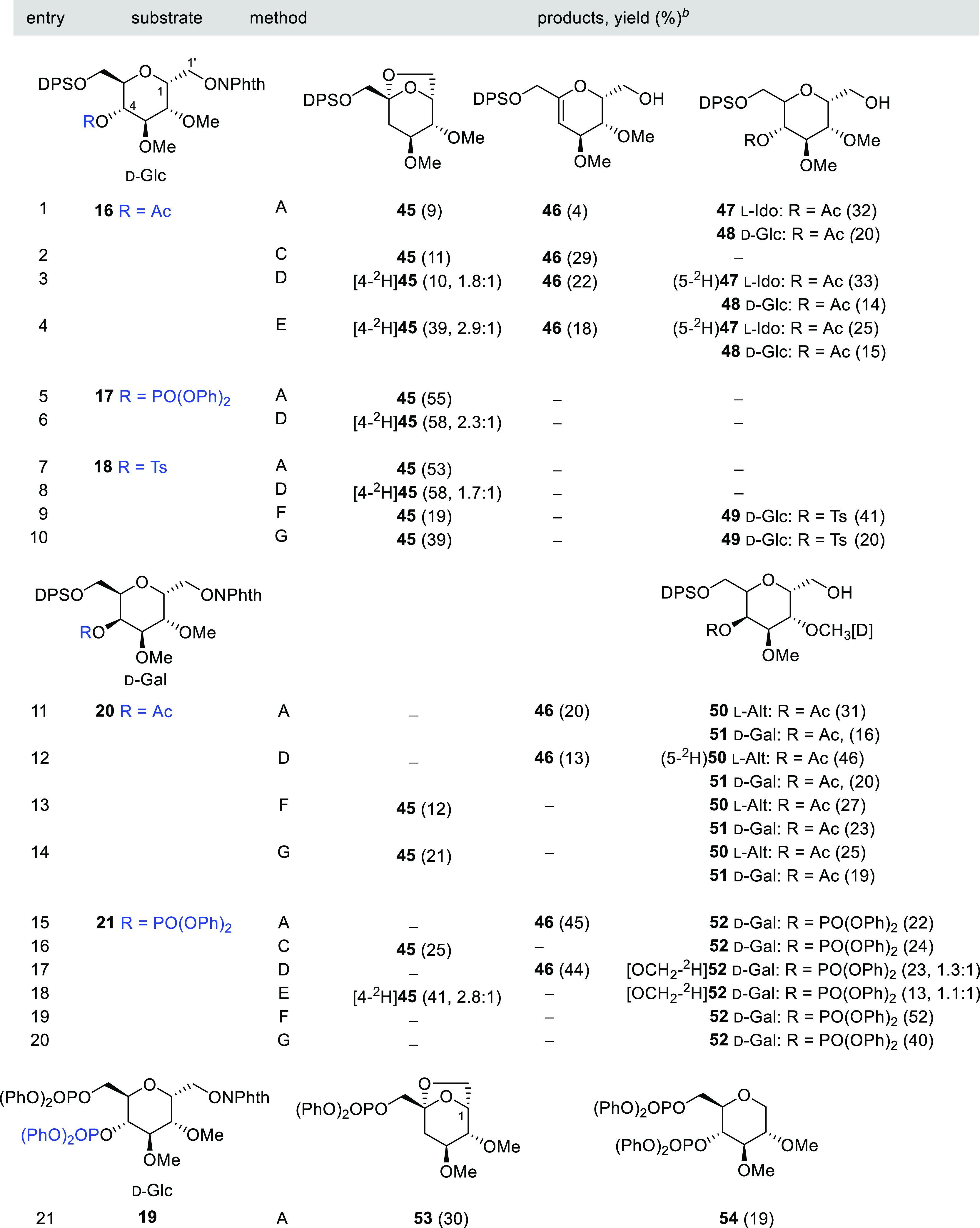
1,5-HAT–S–T Sequence in *C*-(α-d-Glc*p*)- and *C*-(α-d-Gal*p*)*N*-methoxyphthalimides **16**–**21**^a^

a Reagents and conditions: method A: *n*-Bu_3_SnH (1 equiv), AIBN (0.1 equiv), PhCH_3_ (0.013 M), reflux; method C: TTMSS (1 equiv), AIBN (0.1 equiv), PhCH_3_ (0.013 M), reflux; method D: *n*-Bu_3_SnD (1 equiv), AIBN (0.1 equiv), PhCH_3_ (0.013 M), reflux; method E: *n*-Bu_3_SnD (1 equiv), BF_3_•Et_2_O (0.2 equiv), AIBN (0.1 equiv), PhCH_3_ (0.013 M), reflux; method F: Hantzsch ester (1.1 equiv), *fac*-Ir(ppy)_3_ (0.01 equiv), THF (0.007 M), rt, blue LED; and method G: Hantzsch ester (0.37 equiv/h), *fac*-Ir(ppy)_3_ (0.01 equiv), THF (0.007 M), rt, blue LED.

b Values in parentheses are isolate yields; deuterium incorporation (^2^H/^1^H) is included in partially labeled compounds.

**Table 6:**
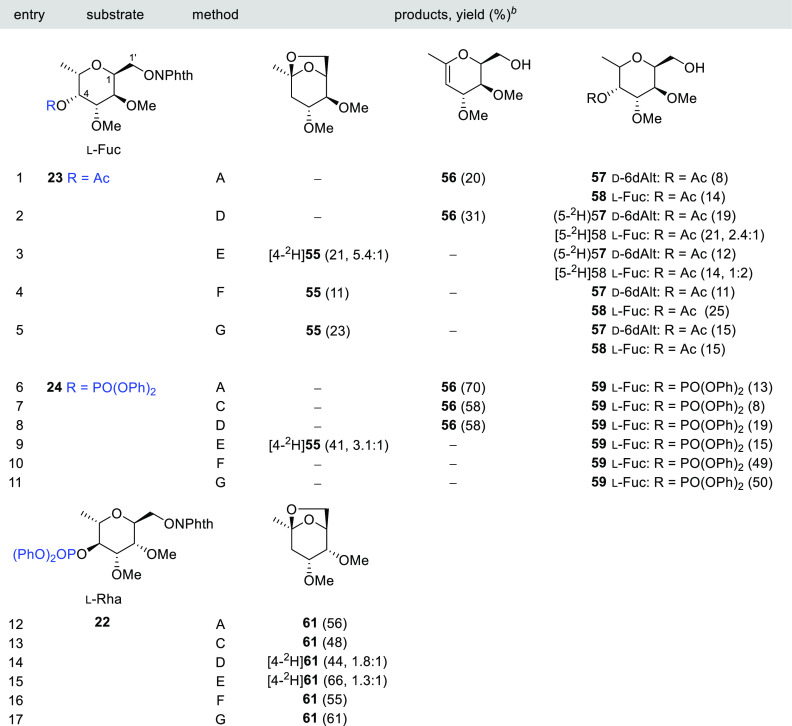
1,5-HAT–S–T Sequence in *C*-(α-l-Fuc*p*)- and *C*-(α-l-Rha*p*)*N*-methoxyphthalimides **22**–**24**^a^

a Reagents and conditions: method A: *n*-Bu_3_SnH (1 equiv), AIBN (0.1 equiv), PhCH_3_ (0.013 M), reflux; method C: TTMSS (1 equiv), AIBN (0.1 equiv), PhCH_3_ (0.013 M), reflux; method D: *n*-Bu_3_SnD (1 equiv), AIBN (0.1 equiv), PhCH_3_ (0.013 M), reflux; method E: *n*-Bu_3_SnD (1 equiv), BF_3_•Et_2_O (0.2 equiv), AIBN (0.1 equiv), PhCH_3_ (0.013 M), reflux; method F: Hantzsch ester (1.1 equiv), *fac*-Ir(ppy)_3_ (0.01 equiv), THF (0.007 M), rt, blue LED; and method G: Hantzsch ester (0.37 equiv/h), *fac*-Ir(ppy)_3_ (0.01 equiv), THF (0.007 M), rt, blue LED.

b Values in parentheses are isolate yields; deuterium incorporation (^2^H/^1^H) is included in partially labeled compounds.

The structural and stereochemical assignment of these compounds rests on spectroscopic and analytical data. Conformational evidence was obtained by extracting the ring *J*-coupling from a simulated spectrum (see Table S7 in the SI). In addition, the long-range couplings ^4^*J*_2,1’_ (1.1 Hz, calcd 1.1 Hz)^48^ observed in the spectrum of **45** and ^4^*J*_2,4_ (1.2 Hz, calcd 1.2 Hz)^48^ in **47** confirms the ^4^*C*_1_ and ^1^*C*_4_ conformations, respectively, for the sugar rings (see Table S4 in the SI). Therefore, the main product **47** was assigned an l-Ido structure by the inversion of configuration at C5. Using TTMSS or *n*-Bu_3_SnD as reductants did not significantly improve the yield of the bicycle **45** but markedly increased the formation of the olefin **46** (Table 5, entries 2 and 3). As expected, the best yield was achieved by adding Lewis acid; [4-^2^H]**45** (39%; ^2^H/^1^H, 2.9:1; 4*R*/4*S*, 1:1.2) was formed with a high deuterium content but low stereoselectivity (Table 5, entry 4).

As shown in Table 5 (entries 5–8), we need better LGs for the sequence of reactions to reach the end. In these experiments, the starting 4-*O*-diphenoxyphosphoryl **17** and 4-*O*-tosyl-phthalimide **18** were exclusively transformed into **45** or [4-^2^H]**45** and no prereduction or β-fragmentation byproducts were detected. Under photoredox catalysis conditions, tosyl derivative **18** gave the bicycle **45** in poor yield, which could be substantially enhanced by adding the Hantzsch ester slowly via a syringe pump (Table 5, entries 9 and 10).

The reaction of the models *C*-(6-*O*-*tert*-butyldiphenylsilyl-2,3-di-*O*-methyl-α-d-galactopyranosyl)*N*-methoxyphthalimide (**20** and **21**) was then examined, and the results are presented in Table 5. Under the *n*-Bu_3_SnH(D) conditions and irrespective of whether the starting phthalimide was **20** or **21**, no products with the 6,8-dioxabicyclo[3.2.1]octane skeleton were detected (Table 5, entries 11, 12, 15, and 17). In these experiments, the only relevant products isolated were olefin **46** and the l-altrose derivative **50**, both generated by the radical quenching at C5 prior to the S–T rearrangement. Best results were ultimately attained using *n*-Bu_3_SnD under the Lewis acid catalyst, with compounds [4-^2^H]**45** (41%; ^2^H/^1^H, 2.8:1; 4*R*/4*S*, 1:1.2) and [OCH_2_-^2^H]**52** being obtained (Table 5, entry 18). Analogously to the reaction of **16**, [4-^2^H]**45** was formed with a high deuterium incorporation at C4 but with a low stereoselectivity (Table 5, compare entries 4 and 18). The incorporation of deuterium in [OCH_2_-^2^H]**52** (^2^H/^1^H, 1.1:1) indicates a competitive abstraction of the H5 and the methoxyl group at C2 initiated by the alkoxyl radical through 1,5-HAT and 1,6-HAT processes, respectively. As would be expected, in this d-galactose model **21**, which is less prone to undergo the 1,2-(ester)alkyl radical migration, the photoredox catalytic reaction gave only the prereduced alcohol **52**, with compound **45** being undetectable by ^1^H NMR (Table 5, entries 19 and 20).

**Scheme 3:**
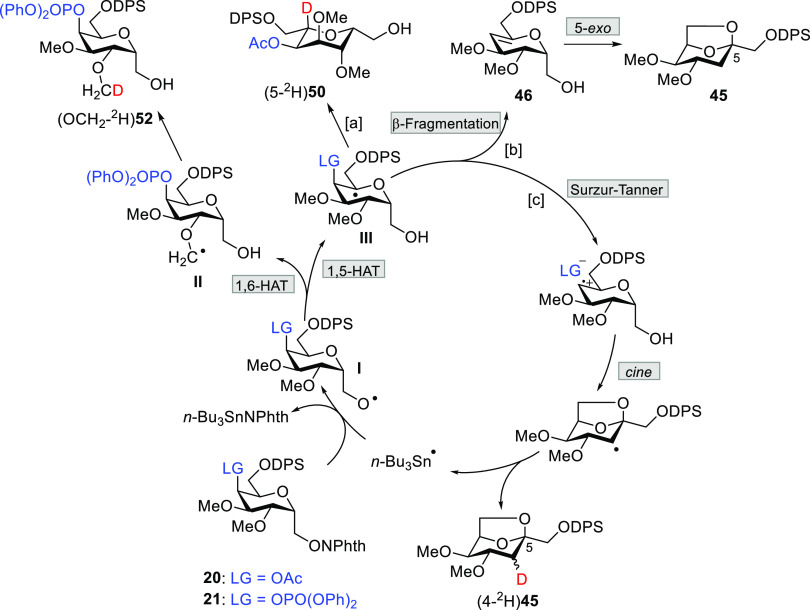
Propagation Cycle of *C*-(α-d-Gal*p*)methan-1-*O*-yl Radicals The prereduction of the alkoxyl radical and the initiation and termination steps are omitted for clarity.

A propagation cycle for the acetyl and diphenoxyphosphoryl (α-d-Gal*p*)methan-1-*O*-yl radical chain reactions, employing tin deuteride as the reductant, is shown in Scheme 3. The alkoxyl radical (I) initiated two competitive abstraction processes: 1,5-HAT of the 5H and 1,6-HAT of one hydrogen of the methoxyl group at C2. The radical II leads to (OCH_2_-^2^H)**52**, whereas the radical at C5 (III) may be stabilized by three different mechanisms: reduction with inversion of configuration giving rise to l-altrose derivative (5-^2^H)**50** (path a), radical β-fragmentation of the LG that can explain the formation of olefin **46** and the unlabeled **45** (path b),^45^ or continuing the sequence by the radical–ionic mechanism that finally provided (4-^2^H)**45** through the *cine* cyclization step (path c).

A phthalimide precursor **19** having two plausible LGs at C4 and C6 was also included in this study (Table 5, entry 21). Since the endocyclic alkene radical cation intermediate should be more stable than the exocyclic alternative, it is not surprising that we only obtained the 4-deoxy-bicycle **53** by C4-OPO(OPh)_2_ migration. This product was accompanied by d-glucitol derivative **54** as a result of the competitive β-fragmentation of the primary alkoxyl radical, which had not previously been observed in other members of this series.,^50c^^53^

Examples with l-sugar frameworks such as α-l-Fuc*p* (**23** and **24**) and α-l-Rha*p* (**22**) have also been accomplished, and the results are summarized in Table 6. Since α-l-Fuc*p* and α-d-Gal*p* have a pseudoenantiomeric relationship, an analogous reaction pattern could be expected. Indeed, the results obtained for the l-Fuc*p* derivatives (**23** and **24**) are quite similar to those observed for the previously studied d-Gal*p* phthalimides **20** and **21** (compare Table 5, entries 11–20 with Table 6, entries 1–11). Thus, neither the acetyl **23** nor diphenoxyphosphoryl **24** precursor gave the desired 6,8-dioxabicyclic compound when submitted to the *n*-Bu_3_SnH(D)/AIBN conditions in the absence of activating additives (Table 6, entries 1, 2, 6, and 8). In both cases, olefin **56** was the main product with a yield that reached a maximum of 70% using phosphatoxy as LG (Table 6, entry 6).

In the reaction of acetylphthalimide **23**, the olefin **56** was always accompanied by small amounts of **57** with an inverted 6-deoxy-d-altrose structure (Table 6, entries 1–5). The conversion of phthalimides **23** and **24** into 6,8-dioxabicyclic compound [4-^2^H]**55** was only possible in the presence of a catalytic amount of BF_3_•Et_2_O (Table 6, entries 3 and 9). Under these conditions, a new anhydro-alditol 3-*O*-acetyl-2,6-anhydro-1-deoxy-4,5-di-*O*-methyl-d-(6-^2^H)galactitol [(1-^2^H)**60**] was also isolated in a very low yield (3%), probably generated by the β-fragmentation of the alkoxyl radical at the beginning of the sequence (not shown in Table 6, entry 3). Parallel to what occurred for the d-Gal*p* derivatives **20** and **21**, surprisingly, under photoredox conditions, the acetyl precursor **23** afforded the 6,8-dioxabicyclo **55** although in low yields, while the diphenoxyphosphoryl precursor **24** yielded exclusively the reduced alcohol **59** (compare Table 6, entries 4, 5, 10, and 11 with Table 5, entries 13, 14, 19, and 20).

The phthalimides derived from α-l-Rha*p***22** and α-d-Glcp **17** have a very similar stereochemical arrangement to the atoms involved in the radical sequence, and consequently, an analogous behavior should be expected (compare Table 5, entries 5 and 6 with Table 6, entries 12 and 14). Indeed, the phthalimide **22** afforded exclusively the 6,8-dioxabicyclo **61** in good yield not only with tin hydride but also employing TTMSS or under the photoredox conditions.

## Conclusions

In summary, the fate of the 3-*C*-(α,β-d,l-glycopyranosyl)1-propan-*O*-yl radical moves through the *C*-glycosyl skeleton by a 1,5-HAT–S–T rearrangement radical/polar sequence giving 1,4-anhydro-5-deoxy-non-4-ulopyranoses with a 10-deoxy-1,6-dioxaspiro[4.5]decane structure.

The reaction under the tin hydride conditions appears to be reasonably independent of the axial or equatorial configuration of the abstractable 1H but is highly influenced by the nature and stereochemistry of the LGs (2-acetoxy or 2-phosphatoxy) used in the S–T rearrangement at the end of the sequence.^39^ Thus, the 2-phosphatoxy LG in an equatorial position as in 3-*C*-(2-*O*-diphenoxyphosphoryl-α,β-d-Glc*p*)1-propoxyphthalimides **2** and **4** was found to provide the best results (Table 1, entries 5–7 and 12–14). With a poorer 2-acetoxy LG axially disposed as in *C*-(2-*O*-acetyl-α,β-d-Man*p*)1-propoxyphthalimides **5** and **7**, the sequence did not reach the end and only the prereduced compound **29β** was obtained (Table 2, entries 1, 2, and 7–10). In the two intermediate situations—equatorial 2-acetoxy phthalimides **1** and **3** (Table 1, entries 1–4 and 8–11) and axial 2-diphenoxyphosphoryl phthalimides **6** and **8** (Table 2, entries 3–6 and 12–17)—the spirocycle **25** is formed in significant amounts, indicating that the low migratory capacity of the LG can be compensated by favorable stereochemical effects and vice versa. A comparison of the best results obtained with the different LGs has been summarized in Table S9 at the SI.

Some other interesting facts can be culled from the data described in Tables 1 and 2. First, an expected KIE was observed during the formation of **25**, with the yield increasing significantly in most cases when deuteride was used instead of hydride donors (e.g., Table 1, compare entries 8 and 11; see also Table S9 in the SI). The sequence yield was also dramatically improved by complexation with BF_3_•Et_2_O (Table 2, compare entries 10 and 11).

All these observations are in excellent agreement with experimental results obtained in the application of this methodology to *C*-glycopyranosyl models for the synthesis of either 10-deoxy-1,6-dioxaspiro[4.5]decane (Tables 1–3) or 4-deoxy-6,8-dioxabicyclo[3.2.1]octane frameworks (Tables 5–6). For example, 3-*C*-(α-l-Fuc*p*)1-propoxyphthalimides **9** and **10** with the pyranosyl ring in a ^1^*C*_4_ conformation and the 2-acetoxy and 2-phosphatoxy LGs equatorially disposed afforded the spiroketal **31** in good yield (Table 3, entries 1, 2 and 5, 6). Nevertheless, *C*-(α-d-Gal*p*)*N*-methoxyphthalimides **20** and **21** with the pyranosyl ring in a ^4^*C*_1_ conformation and the 4-acetoxy and 4-phosphatoxy LGs axially oriented did not give the expected bicyclic ketal **45**, which was achieved only after the addition of BF_3_•Et_2_O to the reaction media, as evidenced in the case of **21** (Table 5, entry 18). Also in this line, *C*-(α-l-Rha*p*)*N*-methoxyphthalimide **22** (^1^*C*_4_, 4-phosphatoxy equatorially positioned) smoothly led to the desired compound **61** (Table 6, entries 12–15), whereas *C*-(α-l-Fuc*p*)*N*-methoxyphthalimides **23** and **24** (^1^*C*_4_, axial LGs) reacted only under acid catalysis (Table 6, entries 3 and 9).

In the reaction of *C*-(d,l-Gly*p*)*N*-methoxyphthalimides, a new olefin with a *C*-(hex-4-enopyranosyl)methanol structure was formed (Tables 5 and 6), appearing exclusively when the S–T rearrangement is unfavored: with the 4-acetoxy group in the equatorial or axial disposition (compounds **16**, **20**, and **23**; 4–31%) or with the 4-phosphatoxy group axially oriented (compounds **21** and **24**; 44–70%). It is not detected in favored S–T rearrangements (4-phosphatoxy or 4-*p*-toluenesulfonyloxy equatorial) (compounds **17**, **18**, and **22**). Presumably due to the highly strained dioxabicyclo[3.2.1]octane system, a pure radical β-(ester)alkyl fragmentation competes, in some cases very favorably, with a radical-polar β-(ester)alkyl shift mechanism.

The results observed when the reaction is applied to d-pentoses deserve special comments (Tables 3 and 4). With 3-*C*-(2-*O*-diphenoxyphosphoryl-α,β-d-Ara*p*)1-propoxyphthalimides **11** and **12**, the reaction behaved analogously and the expected spiroketals **37** and **38** were, respectively, formed in similar yields (Table 3, entries 7–11). Notwithstanding, some differences with these trends are observed during the reaction of d-pentofuranosyl substrates. The reaction of 3-*C*-(2-acetyl-α-d-Rib*f*)1-propoxyphthalimide **13** proceeds exclusively in the presence of BF_3_•Et_2_O and the use of a better LG is necessary, as observed in compounds **14** and **15** (Table 4, entries 5–11).

In these more flexible five-membered rings, the configuration of the LGs does not appear to be as important. A pseudo-rotational analysis of compounds **13**, **14**, and **15** shows that the most populated conformers appear at phase angles of *P* = 354–9° (^3^*T*_2_) in the northern region of the pseudo-rotational itinerary, leaving the LG in a pseudo-axial configuration (see Table S8 in the SI for details).

When the sequences were initiated by visible light photocatalysis, low yields were observed in all 3-*C*-(α,β-d,l-Gly*p*)1-propoxyphthalimides, which were in general lower than those obtained with tin hydride (Tables 2 and 3, methods F and G). The spirocycles were always accompanied by high percentages of prereduced products. A similar behavior was observed in most cases of *C*-(d,l-Gly*p*)*N*-methoxyphthalimides. Thus, in the reaction of **21**, no traces of products resulting from the 1,5-HAT could be detected, with the prereduced alcohol **52** being formed exclusively (Table 5, entries 19 and 20). This means that, under these conditions, the six-membered TS of the 1,5-HAT cannot be reached probably due to conformational restrictions promoted by the bulky axially oriented diphenoxyphosphatoxy group. Paradoxically, with a poorer 2-acetoxy LG axially disposed as in *C*-(4-*O*-acetyl-α-d-Gal*p*)*N*-methoxyphthalimide **20**, the [3.2.1]bicyclic **45** and the inverted l-*altro* derivative **50** were obtained in a 46% combined yield (Table 5, entries 13 and 14). This is presumably due to the smaller steric demands of the acetoxy group. The same occurred with *C*-(α-l-Fuc*p*)*N*-methoxyphthalimides **23** and **24** with which **20** and **21** present a pseudoenantiomeric relationship (Table 6, entries 4, 5 and 10, 11). We have also noted that, under these photoredox conditions, no 4-enopyranosyl olefins (i.e., **46** or **56**) were detected (Tables 5–6, methods F and G).

In the best of situations, *C*-(4-*O*-diphenoxyphosphoryl-2,3-di-*O*-methyl-α-l-Rha*p*)*N*-methoxyphthalimide (**22**) (^1^*C*_4_, phosphatoxy equatorial) with the Hantzsch ester introduced slowly by a syringe pump, the bicycle **61** is produced in a yield (61%) comparable to that obtained with *n*-Bu_3_SnD/BF_3_•Et_2_O (Table 6, compare entries 15 and 17). These photocatalyzed reactions, carried out at room temperature, appear to be strongly influenced by the conformational equilibrium of the pyranosyl ring. However, under the tin hydride conditions (refluxing toluene, 110 °C), the TS required for the HAT reaction can be more readily attained.

### Preparation of 3-*C*-(Glycopyranosyl)1-propanol and 3-*C*-(Glycofuranosyl)1-propanol Models

**Scheme 4:**
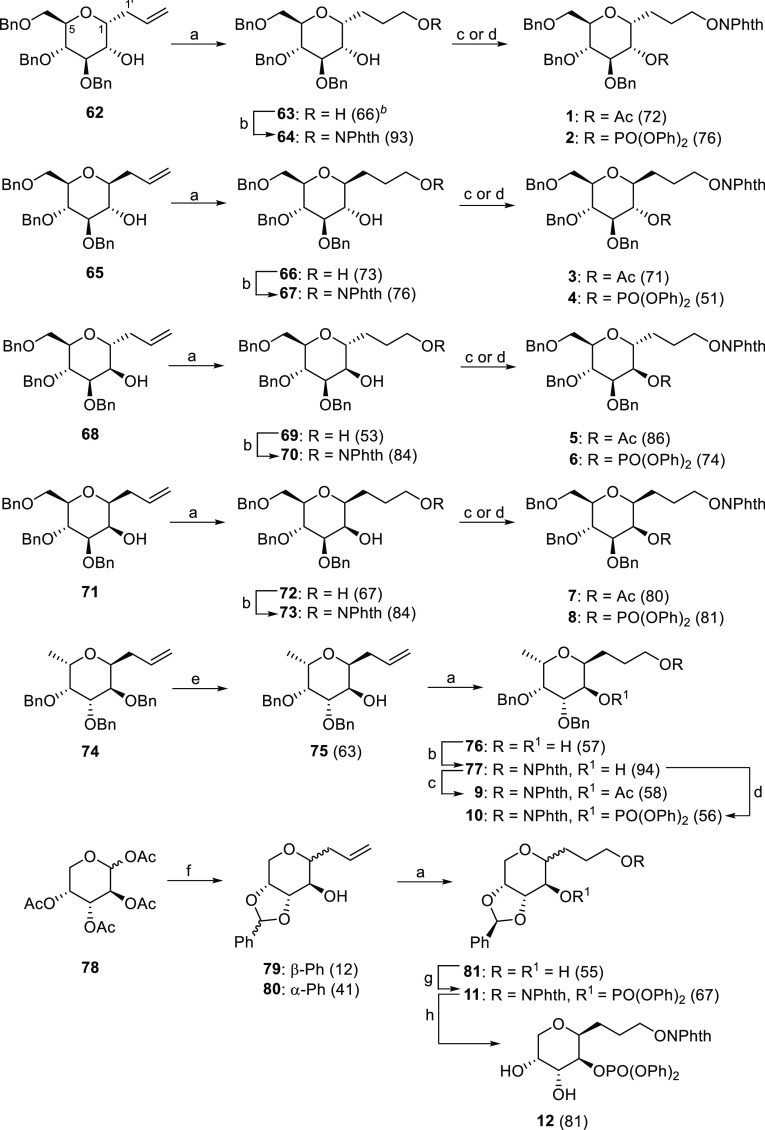
Synthesis of 3-*C*-(Glycopyranosyl)1-propoxyphthalimide (**1**–**12**) Precursors of 1,6-Dioxaspiro[4.5]decane Structures Reagents and conditions: (a) (*i*) BH_3_•THF 1 M complex, THF, 0 °C to rt, 1 h. (*ii*) NaOH 3 M, H_2_O_2_ 30%, 0 °C, 1 h. (b) HONPhth, Ph_3_P, DEAD, THF, 0 °C to rt, 1–4 h. (c) Ac_2_O, Py, DMAP, 0 °C to rt, 1 h. (d) ClPO(OPh)_2_, DMAP, CH_2_Cl_2_, 0 °C to rt, 2 h. (e) (*i*) I_2_, CH_2_Cl_2_, rt, 3 h. (*ii*) Zn dust, AcOH, Et_2_O:MeOH, rt, overnight. (f) (*i*) allyltrimethylsilane, BF_3_•Et_2_O, CH_3_CN, 0 °C to rt, 1.5 h. (*ii*) Na_2_CO_3_, MeOH, rt, 2.5 h. (*iii*) PhCH(OMe)_2_, CSA, DMF, rt, overnight. (g) (*i*) HONPhth, Ph_3_P, DEAD, THF, 0 °C to rt, 0.5 h. (*ii*) ClPO(OPh)_2_, DMAP, CH_2_Cl_2_, 0 °C to rt, 1.5 h. (h) TFA/H_2_O, CH_2_Cl_2_, 0 °C to rt, 1 h. Values in parentheses are isolate yields.

*C*-Glycosyl compounds of the 3-*C*-(α,β-d,l-glycopyranosyl)1-propene type **62**, **65**, **68**, **71**, and **75** were synthesized starting from perbenzylated d-glucose, d-mannose, or l-fucose, as required in each case, according to the procedure reported by Nicotra et al. (Scheme 4).^54^ Otherwise, for the d-arabinopyranose series, the allylation of **78** with allyltrimethylsilane and BF_3_•Et_2_O gave an inseparable anomeric mixture of allyl derivatives in 69% yield. The saponification of the acetyl groups and the selective acetal protection by treatment overnight with PhCH(OMe)_2_ and CSA gave access to β- and α-phenyl benzylidene substituted products as a mixture of anomers **79** (β/α, 3:1) and **80** (β/α, 3.5:1) with a free hydroxyl group at C2. Next, oxidative hydroboration of all the allyl compounds mentioned above gave efficiently the corresponding diols **63**, **66**, **69**, **72**, **76**, and **81**, whose primary hydroxyl groups were converted selectively to 3-*C*-(α,β-d,l-glycopyranosyl)*N*-propoxyphthalimides by the reaction with *N*-hydroxyphthalimide via Mitsunobu condensation yielding **64**, **67**, **70**, **73**, and **77**.^55^ There only remains the subsequent protection of the free secondary hydroxyl group as a good LG. We thus prepared the acetyl derivatives **1**, **3**, **5**, **7**, and **9** and the phenyl phosphates **2**, **4**, **6**, **8**, **10**, and **11**. Finally, acid hydrolysis of the benzylidene acetal in the diastereoisomeric mixture **11** provides, after chromatographic purification, the pure major β-diastereomer **12**.

**Scheme 5:**
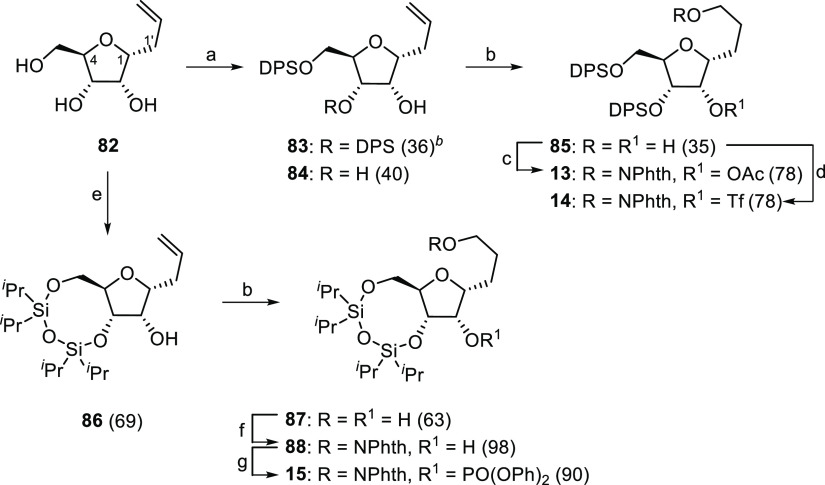
Synthesis of 3-*C*-(α-d-Ribofuranosyl)1-propoxyphthalimide (**13**–**15**) Precursors of 1,6-Dioxaspiro[4.4]nonane Structures Reagents and conditions: (a) DPSCl, imidazole, DMF, 0 °C, 0.5 h. (b) (*i*) BH_3_•THF 1 M complex, THF, 0 °C to rt, 2.5 h. (*ii*) NaHCO_3_, H_2_O_2_ 30%, 0 °C, 1 h. (c) (*i*) HONPhth, Ph_3_P, DEAD, 50 °C, 2 h. (*ii*) Ac_2_O, DMAP, Py, rt, 1 h. (d) (*i*) HONPhth, Ph_3_P, DEAD, 50 °C, 2 h. (*ii*) Tf_2_O, Py, rt, 1 h. (e) 1,3-dichloro-1,1,3,3-tetraisopropyldisiloxane, Py, 0 °C, 20 h. (f) HONPhth, Ph_3_P, DEAD, 50 °C, overnight. (g) ClPO(OPh)_2_, DMAP, CH_2_Cl_2_, rt, 3 h. Values in parentheses are isolate yields.

In the furanose series, we prepared the corresponding allyl ribose derivative **82** following a similar strategy to that described before for the d-arabinopyranose model (Scheme 5).^56^ Saponification of the acetyl groups and treatment of the corresponding triol with DPSCl and imidazole in dichloromethane at 0 °C produced the diprotected product **83** in 36% yield together with the diol **84** obtained in 40% yield.^13,57^ On the other hand, the reaction of **82** with 1,3-dichloro-1,1,3,3-tetraisopropyldisiloxane in dry pyridine afforded the monoalcohol **86** in 69% yield. Once again, oxidative hydroboration of **83** and **86** gave the corresponding diols **85** and **87**. The conversion of the primary alcohol to an *N*-alkoxyphthalimide and the introduction of an LG at C2 afforded the required models: the acetate **13**, triflate **14**, and phenyl phosphate **15**.

### Preparation of *C*-(Glycopyranosyl)methanol Models

**Scheme 6:**
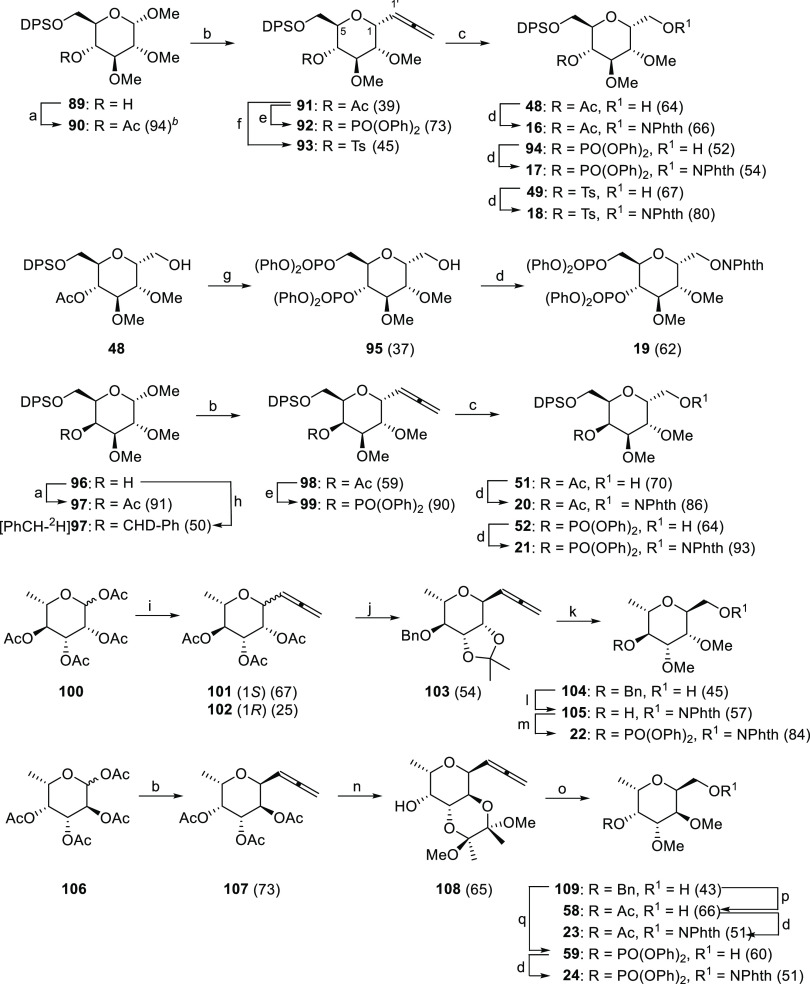
Synthesis of *C*-(Glycopyranosyl)*N*-methoxyphthalimide Precursors of 6,8-Dioxabicyclo[3.2.1]heptane Structures Reagents and conditions: (a) Ac_2_O, Py, DMAP, rt, 0.5–1.5 h. (b) (*i*) propargyl trimethylsilane/Et_2_O 39% v/v, TMSOTf, CH_3_CN, sonication, rt, 1.5–3 h. (*ii*) DPSCl, imidazole, DMF, 0 °C, 2 h. (c) (*i*) O_3_, CH_2_Cl_2_–MeOH, −78 °C. (*ii*) NaBH_4_, 0 °C to rt, 1–3 h. (d) HONPhth, Ph_3_P, DEAD, 0 °C, 1.5 h–overnight. (e) (*i*) K_2_CO_3_, MeOH, rt, overnight. (*ii*) ClPO(OPh)_2_, DMAP, CH_2_Cl_2_, rt, 2.5–7 h. (f) (*i*) K_2_CO_3_, MeOH, rt, overnight. (*ii*) TsCl, Py, rt, overnight. (g) (*i*) DHP, *p*-TsOH•H_2_O, CH_2_Cl_2_, rt, 2 h. (*ii*) TBAF/THF 1 M, THF, rt, 3 h. (*iii*) K_2_CO_3_, MeOH, rt, 4 h. (*iv*) ClPO(OPh)_2_, Py, rt, overnight. (h) NaH 60%, *p*-TsO-CHD-Ph, DMF/CH_2_Cl_2_, rt, 1 h. (i) BF_3_•OEt_2_, TMSOTf, propargyl trimethylsilane, CH_3_CN, 0 °C to rt, 15 h. (j) (*i*) K_2_CO_3_, MeOH, rt, 3 h. (*ii*) 2,2-dimethoxypropane, *p*-TsOH•H_2_O, acetone, rt, 3 h. (*iii*) NaH 60%, BnBr, DMF, 0 °C, 3 h. (k) (*i*) TFA/H_2_O, rt, 2 h. (*ii*) NaH 60%, MeI, DMF, 0 °C, 1.5 h. (*iii*) O_3_, CH_2_Cl_2_–MeOH, −78 °C. (*iv*) NaBH_4_, 0 °C to rt, 0.75 h. (l) (*i*) H_2_, Pd/C 10%, EtOAc, rt, overnight. (*ii*) HONPhth, Ph_3_P, DEAD, 0 °C to rt, 3.5 h. (m) ClPO(OPh)_2_, DMAP, CH_2_Cl_2_, rt, 2 h. (n) (*i*) K_2_CO_3_, MeOH, rt, 3 h. (*ii*) 2,3-butanedione, (MeO)_3_CH, BF_3_•Et_2_O, MeOH, 60 °C, 4.5 h. (o) (*i*) NaH 60%, BnBr, DMF, 0 °C, 2 h. (*ii*) TFA/H_2_O, 40 °C, overnight. (*iii*) NaH 60%, MeI, DMF, 0 °C to rt, 2 h. (*iv*) O_3_, CH_2_Cl_2_–MeOH, −78 °C. (*v*) NaBH_4_, 0 °C to rt, 1 h. (p) (*i*) DPSCl, imidazole, DMF, rt, 3 h. (*ii*) H_2_, Pd/C 10%, EtOAc, rt, overnight. (*iii*) Ac_2_O, Py, DMAP, rt, 0.5 h. (*iv*) TBAF/THF, 1 M, THF, rt, 4 h. (q) (*i*) DPSCl, imidazole, DMF, rt, 3 h. (*ii*) H_2_, Pd/C 10%, EtOAc, rt, overnight. (*iii*) ClPO(OPh)_2_, DMAP, CH_2_Cl_2_, rt, 3.5 h. (*iv*) TBAF/THF, 1 M, THF, rt, 3.5 h. Values in parentheses are isolate yields.

The synthesis of 6,8-dioxabicyclo[3.2.1]heptane scaffolds commenced with the preparation of *C*-(4-*O*-acetyl-α-d,l-glycopyranosyl)allenes **91**, **98**, **101**, **102**, and **107** (Scheme 6). To achieve this with high α-diastereoselectivity, we employed the ultrasound-assisted *C*-glycosylation described by Murphy et al. using propargyl trimethylsilane and a Lewis acid catalyst.^58^ Next, LG was interchanged from OAc to PO(OPh)_2_, yielding **92** and **99**, and to the tosyl group, giving access to **93**, by saponification of the acetyl group and treatment with the corresponding acid chloride in basic media. Subsequent reductive ozonolysis afforded the *C*-(α-glycopyranosyl)methanol derivatives **48**, **94**, **49**, **51**, and **52** in good yields. Product **48** was also used as a precursor to prepare a diphosphate substrate **95** to analyze whether competitive migrations of the LGs at C4 and C6 could occur. First, it was necessary to protect the primary C1’-alcohol as a tetrahydropyranyl (THP) ether; then removal of both the silyl and the acyl protectors gave a diol, which was transformed to a diphosphate by treatment with ClPO(OPh)_2_ in pyridine overnight. Acid hydrolysis of the THP protector afforded **95** in a 37% overall yield (four-step). Finally, Mitsunobu condensation of all the primary alcohols mentioned above with *N*-hydroxyphthalimide yielded *C*-(α-glycopyranosyl)*N*-methoxyphthalimide derivatives **16**, **17**, **18**, **19**, **20**, and **21** in good to excellent yields.

In the case of the l-rhamnose **101** and l-fucose **107** derivatives, it was found necessary to hydrolyze the acetyl groups at C2, C3, and C4 to protect selectively the C2 and C3 hydroxyl groups as cyclic acetals to enable the ulterior introduction of the LG at C4. Once the isopropylidene group was introduced for the l-rhamnose derivative, benzylation of the free alcohol at C4 afforded **103** in 54% yield. Acid hydrolysis of the transitory acetal assembly, methylation of both C2 and C3 hydroxyl groups, and reductive ozonolysis gave monoalcohol **104** in 45% overall yield. Afterward, palladium-catalyzed hydrogenolysis of the benzyl protective group gave the corresponding diol that was subsequently transformed into the *N*-alkoxyphthalimide **105** in 57% yield. The corresponding phenyl phosphate **22** was obtained efficiently after 2 h by treatment with ClPO(OPh)_2_ and DMAP at room temperature in dichloromethane.

For the l-fucose derivative, butane 2,3-bisacetal protection^59^ was selected to obtain **108** in 65% yield. Next, a similar strategy as described for the previous model was employed to afford monoalcohol **109** in 43% overall yield. Transient protection of the primary alcohol as a DPS ether allows the hydrogenolysis of the benzyl ether and the introduction of the LG at C4. Therefore, the formation of the acetate or phenyl phosphate followed by DPS removal (TBAF) allowed the generation of **58** and **59** in good overall yields*.* The conversion of the corresponding primary alcohols to *N*-alkoxyphthalimides occurred in 51% yield for both substrates to generate **23** and **24**, respectively.

## Experimental Section

### General Information

Commercially available reagents and solvents were analytical grade or were purified by standard procedures prior to use. Solvents for starting material preparation and radical reactions were dried before use. The spray reagents for TLC analysis were conducted with 0.5% vanillin in H_2_SO_4_–EtOH (4:1) or, in some specific cases, with the Pancaldi reagent {(NH_4_)_6_MoO_4_, Ce(SO_4_)_2_, H_2_SO_4_, H_2_O}^60^ and further heating until the development of color. Melting points were determined with a hot-stage apparatus. Optical rotations were measured at the sodium line at the ambient temperature in CHCl_3_ solutions. IR spectra were measured as thin films on CHCl_3_ solutions. NMR spectra were determined at 500 or 400 MHz for ^1^H and at 125.7 or 100 MHz for ^13^C{^1^H} in CDCl_3_ or C_6_D_6_ as stated. The chemical shifts are given in parts per million (ppm) relative to TMS at δ 0.00 ppm or to residual CDCl_3_ at δ 7.26 ppm for proton spectra and relative to CDCl_3_ at δ 77.00 ppm for carbon spectra. NMR spectra were assigned with the aid of 1D and 2D techniques, including ^13^C DEPT-135, COSY, HSQC, HMBC, and NOESY. The DAISY program as implemented in the TopSpin 4.0.6 software package was used for the simulation of ^1^H NMR spectra. Low- and high-resolution mass spectra were recorded by using an electrospray (ESI^+^) and TOF analyzer. Flash column chromatography was performed on a Merck silica gel 60 PF (0.063–0.2 mm). For the chromatography of the radical reactions with *n*-Bu_3_SnH or *n*-Bu_3_SnD, 10% KF was added and mixed with the silica gel. Circular layers of 1 and 2 mm of the Merck silica gel 60 PF_254_ were used on a Chromatotron for centrifugally assisted chromatography. HPLC separations were undertaken using a semipreparative (10 × 250 mm) Ascentis Si normal-phase column. An ultrasonic bath was used (2510E-DTH, Branson) for the synthesis of the allene precursors and for the deoxygenation of the THF for the photocatalytic reactions. Photochemical reactions were carried out with 15 W blue LEDs (468 nm peak wavelength, 25 nm spectral half-wave width, composed of 15 LED units each with 1 W, 3 V, 300 mA, 5 cm distance from the light source to the irradiation vessel). For convenience, the atom-numbering system used along this section and in the assignments of the Experimental Section corresponds to the one depicted in structures of the schemes and tables, although an IUPAC systematic nomenclature has been used throughout this paper. The IUPAC nomenclature for deuterated carbohydrates (2-Carb-16.6, with the parentheses indicating substitution and square brackets for partial labeling) has been used throughout the manuscript.

### General Methods for Radical and Photoredox Reactions (Tables 1–6)

#### Method A*:* Fast Addition of *n-*Bu_3_SnH

A solution of the phthalimide (1 mmol) in dry toluene (75 mL) was treated with *n-*Bu_3_SnH (269 μL, 1 mmol) and AIBN (16.4 mg, 0.1 mmol) and heated under reflux. Every hour, the same quantity of AIBN was added. In some cases, a supplementary addition of *n-*Bu_3_SnH (269 μL, 1 mmol) was required as indicated. When all the starting material was consumed, the reaction mixture was directly poured into a column chromatography on a silica gel with 10% KF (hexanes to hexanes–EtOAc) to give the corresponding products.

#### Method B*:* Slow Addition of *n-*Bu_3_SnH (1 equiv/h)

A solution of the phthalimide (1 mmol) in dry toluene (75 mL) was treated with AIBN (16.4 mg, 0.1 mmol), and *n-*Bu_3_SnH (269 μL, 1 mmol) was dropwise added during 1 h by means of a syringe pump under reflux. Every hour, the same quantity of AIBN was added. In some cases, a supplementary addition of *n-*Bu_3_SnH (269 μL, 1 mmol) was required as indicated. When all the starting material was consumed, the reaction mixture was directly poured into a column chromatography on a silica gel with 10% KF (hexanes to hexanes–EtOAc) to give the corresponding products.

#### Method C*:* Fast Addition of TTMSS

A solution of the phthalimide (1 mmol) in dry toluene (75 mL) was treated with AIBN (16.4 mg, 0.1 mmol) and TTMSS (308.5 μL, 1 mmol) and heated under reflux. Every hour, the same quantity of AIBN was added. In some cases, a supplementary addition of TTMSS (308.5 μL, 1 mmol) was required as indicated. When all the starting material was consumed, the reaction mixture was evaporated and purified by column chromatography (hexanes–EtOAc) to give the corresponding products.

#### Method D*:* Fast Addition of *n-*Bu_3_SnD

A solution of the phthalimide (1 mmol) in dry toluene (75 mL) was treated with *n-*Bu_3_SnD (270.4 μL, 1 mmol) and AIBN (16.4 mg, 0.1 mmol) and heated under reflux. Every hour, the same quantity of AIBN was added. In some cases, a supplementary addition of *n-*Bu_3_SnD (270.4 μL, 1 mmol) was required as indicated. When all the starting material was consumed, the reaction mixture was directly poured into a column chromatography on a silica gel with 10% KF (hexanes to hexanes–EtOAc) to give the corresponding products.

#### Method E*:* Fast Addition of *n-*Bu_3_SnD and BF_3_•Et_2_O

A solution of the phthalimide (1 mmol) in dry toluene (75 mL) was treated with *n-*Bu_3_SnD (270.4 μL, 1 mmol), BF_3_•Et_2_O (24.7 μL, 0.2 mmol), and AIBN (16.4 mg, 0.11 mmol) and heated at 100 °C. Every hour, the same quantity of AIBN was added. In some cases, a supplementary addition of *n-*Bu_3_SnD (270.4 μL, 1 mmol) and BF_3_•Et_2_O (24.7 μL, 0.2 mmol) was required as indicated. When all the starting material was consumed, the reaction mixture was directly poured into a column chromatography on a silica gel with 10% KF (hexanes to hexanes–EtOAc) to give the corresponding products.

#### Method F: Photoredox Conditions

A deoxygenated solution of the phthalimide (1 mmol), Hantzsch ester (278.6 mg, 1.1 mmol), and *fac*-Ir(ppy)_3_ (6.5 mg, 0.01 mmol) in dry THF (148.7 mL) was placed in a Schlenk tube under nitrogen and irradiated with blue LEDs at room temperature. The reaction mixture was concentrated and purified directly by chromatotron (hexanes–EtOAc) to give the corresponding products.

#### Method G: Photoredox Conditions with Slow Addition of Hantzsch Ester

A deoxygenated solution of the phthalimide (1 mmol) and *fac*-Ir(ppy)_3_ (6.5 mg, 0.01 mmol) in dry THF (116 mL) was placed in a Schlenk tube under nitrogen and irradiated with blue LEDs at room temperature. A solution of Hantzsch ester (279.1 mg, 1.1 mmol) in dry THF (34.9 mL) was then slowly added with a syringe pump over a period of 3 h. The reaction mixture was concentrated and purified directly by chromatotron (hexanes–EtOAc, 8:2 to 1:1) to give the corresponding products.

### Synthesis of 10-Deoxy-1,6-dioxaspiro[4.5]decane Structures (Tables 1–3)

#### Radical Reactions of **1**

##### Method A

Following the general procedure, starting from substrate **1** (62.8 mg, 0.092 mmol), after 2 h of reaction, a supplementary addition of *n-*Bu_3_SnH (25 μL, 0.092 mmol) was required. All the starting material was consumed after 3 h. Column chromatography (hexanes to hexanes–EtOAc, 1:1) gave (4*S*)-1,4-anhydro-6,7,9-tri-*O*-benzyl-2,3,5-trideoxy-d-*arabino*-non-4-ulopyranose (**25**) (18.6 mg, 0.039 mmol, 43%) as an amorphous solid, 3-*C*-(2-*O*-acetyl-3,4,6-tri-*O*-benzyl-β-d-glucopyranosyl)1-propanol (**26β**) (6.9 mg, 0.013 mmol, 14%) as a colorless oil, and 3-*C*-(2-*O*-acetyl-3,4,6-tri-*O*-benzyl-α-d-glucopyranosyl)1-propanol (**26α**) (4.1 mg, 0.008 mmol, 8%) as an amorphous solid. Compound **25**: [α]_D_ = +40.0 (*c =* 0.18, CHCl_3_). ^1^H NMR (500 MHz, CDCl_3_, simulated ring coupling constants using DAISY) δ_H_ 7.34–7.18 (m, 15H, Ar), 4.89 (d, *J* = 11.1 Hz, 1H, OBn), 4.66 (d, *J* = 11.7 Hz, 1H, OBn), 4.62 (d, *J* = 11.7 Hz, 1H, OBn), 4.61 (d, *J* = 12.3 Hz, 1H, OBn), 4.54 (d, *J* = 11.0 Hz, 1H, OBn), 4.51 (d, *J* = 12.3 Hz, 1H, OBn), 3.990 (ddd, *J* = 11.5, 8.9, 5.1 Hz, 1H, 3-H), 3.89 (ddd, *J* = 8.2, 8.2, 5.4 Hz, 1H, 3’-H_b_), 3.83 (ddd, *J* = 8.2, 8.2, 6.3 Hz, 1H, 3’-H_a_), 3.79 (ddd, *J* = 9.9, 4.3, 1.9 Hz, 1H, 5-H), 3.74 (dd, *J* = 10.8, 4.3 Hz, 1H, 6-H_b_), 3.64 (dd, *J* = 10.8, 1.9 Hz, 1H, 6-H_a_), 3.57 (dd, *J* = 9.9, 8.9 Hz, 1H, 4-H), 2.222 (dd, *J* = 12.7, 5.1 Hz, 1H, 2-H_b_), 2.12–2.00 (m, 2H, 1’-H_b_, 2’-H_b_), 1.872 (dd, *J* = 12.7, 11.5 Hz, 1H, 2-H_a_), 1.85 (m, 1H, 2’-H_a_), 1.76 ppm (m, 1H, 1’-H_a_). ^13^C{^1^H} NMR (100.6 MHz, CDCl_3_) δ_C_ 138.9 (2 × C, Ar), 138.5 (C, Ar), 128.34 (2 × CH, Ar), 128.28 (2 × CH, Ar), 128.2 (2 × CH, Ar), 127.76 (2 × CH, Ar), 127.74 (2 × CH, Ar), 127.6 (2 × CH, Ar), 127.5 (2 × CH, Ar), 127.4, (CH, Ar), 106.33 (C, C-1), 79.2 (CH, C-4), 78.51 (CH, C-3), 74.7 (CH_2_, OBn), 73.3 (CH_2_, OBn), 71.8 (CH_2_, OBn), 71.8 (CH, C-5), 69.4 (CH_2_, C-6), 67.3 (CH_2_, C-3’), 38.62 (CH_2_, C-2), 37.2 (CH_2_, C-1’), 23.5 ppm (CH_2_, C-2’). IR (CHCl_3_): ν = 3009, 2939, 1456, 1089 cm^–1^. MS (ESI) *m*/*z* (%) = 497 (100) [M + Na]^+^. HRMS (ESI) *m*/*z:* [M + Na]^+^ calcd for C_30_H_34_NaO_5_ 497.2304; found 497.2303. Anal. calcd for C_30_H_34_O_5_: C, 75.92; H, 7.12. Found: C, 76.07; H, 7.21. Compound **26β**: [α]_D_ = +12.3 (*c =* 0.43, CHCl_3_). ^1^H NMR (500 MHz, CDCl_3_) δ_H_ 7.34–7.15 (m, 15H, Ar), 4.887 (dd, *J* = 9.3, 9.3 Hz, 1H, 2-H), 4.81 (d, *J* = 11.4 Hz, 1H, OBn), 4.77 (d, *J* = 10.9 Hz, 1H, OBn), 4.65 (d, *J* = 11.4 Hz, 1H, OBn), 4.59 (d, *J* = 12.2 Hz, 1H, OBn), 4.53 (d, *J* = 12.2 Hz, 1H, OBn), 4.52 (d, *J* = 10.6 Hz, 1H, OBn), 3.68 (dd, *J* = 10.9, 1.9 Hz, 1H, 6-H_b_), 3.66–3.59 (m, 5H, 3’-H_2_, 3-H, 4-H, 6-H_a_), 3.46 (m, 1H, 5-H), 3.34 (ddd, *J* = 9.8, 9.8, 2.4 Hz, 1H, 1-H), 1.94 (s, 3H, OAc), 1.75–1.61 (m, 3H, 1’-H_b_, 2’-H_2_), 1.509 ppm (m, 1H, 1’-H_a_), 1H from OH is missing. ^13^C{^1^H} NMR (100.6 MHz, CDCl_3_) δ_C_ 169.9 (C, OAc), 138.4 (C, Ar), 138.1 (C, Ar), 138.0 (C, Ar), 128.42 (2 × CH, Ar), 128.41 (2 × CH, Ar), 128.37 (2 × CH, Ar), 128.0 (2 × CH, Ar), 127.8 (3 × CH, Ar), 127.70 (2 × CH, Ar), 127.67 (CH, Ar), 127.6 (CH, Ar), 84.7 (CH, C-3), 79.0 (CH, C-4), 78.5 (CH, C-5), 78.0 (CH, C-1), 75.2 (CH_2_, OBn), 75.0 (CH_2_, OBn), 73.81 (CH, C-2), 73.5 (CH_2_, OBn), 69.1 (CH_2_, C-6), 62.7 (CH_2_, C-3’), 28.84 (CH_2_, C-2’), 28.24 (CH_2_, C-1’), 20.9 ppm (CH_3_, OAc). IR (CHCl_3_): ν = 3430, 3014, 1738, 1229, 1039 cm^–1^. MS (ESI) *m*/*z* (%) = 557 (100) [M + Na]^+^. HRMS (ESI) *m*/*z:* [M + Na]^+^ calcd for C_32_H_38_NaO_7_ 557.2515; found 557.2513. Anal. calcd for C_32_H_38_O_7_: C, 71.89; H, 7.16. Found: C, 71.86; H, 7.37. Compound **26α**: [α]_D_ = +41.4 (*c =* 0.36, CHCl_3_). ^1^H NMR (500 MHz, CDCl_3_) δ_H_ 7.34–7.14 (m, 15H, Ar), 5.03 (dd, *J* = 8.5, 5.4 Hz, 1H, 2-H), 4.75 (d, *J* = 11.7 Hz, 1H, OBn), 4.70 (d, *J* = 11.0 Hz, 1H, OBn), 4.70 (d, *J* = 11.0 Hz, 1H, OBn), 4.59 (d, *J* = 12.0 Hz, 1H, OBn), 4.51 (d, *J* = 12.0 Hz, 1H, OBn), 4.48 (d, *J* = 11.1 Hz, 1H, OBn), 4.13 (m, 1H, 1-H), 3.81 (dd, *J* = 8.2, 8.2 Hz, 1H, 3-H), 3.75 (ddd, *J* = 8.2, 3.8, 3.8 Hz, 1H, 5-H), 3.71–3.63 (m, 4H, 3’-H_2_, 6-H_2_), 3.60 (dd, *J* = 8.2, 8.2 Hz, 1H, 4-H), 1.99 (s, 3H, OAc), 1.87 (br s, 1H, OH), 1.80 (m, 1H, 1’-H_b_), 1.70–1.58 (m, 2H, 2’-H_2_), 1.51 ppm (m, 1H, 1’-H_a_). ^13^C{^1^H} NMR (100.6 MHz, CDCl_3_) δ_C_ 170.0 (C, OAc), 138.3 (C, Ar), 138.0 (2 × C, Ar), 127.6–128.4 (15 × CH, Ar), 79.9 (CH, C-3), 77.5 (CH, C-4), 74.6 (CH_2_, OBn), 74.5 (CH_2_, OBn), 73.4 (CH_2_, OBn), 73.0 (CH, C-2), 72.4 (CH, C-1), 72.0 (CH, C-5), 69.1 (CH_2_, C-6), 62.2 (CH_2_, C-3’), 28.9 (CH_2_, C-2’), 22.5 (CH_2_, C-1’), 20.9 ppm (CH_3_, OAc). IR (CHCl_3_): ν = 3477, 3014, 2942, 1740, 1236, 1100 cm^–1^. MS (ESI) *m*/*z* (%) = 557 (100) [M + Na]^+^. HRMS (ESI) *m*/*z:* [M + Na]^+^ calcd for C_32_H_38_NaO_7_ 557.2515; found 557.2515. Anal. calcd for C_32_H_38_O_7_: C, 71.89; H, 7.16. Found: C, 71.57; H, 7.51.

##### Method B

Following the general procedure, starting from substrate **1** (123.9 mg, 0.18 mmol), after 2 h of reaction, two more equivalents of *n-*Bu_3_SnH (98 μL, 0.36 mmol) added by a syringe pump were required. All the starting material was consumed after 14 h. Column chromatography (hexanes to hexanes–EtOAc, 1:1) gave **25** (43.4 mg, 0.09 mmol, 50%) and **26α** (14.1 mg, 0.026 mmol, 15%).

##### Method C

Following the general procedure, starting from substrate **1** (62.8 mg, 0.092 mmol), after 2 h of reaction, a supplementary addition of TTMSS (28.5 μL, 0.092 mmol) was required. All the starting material was consumed after 5 h. Column chromatography (hexanes–EtOAc, 9:1 to 6:4) gave **25** (8.4 mg, 0.018 mmol, 19%) and the reduced product **26α** (1.5 mg, 0.003 mmol, 3%).

##### Method D

Following the general procedure, starting from substrate **1** (94 mg, 0.14 mmol), after 2 h of reaction, a supplementary addition of *n-*Bu_3_SnD (37 μL, 0.14 mmol) was required. All the starting material was consumed after 5 h. Column chromatography (hexanes to hexanes–EtOAc, 1:1) gave (4*S*)-1,4-anhydro-6,7,9-tri-*O*-benzyl-2,3,5-trideoxy-d-(5-^2^H)*arabino*-non*-*4-ulopyranose [(2-^2^H)**25**] (28.5 mg, 0.060 mmol, 43%, 2β-^2^H/2α-^2^H, 7:3) as an amorphous solid, 3-*C*-(2-*O*-acetyl-3,4,6-tri-*O*-benzyl-β-d-(1-^2^H)glucopyranosyl)1-propanol [(1-^2^H)**26β**] (4.8 mg, 0.009 mmol, 6%) as a colorless oil, and the prematurely reduced product **26α** (3 mg, 0.006 mmol, 4%). Compound (2-^2^H)**25**: ^1^H NMR (400 MHz, CDCl_3_, simulated ring coupling constants using DAISY) δ_H_ 7.35–7.18 (m, 15H, Ar). 4.89 (d, *J* = 11.1 Hz, 1H, OBn), 4.66 (d, *J* = 11.7 Hz, 1H, OBn), 4.61 (d, *J* = 12.3 Hz, 1H, OBn), 4.61 (d, *J* = 12.3 Hz, 1H, OBn), 4.54 (d, *J* = 11.0 Hz, 1H, OBn), 4.51 (d, *J* = 12.3 Hz, 1H, OBn), 3.987 [dd, *J* = 8.9, 5.1 Hz, 0.7H, 3-H (from 2β-^2^H)], 3.987 [dd, *J* = 11.5, 8.9 Hz, 0.3H, 3-H (from 2α-^2^H)], 3.89 (ddd, *J* = 8.2, 8.2, 5.3 Hz, 1H, 3’-H_b_), 3.83 (ddd, *J* = 8.2, 8.2, 6.6 Hz, 1H, 3’-H_a_), 3.79 (ddd, *J* = 9.9, 4.2, 1.7 Hz, 1H, 5-H), 3.74 (dd, *J* = 10.7, 4.2 Hz, 1H, 6-H_b_), 3.65 (dd, *J* = 10.7, 1.7 Hz, 1H, 6-H_a_), 3.57 (dd, *J* = 9.9, 8.9 Hz, 1H, 4-H), 2.200 (d, *J* = 5.1 Hz, 0.7H, 2α-H), 2.13–1.99 (m, 2H, 1’-H_b_, 2’-H_b_), 1.86 (m, 1H, 2’-H_a_), 1.85 (d, *J* = 11.5 Hz, 0.3H, 2β-H), 1.74 ppm (m, 1H, 1’-H_a_). ^13^C{^1^H} NMR (100.6 MHz, CDCl_3_) δ_C_ 138.9 (2 × C, Ar), 138.5 (C, Ar), 128.33 (2 × CH, Ar), 128.28 (2 × CH, Ar), 128.2 (2 × CH, Ar), 127.76 (2 × CH, Ar), 127.74 (2 × CH, Ar), 127.6 (2 × CH, Ar), 127.5 (2 × CH, Ar), 127.4, (CH, Ar), 106.30 (C, C-1), 79.1 (CH, C-4), 78.50 (CH, C-3), 74.7 (CH_2_, OBn), 73.3 (CH_2_, OBn), 71.8 (CH_2_, OBn), 71.8 (CH, C-5), 69.4 (CH_2_, C-6), 67.2 (CH_2_, C-3’), 38.26 (CHD, t, *J*_CD_ = 19.7 Hz, C-2), 37.1 (CH_2_, C-1’), 23.5 ppm (CH_2_, C-2’). MS (ESI) *m/z* (%) = 498 (100) [M + Na]^+^. HRMS (ESI) *m/z:* [M + Na]^+^ calcd for C_30_H_33_^2^HNaO_5_ 498.2367; found 498.2366. Compound (1-^2^H)**26β**: ^1^H NMR (400 MHz, CDCl_3_) δ_H_ 7.34–7.14 (m, 15H, Ar), 4.881 (d, *J* = 8.8 Hz, 1H, 2-H), 4.81 (d, *J* = 11.4 Hz, 1H, OBn), 4.77 (d, *J* = 10.8 Hz, 1H, OBn), 4.65 (d, *J* = 11.4 Hz, 1H, OBn), 4.59 (d, *J* = 12.2 Hz, 1H, OBn), 4.55 (d, *J* = 10.2 Hz, 1H, OBn), 4.52 (d, *J* = 10.6 Hz, 1H, OBn), 3.69 (dd, *J* = 10.7, 1.9 Hz, 1H, 6-H_b_), 3.66–3.57 (m, 5H, 3’-H_2_, 3-H, 4-H, 6-H_a_), 3.46 (m, 1H, 5-H), 1.94 (s, 3H, OAc), 1.75–1.61 (m, 3H, 1’-H_b_, 2’-H_2_), 1.497 ppm (ddd, *J* = 13.4, 13.4, 6.4 Hz, 1H, 1’-H_a_), 1H from OH is missing. ^13^C{^1^H} NMR (100.6 MHz, CDCl_3_) δ_C_ 169.9 (C, OAc), 138.4 (C, Ar), 138.1 (C, Ar), 138.0 (C, Ar), 128.42 (4 × CH, Ar), 128.38 (2 × CH, Ar), 128.0 (2 × CH, Ar), 127.8 (3 × CH, Ar), 127.70 (2 × CH, Ar), 127.67 (CH, Ar), 127.65 (CH, Ar), 84.6 (CH, C-3), 78.9 (CH, C-4), 78.5 (CH, C-5), 75.2 (CH_2_, OBn), 75.0 (CH_2_, OBn), 73.74 (CH, C-2), 73.5 (CH_2_, OBn), 69.1 (CH_2_, C-6), 62.7 (CH_2_, C-3’), 28.8 (CH_2_, C-2’), 28.13 (CH_2_, C-1’), 20.9 ppm (CH_3_, OAc). MS (ESI) *m/z* (%) = 558 (100) [M + Na]^+^. HRMS (ESI) *m/z:* [M + Na]^+^ calcd for C_32_H_37_^2^HNaO_7_ 558.2578; found 558.2574.

#### Radical Reactions of **2**

##### Method A

Following the general procedure, starting from substrate **2** (89.7 mg, 0.10 mmol), after 2 h of reaction, a supplementary addition of *n-*Bu_3_SnH (29 μL, 0.11 mmol) was required. All the starting material was consumed after 4 h. Column chromatography (hexanes to hexanes–EtOAc, 1:1) gave **25** (20.8 mg, 0.044 mmol, 44%) and an inseparable mixture of alcohols (12.8 mg, 0.018 mmol, 16%) that was elucidated by the usual acetylation to obtain 3-*C*-(3,4,6-tri-*O*-benzyl-2-*O*-diphenoxyphosphoryl-β-d-glucopyranosyl)1-propyl acetate (**27β**) (3.9 mg, 0.005 mmol, 5% from **2**) and 3-*C*-(3,4,6-tri-*O*-benzyl-2-*O*-diphenoxyphosphoryl-α-d-glucopyranosyl)1-propyl acetate (**27α**) (6.1 mg, 0.008 mmol, 7% from **2**), both as colorless oils. Compound **27β**: [α]_D_ = +16.2 (*c =* 0.33, CHCl_3_). ^1^H NMR (500 MHz, CDCl_3_) δ_H_ 7.36–7.11 (m, 25H, Ar), 4.59 (d, *J* = 12.3 Hz, 1H, OBn), 4.52 (m, 1H, 2-H), 4.52 (d, *J* = 12.0 Hz, 1H, OBn), 4.51 (d, *J* = 12.0 Hz, 1H, OBn), 4.46 (d, *J* = 12.3 Hz, 1H, OBn), 4.43 (d, *J* = 12.0 Hz, 1H, OBn), 4.13 (d, *J* = 12.3 Hz, 1H, OBn), 4.09 (dd, *J* = 2.5, 2.5 Hz, 1H, 3-H), 4.01–3.92 (m, 3H, 5-H, 3’-H_2_), 3.78 (m, 1H, 1-H), 3.68 (dd, *J* = 10.1, 6.6 Hz, 1H, 6-H_b_), 3.54 (dd, *J* = 10.1, 5.7 Hz, 1H, 6-H_a_), 3.35 (br s, 1H, 4-H), 1.98 (s, 3H, OAc), 1.83–1.75 (m, 2H, 1’-H_b_, 2’-H_b_), 1.65–1.39 ppm (m, 2H, 1’-H_a_, 2’-H_a_). ^13^C{^1^H} NMR (125.7 MHz, CDCl_3_) δ_C_ 171.1 (C, OAc), 150.5 (2 × C, Ar), 138.3 (C, Ar), 137.7 (C, Ar), 137.4 (C, Ar), 120.0–129.7 (25 × CH, Ar), 77.2 (CH, C-2), 75.4 (CH, C-5), 74.6 (d, ^3^*J*_PC_ = 6.4 Hz, CH, C-1), 73.4 (CH_2_, OBn), 72.3 (CH_2_, OBn), 71.7 (CH, C-3 or C-4), 71.6 (CH_2_, OBn), 71.5 (CH, C-3 or C-4), 69.8 (CH_2_, C-6), 64.3 (CH_2_, C-3’), 27.5 (CH_2_, C-1’ or C-2’), 24.9 (CH_2_, C-1’ or C-2’), 20.9 ppm (CH_3_, OAc). IR (CHCl_3_): ν = 2927, 1733, 1491, 1027 cm^–1^. MS (ESI) *m/z* (%) = 789 (100) [M + Na]^+^. HRMS (ESI) *m/z:* [M + Na]^+^ calcd for C_44_H_47_NaO_10_P 789.2805; found 789.2831. Compound **27α**: [α]_D_ = +22.0 (*c =* 0.30, CHCl_3_). ^1^H NMR (500 MHz, CDCl_3_) δ_H_ 7.32–7.08 (m, 25H, Ar), 4.82 (d, *J* = 11.1 Hz, 1H, OBn), 4.79 (ddd, *J* = 8.3, 5.7 Hz, ^3^*J*_PH_ = 8.3 Hz, 1H, 2-H), 4.74 (d, *J* = 10.7 Hz, 1H, OBn), 4.73 (d, *J* = 11.1 Hz, 1H, OBn), 4.60 (d, *J* = 12.0 Hz, 1H, OBn), 4.46 (d, *J* = 12.3 Hz, 1H, OBn), 4.45 (d, *J* = 10.5 Hz, 1H, OBn), 4.15 (m, 1H, 1-H), 4.02 (ddd, *J* = 10.7, 10.7, 6.3 Hz, 1H, 3’-H_b_), 3.98 (ddd, *J* = 10.8, 10.8, 6.3 Hz, 1H, 3’-H_a_), 3.86 (dd, *J* = 8.6, 8.6 Hz, 1H, 3-H), 3.70–3.60 (m, 4H, 4-H, 5-H, 6-H_2_), 2.00 (s, 3H, OAc), 1.77–1.69 (m, 2H, 1’-H_b_, 2’-H_b_), 1.62–1.49 ppm (m, 2H, 1’-H_a_, 2’-H_a_). ^13^C{^1^H} NMR (125.7 MHz, CDCl_3_) δ_C_ 171.1 (C, OAc), 150.5 (2 × C, Ar), 138.0 (C, Ar), 137.9 (C, Ar), 137.8 (C, Ar), 129.8 (2 × CH, Ar), 129.7 (2 × CH, Ar), 128.41 (2 × CH, Ar), 128.37 (2 × CH, Ar), 128.3 (2 × CH, Ar), 127.9 (2 × CH, Ar), 127.84 (3 × CH, Ar), 127.78 (2 × CH, Ar), 127.7 (CH, Ar), 127.6 (CH, Ar), 125.5 (CH, Ar), 125.3 (CH, Ar), 120.14 (CH, Ar), 120.10 (CH, Ar), 120.0 (CH, Ar), 119.9 (CH, Ar), 119.9–129.8 (25 × CH, Ar), 80.5 (d, ^3^*J*_PC_ = 6.4 Hz, CH, C-3), 78.4 (d, ^3^*J*_PC_ = 7.4 Hz, CH, C-1), 77.7 (CH, C-2), 75.1 (CH_2_, OBn), 74.9 (CH_2_, OBn), 73.5 (CH_2_, OBn), 73.4 (CH, C-5), 71.5 (CH, C-4), 68.6 (CH_2_, C-6), 64.1 (CH_2_, C-3’), 24.5 (CH_2_, C-1’ or C-2’), 24.5 (CH_2_, C-1’ or C-2’), 21.0 ppm (CH_3_, OAc). IR (CHCl_3_): ν = 3013, 2928, 1730, 1026 cm^–1^. MS (ESI) *m/z* (%) = 789 (100) [M + Na]^+^. HRMS (ESI) *m/z:* [M + Na]^+^ calcd for C_44_H_47_NaO_10_P 789.2805; found 789.2823.

##### Method B

Following the general procedure, starting from substrate **2** (56 mg, 0.064 mmol), after 2 h of reaction, two more equivalents of *n-*Bu_3_SnH (35 μL, 0.128 mmol) added by a syringe pump were required. All the starting material was consumed after 11 h. Column chromatography (hexanes to hexanes–EtOAc, 1:1) gave **25** (16.2 mg, 0.034 mmol, 53%).

##### Method D

Following the general procedure, starting from substrate **2** (66.3 mg, 0.076 mmol), after 2 h of reaction, a supplementary addition of *n-*Bu_3_SnD (21 μL, 0.076 mmol) was required. All the starting material was consumed after 4 h. Column chromatography (hexanes to hexanes–EtOAc, 1:1) gave (2-^2^H)**25** (22.5 mg, 0.047 mmol, 62%) and the inseparable mixture of 3-*C*-(3,4,6-tri-*O*-benzyl-2-*O*-diphenoxyphosphoryl-β-d-(1-^2^H)glucopyranosyl)1-propanol [(1-^2^H)**28β**] and 3-*C*-(3,4,6-tri-*O*-benzyl-2-*O*-diphenoxyphosphoryl-α-d-glucopyranosyl)1-propanol (**28α**) (11.1 mg, 0.015 mmol, 20%, (1-^2^H)**28β**/**28α**, 1:2.1) as a colorless oil. Compounds (1-^2^H)**28β** and **28α**: ^1^H NMR (500 MHz, CDCl_3_, selected resolved signals of (1-^2^H)**28β** from the mix spectrum) δ_H_ 4.74 (d, *J* = 11.0 Hz, 1H, OBn), 4.74 (d, *J* = 11.0 Hz, 1H, OBn), 4.59 (d, *J* = 12.0 Hz, 1H, OBn), 4.532 (d, *J* = 2.6 Hz, 1H, 2-H), 4.53 (d, *J* = 12.0 Hz, 1H, OBn), 4.46 (d, *J* = 11.0 Hz, 1H, OBn), 4.42 (d, *J* = 11.7 Hz, 1H, OBn), 4.10 (d, *J* = 12.2 Hz, 1H, OBn), 4.07 (dd, *J* = 2.6, 2.6 Hz, 1H, 3-H), 3.44 (dd, *J* = 10.1, 0.0 Hz, 1H, 6-H_a_), 3.31 ppm (dd, *J* = 2.6, 1.0 Hz, 1H, 4-H). ^13^C{^1^H} NMR (125.7 MHz, CDCl_3_, selected resolved signals of (1-^2^H)**28β** from the mix spectrum) δ_C_ 150.4 (d, ^2^*J*_PC_ = 7.0 Hz, 2 × C, Ar), 138.1 (C, Ar), 137.6 (C, Ar), 137.3 (C, Ar), 80.5 (d, ^3^*J*_PC_ = 6.4 Hz, CH, C-3), 78.55 (d, ^2^*J*_PC_ = 7.4 Hz, CH, C-2), 75.0 (CH, C-5), 73.5 (CH_2_, OBn), 72.2 (CH_2_, OBn), 71.5 (CH_2_, OBn), 71.5 (CH, C-4), 69.8 (CH_2_, C-6), 62.5 (CH_2_, C-3’), 29.68 (CH_2_, C-1’ or C-2’), 28.08 ppm (CH_2_, C-1’ or C-2’). ^1^H NMR (500 MHz, CDCl_3_, selected resolved signals of **28α** from the mix spectrum) δ_H_ 4.80 (d, *J* = 11.0 Hz, 1H, OBn), 4.770 (ddd, *J* = 7.6, 4.5 Hz, ^3^*J*_PH_ = 6.3 Hz, 1H, 2-H), 4.71 (d, *J* = 10.8 Hz, 1H, OBn), 4.58 (d, *J* = 12.0 Hz, 1H, OBn), 4.48 (d, *J* = 12.3 Hz, 1H, OBn), 4.44 (d, *J* = 11.1 Hz, 1H, OBn), 4.23 (ddd, *J* = 11.4, 5.7, 2.2 Hz, 1-H), 3.85 ppm (dd, *J* = 8.5, 8.5 Hz, 1H, 3-H). ^13^C{^1^H} NMR (125.7 MHz, CDCl_3_, selected resolved signals of **28α** from the mix spectrum) δ_C_ 150.5 (d, ^2^*J*_PC_ = 7.0 Hz, 2 × C, Ar), 138.0 (C, Ar), 137.8 (2 × C, Ar), 80.5 (d, ^3^*J*_PC_ = 6.4 Hz, CH, C-3), 78.55 (d, ^2^*J*_PC_ = 7.4 Hz, CH, C-2), 77.8 (CH, C-5), 75.1 (CH_2_, OBn), 74.8 (CH_2_, OBn), 73.6 (CH, C-1), 73.5 (CH_2_, OBn), 71.4 (CH, C-4), 68.8 (CH_2_, C-6), 61.8 (CH_2_, C-3’), 28.56 (CH_2_, C-1’ or C-2’), 20.69 ppm (CH_2_, C-1’ or C-2’). IR (CHCl_3_): ν = 3490, 3422, 2928, 1550, 1491, 1192 cm^–1^. MS (ESI) *m*/*z* (%) = 747 (99.6) [M + Na]^+^, 748 (100) [M + Na]^+^. HRMS (ESI) *m*/*z:* [M + Na]^+^ calcd for C_42_H_45_NaO_9_P 747.2699; found 747.2697, [M + Na]^+^ calcd for C_42_H_44_^2^HNaO_9_P 748.2762; found 748.2764.

#### Radical Reactions of **3**

##### Method A

Following the general procedure, starting from substrate **3** (89.2 mg, 0.13 mmol), after 2 h of reaction, a supplementary addition of *n-*Bu_3_SnH (36 μL, 0.13 mmol) was required. All the starting material was consumed after 4 h. Column chromatography (hexanes to hexanes–EtOAc, 6:4) gave **25** (13.9 mg, 0.029 mmol, 23%) and **26β** (20.4 mg, 0.038 mmol, 29%).

##### Method B

Following the general procedure, starting from substrate **3** (110.2 mg, 0.16 mmol), after 2 h of reaction, two more equivalents of *n-*Bu_3_SnH (88 μL, 0.32 mmol) added by a syringe pump over 2 h were required. All the starting material was consumed after 5 h. Column chromatography (hexanes to hexanes–EtOAc, 6:4) gave **25** (22.4 mg, 0.047 mmol, 30%) and the reduced product **26β** (16.4 mg, 0.031 mmol, 19%).

##### Method C

Following the general procedure, starting from substrate **3** (96.2 mg, 0.14 mmol), after 2 h of reaction, a supplementary addition of TTMSS (66 μL, 0.21 mmol) was required. All the starting material was consumed after 5 h. Column chromatography (hexanes–EtOAc, 9:1 to 6:4) gave **25** (10.2 mg, 0.022 mmol, 23%) and the reduced product **26β** (3 mg, 0.006 mmol, 4%).

##### Method D

Following the general procedure, starting from substrate **3** (106.6 mg, 0.16 mmol), after 2 h of reaction, a supplementary addition of *n-*Bu_3_SnD (42 μL, 0.16 mmol) was required. All the starting material was consumed after 5 h. Column chromatography (hexanes to hexanes–EtOAc, 6:4) gave (2-^2^H)**25** (25.2 mg, 0.053 mmol, 33%) and [1-^2^H]**26β** (8.3 mg, 0.016 mmol, 10%, ^2^H/^1^H 1:1).

#### Radical Reactions of **4**

##### Method A

Following the general procedure, starting from substrate **4** (57.3 mg, 0.066 mmol), after 2 h of reaction and again after 4 h, a supplementary addition of *n-*Bu_3_SnH (18 μL, 0.066 mmol) was required. All the starting material was consumed after 6 h. Column chromatography (hexanes to hexanes–EtOAc, 8:2) gave **25** (14.2 mg, 0.030 mmol, 45%).

##### Method B

Following the general procedure, starting from substrate **4** (53 mg, 0.061 mmol), after 2 h of reaction, a supplementary addition of *n-*Bu_3_SnH (16 μL, 0.061 mmol) added by a syringe pump over 1 h was required. All the starting material was consumed after 6 h. Column chromatography (hexanes to hexanes–EtOAc, 8:2) gave **25** (12 mg, 0.025 mmol, 42%).

##### Method D

Following the general procedure, starting from substrate **4** (75 mg, 0.086 mmol), after 2 h of reaction, a supplementary addition of *n-*Bu_3_SnD (23 μL, 0.086 mmol) was required. All the starting material was consumed after 5 h. Column chromatography (hexanes to hexanes–EtOAc, 8:2) gave (2-^2^H)**25** (22.5 mg, 0.047 mmol, 55%).

#### Radical Reactions of **5**

##### Method A

Following the general procedure, starting from substrate **5** (40.2 mg, 0.059 mmol), after 2 h of reaction, a supplementary addition of *n-*Bu_3_SnH (16 μL, 0.059 mmol) was required. All the starting material was consumed after 7 h. Column chromatography (hexanes to hexanes–EtOAc, 6:4) gave an inseparable mixture of isomers 3-*C*-(2-*O*-acetyl-3,4,6-tri-*O*-benzyl-β-d-mannopyranosyl)1-propanol (**29β**) and 3-*C*-(2-*O*-acetyl-3,4,6-tri-*O*-benzyl-α-d-mannopyranosyl)1-propanol (**29α**) (16.1 mg, 0.030 mmol, 51%, 2.3:1) as a colorless oil. Compounds **29β** and **29α**: ^1^H NMR (500 MHz, selected resolved signals of **29β** from the mix spectrum) δ_H_ 5.473 (dd, *J* = 2.2, 0.0 Hz, 1H, 2-H), 4.85 (d, *J* = 10.8 Hz, 1H, OBn), 4.74 (d, *J* = 11.4 Hz, 1H, OBn), 4.61 (d, *J* = 12.3 Hz, 1H, OBn), 4.54 (d, *J* = 12.0 Hz, 1H, OBn), 4.50 (d, *J* = 11.1 Hz, 1H, OBn), 4.49 (d, *J* = 11.4 Hz, 1H, OBn), 4.48 (d, *J* = 10.4 Hz, 1H, OBn), 2.17 ppm (s, 3H, OAc). ^13^C{^1^H} NMR (100.6 MHz, CDCl_3_, selected resolved signals of **29β** from the mix spectrum) δ_C_ 170.9 (C, OAc), 138.3 (C, Ar), 138.2 (C, Ar), 137.8 (C, Ar), 81.9 (CH), 79.3 (CH), 77.2 (CH, C-1), 75.1 (CH_2_, OBn), 74.7 (CH), 73.5 (CH_2_, OBn), 71.6 (CH_2_, OBn), 69.6 (CH_2_, C-6), 69.56 (CH, C-2), 62.6 (CH_2_, C-3’), 29.5 (CH_2_, C-2’), 29.3 (CH_2_, C-2’), 28.17 (CH_2_, C-1’), 21.0 ppm (CH_3_, OAc). ^1^H NMR (500 MHz, CDCl_3_, selected resolved signals of **29α** from the mix spectrum) δ_H_ 5.245 (dd, *J* = 2.8, 2.8 Hz, 1H, 2-H), 4.81 (d, *J* = 11.1 Hz, 1H, OBn), 4.65 (d, *J* = 11.4 Hz, 1H, OBn), 4.61 (d, *J* = 12.3 Hz, 1H, OBn), 4.51 (d, *J* = 11.7 Hz, 1H, OBn), 4.47 (d, *J* = 11.1 Hz, 1H, OBn), 3.99 (ddd, *J* = 10.7, 6.6, 3.8 Hz, 1H, 1-H), 3.85 (dd, *J* = 8.2, 3.2 Hz, 1H, 3-H), 2.13 ppm (s, 3H, OAc). ^13^C{^1^H} NMR (100.6 MHz, CDCl_3_, selected resolved signals of **29α** from the mix spectrum) δ_C_ 170.6 (C, OAc), 138.3 (C, Ar), 138.2 (C, Ar) 137.8 (C, Ar), 77.7 (CH), 75.3 (CH_2_, OBn), 74.8 (CH), 74.7 (CH), 73.5 (CH_2_, OBn), 72.8 (CH), 71.9 (CH_2_, OBn), 70.9 (CH), 69.4 (CH_2_, C-6), 62.0 (CH_2_, C-3’), 24.95 (CH_2_, C-1’), 21.2 ppm (CH_3_, OAc). IR (CHCl_3_): ν = 3496, 3014, 2928, 1735, 1238, 1095 cm^–1^. MS (ESI) *m*/*z* (%) = 557 (100) [M + Na]^+^. HRMS (ESI) *m*/*z:* [M + Na]^+^ calcd for C_32_H_38_NaO_7_ 557.2515; found 557.2515. Anal. calcd for C_32_H_38_O_7_: C, 71.89; H, 7.16. Found: C, 71.55; H, 7.11.

##### Method D

Following the general procedure, starting from substrate **5** (30.2 mg, 0.044 mmol), after 2 h of reaction, a supplementary addition of *n-*Bu_3_SnD (12 μL, 0.044 mmol) was required. All the starting material was consumed after 5 h. Column chromatography (hexanes to hexanes–EtOAc, 7:3) gave the mixture of 3-*C*-(2-*O*-acetyl-3,4,6-tri-*O*-benzyl-β-d-(1-^2^H)mannopyranosyl)1-propanol [(1-^2^H)**29β**] and **29α** (14.4 mg, 0.027 mmol, 61%, ^2^H/^1^H 1.8:1) as a colorless oil. Compounds (1-^2^H)**29β** and **29α**: ^1^H NMR (500 MHz, CDCl_3_, selected resolved signals of (1-^2^H)**29β** from the mix spectrum) δ_H_ 4.74 (d, *J* = 11.0 Hz, 1H, OBn), 4.74 (d, *J* = 11.0 Hz, 1H, OBn), 4.59 (d, *J* = 12.0 Hz, 1H, OBn), 4.532 (d, *J* = 2.6 Hz, 1H, 2-H), 4.53 (d, *J* = 12.0 Hz, 1H, OBn), 4.46 (d, *J* = 11.0 Hz, 1H, OBn), 4.42 (d, *J* = 11.7 Hz, 1H, OBn), 4.10 (d, *J* = 12.2 Hz, 1H, OBn), 4.07 (dd, *J* = 2.6, 2.6 Hz, 1H, 3-H), 3.44 (dd, *J* = 10.1, 0.0 Hz, 1H, 6-H_a_), 3.31 ppm (dd, *J* = 2.6, 1.0 Hz, 1H, 4-H). ^13^C{^1^H} NMR (125.7 MHz, CDCl_3_, selected resolved signals of (1-^2^H)**29β** from the mix spectrum) δ_C_ 150.4 (d, ^2^*J*_PC_ = 7.0 Hz, 2 × C, Ar), 138.1 (C, Ar), 137.6 (C, Ar), 137.3 (C, Ar), 80.5 (d, ^3^*J*_PC_ = 6.4 Hz, CH, C-3), 78.55 (d, ^2^*J*_PC_ = 7.4 Hz, CH, C-2), 75.0 (CH, C-5), 73.5 (CH_2_, OBn), 72.2 (CH_2_, OBn), 71.5 (CH_2_, OBn), 71.5 (CH, C-4), 69.8 (CH_2_, C-6), 62.5 (CH_2_, C-3’), 29.68 (CH_2_, C-1’ or C-2’), 28.08 ppm (CH_2_, C-1’ or C-2’). IR (CHCl_3_): ν = 3490, 3422, 2928, 1550, 1491, 1192 cm^–1^. MS (ESI) *m*/*z* (%) = 747 (99.6) [M + Na]^+^, 748 (100) [M + Na]^+^. HRMS (ESI) *m*/*z:* [M + Na]^+^ calcd for C_42_H_45_NaO_9_P 747.2699; found 747.2697, [M + Na]^+^ calcd for C_42_H_44_^2^HNaO_9_P 748.2762; found 748.2764.

#### Radical Reactions of **6**

##### Method A

Following the general procedure, starting from substrate **6** (39 mg, 0.045 mmol), after 2 h of reaction, a supplementary addition of *n-*Bu_3_SnH (12 μL, 0.045 mmol) was required. All the starting material was consumed after 6 h. Column chromatography (hexanes to hexanes–EtOAc, 1:1) gave **25** (9.7 mg, 0.020 mmol, 46%) and 3-*C*-(3,4,6-tri-*O*-benzyl-2-*O*-diphenoxyphosphoryl-β-d-mannopyranosyl)1-propanol (**30α**) (4 mg, 0.006 mmol, 12%) as a colorless oil. Compound **30α**: [α]_D_ = +1.6 (*c =* 0.77, CHCl_3_). ^1^H NMR (500 MHz, CDCl_3_) δ_H_ 7.36–7.11 (m, 25H, Ar), 4.90 (ddd, *J* = 3.2, 3.2 Hz, ^3^*J*_PH_ = 6.3 Hz, 1H, 2-H), 4.74 (d, *J* = 11.4 Hz, 1H, OBn), 4.65 (d, *J* = 11.4 Hz, 1H, OBn), 4.56 (d, *J* = 12.0 Hz, 1H, OBn), 4.52 (d, *J* = 12.0 Hz, 1H, OBn), 4.47 (d, *J* = 11.4 Hz, 1H, OBn), 4.38 (d, *J* = 11.1 Hz, 1H, OBn), 4.04 (ddd, *J* = 10.1, 3.5, 3.5 Hz, 1H, 1-H), 3.85 (ddd, *J* = 8.2, 2.5 Hz, ^4^*J*_PH_ = 2.5 Hz, 1H, 3-H), 3.76 (ddd, *J* = 8.5, 5.4, 3.5 Hz, 1H, 5-H), 3.69–3.56 (m, 5H, 3’-H_2_, 4-H, 6-H_2_), 1.78–1.47 ppm (m, 4H, 1’-H_2_, 2’-H_2_), 1H from OH is missing. Stereochemistry was assigned as 1α since the starting phthalimide **6** was obtained by the reaction with *N*-hydroxyphthalimide under Mitsunobu conditions. ^13^C{^1^H} NMR (125.7 MHz, CDCl_3_) δ_C_ 150.7 (C, Ar), 150.5 (C, Ar), 138.1 (C, Ar), 138.0 (C, Ar), 137.6 (C, Ar), 129.8 (2 × CH, Ar), 129.6 (2 × CH, Ar), 128.38 (2 × CH, Ar), 128.36 (2 × CH, Ar), 128.3 (2 × CH, Ar), 128.2 (2 × CH, Ar), 128.0 (2 × CH, Ar), 127.8 (4 × CH, Ar), 127.6 (CH, Ar), 125.4 (CH, Ar), 125.1 (CH, Ar), 120.4 (CH, Ar), 120.3 (CH, Ar), 120.23 (CH, Ar), 120.19 (CH, Ar), 77.7 (d, ^2^*J*_PC_ = 6.4 Hz, CH, C-2), 77.6 (2 × CH, C-3, C-4), 74.5 (CH_2_, OBn), 74.4 (CH, C-1), 73.4 (CH_2_, OBn), 73.1 (CH, C-5), 72.1 (CH_2_, OBn), 69.1 (CH_2_, C-6), 61.8 (CH_2_, C-3’), 29.4 (CH_2_, C-1’ or C-2’), 29.1 ppm (CH_2_, C-1’ or C-2’). IR (CHCl_3_): ν = 3503, 2928, 1712, 1491, 1192 cm^–1^. MS (ESI) *m*/*z* (%) = 747 (100) [M + Na]^+^. HRMS (ESI) *m*/*z:* [M + Na]^+^ calcd for C_42_H_45_NaO_9_P 747.2699; found 747.2692.

##### Method D

Following the general procedure, starting from substrate **6** (30.2 mg, 0.035 mmol), after 2 h of reaction, a supplementary addition of *n-*Bu_3_SnD (9 μL, 0.035 mmol) was required. All the starting material was consumed after 4 h. Column chromatography (hexanes to hexanes–EtOAc, 1:1) gave (2-^2^H)**25** (8.6 mg, 0.018 mmol, 52%) and **30α** (4.7 mg, 0.005 mmol, 19%).

##### Method F

Following the general procedure, starting from substrate **6** (57.3 mg, 0.066 mmol), all the starting material was consumed after 1 h. Column chromatography (hexanes–EtOAc, 85:15 to 4:6) gave **25** (9 mg, 0.019 mmol, 29%) and **30α** (24.2 mg, 0.033 mmol, 51%).

##### Method G

Following the general procedure, starting from substrate **6** (59 mg, 0.068 mmol), all the starting material was consumed after 3 h. Column chromatography (hexanes–EtOAc, 8:2 to 4:6) gave **25** (11.3 mg, 0.024 mmol, 35%) and **30α** (22.1 mg, 0.031 mmol, 45%).

#### Radical Reactions of **7**

##### Method A

Following the general procedure, starting from substrate **7** (74.6 mg, 0.11 mmol), after 2 h of reaction, a supplementary addition of *n-*Bu_3_SnH (30 μL, 0.11 mmol) was required. All the starting material was consumed after 4 h. Column chromatography (hexanes to hexanes–EtOAc, 6:4) gave 3-*C*-(2-*O*-acetyl-3,4,6-tri-*O*-benzyl-β-d-mannopyranosyl)1-propanol (**29β**) (56.8 mg, 0.11 mmol, 97%) as an amorphous solid: [α]_D_ = −27.6 (*c =* 0.87, CHCl_3_). ^1^H NMR (500 MHz, CDCl_3_) δ_H_ 7.35–7.15 (m, 15H, Ar), 5.474 (dd, *J* = 2.2, 0.0 Hz, 1H, 2-H), 4.85 (d, *J* = 10.7 Hz, 1H, OBn), 4.74 (d, *J* = 11.1 Hz, 1H, OBn), 4.61 (d, *J* = 12.0 Hz, 1H, OBn), 4.54 (d, *J* = 12.3 Hz, 1H, OBn), 4.48 (d, *J* = 11.0 Hz, 1H, OBn), 4.48 (d, *J* = 11.0 Hz, 1H, OBn), 3.74 (dd, *J* = 10.7, 1.9 Hz, 1H, 6-H_b_), 3.70–3.63 (m, 5H, 3-H, 4-H, 5-H, 6-H_a_, 3’-H_b_), 3.51–3.46 (m, 2H, 1-H, 3’-H_a_), 2.17 (s, 3H, OAc), 1.73–1.66 (m, 3H, 1’-H_b_, 2’-H_2_), 1.572 ppm (m, 1H, 1’-H_a_), 1H from OH is missing. ^13^C{^1^H} NMR (125.7 MHz, CDCl_3_) δ_C_ 170.9 (C, OAc), 138.3 (C, Ar), 138.2 (C, Ar), 137.8 (C, Ar), 127.5–128.3 (15 × CH, Ar), 81.8 (CH), 79.2 (CH), 77.1 (CH, C-1), 75.1 (CH_2_, OBn), 74.7 (CH), 73.4 (CH_2_, OBn), 71.5 (CH_2_, OBn), 69.49 (CH, C-2), 69.5 (CH_2_, C-6), 62.4 (CH_2_, C-3’), 29.3 (CH_2_, C-2’), 28.08 (CH_2_, C-1’), 20.9 ppm (CH_3_, OAc). IR (CHCl_3_): ν = 3430, 3015, 2936, 1735, 1091 cm^–1^. MS (ESI) *m*/*z* (%) = 557 (100) [M + Na]^+^. HRMS (ESI) *m*/*z:* [M + Na]^+^ calcd for C_32_H_38_NaO_7_ 557.2515; found 557.2519. Anal. calcd for C_32_H_38_O_7_: C, 71.89; H, 7.16. Found: C, 71.93; H, 7.08.

##### Method B

Following the general procedure, starting from substrate **7** (63.8 mg, 0.094 mmol), after 2 h of reaction, a supplementary addition of *n-*Bu_3_SnH (13 μL, 0.046 mmol) added by a syringe pump over 1 h was required. All the starting material was consumed after 5 h. Column chromatography (hexanes to hexanes–EtOAc, 6:4) gave **29β** (38.3 mg, 0.072 mmol, 76%).

##### Method C

Following the general procedure, starting from substrate **7** (63.9 mg, 0.094 mmol), after 3 h of reaction, a supplementary addition of TTMSS (29 μL, 0.094 mmol) was required. All the starting material was consumed after 5 h. Column chromatography (hexanes–EtOAc, 7:3 to 1:1) gave **29β** (28.8 mg, 0.054 mmol, 57%).

##### Method D

Following the general procedure, starting from substrate **7** (52.4 mg, 0.077 mmol), after 3 h of reaction, a supplementary addition of *n-*Bu_3_SnD (21 μL, 0.077 mmol) was required. All the starting material was consumed after 6 h. Column chromatography (hexanes to hexanes–EtOAc, 1:1) gave 3-*C*-(2-*O*-acetyl-3,4,6-tri-*O*-benzyl-β-d-[1-^2^H]mannopyranosyl)1-propanol ([1-^2^H]**29β**) (30.8 mg, 0.057 mmol, 75%, ^2^H/^1^H 5.8:1) as an amorphous solid: ^1^H NMR (500 MHz, CDCl_3_) δ_H_ 7.36–7.15 (m, 15H, Ar), 5.466 (d, *J* = 2.9 Hz, 1H, 2-H), 4.85 (d, *J* = 10.7 Hz, 1H, OBn), 4.74 (d, *J* = 11.1 Hz, 1H, OBn), 4.61 (d, *J* = 12.0 Hz, 1H, OBn), 4.54 (d, *J* = 12.3 Hz, 1H, OBn), 4.48 (d, *J* = 10.4 Hz, 1H, OBn), 4.48 (d, *J* = 10.4 Hz, 1H, OBn), 3.73 (dd, *J* = 10.8, 1.9 Hz, 1H, 6-H_b_), 3.70–3.63 (m, 5H, 3-H, 4-H, 5-H, 6-H_a_, 3’-H_b_), 3.47 (ddd, *J* = 8.6, 6.0, 1.9 Hz, 1H, 3’-H_a_), 2.18 (s, 3H, OAc), 1.71–1.66 (m, 3H, 1’-H_b_, 2’-H_2_), 1.566 ppm (m, 1H, 1’-H_a_), 1H from OH is missing. ^13^C{^1^H} NMR (125.7 MHz, CDCl_3_) δ_C_ 170.9 (C, OAc), 138.3 (C, Ar), 138.2 (C, Ar), 137.8 (C, Ar), 127.6–128.4 (15 × CH, Ar), 81.8 (CH), 79.2 (CH), 75.1 (CH_2_, OBn), 74.7 (CH), 73.4 (CH_2_, OBn), 71.5 (CH_2_, OBn), 69.50 (CH, C-2), 69.4 (CH_2_, C-6), 62.5 (CH_2_, C-3’), 29.4 (CH_2_, C-2’), 28.06 (CH_2_, C-1’), 21.0 ppm (CH_3_, OAc), C-1 was undetectable. MS (ESI) *m*/*z* (%) = 558 (100) [M + Na]^+^, 557 (16) [M + Na]^+^. HRMS (ESI) *m*/*z:* [M + Na]^+^ calcd for C_32_H_37_^2^HNaO_7_ 558.2578; found 558.2582, [M + Na]^+^ calcd for C_32_H_38_NaO_7_ 557.2515; found 557.2511.

##### Method E

Following the general procedure, starting from substrate **7** (57.8 mg, 0.085 mmol), after 2 h of reaction, a supplementary addition of *n-*Bu_3_SnD (23 μL, 0.085 mmol) and BF_3_•Et_2_O (2 μL, 0.017 mmol) was required. All the starting material was consumed after 5 h. Column chromatography (hexanes to hexanes–EtOAc, 1:1) gave [2-^2^H]**25** (20.2 mg, 0.043 mmol, 50%, ^2^H/^1^H 1.6:1) and [1-^2^H]**29β** (8.6 mg, 0.016 mmol, 19%, ^2^H/^1^H 3.5:1).

#### Radical Reactions of **8**

##### Method A

Following the general procedure, starting from substrate **8** (60.4 mg, 0.07 mmol), after 2 h of reaction, a supplementary addition of *n-*Bu_3_SnH (19 μL, 0.07 mmol) was required. All the starting material was consumed after 6 h. Column chromatography (hexanes to hexanes–EtOAc, 8:2) gave **25** (12 mg, 0.025 mmol, 37%).

##### Method B

Following the general procedure, starting from substrate **8** (64.6 mg, 0.075 mmol), after 2 h of reaction, a supplementary addition of *n-*Bu_3_SnH (20 μL, 0.075 mmol) added by a syringe pump over 1 h was required. All the starting material was consumed after 11 h. Column chromatography (hexanes to hexanes–EtOAc, 8:2) gave **25** (13.7 mg, 0.029 mmol, 39%).

##### Method D

Following the general procedure, starting from substrate **8** (92.7 mg, 0.11 mmol), after 2 h of reaction, a supplementary addition of *n-*Bu_3_SnD (29 μL, 0.11 mmol) was required. All the starting material was consumed after 3 h. Column chromatography (hexanes to hexanes–EtOAc, 8:2) gave (2-^2^H)**25** (26.3 mg, 0.055 mmol, 52%).

##### Method E

Following the general procedure, starting from substrate **8** (60 mg, 0.069 mmol), after 2 h of reaction, a supplementary addition of *n-*Bu_3_SnD (17 μL, 0.069 mmol) and BF_3_•Et_2_O (2 μL, 0.016 mmol) was required. All the starting material was consumed after 3 h. Column chromatography (hexanes to hexanes–EtOAc, 1:1) gave [2-^2^H]**25** (20.2 mg, 0.043 mmol, 65%, ^2^H/^1^H 2.4:1).

##### Method F

Following the general procedure, starting from substrate **8** (33.6 mg, 0.039 mmol), all the starting material was consumed after 2 h. Column chromatography (hexanes–EtOAc, 8:2 to 1:1) gave **25** (4.1 mg, 8.6·10^–3^ mmol, 22%) and 3-*C*-(3,4,6-tri-*O*-benzyl-2-*O*-diphenoxyphosphoryl-β-d-mannopyranosyl)1-propanol (**30β**) (10.3 mg, 0.014 mmol, 37%) as a colorless oil. Compound **30β**: [α]_D_ = −27.9 (*c =* 0.10, CHCl_3_). ^1^H NMR (400 MHz, CDCl_3_) δ_H_ 7.39–7.05 (m, 25H, Ar), 5.01 (dd, *J* = 9.0, 2.1, 1H, 2-H), 4.90 (d, *J* = 11.3 Hz, 1H, OBn), 4.63 (d, *J* = 11.1 Hz, 1H, OBn), 4.60 (d, *J* = 12.5 Hz, 1H, OBn), 4.53 (d, *J* = 12.2 Hz, 1H, OBn), 4.51 (d, *J* = 11.4 Hz, 1H, OBn), 4.29 (d, *J* = 10.8 Hz, 1H, OBn), 3.67 (dd, *J* = 10.8, 1.8 Hz, 1H), 3.63–3.56 (m, 3H), 3.54–3.49 (m, 2H), 3.44–3.40 (m, 2H), 1.68–1.54 ppm (m, 4H, 1’-H_2_, 2’-H_2_), 1H from OH is missing. ^13^C{^1^H} NMR (100.6 MHz, CDCl_3_) δ_C_ 150.9 (d, ^2^*J*_PC_ = 8.5 Hz, C, Ar), 150.7 (d, ^2^*J*_PC_ = 6.4 Hz, C, Ar), 138.2 (C, Ar), 138.1 (C, Ar), 137.6 (C, Ar), 129.7 (2 × CH, Ar), 129.4 (2 × CH, Ar), 128.4 (2 × CH, Ar), 128.33 (4 × CH, Ar), 128.26 (2 × CH, Ar), 128.0 (2 × CH, Ar), 127.8 (2 × CH, Ar), 127.70 (CH, Ar), 127.65 (CH, Ar), 127.6 (CH, Ar), 125.3 (CH, Ar), 124.8 (CH, Ar), 120.4 (CH, Ar), 120.3 (CH, Ar), 120.22 (CH, Ar), 120.16 (CH, Ar), 81.8 (CH, C-3), 79.2 (CH, C-5), 77.3 (CH, C-2), 77.2 (d, ^3^*J*_PC_ = 7.8 Hz, CH, C-1), 75.2 (CH_2_, OBn), 74.1 (CH, C-4), 73.4 (CH_2_, OBn), 71.7 (CH_2_, OBn), 69.3 (CH_2_, C-6), 62.4 (CH_2_, C-3’), 29.3 (CH_2_, C-1’ or C-2’), 28.1 ppm (CH_2_, C-1’ or C-2’). IR (CHCl_3_): ν = 3567, 2928, 2858, 1490, 1212 cm^–1^. MS (ESI) *m*/*z* (%) = 747 (100) [M + Na]^+^. HRMS (ESI) *m*/*z:* [M + Na]^+^ calcd for C_42_H_45_NaO_9_P 747.2699; found 747.2709.

##### Method G

Following the general procedure, starting from substrate **8** (37.1 mg, 0.043 mmol), all the starting material was consumed after 3 h. Column chromatography (hexanes–EtOAc, 8:2 to 1:1) gave **25** (6.5 mg, 0.014 mmol, 32%) and **30β** (7.8 mg, 0.011 mmol, 25%).

#### Radical Reactions of **9**

##### Method A

Following the general procedure, starting from substrate **9** (64.4 mg, 0.11 mmol), after 2 h of reaction, a supplementary addition of *n-*Bu_3_SnH (30 μL, 0.11 mmol) was required. All the starting material was consumed after 5 h. Column chromatography (hexanes to hexanes–EtOAc, 6:4) gave (4*S*)-1,4-anhydro-6,7-di-*O*-benzyl-2,3,5,9-tetradeoxy-β-l-*lyxo*-non-4-ulopyranose (**31***S*) contaminated with the thermodynamic isomer (4*R*)-1,4-anhydro-6,7-di-*O*-benzyl-2,3,5,9-tetradeoxy-α-l-*lyxo*-non-4-ulopyranose (**31***R*) (19.2 mg, 0.05 mmol, 46%, *S*/*R*, 85:15) as an amorphous solid and an inseparable mixture of 3-*C*-(2-*O*-acetyl-3,4-di-*O*-benzyl-6-deoxy-β-d-altropyranosyl)1-propanol (**32**) and 3-*C*-(2-*O*-acetyl-3,4-di-*O*-benzyl-α-l-fucopyranosyl)1-propanol (**33**) (7.4 mg, 0.017 mmol, 15%, 1:1.7) as a colorless oil. Compound **31***S*: ^1^H NMR (500 MHz, CDCl_3_, simulated ring coupling constants using DAISY) δ_H_ 7.41–7.25 (m, 10H, Ar), 4.955 (d, *J* = 12.0 Hz, 1H, OBn), 4.73 (d, *J* = 11.7 Hz, 1H, OBn), 4.63 (d, *J* = 12.0 Hz, 1H, OBn), 4.60 (d, *J* = 12.0 Hz, 1H, OBn), 3.93 (ddd, *J* = 12.1, 4.5, 2.7 Hz, 1H, 3-H), 3.87 (ddd, *J* = 8.2, 8.2, 5.4 Hz, 1H, 3’-H_b_), 3.82 (dddd, *J* = 6.5, 6.5, 6.5, 1.7 Hz, 1H, 5-H), 3.81 (ddd, *J* = 8.2, 8.2, 6.6 Hz, 1H, 3’-H_a_), 3.58 (ddd, *J* = 2.7, 1.7 Hz, ^4^*J*_2a,4_ = 1.3 Hz, 1H, 4-H), 2.32 (dd, *J* = 12.3, 12.1 Hz, 1H, 2-H_b_), 2.13–2.00 (m, 2H, 1’-H_b_, 2’-H_b_), 1.92 (dd, *J* = 12.3, 4.5 Hz, ^4^*J*_2a,4_ = 1.3 Hz, 1H, 2-H_a_), 1.90 (m, 1H, 2’-H_a_), 1.75 (m, 1H, 1’-H_a_), 1.14 ppm (d, *J* = 6.5 Hz, 3H, 6-H_3_), ^1^H NMR (500 MHz, C_6_D_6_, simulated ring coupling constants using DAISY) δ_Η_ 7.44–7.42 (m, 2H, Ar), 7.38–7.35 (m, 2H, Ar), 7.22–7.13 (m, 6H, Ar), 5.05 (d, *J* = 11.5 Hz, 1H, C_4_-OBn), 4.59 (d, *J* = 11.5 Hz, 1H, C_4_-OBn), 4.44 (d, *J* = 12.1 Hz, 1H, C_3_-OBn), 4.40 (d, *J* = 12.1 Hz, 1H, C_3_-OBn), 4.02 (ddd, *J* = 12.0, 4.5, 2.7 Hz, 1H, 3-H), 3.87 (dddd, *J* = 6.5, 6.5, 6.5, 1.4 Hz, 1H, 5-H), 3.79–3.73 (m, 1H, 3’-H_b_), 3.71–3.64 (m, 1H, 3’-H_a_), 3.36 (ddd, *J* = 2.7, 1.4 Hz, ^4^*J*_2a,4_ = 1.2 Hz, 1H, 4-H), 2.49 (dd, *J* = 12.1, 12.0 Hz, 1H, 2-H_b_), 2.03–2.00 (m, 1H), 1.94 (ddd, *J* = 12.1, 4.5 Hz, ^4^*J*_2a,4_ = 1.2 Hz, 1H, 2-H_a_), 1.86–1.78 (m, 1H), 1.46–1.40 (m, 2H), 1.28 ppm (d, *J* = 6.5 Hz, 3H, 6-H_3_). ^13^C{^1^H} NMR (125.7 MHz, CDCl_3_) δ_C_ 139.0 (C, Ar), 138.8 (C, Ar), 128.5 (2 × CH, Ar), 128.4 (2 × CH, Ar), 128.1 (2 × CH, Ar), 127.2 (2 × CH, Ar), 127.2 (2 × CH, Ar), 106.8 (C, C-1), 77.3 (CH, C-3), 75.0 (CH, C-4), 74.16 (CH_2_, OBn), 70.5 (CH_2_, OBn), 67.6 (CH, C-5), 67.1 (CH_2_, C-3’), 37.5 (CH_2_, C-1’), 34.0 (CH_2_, C-2), 23.6 (CH_2_, C-2’), 17.4 ppm (CH_3_, C-6). ^13^C{^1^H} NMR (125.7 MHz, C_6_D_6_) δ_C_ 140.3 (C, Ar) some aromatic carbons were not observed, 140.0 (C, Ar), 107.4 (C, C-1), 78.0 (CH, C-3), 76.9 (CH, C-4), 75.3 (CH_2_, OBn), 70.8 (CH_2_, OBn), 68.5 (CH, C-5), 67.6 (CH_2_, C-3’), 38.2 (CH_2_, C-1’), 34.8 (CH_2_, C-2), 24.4 (CH_2_, C-2’), 18.1 ppm (CH_3_, C-6). IR (CHCl_3_): ν = 2930, 1226, 1206 cm^–1^. MS (ESI) *m*/*z* (%) = 391 (100) [M + Na]^+^. HRMS (ESI) *m*/*z:* [M + Na]^+^ calcd for C_23_H_28_NaO_4_ 391.1885; found 391.1891. Compounds **32** and **33**: ^1^H NMR (500 MHz, CDCl_3_, selected signals of **32** from the mix spectrum) δ_H_ 4.95 (dd, *J* = 3.8, 1.6 Hz, 1H, 2-H), 4.72 (br s, 2H, OBn), 4.45 (d, *J* = 11.7 Hz, 1H, OBn), 4.34 (d, *J* = 11.7 Hz, 1H, OBn), 3.93 (dddd, *J* = 9.5, 6.3, 6.3, 6.3 Hz, 1H, 5-H), 3.88 (ddd, *J* = 9.2, 4.1, 1.3 Hz, 1H, 1-H), 3.248 (dd, *J* = 9.8, 3.2 Hz, 1H, 4-H), 1.286 ppm (d, *J* = 6.3 Hz, 3H, 6-H_3_). ^13^C{^1^H} NMR (125.7 MHz, CDCl_3_, selected signals of **32** from the mix spectrum) δ_C_ 170.4 (C, OAc), 138.0 (C, Ar), 137.7 (C, Ar), 77.67 (CH, C-4), 73.4 (CH, C-1), 72.6 (CH_2_, OBn), 71.7 (CH, C-3), 71.4 (CH, C-2), 71.3 (CH_2_, OBn), 71.2 (CH, C-5), 62.6 (CH_2_, C-3’), 27.6 (CH_2_, C-1’ or C-2’), 26.1 (CH_2_, C-1’ or C-2’), 8.19 ppm (CH_3_, C-6). ^1^H NMR (500 MHz, CDCl_3_, selected signals of **33** from the mix spectrum) δ_H_ 5.08 (dd, *J* = 5.1, 2.6 Hz, 1H, 2-H), 4.76 (d, *J* = 12.0 Hz, 1H, OBn), 4.67 (d, *J* = 12.0 Hz, 1H, OBn), 4.63 (d, *J* = 12.0 Hz, 1H, OBn), 4.54 (d, *J* = 12.0 Hz, 1H, OBn), 4.14–4.06 (m, 2H, 1-H, 5-H), 3.75 (dd, *J* = 4.8, 3.2 Hz, 1H, 4-H), 1.41 ppm (d, *J* = 6.9 Hz, 3H, 6-H_3_). ^13^C{^1^H} NMR (125.7 MHz, CDCl_3_, selected signals of **33** from the mix spectrum) δ_C_ 170.3 (C, OAc), 138.4 (C, Ar), 138.2 (C, Ar), 75.4 (CH, C-3), 74.2 (CH, C-4), 73.0 (CH_2_, OBn), 72.0 (CH_2_, OBn), 71.8 (CH, C-2), 70.0 (CH, C-5), 67.5 (CH, C-1), 62.6 (CH_2_, C-3’), 29.7 (CH_2_, C-1’ or C-2’), 29.4 (CH_2_, C-1’ or C-2’), 14.2 ppm (CH_3_, C-6). IR (CHCl_3_): ν = 3690, 3018, 1735, 1222 cm^–1^. MS (ESI) *m*/*z* (%) = 451 (100) [M + Na]^+^. HRMS (ESI) *m*/*z:* [M + Na]^+^ calcd for C_25_H_32_NaO_6_ 451.2097; found 451.2095.

##### Method D

Following the general procedure, starting from substrate **9** (59.2 mg, 0.10 mmol), after 2 h of reaction, a supplementary addition of *n-*Bu_3_SnD (28 μL, 0.10 mmol) was required. All the starting material was consumed after 4 h. Column chromatography (hexanes to hexanes–EtOAc, 6:4) gave (4*S*)-1,4-anhydro-6,7-di-*O*-benzyl-2,3,5,9-tetradeoxy-β-l-[7-*O*-PhCH-^2^H]*lyxo*-non-4-ulopyranose ([PhCH-^2^H]**31**) (19.5 mg, 0.053 mmol, 53%, ^2^H/^1^H 1.5:1) and a mixture of 3-*C*-(2-*O*-acetyl-3,4-di-*O*-benzyl-6-deoxy-β-d-(5-^2^H)altropyranosyl)1-propanol (5-^2^H)**32** and **33** (15.8 mg, 0.037 mmol, 36%, ^2^H/^1^H 1:2.8) as colorless oils. Compound [PhCH-^2^H]**31**: ^1^H NMR (500 MHz, CDCl_3_) δ_H_ 7.40–7.25 (m, 10H, Ar), 4.95 (d, *J* = 12.0 Hz, 0.4H, OBn), 4.93 (br s, 0.5H, O-CHD-Ph), 4.72 (d, *J* = 12.0 Hz, 0.4H, OBn), 4.71 (br s, 0.1H, O-CHD-Ph), 4.62 (d, *J* = 12.0 Hz, 1H, OBn), 4.59 (d, *J* = 12.0 Hz, 1H, OBn), 3.931 (ddd, *J* = 12.3, 4.4, 2.5 Hz, 0.5H, 3-H), 3.929 (ddd, *J* = 12.3, 5.0, 2.8 Hz, 0.5H, 3-H), 3.87 (ddd, *J* = 8.2, 8.2, 5.7 Hz, 1H, 3’-H_b_), 3.82 (ddd, *J* = 8.2, 8.2, 6.6 Hz, 1H, 3’-H_a_), 3.81 (m, 1H, 5-H), 3.57 (br s, 1H, 4-H), 2.32 (dd, *J* = 12.3, 12.3 Hz, 1H, 2-H_b_), 2.13–1.99 (m, 2H, 1’-H_b_, 2’-H_b_), 1.92 (ddd, *J* = 12.3, 4.7 Hz, ^4^*J*_2a,4_ = 1.0 Hz, 1H, 2-H_a_), 1.85 (m, 1H, 2’-H_a_), 1.74 (ddd, *J* = 12.3, 10.4, 7.9 Hz, 1H, 1’-H_a_), 1.135 (d, *J* = 6.3 Hz, 1.5H, 6-H_3_), 1.133 (d, *J* = 6.6 Hz, 0.3H, 6-H_3_), 1.130 ppm (d, *J* = 6.3 Hz, 1.2H, 6-H_3_). ^13^C{^1^H} NMR (125.7 MHz, CDCl_3_) δ_C_ 139.0 (0.4C, Ar), 138.9 (0.6C, Ar), 138.8 (C, Ar), 128.49 (CH, Ar), 128.45 (CH, Ar), 128.4 (2 × CH, Ar), 128.1 (2 × CH, Ar), 127.4 (2 × CH, Ar), 127.2 (2 × CH, Ar), 106.8 (C, C-1), 77.7 (CH, C-3), 75.09 (0.4CH, C-4), 75.02 (0.1CH, C-4), 74.97 (0.5CH, C-4), 74.18 (0.4CH_2_, OBn), 73.59 (t, *J*_CD_ = 22.1 Hz, 0.6CHD-Ph), 70.5 (CH_2_, OBn), 67.7 (CH, C-5), 67.1 (CH_2_, C-3’), 37.5 (CH_2_, C-1’), 34.0 (CH_2_, C-2), 23.6 (CH_2_, C-2’), 17.4 ppm (CH_3_, C-6). ^1^H NMR (500 MHz, C_6_D_6_) δ_H_ 7.44–7.42 (m, 2H, Ar), 7.36–7.35 (m, 2H, Ar), 7.22–7.10 (m, 6H, Ar), 5.046 (d, *J* = 11.6 Hz, 0.4H, OBn), 5.019 (br s, 0.5H, O-CHD-Ph), 4.59 (d, *J* = 11.5 Hz, 0.4H, OBn), 4.567 (br s, 0.1H, O-CHD-Ph), 4.44 (d, *J* = 12.1 Hz, 1H, OBn), 4.40 (d, *J* = 11.9 Hz, 1H, OBn), 4.02 (ddd, *J* = 12.1, 4.8, 2.8 Hz, 1H, 3-H), 3.89–3.85 (m, 1H, 5-H), 3.78–3.74 (m, 1H, 3’-H_b_), 3.79–3.66 (m, 1H, 3’-H_a_), 3.36 (m, 1H, 4-H), 2.496 (dd, *J* = 12.1, 12.1 Hz, 0.5H, 2-H_b_), 2.494 (dd, *J* = 12.1, 12.1 Hz, 0.5H, 2-H_b_), 2.02 (m, 1H), 1.94 (ddd, *J* = 12.6, 4.3 Hz, ^4^*J*_2a,4_ = 0.6 Hz, 1H, 2-H_a_), 1.86–1.78 (m, 1H), 1.46–1.40 (m, 2H), 1.280 (d, *J* = 6.6 Hz, 1.5H, 6-H_3_), 1.276 ppm (d, *J* = 6.6 Hz, 1.5H, 6-H_3_). ^13^C{^1^H} NMR (125.7 MHz, C_6_D_6_) δ_C_ the aromatic carbons were not observed, 107.1 ppm (C, C-1), 77.7 (CH, C-3), 76.93 (0.4CH, C-4), 76.88 (0.1CH, C-4), 76.85 (0.5CH, C-4), 75.26 (0.4CH_2_, OBn), 74.85 (t, *J*_CD_ = 22.1 Hz, 0.6CHD-Ph), 70.8 (CH_2_, OBn), 68.5 (CH, C-5), 67.6 (CH_2_, C-3’), 38.2 (CH_2_, C-1’), 34.8 (CH_2_, C-2), 24.4 (CH_2_, C-2’), 18.2 ppm (CH_3_, C-6). MS (ESI) *m*/*z* (%) = 392 (100) [M + Na]^+^, 391 (48) [M + Na]^+^. HRMS (ESI) *m*/*z:* [M + Na]^+^ calcd for C_23_H_27_^2^HNaO_4_ 392.1948; found 392.1936, [M + Na]^+^ calcd for C_23_H_28_NaO_4_ 391.1885; found 391.1891. Mixture of (5-^2^H)**32**/**33**: ^1^H NMR (400 MHz, CDCl_3_, only the deuterated product (5-^2^H)**32** is described) δ_H_ 7.40–7.22 (m, 10H, Ar), 4.95 (dd, *J* = 3.5, 1.2 Hz, 1H, 2-H), 4.72 (br s, 2H, OBn), 4.45 (d, *J* = 11.7 Hz, 1H, OBn), 4.34 (d, *J* = 11.7 Hz, 1H, OBn), 3.88 (ddd, *J* = 9.1, 5.3, 1.3 Hz, 1H, 1-H), 3.80 (m, 1H, 3-H), 3.69–3.58 (m, 2H, 3’-H_2_), 3.248 (d, *J* = 3.1 Hz, 1H, 4-H), 2.06 (s, 3H, OAc), 1.67–1.45 (m, 4H, 1’-H_2_, 2’-H_2_), 1.280 ppm (s, 3H, 6-H_3_), 1H from OH is missing. ^13^C{^1^H} NMR (100.6 MHz, CDCl_3_, only the deuterated product (5-^2^H)**32** is described) δ_C_ 170.3 (C, OAc), 138.0 (C, Ar), 137.7 (C, Ar), 127.5–128.3 (10 × CH, Ar), 77.48 (CH, C-4), 73.3 (CH, C-1), 72.7 (CH_2_, OBn), 71.8 (CH, C-3), 71.4 (CH, C-2), 71.4 (CH_2_, OBn), 62.6 (CH_2_, C-3’), 27.6 (CH_2_, C-1’ or C-2’), 26.1 (CH_2_, C-1’ or C-2’), 21.0 (CH_3_, OAc), 18.07 ppm (CH_3_, C-6). MS (ESI) *m*/*z* (%) = 452 (100) [M + Na]^+^, 451 (75) [M + Na]^+^. HRMS (ESI) *m*/*z:* [M + Na]^+^ calcd for C_25_H_31_^2^HNaO_6_ 452.2159; found 452.2159, [M + Na]^+^ calcd for C_25_H_32_NaO_6_ 451.2097; found 451.2098.

##### Method F

Following the general procedure, starting from substrate **9** (17.9 mg, 0.031 mmol), all the starting material was consumed after 3 h. Column chromatography (hexanes–EtOAc, 6:4 to 4:6) gave **33** (8.7 mg, 0.020 mmol, 65%).

##### Method G

Following the general procedure, starting from substrate **9** (18.2 mg, 0.032 mmol), all the starting material was consumed after 3 h. Column chromatography (hexanes–EtOAc, 6:4 to 4:6) gave **33** (13.1 mg, 0.031 mmol, 67%).

#### Radical Reactions of **10**

##### Method A

Following the general procedure, starting from substrate **10** (95.8 mg, 0.13 mmol), after 2 h of reaction, a supplementary addition of *n-*Bu_3_SnH (15 μL, 0.07 mmol) was required. All the starting material was consumed after 4 h. Column chromatography (hexanes to hexanes–EtOAc, 4:6) gave **31** (24.7 mg, 0.07 mmol, 52%, *S*/*R*, 83:17), 2,6:5,9-di-anhydro-3,4-di-*O*-benzyl-1,7,8-trideoxy-d-*glycero*-d-*galacto*-nonitol (**36**) (4.6 mg, 0.013 mmol, 10%), 3-*C*-(3,4-di-*O*-benzyl-6-deoxy-2-*O*-diphenoxyphosphoryl-β-d-altropyranosyl)1-propanol (**34**) (5.1 mg, 0.008 mmol, 6%), and 3-*C*-(3,4-di-*O*-benzyl-2-*O*-diphenoxyphosphoryl-α-l-fucopyranosyl)1-propanol (**35**) (9.7 mg, 0.016 mmol, 12%), all as colorless oils. Compound **36**: [α]_D_ = +2.2 (*c =* 0.27, CHCl_3_). ^1^H NMR (500 MHz, CDCl_3_, simulated ring coupling constants using DAISY) δ_H_ 7.42–7.27 (m, 10H, Ar), 4.98 (d, *J* = 11.7 Hz, 1H, OBn), 4.88 (d, *J* = 12.3 Hz, 1H, OBn), 4.71 (d, *J* = 11.7 Hz, 1H, OBn), 4.70 (d, *J* = 12.3 Hz, 1H, OBn), 3.98 (m, 1H, 3’-H_b_), 3.629 (dd, *J* = 9.8, 9.1 Hz, 1H, 2-H), 3.62 (dd, *J* = 3.0, 1.3 Hz, 1H, 4-H), 3.52 (dd, *J* = 9.8, 3.0 Hz, 1H, 3-H), 3.51 (dddd, *J* = 6.4, 6.4, 6.4, 1.3 Hz, 1H, 5-H), 3.46 (m, 1H, 3’-H_a_), 3.06 (ddd, *J* = 11.1, 9.1, 4.3 Hz, 1H, 1-H), 2.038 (m, 1H, 1’-H_b_), 1.75–1.70 (m, 2H, 2’-H_2_), 1.611 (m, 1H, 1’-H_a_), 1.16 ppm (d, *J* = 6.4 Hz, 3H, 6-H_3_). ^13^C{^1^H} NMR (125.7 MHz, CDCl_3_) δ_C_ 139.0 (C, Ar), 138.7 (C, Ar), 128.6 (2 × CH, Ar), 128.4 (2 × CH, Ar), 128.2 (2 × CH, Ar), 127.6 (CH, Ar), 127.52 (2 × CH, Ar), 127.47 (CH, Ar), 81.9 (CH, C-3), 79.60 (CH, C-2 or C-4), 77.88 (CH, C-2 or C-4), 76.4 (CH, C-1), 74.9 (CH, C-5), 74.9 (CH_2_, OBn), 73.0 (CH_2_, OBn), 67.9 (CH_2_, C-3’), 29.12 (CH_2_, C-1’), 25.6 (CH_2_, C-2’), 17.3 ppm (CH_3_, C-6). IR (CHCl_3_): ν = 3017, 1454, 1220 cm^–1^. MS (ESI) *m*/*z* (%) = 391 (100) [M + Na]^+^. HRMS (ESI) *m*/*z:* [M + Na]^+^ calcd for C_23_H_28_NaO_4_ 391.1885; found 391.1878. Compound **34**: [α]_D_ = +10.0 (*c =* 0.50, CHCl_3_). ^1^H NMR (500 MHz, CDCl_3_) δ_H_ 7.37–7.20 (m, 20H, Ar), 4.68 (d, *J* = 12.0 Hz, 1H, OBn), 4.62 (d, *J* = 12.0 Hz, 1H, OBn), 4.59 (m, 1H, 2-H), 4.21 (d, *J* = 11.7 Hz, 1H, OBn), 4.11 (d, *J* = 11.7 Hz, 1H, OBn), 4.03 (dd, *J* = 3.2, 3.2 Hz, 1H, 3-H), 3.90–3.81 (m, 2H, 1-H, 5-H), 3.57–3.54 (m, 2H, 3’-H_2_), 3.240 (dd, *J* = 9.8, 2.9 Hz, 1H, 4-H), 1.63–1.50 (m, 4H, 1’-H_2_, 2’-H_2_), 1.262 ppm (d, *J* = 6.3 Hz, 3H, 6-H_3_), 1H from OH is missing. ^13^C{^1^H} NMR (125.7 MHz, CDCl_3_) δ_C_ 150.5 (2 × C, Ar), 137.8 (2 × C, Ar), 129.9 (2 × CH, Ar), 129.8 (2 × CH, Ar), 128.4 (2 × CH, Ar), 128.3 (2 × CH, Ar), 128.1 (2 × CH, Ar), 127.9 (CH, Ar), 127.7 (3 × CH, Ar), 125.6 (2 × CH, Ar), 120.2 (CH, Ar), 120.1 (CH, Ar), 120.09 (CH, Ar), 120.06 (CH, Ar), 77.99 (CH, C-4), 77.3 (CH, C-2), 73.5 (d, ^3^*J*_PC_ = 6.4 Hz, CH, C-1), 73.0 (CH_2_, OBn), 72.2 (CH, C-3), 71.5 (CH_2_, OBn), 71.1 (CH, C-5), 62.6 (CH_2_, C-3’), 29.3 (CH_2_, C-1’), 27.5 (CH_2_, C-2’), 18.23 ppm (CH_3_, C-6). IR (CHCl_3_): ν = 3426, 3022, 1490, 1220 cm^–1^. MS (ESI) *m*/*z* (%) = 641 (100) [M + Na]^+^. HRMS (ESI) *m*/*z:* [M + Na]^+^ calcd for C_35_H_39_NaO_8_P 641.2280; found 641.2288. Compound **35**: [α]_D_ = −24.8 (*c =* 0.72, CHCl_3_). ^1^H NMR (500 MHz, CDCl_3_) δ_H_ 7.33–7.16 (m, 20H, Ar), 4.83 (ddd, *J* = 6.0, 2.8 Hz, ^3^*J*_PH_ = 8.5 Hz, 1H, 2-H), 4.70 (d, *J* = 11.7 Hz, 1H, OBn), 4.63 (d, *J* = 11.7 Hz, 1H, OBn), 4.54 (d, *J* = 12.0 Hz, 1H, OBn), 4.41 (d, *J* = 11.7 Hz, 1H, OBn), 4.09–4.04 (m, 2H, 1-H, 5-H), 3.92 (dd, *J* = 5.7, 3.2 Hz, 1H, 3-H), 3.721 (dd, *J* = 3.8, 3.8 Hz, 1H, 4-H), 3.55 (br s, 2H, 3’-H_2_), 1.84 (br s, 1H, OH), 1.63–1.52 (m, 4H, 1’-H_2_, 2’-H_2_), 1.361 ppm (d, *J* = 6.9 Hz, 3H, 6-H_3_). ^13^C{^1^H} NMR (125.7 MHz, CDCl_3_) δ_C_ 150.5 (d, ^2^*J*_PC_ = 7.4 Hz, C, Ar), 150.5 (d, ^2^*J*_PC_ = 7.4 Hz, C, Ar), 138.2 (C, Ar), 138.0 (C, Ar), 129.81 (2 × CH, Ar), 129.76 (2 × CH, Ar), 128.4 (2 × CH, Ar), 128.3 (2 × CH, Ar), 127.7 (3 × CH, Ar), 127.62 (CH, Ar), 127.56 (2 × CH, Ar), 125.4 (2 × CH, Ar), 120.2 (CH, Ar), 120.12 (CH, Ar), 120.06 (CH, Ar), 120.0 (CH, Ar), 77.5 (CH, C-2), 75.9 (CH, C-3), 74.65 (CH, C-4), 73.2 (CH_2_, OBn), 72.4 (CH_2_, OBn), 69.6 (CH, C-5), 68.5 (d, ^3^*J*_PC_ = 4.2 Hz, CH, C-1), 62.4 (CH_2_, C-3’), 29.7 (CH_2_, C-1’), 29.2 (CH_2_, C-2’), 14.44 ppm (CH_3_, C-6). IR (CHCl_3_): ν = 3454, 3015, 1490, 1205 cm^–1^. MS (ESI) *m*/*z* (%) = 641 (100) [M + Na]^+^. HRMS (ESI) *m*/*z:* [M + Na]^+^ calcd for C_35_H_39_NaO_8_P 641.2280; found 641.2278.

##### Method D

Following the general procedure, starting from substrate **10** (85.2 mg, 0.11 mmol), after 2 h of reaction, a supplementary addition of *n-*Bu_3_SnD (15 μL, 0.07 mmol) was required. All the starting material was consumed after 4 h. Column chromatography (hexanes to hexanes–EtOAc, 4:6) gave [PhCH-^2^H]**31** (20.1 mg, 0.05 mmol, 49%, ^2^H/^1^H 1.7:1), 2,6:5,9-di-anhydro-3,4-di-*O*-benzyl-1,7,8-trideoxy-d-[6-^2^H]*glycero*-d-*galacto*-nonitol [(1-^2^H)**36**] (4.5 mg, 0.012 mmol, 11%, ^2^H/^1^H 8:1), and 3-*C*-(3,4-di-*O*-benzyl-6-deoxy-2-*O*-diphenoxyphosphoryl-β-d-(5-^2^H)altropyranosyl)1-propanol [(5-^2^H)**34**] (4.3 mg, 0.007 mmol, 6%) as colorless oils and **35** (6 mg, 0.01 mmol, 9%). Compound (1-^2^H)**36**: ^1^H NMR (500 MHz, only the deuterated product is described) δ_H_ 7.42–7.26 (m, 10H, Ar), 4.98 (d, *J* = 11.7 Hz, 1H, OBn), 4.88 (d, *J* = 12.3 Hz, 1H, OBn), 4.71 (d, *J* = 11.7 Hz, 1H, OBn), 4.70 (d, *J* = 12.3 Hz, 1H, OBn), 3.98 (m, 1H, 3’-H_b_), 3.637 (d, *J* = 9.8 Hz, 1H, 2-H), 3.62 (dd, *J* = 2.8, 1.0 Hz, 1H, 4-H), 3.52 (dd, *J* = 9.8, 2.8 Hz, 1H, 3-H), 3.51 (dddd, *J* = 6.3, 6.3, 6.3, 1.3 Hz, 1H, 5-H), 3.46 (m, 1H, 3’-H_a_), 2.029 (m, 1H, 1’-H_b_), 1.74–1.70 (m, 2H, 2’-H_2_), 1.606 (m, 1H, 1’-H_a_), 1.16 ppm (d, *J* = 6.7 Hz, 3H, 6-H_3_). ^13^C{^1^H} NMR (125.7 MHz, CDCl_3_, only the deuterated product is described) δ_C_ 138.9 (C, Ar), 138.6 (C, Ar), 128.5 (2 × CH, Ar), 128.3 (2 × CH, Ar), 128.1 (2 × CH, Ar), 127.54 (CH, Ar), 127.49 (2 × CH, Ar), 127.4 (CH, Ar), 81.9 (CH, C-3), 79.49 (CH, C-2), 77.84 (CH, C-4), 74.8 (CH, C-5), 74.8 (CH_2_, OBn), 73.0 (CH_2_, OBn), 67.9 (CH_2_, C-3’), 28.99 (CH_2_, C-1’), 25.6 (CH_2_, C-2’), 17.3 ppm (CH_3_, C-6). MS (ESI) *m*/*z* (%) = 392 (100) [M + Na]^+^, 391 (12) [M + Na]^+^. HRMS (ESI) *m*/*z:* [M + Na]^+^ calcd for C_23_H_27_^2^HNaO_4_ 392.1948; found 392.1954. Compound (5-^2^H)**34**: ^1^H NMR (500 MHz, CDCl_3_) δ_H_ 7.37–7.20 (m, 20H, Ar), 4.68 (d, *J* = 12.0 Hz, 1H, OBn), 4.62 (d, *J* = 12.0 Hz, 1H, OBn), 4.57 (m, 1H, 2-H), 4.20 (d, *J* = 11.7 Hz, 1H, OBn), 4.11 (d, *J* = 11.7 Hz, 1H, OBn), 4.02 (dd, *J* = 3.5, 3.5 Hz, 1H, 3-H), 3.83 (m, 1H, 1-H), 3.57–3.54 (m, 2H, 3’-H_2_), 3.235 (d, *J* = 2.9 Hz, 1H, 4-H), 2.00 (br s, 1H, OH), 1.64–1.50 (m, 4H, 1’-H_2_, 2’-H_2_), 1.248 ppm (s, 3H, 6-H_3_). ^13^C{^1^H} NMR (125.7 MHz, CDCl_3_) δ_C_ 150.5 (2 × C, Ar), 137.9 (2 × C, Ar), 129.9 (2 × CH, Ar), 129.8 (2 × CH, Ar), 128.4 (2 × CH, Ar), 128.3 (2 × CH, Ar), 128.1 (2 × CH, Ar), 127.9 (CH, Ar), 127.7 (2 × CH, Ar), 127.4 (CH, Ar), 125.5 (2 × CH, Ar), 120.15 (CH, Ar), 120.10 (CH, Ar), 120.08 (CH, Ar), 120.04 (CH, Ar), 77.91 (CH, C-4), 77.3 (CH, C-2), 73.5 (d, ^3^*J*_PC_ = 6.4 Hz, CH, C-1), 73.0 (CH_2_, OBn), 72.2 (CH, C-3), 71.5 (CH_2_, OBn), 62.6 (CH_2_, C-3’), 29.3 (CH_2_, C-1’), 27.5 (CH_2_, C-2’), 18.10 ppm (CH_3_, C-6). MS (ESI) *m*/*z* (%) = 642 (100) [M + Na]^+^. HRMS (ESI) *m*/*z:* [M + Na]^+^ calcd for C_35_H_38_^2^HNaO_8_P 642.2280; found 642.2291.

#### Radical Reactions of **11**

##### Method A

Following the general procedure, starting from substrate **11** (68.2 mg, 0.10 mmol), after 2 h of reaction, a supplementary addition of *n-*Bu_3_SnH (29 μL, 0.10 mmol) was required. All the starting material was consumed after 3 h. Column chromatography (hexanes to hexanes–EtOAc, 9:1) gave (4*S*)-1,4-anhydro-6,7-*O*-benzylidene-2,3,5-trideoxy-d-*erythro*-oct-4-ulopyranose (**37**) (15.8 mg, 0.06 mmol, 60%) as a colorless oil: [α]_D_ = −109.3 (*c =* 0.28, CHCl_3_). ^1^H NMR (500 MHz, CDCl_3_, simulated ring coupling constants using DAISY) δ_H_ 7.48–7.46 (m, 2H, Ar), 7.40–7.35 (m, 3H, Ar), 6.20 (s, 1H, PhC*H*), 4.700 (ddd, *J* = 8.7, 6.3, 5.5 Hz, 1H, 3-H), 4.10 (ddd, *J* = 5.5, 2.4, 1.0 Hz, 1H, 4-H), 4.03 (dd, *J* = 13.3, 1.0 Hz, 1H, 5-H_b_), 4.02 (dd, *J* = 13.3, 2.4 Hz, 1H, 5-H_a_), 3.95 (ddd, *J* = 8.2, 8.2, 5.7 Hz, 1H, 3’-H_b_), 3.88 (ddd, *J* = 8.2, 8.2, 6.3 Hz, 1H, 3’-H_a_), 2.11 (dd, *J* = 13.5, 8.7 Hz, 1H, 2-H_b_), 2.05 (dd, *J* = 13.5, 6.3 Hz, 1H, 2-H_a_), 2.18–2.04 (m, 2H, 1’-H_b_, 2’-H_b_), 1.93 (m, 1H, 2’-H_a_), 1.80 ppm (ddd, *J* = 12.3, 10.1, 7.9 Hz, 1H, 1’-H_a_). ^13^C{^1^H} NMR (125.7 MHz, CDCl_3_) δ_C_ 139.3 (C, Ar), 128.9 (CH, Ar), 128.4 (2 × CH, Ar), 126.1 (2 × CH, Ar), 105.75 (C, C-1), 102.6 (CH, PhC*H*), 71.83 (CH, C-4), 71.72 (CH, C-3), 67.4 (CH_2_, C-3’), 60.6 (CH_2_, C-5), 37.9 (CH_2_, C-1’), 34.0 (CH_2_, C-2), 23.6 ppm (CH_2_, C-2’). IR (CHCl_3_): ν = 3024, 1458, 1210 cm^–1^. MS (ESI) *m*/*z* (%) = 285 (100) [M + Na]^+^. HRMS (ESI) *m*/*z:* [M + Na]^+^ calcd for C_15_H_18_NaO_4_ 285.1103; found 285.1103. Anal. calcd for C_15_H_18_O_4_: C, 68.68; H, 6.92. Found: C, 68.68; H, 7.00.

##### Method D

Following the general procedure, starting from substrate **11** (72.6 mg, 0.11 mmol), after 2 h, all the starting material was consumed. Column chromatography (hexanes to hexanes–EtOAc, 9:1) gave (4*S*)-1,4-anhydro-6,7-*O*-benzylidene-2,3,5-trideoxy-d-[5-^2^H]*erythro*-oct-4-ulopyranose ([2-^2^H]**37**) (15.7 mg, 0.06 mmol, 54%, ^2^H/^1^H 1:1) as a colorless oil: ^1^H NMR (500 MHz, CDCl_3_, only the deuterated compound is described) δ_H_ 7.48–7.46 (m, 2H, Ar), 7.40–7.34 (m, 3H, Ar), 6.20 (s, 1H, PhC*H*), 4.701 (dd, *J* = 8.6, 5.4 Hz, 1H, 3-H), 4.11 (ddd, *J* = 5.4, 2.3, 1.3 Hz, 1H, 4-H), 4.03 (dd, *J* = 13.6, 1.0 Hz, 1H, 5-H_b_), 4.00 (dd, *J* = 13.3, 2.2 Hz, 1H, 5-H_a_), 3.94 (ddd, *J* = 8.2, 8.2, 5.7 Hz, 1H, 3’-H_b_), 3.88 (ddd, *J* = 8.2, 8.2, 6.3 Hz, 1H, 3’-H_a_), 2.18–2.04 (m, 3H, 2-H, 1’-H_b_, 2’-H_b_), 1.92 (m, 1H, 2’-H_a_), 1.79 ppm (ddd, *J* = 12.3, 10.1, 7.9 Hz, 1H, 1’-H_a_). ^13^C{^1^H} NMR (125.7 MHz, CDCl_3_, only the deuterated compound is described) δ_C_ 139.3 (C, Ar), 128.9 (CH, Ar), 128.4 (2 × CH, Ar), 126.1 (2 × CH, Ar), 105.72 (C, C-1), 102.5 (CH, PhC*H*), 71.80 (CH, C-3 or C-4), 71.67 (CH, C-3 or C-4), 67.4 (CH_2_, C-3’), 60.6 (CH_2_, C-5), 37.9 (CH_2_, C-1’), 33.69 (t, *J*_CD_ = 20.1 Hz, CHD, C-2), 23.6 ppm (CH_2_, C-2’). MS (ESI) *m*/*z* (%) = 286 (100) [M + Na]^+^, 285 (83) [M + Na]^+^. HRMS (ESI) *m*/*z:* [M + Na]^+^ calcd for C_15_H_17_^2^HNaO_4_ 286.1166; found 286.1154, [M + Na]^+^ calcd for C_15_H_18_NaO_4_ 285.1103; found 285.1107.

##### Method E

Following the general procedure, starting from substrate **11** (60 mg, 0.091 mmol), after 2 h, all the starting material was consumed. Column chromatography (hexanes to hexanes–EtOAc, 9:1) gave [2-^2^H]**37** (14.4 mg, 0.055 mmol, 60%, ^2^H/^1^H 1:1).

#### Radical Reactions of **12**

##### Method A

Following the general procedure, starting from substrate **12** (65.2 mg, 0.11 mmol), all the starting material was consumed after 2 h. Column chromatography (DCM to DCM–MeOH, 97:3) gave (4*S*)-1,4-anhydro-2,3,5-trideoxy-d-*erythro*-oct-4-ulopyranose (**38**) (10.4 mg, 0.06 mmol, 54%) as a colorless oil: [α]_D_ = −57.3 (*c =* 0.62, CHCl_3_). ^1^H NMR (400 MHz, CDCl_3_, simulated ring coupling constants using DAISY) δ_H_ 4.055 (ddd, *J* = 11.7, 5.3, 3.5 Hz, 1H, 3-H), 3.90 (dd, *J* = 12.5, 1.6 Hz, 1H, 5-H_b_), 3.96–3.83 (m, 2H, 3’-H_2_), 3.80 (br s, 1H, 4-H), 3.72 (dd, *J* = 12.5, 2.3 Hz, 1H, 5-H_a_), 2.31 (br s, 1H, OH), 2.17 (br s, 1H, OH), 2.09–2.01 (m, 2H, 1’-H_b_, 2’-H_b_), 1.99 (dd, *J* = 12.8, 11.7 Hz, 1H, 2-H_b_), 1.88 (m, 1H, 2’-H_a_), 1.86 (dd, *J* = 12.8, 5.3 Hz, 1H, 2-H_a_), 1.72 ppm (m, 1H, 1’-H_a_). ^13^C{^1^H} NMR (125.7 MHz, CDCl_3_) δ_C_ 106.63 (C, C-1), 67.9 (CH, C-4), 67.5 (CH_2_, C-3’), 66.59 (CH, C-3), 63.7 (CH_2_, C-5), 37.5 (CH_2_, C-1’), 36.83 (CH_2_, C-2), 23.3 ppm (CH_2_, C-2’). IR (CHCl_3_): ν = 3685, 3571, 3020, 1056 cm^–1^. MS (ESI) *m*/*z* (%) = 197 (100) [M + Na]^+^. HRMS (ESI) *m*/*z:* [M + Na]^+^ calcd for C_8_H_14_NaO_4_ 197.0790; found 197.0789. Anal. calcd for C_8_H_14_O_4_: C, 55.16; H, 8.10. Found: C, 54.97; H, 7.92.

##### Method D

Following the general procedure, starting from substrate **12** (64.2 mg, 0.11 mmol), after 2 h, all the starting material was consumed. Column chromatography (DCM to DCM–MeOH, 97:3) gave (4*S*)-1,4-anhydro-2,3,5-trideoxy-d-[5-^2^H]*erythro*-oct-4-ulopyranose ([2-^2^H]**38**) (12.2 mg, 0.07 mmol, 63%, ^2^H/^1^H 2.3:1) as a colorless oil: ^1^H NMR (500 MHz, CDCl_3_, only the deuterated compound is described) δ_H_ 4.049 (m, 1H, 3-H), 3.95–3.85 (m, 2H, 3’-H_2_), 3.90 (dd, *J* = 12.6, 1.3 Hz, 1H, 5-H_b_), 3.80 (br s, 1H, 4-H), 3.72 (dd, *J* = 12.6, 2.2 Hz, 1H, 5-H_a_), 2.22 (br s, 1H, OH), 2.09–2.00 (m, 2H, 1’-H_b_, 2’-H_b_), 1.92–1.85 (m, 2H, 2-H, 2’-H_a_), 1.72 ppm (m, 1H, 1’-H_a_), 1H from OH is missing. ^13^C{^1^H} NMR (125.7 MHz, CDCl_3_, only the deuterated compound is described) δ_C_ 106.57 (C, C-1), 67.9 (CH_2_, C-4), 67.5 (CH_2_, C-3’), 66.52 (CH, C-3), 63.7 (CH, C-5), 37.4 (CH_2_, C-1’), 23.3 ppm (CH_2_, C-2’), C-2 was undetectable. MS (ESI) *m*/*z* (%) = 198 (100) [M + Na]^+^, 197 (44) [M + Na]^+^. HRMS (ESI) *m*/*z:* [M + Na]^+^ calcd for C_8_H_13_^2^HNaO_4_ 198.0853; found 198.0856, [M + Na]^+^ calcd for C_8_H_14_NaO_4_ 197.0790; found 197.0796.

### Synthesis of 9-Deoxy-1,6-dioxaspiro[4.4]nonane structures (Table 4)

#### Radical Reactions of **13**

##### Method A

Following the general procedure, starting from substrate **13** (102 mg, 0.12 mmol), after 2 h of reaction, a supplementary addition of *n-*Bu_3_SnH (32 μL, 0.12 mmol) was required. All the starting material was consumed after 3 h. Column chromatography on a silica gel without KF (hexanes to hexanes–EtOAc, 8:2) gave 3-*C*-(2-*O*-acetyl-3,5-di-*O*-*tert*-butyldiphenylsilyl-α-d-ribofuranosyl)1-propanol (**40**) (51.2 mg, 0.072 mmol, 56%) as a colorless oil: [α]_D_ = +46.0 (*c =* 0.61, CHCl_3_). ^1^H NMR (500 MHz, CDCl_3_) δ_H_ 7.65–7.26 (m, 20H, Ar), 5.12 (dd, *J* = 4.4, 4.4 Hz, 1H, 2-H), 4.61 (dd, *J* = 6.7, 4.5 Hz, 1H, 3-H), 4.03 (m, 1H, 4-H), 3.97 (m, 1H, 1-H), 3.66–3.60 (m, 3H, 5-H_b_, 3’-H_2_), 3.32 (dd, *J* = 11.4, 3.5 Hz, 1H, 5-H_a_), 2.13 (s, 3H, OAc), 1.69–1.53 (m, 4H, 1’-H_2_, 2’-H_2_), 1.02 (s, 9H, *^t^*Bu), 0.91 ppm (s, 9H, *^t^*Bu). ^13^C{^1^H} NMR (125.7 MHz, CDCl_3_) δ_C_ 170.4 (C, OAc). 135.78 (2 × CH, Ar), 135.72 (2 × CH, Ar), 135.60 (2 × CH, Ar), 135.55 (2 × CH, Ar), 133.3 (C, Ar), 133.24 (C, Ar), 133.22 (C, Ar), 132.7 (C, Ar), 129.98 (CH, Ar), 129.96 (CH, Ar), 129.5 (2 × CH, Ar), 127.76 (2 × CH, Ar), 127.72 (2 × CH, Ar), 127.56 (2 × CH, Ar), 127.54 (2 × CH, Ar), 82.8 (CH, C-4), 79.5 (CH, C-1), 75.0 (CH, C-2), 72.8 (CH, C-3), 63.6 (CH_2_, C-5), 62.6 (CH_2_, C-3’), 29.6 (CH_2_, C-1’ or C-2’), 26.8 (3 × CH_3_, *^t^*Bu), 26.7 (3 × CH_3_, *^t^*Bu), 26.5 (CH_2_, C-1’ or C-2’), 21.0 (CH_3_, OAc), 19.2 (C, *^t^*Bu), 19.1 ppm (C, *^t^*Bu). IR (CHCl_3_): ν = 3451, 2932, 1735, 1216, 1113 cm^–1^. MS (ESI) *m*/*z* (%) = 733 (100) [M + Na]^+^. HRMS (ESI) *m*/*z:* [M + Na]^+^ calcd for C_42_H_54_NaO_6_Si_2_ 733.3357; found 733.3360. Anal. calcd for C_42_H_54_O_6_Si_2_: C, 70.95; H, 7.66. Found: C, 71.05; H, 7.82.

##### Method D

Following the general procedure, starting from substrate **13** (98.4 mg, 0.12 mmol), after 2 h of reaction and again after 4 h, a supplementary addition of *n-*Bu_3_SnD (31 μL, 0.12 mmol) was required. All the starting material was consumed after 5 h. Column chromatography on a silica gel without KF (hexanes to hexanes–EtOAc, 8:2) gave 3-*C*-(2-*O*-acetyl-3,5-di-*O*-*tert*-butyldiphenylsilyl-α-d-[1-^2^H]ribofuranosyl)1-propanol ([1-^2^H]**40**) (46.1 mg, 0.065 mmol, 54%, ^2^H/^1^H 2:1) as a colorless oil: ^1^H NMR (500 MHz, CDCl_3_, only the deuterated compound is described) δ_H_ 7.65–7.26 (m, 20H, Ar), 5.117 (d, *J* = 4.7 Hz, 1H, 2-H), 4.61 (dd, *J* = 6.9, 4.7 Hz, 1H, 3-H), 4.03 (m, 1H, 4-H), 3.66–3.60 (m, 3H, 5-H_b_, 3’-H_2_), 3.31 (dd, *J* = 11.4, 3.5 Hz, 1H, 5-H_a_), 2.13 (s, 3H, OAc), 1.69–1.53 (m, 4H, 1’-H_2_, 2’-H_2_), 1.02 (s, 9H, *^t^*Bu), 0.91 ppm (s, 9H, *^t^*Bu). ^13^C{^1^H} NMR (100.6 MHz, CDCl_3_) δ_C_ 170.4 (C, OAc), 135.79 (2 × CH, Ar), 135.73 (2 × CH, Ar), 135.61 (2 × CH, Ar), 135.56 (2 × CH, Ar), 133.4 (C, Ar), 133.28 (C, Ar), 133.25 (C, Ar), 132.7 (C, Ar), 129.98 (CH, Ar), 129.95 (CH, Ar), 129.5 (2 × CH, Ar), 127.76 (2 × CH, Ar), 127.72 (2 × CH, Ar), 127.56 (2 × CH, Ar), 127.54 (2 × CH, Ar), 82.8 (CH, C-4), 74.96 (CH, C-2), 72.8 (CH, C-3), 63.7 (CH_2_, C-5), 62.6 (CH_2_, C-3’), 29.6 (CH_2_, C-1’ or C-2’), 26.8 (3 × CH_3_, *^t^*Bu), 26.7 (3 × CH_3_, *^t^*Bu), 26.5 (CH_2_, C-1’ or C-2’), 21.0 (CH_3_, OAc), 19.2 (C, *^t^*Bu), 19.1 ppm (C, *^t^*Bu). MS (ESI) *m*/*z* (%) = 734 (100) [M + Na]^+^, 733 (34) [M + Na]^+^. HRMS (ESI) *m*/*z:* [M + Na]^+^ calcd for C_42_H_53_^2^HNaO_6_Si_2_ 734.3419; found 734.3417, [M + Na]^+^ calcd for C_42_H_54_NaO_6_Si_2_ 733.3357; found 733.3351.

##### Method E

Following the general procedure, starting from substrate **13** (128.6 mg, 0.15 mmol), after 2 h, a supplementary addition of *n-*Bu_3_SnD (41 μL, 0.15 mmol) and BF_3_•Et_2_O (4 μL, 0.03 mmol) was required. All the starting material was consumed after 5 h. Column chromatography on a silica gel without KF (hexanes to hexanes–EtOAc, 75:25) gave (4*RS*)-1,4-anhydro-5-*O*-acetyl-6,8-bis-*O*-*tert*-butyldiphenylsilyl-2,3-dideoxy-d-[5-^2^H]*ribo*-oct-4-ulofuranose ([2-^2^H]**39**) (39.1 mg, 0.06 mmol, 40%, ^2^H/^1^H 1.3:1, 1*R*/1*S* 1:1) as a colorless oil and (1-^2^H)**40** (13.9 mg, 0.02 mmol, 13%). Compound [2-^2^H]**39**: ^1^H NMR (500 MHz, CDCl_3_, only nondeuterated products of both isomers are described) δ_H_ 7.72–7.25 (m, 40H, Ar), 4.44 (m, 2H, 3-H), 4.12–4.08 (m, 2H, 4-H), 4.00 (ddd, *J* = 8.5, 8.5, 5.4 Hz, 1H, 3’-H_b_), 3.90 (ddd, *J* = 7.6, 7.6, 7.6 Hz, 1H, 3’-H_a_), 3.83 (ddd, *J* = 8.2, 8.2, 4.5 Hz, 1H, 3’-H_b_), 3.70 (ddd, *J* = 7.6, 7.6, 7.6 Hz, 1H, 3’-H_a_), 3.62 (dd, *J* = 11.4, 2.6 Hz, 1H, 5-H_b_), 3.50 (dd, *J* = 10.8, 5.4 Hz, 1H, 5-H_b_), 3.45 (dd, *J* = 11.0, 5.7 Hz, 1H, 5-H_a_), 3.40 (dd, *J* = 11.0, 3.5 Hz, 1H, 5-H_a_), 2.22 (dd, *J* = 9.5, 9.5 Hz, 1H, 1’-H_b_), 2.12 (m, 1H, 2’-H_b_), 2.10–1.96 (m, 6H, 1’-H_b_, 2 × 2’-H_2_, 2’-H_a_, 2 × 2-H_b_), 1.88–1.81 (m, 3H, 1’-H_a_, 2 × 2-H_a_), 1.73 (ddd, *J* = 12.0, 8.9, 8.9 Hz, 1H, 1’-H_a_), 1.06 (s, 18H, *^t^*Bu), 0.96 (s, 9H, *^t^*Bu), 0.93 ppm (s, 9H, *^t^*Bu). ^13^C{^1^H} NMR (125.7 MHz, CDCl_3_, only nondeuterated products of both isomers are described) δ_C_ 134.07 (C, Ar), 133.87 (C, Ar), 133.81 (C, Ar), 133.63 (2 × C, Ar), 133.61 (C, Ar), 133.59 (C, Ar), 133.5 (C, Ar), 127.5–135.9 (40 × CH, Ar), 114.8 (C, C-1), 114.2 (C, C-1), 86.9 (CH, C-4), 85.9 (CH, C-4), 74.1 (CH, C-3), 72.9 (CH, C-3), 67.4 (CH_2_, C-3’), 67.1 (CH_2_, C-3’), 65.0 (CH_2_, C-5), 63.4 (CH_2_, C-5), 44.3 (CH_2_, C-2’), 43.5 (CH_2_, C-2’), 36.8 (CH_2_, C-1’), 36.1 (CH_2_, C-1’), 27.0 (3 × CH_3_, *^t^*Bu), 26.9 (3 × CH_3_, *^t^*Bu), 26.8 (3 × CH_3_, *^t^*Bu), 26.7 (3 × CH_3_, *^t^*Bu), 24.22 (CH_2_, C-2), 24.20 (CH_2_, C-2), 19.19 (C, *^t^*Bu), 19.16 (2 × C, *^t^*Bu), 19.1 ppm (C, *^t^*Bu). IR (CHCl_3_): ν = 2932, 1428, 1222, 1113 cm^–1^. MS (ESI) *m*/*z* (%) = 674 (100) [M + Na]^+^, 673 (33) [M + Na]^+^. HRMS (ESI) *m*/*z:* [M + Na]^+^ calcd for C_40_H_49_^2^HNaO_4_Si_2_ 674.3208; found 674.3206, [M + Na]^+^ calcd for C_40_H_50_NaO_4_Si_2_ 673.3145; found 673.3163.

##### Method F

Following the general procedure, starting from substrate **13** (40.8 mg, 0.048 mmol), all the starting material was consumed after 75 h. Chromatotron chromatography (hexanes–EtOAc, 7:3) gave (4*RS*)-1,4-anhydro-5-*O*-acetyl-6,8-bis-*O*-*tert*-butyldiphenylsilyl-2,3-dideoxy-d-*ribo*-oct-4-ulofuranose (**39**) (0.6 mg, 9.6·10^–4^ mmol, 2%, 1*R*/1*S* 1:1) and **40** (22.8 mg, 0.032 mmol, 67%) as colorless oils. Compound **39**: ^1^H NMR (500 MHz, CDCl_3_, descrived above for the [2-^2^H]**39**). ^13^C{^1^H} NMR (125.7 MHz, CDCl_3_, descrived above for the [2-^2^H]**39**). MS (ESI) *m*/*z* (%) = 673 (100) [M + Na]^+^. HRMS (ESI) *m*/*z:* [M + Na]^+^ calcd for C_40_H_50_NaO_4_Si_2_: 673.3145 [M + Na]^+^; found 673.3146. C_40_H_50_NaO_4_Si_2_ (650.99): calcd. C 73.80, H 7.74; found: C 73.70, H 7.74.

#### Radical Reactions of **14**

##### Method A

Following the general procedure, starting from substrate **14** (56.9 mg, 0.06 mmol), after 2 h of reaction, a supplementary addition of *n-*Bu_3_SnH (16 μL, 0.06 mmol) was required. All the starting material was consumed after 4 h. Column chromatography on a silica gel without KF (hexanes to hexanes–EtOAc, 7:3) gave **39** (18 mg, 0.028 mmol, 46%, 1*R*/1*S* 1.2:1).

##### Method D

Following the general procedure, starting from substrate **14** (54.5 mg, 0.06 mmol), after 2 h of reaction, a supplementary addition of *n-*Bu_3_SnD (16 μL, 0.06 mmol) was required. All the starting material was consumed after 5 h. Column chromatography on a silica gel without KF (hexanes to hexanes–EtOAc, 7:3) gave [2-^2^H]**39** (13 mg, 0.02 mmol, 35%, ^2^H/^1^H 1:1.4).

##### Method E

Following the general procedure, starting from substrate **14** (49.8 mg, 0.05 mmol), after 4 h, the reaction was discarded since although the remaining starting material was present, several more polar products were detected in the TLC.

##### Method F

Following the general procedure, starting from substrate **14** (57.4 mg, 0.06 mmol), all the starting material was consumed after 0.75 h. Chromatotron chromatography (hexanes–EtOAc, 97:3 to 7:3) gave **39** (4.8 mg, 0.007 mmol, 12%, 1*R*/1*S* 1.2:1) and 3-*C*-(3,5-di-*O*-*tert*-butyldiphenylsilyl-2-*O*-trifluoromethanesulfonyl-α-d-ribofuranosyl)1-propanol (**41**) (20.3 mg, 0.025 mmol, 42%) as a colorless oil. Compound **41**: [α]_D_ = +18.8 (*c =* 1.3, CHCl_3_). ^1^H NMR (500 MHz, CDCl_3_) δ_H_ 7.45–7.22 (m, 20H, Ar), 5.26 (dd, *J* = 4.1, 4.1 Hz, 1H, 2-H), 4.67 (dd, *J* = 4.8, 4.8 Hz, 1H, 3-H), 4.17 (ddd, *J* = 10.1, 3.2, 3.2 Hz, 1H, 1-H), 3.95 (ddd, *J* = 5.4, 2.9, 2.9 Hz, 1H, 4-H), 3.65 (m, 2H, 3’-H_2_), 3.34 (dd, *J* = 11.7, 2.2 Hz, 1H, 5-H_b_), 2.75 (dd, *J* = 11.7, 3.2 Hz, 1H, 5-H_a_), 1.86 (m, 1H, 1’-H_b_), 1.77–1.65 (m, 3H, 1’-H_a_, 2’-H_2_), 1.06 (s, 9H, *^t^*Bu), 0.87 ppm (s, 9H, *^t^*Bu). ^13^C{^1^H} NMR (125.7 MHz, CDCl_3_) δ_C_ 135.9 (2 × CH, Ar), 135.8 (2 × CH, Ar), 135.5 (4 × CH, Ar), 133.1 (C, Ar), 132.96 (C, Ar), 132.95 (C, Ar), 131.7 (C, Ar), 130.15 (CH, Ar), 130.10 (CH, Ar), 129.6 (2 × CH, Ar), 127.9 (2 × CH, Ar), 127.8 (2 × CH, Ar), 127.6 (4 × CH, Ar), 89.1 (CH, C-2), 82.8 (CH, C-4), 78.4 (CH, C-1), 73.3 (CH, C-3), 63.7 (CH_2_, C-5), 62.4 (CH_2_, C-3’), 29.4 (CH_2_, C-1’), 26.7 (3 × CH_3_, *^t^*Bu), 26.6 (3 × CH_3_, *^t^*Bu), 26.5 (CH_2_, C-2’), 19.2 (C, *^t^*Bu), 19.0 ppm (C, *^t^*Bu), 1C from CF_3_ group is missing. IR (CHCl_3_): ν = 3694, 3429, 3020, 2933, 2254, 1778, 1740, 1224, 1113 cm^–1^. MS (ESI) *m*/*z* (%) = 823 (100) [M + Na]^+^. HRMS (ESI) *m*/*z:* [M + Na]^+^ calcd for C_41_H_51_F_3_NaO_7_SSi_2_ 823.2744; found 823.2750. Anal. calcd for C_41_H_51_F_3_O_7_SSi_2_: C, 61.47; H, 6.42; S, 4.00. Found: C, 61.20; H, 6.44; S, 3.62.

#### Radical Reactions of **15**

##### Method A

Following the general procedure, starting from substrate **15** (93.8 mg, 0.12 mmol), after 2 h of reaction, a supplementary addition of *n-*Bu_3_SnH (31 μL, 0.12 mmol) was required. All the starting material was consumed after 3 h. Column chromatography on a silica gel without KF (hexanes to hexanes–EtOAc, 97:3) gave (4*R*)-1,4-anhydro-2,3,5-trideoxy-6,8-bis-*O*-(1,1,3,3-tetraisopropyldisiloxanyl)-d-*erythro*-oct-4-ulofuranose (**42**) (13 mg, 0.031 mmol, 27%) and (4*S*)-1,4-anhydro-2,3,5-trideoxy-6,8-bis-*O*-(1,1,3,3-tetraisopropyldisiloxanyl)-d-*erythro*-oct-4-ulofuranose (**43**) (16.8 mg, 0.040 mmol, 35%), both as colorless oils. Compound **42**: [α]_D_ = −56.6 (*c =* 0.53, CHCl_3_). ^1^H NMR (500 MHz, CDCl_3_) δ_H_ 4.64 (ddd, *J* = 8.9, 7.3, 5.4 Hz, 1H, 3-H), 3.95 (dd, *J* = 10.1, 2.5 Hz, 1H, 5-H_b_), 3.89 (ddd, *J* = 8.2, 8.2, 5.4 Hz, 1H, 3’-H_b_), 3.84–3.77 (m, 3H, 4-H, 5-H_a_, 3’-H_a_), 2.35 (dd, *J* = 12.3, 7.3 Hz, 1H, 2-H_b_), 2.18 (dd, *J* = 12.7, 8.9 Hz, 1H, 2-H_a_), 2.06 (ddd, *J* = 11.7, 11.7, 3.2 Hz, 1H, 1’-H_b_), 2.02 (m, 1H, 2’-H_b_), 1.93–1.82 (m, 2H, 1’-H_a_, 2’-H_a_), 1.10–0.99 ppm (m, 28H, *^i^*Pr). ^13^C{^1^H} NMR (125.7 MHz, CDCl_3_) δ_C_ 113.1 (C, C-1), 84.4 (CH, C-4), 74.7 (CH, C-3), 67.3 (CH_2_, C-3’), 66.1 (CH_2_, C-5), 44.0 (CH_2_, C-2), 34.9 (CH_2_, C-1’), 23.9 (CH_2_, C-2’), 17.6 (CH_3_, *^i^*Pr), 17.4 (3 × CH_3_, *^i^*Pr), 17.3 (CH_3_, *^i^*Pr), 17.1 (CH_3_, *^i^*Pr), 17.02 (CH_3_, *^i^*Pr), 16.99 (CH_3_, *^i^*Pr), 13.4 (2 × CH, *^i^*Pr), 12.8 (CH, *^i^*Pr), 12.6 ppm (CH, *^i^*Pr). IR (CHCl_3_): ν = 2947, 2868, 1464, 1136, 1035 cm^–1^. MS (ESI) *m*/*z* (%) = 439 (100) [M + Na]^+^. HRMS (ESI) *m*/*z:* [M + Na]^+^ calcd for C_20_H_40_NaO_5_Si_2_ 439.2312; found 439.2308. Anal. calcd for C_20_H_40_O_5_Si_2_: C, 57.65; H, 9.68. Found: C, 57.39; H, 9.46. Compound **43**: [α]_D_ = +20.8 (*c =* 0.89, CHCl_3_). ^1^H NMR (500 MHz, CDCl_3_) δ_H_ 4.33 (ddd, *J* = 8.2, 8.2, 6.9 Hz, 1H, 3-H), 3.99 (dd, *J* = 11.4, 2.2 Hz, 1H, 5-H_b_), 3.94–3.89 (m, 2H, 3’-H_2_), 3.86–3.80 (m, 2H, 4-H, 5-H_a_), 2.38 (dd, *J* = 13.3, 8.2 Hz, 1H, 2-H_b_), 2.23 (dd, *J* = 13.2, 7.3 Hz, 1H, 2-H_a_), 2.08–2.01 (m, 2H, 1’-H_b_, 2’-H_b_), 1.91–1.82 (m, 2H, 1’-H_a_, 2’-H_a_), 1.10–0.99 ppm (m, 28H, *^i^*Pr). ^13^C{^1^H} NMR (125.7 MHz, CDCl_3_) δ_C_ 112.7 (C, C-1), 82.9 (CH, C-4), 71.5 (CH, C-3), 67.3 (CH_2_, C-3’), 62.3 (CH_2_, C-5), 43.3 (CH_2_, C-2), 36.5 (CH_2_, C-1’), 24.2 (CH_2_, C-2’), 17.5 (CH_3_, *^i^*Pr), 17.36 (2 × CH_3_, *^i^*Pr), 17.35 (CH_3_, *^i^*Pr), 17.27 (CH_3_, *^i^*Pr), 17.2 (CH_3_, *^i^*Pr), 17.0 (CH_3_, *^i^*Pr), 16.9 (CH_3_, *^i^*Pr), 13.5 (CH, *^i^*Pr), 13.2 (CH, *^i^*Pr), 12.8 (CH, *^i^*Pr), 12.6 ppm (CH, *^i^*Pr). IR (CHCl_3_): ν = 2947, 2868, 1465, 1210, 1133, 1043 cm^–1^. MS (ESI) *m*/*z* (%) = 439 (100) [M + Na]^+^. HRMS (ESI) *m*/*z:* [M + Na]^+^ calcd for C_20_H_40_NaO_5_Si_2_ 439.2312; found 439.2312. Anal. calcd for C_20_H_40_O_5_Si_2_: C, 57.65; H, 9.68. Found: C, 57.39; H, 9.46.

##### Method D

Following the general procedure, starting from substrate **15** (93.7 mg, 0.12 mmol), after 2 h of reaction, a supplementary addition of *n-*Bu_3_SnD (31 μL, 0.12 mmol) was required. All the starting material was consumed after 6 h. Column chromatography on a silica gel without KF (hexanes to hexanes–EtOAc, 97:3) gave (4*R*)-1,4-anhydro-2,3,5-trideoxy-6,8-bis-*O*-(1,1,3,3-tetraisopropyldisiloxanyl)-β-d-[5-^2^H]*erythro*-oct-4-ulofuranose ([2-^2^H]**42**) (11.7 mg, 0.02 mmol, 24%, ^2^H/^1^H 1.2:1) and (4*S*)-1,4-anhydro-2,3,5-trideoxy-6,8-bis-*O*-(1,1,3,3-tetraisopropyldisiloxanyl)-β-d-[5-^2^H]*erythro*-oct-4-ulofuranose ([2-^2^H]**43**) (10 mg, 0.024 mmol, 21%, ^2^H/^1^H 1.5:1), which was obtained as a 1:1.2 mixture with [2-^2^H]**42**. Compound [2-^2^H]**42**: ^1^H NMR (500 MHz, CDCl_3_, only deuterated 2*RS* isomers are described) δ_H_ 4.64 (m, 1H, 3-H), 3.96–3.87 (m, 2H, 5-H_b_, 3’-H_b_), 3.84–3.77 (m, 3H, 4-H, 5-H_a_, 3’-H_a_), 2.336 (d, *J* = 7.3 Hz, 1H, 2-H, 2*R* isomer), 2.166 (d, *J* = 9.2 Hz, 1H, 2-H, 2*S* isomer), 2.06 (ddd, *J* = 11.4, 11.4, 2.9 Hz, 1H, 1’-H_b_), 2.01 (m, 1H, 2’-H_b_), 1.93–1.83 (m, 2H, 1’-H_a_, 2’-H_a_), 1.10–0.99 ppm (m, 28H, *^i^*Pr). ^13^C{^1^H} NMR (125.7 MHz, CDCl_3_, only deuterated 2*RS* isomers are described) δ_C_ 113.1 (C, C-1), 84.4 (CH, C-4), 74.60 (CH, C-3), 67.3 (CH_2_, C-3’), 66.1 (CH_2_, C-5), 43.68 (t, *J*_CD_ = 22.2 Hz, CHD, C-2), 34.9 (CH_2_, C-1’), 23.9 (CH_2_, C-2’), 17.6 (CH_3_, *^i^*Pr), 17.4 (3 × CH_3_, *^i^*Pr), 17.3 (CH_3_, *^i^*Pr), 17.1 (CH_3_, *^i^*Pr), 17.02 (CH_3_, *^i^*Pr), 16.99 (CH_3_, *^i^*Pr), 13.4 (2 × CH, *^i^*Pr), 12.8 (CH, *^i^*Pr), 12.6 ppm (CH, *^i^*Pr). MS (ESI) *m*/*z* (%) = 440 (100) [M + Na]^+^, 439 (68) [M + Na]^+^. HRMS (ESI) *m*/*z:* [M + Na]^+^ calcd for C_20_H_39_^2^HNaO_5_Si_2_ 440.2375; found 440.2372, [M + Na]^+^ calcd for C_20_H_40_NaO_5_Si_2_ 439.2312; found 439.2300. Compound [2-^2^H]**43**: ^1^H NMR (500 MHz, CDCl_3_, only deuterated 2*RS* isomers are described) δ_H_ 4.33 (ddd, *J* = 8.2, 8.2, 6.9 Hz, 1H, 3-H), 3.99 (dd, *J* = 11.4, 2.2 Hz, 1H, 5-H_b_), 3.94–3.89 (m, 2H, 3’-H_2_), 3.86–3.80 (m, 2H, 4-H, 5-H_a_), 2.365 (d, *J* = 8.5 Hz, 1H, 2-H), 2.228 (d, *J* = 6.9 Hz, 1H, 2-H), 2.08–2.01 (m, 2H, 1’-H_b_, 2’-H_b_), 1.91–1.82 (m, 2H, 1’-H_a_, 2’-H_a_), 1.10–0.99 ppm (m, 28H, *^i^*Pr). ^13^C{^1^H} NMR (125.7 MHz, CDCl_3_) δ_C_ 112.7 (C, C-1), 82.9 (CH, C-4), 71.5 (CH, C-3), 67.3 (CH_2_, C-3’), 62.3 (CH_2_, C-5), 42.95 (t, *J*_CD_ = 21.2 Hz, CHD, C-2), 36.5 (CH_2_, C-1’), 24.2 (CH_2_, C-2’), 17.5 (CH_3_, *^i^*Pr), 17.4 (2 × CH_3_, *^i^*Pr), 17.3 (2 × CH_3_, *^i^*Pr), 17.2 (CH_3_, *^i^*Pr), 17.0 (CH_3_, *^i^*Pr), 16.9 (CH_3_, *^i^*Pr), 13.5 (CH, *^i^*Pr), 13.2 (CH, *^i^*Pr), 12.8 (CH, *^i^*Pr), 12.6 ppm (CH, *^i^*Pr). IR (CHCl_3_): ν = 2947, 2868, 1465, 1210, 1133, 1043 cm^–1^. MS (ESI) *m*/*z* (%) = 440 (100) [M + Na]^+^, 439 (55) [M + Na]^+^. HRMS (ESI) *m*/*z:* [M + Na]^+^ calcd for C_20_H_39_^2^HNaO_5_Si_2_ 440.2375; found 440.2374, [M + Na]^+^ calcd for C_20_H_40_NaO_5_Si_2_ 439.2312; found 439.2306.

##### Method F

Following the general procedure, starting from substrate **15** (53.8 mg, 0.066 mmol), all the starting material was consumed after 0.5 h. Chromatotron chromatography (hexanes–EtOAc, 95:5 to 1:1) gave **42** and **43** (7.3 mg, 0.018 mmol, 26%, 1*R*/1*S* 1:2.5), and 3-*C*-(2-*O*-diphenoxyphosphoryl-3,5-bis-*O*-(1,1,3,3-tetraisopropyldisiloxanyl)-α-d-ribofuranosyl)1-propanol (**44**) (9.5 mg, 0.014 mmol, 22%) as a colorless oil. Compound **44**: [α]_D_ = +12.9 (*c =* 0.71, CHCl_3_). ^1^H NMR (500 MHz, CDCl_3_) δ_H_ 7.33–7.15 (m, 10H, Ar), 5.13 (ddd, *J* = 3.8, 3.8 Hz, ^3^*J*_PH_ = 7.9 Hz, 1H, 2-H), 4.47 (m, 1H, 3-H), 4.12 (m, 1H, 1-H), 4.00 (dd, *J* = 12.6, 2.8 Hz, 1H, 5-H_b_), 3.95–3.91 (m, 2H, 4-H, 5-H_a_), 3.51–3.49 (m, 2H, 3’-H_2_), 1.66–1.51 (m, 4H, 1’-H_2_, 2’-H_2_), 1.09–0.81 ppm (m, 28H, *^i^*Pr), 1H from OH is missing. ^13^C{^1^H} NMR (125.7 MHz, CDCl_3_) δ_C_ 150.9 (d, ^2^*J*_PC_ = 7.4 Hz, C, Ar), 150.6 (d, ^2^*J*_PC_ = 7.4 Hz, C, Ar), 129.7 (2 × CH, Ar), 129.6 (2 × CH, Ar), 125.3 (CH, Ar), 125.1 (CH, Ar), 120.19 (CH, Ar), 120.14 (CH, Ar), 120.0 (CH, Ar), 119.9 (CH, Ar), 81.6 (d, ^2^*J*_PC_ = 6.4 Hz, CH, C-2), 79.8 (CH, C-4), 79.5 (d, ^3^*J*_PC_ = 6.3 Hz, CH, C-1), 71.6 (CH, C-3), 62.5 (CH_2_, C-3’), 60.9 (CH_2_, C-5), 29.2 (CH_2_, C-1’ or C-2’), 26.7 (CH_2_, C-1’ or C-2’), 17.4 (CH_3_, *^i^*Pr), 17.28 (CH_3_, *^i^*Pr), 17.27 (CH_3_, *^i^*Pr), 17.25 (CH_3_, *^i^*Pr), 17.0 (2 × CH_3_, *^i^*Pr), 16.8 (CH_3_, *^i^*Pr), 16.7 (CH_3_, *^i^*Pr), 13.5 (CH, *^i^*Pr), 13.1 (CH, *^i^*Pr), 12.6 (CH, *^i^*Pr), 12.4 ppm (CH, *^i^*Pr). IR (CHCl_3_): ν = 3692, 3610, 3022, 2948, 1490.1210, 1039 cm^–1^. MS (ESI) *m*/*z* (%) = 689 (100) [M + Na]^+^. HRMS (ESI) *m*/*z:* [M + Na]^+^ calcd for C_32_H_51_NNaO_9_PSi_2_ 689.2707; found 689.2706. Anal. calcd for C_32_H_51_NO_9_PSi_2_: C, 57.63; H, 7.71. Found: C, 57.61; H, 8.07.

### Synthesis of 4-Deoxy-6,8-dioxabicyclo[3.2.1]heptane Structures (Tables 5 and 6)

#### Radical Reactions of **16**

##### Method A

Following the general procedure, starting from substrate **16** (49 mg, 0.076 mmol), after 2 h of reaction, a supplementary addition of *n-*Bu_3_SnH (20 μL, 0.076 mmol) was required. All the starting material was consumed after 5 h. Column chromatography (hexanes to hexanes–EtOAc, 6:4) gave (2*S*)-2,7-anhydro-1-*O*-*tert*-butyldiphenylsilyl-3-deoxy-4,5-di-*O*-methyl-β-d-*xylo*-hept-2-ulopyranose (**45**) (3.1 mg, 0.007 mmol, 9%), an inseparable mixture of **48** (7.6 mg, 0.015 mmol, 20%) and unstable *C*-(6-*O*-*tert*-butyldiphenylsilyl-4-deoxy-2,3-di-*O*-methyl-β-l-*threo*-hex-4-enopyranosyl)methanol (**46**) (1.5 mg, 0.03 mmol, 4%), and *C*-(4-*O*-acetyl-6-*O*-*tert*-butyldiphenylsilyl-2,3-di-*O*-methyl-β-l-idopyranosyl)methanol (**47**) (12.1 mg, 0.024 mmol, 32%), all as colorless oils. Compound **45**: [α]_D_ = +14.5 (*c* = 0.38, CHCl_3_). ^1^H NMR (500 MHz, CDCl_3_, simulated ring coupling constants using DAISY) δ_H_ 7.70–7.68 (m, 4H, Ar), 7.45–7.37 (m, 6H, Ar), 4.56 (ddd, *J* = 5.0, 4.3, 0.0 Hz, 1H, 1-H), 4.03 (dd, *J* = 7.6, 0.0 Hz, 1H, 1’-H_b_), 3.75 (d, *J* = 11.0 Hz, 1H, 6-H_b_), 3.73 (d, *J* = 10.7 Hz, 1H, 6-H_a_), 3.68 (dd, *J* = 7.5, 5.0 Hz, ^4^*J*_2,1_’_a_ = 1.1 Hz, 1H, 1’-H_a_), 3.571 (ddd, *J* = 10.1, 8.2, 6.6 Hz, 1H, 3-H), 3.50 (s, 3H, OMe), 3.42 (s, 3H, OMe), 3.40 (ddd, *J* = 8.2, 4.4 Hz,^4^*J*_2,1_’_a_ = 1.1 Hz, 1H, 2-H), 2.36 (dd, *J* = 13.0, 6.6 Hz, 1H, 4-H_b_), 1.70 (dd, *J* = 13.0, 10.1 Hz, 1H, 4-H_a_), 1.08 ppm (s, 9H, *^t^*Bu). ^13^C{^1^H} NMR (100.6 MHz, CDCl_3_) δ_C_ 135.68 (2 × CH, Ar), 135.67 (2 × CH, Ar), 133.2 (2 × C, Ar), 129.7 (2 × CH, Ar), 127.7 (4 × CH, Ar), 107.94 (C, C-5), 81.0 (CH, C-2), 77.78 (CH, C-3), 73.7 (CH, C-1), 66.8 (CH_2_, C-6), 65.8 (CH_2_, C-1’), 58.4 (CH_3_, OMe), 57.2 (CH_3_, OMe), 37.08 (CH_2_, C-4), 26.8 (3 × CH_3_, DPS), 19.3 ppm (C, DPS). IR (CHCl_3_): ν = 2931, 1464, 1113 cm^–1^. MS (ESI) *m*/*z* (%) = 465 (100) [M + Na]^+^. HRMS (ESI) *m*/*z:* [M + Na]^+^ calcd for C_25_H_34_NaO_5_Si 465.2073; found 465.2071. Anal. calcd for C_25_H_34_O_5_Si: C, 67.84; H, 7.74. Found: C, 67.63; H, 7.68. Compound **46**: could not be purified perfectly due to its instability. ^1^H NMR (500 MHz, CDCl_3_, simulated coupling constants of the allylic system using DAISY) δ_H_ 7.74–7.34 (m, 10H, Ar), 5.23 (dddd, *J* = 4.9 Hz, ^4^*J* = 1.5, 1.5, 1.0 Hz, 1H, 4-H), 4.16 (ddd, *J* = 13.9 Hz, ^4^*J* = 1.0 Hz, ^5^*J* = 1.6 Hz, 1H, 6-H_b_), 4.12 (ddd, *J* = 13.9 Hz, ^4^*J* = 1.5 Hz, ^5^*J* = 0.7 Hz, 1H, 6-H_a_), 3.98–3.92 (m, 2H, 1-H, 1’-H_b_), 3.81 (m, 1H, 1’-H_a_), 3.72 (dddd, *J* = 4.9, 2.3 Hz, ^5^*J* = 1.6, 0.7 Hz, 1H, 3-H), 3.454 (s, 3H, OMe), 3.450 (m, 1H, 2-H), 3.42 (s, 3H, OMe), 1.08 ppm (s, 9H, *^t^*Bu), 1H from OH is missing. ^13^C{^1^H} NMR (100.6 MHz, CDCl_3_) δ_C_ 156.0 (C, C-5), 135.58 (2 × CH, Ar), 135.56 (2 × CH, Ar), 133.3 (2 × C, Ar), 129.7 (2 × CH, Ar), 127.7 (4 × CH, Ar), 92.8 (CH, C-4), 76.5 (CH, C-3), 74.1 (CH, C-1), 69.4 (CH, C-2), 62.7 (2 × CH_2_, C-1, C-6), 58.0 (CH_3_, OMe), 55.4 (CH_3_, OMe), 26.8 (3 × CH_3_, DPS), 19.3 ppm (C, DPS). IR (CHCl_3_): ν = 3674, 3504, 2931, 1113 cm^–1^. MS (ESI) *m*/*z* (%) = 465 (100) [M + Na]^+^. HRMS (ESI) *m*/*z:* [M + Na]^+^ calcd for C_25_H_34_NaO_5_Si 465.2073; found 465.2061. Compound **47**: [α]_D_ = +0.4 (*c* = 1.20, CHCl_3_). ^1^H NMR (500 MHz, CDCl_3_, simulated ring coupling constants using DAISY) δ_H_ 7.64–7.61 (m, 4H, Ar), 7.45–7.35 (m, 6H, Ar), 5.074 (ddd, *J* = 2.6, 1.9, ^4^*J*_2,4_ = 1.2 Hz, 1H, 4-H), 4.01 (ddd, *J* = 9.1, 5.2, 1.6 Hz, 1H, 5-H), 3.94 (dd, *J* = 11.7, 8.0 Hz, 1H, 1’-H_b_), 3.81 (dd, *J* = 9.8, 5.2 Hz, 1H, 6-H_b_), 3.79 (ddd, *J* = 8.0, 4.0, 1.6 Hz, 1H, 1-H), 3.78 (dd, *J* = 11.7, 9.1 Hz, 1H, 6-H_a_), 3.74 (dd, *J* = 2.7, 2.6 Hz, 1H, 3-H), 3.63 (dd, *J* = 11.7, 4.0 Hz, 1H, 1’-H_a_), 3.55 (s, 3H, OMe), 3.36 (s, 3H, OMe), 3.20 (ddd, *J* = 2.7, 1.6, ^4^*J*_2,4_ = 1.2 Hz, 1H, 2-H), 2.03 (s, 3H, OAc), 1.85 (br s, 1H, OH), 1.04 ppm (s, 9H, *^t^*Bu). ^13^C{^1^H} NMR (100.6 MHz, CDCl_3_) δ_C_ 170.8 (C, OAc), 135.6 (2 × CH, Ar), 135.5 (2 × CH, Ar), 133.3 (C, Ar), 133.2 (C, Ar), 129.74 (CH, Ar), 129.72 (CH, Ar), 127.7 (4 × CH, Ar), 76.3 (CH, C-1 or C-2), 76.1 (CH, C-1 or C-2), 74.6 (CH, C-5), 71.7 (CH, C-3), 66.20 (CH, C-4), 62.6 (CH_2_, C-1’), 61.62 (CH_2_, C-6), 58.1 (CH_3_, OMe), 58.0 (CH_3_, OMe), 26.8 (3 × CH_3_, DPS), 21.0 (CH_3_, OAc), 19.1 ppm (C, DPS). IR (CHCl_3_): ν = 3675, 3594, 2933, 1731, 1103 cm^–1^. MS (ESI) *m*/*z* (%) = 525 (100) [M + Na]^+^. HRMS (ESI) *m*/*z:* [M + Na]^+^ calcd for C_27_H_38_NaO_7_Si 525.2285; found 525.2276. Anal. calcd for C_27_H_38_O_7_Si: C, 64.51; H, 7.62. Found: C, 64.81; H, 7.86.

##### Method C

Following the general procedure, starting from substrate **16** (54.5 mg, 0.084 mmol), after 2 h of reaction, a supplementary addition of TTMSS (26 μL, 0.084 mmol) was required. All the starting material was consumed after 7 h. Column chromatography (hexanes–EtOAc, 9:1 to 7:3) gave **45** (4.1 mg, 0.009 mmol, 11%) and **46** (10.8 mg, 0.024 mmol, 29%).

##### Method D

Following the general procedure, starting from substrate **16** (69.9 mg, 0.11 mmol), after 2 h of reaction, a supplementary addition of *n-*Bu_3_SnD (29 μL, 0.11 mmol) was required. All the starting material was consumed after 4 h. Column chromatography (hexanes to hexanes–EtOAc, 1:1) gave 2,7-anhydro-1-*O*-*tert*-butyldiphenylsilyl-3-deoxy-4,5-di-*O*-methyl-β-d-[3-^2^H]*xylo*-hept-2-ulopyranose ([4-^2^H]**45**) (5.3 mg, 0.011 mmol, 10%, ^2^H/^1^H 1.8:1, 4*R*/4*S* 1:1.2), an inseparable mixture of reduced alcohol **48** (7.6 mg, 0.015 mmol, 14%) and olefin **46** (10.7 mg, 0.024 mmol, 22%), and *C*-(4-*O*-acetyl-6-*O*-*tert*-butyldiphenylsilyl-2,3-di-*O*-methyl-β-l-(5-^2^H)idopyranosyl)methanol [(5-^2^H)**47**] (18.3 mg, 0.036 mmol, 33%), all as colorless oils. Compound [4-^2^H]**45**: ^1^H NMR (500 MHz, CDCl_3_, only deuterated 4*RS* isomers are described) δ_H_ 7.70–7.65 (m, 4H, DPS), 7.45–7.37 (m, 6H, DPS), 4.56 (ddd, *J* = 4.4, 4.4, 0.0 Hz, 1H, 1-H), 4.03 (dd, *J* = 7.3, 0.0 Hz, 1H, 1’-H_b_), 3.75 (d, *J* = 11.0 Hz, 1H, 6-H_b_), 3.73 (d, *J* = 11.0 Hz, 1H, 6-H_a_), 3.70–3.66 (m, 1H, 1’-H_a_), 3.60–3.54 (m, 1H, 3-H), 3.50 (s, 3H, OMe), 3.43 (s, 3H, OMe), 3.42 (dd, *J* = 8.8, 3.5 Hz, 1H, 2-H), 2.35 (d, *J* = 6.6 Hz, 1H, 4-H, 4*R* isomer), 1.69 (d, *J* = 10.1 Hz, 1H, 4-H, 4*S* isomer), 1.08 ppm (s, 18H, *^t^*Bu). ^13^C{^1^H} NMR (100.6 MHz, CDCl_3_, only deuterated 4*RS* isomers are described) δ_C_ 135.68 (2 × CH, DPS), 135.66 (2 × CH, DPS), 133.27 (C, DPS), 133.20 (C, DPS), 129.7 (2 × CH, DPS), 127.7 (4 × CH, DPS), 107.90 (C, C-5), 81.0 (CH, C-2), 77.73 (CH, C-3, 4*R* or 4*S* isomer), 77.70 (CH, C-3, 4*R* or 4*S* isomer), 73.7 (CH, C-1), 66.8 (CH_2_, C-6), 65.8 (CH_2_, C-1’), 58.4 (CH_3_, OMe), 57.2 (CH_3_, OMe), 36.75 (t, *J*_CD_ = 19.1 Hz, CHD, C-4), 26.8 (3 × CH_3_, DPS), 19.3 ppm (C, DPS). MS (ESI) *m*/*z* (%) = 466 (100) [M + Na]^+^, 465 (46) [M + Na]^+^. HRMS (ESI) *m*/*z:* [M + Na]^+^ calcd for C_25_H_33_^2^HNaO_5_Si 466.2136; found 466.2141, [M + Na]^+^ calcd for C_25_H_34_NaO_5_Si 465.2073; found 465.2060. Compound (5-^2^H)**47**: ^1^H NMR (400 MHz, CDCl_3_, simulated ring coupling constants using DAISY) δ_H_ 7.64–7.61 (m, 4H, Ar), 7.45–7.35 (m, 6H, Ar), 5.070 (dd, *J* = 2.7 Hz, ^4^*J*_2,4_ = 1.2 Hz, 1H, 4-H), 3.93 (dd, *J* = 11.7, 8.1 Hz, 1H, 1’-H_b_), 3.81 (d, *J* = 9.6 Hz, 1H, 6-H_a_), 3.79 (d, *J* = 9.6 Hz, 1H, 6-H_b_), 3.79 (ddd, *J* = 8.1, 4.0, 1.6 Hz, 1H, 1-H), 3.73 (dd, *J* = 2.7, 2.7 Hz, 1H, 3-H), 3.62 (dd, *J* = 11.7, 4.0 Hz, 1H, 1’-H_a_), 3.55 (s, 3H, OMe), 3.36 (s, 3H, OMe), 3.19 (ddd, *J* = 2.7, 1.6 Hz, ^4^*J*_2,4_ = 1.2 Hz, 1H, 2-H), 2.03 (s, 3H, OAc), 1.04 ppm (s, 9H, *^t^*Bu), 1H from OH is missing. ^13^C{^1^H} NMR (100.6 MHz, CDCl_3_) δ_C_ 170.8 (C, OAc), 135.6 (2 × CH, Ar), 135.5 (2 × CH, Ar), 133.3 (C, Ar), 133.2 (C, Ar), 129.74 (CH, Ar), 129.71 (CH, Ar), 127.7 (4 × CH, Ar), 76.2 (CH, C-1 or C-2), 76.1 (CH, C-1 or C-2), 71.7 (CH, C-3), 66.14 (CH, C-4), 62.6 (CH_2_, C-1’), 61.54 (CH_2_, C-6), 58.1 (CH_3_, OMe), 58.0 (CH_3_, OMe), 26.8 (3 × CH_3_, DPS), 21.0 (CH_3_, OAc), 19.1 ppm (C, DPS). MS (ESI) *m*/*z* (%) = 526 (100) [M + Na]^+^. HRMS (ESI) *m*/*z:* [M + Na]^+^ calcd for C_27_H_37_^2^HNaO_7_Si 526.2347; found 526.2346.

##### Method E

Following the general procedure, starting from substrate **16** (38.3 mg, 0.059 mmol), after 2 h, a supplementary addition of *n-*Bu_3_SnD (16 μL, 0.059 mmol) and BF_3_•Et_2_O (2 μL, 0.012 mmol) was required. All the starting material was consumed after 4 h. Column chromatography (hexanes to hexanes–EtOAc, 1:1) gave [4-^2^H]**45** (10.3 mg, 0.023 mmol, 39%, ^2^H/^1^H 2.9:1, 4*R*/4*S* 1:1.2), an inseparable mixture of **48** (4.6 mg, 0.009 mmol, 15%) and unstable **46** (4.6 mg, 0.010 mmol, 18%), and (5-^2^H)**47** (7.3 mg, 0.015 mmol, 25%).

#### Radical Reactions of **17**

##### Method A

Following the general procedure, starting from substrate **17** (49 mg, 0.058 mmol), after 2 h of reaction, a supplementary addition of *n-*Bu_3_SnH (16 μL, 0.058 mmol) was required. All the starting material was consumed after 5 h. Column chromatography on a silica gel without KF (hexanes to hexanes–EtOAc, 9:1) gave **45** (14 mg, 0.032 mmol, 55%).

##### Method D

Following the general procedure, starting from substrate **17** (59.6 mg, 0.071 mmol), after 2 h of reaction, a supplementary addition of *n-*Bu_3_SnD (19 μL, 0.071 mmol) was required. All the starting material was consumed after 4 h. Column chromatography (hexanes to hexanes–EtOAc, 9:1) gave [4-^2^H]**45** (18.1 mg, 0.041 mmol, 58%, ^2^H/^1^H 2.3:1, 4*R*/4*S* 1:1.2).

#### Radical Reactions of **18**

##### Method A

Following the general procedure, starting from substrate **18** (38 mg, 0.05 mmol), after 2 h of reaction, a supplementary addition of *n-*Bu_3_SnH (13 μL, 0.05 mmol) was required. All the starting material was consumed after 6 h. Column chromatography on a silica gel without KF (hexanes to hexanes–EtOAc, 9:1) gave **45** (11.7 mg, 0.027 mmol, 53%).

##### Method D

Following the general procedure, starting from substrate **18** (38.8 mg, 0.05 mmol), after 2 h of reaction, a supplementary addition of *n-*Bu_3_SnD (14 μL, 0.05 mmol) was required. All the starting material was consumed after 4 h. Column chromatography (hexanes to hexanes–EtOAc, 9:1) gave [4-^2^H]**45** (13 mg, 0.029 mmol, 58%, ^2^H/^1^H 1.7:1, 4*R*/4*S* 1:1.2).

##### Method F

Following the general procedure, starting from substrate **18** (12.4 mg, 0.016 mmol), all the starting material was consumed after 1.5 h. Chromatotron chromatography (hexanes–EtOAc, 8:2 to 4:6) gave **45** (1.4 mg, 0.003 mmol, 19%) and **49** (4.1 mg, 0.007 mmol, 41%).

##### Method G

Following the general procedure, starting from substrate **18** (14.1 mg, 0.019 mmol), all the starting material was consumed after 3 h. Chromatotron chromatography (hexanes–EtOAc, 8:2 to 4:6) gave **45** (3.2 mg, 0.007 mmol, 39%) and **49** (2.3 mg, 0.004 mmol, 20%).

#### Radical Reactions of **19**

##### Method A

Following the general procedure, starting from substrate **19** (72.6 mg, 0.087 mmol), after 2 h, a supplementary addition of *n-*Bu_3_SnH (24 μL, 0.087 mmol) was required. All the starting material was consumed after 3 h. Column chromatography (hexanes to hexanes–EtOAc, 2:8) gave 2,7-anhydro-3-deoxy-1-*O*-diphenoxyfosforyl-4,5-di-*O*-methyl-β-d-*xylo*-hept-2-ulopyranose (**53**) (11.5 mg, 0.026 mmol, 30%) and 1,5-anhydro-4,6-bis-*O*-diphenoxyphosphoryl-2,3-di-*O*-methyl-d-glucitol (**54**) (11 mg, 0.017 mmol, 19%) as colorless oils. Compound **53**: [α]_D_ = +2.6 (*c* = 0.46, CHCl_3_). ^1^H NMR (400 MHz, CDCl_3_, simulated ring coupling constants using DAISY) δ_H_ 7.36–7.17 (m, 10H, Ar), 4.54 (ddd, *J* = 5.1, 3.9, 0.0 Hz, 1H, 1-H), 4.29 (d, ^3^*J*_PH_ = 8.2 Hz, 2H, 6-H_2_), 4.02 (dd, *J* = 7.7, 0.0 Hz, 1H, 1’-H_b_), 3.65 (ddd, *J* = 7.5, 5.1 Hz, ^4^*J*_2,1_’_a_ = 1.1 Hz, 1H, 1’-H_a_), 3.528 (ddd, *J* = 10.0, 7.8, 6.5 Hz, 1H, 3-H), 3.47 (s, 3H, OMe), 3.36 (ddd, *J* = 7.8, 3.9 Hz, ^4^*J*_2,1_’_a_ = 1.1 Hz, 1H, 2-H), 3.36 (s, 3H, OMe), 2.29 (dd, *J* = 12.9, 6.5 Hz, 1H, 4-H_b_), 1.52 ppm (dd, *J* = 12.9, 10.0 Hz, 1H, 4-H_a_). ^13^C{^1^H} NMR (125.7 MHz, CDCl_3_) δ_C_ 150.5 (d, ^2^*J*_PC_ = 6.3 Hz, 2 × C, Ar), 120.1–129.8 (10 × CH, Ar), 105.65 (d, ^3^*J*_PC_ = 7.4 Hz, C, C-5), 80.4 (CH, C-2), 77.31 (CH, C-3), 73.9 (CH, C-1), 69.3 (d, ^2^*J*_PC_ = 5.3 Hz, CH_2_, C-6), 66.2 (CH_2_, C-1’), 58.5 (CH_3_, OMe), 57.1 (CH_3_, OMe), 36.76 ppm (CH_2_, C-4). IR (CHCl_3_): ν = 2929, 1490, 1232 cm^–1^. MS (ESI) *m*/*z* (%) = 459 (100) [M + Na]^+^. HRMS (ESI) *m*/*z:* [M + Na]^+^ calcd for C_21_H_25_NaO_8_P 459.1185; found 459.1175. Compound **54**: [α]_D_ = +27.0 (*c* = 0.70, CHCl_3_). ^1^H NMR (500 MHz, CDCl_3_, simulated ring coupling constants using DAISY) δ_H_ 7.33–7.10 (m, 20H, Ar), 4.42 (ddd, *J* = 11.6, 2.0 Hz, ^3^*J*_PH_ = 8.2 Hz, 1H, 6-H_b_), 4.36 (ddd, *J* = 9.7, 9.1 Hz, ^3^*J*_PH_ = 9.4 Hz, 1H, 4-H), 4.13 (ddd, *J* = 11.6, 5.9 Hz, ^3^*J*_PH_ = 9.8 Hz, 1H, 6-H_a_), 3.950 (dd, *J* = 11.3, 5.2 Hz, 1H, 1-H_b_), 3.51 (ddd, *J* = 9.7, 5.9, 2.0 Hz, 1H, 5-H), 3.45 (s, 3H, OMe), 3.43 (s, 3H, OMe), 3.29 (dd, *J* = 9.1, 8.8 Hz, 1H, 3-H), 3.207 (ddd, *J* = 10.6, 8.8, 5.2 Hz, 1H, 2-H), 3.040 ppm (dd, *J* = 11.3, 10.6 Hz, 1H, 1-H_a_). ^13^C{^1^H} NMR (125.7 MHz, CDCl_3_) δ_C_ 150.7 (d, ^2^*J*_PC_ = 7.4 Hz, C, Ar), 150.6 (d, ^2^*J*_PC_ = 7.4 Hz, C, Ar), 150.5 (d, ^2^*J*_PC_ = 7.4 Hz, C, Ar), 150.4 (d, ^2^*J*_PC_ = 7.4 Hz, C, Ar), 120.0–129.8 (20 × CH, Ar), 84.9 (CH, C-3), 79.82 (CH, C-2), 76.7 (CH, C-5), 75.8 (d, ^2^*J*_PC_ = 6.3 Hz, CH, C-4), 67.5 (d, ^2^*J*_PC_ = 6.4 Hz, CH_2_, C-6), 67.19 (CH_2_, C-1), 60.5 (CH_3_, OMe), 58.7 ppm (CH_3_, OMe). IR (CHCl_3_): ν = 3020, 2929, 1490, 1218 cm^–1^. MS (ESI) *m*/*z* (%) = 679 (100) [M + Na]^+^. HRMS (ESI) *m*/*z:* [M + Na]^+^ calcd for C_32_H_34_NaO_11_P_2_ 679.1474; found 679.1474.

#### Radical Reactions of **20**

##### Method A

Following the general procedure, starting from substrate **20** (89 mg, 0.14 mmol), after 2 h of reaction, a supplementary addition of *n-*Bu_3_SnH (37 μL, 0.14 mmol) was required. All the starting material was consumed after 5 h. Column chromatography on a silica gel without KF (hexanes to hexanes–EtOAc, 75:25) gave an inseparable mixture of *C*-(4-*O*-acetyl-6-*O*-*tert*-butyldiphenylsilyl-2,3-di-*O*-methyl-β-l-altropyranosyl)methanol (**50**) and **51** (33 mg, 0.066 mmol, 47%, 2:1) as a colorless oil, and **46** (12.1 mg, 0.027 mmol, 20%). Compounds **50** and **51**: ^1^H NMR (500 MHz, CDCl_3_, only **50** is described) δ_H_ 7.73–7.63 (m, 4H, Ar), 7.46–7.35 (m, 6H, Ar), 5.144 (dd, *J* = 10.1, 2.9 Hz, 1H, 4-H), 3.93–3.77 (m, 7H, 1-H, 3-H, 5-H, 6-H_2_, 1’-H_2_), 3.46 (s, 3H, OMe), 3.45 (s, 3H, OMe), 3.37 (dd, *J* = 3.8, 1.0 Hz, 1H, 2-H), 2.03 (s, 3H, OAc), 1.05 ppm (s, 9H, *^t^*Bu), 1H from OH is missing. ^13^C{^1^H} NMR (100.6 MHz, CDCl_3_, only **50** is described) δ_C_ 169.9 (C, OAc), 135.8 (2 × CH, Ar), 135.6 (2 × CH, Ar), 133.9 (C, Ar), 133.6 (C, Ar), 129.5 (2 × CH, Ar), 127.6 (2 × CH, Ar), 127.5 (2 × CH, Ar), 77.5 (CH, C-2), 74.7 (CH, C-1 or C-5), 74.4 (CH, C-1 or C-5), 74.4 (CH, C-3), 68.55 (CH, C-4), 63.88 (CH_2_, C-6), 62.8 (CH_2_, C-1’), 59.2 (CH_3_, OMe), 58.2 (CH_3_, OMe), 26.7 (3 × CH_3_, DPS), 20.9 (CH_3_, OAc), 19.3 ppm (C, DPS). IR (CHCl_3_): ν = 3690, 3567, 2933, 1737, 1217 cm^–1^. MS (ESI) *m*/*z* (%) = 525 (100) [M + Na]^+^. HRMS (ESI) *m*/*z:* [M + Na]^+^ calcd for C_27_H_38_NaO_7_Si 525.2285; found 525.2267. Anal. calcd for C_27_H_38_O_7_Si: C, 64.51; H, 7.62. Found: C, 64.58; H. 7.84.

##### Method D

Following the general procedure, starting from substrate **20** (38 mg, 0.06 mmol), after 2 h of reaction and again after 4 h, a supplementary addition of *n-*Bu_3_SnD (16 μL, 0.06 mmol) was required. All the starting material was consumed after 9 h. Column chromatography on a silica gel without KF (hexanes to hexanes–EtOAc, 8:2) gave an inseparable mixture of three compounds, (5-^2^H)**50** and **51** (19.3 mg, 0.04 mmol, 66%, 2.3:1) and olefin **46** (3.3 mg, 0.007 mmol, 13%), as a colorless oil. Mixture of (5-^2^H)**50/51/46**: ^1^H NMR (400 MHz, CDCl_3_, only (5-^2^H)**50** is described) δ_H_ 7.73–7.60 (m, 4H, Ar), 7.44–7.32 (m, 6H, Ar), 5.137 (d, *J* = 3.1 Hz, 1H, 4-H), 3.94–3.76 (m, 6H, 1-H, 3-H, 6-H_2_, 1’-H_2_), 3.45 (s, 6H, 2 × OMe), 3.37 (dd, *J* = 3.8, 1.0 Hz, 1H, 2-H), 2.03 (s, 3H, OAc), 1.05 ppm (s, 9H, *^t^*Bu), 1H from OH is missing. ^13^C{^1^H} NMR (100.6 MHz, CDCl_3_, only (5-^2^H)**50** is described) δ_C_ 169.9 (C, OAc), 135.8 (4 × CH, Ar), 133.4 (C, Ar), 133.3 (C, Ar), 129.5 (2 × CH, Ar), 127.5 (4 × CH, Ar), 77.6 (CH, C-2), 74.7 (CH, C-1), 74.5 (CH, C-3), 68.53 (CH, C-4), 63.84 (CH_2_, C-6), 62.7 (CH_2_, C-1’), 59.3 (CH_3_, OMe), 58.2 (CH_3_, OMe), 26.7 (3 × CH_3_, DPS), 20.9 (CH_3_, OAc), 19.3 ppm (C, DPS). MS (ESI) *m*/*z* (%) = 526 (100) [M + Na]^+^, 525 (48) [M + Na]^+^, 465 (54) [M + Na]^+^. HRMS (ESI) *m*/*z:* [M + Na]^+^ calcd for C_27_H_37_^2^HNaO_7_Si 526.2358; found 526.2358, [M + Na]^+^ calcd for C_27_H_38_NaO_7_Si 525.2285; found 525.2282, [M + Na]^+^ calcd for C_25_H_34_NaO_5_Si 465.2073; found 465.2089.

##### Method F

Following the general procedure, starting from substrate **20** (57 mg, 0.088 mmol), all the starting material was consumed after 2 h. Chromatotron chromatography (hexanes–EtOAc, 8:2 to 7:3) gave **45** (4.67 mg, 0.011 mmol, 12%) and an inseparable mixture of **50** and **51** (22.1 mg, 0.044 mmol, 50%, 1.2:1).

##### Method G

Following the general procedure, starting from substrate **20** (43 mg, 0.066 mmol), all the starting material was consumed after 3 h. Chromatotron chromatography (hexanes–EtOAc, 8:2 to 7:3) gave **45** (6.1 mg, 0.014 mmol, 21%) and an inseparable mixture of **50** and **51** (14.6 mg, 0.029 mmol, 44%, 1.3:1).

#### Radical Reactions of **21**

##### Method A

Following the general procedure, starting from substrate **21** (75.7 mg, 0.09 mmol), all the starting material was consumed after 2 h. Column chromatography on a silica gel without KF (hexanes to hexanes–EtOAc, 7:3) gave olefin **46** (18.1 mg, 0.041 mmol, 45%) and alcohol **52** (13.8 mg, 0.020 mmol, 22%).

##### Method C

Following the general procedure, starting from substrate **21** (73 mg, 0.087 mmol), after 2 h of reaction, a supplementary addition of TTMSS (27 μL, 0.087 mmol) was required. All the starting material was consumed after 4 h. Column chromatography (hexanes–EtOAc, 8:2) gave **45** (9.6 mg, 0.022 mmol, 25%) and **52** (14.5 mg, 0.021 mmol, 24%).

##### Method D

Following the general procedure, starting from substrate **21** (74.3 mg, 0.089 mmol), all the starting material was consumed after 2 h. Column chromatography on a silica gel without KF (hexanes to hexanes–EtOAc, 1:1) gave *C*-(6-*O*-*tert*-butyldiphenylsilyl-4-*O*-diphenoxyphosphoryl-2,3-di-*O*-methyl-α-d-[2-OMe-^2^H]galactopyranosyl)methanol ([OCH_2_-^2^H]**52**) (14.2 mg, 0.020 mmol, 23%, ^2^H/^1^H 1.3:1) and olefin **46** (17.3 mg, 0.039 mmol, 44%), both as colorless oils. Compound [OCH_2_-^2^H]**52**: ^1^H NMR (500 MHz, CDCl_3_, only the deuterated product is described) δ_H_ 7.65–7.05 (m, 20H, Ar), 5.05 (ddd, *J* = 2.8, 2.2 Hz, ^3^*J*_PH_ = 8.8 Hz, 1H, 4-H), 4.09 (ddd, *J* = 7.3, 5.7, 5.7 Hz, 1H, 1-H), 3.80–3.70 (m, 5H, 5-H, 6-H_2_, 1’-H_2_), 3.57 (dd, *J* = 8.5, 5.7 Hz, 1H, 2-H), 3.38 (m, 1H, 3-H), 3.372 (t, *J* = 1.6 Hz, 2H, OCH_2_D), 3.35 (s, 3H, OMe), 1.03 ppm (s, 9H, *^t^*Bu), 1H from OH is missing. ^13^C{^1^H} NMR (125.7 MHz, CDCl_3_, only the deuterated product is described) δ_C_ 150.8 (d, ^2^*J*_PC_ = 7.4 Hz, C, Ar), 150.4 (d, ^2^*J*_PC_ = 7.4 Hz, C, Ar), 135.6 (2 × CH, Ar), 135.5 (2 × CH, Ar), 133.3 (C, Ar), 133.2 (C, Ar), 129.8 (CH, Ar), 129.72 (CH, Ar), 129.65 (2 × CH, Ar), 129.4 (2 × CH, Ar), 127.7 (4 × CH, Ar), 125.2 (CH, Ar), 125.1 (CH, Ar), 120.34 (CH, Ar), 120.30 (CH, Ar), 120.02 (CH, Ar), 119.98 (CH, Ar), 78.7 (CH, C-3), 77.0 (CH, C-2), 74.2 (d, ^2^*J*_PC_ = 6.3 Hz, CH, C-4), 72.9 (d, ^3^*J*_PC_ = 5.3 Hz, CH, C-5), 72.6 (CH, C-1), 62.2 (CH_2_, C-6), 59.3 (CH_2_, C-1’), 59.26 (CH_2_D), 57.7 (CH_3_, OMe), 26.7 (3 × CH_3_, DPS), 19.1 ppm (C, DPS). MS (ESI) *m*/*z* (%) = 716 (100) [M + Na]^+^, 715 (67) [M + Na]^+^. HRMS (ESI) *m*/*z:* [M + Na]^+^ calcd for C_37_H_44_^2^HNaO_9_PSi 716.2531; found 716.2554, *M* + Na]^+^ calcd for C_37_H_45_NaO_9_PSi 715.2468; found 715.2471.

##### Method E

Following the general procedure, starting from substrate **21** (81.8 mg, 0.098 mmol), after 2 h, a supplementary addition of *n-*Bu_3_SnD (26 μL, 0.098 mmol) and BF_3_•Et_2_O (3 μL, 0.024 mmol) was required. All the starting material was consumed after 3 h. Column chromatography on a silica gel without KF (hexanes to hexanes–EtOAc, 1:1) gave [4-^2^H]**45** (17.8 mg, 0.040 mmol, 41%, ^2^H/^1^H 2.8:1, 4*R*/4*S* 1:1.2) and [OCH_2_-^2^H]**52** (8.6 mg, 0.012 mmol, 13%, ^2^H/^1^H 1.1:1).

##### Method F

Following the general procedure, starting from substrate **21** (36.9 mg, 0.044 mmol), all the starting material was consumed after 3 h. Chromatotron chromatography (hexanes–EtOAc, 8:2 to 4:6) gave **52** (15.8 mg, 0.023 mmol, 52%).

##### Method G

Following the general procedure, starting from substrate **21** (40.5 mg, 0.048 mmol), all the starting material was consumed after 3 h. Chromatotron chromatography (hexanes–EtOAc, 8:2 to 4:6) gave **52** (13.5 mg, 0.019 mmol, 40%).

#### Radical Reactions of **22**

##### Method A

Following the general procedure, starting from substrate **22** (106.8 mg, 0.18 mmol), after 2 h, a supplementary addition of *n-*Bu_3_SnH (49 μL, 0.18 mmol) was required. All the starting material was consumed after 5 h. Column chromatography (hexanes to hexanes–EtOAc, 1:1) gave 2,7-anhydro-1,3-dideoxy-4,5-di-*O*-methyl-β-l-*ribo*-hept-2-ulopyranose (**61**) (18.9 mg, 0.10 mmol, 56%) as a colorless oil. [α]_D_ = +0.02 (*c* = 0.34, CHCl_3_). ^1^H NMR (500 MHz, CDCl_3_, simulated ring coupling constants using DAISY) δ_H_ 4.75 (ddd, *J* = 5.8, 2.8, 0.9 Hz, 1H, 1-H), 3.82 (dd, *J* = 7.7, 5.8 Hz, 1H, 1’-H_b_), 3.67 (dd, *J* = 7.7, 0.9 Hz, 1H, 1’-H_a_), 3.626 (ddd, *J* = 11.1, 6.0, 4.1 Hz, 1H, 3-H), 3.46 (dd, *J* = 4.1, 2.8 Hz, 1H, 2-H), 3.56 (s, 3H, OMe), 3.38 (s, 3H, OMe), 2.121 (dd, *J* = 12.5, 6.0 Hz, 1H, 4-H_b_), 1.816 (dd, *J* = 12.5, 11.1 Hz, 1H, 4-H_a_), 1.51 ppm (s, 3H, 6-H_3_). ^13^C{^1^H} NMR (125.7 MHz, CDCl_3_) δ_C_ 106.96 (C, C-5), 75.4 (CH, C-2), 74.1 (CH, C-1), 73.78 (CH, C-3), 65.8 (CH_2_, C-1’), 57.9 (CH_3_, OMe), 56.2 (CH_3_, OMe), 38.69 (CH_2_, C-4), 23.8 ppm (CH_3_, C-6). IR (CHCl_3_): ν = 3015, 2934, 1389, 1198 cm^–1^. MS (ESI) *m*/*z* (%) = 211 (100) [M + Na]^+^. HRMS (ESI) *m*/*z:* [M + Na]^+^ calcd for C_9_H_16_NaO_4_ 211.0946; found 211.0948. Anal. calcd for C_9_H_16_O_4_: C, 57.43; H, 8.57. Found: C, 57.63; H, 8.63.

##### Method C

Following the general procedure, starting from substrate **22** (58.8 mg, 0.10 mmol), after 2 h of reaction, a supplementary addition of TTMSS (31 μL, 0.10 mmol) was required. All the starting material was consumed after 4 h. Column chromatography (hexanes–EtOAc, 9:1 to 6:4) gave **61** (9 mg, 0.048 mmol, 48%).

##### Method D

Following the general procedure, starting from substrate **22** (61.5 mg, 0.105 mmol), after 2 h, a supplementary addition of *n-*Bu_3_SnD (29 μL, 0.105 mmol) was required. All the starting material was consumed after 5 h. Column chromatography (hexanes to hexanes–EtOAc, 1:1) gave 2,7-anhydro-1,3-dideoxy-4,5-di-*O*-methyl-β-l-[4-^2^H]*ribo*-hept-2-ulopyranose ([4-^2^H]**61**) (8.7 mg, 0.046 mmol, 44%, ^2^H/^1^H 1.8:1, 4*R*/4*S* 1:1.3) as a colorless oil: ^1^H NMR (500 MHz, CDCl_3_, only deuterated isomers are described) δ_H_ 4.75 (ddd, *J* = 5.7, 2.8, 0.0 Hz, 1H, 1-H), 3.82 (dd, *J* = 7.6, 5.7 Hz, 1H, 1’-H_b_), 3.67 (dd, *J* = 7.9, 0.0 Hz, 1H, 1’-H_a_), 3.63–3.60 (m, 1H, 3-H), 3.55 (s, 3H, OMe), 3.47–3.44 (m, 1H, 2-H), 3.38 (s, 3H, OMe), 2.101 (d, *J* = 5.7 Hz, 1H, 4-H, 4*S* isomer), 1.793 (d, *J* = 11.1 Hz, 1H, 4-H, 4*R* isomer), 1.51 ppm (s, 3H, 6-H_3_). ^13^C{^1^H} NMR (125.7 MHz, CDCl_3_, only deuterated isomers are described) δ_C_ 106.92 (C, C-5), 75.4 (CH, C-2), 74.1 (CH, C-1), 73.69 (CH, C-3), 65.8 (CH_2_, C-1’), 57.8 (CH_3_, OMe), 56.2 (CH_3_, OMe), 38.52 (t, *J*_CD_ = 20.1 Hz, CHD, C-4), 23.8 ppm (CH_3_, C-6). MS (ESI) *m*/*z* (%) = 212 (100) [M + Na]^+^, 211 (100) [M + Na]^+^. HRMS (ESI) *m*/*z:* [M + Na]^+^ calcd for C_9_H_15_^2^HNaO_4_ 212.1009; found 212.1005, [M + Na]^+^ calcd for C_9_H_16_NaO_4_ 211.0946; found 211.0948.

##### Method E

Following the general procedure, starting from substrate **22** (77 mg, 0.13 mmol), after 2 h, a supplementary addition of *n-*Bu_3_SnD (35 μL, 0.13 mmol) and BF_3_·Et_2_O (3.3 μL, 0.026 mmol) was required. All the starting material was consumed after 7 h. Column chromatography (hexanes to hexanes–EtOAc, 6:4) gave [4-^2^H]**61** (16.2 mg, 0.086 mmol, 66%, ^2^H/^1^H 1.3:1, 4*R*/4*S* 1:1.3).

##### Method F

Following the general procedure, starting from substrate **22** (28.9 mg, 0.05 mmol), all the starting material was consumed after 1.5 h. Chromatotron chromatography (hexanes–EtOAc, 4:6 to 3:7) gave **61** (5.2 mg, 0.028 mmol, 55%).

##### Method G

Following the general procedure, starting from substrate **22** (29.1 mg, 0.050 mmol), all the starting material was consumed after 3 h. Chromatotron chromatography (hexanes–EtOAc, 4:6 to 3:7) gave **61** (5.7 mg, 0.030 mmol, 61%).

#### Radical Reactions of **23**

##### Method A

Following the general procedure, starting from substrate **23** (88 mg, 0.22 mmol), after 2 h, a supplementary addition of *n-*Bu_3_SnH (60 μL, 0.22 mmol) was required. All the starting material was consumed after 6 h. Column chromatography (hexanes to hexanes–EtOAc, 4:6) gave *C*-(4,6-dideoxy-2,3-di-*O*-methyl-β-d-*threo*-hex-4-enopyranosyl)methanol (**56**) (8.3 mg, 0.044 mmol, 20%) and *C*-(4-*O*-acetyl-2,3-6-deoxy-di-*O*-methyl-β-d-altropyranosyl)methanol (**57**) (4.4 mg, 0.018 mmol, 8%) as colorless oils, and **58** (7.8 mg, 0.031 mmol, 14%). Compound **56**: [α]_D_ = −119.1 (*c* = 0.45, CHCl_3_). ^1^H NMR (500 MHz, CDCl_3_, simulated ring coupling constants using DAISY) δ_H_ 4.78 (br ddd, *J* = 5.3 Hz, ^4^*J* = 2.0, 0.9 Hz, 1H, 4-H), 4.01 (dd, *J* = 11.4, 6.4 Hz, 1H, 1’-H_b_), 3.94 (ddd, *J* = 6.4, 4.1, 1.5 Hz, 1H, 1-H), 3.86 (br dd, *J* = 11.4, 4.1 Hz, 1H, 1’-H_a_), 3.60 (ddd, *J* = 5.3, 2.0 Hz, ^5^*J* = 1.0 Hz, 1H, 3-H), 3.45 (s, 3H, OMe), 3.40 (ddd, *J* = 2.0, 1.5 Hz, ^4^*J* = 2.0 Hz, 1H, 2-H), 3.39 (s, 3H, OMe), 2.25 (br s, 1H, OH), 1.86 ppm (dd, ^5^*J* = 1.0 Hz, ^4^*J* = 0.9 Hz, 3H, 6-H_3_). ^13^C{^1^H} NMR (125.7 MHz, CDCl_3_) δ_C_ 154.7 (C, C-5), 93.7 (CH, C-4), 75.7 (CH, C-2), 73.8 (CH, C-1), 69.7 (CH, C-3), 63.0 (CH_2_, C-1’), 58.1 (CH_3_, OMe), 55.4 (CH_3_, OMe), 20.0 ppm (CH_3_, C-6). IR (CHCl_3_): ν = 3691, 3602, 3013, 2933, 1672, 1226 cm^–1^. MS (ESI) *m*/*z* (%) = 211 (100) [M + Na]^+^. HRMS (ESI) *m*/*z:* [M + Na]^+^ calcd for C_9_H_16_NaO_4_ 211.0946; found 211.0942. Anal. calcd for C_9_H_16_O_4_: C, 57.43; H, 8.57. Found: C, 57.10; H, 8.27. Compound **57**: [α]_D_ = +50.3 (*c* = 0.35, CHCl_3_). ^1^H NMR (500 MHz, CDCl_3_) δ_H_ 4.799 (dd, *J* = 10.1, 3.2 Hz, 1H, 4-H), 3.92–3.84 (m, 4H, 1-H, 2-H, 5-H, 1’-H_b_), 3.67 (m, 1H, 1’-H_a_), 3.464 (s, 3H, OMe), 3.462 (s, 3H, OMe), 3.40 (dd, *J* = 3.8, 1.0 Hz, 1H, 3-H), 2.12 (s, 3H, OAc), 1.201 ppm (d, *J* = 6.4 Hz, 3H, 6-H_3_), 1H from OH is missing. ^13^C{^1^H} NMR (125.7 MHz, CDCl_3_) δ_C_ 170.2 (C, OAc), 77.7 (CH, C-3), 74.7 (CH, C-2), 74.2 (CH, C-1), 73.40 (CH, C-4), 69.68 (CH, C-5), 62.7 (CH_2_, C-1’), 59.3 (CH_3_, OMe), 58.4 (CH_3_, OMe), 21.1 (CH_3_, OAc), 17.79 ppm (CH_3_, C-6). IR (CHCl_3_): ν = 3690, 3603, 3018, 2935, 1734, 1220 cm^–1^. MS (ESI) *m*/*z* (%) = 271 (100) [M + Na]^+^. HRMS (ESI) *m*/*z:* [M + Na]^+^ calcd for C_11_H_20_NaO_6_ 271.1158; found 271.1167. Anal. calcd for C_11_H_20_O_6_: C, 53.21; H, 8.12. Found: C, 52.92; H, 7.97.

##### Method D

Following the general procedure, starting from substrate **23** (62 mg, 0.16 mmol), after 2 h, a supplementary addition of *n-*Bu_3_SnD (43 μL, 0.16 mmol) was required. All the starting material was consumed after 4 h. Column chromatography (hexanes to hexanes–EtOAc, 3:7) gave **56** (9.4 mg, 0.05 mmol, 31%), *C*-(4-*O*-acetyl-2,3-6-deoxy-di-*O*-methyl-β-d-(5-^2^H)altropyranosyl)methanol [(5-^2^H)**57**] (7.6 mg, 0.031 mmol, 19%), and *C*-(4-*O*-acetyl-2,3-di-*O*-methyl-α-d-[5-^2^H]fucopyranosyl)methanol (5-^2^H]**58**) (8.4 mg, 0.034 mmol, 21%, ^2^H/^1^H 2.4:1) as colorless oils. Compound (5-^2^H)**57**: ^1^H NMR (500 MHz, CDCl_3_) δ_H_ 4.796 (d, *J* = 2.9 Hz, 1H, 4-H), 3.92–3.84 (m, 3H, 1-H, 2-H, 1’-H_b_), 3.67 (m, 1H, 1’-H_a_), 3.463 (s, 3H, OMe), 3.461 (s, 3H, OMe), 3.40 (dd, *J* = 3.5, 1.0 Hz, 1H, 3-H), 2.12 (s, 3H, OAc), 1.192 ppm (s, 3H, 6-H_3_), 1H from OH is missing. ^13^C{^1^H} NMR (125.7 MHz, CDCl_3_) δ_C_ 170.2 (C, OAc), 77.7 (CH, C-3), 74.7 (CH, C-2), 74.2 (CH, C-1), 73.33 (CH, C-4), 69.26 (t, *J*_CD_ = 21.2 Hz, C, C-5), 62.7 (CH_2_, C-1’), 59.3 (CH_3_, OMe), 58.4 (CH_3_, OMe), 21.1 (CH_3_, OAc), 17.66 ppm (CH_3_, C-6). MS (ESI) *m*/*z* (%) = 272 (100) [M + Na]^+^. HRMS (ESI) *m*/*z:* [M + Na]^+^ calcd for C_11_H_19_^2^HNaO_6_ 272.1220; found 272.1219. Compound [5-^2^H]**58**: ^1^H NMR (500 MHz, CDCl_3_, only the deuterated product is described) δ_H_ 5.323 (d, *J* = 3.2 Hz, 1H, 4-H), 4.23 (m, 1H, 1-H), 3.91–3.83 (m, 2H, 1’-H_2_), 3.70 (dd, *J* = 9.1, 6.0 Hz, 1H, 2-H), 3.50 (dd, *J* = 9.1, 3.5 Hz, 1H, 3-H), 3.50 (s, 3H, OMe), 3.42 (s, 3H, OMe), 2.17 (s, 3H, OAc), 1.169 ppm (s, 3H, 6-H_3_), 1H from OH is missing. ^13^C{^1^H} NMR (125.7 MHz, CDCl_3_, only the deuterated product is described) δ_C_ 170.7 (C, OAc). 78.8 (CH, C-3), 77.1 (CH, C-2), 73.4 (CH, C-1), 69.46 (CH, C-4), 59.7 (CH_2_, C-1’), 59.4 (CH_3_, OMe), 57.5 (CH_3_, OMe), 20.8 (CH_3_, OAc), 16.37 ppm (CH_3_, C-6). MS (ESI) *m*/*z* (%) = 272 (71) [M + Na]^+^, 271 (28) [M + Na]^+^. HRMS (ESI) *m*/*z:* [M + Na]^+^ calcd for C_11_H_19_^2^HNaO_6_ 272.1220; found 272.1222, [M + Na]^+^ calcd for C_11_H_20_NaO_6_ 271.1158; found 271.1164.

##### Method E

Following the general procedure, starting from substrate **23** (126 mg, 0.32 mmol), after 2 h, a supplementary addition of *n-*Bu_3_SnD (87 μL, 0.32 mmol) and BF_3_•Et_2_O (8 μL, 0.064 mmol) was required. All the starting material was consumed after 4 h. Column chromatography (hexanes to hexanes–EtOAc, 3:7) gave (5-^2^H)**57** (9.5 mg, 0.038 mmol, 12%), [5-^2^H]**58** (11.4 mg, 0.046 mmol, 14%, ^2^H/^1^H 1:2), the unstable and volatile 2,7-anhydro-1,3-dideoxy-4,5-di-*O*-methyl-β-l-[3-^2^H]*xylo*-hept-2-ulopyranose ([4-^2^H]**55**) (13 mg, 0.069 mmol, 21%, ^2^H/^1^H 5.4:1), and 3-*O*-acetyl-2,6-anhydro-1-deoxy-4,5-di-*O*-methyl-d-(6-^2^H)galactitol [(1-^2^H)**60**] (2 mg, 0.009 mmol, 3%) as colorless oils. Compound [4-^2^H]**55**: ^1^H NMR (500 MHz, CDCl_3_) δ_H_ 4.51 (ddd, *J* = 4.7, 4.7, 0.0 Hz, 1H, 1-H), 3.99 (dd, *J* = 7.6, 0.0 Hz, 1H, 1’-H_b_), 3.74 (dd, *J* = 7.6, 5.4 Hz, 1H, 1’-H_a_), 3.53–3.48 (m, 1H, 3-H), 3.48 (s, 3H, OMe), 3.40 (s, 3H, OMe), 3.35 (dd, *J* = 8.2, 4.1 Hz, 1H, 2-H), 2.33 (dd, *J* = 13.2, 6.7 Hz, 1H, 4-H_b_), 2.32 (d, *J* = 6.6 Hz, 1H, 4-HD), 1.44 (m, 1H, 4-H_a_), 1.49 ppm (s, 3H, 6-H_3_). ^13^C{^1^H} NMR (125.7 MHz, CDCl_3_) δ_C_ 107.0 (C, C-5), 80.7 (CH, C-2), 77.3 (CH, C-3), 73.5 (CH, C-1), 65.8 (CH_2_, C-1’), 58.3 (CH_3_, OMe), 57.1 (CH_3_, OMe), 36.5 (CH_2_, C-4 reduced product), 23.5 ppm (CH_3_, C-6), expected triplet for C-4 was imperceptible for the deuterated product. IR (CHCl_3_): ν = 3022, 2929, 1226 cm^–1^. MS (ESI) *m*/*z* (%) = 212 (100) [M + Na]^+^, 211 (18) [M + Na]^+^. HRMS (ESI) *m*/*z:* [M + Na]^+^ calcd for C_9_H_15_^2^HNaO_4_ 212.1009; found 212.1015, [M + Na]^+^ calcd for C_9_H_16_NaO_4_ 211.0946; found 211.0950. Compound (1-^2^H)**60**: [α]_D_ = −11.1 (*c* = 0.45, CHCl_3_). ^1^H NMR (500 MHz, CDCl_3_, simulated ring coupling constants using DAISY) δ_H_ 5.34 (dd, *J* = 3.4, 1.31 Hz, 1H, 4-H), 4.10 (br d, *J* = 4.4 Hz, 1H, 1-H), 3.58 (dddd, *J* = 6.6, 6.6, 6.6, 1.1 Hz, 1H, 5-H), 3.52 (m, 1H, 2-H), 3.50 (s, 3H, OMe), 3.43 (s, 3H, OMe), 3.24 (dd, *J* = 9.3, 3.4 Hz, 1H, 3-H), 2.18 (s, 3H, OAc), 1.17 ppm (d, *J* = 6.6 Hz, 3H, 6-H_3_). ^13^C{^1^H} NMR (125.7 MHz, CDCl_3_) δ_C_ 170.8 (C, OAc), 82.8 (CH, C-3), 75.4 (CH, C-2), 73.5 (CH, C-5), 69.4 (CH, C-4), 59.0 (CH_3_, OMe), 57.4 (CH_3_, OMe), 20.8 (CH_3_, OAc), 16.8 ppm (CH_3_, C-6), C-1 was imperceptible. IR (CHCl_3_): ν = 3016, 2932, 1226 cm^–1^. MS (ESI) *m*/*z* (%) = 242 (100) [M + Na]^+^. HRMS (ESI) *m*/*z:* [M + Na]^+^ calcd for C_10_H_17_^2^HNaO_5_ 242.1115; found 242.1111.

##### Method F

Following the general procedure, starting from substrate **23** (13.9 mg, 0.035 mmol), all the starting material was consumed after 3 h. Chromatotron chromatography (hexanes–EtOAc, 4:6 to 0:1) gave **57** (1 mg, 0.004 mmol, 11%), **58** (2.2 mg, 0.09 mmol, 25%), and product **55** (0.7 mg, 0.004 mmol, 11%).

##### Method G

Following the general procedure, starting from substrate **23** (13.7 mg, 0.035 mmol), all the starting material was consumed after 3 h. Chromatotron chromatography (hexanes–EtOAc, 4:6 to 0:1) gave **57** (1.3 mg, 0.030 mmol, 15%), **58** (1.3 mg, 0.005 mmol, 15%), and product **55** (1.5 mg, 0.008 mmol, 23%).

#### Radical Reactions of **24**

##### Method A

Following the general procedure, starting from substrate **24** (80.5 mg, 0.18 mmol), after 2 h, a supplementary addition of *n-*Bu_3_SnH (50 μL, 0.18 mmol) was required. All the starting material was consumed after 3 h. Column chromatography (hexanes to hexanes–EtOAc, 4:6) gave **56** (23.6 mg, 0.126 mmol, 70%) and **59** (10.2 mg, 0.023 mmol, 13%).

##### Method C

Following the general procedure, starting from substrate **24** (62 mg, 0.11 mmol), after 2 h of reaction, a supplementary addition of TTMSS (33 μL, 0.11 mmol) was required. All the starting material was consumed after 4 h. Column chromatography (hexanes–EtOAc, 6:4 to 3:7) gave **56** (12 mg, 0.064 mmol, 58%) and **59** (3.7 mg, 0.008 mmol, 8%).

##### Method D

Following the general procedure, starting from substrate **24** (50 mg, 0.086 mmol), after 2 h, a supplementary addition of *n-*Bu_3_SnD (23 μL, 0.086 mmol) was required. All the starting material was consumed after 4 h. Column chromatography (hexanes to hexanes–EtOAc, 3:7) gave **56** (12 mg, 0.064 mmol, 58%) and **59** (7.2 mg, 0.016 mmol, 19%).

##### Method E

Following the general procedure, starting from substrate **24** (37.7 mg, 0.066 mmol), after 2 h, a supplementary addition of *n-*Bu_3_SnD (17 μL, 0.066 mmol) and BF_3_•Et_2_O (2 μL, 0.016 mmol) was required. All the starting material was consumed after 4 h. Column chromatography (hexanes to hexanes–EtOAc, 3:7) gave **59** (4.2 mg, 0.009 mmol, 15%) and [4-^2^H]**55** (5.1 mg, 0.027 mmol, 41%, ^2^H/^1^H 3.1:1).

##### Method F

Following the general procedure, starting from substrate **24** (53 mg, 0.091 mmol), all the starting material was consumed after 2 h. Chromatotron chromatography (hexanes–EtOAc, 3:7) gave **59** (19.7 mg, 0.045 mmol, 49%).

##### Method G

Following the general procedure, starting from substrate **24** (61 mg, 0.105 mmol), all the starting material was consumed after 3 h. Chromatotron chromatography (hexanes–EtOAc, 3:7) gave **59** (22.9 mg, 0.052 mmol, 50%).

### 3-*C*-(3,4-Di-*O*-benzyl-α-l-fucopyranosyl)1-propene (**75**)

3-*C*-(2,3,4-Tri-*O*-benzyl-α-l-fucopyranosyl)1-propene (**74**)^61^ (1.56 g, 3.41 mmol) was dissolved in dry CH_2_Cl_2_ (68 mL) under a N_2_ atmosphere, and I_2_ (8.6 g, 34.1 mmol) was added. The mixture was stirred at room temperature for 3 h, and then it was poured over an aqueous solution of Na_2_S_2_O_3_ and extracted with CH_2_Cl_2_. The combined extracts were dried over Na_2_SO_4_ and concentrated under reduced pressure. The resulting crude was dissolved in Et_2_O/MeOH (1:1) (35 mL), and Zn dust (2.04 g, 31.2 mmol) and AcOH (357 μL) were subsequently added, with the mixture stirred overnight at room temperature. Then, it was filtered over Celite, evaporated, poured over a saturated aqueous solution of NaHCO_3_, and extracted with CH_2_Cl_2_. The organic layers were dried over Na_2_SO_4_ and concentrated under reduced pressure. Column chromatography of the residue (hexanes–EtOAc, 8:2) gave **75** (791.8 mg, 2.15 mmol, 63%) as an amorphous solid: [α]_D_ = −57.1 (*c* = 0.42, CHCl_3_). ^1^H NMR (500 MHz, CDCl_3_) δ_H_ 7.35–7.24 (m, 10H, Ar), 5.81 (dddd, *J* = 17.1, 10.1, 6.7, 6.7 Hz, 1H, 2’-H), 5.10 (dd, *J* = 17.1, 1.0 Hz, 1H, 3’-H_b_), 5.05 (dd, *J* = 10.1, 0.0 Hz, 1H, 3’-H_a_), 4.78 (d, *J* = 12.0 Hz, 1H, OBn), 4.73 (d, *J* = 12.0 Hz, 1H, OBn), 4.59 (d, *J* = 12.0 Hz, 1H, OBn), 4.58 (d, *J* = 11.9 Hz, 1H, OBn), 4.11 (m, 1H, 1-H), 4.04 (br s, 1H, 2-H), 3.94 (m, 1H, 5-H), 3.77 (dd, *J* = 3.2, 3.2 Hz, 1H, 4-H), 3.73 (dd, *J* = 7.0, 2.9 Hz, 1H, 3-H), 2.33 (dd, *J* = 7.6, 7.6 Hz, 2H, 1’-H_2_), 2.18 (br s, 1H, OH), 1.31 ppm (d, *J* = 6.6 Hz, 3H, 6-H_3_). ^13^C{^1^H} NMR (100.6 MHz, CDCl_3_) δ_C_ 138.4 (C, Ar), 138.3 (C, Ar), 135.0 (CH, C-2’), 128.4 (2 × CH, Ar), 128.3 (2 × CH, Ar), 127.7 (3 × CH, Ar), 127.6 (3 × CH, Ar), 116.7 (CH_2_, C-3’), 78.8 (CH, C-3), 75.0 (CH, C-4), 73.0 (CH_2_, OBn), 72.6 (CH_2_, OBn), 70.8 (CH, C-1), 69.1 (CH, C-5), 68.9 (CH, C-2), 31.9 (CH_2_, C-1’), 15.4 ppm (CH_3_, C-6). IR (CHCl_3_): ν = 3580, 3021, 1210 cm^–1^. MS (ESI) *m*/*z* (%) = 391 (100) [M + Na]^+^. HRMS (ESI) *m*/*z*: [M + Na]^+^ calcd for C_23_H_28_NaO_4_ 391.1885; found 391.1889.

### 3-*C*-(3,4-Di-*O*-benzylidene-α,β-d-arabinopyranosyl)1-propene (**79** and **80**)

Tetra-*O*-acetyl-d-arabinopyranose (**78**)^62^ (3.35 g, 10.54 mmol) was dissolved in dry CH_3_CN (129 mL) under a N_2_ atmosphere, and allyltrimethylsilane (9.8 mL, 61.51 mmol) and BF_3_•Et_2_O (6.2 mL, 49.2 mmol) were dropwise added at 0 °C. Then, the mixture was stirred at room temperature for 1.5 h. Subsequently, the solution was poured over a saturated aqueous solution of NaCl, extracted with EtOAc, dried over Na_2_SO_4_, and evaporated. Column chromatography of the residue (hexanes–EtOAc, 7:3) gave the allyl derivative (2.18 g, 7.27 mmol, 69%, 1*S*/1*R* isomers 6.8:1) as a colorless oil, which was subsequently dissolved in dry MeOH (34 mL), and Na_2_CO_3_ (1.23 g, 11.60 mmol) was added. The mixture was stirred at room temperature for 2.5 h, and then it was filtered, neutralized with the Amberlyst 15 H^+^ ion exchange resin, and evaporated. The crude was submitted to the benzylidene protection by treatment overnight with PhCH(OMe)_2_ (1.5 mL, 10.91 mmol) and CSA (17 mg, 0.07 mmol) in dry DMF (7.3 mL) at room temperature under a N_2_ atmosphere. The reaction was evaporated in a high vacuum rotovap and purified by column chromatography (hexanes–EtOAc, 8:2) to give **79** (228 mg, 0.87 mmol, 12%, d.r., 3:1) and **80** (780.8 g, 2.98 mmol, 41%, d.r., 3.5:1) as colorless oils. Compound **79**: ^1^H NMR (500 MHz, CDCl_3_, only the major isomer is described) δ_H_ 7.48–7.34 (m, 5H, Ar), 6.23 (s, 1H, PhC*H*), 5.86 (dddd, *J* = 17.0, 10.1, 7.0, 7.0 Hz, 1H, 2’-H), 5.18 (dd, *J* = 17.0, 1.6 Hz, 1H, 3’-H_b_), 5.13 (dd, *J* = 10.1, 1.6 Hz, 1H, 3’-H_a_), 4.57 (ddd, *J* = 9.2, 6.7, 5.1 Hz, 1H, 4-H), 4.30 (dd, *J* = 5.1, 2.9 Hz, 1H, 3-H), 4.10 (dd, *J* = 12.0, 6.7 Hz, 1H, 5-H_b_), 3.97 (br d, *J* = 5.4 Hz, 1H, 2-H), 3.73 (ddd, *J* = 7.9, 6.7, 1.6 Hz, 1H, 1-H), 3.55 (dd, *J* = 12.0, 9.1 Hz, 1H, 5-H_a_), 2.48 (m, 1H, 1’-H_b_), 2.36 ppm (m, 1H, 1’-H_a_), 1H from OH is missing. ^13^C{^1^H} NMR (125.7 MHz, CDCl_3_, only the major isomer is described) δ_C_ 139.0 (C, Ar), 134.0 (CH, C-2’), 129.1 (CH, Ar), 128.4 (2 × CH, Ar), 125.9 (2 × CH, Ar), 117.6 (CH_2_, C-3’), 103.3 (CH, Ph*C*H), 75.7 (CH, C-3), 75.0 (CH, C-1), 70.1 (CH, C-4), 67.5 (CH, C-2), 60.0 (CH_2_, C-5), 34.6 ppm (CH_2_, C-1’). IR (CHCl_3_): ν = 3567, 3452, 1643, 1457, 1100 cm^–1^. MS (ESI) *m*/*z* (%) = 285 (100) [M + Na]^+^. HRMS (ESI) *m*/*z*: [M + Na]^+^ calcd for C_15_H_18_NaO_4_ 285.1103; found 285.1099. Compound **80**: ^1^H NMR (500 MHz, CDCl_3_, only the major isomer is described) δ_H_ 7.52–7.39 (m, 5H, Ar), 5.95 (s, 1H, PhC*H*), 5.84 (dddd, *J* = 17.4, 10.1, 7.3, 7.3 Hz, 1H, 2’-H), 5.18 (br d, *J* = 17.2 Hz, 1H, 3’-H_b_), 5.11 (br d, *J* = 10.1 Hz, 1H, 3’-H_a_), 4.43 (m, 1H, 4-H), 4.36 (dd, *J* = 5.7, 2.9 Hz, 1H, 3-H), 4.08 (dd, *J* = 12.0, 6.3 Hz, 1H, 5-H_b_), 4.00 (dd, *J* = 2.5, 1.9 Hz, 1H, 2-H), 3.73 (ddd, *J* = 8.2, 6.6, 1.9 Hz, 1H, 1-H), 3.50 (dd, *J* = 12.0, 8.5 Hz, 1H, 5-H_a_), 2.45 (m, 1H, 1’-H_b_), 2.34 ppm (m, 1H, 1’-H_a_), 1H from OH is missing. ^13^C{^1^H} NMR (125.7 MHz, CDCl_3_, only the major isomer is described) δ_C_ 137.1 (C, Ar), 134.0 (CH, C-2’), 129.1 (CH, Ar), 128.4 (2 × CH, Ar), 126.4 (2 × CH, Ar), 117.6 (CH_2_, C-3’), 104.3 (CH, Ph*C*H), 77.7 (CH, C-3), 75.0 (CH, C-1), 69.2 (CH, C-4), 68.0 (CH_2_, C-5), 67.5 (CH, C-2), 34.8 ppm (CH_2_, C-1’). IR (CHCl_3_): ν = 3567, 3422, 3023, 1643, 1459, 1068 cm^–1^. MS (ESI) *m*/*z* (%) = 285 (100) [M + Na]^+^. HRMS (ESI) *m*/*z*: [M + Na]^+^ calcd for C_15_H_18_NaO_4_ 285.1103; found 285.1097.

### 3-*C*-(3,5-Di-*O*-*tert*-butyldiphenylsilyl-α-d-ribofuranosyl)1-propene (**83**)

To a solution of 3-*C*-(α-d-ribofuranosyl)1-propene (**82**)^56,57^ (5.24 g, 30.11 mmol, 87%) in dry CH_2_Cl_2_ (145 mL) at 0 °C were sequentially added imidazole (3.07 g, 45.17 mmol) and DPSCl (7.72 mL, 30.11 mmol). The resulting mixture was stirred at 0 °C for 3 h, treated with saturated aqueous NH_4_Cl, and extracted with CH_2_Cl_2_. Purification by column chromatography (hexanes–EtOAc, 97:3 to 6:4) afforded monoalcohol **83** (7.11 g, 10.94 mmol, 36%) and known diol 3-*C*-(5-*O*-*tert*-butyldiphenylsilyl-α-d-ribofuranosyl)1-propene (**84**)^13,57^ (4.92 g, 11.94 mmol, 40%) as colorless oils. Compound **83**: [α]_D_ = +33.1 (*c =* 0.58, CHCl_3_). ^1^H NMR (500 MHz, CDCl_3_) δ_H_ 7.57–7.15 (m, 20H, Ar), 5.77 (dddd, *J* = 17.0, 10.1, 7.0, 7.0 Hz, 1H, 2’-H), 5.04 (br d, *J* = 17.4 Hz, 1H, 3’-H_b_), 4.97 (br d, *J* = 10.1 Hz, 1H, 3’-H_a_), 4.49 (dd, *J* = 5.4, 5.4 Hz, 1H, 3-H), 3.87 (m, 1H, 4-H), 3.84 (m, 1H, 1-H), 3.77 (dd, *J* = 4.7, 4.7 Hz, 1H, 2-H), 3.50 (dd, *J* = 11.5, 2.2 Hz, 1H, 5-H_b_), 3.14 (dd, *J* = 11.4, 3.2 Hz, 1H, 5-H_a_), 2.76 (br s, 1H, OH), 2.38 (dd, *J* = 6.9, 6.9 Hz, 2H, 1’-H_2_), 1.00 (s, 9H, *^t^*Bu), 0.80 ppm (s, 9H, *^t^*Bu). ^13^C{^1^H} NMR (125.7 MHz, CDCl_3_) δ_C_ 135.7 (2 × CH, Ar), 135.63 (2 × CH, Ar), 135.61 (CH, C-2’), 135.5 (2 × CH, Ar), 135.1 (2 × CH, Ar), 133.4 (C, Ar), 133.2 (C, Ar), 132.6 (C, Ar), 132.4 (C, Ar), 130.20 (CH, Ar), 130.16 (CH, Ar), 129.5 (2 × CH, Ar), 128.0 (2 × CH, Ar), 127.8 (2 × CH, Ar), 127.5 (4 × CH, Ar), 116.6 (CH_2_, C-3’), 83.1 (CH, C-4), 81.0 (CH, C-1), 74.5 (CH, C-3), 72.7 (CH, C-2), 64.0 (CH_2_, C-5), 34.1 (CH_2_, C-1’), 26.9 (3 × CH_3_, *^t^*Bu), 26.7 (3 × CH_3_, *^t^*Bu), 19.2 (C, *^t^*Bu), 19.0 ppm (C, *^t^*Bu). IR (CHCl_3_): ν = 3673, 3541, 2932, 2860, 1428, 1113 cm^–1^. MS (ESI) *m*/*z* (%) = 673 (100) [M + Na]^+^. HRMS (ESI) *m*/*z*: [M + Na]^+^ calcd for C_40_H_50_NaO_4_Si_2_ 673.3145; found 673.3141.

### 3-*C*-(3,5-Di-*O*-1,1,3,3-tetraisopropyldisiloxanyl-α-d-ribofuranosyl)1-propene (**86**)

1,3-Dichloro-1,1,3,3-tetraisopropyldisiloxane (1.1 mL, 3.44 mmol) was added to a stirred solution of triol **82**(56,57) (300 mg, 1.72 mmol) in dry pyridine (53 mL) at 0 °C. The reaction mixture was stirred at room temperature for 20 h, and then the pyridine was evaporated under reduced pressure. The residue was poured over 10% HCl and extracted with EtOAc. The combined organic extracts were washed with a saturated solution of NaHCO_3_, dried over Na_2_SO_4_, filtered, and evaporated. The crude was subjected to chromatography (hexanes–EtOAc, 95:5) to afford monoalcohol **86** (496.4 mg, 1.19 mmol, 69%) as a colorless oil: [α]_D_ = −17.6 (*c =* 0.51, CHCl_3_). ^1^H NMR (500 MHz, CDCl_3_) δ_H_ 5.86 (1H, dddd, *J* = 17.1, 10.1, 7.0, 7.0 Hz, 1H, 2’-H), 5.15 (br d, *J* = 17.1 Hz, 1H, 3’-H_b_), 5.06 (br d, *J* = 10.1 Hz, 1H, 3’-H_a_), 4.37 (dd, *J* = 7.3, 4.8 Hz, 1H, 3-H), 4.10 (dd, *J* = 4.4, 4.4 Hz, 1H, 2-H), 4.01–3.97 (m, 2H, 1-H, 5-H_b_), 3.93 (ddd, *J* = 7.0, 7.0, 3.5 Hz, 1H, 4-H), 3.83 (dd, *J* = 11.7, 6.3 Hz, 1H, 5-H_a_), 2.52 (ddd, *J* = 14.2, 6.9, 6.9 Hz, 1H, 1’-H_b_), 2.42 (ddd, *J* = 14.5, 7.3, 7.3 Hz, 1H, 1’-H_a_), 1.10–0.89 ppm (m, 28H, *^i^*Pr), 1H from OH is missing. ^13^C{^1^H} NMR (125.7 MHz, CDCl_3_) δ_C_ 134.6 (CH, C-2’), 117.1 (CH_2_, C-3’), 80.5 (CH, C-1 or C-4), 80.3 (CH, C-1 or C-4), 74.9 (CH, C-3), 72.4 (CH, C-2), 63.4 (CH_2_, C-5), 33.8 (CH_2_, C-1’), 17.5 (CH_3_, *^i^*Pr), 17.38 (CH_3_, *^i^*Pr), 17.35 (2 × CH_3_, *^i^*Pr), 17.2 (CH_3_, *^i^*Pr), 17.1 (2 × CH_3_, *^i^*Pr), 17.0 (CH_3_, *^i^*Pr), 13.4 (CH, *^i^*Pr), 13.2 (CH, *^i^*Pr), 12.9 (CH, *^i^*Pr), 12.6 ppm (CH, *^i^*Pr). IR (CHCl_3_): ν = 3671, 3540, 2949, 1732, 1643, 1465, 1120 cm^–1^. MS (ESI) *m*/*z* (%) = 439 (100) [M + Na]^+^. HRMS (ESI) *m*/*z*: [M + Na]^+^ calcd for C_20_H_40_NaO_5_Si_2_ 439.2312; found 439.2310.

### General Procedure of Hydroboration to Give **63**, **66**, **69**, **72**, **76**, **81**, **85**, and **87**

The corresponding allyl derivative (1 mmol) was dissolved in dry THF (10.5 mL). BH_3_·THF 1 M complex (4 mL, 4 mmol) was added under a N_2_ atmosphere at 0 °C, and then the reaction was stirred at room temperature for 1 h. At 0 °C, an aqueous solution of NaOH 3 M (20 mL) was dropwise added followed by H_2_O_2_ 30% (20 mL) and stirring was continued during 1 h at that temperature. The reaction was poured into brine and extracted with CH_2_Cl_2_. The organic layers were dried over Na_2_SO_4_ and concentrated under reduced pressure. The residue was purified by column chromatography (hexanes–EtOAc) to give the corresponding alcohol.

#### 3-*C*-(3,4,6-Tri-*O*-benzyl-α-d-glucopyranosyl)1-propanol (**63**)

Following the general procedure for the hydroboration, starting from 3-*C*-(3,4,6-tri-*O*-benzyl-α-d-glucopyranosyl)1-propene (**62**)^54^ (700 mg, 1.48 mmol) and purification by column chromatography (hexanes–EtOAc, 8:2), the alcohol **63** (478 mg, 0.97 mmol, 66%) was obtained as a crystalline solid: mp 101.5–102.3 °C (*n-*hexane–EtOAc); [α]_D_ = +31.6 (*c =* 0.31, CHCl_3_). ^1^H NMR (400 MHz, CDCl_3_) δ_H_ 7.35–7.20 (m, 15H, Ar), 4.67 (d, *J* = 11.7 Hz, 1H, OBn), 4.63 (d, *J* = 11.4 Hz, 1H, OBn), 4.59 (d, *J* = 11.8 Hz, 1H, OBn), 4.54 (d, *J* = 12.2 Hz, 1H, OBn), 4.53 (d, *J* = 11.2 Hz, 1H, OBn), 4.50 (d, *J* = 12.1 Hz, 1H, OBn), 4.00 (ddd, *J* = 5.1, 5.1, 5.1 Hz, 1H, 5-H), 3.90 (ddd, *J* = 9.5, 3.2, 3.2 Hz, 1H, 1-H), 3.79 (dd, *J* = 10.1, 6.0 Hz, 1H, 6-H_b_), 3.73 (dd, *J* = 5.8, 5.8 Hz, 1H, 3-H), 3.68–3.61 (m, 4H, 3’-H_2_, 2-H, 6-H_a_), 3.58 (dd, *J* = 5.4, 5.4 Hz, 1H, 4-H), 2.96 (br s, 1H, OH), 2.15 (br s, 1H, OH), 1.80–1.61 ppm (m, 4H, 1’-H_2_, 2’-H_2_). ^13^C{^1^H} NMR (100.6 MHz, CDCl_3_) δ_C_ 138.0 (2 × C, Ar), 137.4 (C, Ar), 127.6–128.5 (15 × CH, Ar), 78.1 (CH, C-3), 75.2 (CH, C-4), 73.5 (CH_2_, OBn), 73.3 (CH_2_, OBn), 73.3 (CH, C-5), 73.0 (CH_2_, OBn), 71.9 (CH, C-1), 70.0 (CH, C-2), 68.2 (CH_2_, C-6), 62.6 (CH_2_, C-3’), 29.2 (CH_2_, C-2’), 24.8 ppm (CH_2_, C-1’), IR (CHCl_3_): ν = 3496, 2938, 1455, 1086 cm^–1^. MS (ESI) *m*/*z* (%) = 515 (100) [M + Na]^+^. HRMS (ESI) *m*/*z*: [M + Na]^+^ calcd for C_30_H_36_NaO_6_ 515.2410; found 515.2407. Anal. calcd for C_30_H_36_O_6_: C, 73.15; H, 7.37. Found: C, 72.90; H, 7.26.

#### 3-*C*-(3,4,6-Tri-*O*-benzyl-β-d-glucopyranosyl)1-propanol (**66**)

Following the general procedure for the hydroboration, starting from 3-*C*-(3,4,6-tri-*O*-benzyl-β-d-glucopyranosyl)1-propene (**65**)^50c^ (103 mg, 0.22 mmol) and purification by column chromatography (hexanes–EtOAc, 7:3), the alcohol **66** (77.3 mg, 0.16 mmol, 73%) was obtained as a crystalline solid: mp 112.0–112.7 °C (*n-*hexane–EtOAc); [α]_D_ = +35.7 (*c =* 0.63, CHCl_3_). ^1^H NMR (400 MHz, CDCl_3_) δ_H_ 7.28–7.18 (m, 13H, Ar), 7.12–7.10 (m, 2H, Ar), 4.88 (d, *J* = 11.6 Hz, 1H, OBn), 4.71 (d, *J* = 10.8 Hz, 1H, OBn), 4.67 (d, *J* = 11.7 Hz, 1H, OBn), 4.53 (d, *J* = 12.2 Hz, 1H, OBn), 4.49 (d, *J* = 10.8 Hz, 1H, OBn), 4.45 (d, *J* = 12.1 Hz, 1H, OBn), 3.61 (ddd, *J* = 10.8, 10.8, 2.2 Hz, 1H, 5-H), 3.58–3.55 (m, 3H, 3’-H_2_, 6-H_b_), 3.51 (dd, *J* = 9.3, 9.3 Hz, 1H, 4-H), 3.39 (dd, *J* = 8.9, 8.9 Hz, 1H, 3-H), 3.37 (m, 1H, 6-H_a_), 3.26 (dd, *J* = 9.2, 9.2 Hz, 1H, 2-H), 3.15 (ddd, *J* = 8.5, 8.5, 2.4 Hz, 1H, 1-H), 2.22 (br s, 2H, 2 × OH), 1.92 (m, 1H, 1’-H_b_), 1.68–1.62 (m, 2H, 2’-H_2_), 1.49 ppm (dddd, *J* = 7.7, 7.7, 7.7, 7.7 Hz, 1H, 1’-H_a_), ^13^C{^1^H} NMR (100.6 MHz, CDCl_3_) δ_C_ 138.6 (C, Ar), 138.0 (2 × C, Ar), 128.6 (2 × CH, Ar), 128.4 (2 × CH, Ar), 128.3 (2 × CH, Ar), 127.89 (3 × CH, Ar), 127.84 (2 × CH, Ar), 127.79 (2 × CH, Ar), 127.75 (CH, Ar), 127.6 (CH, Ar), 86.8 (CH, C-3), 79.4 (CH, C-4), 78.8 (CH, C-5), 78.4 (CH, C-1), 75.2 (CH_2_, OBn), 74.7 (CH_2_, OBn), 73.7 (CH, C-2), 73.5 (CH_2_, OBn), 69.0 (CH_2_, C-6), 62.7 (CH_2_, C-3’), 28.8 (CH_2_, C-2’), 28.5 ppm (CH_2_, C-1’). IR (CHCl_3_): ν = 3588, 3500, 2928, 1455, 1052 cm^–1^. MS (ESI) *m*/*z* (%) = 515 (100) [M + Na]^+^. HRMS (ESI) *m*/*z*: [M + Na]^+^ calcd for C_30_H_36_NaO_6_ 515.2410; found 515.2412.

#### 3-*C*-(3,4,6-Tri-*O*-benzyl-α-d-mannopyranosyl)1-propanol (**69**)

Following the general procedure for the hydroboration, starting from 3-*C*-(3,4,6-tri-*O*-benzyl-α-d-mannopyranosyl)1-propene (**68**)^54,63^ (675.6 mg, 1.42 mmol) and purification by column chromatography (hexanes–EtOAc, 1:1 to 3:7), the alcohol **69** (368.4 mg, 0.75 mmol, 53%) was obtained as an amorphous solid: [α]_D_ = +34.0 (*c =* 0.45, CHCl_3_). ^1^H NMR (500 MHz, CDCl_3_) δ_H_ 7.35–7.25 (m, 13H, Ar), 7.22–7.20 (m, 2H, Ar), 4.71 (d, *J* = 11.1 Hz, 1H, OBn), 4.61 (d, *J* = 11.7 Hz, 1H, OBn), 4.57 (d, *J* = 10.1 Hz, 1H, OBn), 4.55 (d, *J* = 11.1 Hz, 1H, OBn), 4.52 (d, *J* = 11.4 Hz, 1H, OBn), 4.51 (d, *J* = 12.0 Hz, 1H, OBn), 3.89 (m, 1H, 1-H), 3.82–3.80 (m, 2H, 2-H, 3-H), 3.77–3.75 (m, 2H, 4-H, 5-H), 3.70 (dd, *J* = 10.1, 5.4 Hz, 1H, 6-H_b_), 3.68–3.60 (m, 3H, 3’-H_2_, 6-H_a_), 2.25 (br s, 2H, 2 × OH), 1.76–1.59 ppm (m, 4H, 1’-H_2_, 2’-H_2_). ^13^C{^1^H} NMR (100.6 MHz, CDCl_3_) δ_C_ 138.1 (2 × C, Ar), 137.6 (C, Ar), 127.6–128.6 (15 × CH, Ar), 79.2 (CH, C-3), 75.1 (CH, C-1), 74.2 (CH, C-5), 74.0 (CH_2_, OBn), 73.4 (CH_2_, OBn), 72.8 (CH, C-4), 72.3 (CH_2_, OBn), 69.4 (CH, C-2), 69.0 (CH_2_, C-6), 62.2 (CH_2_, C-3’), 29.2 (CH_2_, C-2’), 25.8 ppm (CH_2_, C-1’). IR (CHCl_3_): ν = 3562, 3500, 2933, 1094 cm^–1^. MS (ESI) *m*/*z* (%) = 515 (100) [M + Na]^+^. HRMS (ESI) *m*/*z*: [M + Na]^+^ calcd for C_30_H_36_NaO_6_ 515.2410; found 515.2403. Anal. calcd for C_30_H_36_O_6_: C, 78.15; H, 7.87. Found C, 78.07; H, 7.60.

#### 3-*C*-(3,4,6-Tri-*O*-benzyl-β-d-mannopyranosyl)1-propanol (**72**)

Following the general procedure for the hydroboration, starting from 3-*C*-(3,4,6-tri-*O*-benzyl-β-d-mannopyranosyl)1-propene (**71**)^54,63^ (106 mg, 0.22 mmol) and purification by column chromatography (hexanes–EtOAc, 7:3), the alcohol **72** (72.6 mg, 0.15 mmol, 67%) was obtained as a colorless oil: [α]_D_ = +2.1 (*c =* 0.48, CHCl_3_). ^1^H NMR (400 MHz, CDCl_3_) δ_H_ 7.37–7.17 (m, 15H, Ar), 4.83 (d, *J* = 10.8 Hz, 1H, OBn), 4.71 (d, *J* = 11.6 Hz, 1H, OBn), 4.64 (d, *J* = 11.7 Hz, 1H, OBn), 4.57 (d, *J* = 12.1 Hz, 1H, OBn), 4.52 (d, *J* = 12.1 Hz, 1H, OBn), 4.49 (d, *J* = 10.8 Hz, 1H, OBn), 3.90 (dd, *J* = 2.9, 0.0 Hz, 1H, 2-H), 3.74 (dd, *J* = 9.6, 9.6 Hz, 1H, 4-H), 3.70 (dd, *J* = 8.0, 1.6 Hz, 1H, 6-H_b_), 3.63 (m, 3H, 6-H_a_, 3’-H_2_), 3.57 (dd, *J* = 9.0, 3.2 Hz, 1H, 3-H), 3.40 (ddd, *J* = 9.8, 5.4, 1.9 Hz, 1H, 5-H), 3.35 (ddd, *J* = 9.2, 3.8, 0.0 Hz, 1H, 1-H), 2.94 (br s, 2H, 2 × OH), 1.88 (m, 1H, 1’-H_b_), 1.75–1.64 ppm (m, 3H, 1’-H_a_, 2’-H_2_). ^13^C{^1^H} NMR (100.6 MHz, CDCl_3_) δ_C_ 138.2 (C, Ar), 138.1 (C, Ar), 137.8 (C, Ar), 127.6–128.5 (15 × CH, Ar), 83.4 (CH, C-3), 79.0 (CH, C-1), 78.1 (CH, C-5), 75.1 (CH_2_, OBn), 74.7 (CH, C-4), 73.4 (CH_2_, OBn), 71.6 (CH_2_, OBn), 69.4 (CH_2_, C-6), 68.7 (CH, C-2), 62.6 (CH_2_, C-3’), 29.4 (CH_2_, C-2’), 28.0 ppm (CH_2_, C-1’). IR (CHCl_3_): ν = 3461, 2869, 1455, 1093 cm^–1^. MS (ESI) *m*/*z* (%) = 515 (100) [M + Na]^+^. HRMS (ESI) *m*/*z*: [M + Na]^+^ calcd for C_30_H_36_NaO_6_ 515.2410; found 515.2403. Anal. calcd for C_30_H_36_O_6_: C, 73.15; H, 7.37. Found: C, 73.36; H, 7.67.

#### 3-*C*-(3,4-Di-*O*-benzyl-α-l-fucopyranosyl)1-propanol (**76**)

Following the general procedure for the hydroboration, starting from **75** (744.3 mg, 2.02 mmol) and purification by column chromatography (hexanes–EtOAc, 2:8), the alcohol **76** (442.9 mg, 1.15 mmol, 57%) was obtained as a colorless oil: [α]_D_ = −51.4 (*c* = 0.52, CHCl_3_). ^1^H NMR (500 MHz, CDCl_3_) δ_H_ 7.35–7.25 (m, 10H, Ar), 4.78 (d, *J* = 11.7 Hz, 1H, OBn), 4.75 (d, *J* = 11.7 Hz, 1H, OBn), 4.60 (d, *J* = 11.7 Hz, 1H, OBn), 4.58 (d, *J* = 11.7 Hz, 1H, OBn), 4.07–4.06 (m, 2H, 1-H, 2-H), 3.91 (m, 1H, 5-H), 3.76 (dd, *J* = 3.2, 3.2 Hz, 1H, 4-H), 3.71 (dd, *J* = 6.6, 2.5 Hz, 1H, 3-H), 3.67–3.61 (m, 2H, 3’-H_2_), 2.40 (br s, 1H, OH), 2.21 (br s, 1H, OH), 1.69–1.62 (m, 4H, 1’-H_2_, 2’-H_2_), 1.31 ppm (d, *J* = 6.6 Hz, 3H, 6-H_3_). ^13^C{^1^H} NMR (100.6 MHz, CDCl_3_) δ_C_ 138.4 (C, Ar), 138.2 (C, Ar), 128.5 (2 × CH, Ar), 128.3 (2 × CH, Ar), 127.7 (3 × CH, Ar), 127.6 (3 × CH, Ar), 79.0 (CH, C-3), 75.1 (CH, C-4), 73.2 (CH_2_, OBn), 72.6 (CH_2_, OBn), 71.8 (CH, C-1 or C-2), 69.1 (CH, C-5), 68.9 (CH, C-1 or C-2), 62.6 (CH_2_, C-3’), 23.6 (2 × CH_2_, C-1’, C-2’), 15.6 ppm (CH_3_, C-6). IR (CHCl_3_): ν = 3585, 3422, 3016, 1228 cm^–1^. MS (ESI) *m*/*z* (%) = 409 (100) [M + Na]^+^. HRMS (ESI) *m*/*z*: [M + Na]^+^ calcd for C_23_H_30_NaO_5_ 409.1991; found 409.1990.

#### 3-*C*-(3,4-Di-*O*-benzylidene-α,β-d-arabinopyranosyl)1-propanol (**81**)

Following the general procedure for the hydroboration, starting from **79** (180 mg, 0.69 mmol) and purification by column chromatography (hexanes–EtOAc, 2:8), the alcohol **81** (106.1 mg, 0.38 mmol, 55%) was obtained as a colorless oil: ^1^H NMR (500 MHz, CDCl_3_, only the major 1*S* isomer is described) δ_H_ 7.47–7.37 (m, 5H, Ar), 6.22 (br s, 1H, PhC*H*), 4.57 (ddd, *J* = 8.9, 6.4, 5.1 Hz, 1H, 4-H), 4.29 (dd, *J* = 5.1, 2.3 Hz, 1H, 3-H), 4.08 (dd, *J* = 11.7, 6.6 Hz, 1H, 5-H_b_), 3.95 (br s, 1H, 2-H), 3.70–3.66 (m, 3H, 3’-H_2_, 1-H), 3.54 (dd, *J* = 11.7, 8.8 Hz, 1H, 5-H_a_), 2.5 (br s, 1H, OH), 2.66 (br s, 1H, OH), 1.82–1.66 ppm (m, 4H, 1’-H_2_, 2’-H_2_). ^13^C{^1^H} NMR (125.7 MHz, CDCl_3_, only the major 1*S* isomer is described) δ_C_ 139.0 (C, Ar), 129.1 (CH, Ar), 128.4 (2 × CH, Ar), 125.9 (2 × CH, Ar), 103.3 (CH, Ph*C*H), 75.7 (CH, C-1 or C-3), 75.6 (CH, C-1 or C-3), 70.1 (CH, C-4), 68.0 (CH, C-2), 65.9 (CH_2_, C-5), 62.6 (CH_2_, C-3’), 28.9 (CH_2_, C-1’ or C-2’), 26.7 ppm (CH_2_, C-1’ or C-2’). IR (CHCl_3_): ν = 3613, 3417, 2927, 1208, 1070 cm^–1^. MS (ESI) *m*/*z* (%) = 303 (100) [M + Na]^+^. HRMS (ESI) *m*/*z*: [M + Na]^+^ calcd for C_15_H_20_NaO_5_ 303.1208; found 303.1203.

#### 3-*C*-(3,5-Di-*O*-*tert*-butyldiphenylsilyl-α-d-ribofuranosyl)1-propanol (**85**)

The general procedure for the hydroboration starting from **83** (3.55 g, 5.46 mmol) but adding dropwise at 0 °C an aqueous saturated solution of NaHCO_3_ (35.5 mL) instead of the NaOH solution followed by H_2_O_2_ 30% (18 mL) gave, after purification by column chromatography (hexanes–EtOAc, 97:3 to 7:3), the alcohol **85** (1.28 mg, 1.92 mmol, 35%) as a colorless oil: [α]_D_ = +23.0 (*c =* 0.64, CHCl_3_). ^1^H NMR (500 MHz, CDCl_3_) δ_H_ 7.65–7.26 (m, 20H, Ar), 4.53 (dd, *J* = 5.4, 5.4 Hz, 1H, 3-H), 3.97 (m, 1H, 4-H), 3.88 (m, 1H, 1-H), 3.82 (dd, *J* = 5.1, 5.1 Hz, 1H, 2-H), 3.71–3.62 (m, 2H, 3’-H_2_), 3.57 (dd, *J* = 11.4, 2.2 Hz, 1H, 5-H_b_), 3.25 (dd, *J* = 11.4, 3.8 Hz, 1H, 5-H_a_), 1.82–1.77 (m, 2H, 1’-H_2_), 1.71–1.66 (m, 2H, 2’-H_2_), 1.61 (br s, 2H, OH), 1.08 (s, 9H, *^t^*Bu), 0.91 ppm (s, 9H, *^t^*Bu). ^13^C{^1^H} NMR (125.7 MHz, CDCl_3_) δ_C_ 135.67 (2 × CH, Ar), 135.62 (2 × CH, Ar), 135.58 (2 × CH, Ar), 135.53 (2 × CH, Ar), 133.3 (C, Ar), 133.2 (C, Ar), 132.5 (C, Ar), 132.3 (C, Ar), 130.25 (CH, Ar), 130.20 (CH, Ar), 129.5 (2 × CH, Ar), 128.0 (2 × CH, Ar), 127.9 (2 × CH, Ar), 127.6 (4 × CH, Ar), 83.0 (CH, C-4), 81.5 (CH, C-1), 74.6 (CH, C-3), 73.0 (CH, C-2), 64.0 (CH_2_, C-5), 62.8 (CH_2_, C-3’), 29.6 (CH_2_, C-2’), 26.9 (3 × CH_3_, *^t^*Bu), 26.7 (3 × CH_3_, *^t^*Bu), 26.1 (CH_2_, C-1’), 19.2 (C, *^t^*Bu), 19.1 ppm (C, *^t^*Bu). IR (CHCl_3_): ν = 3532, 2932, 1428, 1206, 1113 cm^–1^. MS (ESI) *m*/*z* (%) = 691 (100) [M + Na]^+^. HRMS (ESI) *m*/*z*: [M + Na]^+^ calcd for C_40_H_52_NaO_5_Si_2_ 691.3251; found 691.3250.

#### 3-*C*-(3,5-Di-*O*-1,1,3,3-tetraisopropyldisiloxanyl-α-d-ribofuranosyl)1-propanol (**87**)

The general procedure for the hydroboration starting from **86** (228 mg, 0.55 mmol) but adding dropwise at 0 °C an aqueous saturated solution of NaHCO_3_ (3.1 mL) instead of the NaOH solution followed by H_2_O_2_ 30% (1.6 mL) gave, after purification by column chromatography (hexanes–EtOAc, 1:1), the alcohol **87** (150.47 mg, 0.35 mmol, 63%) as a colorless oil: [α]_D_ = −10.8 (*c =* 0.62, CHCl_3_). ^1^H NMR (500 MHz, CDCl_3_) δ_H_ 4.38 (dd, *J* = 7.3, 4.8 Hz, 1H, 3-H), 5.00 (dd, *J* = 4.7, 4.7 Hz, 1H, 2-H), 4.00–3.96 (m, 2H, 1-H, 5-H_b_), 3.91 (ddd, *J* = 7.3, 7.3, 3.5 Hz, 1H, 4-H), 3.64 (dd, *J* = 12.0, 6.3 Hz, 1H, 5-H_a_), 3.69–3.63 (m, 2H, 3’-H_2_), 2.23 (br s, 2H, OH), 1.84 (ddd, *J* = 14.2, 7.6, 7.6 Hz, 1H, 1’-H_b_), 1.79–1.64 (m, 3H, 1’-H_a_, 2’-H_2_), 1.11–0.95 ppm (m, 28H, *^i^*Pr). ^13^C{^1^H} NMR (125.7 MHz, CDCl_3_) δ_C_ 80.8 (CH, C-1), 80.3 (CH, C-4), 74.8 (CH, C-3), 72.8 (CH, C-2), 63.2 (CH_2_, C-5), 62.7 (CH_2_, C-3’), 29.3 (CH_2_, C-2’), 25.8 (CH_2_, C-1’), 17.4 (CH_3_, *^i^*Pr), 17.33 (CH_3_, *^i^*Pr), 17.30 (2 × CH_3_, *^i^*Pr), 17.19 (CH_3_, *^i^*Pr), 17.02 (2 × CH_3_, *^i^*Pr), 16.93 (CH_3_, *^i^*Pr), 13.4 (CH, *^i^*Pr), 13.2 (CH, *^i^*Pr), 12.8 (CH, *^i^*Pr), 12.6 ppm (CH, *^i^*Pr). IR (CHCl_3_): ν = 3622, 3528, 2948, 2870, 1465, 1041 cm^–1^. MS (ESI) *m*/*z* (%) = 457 (100) [M + Na]^+^. HRMS (ESI) *m*/*z*: [M + Na]^+^ calcd for C_20_H_42_NaO_6_Si_2_ 457.2418; found 457.2413.

### General Procedure for the Mitsunobu Reaction to Give Phthalimide Derivatives **11**, **13**, **14**, **64**, **67**, **70**, **73**, **77**, and **88**

DEAD (449 μL, 2.58 mmol) was added dropwise to a stirred solution of the alcohol (1 mmol), *N-*hydroxyphthalimide (420 mg, 2.58 mmol), and PPh_3_ (670 mg, 2.58 mmol) in dry THF (10.3 mL), and the resulting solution was stirred at 0 °C for 1–4 h. Then, the solvent was removed and the crude was quenched with water and extracted with CHCl_3_. The combined extracts were dried over Na_2_SO_4_ and concentrated under reduced pressure. Column chromatography of the residue (hexanes–EtOAc) gave the corresponding phthalimide.

#### 3-*C*-(3,4-Di-*O*-benzylidene-2-*O*-diphenoxyphosphoryl-α,β-d-arabinopyranosyl)1-propoxyphthalimide (**11**)

Following the general procedure starting from alcohol **81** (95.2 mg, 0.34 mmol) stirring at 0 °C for 0.5 h, after purification by column chromatography (hexanes–EtOAc, 6:4), a phthalimide intermediate (202.4 mg) was obtained as a yellow oil. The crude (202.4 mg) was dissolved in dry CH_2_Cl_2_ (10.2 mL) under a N_2_ atmosphere. ClPO(OPh)_2_ (324 μL, 1.6 mmol) and DMAP (195.5 mg, 1.6 mmol) were added at 0 °C, and after 5 min, the mixture was stirred at room temperature for 1.5 h. The reaction was quenched with a saturated aqueous NH_4_Cl solution and extracted with CH_2_Cl_2_. The organic extracts were dried over Na_2_SO_4_ and concentrated under reduced pressure. Column chromatography of the residue (PhCH_3_–EtOAc, 9:1) gave **11** (149.4 mg, 0.23 mmol, 67%, 1*S*/1*R* 4.7:1) as a colorless oil. ^1^H NMR (500 MHz, CDCl_3_, only the major 1*S* isomer is described, simulated ring coupling constants using DAISY) δ_H_ 7.84–7.81 (m, 2H, Ar), 7.75–7.72 (m, 2H, Ar), 7.40–7.14 (m, 15H, Ar), 6.20 (s, 1H, PhC*H*), 4.88 (ddd, *J* = 3.3, 2.0 Hz, ^3^*J*_PH_ = 9.2 Hz, 1H, 2-H), 4.38 (ddd, *J* = 7.9, 6.0, 5.2 Hz, 1H, 4-H), 4.32 (dd, *J* = 5.2, 3.3 Hz, 1H, 3-H), 4.13 (ddd, *J* = 6.7, 6.7, 1.3 Hz, 2H, 3’-H_2_), 4.04 (dd, *J* = 12.2, 6.0 Hz, 1H, 5-H_b_), 3.83 (m, 1H, 1-H), 3.59 (dd, *J* = 12.2, 7.9 Hz, 1H, 5-H_a_), 1.92 (m, 1H, 2’-H_b_), 1.85–1.76 (m, 2H, 1’-H_b_, 2’-H_a_), 1.67 ppm (m, 1H, 1’-H_a_). ^13^C{^1^H} NMR (125.7 MHz, CDCl_3_, only the major 1*S* isomer is described) δ_C_ 163.6 (2 × C, CO), 150.4 (d, ^2^*J*_PC_ = 8.4 Hz, C, Ar), 150.3 (d, ^2^*J*_PC_ = 7.4 Hz, C, Ar), 138.6 (C, Ar), 134.4 (2 × CH, Ar), 129.9 (2 × CH, Ar), 129.8 (2 × CH, Ar), 129.1 (2 × C, Ar), 129.0 (CH, Ar), 128.4 (2 × CH, Ar), 126.0 (2 × CH, Ar), 125.58 (CH, Ar), 125.55 (CH, Ar), 123.5 (2 × CH, Ar), 120.2 (2 × CH, Ar), 120.1 (2 × CH, Ar), 103.4 (CH, Ph*C*H), 77.9 (CH_2_, C-3’), 75.3 (d, ^2^*J*_PC_ = 6.3 Hz, CH, C-2), 73.6 (d, ^3^*J*_PC_ = 5.3 Hz, CH, C-1 or C-3), 73.5 (d, ^3^*J*_PC_ = 2.1 Hz, CH, C-1 or C-3), 70.5 (CH, C-4), 64.6 (CH_2_, C-5), 25.8 (CH_2_, C-1’), 24.5 ppm (CH_2_, C-2’). IR (CHCl_3_): ν = 3018, 1791, 1734, 1226 cm^–1^. MS (ESI) *m*/*z* (%) = 680 (100) [M + Na]^+^. HRMS (ESI) *m*/*z*: [M + Na]^+^ calcd for C_35_H_32_NNaO_10_P 680.1662; found 680.1661. Anal. calcd for C_35_H_32_NO_10_P: C, 63.92; H, 4.90: N, 2.13. Found: C, 64.10; H, 5.16; N, 2.33.

#### 3-*C*-(2-*O*-Acetyl-3,5-di-*O*-*tert*-butyldiphenylsilyl-α-d-ribofuranosyl)1-propoxyphthalimide (**13**)

Following the general procedure starting from alcohol **85** (604 mg, 0.90 mmol) and stirring at 50 °C for 2 h, after purification by column chromatography (hexanes–EtOAc, 8:2), a phthalimide intermediate was obtained (715.4 mg, 0.88 mmol, 98%) as a colorless oil. Phthalimide (715.4 mg, 0.88 mmol) was dissolved in dry pyridine (3.4 mL), and Ac_2_O (1.15 mL) and DMAP (1.1 mg, 9.0·10^–3^ mmol) were added. The reaction was stirred at room temperature for 1 h, and then it was evaporated in a high vacuum rotovap, quenched with an aqueous solution of HCl 10%, and extracted with CH_2_Cl_2_. The combined organic extracts were dried over Na_2_SO_4_ and concentrated under reduced pressure. Column chromatography of the residue (hexanes–EtOAc, 8:2) gave **13** (589.9 mg, 0.69 mmol, 78%) as a white solid. mp 42.3–43.7 °C (*n-*hexane–EtOAc); [α]_D_ = +33.2 (*c =* 0.76, CHCl_3_). ^1^H NMR (500 MHz, CDCl_3_, simulated ring coupling constants using DAISY) δ_H_ 7.82–7.71 (m, 4H, Ar), 7.67–7.27 (m, 20H, Ar), 5.17 (dd, *J* = 4.7, 3.4 Hz, 1H, 2-H), 4.64 (dd, *J* = 6.7, 4.7 Hz, 1H, 3-H), 4.22–4.17 (m, 2H, 3’-H_2_), 4.03–3.98 (m, 2H, 1-H, 4-H), 3.61 (dd, *J* = 11.4, 2.2 Hz, 1H, 5-H_b_), 3.31 (dd, *J* = 11.4, 3.2 Hz, 1H, 5-H_a_), 2.15 (s, 3H, OAc), 1.95 (m, 1H, 2’-H_b_), 1.81–1.68 (m, 3H, 1’-H_2_, 2’-H_a_), 1.04 (s, 9H, *^t^*Bu), 0.90 ppm (s, 9H, *^t^*Bu). ^13^C{^1^H} NMR (125.7 MHz, CDCl_3_) δ_C_ 170.4 (C, OAc), 163.6 (2 × C, CO), 135.84 (2 × CH, Ar), 135.79 (2 × CH, Ar), 135.64 (2 × CH, Ar), 135.59 (2 × CH, Ar), 134.4 (2 × CH, Ar), 133.5 (C, Ar), 133.4 (C, Ar), 133.3 (C, Ar), 132.8 (C, Ar), 129.98 (CH, Ar), 129.95 (CH, Ar), 129.5 (2 × CH, Ar), 129.0 (2 × C, Ar), 127.78 (2 × CH, Ar), 127.74 (2 × CH, Ar), 127.57 (2 × CH, Ar), 127.55 (2 × CH, Ar), 123.5 (2 × CH, Ar), 82.8 (CH, C-4), 79.0 (CH, C-1), 78.3 (CH_2_, C-3’), 74.9 (CH, C-2), 72.8 (CH, C-3), 63.6 (CH_2_, C-5), 26.8 (3 × CH_3_, *^t^*Bu), 26.7 (3 × CH_3_, *^t^*Bu), 26.1 (CH_2_, C-1’), 25.0 (CH_2_, C-2’), 21.0 (CH_3_, OAc), 19.2 (C, *^t^*Bu), 19.1 ppm (C, *^t^*Bu). IR (CHCl_3_): ν = 2932, 2860, 1791, 1731, 1428, 1242, 1113 cm^–1^. MS (ESI) *m*/*z* (%) = 878 (100) [M + Na]^+^. HRMS (ESI) *m*/*z*: [M + Na]^+^ calcd for C_50_H_57_NNaO_8_Si_2_ 878.3520; found 878.3530.

#### 3-*C*-(3,5-Di-*O*-*tert*-butyldiphenylsilyl-2-*O*-trifluoromethylsulfonyl-α-d-ribofuranosyl)1-propoxyphthalimide (**14**)

Following the general procedure starting from alcohol **85** (604 mg, 0.90 mmol) and stirring at 50 °C for 2 h, after purification by column chromatography (hexanes–EtOAc, 8:2), a phthalimide intermediate was obtained (715.4 mg, 0.88 mmol, 98%) as a colorless oil. Phthalimide (715.4 mg, 0.88 mmol) was dissolved in dry pyridine (0.26 mL), and Tf_2_O (22 μL, 0.13 mmol) was added. The reaction was stirred at room temperature for 1 h, and then it was evaporated in a high vacuum rotovap, quenched with an aqueous solution of HCl 10%, and extracted with CH_2_Cl_2_. The combined organic extracts were dried over Na_2_SO_4_ and concentrated under reduced pressure. Column chromatography of the residue (hexanes–EtOAc, 8:2) gave **14** (48.4 mg, 0.051 mmol, 78%) as a white solid. mp 42.6–43.9 °C (*n-*hexane–EtOAc); [α]_D_ = +20.8 (*c =* 0.63, CHCl_3_). ^1^H NMR (500 MHz, CDCl_3_, simulated ring coupling constants using DAISY) δ_H_ 7.44–7.42 (m, 4H, Ar), 7.41–7.24 (m, 20H, Ar), 5.30 (dd, *J* = 4.6, 4.1 Hz, 1H, 2-H), 4.71 (dd, *J* = 5.7, 4.6 Hz, 1H, 3-H), 4.25–4.16 (m, 3H, 1-H, 3’-H_2_), 3.95 (m, 1H, 4-H), 3.35 (dd, *J* = 11.6, 2.0 Hz, 1H, 5-H_b_), 2.76 (dd, *J* = 11.6, 3.1 Hz, 1H, 5-H_a_), 2.05–1.92 (m, 3H, 1’-H_2_ or 2’-H_2_, 1’-H_b_ or 2’-H_b_), 1.82 (dddd, *J* = 12.9, 12.9, 6.3, 6.3 Hz, 1H, 1’-H_a_ or 2’-H_a_), 1.06 (s, 9H, *^t^*Bu), 0.85 ppm (s, 9H, *^t^*Bu). ^13^C{^1^H} NMR (125.7 MHz, CDCl_3_) δ_C_ 163.5 (2 × C, CO), 135.9 (2 × CH, Ar), 135.8 (2 × CH, Ar), 135.5 (4 × CH, Ar), 134.4 (2 × CH, Ar), 133.2 (C, Ar), 133.03 (C, Ar), 132.97 (C, Ar), 131.6 (C, Ar), 130.13 (CH, Ar), 130.06 (CH, Ar), 129.58 (CH, Ar), 129.56 (CH, Ar), 129.0 (2 × C, Ar), 127.9 (2 × CH, Ar), 127.7 (2 × CH, Ar), 127.55 (4 × CH, Ar), 123.5 (2 × CH, Ar), 89.3 (CH, C-2), 82.6 (CH, C-4), 77.84 (CH, C-1), 77.81 (CH_2_, C-3’), 73.1 (CH, C-3), 63.5 (CH_2_, C-5), 26.65 (3 × CH_3_, *^t^*Bu), 26.63 (3 × CH_3_, *^t^*Bu), 25.9 (CH_2_, C-1’ or C-2’), 24.8 (CH_2_, C-1’ or C-2’), 19.2 (C, *^t^*Bu), 19.0 ppm (C, *^t^*Bu), 1C from CF_3_ group is missing. IR (CHCl_3_): ν = 2932, 1791, 1734, 1113 cm^–1^. MS (ESI) *m*/*z* (%) = 968 (100) [M + Na]^+^. HRMS (ESI) *m*/*z*: [M + Na]^+^ calcd for C_49_H_54_F_3_NNaO_9_SSi_2_ 968.2908; found 968.2907. Anal. calcd for C_49_H_54_F_3_NO_9_SSi_2_: C, 62.20; H, 5.75; N, 1.48; S, 3.39. Found: C, 62.11; H, 5.97; N, 1.52; S, 3.19.

#### 3-*C*-(3,4,6-Tri-*O*-benzyl-α-d-glucopyranosyl)1-propoxyphthalimide (**64**)

Following the general procedure starting from alcohol **63** (85.6 mg, 0.18 mmol) and purification by column chromatography (hexanes–Et_2_O, 1:1), product **64** (107 mg, 0.17 mmol, 93%) was obtained as a colorless oil: [α]_D_ = +12.3 (*c =* 0.26, CHCl_3_). ^1^H NMR (400 MHz, CDCl_3_) δ_H_ 7.82–7.71 (m, 4H, Ar), 7.34–7.21 (m, 15H, Ar), 4.69 (d, *J* = 11.5 Hz, 1H, OBn), 4.63 (d, *J* = 11.4 Hz, 1H, OBn), 4.62 (d, *J* = 11.7 Hz, 1H, OBn), 4.56 (d, *J* = 12.4 Hz, 1H, OBn), 4.56 (d, *J* = 12.4 Hz, 1H, OBn), 4.48 (d, *J* = 12.0 Hz, 1H, OBn), 4.26–4.21 (m, 2H, 3’-H_2_), 4.02–3.96 (m, 2H, 1-H, 2-H), 3.82 (dd, *J* = 10.2, 5.7 Hz, 1H, 6-H_b_), 3.77 (dd, *J* = 5.8, 5.8 Hz, 1H, 4-H), 3.73–3.68 (m, 2H, 5-H, 6-H_a_), 3.63 (dd, *J* = 5.3, 5.3 Hz, 1H, 3-H), 1.99–1.76 ppm (m, 4H, 1’-H_2_, 2’-H_2_), 1H from OH is missing. ^13^C{^1^H} NMR (100.6 MHz, CDCl_3_) δ_C_ 163.6 (2 × C, CO), 138.1 (2 × C, Ar), 137.5 (C, Ar), 134.3 (2 × CH, Ar), 129.0 (2 × C, Ar), 128.5 (2 × CH, Ar), 128.4 (2 × CH, Ar), 128.3 (2 × CH, Ar), 127.84 (3 × CH, Ar), 127.79 (CH, Ar), 127.7 (2 × CH, Ar), 127.58 (2 × CH, Ar), 127.56 (CH, Ar), 123.4 (2 × CH, Ar), 78.3 (CH_2_, C-3’), 78.1 (CH, C-3), 75.3 (CH, C-4), 73.5 (CH_2_, OBn), 73.3 (CH_2_, OBn), 73.3 (CH, C-5), 72.9 (CH_2_, OBn), 71.4 (CH, C-1), 69.8 (CH, C-2), 68.3 (CH_2_, C-6), 24.5 (CH_2_, C-1’), 24.3 ppm (CH_2_, C-2’). IR (CHCl_3_): ν = 3514, 2935, 2871, 1792, 1737, 1372, 1083 cm^–1^. MS (E/I 70 eV): *m*/*z* (%) = 546 (6) [M – C_7_H_7_]^+^, 529 (51) [M – C_7_H_8_O]^+^, 91 (100) [C_7_H_7_]^+^. HRMS (E/I): *m*/*z*: [M – C_7_H_8_O]^+^ calcd for C_31_H_31_NO_7_ 529.2101; found 529.2122.

#### 3-*C*-(3,4,6-Tri-*O*-benzyl-β-d-glucopyranosyl)1-propoxyphthalimide (**67**)

Following the general procedure starting from alcohol **66** (810 mg, 1.64 mmol) and purification by column chromatography (hexanes–EtOAc, 1:1), product **67** (790 mg, 1.24 mmol, 76%) was obtained as a colorless oil: [α]_D_ = +14.5 (*c* = 0.53, CHCl_3_). ^1^H NMR (400 MHz, CDCl_3_) δ_H_ 7.82–7.79 (m, 2H, Ar), 7.75–7.72 (m, 2H, Ar), 7.37–7.18 (m, 15H, Ar), 4.95 (d, *J* = 11.6 Hz, 1H, OBn), 4.80 (d, *J* = 11.0 Hz, 1H, OBn), 4.80 (d, *J* = 11.0 Hz, 1H, OBn), 4.61 (d, *J* = 12.2 Hz, 1H, OBn), 4.58 (d, *J* = 10.7 Hz, 1H, OBn), 4.53 (d, *J* = 12.3 Hz, 1H, OBn), 4.24 (ddd, *J* = 9.5, 6.6, 1.2 Hz, 2H, 3’-H_2_), 3.71 (br s, 2H, 6-H_2_), 3.61 (dd, *J* = 9.2, 9.2 Hz, 1H, 4-H), 3.52 (dd, *J* = 8.6, 8.6 Hz, 1H, 3-H), 3.42 (ddd, *J* = 9.6, 2.9, 2.9 Hz, 1H, 5-H), 3.39 (dd, *J* = 9.1, 9.1 Hz, 1H, 2-H), 3.28 (ddd, *J* = 8.2, 8.2, 2.2 Hz, 1H, 1-H), 2.14–1.98 (m, 2H, 1’-H_b_, 2’-H_b_), 1.87 (m, 1H, 2’-H_a_), 1.71 ppm (m, 1H, 1’-H_a_), 1H from OH is missing. ^13^C{^1^H} NMR (100.6 MHz, CDCl_3_) δ_C_ 163.7 (2 × C, CO), 138.7 (C, Ar), 138.2 (2 × C, Ar), 134.8 (2 × CH, Ar), 129.4 (2 × C, Ar), 129.0 (2 × CH, Ar), 128.8 (2 × CH, Ar), 128.7 (2 × CH, Ar), 128.31 (2 × CH, Ar), 128.25 (2 × CH, Ar), 128.2 (CH, Ar), 128.14 (2 × CH, Ar), 128.11 (CH, Ar), 127.9 (CH, Ar), 123.9 (2 × CH, Ar), 87.0 (CH, C-3), 79.1 (CH, C-1 or C-5), 78.9 (CH, C-1 or C-5), 78.6 (CH_2_, C-3’), 78.4 (CH, C-4), 75.2 (CH_2_, OBn), 74.8 (CH_2_, OBn), 73.9 (CH, C-2), 73.5 (CH_2_, OBn), 69.1 (CH_2_, C-6), 27.7 (CH_2_, C-1’), 24.0 ppm (CH_2_, C-2’). IR (CHCl_3_): ν = 3565, 2926, 2860, 1791, 1737, 1370, 1097 cm^–1^. MS (ESI) *m*/*z* (%) = 660 (100) [M + Na]^+^. HRMS (ESI) *m*/*z*: [M + Na]^+^ calcd for C_38_H_39_NNaO_8_ 660.2573; found 660.2558.

#### 3-*C*-(3,4,6-Tri-*O*-benzyl-α-d-mannopyranosyl)1-propoxyphthalimide (**70**)

Following the general procedure starting from alcohol **69** (178.8 mg, 0.36 mmol) and purification by column chromatography (hexanes–EtOAc, 1:1), product **70** (206 mg, 0.32 mmol, 90%) was obtained as a colorless oil: [α]_D_ = +13.5 (*c* = 1.50, CHCl_3_). ^1^H NMR (500 MHz, CDCl_3_) δ_H_ 7.83–7.81 (m, 2H, Ar), 7.74–7.73 (m, 2H, Ar), 7.34–7.20 (m, 15H, Ar), 4.73 (d, *J* = 11.4 Hz, 1H, OBn), 4.65 (d, *J* = 12.3 Hz, 1H, OBn), 4.63 (d, *J* = 11.7 Hz, 1H, OBn), 4.56 (d, *J* = 12.0 Hz, 1H, OBn), 4.53 (d, *J* = 11.4 Hz, 1H, OBn), 4.50 (d, *J* = 12.0 Hz, 1H, OBn), 4.27–4.19 (m, 2H, 3’-H_2_), 3.97 (m, 1H, 1-H), 3.89 (dd, *J* = 3.6, 2.9 Hz, 1H, 2-H), 3.83 (dd, *J* = 7.2, 3.1 Hz, 1H, 3-H), 3.81 (dd, *J* = 6.6, 6.6 Hz, 1H, 4-H), 3.77 (m, 1H, 5-H), 3.73 (dd, *J* = 10.4, 5.1 Hz, 1H, 6-H_b_), 3.68 (dd, *J* = 10.4, 3.5 Hz, 1H, 6-H_a_), 1.94 (m, 1H, 2’-H_b_), 1.88–1.78 ppm (m, 3H, 1’-H_2_, 2’-H_a_), 1H from OH is missing. ^13^C{^1^H} NMR (100.6 MHz, CDCl_3_) δ_C_ 163.6 (2 × C, CO), 138.3 (C, Ar), 138.2 (C, Ar), 137.7 (C, Ar), 134.4 (2 × CH, Ar), 129.0 (2 × C, Ar), 128.6 (2 × CH, Ar), 128.4 (2 × CH, Ar), 128.3 (2 × CH, Ar), 128.0 (CH, Ar), 127.9 (4 × CH, Ar), 127.73 (2 × CH, Ar), 127.68 (CH, Ar), 127.5 (CH, Ar), 123.5 (2 × CH, Ar), 79.4 (CH, C-3 or C-4), 78.0 (CH_2_, C-3’), 74.8 (CH, C-1), 74.3 (CH, C-5), 74.1 (CH_2_, OBn), 73.4 (CH_2_, OBn), 72.8 (CH, C-3 or C-4), 72.3 (CH_2_, OBn), 69.3 (CH, C-2), 69.1 (CH_2_, C-6), 25.5 (CH_2_, C-1’ or C-2’), 24.6 ppm (CH_2_, C-1’ or C-2’). IR (CHCl_3_): ν = 3559, 2930, 1789, 1731, 1082 cm^–1^. MS (ESI) *m*/*z* (%) = 660 (100) [M + Na]^+^. HRMS (ESI) *m*/*z*: [M + Na]^+^ calcd for C_38_H_39_NNaO_8_ 660.2573; found 660.2578.

#### 3-*C*-(3,4,6-Tri-*O*-benzyl-β-d-mannopyranosyl)1-propoxyphthalimide (**73**)

Following the general procedure starting from alcohol **72** (72.6 mg, 0.15 mmol) and purification by column chromatography (hexanes–Et_2_O, 1:1), product **73** (80 mg, 0.13 mmol, 84%) was obtained as a colorless oil: [α]_D_ = +5.3 (*c* = 0.34, CHCl_3_). ^1^H NMR (500 MHz, CDCl_3_, simulated ring coupling constants using DAISY) δ_H_ 7.83–7.80 (m, 2H, Ar), 7.75–7.72 (m, 2H, Ar), 7.38–7.18 (m, 15H, Ar), 4.85 (d, *J* = 10.7 Hz, 1H, OBn), 4.75 (d, *J* = 11.7 Hz, 1H, OBn), 4.67 (d, *J* = 11.7 Hz, 1H, OBn), 4.60 (d, *J* = 12.3 Hz, 1H, OBn), 4.53 (d, *J* = 10.4 Hz, 1H, OBn), 4.53 (d, *J* = 12.3 Hz, 1H, OBn), 4.29–4.20 (m, 2H, 3’-H_2_), 3.98 (dd, *J* = 2.9, 0.6 Hz, 1H, 2-H), 3.78 (dd, *J* = 9.4, 9.2 Hz, 1H, 4-H), 3.72 (dd, *J* = 11.3, 1.9 Hz, 1H, 6-H_b_), 3.68 (dd, *J* = 11.3, 5.0 Hz, 1H, 6-H_a_), 3.63 (dd, *J* = 9.2, 2.9 Hz, 1H, 3-H), 3.47 (ddd, *J* = 7.9, 4.1, 0.6 Hz, 1H, 1-H), 3.41 (ddd, *J* = 9.4, 5.0, 1.9 Hz, 1H, 5-H), 2.31 (br s, 1H, OH), 2.04–1.94 (m, 2H, 1’-H_b_, 2’-H_b_), 1.92–1.84 ppm (m, 2H, 1’-H_a_, 2’-H_a_). ^13^C{^1^H} NMR (100.6 MHz, CDCl_3_) δ_C_ 163.6 (2 × C, CO), 138.4 (2 × C, Ar), 137.9 (C, Ar), 134.4 (2 × CH, Ar), 129.0 (2 × C, Ar), 128.5 (2 × CH, Ar), 128.30 (2 × CH, Ar), 128.26 (2 × CH, Ar), 127.93 (2 × CH, Ar), 127.88 (3 × CH, Ar), 127.8 (2 × CH, Ar), 127.6 (CH, Ar), 127.5 (CH, Ar), 123.4 (2 × CH, Ar), 83.6 (CH, C-3), 79.2 (CH, C-1), 78.3 (CH_2_, C-3’), 77.3 (CH, C-5), 75.1 (CH_2_, OBn), 74.8 (CH, C-4), 73.4 (CH_2_, OBn), 71.6 (CH_2_, OBn), 69.5 (CH_2_, C-6), 68.4 (CH, C-2), 27.1 (CH_2_, C-2’), 24.7 ppm (CH_2_, C-1’). IR (CHCl_3_): ν = 3569, 2927, 2862, 1792, 1737, 1118 cm^–1^. MS (ESI) *m*/*z* (%) = 660 (100) [M + Na]^+^. HRMS (ESI) *m*/*z*: [M + Na]^+^ calcd for C_38_H_39_NNaO_8_ 660.2573; found 660.2576. Anal. calcd for C_38_H_39_NO_8_: C, 71.57; H, 6.16; N, 2.20. Found: C, 71.62; H, 6.30; N, 1.95.

#### 3-*C*-(3,4-Di-*O*-benzyl-α-l-fucopyranosyl)1-propoxyphthalimide (**77**)

Following the general procedure starting from alcohol **76** (413 mg, 1.07 mmol) and purification by column chromatography (hexanes–EtOAc, 7:3), product **77** (536.9 mg, 1.01 mmol, 94%) was obtained as a colorless oil: [α]_D_ = −14.4 (*c* = 0.80, CHCl_3_). ^1^H NMR (500 MHz, CDCl_3_) δ_H_ 7.83–7.81 (m, 2H, Ar), 7.74–7.73 (m, 2H, Ar), 7.37–7.24 (m, 10H, Ar), 4.79 (d, *J* = 11.7 Hz, 1H, OBn), 4.76 (d, *J* = 11.9 Hz, 1H, OBn), 4.62 (d, *J* = 12.0 Hz, 1H, OBn), 4.61 (d, *J* = 12.0 Hz, 1H, OBn), 4.28–4.21 (m, 2H, 3’-H_2_), 4.14–4.07 (m, 2H, 1-H, 2-H), 3.94 (m, 1H, 5-H), 3.79 (dd, *J* = 2.8, 2.8 Hz, 1H, 4-H), 3.76 (dd, *J* = 7.3, 2.9 Hz, 1H, 3-H), 2.52 (br s, 1H, OH), 2.04–1.77 (m, 4H, 1’-H_2_, 2’-H_2_), 1.30 ppm (d, *J* = 6.7 Hz, 3H, 6-H_3_). ^13^C{^1^H} NMR (125.7 MHz, CDCl_3_) δ_C_ 163.6 (2 × C, CO), 138.5 (C, Ar), 138.3 (C, Ar), 128.9 (2 × C, Ar), 128.5 (2 × CH, Ar), 128.4 (3 × CH, Ar), 128.2 (2 × CH, Ar), 127.7 (2 × CH, Ar), 127.6 (CH, Ar), 127.5 (2 × CH, Ar), 123.4 (2 × CH, Ar), 79.2 (CH, C-3), 78.3 (CH_2_, C-3’), 75.3 (CH, C-4), 73.2 (CH_2_, OBn), 72.5 (CH_2_, OBn), 71.9 (CH, C-2), 68.7 (CH, C-1), 68.6 (CH, C-5), 24.9 (CH_2_, C-1’ or C-2’), 22.8 (CH_2_, C-1’ or C-2’), 15.7 ppm (CH_3_, C-6). IR (CHCl_3_): ν = 3675, 3574, 3015, 1790, 1733, 1120 cm^–1^. MS (ESI) *m*/*z* (%) = 554 (100) [M + Na]^+^. HRMS (ESI) *m*/*z*: [M + Na]^+^ calcd for C_31_H_33_NNaO_7_ 554.2155; found 554.2147.

#### 3-*C*-(3,5-Di-*O*-1,1,3,3-tetraisopropyldisiloxanyl-α-d-ribofuranosyl)1-propoxyphthalimide (**88**)

Following the general procedure starting from alcohol **87** (350 mg, 0.81 mmol) and purification by column chromatography (hexanes–EtOAc, 8:2), product **88** (457.9 mg, 0.79 mmol, 98%) was obtained as a colorless oil: [α]_D_ = −13.0 (*c =* 0.73, CHCl_3_). ^1^H NMR (400 MHz, CDCl_3_) δ_H_ 7.83–7.72 (m, 4H, Ar), 4.36 (dd, *J* = 7.2, 4.8 Hz, 1H, 3-H), 4.25–4.20 (m, 2H, 3’-H_2_), 4.11 (dd, *J* = 4.8, 3.4 Hz, 1H, 2-H), 4.04 (m, 1H, 1-H), 3.95 (dd, *J* = 11.4, 3.2 Hz, 1H, 5-H_b_), 3.89 (ddd, *J* = 6.1, 6.1, 3.4 Hz, 1H, 4-H), 3.83 (dd, *J* = 11.6, 6.1 Hz, 1H, 5-H_a_), 1.96–1.80 (m, 4H, 1’-H_2_, 2’-H_2_), 1.09–0.97 ppm (m, 28H, *^i^*Pr), 1H from OH is missing. ^13^C{^1^H} NMR (125.7 MHz, CDCl_3_) δ_C_ 163.6 (2 × C, CO), 134.4 (2 × CH, Ar), 129.0 (2 × C, Ar), 123.4 (2 × CH, Ar), 80.2 (CH, C-1 or C-4), 80.2 (CH, C-1 or C-4), 78.3 (CH_2_, C-3’), 74.6 (CH, C-3), 72.5 (CH, C-2), 63.1 (CH_2_, C-5), 25.3 (CH_2_, C-1’ or C-2’), 24.7 (CH_2_, C-1’ or C-2’), 17.43 (CH_3_, *^i^*Pr), 17.31 (3 × CH_3_, *^i^*Pr), 17.19 (CH_3_, *^i^*Pr), 17.02 (2 × CH_3_, *^i^*Pr), 16.94 (CH_3_, *^i^*Pr), 13.4 (CH, *^i^*Pr), 13.2 (CH, *^i^*Pr), 12.8 (CH, *^i^*Pr), 12.6 ppm (CH, *^i^*Pr). IR (CHCl_3_): ν = 3546, 2948, 1791, 1733, 1467, 1039 cm^–1^. MS (ESI) *m*/*z* (%) = 602 (100) [M + Na]^+^. HRMS (ESI) *m*/*z*: [M + Na]^+^ calcd for C_28_H_45_NNaO_8_Si_2_ 602.2581; found 602.2585.

### General Procedure to Give Acetyl Derivatives **1**, **3**, **5**, **7**, and **9**

The phthalimide (1 mmol) was dissolved in dry pyridine (3.83 mL), and acetyl anhydride (1.1 mL) and DMAP (12.6 mg, 0.1 mmol) were added at 0 °C under a N_2_ atmosphere. The mixture was stirred at room temperature for 1 h. Then, the reaction was evaporated on the high vacuum rotovap, and the crude was quenched with HCl 10% and extracted with CH_2_Cl_2_. The organic extracts were washed with a saturated aqueous NaHCO_3_ solution, dried over Na_2_SO_4_, and concentrated under reduced pressure. Column chromatography of the residue (hexanes–EtOAc) gave the corresponding acetyl compound.

#### 3-*C*-(2-*O*-Acetyl-3,4,6-tri-*O*-benzyl-α-d-glucopyranosyl)1-propoxyphthalimide (**1**)

Following the general procedure starting from phthalimide **64** (39.2 mg, 0.06 mmol) and purification by column chromatography (hexanes–EtOAc, 85:15), product **1** (30 mg, 0.04 mmol, 72%) was obtained as a crystalline solid: mp 99.7–100.5 °C (*n*-hexane–EtOAc); [α]_D_ = +46.8 (*c =* 0.31, CHCl_3_). ^1^H NMR (500 MHz, CDCl_3_, simulated ring coupling constants using DAISY) δ_H_ 7.83–7.80 (m, 2H, Ar), 7.74–7.71 (m, 2H, Ar), 7.34–7.15 (m, 15H, Ar), 5.08 (dd, *J* = 9.0, 5.5 Hz, 1H, 2-H), 4.78 (d, *J* = 11.7 Hz, 1H, OBn), 4.75 (d, *J* = 11.1 Hz, 1H, OBn), 4.74 (d, *J* = 11.4 Hz, 1H, OBn), 4.60 (d, *J* = 12.3 Hz, 1H, OBn), 4.50 (d, *J* = 11.1 Hz, 1H, OBn), 4.48 (d, *J* = 12.0 Hz, 1H, OBn), 4.24 (dd, *J* = 6.0, 6.0 Hz, 2H, 3’-H_2_), 4.17 (ddd, *J* = 11.0, 5.5, 3.1 Hz, 1H, 1-H), 3.87 (dd, *J* = 9.0, 7.8 Hz, 1H, 3-H), 3.73–3.65 (m, 4H, 4-H, 5-H, 6-H_2_), 2.04 (s, 3H, OAc), 1.99–1.90 (m, 2H, 1’-H_b_, 2’-H_b_), 1.84–1.73 ppm (m, 2H, 1’-H_a_, 2’-H_a_). ^13^C{^1^H} NMR (100.6 MHz, CDCl_3_) δ_C_ 170.1 (C, OAc), 163.5 (2 × C, CO), 138.5 (C, Ar), 138.1 (2 × C, Ar), 134.4 (2 × CH, Ar), 129.0 (2 × C, Ar), 128.4 (2 × CH, Ar), 128.34 (2 × CH, Ar), 128.31 (2 × CH, Ar), 127.9 (2 × CH, Ar), 127.73 (2 × CH, Ar), 127.69 (CH, Ar), 127.61 (CH, Ar), 127.59 (2 × CH, Ar), 127.5 (CH, Ar), 123.4 (2 × CH, Ar), 80.1 (CH, C-3), 77.9 (CH_2_, C-3’), 77.6 (CH, C-4 or C-5), 74.8 (CH_2_, OBn), 74.6 (CH_2_, OBn), 73.5 (CH_2_, OBn), 73.0 (CH, C-2), 72.1 (CH, C-1), 71.9 (CH, C-4 or C-5), 69.0 (CH_2_, C-6), 24.3 (CH_2_, C-1’ or C-2’), 22.1 (CH_2_, C-1’ or C-2’), 20.9 ppm (CH_3_, OAc). IR (CHCl_3_): ν = 3013, 2870, 1790, 1734, 1236 cm^–1^. MS (ESI) *m*/*z* (%) = 702 (100) [M + Na]^+^. HRMS (ESI) *m*/*z*: [M + Na]^+^ calcd for C_40_H_41_NNaO_9_ 702.2679; found 702.2680. Anal. calcd for C_40_H_41_NO_9_: C, 70.68; H, 6.08; N, 2.06. Found C, 70.55; H, 6.07; N, 2.24.

#### 3-*C*-(2-*O*-Acetyl-3,4,6-tri-*O*-benzyl-β-d-glucopyranosyl)1-propoxyphthalimide (**3**)

Following the general procedure starting from phthalimide **67** (790 mg, 1.26 mmol) and purification by column chromatography (hexanes–EtOAc, 7:3 to 1:1), product **3** (600 mg, 0.88 mmol, 71%) was obtained as a colorless oil: [α]_D_ = +17.7 (*c =* 0.62, CHCl_3_). ^1^H NMR (400 MHz, CDCl_3_) δ_H_ 7.82–7.78 (m, 2H, Ar), 7.74–7.70 (m, 2H, Ar), 7.33–7.16 (m, 15H, Ar), 4.90 (dd, *J* = 9.5, 9.5 Hz, 1H, 2-H), 4.82 (d, *J* = 11.6 Hz, 1H, OBn), 4.78 (d, *J* = 10.8 Hz, 1H, OBn), 4.67 (d, *J* = 11.4 Hz, 1H, OBn), 4.60 (d, *J* = 12.3 Hz, 1H, OBn), 4.55 (d, *J* = 12.6 Hz, 1H, OBn), 4.51 (d, *J* = 12.3 Hz, 1H, OBn), 4.25 (m, 1H, 3’-H_b_), 4.17 (m, 1H, 3’-H_a_), 3.74–3.64 (m, 4H, 3-H, 4-H, 6-H_2_), 3.44 (ddd, *J* = 8.0, 4.0, 2.2 Hz, 1H, 5-H), 3.38 (ddd, *J* = 9.2, 9.2, 1.9 Hz, 1H, 1-H), 2.01 (m, 1H, 2’-H_b_), 1.99 (s, 3H, OAc), 1.89–1.81 (m, 2H, 1’-H_b_, 2’-H_a_), 1.60 ppm (m, 1H, 1’-H_a_). ^13^C{^1^H} NMR (100.6 MHz, CDCl_3_) δ_C_ 170.0 (C, OAc), 163.5 (2 × C, CO), 138.4 (C, Ar), 138.2 (C, Ar), 138.1 (C, Ar), 134.3 (2 × CH, Ar), 129.0 (2 × C, Ar), 128.4 (2 × CH, Ar), 128.33 (2 × CH, Ar), 128.28 (2 × CH, Ar), 127.9 (2 × CH, Ar), 127.7 (CH, Ar), 127.64 (4 × CH, Ar), 127.56 (CH, Ar), 127.5 (CH, Ar), 123.4 (2 × CH, Ar), 84.7 (CH, C-2), 79.1 (CH, C-3), 78.4 (CH, C-4), 78.1 (CH_2_, C-3’), 77.4 (CH, C-5), 75.1 (CH_2_, OBn), 74.9 (CH_2_, OBn), 73.8 (CH, C-1), 73.4 (CH_2_, OBn), 69.0 (CH_2_, C-6), 27.5 (CH_2_, C-1’), 24.0 (CH_2_, C-2’), 20.9 ppm (CH_3_, OAc). IR (CHCl_3_): ν = 3032, 2926, 2863, 1792, 1737, 1455, 1371, 1231, 1103 cm^–1^. MS (ESI) *m*/*z* (%) = 702 (100) [M + Na]^+^. HRMS (ESI) *m*/*z*: [M + Na]^+^ calcd for C_40_H_41_NNaO_9_ 702.2679; found 702.2689. Anal. calcd for C_40_H_41_NO_9_: C, 70.68; H, 6.08; N, 2.06. Found: C, 70.69; H, 6.20; N, 2.33.

#### 3-*C*-(2-*O*-Acetyl-3,4,6-tri-*O*-benzyl-α-d-mannopyranosyl)1-propoxyphthalimide (**5**)

Following the general procedure starting from phthalimide **70** (99.2 mg, 0.16 mmol) and purification by column chromatography (hexanes–EtOAc, 6:4), product **5** (90.7 mg, 0.13 mmol, 86%) was obtained as a colorless oil: [α]_D_ = +5.4 (*c =* 0.43, CHCl_3_). ^1^H NMR (500 MHz, CDCl_3_, simulated ring coupling constants using DAISY) δ_H_ 7.83–7.81 (m, 2H, Ar), 7.74–7.72 (m, 2H, Ar), 7.33–7.17 (m, 15H, Ar), 5.30 (dd, *J* = 3.4, 2.6 Hz, 1H, 2-H), 4.82 (d, *J* = 11.0 Hz, 1H, OBn), 4.69 (d, *J* = 11.4 Hz, 1H, OBn), 4.63 (d, *J* = 12.0 Hz, 1H, OBn), 4.54 (d, *J* = 11.4 Hz, 1H, OBn), 4.50 (d, *J* = 12.3 Hz, 1H, OBn), 4.49 (d, *J* = 11.1 Hz, 1H, OBn), 4.27–4.19 (m, 2H, 3’-H_2_), 4.03 (ddd, *J* = 10.9, 4.1, 2.6 Hz, 1H, 1-H), 3.93 (dd, *J* = 8.7, 3.4 Hz, 1H, 3-H), 3.85 (dd, *J* = 8.7, 8.4 Hz, 1H, 4-H), 3.78–3.69 (m, 3H, 5-H, 6-H_2_), 2.14 (s, 3H, OAc), 2.01–1.91 (m, 2H, 1’-H_2_), 1.87–1.77 ppm (m, 2H, 2’-H_2_). ^13^C{^1^H} NMR (100.6 MHz, CDCl_3_) δ_C_ 170.6 (C, OAc), 163.6 (2 × C, CO), 138.4 (2 × C, Ar), 137.9 (C, Ar), 134.4 (2 × CH, Ar), 129.0 (2 × C, Ar), 128.4 (2 × CH, Ar), 128.31 (2 × CH, Ar), 128.29 (2 × CH, Ar), 128.1 (2 × CH, Ar), 127.9 (2 × CH, Ar), 127.8 (CH, Ar), 127.7 (2 × CH, Ar), 127.6 (CH, Ar), 127.5 (CH, Ar), 123.5 (2 × CH, Ar), 77.9 (CH, C-3), 77.8 (CH_2_, C-3’), 75.0 (CH, C-4), 74.8 (CH, C-5), 74.7 (CH_2_, OBn), 73.5 (CH_2_, OBn), 72.8 (CH, C-1), 72.0 (CH_2_, OBn), 70.7 (CH, C-2), 69.4 (CH_2_, C-6), 25.0 (CH_2_, C-1’ or C-2’), 24.8 (CH_2_, C-1’ or C-2’), 21.2 ppm (CH_3_, OAc). IR (CHCl_3_): ν = 3034, 2929, 1790, 1734, 1189 cm^–1^. MS (ESI) *m*/*z* (%) = 702 (100) [M + Na]^+^. HRMS (ESI) *m*/*z*: [M + Na]^+^ calcd for C_40_H_41_NNaO_9_ 702.2679; found 702.2687. Anal. calcd for C_40_H_41_NO_9_: C, 70.68; H, 6.08; N, 2.06. Found: C, 70.74; H, 6.12; N, 2.04.

#### 3-*C*-(2-*O*-Acetyl-3,4,6-tri-*O*-benzyl-β-d-mannopyranosyl)1-propoxyphthalimide (**7**)

Following the general procedure starting from phthalimide **73** (77 mg, 0.11 mmol) and purification by column chromatography (hexanes–EtOAc, 8:2), product **7** (60 mg, 0.09 mmol, 80%) was obtained as a colorless oil: [α]_D_ = −16.7 (*c =* 0.63, CHCl_3_). ^1^H NMR (500 MHz, CDCl_3_, simulated coupling constants using DAISY) δ_H_ 7.82–7.79 (m, 2H, Ar), 7.74–7.72 (m, 2H, Ar), 7.34–7.16 (m, 15H, Ar), 5.52 (dd, *J* = 3.3, 1.0 Hz, 1H, 2-H), 4.86 (d, *J* = 10.8 Hz, 1H, OBn), 4.77 (d, *J* = 11.1 Hz, 1H, OBn), 4.64 (d, *J* = 12.3 Hz, 1H, OBn), 4.52 (d, *J* = 11.7 Hz, 1H, OBn), 4.50 (d, *J* = 10.4 Hz, 1H, OBn), 4.50 (d, *J* = 10.4 Hz, 1H, OBn), 4.25–4.18 (m, 2H, 3’-H_2_), 3.77 (dd, *J* = 9.8, 9.3 Hz, 1H, 4-H), 3.74 (m, 2H, 6-H_2_), 3.70 (dd, *J* = 9.3, 3.3 Hz, 1H, 3-H), 3.63 (ddd, *J* = 8.3, 4.7, 1.0 Hz, 1H, 1-H), 3.47 (ddd, *J* = 9.8, 5.3, 2.1 Hz, 1H, 5-H), 2.19 (s, 3H, OAc), 1.97 (m, 1H, 2’-H_b_), 1.90–1.83 (m, 2H, 1’-H_b_, 2’-H_a_), 1.72 ppm (m, 1H, 1’-H_a_). ^13^C{^1^H} NMR (125.7 MHz, CDCl_3_) δ_C_ 170.8 (C, OAc), 163.6 (2 × C, CO), 138.4 (C, Ar), 138.3 (C, Ar), 137.9 (C, Ar), 134.4 (2 × CH, Ar), 128.9 (2 × C, Ar), 128.34 (2 × CH, Ar), 128.26 (4 × CH, Ar), 128.1 (2 × CH, Ar), 127.9 (2 × CH, Ar), 127.8 (2 × CH, Ar), 127.7 (CH, Ar), 127.6 (CH, Ar), 127.5 (CH, Ar), 123.4 (2 × CH, Ar), 81.9 (CH, C-3), 79.4 (CH, C-5), 78.0 (CH_2_, C-3’), 76.2 (CH, C-1), 75.1 (CH_2_, OBn), 74.6 (CH, C-4), 73.4 (CH_2_, OBn), 71.5 (CH_2_, OBn), 69.4 (CH_2_, C-6), 69.2 (CH, C-2), 27.2 (CH_2_, C-1’ or C-2’), 24.4 (CH_2_, C-1’ or C-2’), 21.0 ppm (CH_3_, OAc). IR (CHCl_3_): ν = 3033, 2951, 2866, 1792, 1737, 1237, 1120 cm^–1^. MS (ESI) *m*/*z* (%) = 702 (100) [M + Na]^+^. HRMS (ESI) *m*/*z*: [M + Na]^+^ calcd for C_40_H_41_NNaO_9_ 702.2679; found 702.2675. Anal. calcd for C_40_H_41_NO_9_: C, 70.68; H, 6.08; N, 2.06. Found: C, 70.77; H, 6.05; N, 2.10.

#### 3-*C*-(2-*O*-Acetyl-3,4-di-*O*-benzyl-α-l-fucopyranosyl)1-propoxyphthalimide (**9**)

Following the general procedure starting from phthalimide **77** (247.4 mg, 0.46 mmol) and purification by column chromatography (hexanes–EtOAc, 9:1 to7:3), product **9** (153.6 mg, 0.27 mmol, 58%) was obtained as a colorless oil: [α]_D_ = −21.6 (*c =* 0.74, CHCl_3_). ^1^H NMR (500 MHz, CDCl_3_, simulated coupling constants using DAISY) δ_H_ 7.84–7.81 (m, 2H, Ar), 7.74–7.73 (m, 2H, Ar), 7.39–7.25 (m, 10H, Ar), 5.16 (dd, *J* = 5.9, 3.0 Hz, 1H, 2-H), 4.75 (d, *J* = 12.0 Hz, 1H, OBn), 4.70 (d, *J* = 12.0 Hz, 1H, OBn), 4.66 (d, *J* = 12.0 Hz, 1H, OBn), 4.55 (d, *J* = 12.0 Hz, 1H, OBn), 4.22 (dd, *J* = 5.0, 5.0 Hz, 2H, 3’-H_2_), 4.15 (ddd, *J* = 9.4, 4.1, 3.0 Hz, 1H, 1-H), 4.06 (dddd, *J* = 6.7, 6.7, 6.7, 4.5 Hz, 1H, 5-H), 3.82 (dd, *J* = 5.9, 3.2 Hz, 1H, 3-H), 3.74 (dd, *J* = 4.5, 3.2 Hz, 1H, 4-H), 2.08 (s, 3H, OAc), 1.91 (m, 1H, 2’-H_b_), 1.81–1.74 (m, 2H, 1’-H_b_, 2’-H_a_), 1.66 (m, 1H, 1’-H_a_), 1.36 ppm (d, *J* = 6.7 Hz, 3H, 6-H_3_). ^13^C{^1^H} NMR (125.7 MHz, CDCl_3_) δ_C_ 170.3 (C, OAc). 163.6 (2 × C, CO), 138.4 (C, Ar), 138.3 (C, Ar), 134.4 (2 × CH, Ar), 128.9 (2 × C, Ar), 128.28 (2 × CH, Ar), 128.26 (2 × CH, Ar), 127.7 (2 × CH, Ar), 127.6 (CH, Ar), 127.51 (CH, Ar), 127.46 (2 × CH, Ar), 123.4 (2 × CH, Ar), 78.1 (CH_2_, C-3’), 75.6 (CH, C-3), 74.6 (CH, C-4), 72.9 (CH_2_, OBn), 72.2 (CH_2_, OBn), 71.4 (CH, C-2), 69.5 (CH, C-5), 67.6 (CH, C-1), 24.9 (CH_2_, C-1’), 24.7 (CH_2_, C-2’), 21.0 (CH_3_, OAc), 14.6 ppm (CH_3_, C-6). IR (CHCl_3_): ν = 3029, 1791, 1734, 1214 cm^–1^. MS (ESI) *m*/*z* (%) = 596 (100) [M + Na]^+^. HRMS (ESI) *m*/*z*: [M + Na]^+^ calcd for C_33_H_35_NNaO_8_ 596.2260; found 596.2264. Anal. calcd for C_33_H_35_NO_8_: C, 69.10; H, 6.15; N, 2.44. Found: C, 69.25; H, 6.41; N, 2.58.

### General Procedure to Give Diphenoxyphosphoryl Derivatives **2**, **4**, **6**, **8**, **10**, and **15**

The phthalimide (1 mmol) was dissolved in dry CH_2_Cl_2_ (7.5 mL) under a N_2_ atmosphere. ClPO(OPh)_2_ (1 mL, 4.7 mmol) and DMAP (580 mg, 4.75 mmol) were added at 0 °C, and after 5 min, the mixture was stirred at room temperature for 2 h. The reaction was quenched with a saturated aqueous NH_4_Cl solution and extracted with CH_2_Cl_2_. The organic extracts were dried over Na_2_SO_4_ and concentrated under reduced pressure. Column chromatography of the residue (hexanes–EtOAc) gave the phosphatyl precursor.

#### 3-*C*-(3,4,6-Tri-*O*-benzyl-2-*O*-diphenoxyphosphoryl-α-d-glucopyranosyl)1-propoxyphthalimide (**2**)

Following the general procedure starting from phthalimide **64** (129.8 mg, 0.20 mmol) and purification by column chromatography (hexanes–EtOAc, 75:25), product **2** (132.6 mg, 0.15 mmol, 76%) was obtained as a colorless oil: [α]_D_ = +40.5 (*c =* 0.44, CHCl_3_). ^1^H NMR (500 MHz, CDCl_3_) δ_H_ 7.83–7.81 (m, 2H, Ar), 7.74–7.72 (m, 2H, Ar), 7.30–7.10 (m, 25H, Ar), 4.83 (d, *J* = 11.0 Hz, 1H, OBn), 4.80 (ddd, *J* = 10.5, 5.5 Hz, ^3^*J*_PH_ = 8.6 Hz, 1H, 2-H), 4.75 (d, *J* = 11.0 Hz, 1H, OBn), 4.74 (d, *J* = 11.1 Hz, 1H, OBn), 4.60 (d, *J* = 12.0 Hz, 1H, OBn), 4.46 (d, *J* = 11.0 Hz, 1H, OBn), 4.45 (d, *J* = 12.0 Hz, 1H, OBn), 4.20–4.15 (m, 2H, 3’-H_2_), 4.12 (ddd, *J* = 9.4, 7.2, 5.5 Hz, 1H, 1-H), 3.91 (ddd, *J* = 10.5, 5.7 Hz, ^4^*J*_PH_ = 2.9 Hz, 1H, 3-H), 3.71–3.64 (m, 4H, 4-H, 5-H, 6-H_2_), 1.97 (m, 1H, 1’-H_b_ or 2’-H_b_), 1.90 (m, 1H, 1’-H_b_ or 2’-H_b_), 1.72 (m, 1H, 1’-H_a_ or 2’-H_a_), 1.63 ppm (m, 1H, 1’-H_a_ or 2’-H_a_). ^13^C{^1^H} NMR (125.7 MHz, CDCl_3_) δ_C_ 163.5 (2 × C, CO). 150.5 (d, ^2^*J*_PC_ = 7.0 Hz, C, Ar), 150.4 (d, ^2^*J*_PC_ = 6.4 Hz, C, Ar), 138.1 (C, Ar), 138.0 (C, Ar), 137.9 (C, Ar), 134.4 (2 × CH, Ar), 129.8 (2 × CH, Ar), 129.7 (2 × CH, Ar), 128.9 (2 × C, Ar), 128.3 (4 × CH, Ar), 128.2 (2 × CH, Ar), 127.8 (6 × CH, Ar), 127.7 (CH, Ar), 127.6 (CH, Ar), 127.5 (CH, Ar), 125.4 (CH, Ar), 125.2 (CH, Ar), 123.4 (2 × CH, Ar), 120.22 (CH, Ar), 120.18 (CH, Ar), 119.99 (CH, Ar), 119.95 (CH, Ar), 80.5 (d, ^3^*J*_PC_ = 6.3 Hz, CH, C-3), 78.4 (d, ^2^*J*_PC_ = 7.4 Hz, CH, C-2), 77.9 (CH_2_, C-3’), 77.8 (CH, C-1), 75.1 (CH_2_, OBn), 74.8 (CH_2_, OBn), 73.7 (CH, C-4 or C-5), 73.5 (CH_2_, OBn), 71.4 (CH, C-4 or C-5), 68.9 (CH_2_, C-6), 24.3 (CH_2_, C-1’ or C-2’), 21.1 ppm (CH_2_, C-1’ or C-2’). IR (CHCl_3_): ν = 3021, 2946, 1790, 1734, 1213 cm^–1^. MS (ESI) *m*/*z* (%) = 892 (100) [M + Na]^+^. HRMS (ESI) *m*/*z*: [M + Na]^+^ calcd for C_50_H_48_NNaO_11_P 892.2863; found 892.2856. Anal. calcd for C_50_H_48_NO_11_P: C, 69.04; H; 5.56; N, 1.61. Found: C, 69.39; H, 5.74; N, 1.71.

#### 3-*C*-(3,4,6-Tri-*O*-benzyl-2-*O*-diphenoxyphosphoryl-β-d-glucopyranosyl)1-propoxyphthalimide (**4**)

Following the general procedure starting from phthalimide **67** (327 mg, 0.51 mmol) and purification by column chromatography (hexanes–EtOAc, 7:3), product **4** (224 mg, 0.26 mmol, 51%) was obtained as a colorless oil: [α]_D_ = +9.9 (*c =* 0.83, CHCl_3_). ^1^H NMR (500 MHz, CDCl_3_, simulated coupling constants using DAISY) δ_H_ 7.83–7.80 (m, 2H, Ar), 7.74–7.70 (m, 2H, Ar), 7.30–7.06 (m, 25H, Ar), 4.88 (d, *J* = 10.8 Hz, 1H, OBn), 4.83 (d, *J* = 10.8 Hz, 1H, OBn), 4.74 (d, *J* = 11.1 Hz, 1H, OBn), 4.58 (d, *J* = 12.3 Hz, 1H, OBn), 4.54 (d, *J* = 10.7 Hz, 1H, OBn), 4.49 (d, *J* = 12.3 Hz, 1H, OBn), 4.43 (ddd, *J* = 9.5, 8.9 Hz, ^3^*J*_PH_ = 8.2 Hz, 1H, 2-H), 4.13 (ddd, *J* = 8.8, 7.6, 6.3 Hz, 1H, 3’-H_b_), 4.04 (ddd, *J* = 8.8, 7.6, 6.6 Hz, 1H, 3’-H_a_), 3.78 (dd, *J* = 9.2, 9.2 Hz, 1H, 3-H), 3.72–3.66 (m, 2H, 6-H_2_), 3.70 (dd, *J* = 9.5, 9.3 Hz, 1H, 4-H), 3.48 (dddd, *J* = 9.5, 8.2, 8.1, 2.5 Hz, 1H, 1-H), 3.45 (ddd, *J* = 9.5, 3.8, 1.8 Hz, 1H, 5-H), 2.00 (m, 1H, 2’-H_b_), 1.89 (m, 1H, 2’-H_a_), 1.83 (m, 1H, 1’-H_b_), 1.57 ppm (m, 1H, 1’-H_a_). ^13^C{^1^H} NMR (100.6 MHz, CDCl_3_) δ_C_ 163.4 (2 × C, CO), 150.6 (d, ^2^*J*_PC_ = 7.1 Hz, C, Ar), 150.5 (d, ^2^*J*_PC_ = 7.1 Hz, C, Ar), 138.3 (C, Ar), 138.1 (C, Ar), 138.0 (C, Ar), 134.3 (2 × CH, Ar), 129.6 (2 × CH, Ar), 129.5 (2 × CH, Ar), 129.0 (2 × C, Ar), 128.28 (2 × CH, Ar), 128.26 (2 × CH, Ar), 128.0 (2 × CH, Ar), 127.7 (2 × CH, Ar), 127.6 (3 × CH, Ar), 127.5 (3 × CH, Ar), 127.2 (CH, Ar), 125.3 (CH, Ar), 125.0 (CH, Ar), 123.3 (2 × CH, Ar), 120.4 (CH, Ar), 120.3 (CH, Ar), 120.1 (CH, Ar), 120.0 (CH, Ar), 84.7 (d, ^3^*J*_PC_ = 2.1 Hz, CH, C-3), 80.6 (d, ^2^*J*_PC_ = 7.7 Hz, CH, C-2), 79.0 (CH, C-4), 78.5 (CH, C-5), 78.0 (CH_2_, C-3’), 77.8 (d, ^3^*J*_PC_ = 4.9 Hz, CH, C-1), 75.0 (CH_2_, OBn), 74.8 (CH_2_, OBn), 73.4 (CH_2_, OBn), 68.8 (CH_2_, C-6), 27.2 (CH_2_, C-1’), 23.9 ppm (CH_2_, C-2’). IR (CHCl_3_): ν = 3013, 2870, 1776, 1736, 1240, 1090 cm^–1^. MS (ESI) *m*/*z* (%) = 892 (100) [M + Na]^+^. HRMS (ESI) *m*/*z*: [M + Na]^+^ calcd for C_50_H_48_NNaO_11_P 892.2863; found 892.2879. Anal. calcd for C_50_H_48_NO_11_P: C, 69.04; H, 5.56; N, 1.61. Found: C, 69.15; H, 5.64; N, 1.99.

#### 3-*C*-(3,4,6-Tri-*O*-benzyl-2-*O*-diphenoxyphosphoryl-α-d-mannopyranosyl)1-propoxyphthalimide (**6**)

Following the general procedure starting from phthalimide **70** (109.2 mg, 0.17 mmol) and purification by column chromatography (hexanes–EtOAc, 65:35), product **6** (110.6 mg, 0.13 mmol, 74%) was obtained as a colorless oil: [α]_D_ = −10.7 (*c =* 0.54, CHCl_3_). ^1^H NMR (500 MHz, CDCl_3_) δ_H_ 7.80–7.79 (m, 2H, Ar), 7.74–7.70 (m, 2H, Ar), 7.34–7.06 (m, 25H, Ar), 4.96 (ddd, *J* = 2.9, 2.9 Hz, ^3^*J*_PH_ = 8.2 Hz, 1H, 2-H), 4.81 (d, *J* = 11.1 Hz, 1H, OBn), 4.67 (d, *J* = 11.0 Hz, 1H, OBn), 4.57 (d, *J* = 12.0 Hz, 1H, OBn), 4.53 (d, *J* = 11.4 Hz, 1H, OBn), 4.49 (d, *J* = 12.3 Hz, 1H, OBn), 4.40 (d, *J* = 11.0 Hz, 1H, OBn), 4.21–4.16 (m, 2H, 3’-H_2_), 4.07 (ddd, *J* = 9.8, 3.8, 3.8 Hz, 1H, 1-H), 3.92 (m, 1H, 3-H), 3.74–3.66 (m, 4H, 4-H, 5-H, 6-H_2_), 1.94–1.85 (m, 2H, 1’-H_b_, 2’-H_b_), 1.81–1.70 ppm (m, 2H, 1’-H_a_, 2’-H_a_). ^13^C{^1^H} NMR (125.7 MHz, CDCl_3_) δ_C_ 163.5 (2 × C, CO), 150.7 (d, ^2^*J*_PC_ = 7.4 Hz, C, Ar), 150.5 (d, ^2^*J*_PC_ = 7.4 Hz, C, Ar), 138.3 (C, Ar), 138.1 (C, Ar), 137.7 (C, Ar), 134.3 (2 × CH, Ar), 129.7 (2 × CH, Ar), 129.5 (2 × CH, Ar), 128.9 (2 × C, Ar), 128.3 (2 × CH, Ar), 128.24 (4 × CH, Ar), 128.19 (2 × CH, Ar), 127.9 (2 × CH, Ar), 127.64 (2 × CH, Ar), 127.59 (2 × CH, Ar), 127.4 (CH, Ar), 125.2 (CH, Ar), 125.0 (CH, Ar), 123.4 (2 × CH, Ar), 120.3 (CH, Ar), 120.24 (CH, Ar), 120.18 (CH, Ar), 120.1 (CH, Ar), 77.8 (d, ^3^*J*_PC_ = 3.2 Hz, CH, C-3), 77.7 (CH_2_, C-3’), 77.5 (d, ^2^*J*_PC_ = 6.4 Hz, CH, C-2), 74.9 (br s, CH, C-1), 74.5 (CH_2_, OBn), 74.3 (CH, C-4 or C-5), 73.3 (CH_2_, OBn), 73.0 (CH, C-4 or C-5), 72.1 (CH_2_, OBn), 69.1 (CH_2_, C-6), 24.8 (CH_2_, C-1’ or C-2’), 24.6 ppm (CH_2_, C-1’ or C-2’). IR (CHCl_3_): ν = 2929, 2858, 1793, 1737, 1491, 1191 cm^–1^. MS (ESI) *m*/*z* (%) = 892 (100) [M + Na]^+^. HRMS (ESI) *m*/*z*: [M + Na]^+^ calcd for C_50_H_48_NNaO_11_P 892.2863; found 892.2864. Anal. calcd for C_50_H_48_NO_11_P: C, 69.04; H, 5.56; N, 1.61. Found: C, 68.91; H, 5.95; N, 1.91.

#### 3-*C*-(3,4,6-Tri-*O*-benzyl-2-*O*-diphenoxyphosphoryl-β-d-mannopyranosyl)1-propoxyphthalimide (**8**)

Following the general procedure starting from phthalimide **73** (220 mg, 0.34 mmol) and purification by column chromatography (hexanes–EtOAc, 7:3), product **8** (242.7 mg, 0.28 mmol, 81%) was obtained as a colorless oil: [α]_D_ = −16.6 (*c =* 0.53, CHCl_3_). ^1^H NMR (500 MHz, CDCl_3_) δ_H_ 7.83–7.80 (m, 2H, Ar), 7.76–7.72 (m, 2H, Ar), 7.39–7.12 (m, 25H, Ar), 5.09 (br dd, *J* = 1.9 Hz, ^3^*J*_PH_ = 8.8 Hz, 1H, 2-H), 4.94 (d, *J* = 11.4 Hz, 1H, OBn), 4.63 (d, *J* = 10.7 Hz, 1H, OBn), 4.62 (d, *J* = 12.9 Hz, 1H, OBn), 4.52 (d, *J* = 11.7 Hz, 1H, OBn), 4.52 (d, *J* = 11.7 Hz, 1H, OBn), 4.31 (d, *J* = 10.8 Hz, 1H, OBn), 4.08 (dd, *J* = 6.4, 6.4 Hz, 2H, 3’-H_2_), 3.68 (br d, *J* = 3.2 Hz, 2H, 6-H_2_), 3.63 (m, 2H, 3-H, 4-H), 3.56 (m, 1H, 1-H), 3.42 (m, 1H, 5-H), 1.86 (m, 1H, 2’-H_b_), 1.81–1.73 (m, 2H, 1’-H_b_, 2’-H_a_), 1.68 ppm (m, 1H, 1’-H_a_). ^13^C{^1^H} NMR (100.6 MHz, CDCl_3_) δ_C_ 163.5 (2 × C, CO), 150.9 (d, ^2^*J*_PC_ = 7.8 Hz, C, Ar), 150.7 (d, ^2^*J*_PC_ = 7.0 Hz, C, Ar), 138.5 (C, Ar), 138.4 (C, Ar), 137.8 (C, Ar), 134.4 (2 × CH, Ar), 129.7 (2 × CH, Ar), 129.3 (2 × CH, Ar), 129.0 (2 × C, Ar), 128.4 (2 × CH, Ar), 128.3 (4 × CH, Ar), 128.2 (2 × CH, Ar), 127.9 (2 × CH, Ar), 127.7 (2 × CH, Ar), 127.6 (CH, Ar), 127.5 (CH, Ar), 127.4 (CH, Ar), 125.2 (CH, Ar), 124.7 (CH, Ar), 123.4 (2 × CH, Ar), 120.4 (CH, Ar), 120.3 (CH, Ar), 120.23 (CH, Ar), 120.18 (CH, Ar), 82.0 (CH, C-3), 79.5 (CH, C-5), 77.9 (CH_2_, C-3’), 77.0 (CH, C-2), 76.4 (d, ^3^*J*_PC_ = 5.6 Hz, CH, C-1), 75.1 (CH_2_, OBn), 74.2 (CH, C-4), 73.4 (CH_2_, OBn), 71.8 (CH_2_, OBn), 69.3 (CH_2_, C-6), 27.2 (CH_2_, C-1’), 24.3 ppm (CH_2_, C-2’). IR (CHCl_3_): ν = 3013, 2869, 1789, 1733, 1193 cm^–1^. MS (ESI) *m*/*z* (%) = 892 (100) [M + Na]^+^. HRMS (ESI) *m*/*z*: [M + Na]^+^ calcd for C_50_H_48_NNaO_11_P 892.2863; found 892.2861. Anal. calcd for C_50_H_48_NO_11_P: C, 69.04; H, 5.56; N, 1.61. Found: C, 68.94; H, 5.83; N, 1.67.

#### 3-*C*-(3,4-Di-*O*-benzyl-2-*O*-diphenoxyphosphoryl-α-l-fucopyranosyl)1-propoxyphthalimide (**10**)

Following the general procedure starting from phthalimide **77** (253 mg, 0.48 mmol) and purification by column chromatography (hexanes–EtOAc, 7:3), product **10** (205 mg, 0.27 mmol, 56%) was obtained as a colorless oil: [α]_D_ = −19.6 (*c =* 0.43, CHCl_3_). ^1^H NMR (500 MHz, CDCl_3_, simulated ring coupling constants using DAISY) δ_H_ 7.83–7.80 (m, 2H, Ar), 7.75–7.73 (m, 2H, Ar), 7.37–7.13 (m, 20H, Ar), 4.91 (ddd, *J* = 6.3, 3.3 Hz, ^3^*J*_PH_ = 7.5 Hz, 1H, 2-H), 4.69 (d, *J* = 12.0 Hz, 1H, OBn), 4.66 (d, *J* = 12.0 Hz, 1H, OBn), 4.57 (d, *J* = 11.7 Hz, 1H, OBn), 4.42 (d, *J* = 11.7 Hz, 1H, OBn), 4.17–4.09 (m, 3H, 1-H, 3’-H_2_), 4.01 (dddd, *J* = 6.7, 6.7, 6.7, 4.0 Hz, 1H, 5-H), 3.95 (dd, *J* = 6.3, 3.1 Hz, 1H, 3-H), 3.72 (dd, *J* = 4.1, 3.1 Hz, 1H, 4-H), 1.90–1.76 (m, 2H, 1’-H_2_ or 2’-H_2_), 1.74–1.60 (m, 2H, 1’-H_2_ or 2’-H_2_), 1.31 ppm (d, *J* = 6.7 Hz, 3H, 6-H_3_). ^13^C{^1^H} NMR (125.7 MHz, CDCl_3_) δ_C_ 163.5 (2 × C, CO), 150.52 (d, ^2^*J*_PC_ = 7.4 Hz, C, Ar), 150.48 (d, ^2^*J*_PC_ = 7.4 Hz, C, Ar), 138.3 (C, Ar), 138.1 (C, Ar), 134.4 (2 × CH, Ar), 129.7 (4 × CH, Ar), 128.9 (2 × C, Ar), 128.3 (2 × CH, Ar), 128.2 (2 × CH, Ar), 127.6 (4 × CH, Ar), 127.5 (2 × CH, Ar), 125.4 (CH, Ar), 125.3 (CH, Ar), 123.4 (2 × CH, Ar), 120.2 (CH, Ar), 120.12 (CH, Ar), 120.08 (CH, Ar), 120.0 (CH, Ar), 78.1 (CH_2_, C-3’), 77.2 (CH, C-2), 76.1 (CH, C-3), 75.0 (CH, C-4), 73.1 (CH_2_, OBn), 72.6 (CH_2_, OBn), 69.2 (CH, C-5), 68.9 (CH, C-1), 24.7 (CH_2_, C-1’), 24.1 (CH_2_, C-2’), 14.8 ppm (CH_3_, C-6). IR (CHCl_3_): ν = 3027, 1791, 1733, 1214 cm^–1^. MS (ESI) *m*/*z* (%) = 786 (100) [M + Na]^+^. HRMS (ESI) *m*/*z*: [M + Na]^+^ calcd for C_43_H_42_NNaO_10_P 786.2444; found 786.2448. Anal. calcd for C_43_H_42_NO_10_P: C, 67.62; H, 5.54; N, 1.83. Found: C, 67.81; H, 5.88; N, 1.48.

#### 3-*C*-(2-*O*-Diphenoxyphosphoryl-3,5-di-*O*-1,1,3,3-tetraisopropyldisiloxanyl-α-d-ribofuranosyl)1-propoxyphthalimide (**15**)

Following the general procedure starting from phthalimide **88** (351 mg, 0.61 mmol) and purification by column chromatography (hexanes–EtOAc, 8:2 to 7:3), product **15** (443 mg, 0.55 mmol, 90%) was obtained as a colorless oil: [α]_D_ = +15.8 (*c =* 0.83, CHCl_3_). ^1^H NMR (500 MHz, CDCl_3_, simulated ring coupling constants using DAISY) δ_H_ 7.83–7.73 (m, 4H, Ar), 7.32–7.12 (m, 10H, Ar), 5.15 (ddd, *J* = 3.9, 2.9 Hz, ^3^*J*_PH_ = 7.9 Hz, 1H, 2-H), 4.47 (ddd, *J* = 9.1, 3.9 Hz, ^4^*J*_PH_ = 1.5 Hz, 1H, 3-H), 4.19 (m, 1H, 1-H), 4.08 (br dd, *J* = 6.3, 6.3 Hz, 2H, 3’-H_2_), 3.99 (dd, *J* = 12.8, 3.1 Hz, 1H, 5-H_b_), 3.94–3.90 (m, 2H, 4-H, 5-H_a_), 1.88 (m, 1H, 2’-H_b_), 1.71 (m, 1H, 2’-H_a_), 1.65–1.57 (m, 2H, 1’-H_2_), 1.10–0.97 ppm (m, 28H, *^i^*Pr). ^13^C{^1^H} NMR (125.7 MHz, CDCl_3_) δ_C_ 163.6 (2 × C, CO), 150.9 (d, ^2^*J*_PC_ = 7.4 Hz, C, Ar), 150.6 (d, ^2^*J*_PC_ = 6.4 Hz, C, Ar), 134.4 (2 × CH, Ar), 129.7 (2 × CH, Ar), 129.6 (2 × CH, Ar), 129.0 (2 × C, Ar), 125.2 (CH, Ar), 125.0 (CH, Ar), 123.4 (2 × CH, Ar), 120.23 (CH, Ar), 120.18 (CH, Ar), 120.05 (CH, Ar), 120.00 (CH, Ar), 81.5 (d, ^2^*J*_PC_ = 6.4 Hz, CH, C-2), 79.6 (CH, C-4), 78.9 (d, ^3^*J*_PC_ = 5.3 Hz, CH, C-1), 77.9 (CH_2_, C-3’), 71.7 (CH, C-3), 61.0 (CH_2_, C-5), 26.1 (CH_2_, C-1’), 24.6 (CH_2_, C-2’), 17.4 (CH_3_, *^i^*Pr), 17.3 (CH_3_, *^i^*Pr), 17.28 (CH_3_, *^i^*Pr), 17.26 (CH_3_, *^i^*Pr), 17.04 (CH_3_, *^i^*Pr), 17.01 (CH_3_, *^i^*Pr), 16.8 (CH_3_, *^i^*Pr), 16.7 (CH_3_, *^i^*Pr), 13.5 (CH, *^i^*Pr), 13.1 (CH, *^i^*Pr), 12.7 (CH, *^i^*Pr), 12.4 ppm (CH, *^i^*Pr). IR (CHCl_3_): ν = 2948, 1791, 1733, 1490, 1212 cm^–1^. MS (ESI) *m*/*z* (%) = 834 (100) [M + Na]^+^. HRMS (ESI) *m*/*z*: [M + Na]^+^ calcd for C_40_H_54_NNaO_11_PSi_2_ 834.2871; found 834.2874. Anal. calcd for C_40_H_54_NO_11_PSi_2_: C, 59.17; H, 6.70; N, 1.72. Found: C, 59.00; H, 6.94; N, 1.67.

#### 3-*C*-(2-*O*-Diphenoxyphosphoryl-β-d-arabinopyranosyl)1-propoxyphthalimide (**12**)

Compound **11** (227 mg, 0.35 mmol) was dissolved in CH_2_Cl_2_ (1.6 mL), and TFA/H_2_O (230 μL, 80%) was dropwise added at 0 °C. After 1 h, the mixture was neutralized with a saturated aqueous solution of NaHCO_3_ and extracted with CH_2_Cl_2_. The organic extracts were dried over Na_2_SO_4_ and concentrated under reduced pressure. Column chromatography of the residue (hexanes–EtOAc, 3:7) gave **12** (161.7 mg, 0.28 mmol, 81%) as a colorless oil: [α]_D_ = +4.6 (*c =* 0.56, CHCl_3_). ^1^H NMR (500 MHz, CDCl_3_) δ_H_ 7.83–7.80 (m, 2H, Ar), 7.75–7.72 (m, 2H, Ar), 7.35–7.17 (m, 10H, Ar), 4.60 (ddd, *J* = 4.1, 1.3 Hz, ^3^*J*_PH_ = 8.2 Hz, 1H, 2-H), 4.16–4.05 (m, 3H, 3-H, 3’-H_2_), 3.86–3.81 (m, 2H, 1-H, 4-H), 3.74 (dd, *J* = 11.1, 5.1 Hz, 1H, 5-H_b_), 3.48 (dd, *J* = 10.7, 10.7 Hz, 1H, 5-H_a_), 2.75 (br d, *J* = 8.2 Hz, 1H, OH), 2.01 (br s, 1H, OH), 1.86 (m, 1H, 2’-H_b_), 1.75 (m, 1H, 2’-H_a_), 1.68 (m, 1H, 1’-H_b_), 1.58 ppm (m, 1H, 1’-H_a_). ^13^C{^1^H} NMR (125.7 MHz, CDCl_3_) δ_C_ 163.6 (2 × C, CO), 150.3 (d, ^2^*J*_PC_ = 7.4 Hz, C, Ar), 150.2 (d, ^2^*J*_PC_ = 8.4 Hz, C, Ar), 134.4 (2 × CH, Ar), 129.9 (4 × CH, Ar), 128.9 (2 × C, Ar), 125.7 (CH, Ar), 125.6 (CH, Ar), 123.5 (2 × CH, Ar), 120.09 (CH, Ar), 120.06 (CH, Ar), 120.04 (CH, Ar), 120.00 (CH, Ar), 79.1 (d, ^2^*J*_PC_ = 6.4 Hz, CH, C-2), 78.0 (CH_2_, C-3’), 72.4 (d, ^3^*J*_PC_ = 6.4 Hz, CH, C-1), 68.0 (CH, C-3), 65.9 (CH_2_, C-5), 64.2 (CH, C-4), 26.0 (CH_2_, C-1’), 24.5 ppm (CH_2_, C-2’). IR (CHCl_3_): ν = 3688, 3557, 3393, 3026, 1790, 1733, 1211 cm^–1^. MS (ESI) *m*/*z* (%) = 592 (100) [M + Na]^+^. HRMS (ESI) *m*/*z*: [M + Na]^+^ calcd for C_28_H_28_NNaO_10_P 592.1349; found 592.1341. Anal. calcd for C_28_H_28_NO_10_P: C, 59.05; H, 4.96; N, 2.46. Found: C, 59.26; H, 4.99; N, 2.48.

### General Procedure to Give Acetyl Derivatives **90** and **97**

The corresponding alcohol (1 mmol) was dissolved in dry pyridine (3.7 mL), and Ac_2_O (1.2 mL) and DMAP (13.7 mg, 0.11 mmol) were added. The reaction was stirred at room temperature for 0.5 h, and then it was evaporated in a high vacuum rotovap, quenched with an aqueous solution of HCl 10%, and extracted with CH_2_Cl_2_. The combined organic extracts were dried over Na_2_SO_4_ and concentrated under reduced pressure. Column chromatography of the residue (hexanes–EtOAc) gave the acetyl derivative.

#### Methyl 4-*O*-Acetyl-6-*O*-*tert*-butyldiphenylsilyl-2,3-di-*O*-methyl-α-d-glucopyranoside (**90**)

Following the general procedure starting from methyl 6-*O*-*tert*-butyldiphenylsilyl-2,3-di-*O*-methyl-α-d-glucopyranoside (**89**)^64^ (119.7 mg, 0.27 mmol) and purification by column chromatography (hexanes–EtOAc, 7:3), product **90** (122.7 mg, 0.25 mmol, 94%) was obtained as a colorless oil: [α]_D_ = +65.3 (*c* = 0.80, CHCl_3_). ^1^H NMR (400 MHz, CDCl_3_) δ_H_ 7.69–7.67 ppm (m, 4H, Ar), 7.45–7.36 (m, 6H, Ar), 4.87 (d, *J* = 3.5 Hz, 1H, 1-H), 4.87 (dd, *J* = 9.5, 9.5 Hz, 1H, 4-H), 3.75 (ddd, *J* = 10.1, 5.7, 2.5 Hz, 1H, 5-H), 3.71–3.62 (m, 2H, 6-H_2_), 3.58 (dd, *J* = 9.5, 9.5 Hz, 1H, 3-H), 3.54 (s, 3H, OMe), 3.51 (s, 3H, OMe), 3.47 (s, 3H, OMe), 3.30 (dd, *J* = 9.6, 3.7 Hz, 1H, 2-H), 1.95 (s, 3H, OAc), 1.05 ppm (s, 9H, *^t^*Bu). ^13^C{^1^H} NMR (100.6 MHz, CDCl_3_) δ_C_ 169.5 (C, OAc), 135.7 (2 × CH, Ar), 135.6 (2 × CH, Ar), 133.4 (C, Ar), 133.3 (C, Ar), 129.62 (CH, Ar), 129.60 (CH, Ar), 127.62 (2 × CH, Ar), 127.59 (2 × CH, Ar), 97.3 (CH, C-1), 81.5 (CH, C-2 or C-5), 81.1 (CH, C-2 or C-5), 70.6 (CH, C-3 or C-4), 70.5 (CH, C-3 or C-4), 63.2 (CH_2_, C-6), 60.6 (CH_3_, OMe), 59.1 (CH_3_, OMe), 55.1 (CH_3_, OMe), 26.7 (3 × CH_3_, DPS), 20.8 (CH_3_, OAc), 19.2 ppm (C, DPS). IR (CHCl_3_): ν = 2932, 1748, 1233, 1046 cm^–1^. MS (ESI) *m*/*z* (%) = 525 (100) [M + Na]^+^. HRMS (ESI) *m*/*z*: [M + Na]^+^ calcd for C_27_H_38_NaO_7_Si 525.2285; found 525.2272. Anal. calcd for C_27_H_38_O_7_Si: C, 64.51; H, 7.62. Found: C, 64.45; H, 7.85.

#### Methyl 4-*O*-Acetyl-6-*O*-*tert*-butyldiphenylsilyl-2,3-di-*O*-methyl-α-d-galactopyranoside (**97**)

Following the general procedure starting from methyl 6-*O*-*tert*-butyldiphenylsilyl-2,3-di-*O*-methyl-α-d-galactopyranoside (**96**)^64^ (2.15 g, 4.80 mmol) and purification by column chromatography (hexanes–EtOAc, 7:3), product **97** (2.19 g, 4.36 mmol, 91%) was obtained as a colorless oil: [α]_D_ = +65.5 (*c* = 1.45, CHCl_3_). ^1^H NMR (400 MHz, CDCl_3_) δ_H_ 7.69–7.64 (m, 4H, Ar), 7.45–7.36 (m, 6H, Ar), 5.58 (dd, *J* = 3.2, 1.0 Hz, 1H, 4-H), 4.88 (d, *J* = 3.6 Hz, 1H, 1-H), 3.93 (ddd, *J* = 6.9, 6.9, 0.9 Hz, 1H, 5-H), 3.69 (dd, *J* = 10.2, 6.3 Hz, 1H, 6-H_b_), 3.64 (dd, *J* = 10.4, 7.0 Hz, 1H, 6-H_a_), 3.60 (dd, *J* = 10.1, 3.4 Hz, 1H, 3-H), 3.51 (s, 3H, OMe), 3.50 (dd, *J* = 10.1, 6.4 Hz, 1H, 2-H), 3.43 (s, 3H, OMe), 3.40 (s, 3H, OMe), 2.03 (s, 3H, OAc), 1.06 ppm (s, 9H, *^t^*Bu). ^13^C{^1^H} NMR (100.6 MHz, CDCl_3_) δ_C_ 169.9 ppm (C, OAc), 135.5 (4 × CH, Ar), 133.2 (C, Ar), 133.1 (C, Ar), 129.7 (CH, Ar), 129.6 (CH, Ar), 127.7 (2 × CH, Ar), 127.6 (2 × CH, Ar), 97.7 (CH, C-1), 78.1 (CH, C-2 or C-5), 77.3 (CH, C-2 or C-5), 69.2 (CH, C-3 or C-4), 66.9 (CH, C-3 or C-4), 62.2 (CH_2_, C-6), 59.0 (CH_3_, OMe), 57.6 (CH_3_, OMe), 55.2 (CH_3_, OMe), 26.7 (3 × CH_3_, DPS), 20.6 (CH_3_, OAc), 19.0 ppm (C, DPS). IR (CHCl_3_): ν = 2934, 1741, 1239, 1106 cm^–1^. MS (ESI) *m*/*z* (%) = 525 (100) [M + Na]^+^. HRMS (ESI) *m*/*z*: [M + Na]^+^ calcd for C_27_H_38_NaO_7_Si 525.2285; found 525.2279. Anal. calcd for C_27_H_38_O_7_Si: C, 64.51; H, 7.62. Found: C, 64.72; H, 7.50.

### General Procedure to Give Allenyl Derivatives **91**, **98**, and **107**

The precursor (1 mmol) was dissolved in CH_3_CN (11 mL) under a N_2_ atmosphere, and freshly prepared propargyl trimethylsilane/Et_2_O^65^ 39% v/v (0.77 mL, 2 mmol) and TMSOTf (0.4 mL, 2.27 mmol) were dropwise added. The reaction was sonicated in an ultrasonic bath for 1.5–3 h, and then it was poured over a saturated aqueous solution of NaHCO_3_ and extracted with EtOAc. The organic phase was dried over Na_2_SO_4_, filtered, and evaporated. The crude was dissolved in DMF (2 mL), and DPSCl (176 μL, 38.68 mmol) and imidazole (102 mg, 22.42 mmol) were added. The reaction was stirred at room temperature for 2 h, evaporated under reduced high pressure, and purified by column chromatography (hexanes–EtOAc) to give the expected allenyl derivative.

#### *C*-(4-*O*-Acetyl-6-*O*-*tert*-butyldiphenylsilyl-2,3-di-*O*-methyl-α-d-glucopyranosyl)allene (**91**)

Following the general procedure starting from **90** (2.23 g, 4.55 mmol) and purification by column chromatography (hexanes–EtOAc, 9:1), product **91** (991.3 mg, 1.79 mmol, 39%) was obtained as a colorless oil: [α]_D_ = +91.4 (*c* = 0.80, CHCl_3_). ^1^H NMR (400 MHz, CDCl_3_) δ_H_ 7.70–7.66 (m, 4H, Ar), 7.44–7.35 (m, 6H, Ar), 5.38 (ddd, *J* = 6.7, 6.7, 6.7 Hz, 1H, 1’-H), 4.93 (dd, *J* = 9.2, 9.2 Hz, 1H, 3-H), 4.87 (dd, *J* = 6.7, 0.0 Hz, 1H, 3’-H_b_), 4.86 (dd, *J* = 6.7, 0.0 Hz, 1H, 3’-H_a_), 4.78 (m, 1H, 1-H), 3.85–3.80 (m, 2H, 4-H, 6-H_b_), 3.69–3.67 (m, 2H, 5-H, 6-H_a_), 3.51 (s, 3H, OMe), 3.48 (s, 3H, OMe), 3.43 (dd, *J* = 9.5, 6.3 Hz, 1H, 2-H), 1.96 (s, 3H, OAc), 1.06 ppm (s, 9H, *^t^*Bu). ^13^C{^1^H} NMR (100.6 MHz, CDCl_3_) δ_C_ 209.5 (C, C-2’), 169.5 (C, OAc), 135.7 (4 × CH, Ar), 133.6 (C, Ar), 133.4 (C, Ar), 129.6 (CH, Ar), 129.5 (CH, Ar), 127.57 (2 × CH, Ar), 127.55 (2 × CH, Ar), 85.5 (CH, C-1’), 81.0 (2 × CH, C-2, C-5), 76.7 (CH_2_, C-3’), 72.8 (CH, C-4), 71.1 (CH, C-1), 70.5 (CH, C-3), 63.6 (CH_2_, C-6), 60.0 (CH_3_, OMe), 58.4 (CH_3_, OMe), 26.8 (3 × CH_3_, DPS), 20.9 (CH_3_, OAc), 19.2 ppm (C, DPS). IR (CHCl_3_): ν = 3024, 2934, 1956, 1736, 1208 cm^–1^. MS (ESI) *m*/*z* (%) = 533 (100) [M + Na]^+^. HRMS (ESI) *m*/*z*: [M + Na]^+^ calcd for C_29_H_38_NaO_6_Si 533.2335; found 533.2333.

#### *C*-(4-*O*-Acetyl-6-*O*-*tert*-butyldiphenylsilyl-2,3-di-*O*-methyl-α-d-galactopyranosyl)allene (**98**)

Following the general procedure starting from **97** (1.91 g, 3.80 mmol) and purification by column chromatography (hexanes–EtOAc, 8:2), product **98** (1.27 g, 2.49 mmol, 59%) was obtained as a yellow oil: [α]_D_ = +101.0 (*c* = 0.81, CHCl_3_). ^1^H NMR (400 MHz, CDCl_3_) δ_H_ 7.68–7.63 (m, 4H, Ar), 7.45–7.35 (m, 6H, Ar), 5.67 (dd, *J* = 2.9, 1.2 Hz, 1H, 4-H), 5.33 (m, 1H, 1’-H), 4.85–4.79 (m, 3H, 1-H, 3’-H_2_), 3.98 (ddd, *J* = 7.6, 5.7, 1.5 Hz, 1H, 5-H), 3.67 (dd, *J* = 10.1, 5.8 Hz, 1H, 6-H_b_), 3.66 (dd, *J* = 10.5, 5.7 Hz, 1H, 2-H), 3.59 (dd, *J* = 9.8, 7.9 Hz, 1H, 6-H_a_), 3.47 (s, 3H, OMe), 3.46 (s, 3H, OMe), 3.40 (dd, *J* = 9.9, 3.6 Hz, 1H, 3-H), 2.06 (s, 3H, OAc), 1.06 ppm (s, 9H, *^t^*Bu). ^13^C{^1^H} NMR (100.6 MHz, CDCl_3_) δ_C_ 209.0 (C, C-2’). 169.8 (C, OAc), 135.60 (2 × CH, Ar), 135.56 (2 × CH, Ar), 133.4 (C, Ar), 133.2 (C, Ar), 129.72 (CH, Ar), 129.67 (CH, Ar), 127.7 (2 × CH, Ar), 127.6 (2 × CH, Ar), 85.2 (CH, C-1’), 78.8 (CH, C-5), 77.1 (CH, C-2), 76.9 (CH_2_, C-3’), 71.5 (CH, C-4), 71.0 (CH, C-1), 66.6 (CH, C-3), 61.8 (CH_2_, C-6), 58.5 (CH_3_, OMe), 57.7 (CH_3_, OMe), 26.7 (3 × CH_3_, DPS), 20.7 (CH_3_, OAc), 19.1 ppm (C, DPS). IR (CHCl_3_): ν = 3016, 2934, 1955, 1741, 1208 cm^–1^. MS (ESI) *m*/*z* (%) = 533 (100) [M + Na]^+^. HRMS (ESI) *m*/*z*: [M + Na]^+^ calcd for C_29_H_38_NaO_6_Si 533.2335; found 533.2328. Anal. calcd for C_29_H_38_O_6_Si: C, 68.20; H, 7.50. Found: C, 68.24; H, 7.70.

#### *C*-(2,3,4-Tri-*O*-acetyl-α-l-fucopyranosyl)allene (**107**)

Following the general procedure starting from peracetyl l-fucose (**106**) (877 mg, 2.64 mmol) and purification by column chromatography (hexanes–EtOAc, 8:2), product **107** (601.8 mg, 1.93 mmol, 73%) was obtained as a yellow oil: [α]_D_ = −170.0 (*c* = 0.92, CHCl_3_). ^1^H NMR (500 MHz, C_6_D_6_) δ_H_ 5.68 (dd, *J* = 11.0, 6.0 Hz, 1H, 2-H), 5.46 (dd, *J* = 11.0, 3.5 Hz, 1H, 3-H), 5.37 (dd, *J* = 3.5, 1.3 Hz, 1H, 4-H), 5.17 (ddd, *J* = 6.9, 6.9, 6.9 Hz, 1H, 1′-H), 5.04 (dddd, *J* = 6.1, 6.1, 3.2, 3.2 Hz, 1H, 1-H), 4.59 (ddd, *J* = 11.3, 6.6, 3.2 Hz, 1H, 3′-H_b_), 4.55 (ddd, *J* = 11.7, 6.9, 3.5 Hz, 1H, 3′-H_a_), 3.67 (dddd, *J* = 6.3, 6.3, 6.3, 1.0 Hz, 1H, 5-H), 1.75 (s, 3H, OAc), 1.65 (s, 3H, OAc), 1.59 (s, 3H, OAc), 0.97 ppm (d, *J* = 6.3 Hz, 3H, 6-H_3_). ^13^C{^1^H} NMR (125.7 MHz, C_6_D_6_) δ_C_ 209.8 (CH, C-2’), 170.2 (C, OAc), 169.8 (C, OAc), 169.4 (C, OAc), 85.4 (CH, C-1’), 76.6 (CH_2_, C-3’), 71.7 (CH, C-1), 71.5 (CH, C-4), 69.1 (CH, C-3), 68.4 (CH, C-2), 66.6 (CH, C-5), 20.4 (CH_3_, OAc), 20.2 (CH_3_, OAc), 20.0 (CH_3_, OAc), 16.3 ppm (CH_3_, C-6). IR (CHCl_3_): ν = 2989, 2944, 1957, 1750, 1373, 1230, 1062 cm^–1^. MS (ESI) *m*/*z* (%) = 335 (100) [M + Na]^+^. HRMS (ESI) *m*/*z*: [M + Na]^+^ calcd for C_15_H_20_NaO_7_ 335.1107; found 335.1101. Anal. calcd for C_15_H_20_O_7_: C, 57.69; H, 6.45. Found: C, 58.07; H, 6.79.

#### *C*-(2,3,4-Tri-*O*-acetyl-α-l-rhamnopyranosyl)allene (**101**) and *C*-(2,3,4-Tri-*O*-acetyl-β-l-rhamnopyranosyl)allene (**102**)

BF_3_•OEt_2_ (6.40 mL, 51.86 mmol) and TMSOTf (6.20 mL, 34.66 mmol) were dropwise added to a solution of peracetyl l-rhamnose (**100**) (6 g, 18.0 mmol) and fresly prepared propargyl trimethylsilane (5.11 mL, 34.28 mmol) in dry CH_3_CN (39 mL) at 0 °C, and the mixture was stirred at room temperature for 15 h. Then, it was poured over HCl 10% and extracted with EtOAc. The organic extracts were washed with saturated aqueous solutions of NaHCO_3_ and NaCl, dried over Na_2_SO_4_, and concentrated to dryness under reduced pressure. Column chromatography of the residue (hexanes–EtOAc, 95:5) gave **101** (3.80 g, 12.18 mmol, 67%) and **102** (1.40 g, 4.49 mmol, 25%) that could not be completely purified and was isolated as a mixture (1:1) with **101**. Compound **101**: colorless oil, [α]_D_ = −16.7 (*c* = 0.42, CHCl_3_). ^1^H NMR (500 MHz, CDCl_3_) δ_H_ 5.40 (dd, *J* = 3.3, 2.0 Hz, 1H, 2-H), 5.26 (dd, *J* = 9.8, 3.5 Hz, 1H, 3-H), 5.21 (ddd, *J* = 6.8, 6.8, 4.4 Hz, 1H, 1′-H), 5.07 (dd, *J* = 9.5, 9.5 Hz, 1H, 4-H), 5.05 (ddd, *J* = 12.3, 6.9, 4.1 Hz, 1H, 3′-H_b_), 4.99 (ddd, *J* = 11.4, 6.9, 4.4 Hz, 1H, 3′-H_a_), 4.57 (dddd, *J* = 4.1, 4.1, 4.1, 1.9 Hz, 1H, 1-H), 3.84 (dddd, *J* = 9.5, 6.2, 6.2, 6.2 Hz, 1H, 5-H), 2.14 (s, 3H, OAc), 2.04 (s, 3H, OAc), 1.97 (s, 3H, OAc), 1.21 ppm (d, *J* = 6.0 Hz, 3H, 6-H_3_). ^13^C{^1^H} NMR (125.7 MHz, CDCl_3_) δ_C_ 207.7 (CH, C-2’), 170.3 (C, OAc), 170.0 (C, OAc), 169.9 (C, OAc), 87.8 (CH, C-1’), 78.7 (CH_2_, C-3’), 73.3 (CH, C-1), 71.6 (CH, C-4), 70.3 (CH, C-2), 69.3 (CH, C-3), 68.8 (CH, C-5), 21.0 (CH_3_, OAc), 20.8 (CH_3_, OAc), 20.6 (CH_3_, OAc), 17.6 ppm (CH_3_, C-6). IR (CHCl_3_): ν = 2981, 2933, 1955, 1746, 1369, 1221 cm^–1^. MS (ESI) *m*/*z* (%) = 335 (100) [M + Na]^+^. HRMS (ESI) *m/z*: [M + Na]^+^ calcd for C_15_H_20_NaO_7_ 335.1107; found 335.1107. Anal. calcd for C_15_H_20_O_7_: C, 57.69; H, 6.45. Found: C, 58.07; H, 6.79. Compound **102**: ^1^H NMR (500 MHz, CDCl_3_, signals taken from a spectrum of the mix of **101** and **102**) δ_H_ 5.39 (dd, *J* = 2.9, 1.3 Hz, 1H, 2-H), 5.14 (ddd, *J* = 6.6, 6.6, 6.6 Hz, 1H, 1′-H), 5.05 (m, 1H, 3-H), 4.98 (dd, *J* = 6.7, 4.4 Hz, 1H, 4-H), 4.86 (ddd, *J* = 11.4, 6.6, 2.2 Hz, 1H, 3′-H_b_), 4.81 (ddd, *J* = 11.7, 6.9, 2.2 Hz, 1H, 3′-H_a_), 4.19 (dddd, *J* = 6.9, 2.2, 2.2, 1.3 Hz, 1H, 1-H), 3.54 (dddd, *J* = 9.1, 6.3, 6.3, 6.3 Hz, 1H, 5-H), 2.15 (s, 3H, OAc), 2.04 (s, 3H, OAc), 1.96 (s, 3H, OAc), 1.25 ppm (d, *J* = 6.3 Hz, 3H, 6-H_3_). ^13^C{^1^H} NMR (125.7 MHz, CDCl_3_, signals taken from a spectrum of the mix of **101** and **102**) δ_C_ 208.4 (CH, C-2’), 170.4 (C, OAc), 170.3 (C, OAc), 169.9 (C, OAc), 87.3 (CH, C-1’), 77.4 (CH_2_, C-3’), 75.1 (CH, C-1), 74.6 (CH, C-5), 72.3 (CH, C-4), 70.6 (CH, C-3), 70.4 (CH, C-2), 21.0 (CH_3_, OAc), 20.7 (CH_3_, OAc), 20.6 (CH_3_, OAc), 17.7 ppm (CH_3_, C-6). IR (CHCl_3_): ν = 2981, 2937, 1959, 1750, 1373, 1225, 1054 cm^–1^. MS (ESI) *m*/*z* (%) = 335 (100) [M + Na]^+^. HRMS (ESI) *m*/*z*: [M + Na]^+^ calcd for C_15_H_20_NaO_7_ 335.1107; found 335.1107.

#### *C*-(4-*O*-Benzyl-2,3-*O*-isopropylidene-α-l-rhamnopyranosyl)allene (**103**)

To a solution of **101** (1.89 g, 6.06 mmol) in dry MeOH (45 mL) was added K_2_CO_3_ (1.35 g, 9.78 mmol), and the mixture was stirred at room temperature for 3 h. Then, it was filtered and neutralized with the Amberlyst 15 H^+^ ion exchange resin. It was filtered again under vacuum and evaporated. The resulting organic crude was dissolved in acetone (60 mL), and 2,2-dimethoxypropane (1.9 mL, 15.15 mmol) and *p*-TsOH·H_2_O (692 mg, 3.64 mmol) were subsequently added while stirring at room temperature for 3 h. The acetone was evaporated, and the residue was poured over a saturated aqueous solution of NaHCO_3_ and extracted with CH_2_Cl_2_. The organic phase was dried over Na_2_SO_4_ and concentrated to dryness under reduced pressure. The organic crude was dissolved in dry DMF (73 mL) under a N_2_ atmosphere, and NaH 60% in mineral oil (364 mg, 9.09 mmol) was slowly added at 0 °C. After 20 min, BnBr (1.4 mL, 12.12 mmol) was dropwise added and stirring was continued at 0 °C for 3 h. Ice-water was used to destroy the excess of NaH, and the mixture was evaporated in a high vacuum rotovap, poured over a saturated solution of NH_4_Cl, and extracted with CH_2_Cl_2_. The combined extracts were dried over Na_2_SO_4_ and concentrated to dryness under reduced pressure. Column chromatography of the residue (hexanes–EtOAc, 95:5) gave **103** (1.03 g, 3.27 mmol, 54%) as a yellow oil: [α]_D_ = +37.0 (*c* = 0.74, CHCl_3_). ^1^H NMR (500 MHz, CDCl_3_) δ_H_ 7.36–7.24 (m, 5H, Ar), 5.23 (ddd, *J* = 7.0, 7.0, 5.1 Hz, 1H, 1’-H), 4.90–4.86 (m, 3H, OBn, 3’-H_2_), 4.73 (m, 1H, 1-H), 4.62 (d, *J* = 11.7 Hz, 1H, OBn), 4.29 (dd, *J* = 5.4, 1.6 Hz, 1H, 2-H), 4.19 (dd, *J* = 7.0, 5.6 Hz, 1H, 3-H), 3.61 (dddd, *J* = 9.5, 6.0, 6.0, 6.0 Hz, 1H, 5-H), 3.26 (dd, *J* = 9.8, 7.3 Hz, 1H, 4-H), 1.52 (s, 3H, Me), 1.37 (s, 3H, Me), 1.26 ppm (d, *J* = 6.3 Hz, 3H, 6-H_3_). ^13^C{^1^H} NMR (125.7 MHz, CDCl_3_) δ_C_ 208.0 (C, C-2’), 138.2 (C, Ar), 128.1 (2 × CH, Ar), 127.9 (2 × CH, Ar), 127.5 (CH, Ar), 108.6 (C, isopropylidene), 89.5 (CH, C-1’), 81.5 (CH, C-4), 78.0 (CH, C-3), 77.4 (CH_2_, C-3’), 75.5 (CH, C-2), 72.9 (CH_2_, OBn), 70.7 (CH, C-1), 67.0 (CH, C-5), 28.0 (CH_3_, Me), 26.3 (CH_3_, Me), 18.0 ppm (CH_3_, C-6). IR (CHCl_3_): ν = 3016, 2993, 1955, 1228, 1074 cm^–1^. MS (ESI) *m*/*z* (%) = 339 (100) [M + Na]^+^. HRMS (ESI) *m*/*z*: [M + Na]^+^ calcd for C_19_H_24_NaO_4_ 339.1572; found 339.1567. Anal. calcd for C_19_H_24_O_4_: C, 72.13; H, 7.65. Found: C, 72.06; H, 7.74.

#### *C*-(2,3-*O*-[(2*S*,3*S*)-2,3-Dimethoxybutane-2,3-diyl]-α-l-fucopyranosyl)allene (**108**)

To a solution of **107** (5.07 g, 16.25 mmol) in dry MeOH (244 mL) was added K_2_CO_3_ (3.6 g, 26 mmol), and the mixture was stirred at room temperature for 3 h. Then, it was filtered and neutralized with the Amberlyst 15 H^+^ ion exchange resin. It was filtered again under vacuum and evaporated. The resulting organic crude was dissolved in dry MeOH (81.3 mL), and 2,3-butanedione (2.85 mL, 32.5 mmol), (MeO)_3_CH (7.1 mL, 65 mmol), and BF_3_•Et_2_O (3.4 mL, 30.88 mmol) were added dropwise. The mixture was stirred at 60 °C for 4.5 h. A few pipettes of Et_3_N were added while stirring at room temperature for 15 min. Then, it was evaporated to dryness. Column chromatography of the residue (hexanes–EtOAc, 7:3) gave **108** (3.18 g, 10.6 mmol, 65%) as a colorless oil: [α]_D_ = −16.9 (*c* = 1.15, CHCl_3_). ^1^H NMR (500 MHz, CDCl_3_) δ_H_ 5.38 (ddd, *J* = 6.8, 6.8, 4.4 Hz, 1H, 1’-H), 4.85 (dd, *J* = 6.9, 4.1 Hz, 2H, 3’-H_2_), 4.61 (dddd, *J* = 6.0, 4.1, 4.1, 4.1 Hz, 1H, 1-H), 4.29 (dd, *J* = 10.6, 6.0 Hz, 1H, 2-H), 4.00 (dddd, *J* = 6.3, 6.3, 6.3, 0.0 Hz, 1H, 5-H), 3.85 (dd, *J* = 10.6, 3.1 Hz, 1H, 3-H), 3.72 (br s, 1H, 4-H), 3.24 (s, 3H, OMe), 3.23 (s, 3H, OMe), 2.39 (br s, 1H, OH), 1.31 (s, 3H, Me), 1.26 (d, *J* = 6.3 Hz, 3H, 6-H_3_), 1.25 ppm (s, 3H, Me). ^13^C{^1^H} NMR (125.7 MHz, CDCl_3_) δ_C_ 208.7 (C, C-2’), 100.1 (C), 99.8 (C), 85.8 (CH, C-1’), 77.0 (CH_2_, C-3’), 71.5 (CH, C-1), 70.8 (CH, C-4), 68.4 (CH, C-5), 67.9 (CH, C-3), 64.0 (CH, C-2), 47.9 (2 × CH_3_, 2 × OMe), 17.6 (2 × CH_3_, Me, C-6), 16.5 ppm (CH_3_, Me). IR (CHCl_3_): ν = 3672, 3583, 3010, 2942, 1957, 1226 cm^–1^. MS (ESI) *m*/*z* (%) = 323 (100) [M + Na]^+^. HRMS (ESI) *m*/*z*: [M + Na]^+^ calcd for C_15_H_24_NaO_6_ 323.1471; found 323.1476. Anal. calcd for C_15_H_24_O_6_: C, 59.98; H, 8.05. Found: C, 59.90; H, 8.35.

### General Procedure to Give Diphenoxyphosphoryl Derivatives **92**, **95**, and **99**

The corresponding alcohol (1 mmol) in dry CH_2_Cl_2_ (58 mL) was treated with DMAP (562 mg, 4.61 mmol) and ClPO(OPh)_2_ (0.95 mL, 4.61 mmol) at room temperature for 2 h. The reaction was quenched with a saturated aqueous solution of NH_4_Cl and extracted with CH_2_Cl_2_. The organic phase was dried over Na_2_SO_4_, filtered, and evaporated. Column chromatography of the residue (hexanes–EtOAc) gave the diphenoxyphosphoryl derivatives.

#### *C*-(6-*O*-*tert*-Butyldiphenylsilyl-4-*O*-diphenoxyphosphoryl-2,3-di-*O*-methyl-α-d-glucopyranosyl)allene (**92**)

To a solution of the allene **91** (97.4 mg, 0.19 mmol) in dry MeOH (0.95 mL) was added K_2_CO_3_ (2.1 mg, 0.015 mmol), and the mixture was stirred at room temperature overnight. The residue was evaporated to afford the intermediate alcohol that was submitted to the general procedure to give the diphenoxylphosphoryl derivative for 2.5 h. Column chromatography (hexanes–EtOAc, 85:15) gave **92** (96.5 mg, 0.14 mmol, 73%) as a colorless oil: [α]_D_ = +45.6 (*c* = 0.45, CHCl_3_). ^1^H NMR (500 MHz, CDCl_3_) δ_H_ 7.68–7.07 (m, 20H, Ar), 5.36 (ddd, *J* = 8.3, 8.3, 8.3 Hz, 1H, 1’-H), 4.84 (dd, *J* = 8.6, 0.0 Hz, 1H, 3’-H_b_), 4.83 (dd, *J* = 8.4, 0.0 Hz, 1H, 3’-H_a_), 4.74 (m, 1H, 1-H), 4.62 (ddd, *J* = 8.5, 8.5 Hz, ^3^*J*_PH_ = 8.5 Hz, 1H, 4-H), 3.96–3.87 (m, 2H, 5-H, 6-H_b_), 3.79 (dd, *J* = 14.9, 6.7 Hz, 1H, 6-H_a_), 3.57 (dd, *J* = 10.9, 8.3 Hz, 1H, 3-H), 3.46 (m, 1H, 2-H), 3.45 (s, 6H, OMe), 1.04 ppm (s, 9H, *^t^*Bu). ^13^C{^1^H} NMR (100.6 MHz, CDCl_3_) δ_C_ 209.6 (C, C-2’), 150.8 (d, ^2^*J*_PC_ = 7.0 Hz, C, Ar), 150.6 (d, ^2^*J*_PC_ = 7.8 Hz, C, Ar), 133.6 (2 × C, Ar), 119.9–135.7 (20 × CH, Ar), 85.2 (CH, C-1’), 81.6 (CH, C-2), 81.1 (CH, C-3), 76.7 (CH_2_, C-3’), 76.6 (CH, C-4), 72.8 (d, ^3^*J*_PC_ = 7.0 Hz, CH, C-5), 70.9 (CH, C-1), 63.0 (CH_2_, C-6), 60.2 (CH_3_, OMe), 58.2 (CH_3_, OMe), 26.7 (3 × CH_3_, DPS), 19.3 ppm (C, DPS). IR (CHCl_3_): ν = 3015, 2933, 1955, 1592, 1490, 1191 cm^–1^. MS (ESI) *m*/*z* (%) = 723 (100) [M + Na]^+^. HRMS (ESI) *m*/*z*: [M + Na]^+^ calcd for C_39_H_45_NaO_8_PSi 723.2519; found 723.2510. Anal. calcd for C_39_H_45_O_8_Psi: C, 66.84; H, 6.47. Found: C, 66.90; H, 6.42.

#### *C*-(4,6-Bis-*O*-diphenoxyphosphoryl-2,3-di-*O*-methyl-α-d-glucopyranosyl)methanol (**95**)

Compound **48** (620 mg, 1.24 mmol) was dissolved in CH_2_Cl_2_ (6.2 mL), and DHP (282 μL, 3.09 mmol) and *p*-TsOH·H_2_O (15 mg, 0.08 mmol) were added while stirring at room temperature for 2 h. The reaction was poured over a saturated aqueous solution of NaHCO_3_ and extracted with CH_2_Cl_2_. The organic extract was dried over Na_2_SO_4_, filtered, and evaporated. The crude in dry THF (25 mL) was treated with TBAF/THF 1 M solution (1.9 mL, 1.9 mmol) for 3 h at room temperature. Then, the mixture was evaporated to dryness and quickly chromatographed (hexanes–EtOAc 3:7) to obtain the corresponding alcohol (315 mg, 0.91 mmol, 73%) as an orange oil that was saponified with K_2_CO_3_ (25 mg, 0.18 mmol) in MeOH (4.6 mL) at room temperature for 4 h, filtered over a pad of Celite, and concentrated. The resulting diol was treated with ClPO(OPh)_2_ (1.7 mL, 8.19 mmol) and dry pyridine (9.1 mL, 112.5 mmol) at room temperature overnight. The reaction was evaporated in a high vacuum rotovap, quenched with an aqueous solution of HCl 10%, and extracted with CH_2_Cl_2_. The organic phase was washed with a saturated aqueous solution of NaHCO_3_, dried over Na_2_SO_4_, and evaporated. Finally, the THP protecting group was hydrolyzed by treatment with *p*-TsOH·H_2_O (17.3 mg, 0.091 mmol) in MeOH (1.8 mL) at room temperature for 2 h. The mixture was poured over a saturated aqueous solution of NaHCO_3_ and extracted with CH_2_Cl_2_. The organic phase was dried over Na_2_SO_4_, filtered, and evaporated. Column chromatography of the residue (hexanes–EtOAc, 4:6) gave **95** (315.3 mg, 0.46 mmol, 37% overall yield) as a colorless oil: [α]_D_ = +20.6 (*c* = 1.25, CHCl_3_). ^1^H NMR (500 MHz, CDCl_3_) δ_H_ 7.34–7.11 (m, 20H, Ar), 4.33–4.20 (m, 3H, 4-H, 6-H_2_), 4.14–4.09 (m, 2H, 1-H, 5-H), 3.90 (dd, *J* = 12.6, 8.8 Hz, 1H, 1’-H_b_), 3.70 (dd, *J* = 12.6, 3.5 Hz, 1H, 1’-H_a_), 3.51 (dd, *J* = 8.2, 8.2 Hz, 1H, 3-H), 3.42 (s, 6H, 2 × OMe), 3.40 ppm (dd, *J* = 8.2, 5.7 Hz, 1H, 2-H), 1H from OH is missing. ^13^C{^1^H} NMR (125.7 MHz, CDCl_3_) δ_C_ 150.7 (d, ^2^*J*_PC_ = 7.4 Hz, C, Ar), 150.6 (d, ^2^*J*_PC_ = 7.4 Hz, C, Ar), 150.5 (d, ^2^*J*_PC_ = 7.4 Hz, C, Ar), 150.3 (d, ^2^*J*_PC_ = 7.4 Hz, C, Ar), 120.0–129.8 (20 × CH, Ar), 80.4 (2 × CH, C-2, C-3), 75.9 (d, ^2^*J*_PC_ = 6.4 Hz, CH, C-4), 74.1 (CH, C-1), 70.9 (CH, C-5), 68.3 (d, ^2^*J*_PC_ = 7.4 Hz, CH_2_, C-6), 60.3 (CH_3_, OMe), 58.8 (CH_3_, OMe), 58.3 ppm (CH_2_, C-1’). IR (CHCl_3_): ν = 3690, 3620, 3024, 2401, 1491, 1226 cm^–1^. MS (ESI) *m*/*z* (%) = 709 (100) [M + Na]^+^. HRMS (ESI) *m*/*z*: [M + Na]^+^ calcd for C_33_H_36_NaO_12_P_2_ 709.1580; found 709.1582. Anal. calcd for C_33_H_36_O_12_P_2_: C, 57.73; H, 5.28. Found: C, 57.82; H, 5.62.

#### *C*-(6-O-*tert*-Butyldiphenylsilyl-4-*O*-diphenoxyphosphoryl-2,3-di-*O*-methyl-α-d-galactopyranosyl)allene (**99**)

To a solution of the acetyl derivative **98** (749.5 mg, 1.47 mmol) in MeOH (7.3 mL) was added K_2_CO_3_ (16.2 mg, 0.12 mmol), and it was stirred at room temperature overnight. The mixture was filtered, evaporated, and submitted to the general procedure to give the diphenoxyphosphoryl derivative. Column chromatography (hexanes–EtOAc, 8:2) of the residue afforded **99** (928.6 mg, 1.32 mmol, 90%) as a colorless oil: [α]_D_ = +74.6 (*c* = 0.46, CHCl_3_). ^1^H NMR (400 MHz, CDCl_3_) δ_H_ 7.08–7.65 (m, 20H, Ar), 5.28–5.24 (m, 2H, 1-H, 1’-H), 4.79–4.67 (m, 3H, 4-H, 3’-H_2_), 3.85 (m, 1H, 5-H), 3.79 (dd, *J* = 10.1, 7.4 Hz, 1H, 6-H_b_), 3.69 (dd, *J* = 10.1, 6.1 Hz, 1H, 6-H_a_), 3.61 (dd, *J* = 9.6, 5.6 Hz, 1H, 3-H), 3.41 (s, 3H, OMe), 3.38 (s, 3H, OMe), 3.32 (dd, *J* = 9.6, 1.3 Hz, 1H, 2-H), 1.03 ppm (s, 9H, *^t^*Bu). ^13^C{^1^H} NMR (100.6 MHz, CDCl_3_) δ_C_ 208.8 (C, C-2’), 150.8 (d, ^2^*J*_PC_ = 7.8 Hz, C, Ar), 150.7 (d, ^2^*J*_PC_ = 7.1 Hz, C, Ar), 135.61 (2 × CH, Ar), 135.56 (2 × CH, Ar), 133.5 (C, Ar), 133.3 (C, Ar), 129.8 (CH, Ar), 129.7 (CH, Ar), 129.6 (2 × CH, Ar), 129.3 (2 × CH, Ar), 127.64 (2 × CH, Ar), 127.59 (2 × CH, Ar), 125.0 (CH, Ar), 124.9 (CH, Ar), 120.52 (CH, Ar), 120.45 (CH, Ar), 120.12 (CH, Ar), 120.07 (CH, Ar), 85.3 (CH, C-1’), 79.1 (CH, C-2), 76.7 (CH_2_, C-3’), 76.7 (CH, C-3), 74.5 (d, ^2^*J*_PC_ = 6.3 Hz, CH, C-4), 71.7 (d, ^3^*J*_PC_ = 5.6 Hz, CH, C-5), 71.3 (CH, C-1), 62.1 (CH_2_, C-6), 59.0 (CH_3_, OMe), 57.5 (CH_3_, OMe), 26.7 (3 × CH_3_, DPS), 19.1 ppm (C, DPS). IR (CHCl_3_): ν = 3016, 2933, 1956, 1592, 1490, 1112 cm^–1^. MS (ESI) *m*/*z* (%) = 723 (100) [M + Na]^+^. HRMS (ESI) *m*/*z*: [M + Na]^+^ calcd for C_39_H_45_NaO_8_PSi 723.2519; found 723.2513. Anal. calcd for C_39_H_45_O_8_PSi: C, 66.84; H, 6.47. Found: C, 66.80; H, 6.48.

#### *C*-(6-*O*-*tert*-Butyldiphenylsilyl-2,3-di-*O*-methyl-4-*O*-tosyl-α-d-glucopyranosyl)allene (**93**)

To a solution of the allene **91** (301 mg, 0.59 mmol) in dry MeOH (3 mL), K_2_CO_3_ (6 mg, 0.04 mmol) was added and the mixture was stirred at room temperature overnight. The residue was evaporated to afford the intermediate alcohol that was dissolved in dry pyridine (6 mL) and treated with TsCl (343 mg, 1.8 mmol) overnight. The reaction was evaporated at high vacuum rotovap, quenched with HCl 10%, and extracted with CH_2_Cl_2_. The organic phase was washed with a saturated aqueous solution of NaHCO_3_, dried over Na_2_SO_4_, filtered, and evaporated. Column chromatography (hexanes–EtOAc, 9:1) gave **93** (165.1 mg, 0.27 mmol, 45%) as a colorless oil: [α]_D_ = +69.6 (*c* = 0.46, CHCl_3_). ^1^H NMR (500 MHz, CDCl_3_) δ_H_ 7.78–7.18 (m, 14H, Ar), 5.31 (ddd, *J* = 6.7, 6.7, 6.7 Hz, 1H, 1’-H), 4.82 (dd, *J* = 6.7, 0.0 Hz, 1H, 3’-H_b_), 4.81 (dd, *J* = 6.9, 0.0 Hz, 1H, 3’-H_a_), 4.75–4.70 (m, 2H, 1-H, 3-H), 3.88 (dd, *J* = 11.4, 2.2 Hz, 1H, 6-H_b_), 3.82 (m, 1H, 4-H), 3.76 (dd, *J* = 11.0, 4.7 Hz, 1H, 6-H_a_), 3.46 (s, 3H, OMe), 3.42–3.40 (m, 2H, 2-H, 5-H), 3.23 (s, 3H, OMe), 2.39 (s, 3H, OTs), 1.09 ppm (s, 9H, *^t^*Bu). ^13^C{^1^H} NMR (125.7 MHz, CDCl_3_) δ_C_ 209.5 (C, C-2’), 144.2 (C, Ar), 135.0 (C, Ar), 133.7 (C, Ar), 133.6 (C, Ar), 127.5–135.9 (14 × CH, Ar), 85.3 (CH, C-1’), 81.7 (CH, C-2 or C-5), 80.7 (CH, C-2 or C-5), 77.9 (CH, C-1), 76.8 (CH_2_, C-3’), 72.4 (CH, C-4), 70.8 (CH, C-3), 62.8 (CH_2_, C-6), 60.3 (CH_3_, OMe), 58.4 (CH_3_, OMe), 26.8 (3 × CH_3_, DPS), 21.6 (CH_3_, OTs), 19.4 ppm (C, DPS). IR (CHCl_3_): ν = 3015, 2933, 1955, 1599, 1373, 1112 cm^–1^. MS (ESI) *m*/*z* (%) = 645 (100) [M + Na]^+^. HRMS (ESI) *m*/*z*: [M + Na]^+^ calcd for C_34_H_42_NaO_7_SSi 645.2318; found 645.2321. Anal. calcd for C_34_H_42_O_7_SSi: C, 65.56; H, 6.80. Found: C, 65.40; H, 7.05.

### General Procedure for the Synthesis of the Hydroxymethyl Derivatives **48**, **49**, **51**, **52**, **58**, **59**, **94**, **104**, and **109**

A solution of the allene (1 mmol) in CH_2_Cl_2_–MeOH (30 mL, 4:1) was cooled to −78 °C, and ozone was bubbled into the solution until it became blue. Then, nitrogen was introduced through the mixture to expel the excess of ozone, and it was heated to 0 °C. Afterward, NaBH_4_ (75.3 mg, 1.99 mmol) was added slowly and the solution was stirred for 2 h at room temperature. The reaction mixture was then poured into brine, extracted with CH_2_Cl_2_, dried over Na_2_SO_4_, and concentrated. Column chromatography (hexanes–EtOAc) of the residue afforded the title alcohol.

#### *C*-(4-*O*-Acetyl-6-*O*-*tert*-butyldiphenylsilyl-2,3-di-*O*-methyl-α-d-glucopyranosyl)methanol (**48**)

Following the general procedure starting from allene **91** (524 mg, 1.03 mmol) and purification by column chromatography (hexanes–EtOAc, 25:75), alcohol **48** (332.7 mg, 0.66 mmol, 64%) was obtained as a colorless oil: [α]_D_ = +19.0 (*c* = 0.39, CHCl_3_). ^1^H NMR (500 MHz CDCl_3_, 2-H and 3-H are strongly coupled; therefore, 4-H and 1-H show virtual coupling effects; the chemical shifts and coupling constants shown below were obtained using DAISY) δ_H_ 7.70–7.65 ppm (m, 4H, Ar), 7.45–7.36 (m, 6H, Ar), 4.89 (dd, *J* = 8.0, 5.8 Hz, 1H, 4-H), 4.13 (ddd, *J* = 8.6, 4.8, 4.6 Hz, 1H, 1-H), 3.91 (dd, *J* = 12.0, 8.6 Hz, 1H, 1’-H_b_), 3.77 (dd, *J* = 12.0, 4.6 Hz, 1H, 1’-H_a_), 3.76 (ddd, *J* = 8.0, 5.8, 4.1 Hz, 1H, 5-H), 3.72 (dd, *J* = 10.8, 4.1 Hz, 1H, 6-H_b_), 3.71 (dd, *J* = 10.8, 5.8 Hz, 1H, 6-H_a_), 3.480 (s, 3H, OMe), 3.478 (s, 3H, OMe), 3.467 (dd, *J* = 7.8, 7.1 Hz, 1H, 3-H), 3.467 (dd, *J* = 7.1, 4.8 Hz, 1H, 2-H), 1.98 (s, 3H, OAc), 1.06 ppm (s, 9H, *^t^*Bu), 1H from OH is missing. ^1^H NMR (500 MHz, C_6_D_6_) δ_H_ 7.85–7.82 (m, 4H, Ar), 7.26–7.21 (m, 6H, Ar), 5.21 (dd, *J* = 8.5, 8.5 Hz, 1H, 4-H), 4.07 (ddd, *J* = 5.0, 5.0, 8.3 Hz, 1H, 1-H), 3.84–3.79 (m, 5H, 1’-H_2_, 5-H, 6-H_2_), 3.44 (dd, *J* = 8.4, 8.4 Hz, 1H, 3-H), 3.31 (s, 3H, OMe), 3.26 (dd, *J* = 8.5, 5.9 Hz, 1H, 2-H), 2.99 (s, 3H, OMe), 1.63 (s, 3H, OAc), 1.23 ppm (s, 9H, *^t^*Bu). ^13^C{^1^H} NMR (100.6 MHz, CDCl_3_) δ_C_ 169.7 (C, OAc), 135.7 (2 × CH, Ar), 135.6 (2 × CH, Ar), 133.3 (C, Ar), 133.2 (C, Ar), 129.72 (2 × CH, Ar), 129.70 (2 × CH, Ar), 127.69 (2 × CH, Ar), 127.67 (2 × CH, Ar), 79.9 (CH, C-2 or C-5), 79.8 (CH, C-2 or C-5), 73.4 (CH, C-4), 72.5 (CH, C-1), 69.6 (CH, C-3), 63.2 (CH_2_, C-6), 59.8 (CH_3_, OMe), 59.6 (CH_2_, C-1’), 58.9 (CH_3_, OMe), 26.8 (3 × CH_3_, DPS), 20.9 (CH_3_, OAc), 19.2 ppm (C, DPS). IR (CHCl_3_): ν = 3680, 3553, 2934, 1742, 1217 cm^–1^. MS (ESI) *m*/*z* (%) = 525 (100) [M + Na]^+^. HRMS (ESI) *m*/*z*: [M + Na]^+^ calcd for C_27_H_38_NaO_7_Si 525.2285; found 525.2286.

#### *C*-(6-*O*-*tert*-Butyldiphenylsilyl-2,3-di-*O*-methyl-4-*O*-tosyl-α-d-glucopyranosyl)methanol (**49**)

Following the general procedure starting from allene **93** (226.2 mg, 0.36 mmol) and purification by column chromatography (hexanes–EtOAc, 6:4), alcohol **49** (147.9 mg, 0.24 mmol, 67%) was obtained as a colorless oil: [α]_D_ = +15.2 (*c* = 0.54, CHCl_3_). ^1^H NMR (500 MHz, CDCl_3_, simulated coupling constants using DAISY) δ_H_ 7.74–7.17 (m, 14H, Ar), 4.64 (dd, *J* = 7.6, 6.8 Hz, 1H, 4-H), 4.04 (ddd, *J* = 8.5, 5.1, 4.4 Hz, 1H, 1-H), 3.834 (dd, *J* = 10.9, 3.7 Hz, 1H, 6-H_b_), 3.831 (dd, *J* = 12.1, 8.5 Hz, 1H, 1’-H_b_), 3.73 (ddd, *J* = 7.6, 6.0, 3.7 Hz, 1H, 5-H), 3.69 (dd, *J* = 12.1, 4.4 Hz, 1H, 1’-H_a_), 3.66 (dd, *J* = 10.9, 6.0 Hz, 1H, 6-H_a_), 3.51 (dd, *J* = 7.2, 6.8 Hz, 1H, 3-H), 3.47 (s, 3H, OMe), 3.43 (dd, *J* = 7.2, 5.1 Hz, 1H, 2-H), 3.29 (s, 3H, OMe), 2.36 (s, 3H, OTs), 1.05 ppm (s, 9H, *^t^*Bu), 1H from OH is missing. ^13^C{^1^H} NMR (100.6 MHz, CDCl_3_) δ_C_ 144.5 (C, OTs), 134.3 (C, OTs), 133.3 (C, Ar), 133.2 (C, Ar), 127.6–135.6 (14 × CH, Ar), 79.8 (CH, C-2), 79.1 (CH, C-3), 76.3 (CH, C-4), 73.0 (CH, C-5), 72.2 (CH, C-1), 62.5 (CH_2_, C-6), 59.8 (CH_3_, OMe), 59.7 (CH_2_, C-1’), 58.7 (CH_3_, OMe), 26.7 (3 × CH_3_, DPS), 21.6 (CH_3_, OTs), 19.2 ppm (C, DPS). IR (CHCl_3_): ν = 3694, 3574, 3024, 2934, 1600, 1104 cm^–1^. MS (ESI) *m*/*z* (%) = 637 (100) [M + Na]^+^. HRMS (ESI) *m*/*z*: [M + Na]^+^ calcd for C_32_H_42_NaO_8_SSi 637.2267; found 637.2257. Anal. calcd for C_32_H_42_O_8_SSi: C, 62.51; H, 6.89; S, 5.22. Found: C, 62.29; H, 7.09; S, 4.86.

#### *C*-(4-*O*-Acetyl-6-*O*-*tert*-butyldiphenylsilyl-2,3-di-*O*-methyl-α-d-galactopyranosyl)methanol (**51**)

Following the general procedure starting from allene **98** (461.5 mg, 0.90 mmol) and purification by column chromatography (hexanes–EtOAc, 25:75), alcohol **51** (316.3 mg, 0.63 mmol, 70%) was obtained as a colorless oil: [α]_D_ = +30.6 (*c* = 0.66, CHCl_3_). ^1^H NMR (500 MHz, CDCl_3_, simulated ring coupling constants using DAISY) δ_H_ 7.67–7.64 (m, 4H, Ar), 7.46–7.37 (m, 6H, Ar), 5.52 (dd, *J* = 3.3, 1.9 Hz, 1H, 4-H), 4.20 (ddd, *J* = 8.4, 6.1, 4.8 Hz, 1H, 1-H), 3.83 (ddd, *J* = 6.6, 6.2, 1.9 Hz, 1H, 5-H), 3.82 (dd, *J* = 12.0, 8.4 Hz, 1H, 1’-H_b_), 3.77 (dd, *J* = 12.0, 5.1 Hz, 1H, 1’-H_a_), 3.73 (dd, *J* = 10.4, 6.6 Hz, 1H, 6-H_b_), 3.66 (dd, *J* = 9.1, 6.1 Hz, 1H, 2-H), 3.61 (dd, *J* = 10.4, 6.2 Hz, 1H, 6-H_a_), 3.48 (s, 3H, OMe), 3.42 (dd, *J* = 9.1, 3.3 Hz, 1H, 3-H), 3.41 (s, 3H, OMe), 2.02 (s, 3H, OAc), 1.06 ppm (s, 9H, *^t^*Bu), 1H from OH is missing. ^13^C{^1^H} NMR (100.6 MHz, CDCl_3_) δ_C_ 169.9 (C, OAc), 135.6 (4 × CH, Ar), 133.2 (C, Ar), 133.0 (C, Ar), 129.8 (2 × CH, Ar), 127.7 (4 × CH, Ar), 79.0 (CH, C-2 or C-5), 77.5 (CH, C-2 or C-5), 73.4 (CH, C-4), 71.9 (CH, C-1), 66.6 (CH, C-3), 62.2 (CH_2_, C-6), 59.4 (CH_3_, OMe), 59.1 (CH_2_, C-1’), 57.7 (CH_3_, OMe), 26.8 (3 × CH_3_, DPS), 20.7 (CH_3_, OAc), 19.1 ppm (C, DPS). IR (CHCl_3_): ν = 3686, 3620, 3015, 2975, 1742, 1229 cm^–1^. MS (ESI) *m*/*z* (%) = 525 (100) [M + Na]^+^. HRMS (ESI) *m*/*z*: [M + Na]^+^ calcd for C_27_H_38_NaO_7_Si 525.2285; found 525.2284. Anal. calcd for C_27_H_38_O_7_Si: C, 64.51; H, 7.62. Found: C, 64.20; H, 7.65.

#### *C*-(6-*O*-*tert*-Butyldiphenylsilyl-4-*O*-diphenoxyphosphoryl-2,3-di-*O*-methyl-α-d-galactopyranosyl)methanol (**52**)

Following the general procedure starting from allene **99** (896.2 g, 1.28 mmol) and purification by column chromatography (hexanes–EtOAc, 6:4), alcohol **52** (567.3 mg, 0.82 mmol, 64%) was obtained as a colorless oil: [α]_D_ = +25.0 (*c* = 0.32, CHCl_3_). ^1^H NMR (500 MHz, CDCl_3_) δ_H_ 7.65–7.05 (m, 20H, Ar), 5.06 (ddd, *J* = 2.6, 1.6 Hz, ^3^*J*_PH_ = 8.5 Hz, 1H, 4-H), 4.09 (ddd, *J* = 7.3, 5.7, 5.7 Hz, 1H, 1-H), 3.80–3.68 (m, 5H, 5-H, 6-H_2_, 1’-H_2_), 3.57 (dd, *J* = 8.8, 5.6 Hz, 1H, 2-H), 3.389 (s, 3H, OMe), 3.38 (dd, *J* = 8.8, 2.6 Hz, 1H, 3-H), 3.35 (s, 3H, OMe), 1.93 (br s, 1H, OH), 1.03 ppm (s, 9H, *^t^*Bu). ^13^C{^1^H} NMR (125.7 MHz, CDCl_3_) δ_C_ 150.8 (d, ^2^*J*_PC_ = 7.4 Hz, C, Ar), 150.5 (d, ^2^*J*_PC_ = 7.4 Hz, C, Ar), 135.6 (2 × CH, Ar), 135.5 (2 × CH, Ar), 133.3 (C, Ar), 133.2 (C, Ar), 129.74 (CH, Ar), 129.72 (CH, Ar), 129.6 (2 × CH, Ar), 129.4 (2 × CH, Ar), 127.7 (4 × CH, Ar), 125.2 (CH, Ar), 125.1 (CH, Ar), 120.33 (CH, Ar), 120.28 (CH, Ar), 120.00 (CH, Ar), 119.96 (CH, Ar), 78.9 (CH, C-3), 77.0 (CH, C-2), 74.2 (d, ^2^*J*_PC_ = 6.3 Hz, CH, C-4), 72.8 (d, ^3^*J*_PC_ = 6.3 Hz, CH, C-5), 72.6 (CH, C-1), 62.2 (CH_2_, C-6), 59.58 (CH_3_, OMe), 59.2 (CH_2_, C-1’), 57.7 (CH_3_, OMe), 26.7 (3 × CH_3_, DPS), 19.0 ppm (C, DPS). IR (CHCl_3_): ν = 3690, 3546, 3023, 2934, 1592, 1206 cm^–1^. MS (ESI) *m*/*z* (%) = 715 (100) [M + Na]^+^. HRMS (ESI) *m*/*z*: [M + Na]^+^ calcd for C_37_H_45_NaO_9_PSi 715.2468; found 715.2475. Anal. calcd for C_37_H_45_O_9_PSi: C, 64.14; H, 6.55. Found: C, 64.02; H, 6.67.

#### *C*-(4-*O*-Acetyl-2,3-di-*O*-methyl-α-d-fucopyranosyl)methanol (**58**)

Alcohol **109** (200 mg, 0.68 mmol) was dissolved in dry DMF (2.7 mL), and DPSCl (238 μL, 1.01 mmol) and imidazole (138 mg, 2.03 mmol) were added at room temperature. After 3 h, the reaction was evaporated in a high vacuum rotovap, quenched with H_2_O, and extracted with CH_2_Cl_2_. The organic phase was dried over Na_2_SO_4_, filtered, and evaporated. To a solution of the resulting crude in dry EtOAc (16 mL) was added Pd/C 10% (152 mg), and the mixture was submitted to H_2_ atmosphere overnight. The reaction was filtered over a pad of Celite and evaporated. The alcohol was protected by treatment with dry pyridine (2.6 mL), Ac_2_O (0.9 mL), and DMAP (8.3 mg, 0.068 mmol) for 0.5 h. The mixture was evaporated in a high vacuum rotovap, quenched with aqueous HCl 10%, and extracted with CH_2_Cl_2_. The organic phase was washed with a saturated aqueous solution of NaHCO_3_, dried with Na_2_SO_4_, filtered, and concentrated to dryness. Finally, the silyl group was deprotected by treatment with a 1 M solution of TBAF/THF (1.4 mL, 1.4 mmol) in dry THF (13.6 mL) for 4 h at room temperature. The mixture was evaporated, and the residue was chromatographed in a silica gel column (hexanes–EtOAc, 4:6 to 2:8) to give **58** (111 mg, 0.45 mmol, 66%) as a colorless oil: [α]_D_ = −76.0 (*c* = 0.65, CHCl_3_). ^1^H NMR (500 MHz, CDCl_3_, simulated ring coupling constants using DAISY) δ_H_ 5.33 (dd, *J* = 3.5, 2.0 Hz, 1H, 4-H), 4.23 (ddd, *J* = 8.0, 5.9, 4.7 Hz, 1H, 1-H), 3.96 (dddd, *J* = 6.5, 6.5, 6.5, 2.0 Hz, 1H, 5-H), 3.89 (dd, *J* = 12.1, 8.0 Hz, 1H, 1’-H_b_), 3.85 (dd, *J* = 12.1, 4.7 Hz, 1H, 1’-H_a_), 3.71 (dd, *J* = 8.2, 5.9 Hz, 1H, 2-H), 3.50 (dd, *J* = 8.2, 3.5 Hz, 1H, 3-H), 3.50 (s, 3H, OMe), 3.42 (s, 3H, OMe), 2.17 (s, 3H, OAc), 2.10 (br s, 1H, OH), 1.17 ppm (d, *J* = 6.5 Hz, 3H, 6-H_3_). ^13^C{^1^H} NMR (125.7 MHz, CDCl_3_) δ_C_ 170.6 (C, OAc), 78.7 (CH, C-3), 77.1 (CH, C-2), 73.4 (CH, C-1), 69.5 (CH, C-4), 67.0 (CH, C-5), 59.5 (CH_2_, C-1’), 59.3 (CH_3_, OMe), 57.5 (CH_3_, OMe), 20.8 (CH_3_, OAc), 16.5 ppm (CH_3_, C-6). IR (CHCl_3_): ν = 3677, 3502, 3018, 2939, 1739, 1239 cm^–1^. MS (ESI) *m*/*z* (%) = 271 (100) [M + Na]^+^. HRMS (ESI) *m*/*z*: [M + Na]^+^ calcd for C_11_H_20_NaO_6_ 271.1158; found 271.1159.

#### *C*-(4-*O*-Diphenoxyphosphoryl-2,3-di-*O*-methyl-α-d-fucopyranosyl)methanol (**59**)

Alcohol **109** (1.4 g, 4.73 mmol) was dissolved in dry DMF (18.9 mL), and imidazole (960 mg, 14.1 mmol) and DPSCl (1.7 mL, 7.25 mmol) were added at room temperature. After 3 h, the reaction was evaporated in a high vacuum rotovap, quenched with H_2_O, and extracted with CH_2_Cl_2_. The organic phase was dried over Na_2_SO_4_, filtered, and evaporated. To a solution of the resulting crude in dry EtOAc (111 mL) was added Pd/C 10% (1.06 g), and the mixture was submitted to a H_2_ atmosphere overnight. The reaction was filtered over a pad of Celite and evaporated. The alcohol was protected without purification by treatment with DMAP (2.66 g, 21.7 mmol) and ClPO(OPh)_2_ (2.82 mL, 21.7 mmol) in dry CH_2_Cl_2_ (110 mL) for 3.5 h. The mixture was quenched with a saturated aqueous NH_4_Cl solution and extracted with CH_2_Cl_2_. The organic phase was dried with Na_2_SO_4_, filtered, and concentrated to dryness. Finally, the silyl group was deprotected by treatment with a 1 M solution of TBAF/THF (9.46 mL, 9.46 mmol) in dry THF (95 mL) for 3.5 h at room temperature. The mixture was evaporated, and the residue was chromatographed on a silica gel column (hexanes–EtOAc, 1:1) to give **59** (1.24 g, 2.84 mmol, 60%) as a colorless oil: [α]_D_ = −41.8 (*c* = 0.49, CHCl_3_). ^1^H NMR (400 MHz, CDCl_3_, simulated coupling constants using DAISY) δ_H_ 7.37–7.17 (m, 10H, Ar), 4.92 (ddd, *J* = 3.0, 2.5 Hz, ^3^*J*_PH_ = 9.1 Hz, 1H, 4-H), 4.14 (ddd, *J* = 7.5, 5.7, 5.2 Hz, 1H, 1-H), 3.96 (ddddd, *J* = 6.5, 6.5, 6.5, 2.5 Hz, ^4^*J*_PH_ = 1.9 Hz, 1H, 5-H), 3.87–3.77 (m, 2H, 1’-H_2_), 3.60 (dd, *J* = 8.4, 5.2 Hz, 1H, 2-H), 3.53 (ddd, *J* = 8.4, 3.0, Hz, ^4^*J*_PH_ = 1.0 Hz, 1H, 3-H), 3.43 (s, 3H, OMe), 3.41 (s, 3H, OMe), 1.22 ppm (d, *J* = 6.5 Hz, 3H, 6-H_3_), 1H from OH is missing. ^13^C{^1^H} NMR (125.7 MHz, CDCl_3_) δ_C_ 150.8 (d, ^2^*J*_PC_ = 8.5 Hz, C, Ar), 150.6 (d, ^2^*J*_PC_ = 7.4 Hz, C, Ar), 129.7 (2 × CH, Ar), 129.3 (2 × CH, Ar), 125.3 (CH, Ar), 125.1 (CH, Ar), 120.32 (CH, Ar), 120.28 (CH, Ar), 120.11 (CH, Ar), 120.07 (CH, Ar), 78.7 (CH, C-3), 76.9 (CH, C-2), 76.8 (d, ^3^*J*_PC_ = 6.3 Hz, CH, C-4), 72.3 (CH, C-1), 67.9 (d, ^3^*J*_PC_ = 5.3 Hz, CH, C-5), 60.1 (CH_2_, C-1’), 59.5 (CH_3_, OMe), 57.8 (CH_3_, OMe), 15.9 ppm (CH_3_, C-6). IR (CHCl_3_): ν = 3694, 3018, 2938, 1490, 1218 cm^–1^. MS (ESI) *m*/*z* (%) = 461 (100) [M + Na]^+^. HRMS (ESI) *m*/*z*: [M + Na]^+^ calcd for C_21_H_27_NaO_8_P 461.1341; found 461.1342. Anal. calcd for C_21_H_27_O_8_P: C, 57.53; H, 6.21. Found: C, 57.62; H, 6.49.

#### *C*-(6-*O*-*tert*-Butyldiphenylsilyl-4-*O*-diphenoxyphosphoryl-2,3-di-*O*-methyl-α-d-glucopyranosyl)methanol (**94**)

Following the general procedure starting from allene **92** (1.08 g, 1.54 mmol) and purification by column chromatography (hexanes–EtOAc, 6:4), alcohol **94** (556.2 mg, 0.80 mmol, 52%) was obtained as a colorless oil: [α]_D_ = +30.3 (*c* = 0.33, CHCl_3_). ^1^H NMR (500 MHz, CDCl_3_, simulated coupling constants using DAISY) δ_H_ 7.65–7.07 (m, 20H, Ar), 4.56 (ddd, *J* = 8.5, 7.6 Hz, ^3^*J*_PH_ = 9.4 Hz, 1H, 4-H), 4.13 (ddd, *J* = 8.8, 5.7, 4.6 Hz, 1H, 1-H), 3.91 (dd, *J* = 11.0, 32.8 Hz, 1H, 6-H_b_), 3.90 (dd, *J* = 12.1, 8.8 Hz, 1H, 1’-H_b_), 3.83 (ddd, *J* = 8.5, 5.9, 2.8 Hz, 1H, 5-H), 3.76 (dd, *J* = 11.0, 5.9 Hz, 1H, 6-H_a_), 3.75 (dd, *J* = 12.1, 4.6 Hz, 1H, 1’-H_a_), 3.57 (dd, *J* = 7.9, 7.6 Hz, 1H, 3-H), 3.50 (dd, *J* = 7.9, 5.7 Hz, 1H, 2-H), 3.45 (s, 3H, OMe), 3.42 (s, 3H, OMe), 1.03 ppm (s, 9H, *^t^*Bu), 1H from OH is missing. ^13^C{^1^H} NMR (100.6 MHz, CDCl_3_) δ_C_ 150.7 (d, ^2^*J*_PC_ = 7.0 Hz, C, Ar), 150.5 (d, ^2^*J*_PC_ = 7.1 Hz, C, Ar), 133.3 (2 × C, Ar), 120.0–135.7 (20 × CH, Ar), 80.6 (CH, C-2 or C-3), 80.4 (CH, C-2 or C-3), 75.7 (d, ^2^*J*_PC_ = 6.3 Hz, CH, C-4), 73.3 (d, ^3^*J*_PC_ = 6.3 Hz, CH, C-5), 72.6 (CH, C-1), 62.9 (CH_2_, C-6), 59.9 (CH_3_, OMe), 59.3 (CH_2_, C-1’), 58.8 (CH_3_, OMe), 26.8 (3 × CH_3_, DPS), 19.2 ppm (C, DPS). IR (CHCl_3_): ν = 3676, 3532, 3016, 2934, 1591, 1490 cm^–1^. MS (ESI) *m*/*z* (%) = 715 (100) [M + Na]^+^. HRMS (ESI) *m*/*z*: [M + Na]^+^ calcd for C_37_H_45_NaO_9_PSi 715.2468; found 715.2469. Anal. calcd for C_37_H_45_O_9_PSi: C, 64.14; H, 6.55. Found: C, 64.06; H, 6.36.

#### *C*-(4-*O*-Benzyl-2,3-di-*O*-methyl-α-l-rhamnopyranosyl)methanol (**104**)

Allene **103** (154 mg, 0.49 mmol) in TFA/H_2_O (4.5 mL, 4:6) was stirred at room temperature for 2 h. The solution was evaporated in a high vacuum rotovap, poured over a saturated aqueous solution of NaHCO_3_, extracted with CH_2_Cl_2_, dried over Na_2_SO_4_, and concentrated. The crude was dissolved in dry DMF (5.9 mL) under a N_2_ atmosphere, and NaH 60% in mineral oil (58.8 mg, 1.47 mmol) was slowly added at 0 °C. After 20 min, MeI (122 μL, 1.96 mmol) was dropwise added and stirring was continued at 0 °C for 1 h. Ice-water was used to destroy the excess of NaH, and the mixture was evaporated in a high vacuum rotovap, poured over a saturated solution of NH_4_Cl, and extracted with CH_2_Cl_2_. The combined organic extracts were dried over Na_2_SO_4_ and concentrated to dryness under reduced pressure. The organic residue was then submitted to the general procedure for the synthesis of hydroxymethyl derivatives. Column chromatography of the residue (hexanes–EtOAc, 4:6) gave **104** (65.4 mg, 0.22 mmol, 45%, three steps) as a colorless oil: [α]_D_ = −22.4 (*c* = 0.293, CHCl_3_). ^1^H NMR (500 MHz, CDCl_3_) δ_H_ 7.37–7.29 (m, 5H, Ar), 4.70 (d, *J* = 11.7 Hz, 1H, OBn), 4.65 (d, *J* = 11.7 Hz, 1H, OBn), 3.99 (m, 1H, 1-H), 3.89 (dddd, *J* = 6.6, 6.6, 6.6, 4.7 Hz, 1H, 5-H), 3.80 (dd, *J* = 11.7, 7.0 Hz, 1H, 1’-H_b_), 3.70 (dd, *J* = 11.4, 4.5 Hz, 1H, 1’-H_a_), 3.60–3.56 (m, 2H, 2-H, 3-H), 3.52 (dd, *J* = 5.4, 4.7 Hz, 1H, 4-H), 3.45 (s, 3H, OMe), 3.44 (s, 3H, OMe), 2.07 (br s, 1H, OH), 1.34 ppm (d, *J* = 7.0 Hz, 3H, 6-H_3_). ^13^C{^1^H} NMR (125.7 MHz, CDCl_3_) δ_C_ 138.2 (C, Ar), 128.4 (2 × CH, Ar), 127.8 (3 × CH, Ar), 78.4 (CH, C-2 or C-3), 78.0 (CH, C-4), 75.7 (CH, C-2 or C-3), 73.4 (CH_2_, OBn), 70.7 (CH, C-1), 70.6 (CH, C-5), 61.7 (CH_2_, C-1’), 58.0 (CH_3_, OMe), 57.4 (CH_3_, OMe), 17.2 ppm (CH_3_, C-6). IR (CHCl_3_): ν = 3587, 3478, 2587, 2478, 2015, 2935, 1455, 1088 cm^–1^. MS (ESI) *m*/*z* (%) = 319 (100) [M + Na]^+^. HRMS (ESI) *m*/*z*: [M + Na]^+^ calcd for C_16_H_24_NaO_5_ 319.1521; found 319.1525. Anal. calcd for C_16_H_24_O_5_: C, 64.84; H, 8.16. Found: C, 64.61; H, 8.37.

#### *C*-(4-*O*-Benzyl-2,3-di-*O*-methyl-α-d-fucopyranosyl)methanol (**109**)

Alcohol **108** (251.4 mg, 0.84 mmol) was dissolved in dry DMF (10 mL) under a N_2_ atmosphere, and NaH 60% in mineral oil (50.4 mg, 1.26 mmol) was slowly added at 0 °C. After 20 min, BnBr (200 μL, 1.68 mmol) was dropwise added and stirring was continued at 0 °C for 2 h. Ice-water was used to destroy the NaH in excess, and the mixture was evaporated in a high vacuum rotovap, poured over a saturated solution of NH_4_Cl, and extracted with CH_2_Cl_2_. The combined extracts were dried over Na_2_SO_4_ and concentrated to dryness under reduced pressure. The crude was dissolved in TFA/H_2_O (7.7 mL, 4:6) and stirred at 40 °C overnight. The solution was evaporated in a high vacuum rotovap, quenched with a saturated aqueous solution of NaHCO_3_, and extracted with CH_2_Cl_2_. The organic residue was submitted to methyl protection by treatment with NaH 60% (100.8 mg, 2.52 mmol) and MeI (209 μL, 3.36 mmol) in DMF (10 mL) for 2 h from 0 °C until room temperature. Ice-water was used to destroy the excess of NaH, and the mixture was evaporated in a high vacuum rotovap, poured over a saturated solution of NH_4_Cl, and extracted with CH_2_Cl_2_. The organic phase was dried over Na_2_SO_4_ and concentrated to dryness under reduced pressure. Finally, the crude was submitted to the general procedure to give hydroxymethyl derivatives. Column chromatography of the residue (hexanes–EtOAc, 4:6) gave **109** (106.6 mg, 0.36 mmol, 43%) as a colorless oil: [α]_D_ = −30.2 (*c* = 1.35, CHCl_3_). ^1^H NMR (400 MHz, CDCl_3_, simulated ring coupling constants using DAISY) δ_H_ 7.39–7.27 (m, 5H, Ar), 4.77 (d, *J* = 11.8 Hz, 1H, OBn), 4.63 (d, *J* = 11.8 Hz, 1H, OBn), 4.13 (ddd, *J* = 7.9, 4.7, 4.2 Hz, 1H, 1-H), 3.97 (dddd, *J* = 6.7, 6.7, 6.7, 3.6 Hz, 1H, 5-H), 3.83 (dd, *J* = 11.6, 7.9 Hz, 1H, 1’-H_b_), 3.76 (dd, *J* = 3.6, 2.9 Hz, 1H, 4-H), 3.74 (dd, *J* = 11.6, 4.7 Hz, 1H, 1’-Ha), 3.69 (dd, *J* = 7.0, 4.2 Hz, 1H, 2-H), 3.53 (dd, *J* = 7.0, 2.9 Hz, 1H, 3-H), 3.50 (s, 3H, OMe), 3.45 (s, 3H, OMe), 2.11 (br s, 1H, OH), 1.28 ppm (d, *J* = 6.7 Hz, 3H, 6-H_3_). ^13^C{^1^H} NMR (100.6 MHz, CDCl_3_) δ_C_ 138.4 (C, Ar), 128.3 (2 × CH, Ar), 128.0 (2 × CH, Ar), 127.7 (CH, Ar), 79.4 (CH, C-3), 78.1 (CH, C-2), 75.0 (CH, C-4), 73.4 (CH_2_, OBn), 70.7 (CH, C-1), 69.4 (CH, C-5), 60.8 (CH_2_, C-1’), 58.9 (CH_3_, OMe), 58.6 (CH_3_, OMe), 15.5 ppm (CH_3_, C-6). IR (CHCl_3_): ν = 3676, 3588, 3012, 2937, 1101 cm^–1^. MS (ESI) *m*/*z* (%) = 319 (100) [M + Na]^+^. HRMS (ESI) *m*/*z*: [M + Na]^+^ calcd for C_16_H_24_NaO_5_ 319.1521; found 319.1519. Anal. calcd for C_16_H_24_O_5_: C, 64.84; H, 8.16; found: C, 64.89; H, 8.20.

### General Procedure for the Synthesis of Phthalimide Derivatives **16**, **17**, **18**, **19**, **20**, **21**, **22**, **23**, **24**, and **105**

DEAD (394 μL, 2.50 mmol) was added dropwise to a stirred solution of the alcohol (1 mmol), *N-*hydroxyphthalimide (408 mg, 2.5 mmol), and PPh_3_ (656 mg, 2.5 mmol) in dry THF (10 mL), and the resulting solution was stirred at 0 °C for 0.5–2.5 h. Then, the solvent was removed and the crude was quenched with water and extracted with CHCl_3_. The combined organic extracts were dried over Na_2_SO_4_ and concentrated under reduced pressure. Column chromatography of the residue (hexanes–EtOAc) gave the corresponding phthalimides.

#### *C*-(4-*O*-Acetyl-6-*O*-*tert*-Butyldiphenylsilyl-2,3-di-*O*-methyl-α-d-glucopyranosyl)*N*-methoxyphthalimide (**16**)

Following the general procedure starting from alcohol **48** (332 mg, 0.66 mmol) and purification by column chromatography (hexanes–EtOAc, 8:2), product **16** (281 mg, 0.43 mmol, 66%) was obtained as an amorphous solid: [α]_D_ = +10.4 (*c* = 0.53, CHCl_3_). ^1^H NMR (500 MHz, CDCl_3_, simulated coupling constants using DAISY) δ_H_ 7.74–7.62 (m, 8H, Ar), 7.43–7.34 (m, 6H, Ar), 5.17 (dd, *J* = 6.8, 6.5 Hz, 1H, 4-H), 4.58 (dd, *J* = 10.8, 7.5 Hz, 1H, 1’-H_b_), 4.48 (ddd, *J* = 7.5, 4.6, 3.9 Hz, 1H, 1-H), 4.37 (dd, *J* = 10.8, 3.9 Hz, 1H, 1’-H_a_), 3.88 (ddd, *J* = 6.8, 5.0, 4.6 Hz, 1H, 5-H), 3.77 (dd, *J* = 11.0, 4.6 Hz, 1H, 6-H_b_), 3.71 (dd, *J* = 11.0, 5.0 Hz, 1H, 6-H_a_), 3.52 (dd, *J* = 7.1, 4.6 Hz, 1H, 2-H), 3.51 (s, 3H, OMe), 3.49 (s, 3H, OMe), 3.46 (dd, *J* = 7.1, 6.5 Hz, 1H, 3-H), 2.03 (s, 3H, OAc), 1.03 ppm (s, 9H, *^t^*Bu). ^13^C{^1^H} NMR (125.7 MHz, CDCl_3_) δ_C_ 169.7 (C, OAc), 163.1 (2 × C, CO), 135.64 (2 × CH, Ar), 135.60 (2 × CH, Ar), 134.3 (2 × CH, Ar), 133.4 (C, Ar), 133.3 (C, Ar), 129.5 (CH, Ar), 129.4 (CH, Ar), 128.8 (2 × C, Ar), 127.5 (4 × CH, Ar), 123.4 (2 × CH, Ar), 78.3 (CH, C-2 or C-3), 78.0 (CH, C-2 or C-3), 74.5 (CH_2_, C-1’), 73.6 (CH, C-5), 70.3 (CH, C-1), 68.2 (CH, C-4), 62.3 (CH_2_, C-6), 59.3 (CH_3_, OMe), 58.7 (CH_3_, OMe), 26.7 (3 × CH_3_, DPS), 21.0 (CH_3_, OAc), 19.1 ppm (C, DPS). IR (CHCl_3_): ν = 2934, 1792, 1735, 1236 cm^–1^. MS (ESI) *m*/*z* (%) = 670 (100) [M + Na]^+^. HRMS (ESI) *m*/*z*: [M + Na]^+^ calcd for C_35_H_41_NNaO_9_Si 670.2448; found 670.2441. Anal. calcd for C_35_H_41_NO_9_Si: C, 64.89; H, 6.38; N, 2.16. Found: C, 65.05; H, 6.40; N, 2.18.

#### *C*-(6-*O*-*tert*-Butyldiphenylsilyl-4-*O*-diphenoxyphosphoryl-2,3-di-*O*-methyl-α-d-glucopyranosyl)*N*-methoxyphthalimide (**17**)

Following the general procedure starting from alcohol **94** (507.2 mg, 0.73 mmol) and purification by column chromatography (hexanes–EtOAc, 75:25), product **17** (331.3 mg, 0.40 mmol, 54%) was obtained as a an amorphous solid: [α]_D_ = +14.9 (*c* = 0.47, CHCl_3_). ^1^H NMR (500 MHz, CDCl_3_, simulated coupling constants using DAISY) δ_H_ 7.73–7.11 (m, 24H, Ar), 4.87 (ddd, *J* = 7.0, 7.0 Hz, ^3^*J*_PH_ = 9.4 Hz, 1H, 4-H), 4.58 (dd, *J* = 10.9, 7.6 Hz, 1H, 1’-H_b_), 4.48 (ddd, *J* = 7.6, 5.3, 3.6 Hz, 1H, 1-H), 4.36 (dd, *J* = 10.9, 3.6 Hz, 1H, 1’-H_a_), 3.95 (ddd, *J* = 7.0, 4.0, 4.0 Hz, 1H, 5-H), 3.77 (dd, *J* = 4.0, 4.0 Hz, 2H, 6-H_2_), 3.60 (dd, *J* = 7.4, 7.0 Hz, 1H, 3-H), 3.51 (dd, *J* = 7.4, 5.3 Hz, 1H, 2-H), 3.48 (s, 3H, OMe), 3.44 (s, 3H, OMe), 1.00 ppm (s, 9H, *^t^*Bu). ^13^C{^1^H} NMR (100.6 MHz, CDCl_3_) δ_C_ 163.2 (2 × C, CO), 150.7 (d, ^2^*J*_PC_ = 7.0 Hz, C, Ar), 150.6 (d, ^2^*J*_PC_ = 7.8 Hz, C, Ar), 133.5 (C, Ar), 133.4 (C, Ar), 128.8 (2 × C, Ar), 119.9–135.7 (24 × CH, Ar), 79.3 (CH, C-3), 78.8 (CH, C-2), 74.5 (d, ^2^*J*_PC_ = 7.0 Hz, CH, C-4), 74.0 (CH_2_, C-1’), 73.7 (d, ^3^*J*_PC_ = 7.1 Hz, CH, C-5), 70.6 (CH, C-1), 62.1 (CH_2_, C-6), 59.6 (CH_3_, OMe), 58.7 (CH_3_, OMe), 26.7 (3 × CH_3_, DPS), 19.2 ppm (C, DPS). IR (CHCl_3_): ν = 2934, 1792, 1735, 1490, 1190 cm^–1^. MS (ESI) *m*/*z* (%) = 860 (100) [M + Na]^+^. HRMS (ESI) *m*/*z*: [M + Na]^+^ calcd for C_45_H_48_NNaO_11_PSi 860.2632; found 860.2628. Anal. calcd for C_45_H_48_NO_11_PSi: C, 64.50; H, 5.77; N, 1.67. Found: C, 64.42; H, 5.61; N, 1.82.

#### *C*-(6-*O*-*tert*-Butyldiphenylsilyl-2,3-di-*O*-methyl-4-*O*-tosyl-α-d-glucopyranosyl)*N*-methoxyphthalimide (**18**)

Following the general procedure starting from alcohol **49** (124.5 mg, 0.20 mmol) and purification by column chromatography (hexanes–EtOAc, 75:25), phthalimide **18** (121.5 mg, 0.16 mmol, 80%) was obtained as a colorless oil: [α]_D_ = +5.8 (*c* = 0.36, CHCl_3_). ^1^H NMR (500 MHz, CDCl_3_, simulated ring coupling constants using DAISY) δ_H_ 7.78–7.13 (m, 18H, Ar), 4.96 (dd, *J* = 6.2, 5.8 Hz, 1H, 4-H), 4.50 (dd, *J* = 11.0, 7.4 Hz, 1H, 1’-H_b_), 4.39 (ddd, *J* = 7.4, 4.3, 3.8 Hz, 1H, 1-H), 4.32 (dd, *J* = 11.0, 3.8 Hz, 1H, 1’-H_a_), 3.86–3.80 (m, 2H, 5-H, 6-H_b_), 3.71 (m, 1H, 6-H_a_), 3.60 (dd, *J* = 6.4, 5.8 Hz, 1H, 3-H), 3.51 (s, 3H, OMe), 3.48 (dd, *J* = 6.4, 4.3 Hz, 1H, 2-H), 3.36 (s, 3H, OMe), 2.35 (s, 3H, OTs), 1.05 ppm (s, 9H, *^t^*Bu). ^13^C{^1^H} NMR (100.6 MHz, CDCl_3_) δ_C_ 163.1 (2 × C, CO), 144.3 (C, OTs), 134.3 (C, OTs), 133.5 (C, Ar), 133.2 (C, Ar), 128.7 (2 × C, Ar), 123.4–135.6 (18 × CH, Ar), 77.8 (CH, C-2), 77.6 (CH, C-3), 74.8 (CH_2_, C-1’), 74.7 (CH, C-4), 73.5 (CH, C-5), 69.9 (CH, C-1), 61.7 (CH_2_, C-6), 59.3 (CH_3_, OMe), 58.5 (CH_3_, OMe), 26.7 (3 × CH_3_, DPS), 21.5 (CH_3_, OTs), 19.2 ppm (C, DPS). IR (CHCl_3_): ν = 3024, 2934, 1792, 1735, 1215, 1106 cm^–1^. MS (ESI) *m*/*z* (%) = 782 (100) [M + Na]^+^. HRMS (ESI) *m*/*z*: [M + Na]^+^ calcd for C_40_H_45_NNaO_10_SSi 782.2431; found 782.2434. Anal. calcd for C_40_H_45_NO_10_SSi: C, 63.22; H, 5.97; N, 1.84; S, 4.22. Found: C, 63.35; H, 6.17; N, 1.75; S, 4.43.

#### *C*-(4,6-Bis-*O*-diphenoxyphosphoryl-2,3-di-*O*-methyl-α-d-glucopyranosyl)*N*-methoxyphthalimide (**19**)

Following the general procedure starting from alcohol **95** (315 mg, 0.46 mmol) and purification by column chromatography (hexanes–EtOAc, 1:1), product **19** (235.8 mg, 0.28 mmol, 62%) was obtained as a colorless oil: [α]_D_ = +13.5 (*c* = 1.90, CHCl_3_). ^1^H NMR (500 MHz, CDCl_3_) δ_H_ 7.83–7.81 (m, 2H, Ar), 7.74–7.73 (m, 2H, Ar), 7.33–7.11 (m, 20H, Ar), 4.53 (ddd, *J* = 7.9, 7.9 Hz, ^3^*J*_PH_ = 7.9 Hz, 1H, 4-H), 4.49 (dd, *J* = 10.1, 6.6 Hz, 1H, 1’-H_b_), 4.42–4.33 (m, 3H, 1-H, 6-H_b_, 1’-H_a_), 4.26 (ddd, *J* = 12.3, 4.7 Hz, ^3^*J*_PH_ = 7.9 Hz, 1H, 6-H_a_), 4.14 (m, 1H, 5-H), 3.58 (dd, *J* = 7.3, 7.3 Hz, 1H, 3-H), 3.44 (s, 3H, OMe), 3.43 (s, 3H, OMe), 3.41 ppm (dd, *J* = 7.6, 4.7 Hz, 1H, 2-H). ^13^C{^1^H} NMR (125.7 MHz, CDCl_3_) δ_C_ 163.3 (2 × C, CO), 150.6 (d, ^2^*J*_PC_ = 6.3 Hz, 2 × C, Ar), 150.5 (d, ^2^*J*_PC_ = 7.4 Hz, C, Ar), 150.4 (d, ^2^*J*_PC_ = 8.5 Hz, C, Ar), 128.8 (2 × C, Ar), 120.0–134.5 (24 × CH, Ar), 79.3 (CH, C-3), 78.4 (CH, C-2), 74.4 (d, ^2^*J*_PC_ = 6.4 Hz, CH, C-4), 74.1 (CH_2_, C-1’), 72.8 (dd, ^3^*J*_PC_ = 6.1, 6.1 Hz, CH, C-5), 70.4 (CH, C-1), 66.5 (d, ^2^*J*_PC_ = 6.3 Hz, CH_2_, C-6), 59.8 (CH_3_, OMe), 58.8 ppm (CH_3_, OMe). IR (CHCl_3_): ν = 3018, 2937, 1736, 1214 cm^–1^. MS (ESI) *m*/*z* (%) = 854 (100) [M + Na]^+^. HRMS (ESI) *m*/*z:* [M + Na]^+^ calcd for C_41_H_39_NNaO_14_P_2_: 854.1744; found 854.1743. Anal. calcd for C_41_H_39_NO_14_P_2_: C, 59.21; H, 4.73; N, 1.68. Found: C, 58.99; H, 4.89; N, 2.08.

#### *C*-(4-*O*-Acetyl-6-*O*-*tert*-butyldiphenylsilyl-2,3-di-*O*-methyl-α-d-galactopyranosyl)*N*-methoxyphthalimide (**20**)

Following the general procedure starting from alcohol **51** (285.9 mg, 0.57 mmol) and purification by column chromatography (hexanes–EtOAc, 75:25), product **20** (318.2 mg, 0.49 mmol, 86%) was obtained as a crystalline solid: mp 48.9–50.0 °C (*n-*hexane–EtOAc); [α]_D_ = +6.7 (*c* = 0.43, CHCl_3_). ^1^H NMR (500 MHz, CDCl_3_, simulated ring coupling constants using DAISY) δ_H_ 7.74–7.61 (m, 8H, Ar), 7.43–7.35 (m, 6H, Ar), 5.68 (dd, *J* = 3.2, 2.1 Hz, 1H, 4-H), 4.62–4.58 (m, 2H, 1-H, 1’-H_b_), 4.38 (m, 1H, 1’-H_a_), 4.09 (ddd, *J* = 8.1, 5.7, 2.1 Hz, 1H, 5-H), 3.69 (dd, *J* = 9.1, 5.4 Hz, 1H, 2-H), 3.64 (dd, *J* = 10.0, 5.7 Hz, 1H, 6-H_b_), 3.54 (dd, *J* = 10.0, 8.1 Hz, 1H, 6-H_a_), 3.50 (s, 3H, OMe), 3.46 (dd, *J* = 9.1, 3.2 Hz, 1H, 3-H), 3.44 (s, 3H, OMe), 2.02 (s, 3H, OAc), 1.03 ppm (s, 9H, *^t^*Bu). ^13^C{^1^H} NMR (125.7 MHz, CDCl_3_) δ_C_ 169.7 (C, OAc), 163.2 (2 × C, CO), 135.60 (2 × CH, Ar), 135.56 (2 × CH, Ar), 134.4 (2 × CH, Ar), 133.3 (C, Ar), 133.1 (C, Ar), 129.7 (CH, Ar), 129.6 (CH, Ar), 128.8 (2 × C, Ar), 127.67 (2 × CH, Ar), 127.66 (2 × CH, Ar), 123.4 (2 × CH, Ar), 78.9 (CH, C-3), 76.3 (CH, C-2), 72.8 (CH_2_, C-1’), 72.2 (CH, C-1), 71.5 (CH, C-5), 66.1 (CH, C-4), 61.1 (CH_2_, C-6), 59.3 (CH_3_, OMe), 57.7 (CH_3_, OMe), 26.7 (3 × CH_3_, DPS), 20.8 (CH_3_, OAc), 19.0 ppm (C, DPS). IR (CHCl_3_): ν = 2933, 1792, 1734, 1226 cm^–1^. MS (ESI) *m*/*z* (%) = 670 (100) [M + Na]^+^. HRMS (ESI) *m*/*z:* [M + Na]^+^ calcd for C_35_H_41_NNaO_9_Si: 670.2448; found 670.2443. Anal. calcd for C_35_H_41_NO_9_Si: C, 64.89; H, 6.38; N, 2.16. Found: C, 64.67; H, 6.41; N, 2.23.

#### *C*-(6-*O*-*tert*-Butyldiphenylsilyl-4-*O*-diphenoxyphosphoryl-2,3-di-*O*-methyl-α-d-galactopyranosyl)*N*-methoxyphthalimide (**21**)

Following the general procedure starting from alcohol **52** (510 mg, 0.74 mmol) (327 mg, 0.51 mmol) and purification by column chromatography (hexanes–EtOAc, 8:2), product **21** (574 mg, 0.68 mmol, 93%) was obtained as a colorless oil: [α]_D_ = −9.1 (*c* = 0.67, CHCl_3_). ^1^H NMR (500 MHz, CDCl_3_, simulated ring coupling constants using DAISY) δ_H_ 7.74–7.11 (m, 24H, Ar), 5.24 (ddd, *J* = 2.9, 2.7 Hz, ^3^*J*_PH_ = 8.7 Hz, 1H, 4-H), 4.47–4.41 (m, 2H, 1-H, 1’-H_b_), 4.33 (m, 1H, 1’-H_a_), 4.03 (dddd, *J* = 6.7, 5.8, 2.7 Hz, ^4^*J*_PH_ = 2.3 Hz, 1H, 5-H), 3.77 (dd, *J* = 10.6, 5.8 Hz, 1H, 6-H_b_), 3.74 (dd, *J* = 10.6, 6.7 Hz, 1H, 6-H_a_), 3.66 (dd, *J* = 8.2, 5.6 Hz, 1H, 2-H), 3.42 (s, 3H, OMe), 3.39 (dd, *J* = 8.2, 2.9 Hz, 1H, 3-H), 3.35 (s, 3H, OMe), 1.00 ppm (s, 9H, *^t^*Bu). ^13^C{^1^H} NMR (100.6 MHz, CDCl_3_) δ_C_ 163.2 (2 × C, CO), 150.7 (d, ^2^*J*_PC_ = 7.4 Hz, C, Ar), 150.6 (d, ^2^*J*_PC_ = 6.4 Hz, C, Ar), 135.58 (2 × CH, Ar), 135.55 (2 × CH, Ar), 134.4 (2 × CH, Ar), 133.5 (C, Ar), 133.3 (C, Ar), 129.7 (2 × CH, Ar), 129.6 (CH, Ar), 129.5 (CH, Ar), 129.4 (2 × CH, Ar), 128.8 (2 × C, Ar), 127.7 (2 × CH, Ar), 127.6 (2 × CH, Ar), 125.1 (CH, Ar), 125.0 (CH, Ar), 123.4 (2 × CH, Ar), 120.4 (CH, Ar), 120.3 (CH, Ar), 120.1 (CH, Ar), 120.0 (CH, Ar), 78.3 (CH, C-3), 75.7 (CH, C-2), 73.8 (d, ^2^*J*_PC_ = 6.3 Hz, CH, C-4), 73.5 (CH_2_, C-1’), 73.1 (CH, C-1), 70.6 (br s, CH, C-5), 60.9 (CH_2_, C-6), 59.5 (CH_3_, OMe), 57.8 (CH_3_, OMe), 26.7 (3 × CH_3_, DPS), 19.1 ppm (C, DPS). IR (CHCl_3_): ν = 3015, 2933, 1792, 1730, 1490, 1190 cm^–1^. MS (ESI) *m*/*z* (%) = 860 (100) [M + Na]^+^. HRMS (ESI) *m*/*z:* [M + Na]^+^ calcd for C_45_H_48_NNaO_11_PSi: 860.2632; found 860.2648. Anal. calcd for C_45_H_48_NO_11_PSi: C, 64.50; H, 5.77; N, 1.67. Found: C, 64.82; H, 6.01; N, 1.92.

#### *C*-(4-*O*-Diphenoxyphosphoryl-2,3-di-*O*-methyl-α-l-rhamnopyranosyl)*N*-methoxyphthalimide (**22**)

Alcohol **105** (231.7 mg, 0.66 mmol) in dry CH_2_Cl_2_ (38 mL) was treated with DMAP (371 mg, 3.04 mmol) and ClPO(OPh)_2_ (0.63 mL, 3.04 mmol) for 2 h. The reaction was quenched with a saturated aqueous solution of NH_4_Cl and extracted with CH_2_Cl_2_. The organic phase was dried over Na_2_SO_4_, filtered, and evaporated. Column chromatography of the residue (hexanes–EtOAc, 6:4) gave the compound **22** (333 mg, 0.55 mmol, 84%) as an amorphous solid: [α]_D_ = −0.1 (*c* = 0.41, CHCl_3_). ^1^H NMR (500 MHz, CDCl_3_, simulated ring coupling constants using DAISY) δ_H_ 7.84–7.82 (m, 2H, Ar), 7.75–7.73 (m, 2H, Ar), 7.37–7.17 (m, 10H, Ar), 4.63 (ddd, *J* = 5.2, 3.2 Hz, ^3^*J*_PH_ = 9.2 Hz, 1H, 4-H), 4.46 (dd, *J* = 10.9, 3.2 Hz, 1H, 1’-H_b_), 4.38 (dd, *J* = 10.9, 6.2 Hz, 1H, 1’-H_a_), 4.23 (ddd, *J* = 7.6, 6.2, 3.2 Hz, 1H, 1-H), 4.01 (dddd, *J* = 6.9, 6.9, 6.9, 3.7 Hz, 1H, 5-H), 3.71 (dd, *J* = 5.2, 3.2 Hz, 1H, 3-H), 3.61 (dd, *J* = 7.6, 3.2 Hz, 1H, 2-H), 3.42 (s, 3H, OMe), 3.35 (s, 3H, OMe), 1.34 ppm (d, *J* = 6.9 Hz, 3H, 6-H_3_). ^13^C{^1^H} NMR (125.7 MHz, CDCl_3_) δ_C_ 163.2 (2 × C, CO), 150.5 (d, ^2^*J*_PC_ = 7.4 Hz, C, Ar), 150.3 (d, ^2^*J*_PC_ = 7.4 Hz, C, Ar), 134.4 (2 × CH, Ar), 129.8 (2 × CH, Ar), 129.7 (2 × CH, Ar), 128.9 (2 × C, Ar), 125.42 (CH, Ar), 125.37 (CH, Ar), 123.4 (2 × CH, Ar), 120.2 (CH, Ar), 120.12 (CH, Ar), 120.11 (CH, Ar), 120.07 (CH, Ar), 77.4 (d, ^2^*J*_PC_ = 6.4 Hz, CH, C-4), 77.2 (CH_2_, C-1’), 76.2 (d, ^3^*J*_PC_ = 3.2 Hz, CH, C-3), 73.9 (CH, C-2), 71.6 (d, ^3^*J*_PC_ = 5.3 Hz, CH, C-5), 68.2 (CH, C-1), 58.3 (CH_3_, OMe), 57.2 (CH_3_, OMe), 16.2 ppm (CH_3_, C-6). IR (CHCl_3_): ν = 3015, 2939, 1792, 1736, 1490, 1209 cm^–1^. MS (ESI) *m*/*z* (%) = 606 (100) [M + Na]^+^. HRMS (ESI) *m*/*z:* [M + Na]^+^ calcd for C_29_H_30_NNaO_10_P: 606.1505; found 606.1516. Anal. calcd for C_29_H_30_NO_10_P: C, 59.69; H, 5.18; N, 2.40. Found: C, 59.37; H, 5.40; N, 2.68.

#### *C*-(4-*O*-Acetyl-2,3-di-*O*-methyl-α-l-fucopyranosyl)*N*-methoxyphthalimide (**23**)

Following the general procedure starting from **58** (65.6 mg, 0.26 mmol) and purification by column chromatography (hexanes–Et_2_O, 4:6), product **23** (52.4 mg, 0.13 mmol, 51%) was obtained as a white crystalline solid: mp 37.8–38.5 °C (*n-*hexane–EtOAc). [α]_D_ = −42.5 (*c =* 0.72, CHCl_3_). ^1^H NMR (500 MHz, CDCl_3_, simulated ring coupling constants using DAISY) δ_H_ 7.86–7.84 (m, 2H, Ar), 7.78–7.76 (m, 2H, Ar), 5.31 (dd, *J* = 3.4, 2.0 Hz, 1H, 4-H), 4.67 (ddd, *J* = 9.0, 5.9, 1.9 Hz, 1H, 1-H), 4.63 (dd, *J* = 10.7, 9.0 Hz, 1H, 1’-H_b_), 4.39 (dd, *J* = 10.7, 1.9 Hz, 1H, 1’-H_a_), 4.09 (dddd, *J* = 6.4, 6.4, 6.4, 2.0 Hz, 1H, 5-H), 3.71 (dd, *J* = 9.3, 5.9 Hz, 1H, 2-H), 3.51 (s, 3H, OMe), 3.40 (s, 3H, OMe), 3.36 (dd, *J* = 9.3, 3.4 Hz, 1H, 3-H), 2.16 (s, 3H, OAc), 1.11 ppm (d, *J* = 6.4 Hz, 3H, 6-H_3_). ^13^C{^1^H} NMR (125.7 MHz, CDCl_3_) δ_C_ 170.6 (C, OAc), 163.3 (2 × C, CO), 134.5 (2 × CH, Ar), 128.8 (2 × C, Ar), 123.5 (2 × CH, Ar), 78.6 (CH, C-3), 76.1 (CH, C-2), 72.8 (CH_2_, C-1’), 71.8 (CH, C-1), 69.4 (CH, C-4), 67.0 (CH, C-5), 59.2 (CH_3_, OMe), 57.6 (CH_3_, OMe), 20.8 (CH_3_, OAc), 16.1 ppm (CH_3_, C-6). IR (CHCl_3_): ν = 3018, 2936, 1791, 1744, 1239 cm^–1^. MS (ESI) *m*/*z* (%) = 416 (100) [M + Na]^+^. HRMS (ESI) *m*/*z:* [M + Na]^+^ calcd for C_19_H_23_NNaO_8_: 416.1321; found 416.1328. Anal. calcd for C_19_H_23_NO_8_: C, 58.01; H, 5.89; N, 3.56. Found: C, 58.33; H, 5.95; N, 3.83.

#### *C*-(4-*O*-Diphenoxyphosphoryl-2,3-di-*O*-methyl-α-l-fucopyranosyl)*N*-methoxyphthalimide (**24**)

Following the general procedure starting from **59** (232 mg, 0.53 mmol) and purification by column chromatography (hexanes–EtOAc, 6:4), product **24** (52.4 mg, 0.13 mmol, 51%) was obtained as a colorless oil: [α]_D_ = −19.0 (*c =* 0.57, CHCl_3_). ^1^H NMR (500 MHz, CDCl_3_, simulated ring coupling constants using DAISY) δ_H_ 7.84–7.81 (m, 2H, Ar), 7.76–7.74 (m, 2H, Ar), 7.34–7.16 (m, 10H, Ar), 4.91 (ddd, *J* = 2.5, 3.3 Hz, ^3^*J*_PH_ = 9.0 Hz, 1H, 4-H), 4.55–4.50 (m, 2H, 1-H, 1’-H_b_), 4.35 (m, 1H, 1’-H_a_), 4.08 (ddddd, *J* = 6.5, 6.5, 6.5, 2.5 Hz, ^4^*J*_PH_ = 2.0 Hz, 1H, 5-H), 3.61 (dd, *J* = 7.9, 5.0 Hz, 1H, 2-H), 3.43 (dd, *J* = 7.9, 3.3 Hz, 1H, 3-H), 3.42 (s, 3H, OMe), 3.39 (s, 3H, OMe), 1.19 ppm (d, *J* = 6.5 Hz, 3H, 6-H_3_). ^13^C{^1^H} NMR (125.7 MHz, CDCl_3_) δ_C_ 163.3 (2 × C, CO), 150.7 (d, ^2^*J*_PC_ = 7.4 Hz, C, Ar), 150.5 (d, ^2^*J*_PC_ = 6.4 Hz, C, Ar), 134.5 (2 × CH, Ar), 129.7 (2 × CH, Ar), 129.5 (2 × CH, Ar), 128.8 (2 × C, Ar), 125.2 (CH, Ar), 125.1 (CH, Ar), 123.5 (2 × CH, Ar), 120.3 (CH, Ar), 120.2 (CH, Ar), 120.1 (CH, Ar), 120.0 (CH, Ar), 78.4 (CH, C-3), 76.5 (d, ^2^*J*_PC_ = 6.4 Hz, CH, C-4), 75.9 (CH, C-2), 73.8 (CH_2_, C-1’), 70.3 (CH, C-1), 68.1 (d, ^3^*J*_PC_ = 6.3 Hz, CH, C-5), 59.4 (CH_3_, OMe), 57.9 (CH_3_, OMe), 15.4 ppm (CH_3_, C-6). IR (CHCl_3_): ν = 3026, 2938, 1792, 1734, 1210 cm^–1^. MS (ESI) *m*/*z* (%) = 606 (100) [M + Na]^+^. HRMS (ESI) *m*/*z:* [M + Na]^+^ calcd for C_29_H_30_NNaO_10_P: 606.1505; found 606.1500. Anal. calcd for C_29_H_30_NO_10_P: C, 59.69; H, 5.18; N, 2.40. Found: C, 59.61; H, 5.23; N, 2.70.

#### *C*-(2,3-Di-*O*-methyl-α-l-rhamnopyranosyl)*N*-methoxyphthalimide (**105**)

To a solution of **104** (570 mg, 1.92 mmol) in dry EtOAc (45 mL) was added Pd/C 10% (430 mg), and the mixture was submitted to a H_2_ atmosphere overnight. The reaction was filtered over a pad of Celite and evaporated. The residue was then submitted to the general procedure to give phthalimide **105** (383 mg, 1.09 mmol, 57%) as a colorless oil: [α]_D_ = −22.6 (*c* = 1.34, CHCl_3_). ^1^H NMR (500 MHz, CDCl_3_) δ_H_ 7.84–7.82 (m, 2H, Ar), 7.76–7.74 (m, 2H, Ar), 4.53 (dd, *J* = 11.0, 4.9 Hz, 1H, 1’-H_b_), 4.32 (dd, *J* = 11.0, 4.7 Hz, 1H, 1’-H_a_), 4.24 (ddd, *J* = 4.7, 4.7, 4.7 Hz, 1H, 1-H), 4.07 (dd, *J* = 5.1, 3.3 Hz, 1H, 2-H), 3.70–3.67 (m, 2H, 4-H, 5-H), 3.60 (dd, *J* = 7.2, 3.3 Hz, 1H, 3-H), 3.52 (s, 3H, OMe), 3.50 (s, 3H, OMe), 1.28 ppm (d, *J* = 6.3 Hz, 3H, 6-H_3_), 1H from OH is missing. ^13^C{^1^H} NMR (125.7 MHz, CDCl_3_) δ_C_ 163.4 (2 × C, CO), 134.6 (2 × CH, Ar), 128.7 (2 × C, Ar), 123.6 (2 × CH, Ar), 79.5 (CH, C-3), 76.4 (CH_2_, C-1’), 73.6 (CH, C-2), 72.8 (CH, C-4 or C-5), 70.5 (CH, C-4 or C-5), 70.3 (CH, C-1), 57.7 (CH_3_, OMe), 57.5 (CH_3_, OMe), 16.9 ppm (CH_3_, C-6). IR (CHCl_3_): ν = 3674, 3501, 3022, 2937, 1792, 1735, 1212 cm^–1^. MS (ESI) *m*/*z* (%) = 374 (100) [M + Na]^+^. HRMS (ESI) *m*/*z:* [M + Na]^+^ calcd for C_17_H_21_NNaO_7_: 374.1216; found 374.1213. Anal. calcd for C_17_H_21_NO_7_: C, 58.11; H, 6.02; N, 3.99. Found: C, 58.22; H, 6.18; N, 4.11.

#### Methyl 4-*O*-Benzyl-6-*O*-*tert*-butyldiphenylsilyl-2,3-di-*O*-methyl-α-d-[4-*O*-PhCH-^2^H]galactopyranoside ([PhCH-^2^H]**97**)

A mixture of **96** (47.4 mg, 0.10 mmol) and benzyl α-[^2^H]-4-methylbenzenesulfonate^52^ (31.6 mg, 0.12 mmol, ^2^H/^1^H 6.9:1) in DMF (0.2 mL) and CH_2_Cl_2_ (0.2 mL) was cooled to 0 °C. Then, sodium hydride (60% dispersion in mineral oil, 8 mg, 0.20 mmol) was added to the mixture, and the reaction was gradually warmed up to room temperature. After stirring for 1 h, the Dowex 50WX4-200 was added to quench the reaction. The mixture was filtered through a pad of Celite, and the filtrate was evaporated under reduced pressure. The crude residue was purified by column chromatography (hexanes–EtOAc, 8:2 to 7:3) to obtain the product [PhCH-^2^H]**97** (28.6 mg, 0.05 mmol, 50%, ^2^H/^1^H 7:1) as a colorless oil: ^1^H NMR (500 MHz, CDCl_3_) δ_H_ 7.67–7.60 (m, 4H, Ar), 7.46–7.32 (m, 6H, Ar), 7.31–7.21 (m, 5H, Ar), 4.91 (d, *J* = 11.4 Hz, 0.1H, O-CH_2_-Ph), 4.900 (br s, 0.4H, O-CHD-Ph), 4.84 (d, *J* = 3.6 Hz, 1H, 1-H), 4.60 (d, *J* = 11.5 Hz, 0.1H, O-CH_2_-Ph), 4.589 (br s, 0.4H, O-CHD-Ph), 4.01 (br s, 1H, 4-H), 3.79–3.69 (m, 4H, 6-H_2_, 2-H, 5-H), 3.56 (dd, *J* = 10.1, 2.8 Hz, 1H, 3-H), 3.51 (s, 3H, 3-OMe), 3.51 (s, 3H, 2-OMe), 3.32 (s, 3H, 1-OMe), 1.06 ppm (s, 9H). ^13^C{^1^H} NMR (125.7 MHz, CDCl_3_) δ_C_ 138.6 (C, Ar), 135.56 (2 × CH, Ar), 135.53 (2 × CH, Ar), 133.4 (2 × C, Ar), 129.74 (CH, Ar), 129.71 (CH, Ar), 128.2 (2 × CH, Ar), 128.1 (2 × CH, Ar), 127.71 (2 × CH, Ar), 127.70 (2 × CH, Ar), 127.5 (CH, Ar), 97.7 (CH, C-1), 80.8 (CH, C-3), 78.1 (CH, C-2), 74.73 (0.12CH_2_, O-CH_2_-Ph), 74.35 (0.88CH, t, *J*_CD_ = 22.1 Hz, O-CHD-Ph), 73.64 (0.12CH, C-4), 73.59 (0.44CH, C-4), 73.56 (0.44CH, C-4), 70.7 (CH, C-5), 62.8 (CH_2_, C-6), 58. 8 (CH_3_, OMe), 58.3 (CH_3_, OMe), 55.0 (CH_3_, OMe), 26.9 (3 × CH_3_, DPS), 19.2 ppm (C, DPS). ^1^H NMR (500 MHz, C_6_D_6_) δ_H_ 7.84–7.79 (m, 2H, Ar), 7.79–7.74 (m, 2H, Ar), 7.35 (m, 2H, Ar), 7.23–7.07 (m, 9H, Ar), 5.07 (d, *J* = 11.4 Hz, 0.1H, O-CH_2_-Ph), 5.05 (br s, 0.4H, O-CHD-Ph), 4.82 (d, *J* = 3.6 Hz, 1H, 1-H), 4.61 (d, *J* = 11.4 Hz, 0.1H, O-CH_2_-Ph), 4.58 (br s, 0.4H, O-CHD-Ph), 4.105 (dd, *J* = 10.0, 6.6 Hz, 0.5H, 6-H), 4.107 (dd, *J* = 10.1, 6.5 Hz, 0.5H, 6-H), 4.06 (dd, *J* = 10.1, 3.7 Hz, 0.5H, 6-H), 4.05 (dd, *J* = 10.2, 3.8 Hz, 0.5H, 6-H), 3.97–3.90 (m, 3H, 5-H, 4-H, 2-H), 3.73 (dd, *J* = 10.0, 2.9 Hz, 1H, 3-H), 3.35 (s, 3H, 3-OMe), 3.232 (s, 1.5H, 2-OMe), 3.230 (s, 1.5H, 2-OMe), 3.21 (s, 3H, 1-OMe), 1.19 ppm (s, 9H). ^13^C{^1^H} NMR (125.7 MHz, C_6_D_6_) δ_C_ 139.98 (C, Ar), 136.41 (2 × CH, Ar), 136.31 (2 × CH, Ar), 134.33 (C, Ar), 134.16 (C, Ar), 130.42 (CH, Ar), 130.40 (CH, Ar), 98.93 (CH, C-1), 81.71 (CH, C-3), 79.40 (CH, C-2), 75.55 (0.12CH, C-4), 75.51 (0.44CH, C-4), 75.48 (0.44CH, C-4), 75.45 (0.12CH_2_, O-CH_2_-Ph), 75.08 (0.44CH, t, *J*_CD_ = 22.1 Hz, O-CHD-Ph), 75.05 (0.44CH, t, *J*_CD_ = 21.1 Hz, O-CHD-Ph), 71.89 (CH, C-5), 64.14 (CH_2_, C-6), 59.00 (CH_3_, OMe), 58.59 (CH_3_, OMe), 55.26 (CH_3_, OMe), 27.46 (3 × CH_3_, DPS), 19.81 ppm (C, DPS), some aromatic carbons were not observed. IR (CHCl_3_): ν = 3020, 2932, 1471, 1428, 1220, 1103 cm^–1^. MS (ESI) *m*/*z* (%) = 574 (100) [M + Na]^+^, 573 (13) [M + Na]^+^. HRMS (ESI) *m*/*z:* [M + Na]^+^ calcd for C_32_H_41_^2^HNaO_6_Si 574.2711; found 574.2716; [M + Na]^+^ calcd for C_32_H_42_NaO_6_Si 573.2648; found 573.2653.

## Supporting Information Available

Tables of calculated ring coupling constants (^3^*J*_H,H_) of starting phthalimides and final bicyclic ketals; calculated long-range ^4^*J*_w_ coupling constants; selected signals of ^1^H and ^13^C{1H} NMR spectra of labeled [PhCH-^2^H]**31** and [PhCH-^2^H]**97**; reactivity differences between LGs in the 1,5-hydrogen atom transfer/Surzur–Tanner rearrangement sequence; and copies of the ^1^H and ^13^C{1H} NMR spectra of all new compounds (PDF)

## References

[ref1] aGillard R. M.; Brimble M. A. Benzannulated Spiroketal Natural Products: Isolation, Biological Activity, Biosynthesis, and Total Synthesis. Org. Biomol. Chem. 2019, 17, 8272–8307. 10.1039/C9OB01598A. 31478048

[ref2] aZhang W.; Tong R. Synthetic Approaches To Construct the 6,8-DOBCO Framework in Natural Products. J. Org. Chem. 2016, 81, 2203–2212. 10.1021/acs.joc.6b00246. 26954099

[ref3] aMori K. Protective Group-Free Syntheses of (±)-Frontalin, (±)-*endo*-Brevicomin, (±)-*exo*-Brevicomin, and (±)-3,4-Dehydro-*exo*-Brevicomin: Racemic Pheromones with a 6,8-Dioxabicyclo[3.2.1]Octane Ring. Biosci., Biotechnol., Biochem. 2011, 75, 976–981. 10.1271/bbb.110071. 21597189

[ref4] For a study of the abundance of bicyclic acetals in natural products, see Lenci E.; Menchi G.; Saldívar-Gonzalez F. I.; Medina-Franco J. L.; Trabocchi A. Bicyclic Acetals: Biological Relevance, Scaffold Analysis, and Applications in Diversity-Oriented Synthesis. Org. Biomol. Chem. 2019, 17, 1037–1052. 10.1039/C8OB02808G. 30620036

[ref5] For recent reviews, seeaIglesias-Arteaga M. A.; Morzycki J. W. Cephalostatins and Ritterazines. In The Alkaloids Chemistry and Biology; 2013; pp. 153–279, 10.1016/B978-0-12-407774-4.00002-9. 24712099

[ref6] Liu H.; Lin S.; Jacobsen K. M.; Poulsen T. B. Chemical Syntheses and Chemical Biology of Carboxyl Polyether Ionophores: Recent Highlights. Angew. Chem., Int. Ed. 2019, 58, 13630–13642. 10.1002/anie.201812982. 30793459

[ref7] Carroll A. R.; Copp B. R.; Davis R. A.; Keyzers R. A.; Prinsep M. R. Marine Natural Products. Nat. Prod. Rep. 2020, 37, 175–223. 10.1039/C9NP00069K. 32025684

[ref8] aBeaumont S.; Ilardi E. A.; Tappin N. D. C.; Zakarian A. Marine Toxins with Spiroimine Rings: Total Synthesis of Pinnatoxin A. Eur. J. Org. Chem. 2010, 2010, 5743–5765. 10.1002/ejoc.201000842. PMC3066085 21461316

[ref9] aRen J.; Wang J.; Tong R. Asymmetric Total Synthesis of (+)-Attenol B. Org. Lett. 2015, 17, 744–747. 10.1021/acs.orglett.5b00038. 25627786

[ref10] Somsák L., Ed. Carbohydrate-Spiro-Heterocycles; Springer International Publishing, 2019.

[ref11] aChen Y.; Wang X.; Wang J.; Yang Y. Synthesis of D-Manno-Heptulose via a Cascade Aldol/Hemiketalization Reaction. Beilstein J. Org. Chem. 2017, 13, 795–799. 10.3762/bjoc.13.79. 28546836 PMC5433184

[ref12] aMartín A.; Salazar J. A.; Suárez E. Synthesis of Chiral Spiroacetals from Carbohydrates. J. Org. Chem. 1996, 61, 3999–4006. 10.1021/jo960060g. 11667274

[ref13] Francisco C. G.; Herrera A. J.; Suárez E. Intramolecular Hydrogen Abstraction Reaction Promoted by Alkoxy Radicals in Carbohydrates. Synthesis of Chiral 2,7-Dioxabicyclo[2.2.1]Heptane and 6,8-Dioxabicyclo[3.2.1]Octane Ring Systems. J. Org. Chem. 2002, 67, 7439–7445. 10.1021/jo026004z. 12375977

[ref14] For a recent review on HAT reactions, see Nechab M.; Mondal S.; Bertrand M. P. 1,n-Hydrogen-Atom Transfer (HAT) Reactions in Which n ≠5: An Updated Inventory. Chem. - Eur. J. 2014, 20, 16034–16059. 10.1002/chem.201403951. 25345694

[ref15] For the interest of *C*-ketosides as versatile chiral synthons, seeaPérez-Martín I.; Suárez E. Radicals and Carbohydrates. In Encyclopedia of Radicals in Chemistry, Biology and Materials; John Wiley & Sons, Ltd: Chichester, UK, 2012; pp. 1131–1174, 10.1002/9781119953678.rad031.

[ref16] León E. I.; Martín Á.; Pérez-Martín I.; Quintanal L. M.; Suárez E. C-C Bond Formation by Sequential Intramolecular Hydrogen Atom Transfer/Intermolecular Radical Allylation Reaction in Carbohydrate Systems. Eur. J. Org. Chem. 2012, 2012, 3818–3829. 10.1002/ejoc.201200300.

[ref17] aTakahashi H.; Shida T.; Hitomi Y.; Iwai Y.; Miyama N.; Nishiyama K.; Sawada D.; Ikegami S. Divergent Synthesis of L-Sugars and L-Iminosugars from D-Sugars. Chem. - Eur. J. 2006, 12, 5868–5877. 10.1002/chem.200600268. 16718730

[ref18] Wang Z.; Shao H.; Lacroix E.; Wu S.-H.; Jennings H. J.; Zou W. Epimerization of 2‘-Carbonylalkyl-*C*-Glycosides via Enolation, β-Elimination and Intramolecular Cycloaddition. J. Org. Chem. 2003, 68, 8097–8105. 10.1021/jo034446k. 14535789

[ref19] aSurzur J. M.; Teissier P. Addition Radicalaire de Esters Sur Les Alcools Ethyleniques. C. R. Acad. Sci. Fr. Ser. C 1967, 264, 1981–1984.

[ref20] Tanner D. D.; Law F. C. P. Free-Radical Acetoxy Group Migration. J. Am. Chem. Soc. 1969, 91, 7535–7537. 10.1021/ja01054a068.

[ref21] aBeckwith A. L. J.; Duggan P. J. The Mechanism of the β-(Acyloxy)alkyl Radical Rearrangement: Substituent and Solvent Effects. J. Am. Chem. Soc. 1996, 118, 12838–12839. 10.1021/ja963153o.

[ref22] aCrich D.; Yao Q. The β-(Phosphonooxy)Alkyl Radical Rearrangement. J. Am. Chem. Soc. 1993, 115, 1165–1166. 10.1021/ja00056a060.

[ref23] Lacôte E.; Renaud P. Rate Enhancement of the Radical 1,2-Acyloxy Shift (Surzur-Tanner Rearrangement) by Complexation with Lewis Acids. Angew. Chem., Int. Ed. 1998, 37, 2259–2262. 10.1002/(SICI)1521-3773(19980904)37:16<2259::AID-ANIE2259>3.0.CO;2-U. 29711447

[ref24] aGiese B.; Gröninger K. S. 1,3,4,6-Tetra-*O*-Acetyl-2-Deoxy-α-D-Glucopyranose. Org. Synth. 1990, 69, 66–69. 10.15227/orgsyn.069.0066.

[ref25] Gimisis T.; Ialongo G.; Chatgilialoglu C. Generation of C-1′ Radicals through a β-(Acyloxy)Alkyl Rearrangement in Modified Purine and Pyrimidine Nucleosides. Tetrahedron 1998, 54, 573–592. 10.1016/S0040-4020(97)10317-9.

[ref26] aPaul R.; Greenberg M. M. Mechanistic Studies on RNA Strand Scission from a C2′-Radical. J. Org. Chem. 2016, 81, 9199–9205. 10.1021/acs.joc.6b01760. 27668445 PMC5055465

[ref27] aFor a recent review, see Schauer R.; Kamerling J. P. Exploration of the Sialic Acid World. Adv. Carbohydr. Chem. Biochem. 2018, 75, 1–213. 10.1016/bs.accb.2018.09.001. 30509400 PMC7112061

[ref28] aSun Z.; Winschel G. A.; Borovika A.; Nagorny P. Chiral Phosphoric Acid-Catalyzed Enantioselective and Diastereoselective Spiroketalizations. J. Am. Chem. Soc. 2012, 134, 8074–8077. 10.1021/ja302704m. 22545651

[ref29] aElshahawi S. I.; Shaaban K. A.; Kharel M. K.; Thorson J. S. A Comprehensive Review of Glycosylated Bacterial Natural Products. Chem. Soc. Rev. 2015, 44, 7591–7697. 10.1039/C4CS00426D. 25735878 PMC4560691

[ref30] aKim S.; Lee T. A.; Song Y. Facile Generation of Alkoxy Radicals from *N*-Alkoxyphthalimides. Synlett 1998, 1998, 471–472. 10.1055/s-1998-1711.

[ref31] aJia K.; Chen Y. Visible-Light-Induced Alkoxyl Radical Generation for Inert Chemical Bond Cleavage/Functionalization. Chem. Commun. 2018, 54, 6105–6112. 10.1039/C8CC02642D. 29770824

[ref32] aDorigo A. E.; McCarrick M. A.; Loncharich R. J.; Houk K. N. Transition Structures for Hydrogen Atom Transfers to Oxygen. Comparisons of Intermolecular and Intramolecular Processes, and Open- and Closed-Shell Systems. J. Am. Chem. Soc. 1990, 112, 7508–7514. 10.1021/ja00177a009.

[ref33] DAISY program simulator as implemented in TOPSPIN, version 4.0.6, for Bruker.

[ref34] Coupling constants were calculated from a generalization of the Karplus equation established by Haasnoot as implemented in Maestro version 9.0, Schrödinger, LLC, New York, NY, 2009. Haasnoot C. A. G.; de Leeuw F. A. A. M.; Altona C. The Relationship between Proton-Proton NMR Coupling Constants and Substituent Electronegativities-I: An Empirical Generalization of the Karplus Equation. Tetrahedron 1980, 36, 2783–2792. 10.1016/0040-4020(80)80155-4.

[ref35] Crich D.; Huang X.; Newcomb M. Inter- and Intramolecular Pathways for the Formation of Tetrahydrofurans from β-(Phosphatoxy)Alkyl Radicals. Evidence for a Dissociative Mechanism. J. Org. Chem. 2000, 65, 523–529. 10.1021/jo991570o. 10813967

[ref36] Cortezano-Arellano O.; Quintero L.; Sartillo-Piscil F. Total Synthesis of Cephalosporolide E via a Tandem Radical/Polar Crossover Reaction. The Use of the Radical Cations under Nonoxidative Conditions in Total Synthesis. J. Org. Chem. 2015, 80, 2601–2608. 10.1021/jo502757c. 25642728

[ref37] Martín A.; Pérez-Martín I.; Quintanal L. M.; Suárez E. Intramolecular 1,8-Hydrogen Atom Transfer. Stereoselectivity of the Hexopyranos-5′-yl Radical Reactions in Hexp-(1→4)-Hexp Disaccharide Systems. J. Org. Chem 2008, 73, 7710–7720. 10.1021/jo801499d. 18778104

[ref38] Crich D.; Suk D.-H. The β-(Acyloxy)alkyl Radical Rearrangement Revisited. Can. J. Chem. 2004, 82, 75–79. 10.1139/v03-148.

[ref39] aKoch A.; Giese B. Radical Rearrangements of 2-*O*-(Diphenoxyphosphoryl)Glycosyl Bromides. Helv. Chim. Acta 1993, 76, 1687–1701. 10.1002/hlca.19930760426.

[ref40] For comparison between the displacement of acetoxy and diphenoxyphosphatoxy groups in the S-T rearrangement, see Ref (21d).

[ref41] aChatgilialoglu C.; Lalevée J. Recent Applications of the (TMS)_3_SiH Radical-Based Reagent. Molecules 2012, 17, 527–555. 10.3390/molecules17010527. 22269866 PMC6268903

[ref42] aDeslongchamps P.; Rowan D. D.; Pothier N.; Sauvé G.; Saunders J. K. 1,7-Dioxaspiro[5.5]Undecanes. An Excellent System for the Study of Stereoelectronic Effects (Anomeric and Exo-Anomeric Effects) in Acetals. Can. J. Chem. 1981, 59, 1105–1121. 10.1139/v81-164.

[ref43] aBeckwith A. L. J.; Easton C. J. Stereoelectronic Effects in Hydrogen Atom Abstraction from Substituted 1,3-Dioxanes. J. Am. Chem. Soc. 1981, 103, 615–619. 10.1021/ja00393a019.

[ref44] Chatgilialoglu C.; Studer A. Encyclopedia of Radicals in Chemistry, Biology and Materials; John Wiley & Sons, Ltd: Chichester, UK, 2012; pp. 655–692, 10.1002/9781119953678.rad019.

[ref45] Although, examples of pure radical β-(ester)alkyl fragmentation with concomitant decarboxylation are scarce, we have used it as a simple mechanism to explain the partial loss of deuterium found in some of the 1,5-HAT–Surzur-Tanner rearrangement products. Alternative mechanisms like adventitious acid-catalyzed opening and recombination of the spiroketal ring or the use of *n*-Bu_3_SnH(D) as a potential external nucleophile for the trapping of the alkene radical-cation intermediate can also be considered. For references of pure radical β-(ester)alkyl fragmentation, see:aMotherwell W. B.; Imboden C. Decarboxylation via *O*-Acyl Thiohydroxamates. In Radicals in Organic Synthesis; Renaud P.; Sibi M. P., Eds.; Wiley-VCH Verlag GmbH: Weinheim, Germany, 2001; pp. 109–134, 10.1002/9783527618293.ch7.

[ref46] Bruyère I.; Tóth Z.; Benyahia H.; Xue J. L.; Praly J.-P. NaBH_3_CN and other Systems as Substitutes of Tin and Silicon Hydrides in the Light or Heat-initiated Reduction of Halosugars: a Tunable Access to either 2-Deoxy Sugars or 1,5-Anhydro-itols. Tetrahedron 2013, 69, 9656–9662. 10.1016/j.tet.2013.09.032.

[ref47] For a pioneer report, see: Zhang J.; Li Y.; Zhang F.; Hu C.; Chen Y. Generation of Alkoxyl Radicals by Photoredox Catalysis Enables Selective C(Sp 3)–H Functionalization under Mild Reaction Conditions. Angew. Chem., Int. Ed. 2016, 55, 1872–1875. 10.1002/anie.201510014. 26680274

[ref48] Abraham R. J.; Gottschalck H.; Paulsen H.; Thomas W. A. The Proton Magnetic Resonance Spectra and Conformations of Cyclic Compounds. Part II. The p.m.r. Spectra of the Conduritols. J. Chem. Soc. 1965, 6268–6277. 10.1039/jr9650006268.

[ref49] For the synthesis of an analogous *trans-*fused bis(pyran) compound from a sugar, see: Oguri H. Designed Hapten Aimed at Anti-Ciguatoxin Monoclonal Antibody: Synthesis, Immunization and Discrimination of the C2 Configuration. Synthesis 1999, 1999, 1431–1436. 10.1055/s-1999-3646.

[ref50] aGuyenne S.; León E. I.; Martín A.; Pérez-Martín I.; Suárez E. Intramolecular 1,8-Hydrogen Atom Transfer Reactions in Disaccharide Systems Containing Furanose Units. J. Org. Chem. 2012, 77, 7371–7391. 10.1021/jo301153u. 22853298

[ref51] aLee I.-C.; Zulueta M. M. L.; Shie C.-R.; Arco S. D.; Hung S.-C. Deuterium-Isotope Study on the Reductive Ring Opening of Benzylidene Acetals. Org. Biomol. Chem. 2011, 9, 7655–7658. 10.1039/c1ob06056b. 21922112

[ref52] Barnett D. W.; Panigot M. J.; Curley R. W. Stereoselective Route to 15N-Labeled-β-Deuterated Amino Acids: Synthesis of (2*S*,3*R*)-[3-^2^H,15N]-Phenylalanine. Tetrahedron: Asymmetry 2002, 13, 1893–1900. 10.1016/S0957-4166(02)00487-1.

[ref53] aSánchez-Eleuterio A.; Quintero L.; Sartillo-Piscil F. High 1,3-*trans* Stereoselectivity in Nucleophilic Substitution at the Anomeric Position and β-Fragmentation of the Primary Alkoxyl Radical in 3-Amino-3-deoxy-ribofuranose Derivatives: Application to the Synthesis of 2-*epi*-(−)-Jaspine B. J. Org. Chem. 2011, 76, 5466–5471. 10.1021/jo200639t. 21574579

[ref54] Cipolla L.; Lay L.; Nicotra F. New and Easy Access to *C*-Glycosides of Glucosamine and Mannosamine. J. Org. Chem. 1997, 62, 6678–6681. 10.1021/jo970127f.

[ref55] aMitsunobu O. The Use of Diethyl Azodicarboxylate and Triphenylphosphine in Synthesis and Transformation of Natural Products. Synthesis 1981, 1981, 1–28. 10.1055/s-1981-29317.

[ref56] aBennek J. A.; Gray G. R. An Efficient Synthesis of Anhydroalditols and Allylic-Glycosides. J. Org. Chem. 1987, 52, 892–897. 10.1021/jo00381a030.

[ref57] Li X.; Li J.; Mootoo D. R. Synthesis of the ABCD Trioxadispiroketal Subunit of Azaspiracid-1: An Iodoetherification–Dehydroiodination Strategy for Complex Spiroketals. Org. Lett. 2007, 9, 4303–4306. 10.1021/ol701866v. 17880232

[ref58] aJarikote D. V.; O’Reilly C.; Murphy P. V. Ultrasound-Assisted Synthesis of *C*-Glycosides. Tetrahedron Lett. 2010, 51, 6776–6778. 10.1016/j.tetlet.2010.10.113.

[ref59] aGrice P.; Ley S. V.; Pietruszka J.; Priepke H. W. M.; Warriner S. L. Preparation, Structure, Derivatisation and NMR Data of Cyclohexane-1,2-Diacetal Protected Carbohydrates. J. Chem. Soc., Perkin Trans. 1 1997, 351–364. 10.1039/a605851e.

[ref60] aPirrung M. C. The Synthetic Organic Chemist’s Companion; John Wiley & Sons, Inc.: Hoboken, NJ, USA, 2007, pp. 171, 10.1002/9780470141045.

[ref61] La Ferla B.; Russo L.; Airoldi C.; Nicotra F. Solid-Phase Supported Mimic of GDP-L-Galactose. Tetrahedron: Asymmetry 2009, 20, 744–745. 10.1016/j.tetasy.2009.03.005.

[ref62] Allavudeen S. S.; Kuberan B.; Loganathan D. A Method for Obtaining Equilibrium Tautomeric Mixtures of Reducing Sugars via Glycosylamines Using Nonaqueous Media. Carbohydr. Res. 2002, 337, 965–968. 10.1016/S0008-6215(02)00075-7. 12007481

[ref63] Beignet J.; Tiernan J.; Woo C. H.; Kariuki B. M.; Cox L. R. Stereoselective Synthesis of Allyl-*C*-Mannosyl Compounds: Use of a Temporary Silicon Connection in Intramolecular Allylation Strategies with Allylsilanes. J. Org. Chem. 2004, 69, 6341–6356. 10.1021/jo049061w. 15357594

[ref64] Martín A.; Pérez-Martín I.; Quintanal L. M.; Suárez E. Intramolecular 1,8- versus 1,6-Hydrogen Atom Transfer between Pyranose Units in a (1→4)-Disaccharide Model Promoted by Alkoxyl Radicals. Conformational and Stereochemical Requirements. Org. Lett. 2007, 9, 1785–1788. 10.1021/ol070496q. 17407301

[ref65] aKobertz W. R.; Bertozzi C. R.; Bednarski M. D. An Efficient Method for the Synthesis of α- and β-*C*-Glycosyl Aldehydes. Tetrahedron Lett. 1992, 33, 737–740. 10.1016/S0040-4039(00)77703-3.

