# Supplementary material for: 1,5-Hydrogen Atom Transfer/Surzur–Tanner Rearrangement: A Radical Cascade Approach for the Synthesis of 1,6-Dioxaspiro[4.5]decane and 6,8-Dioxabicyclo[3.2.1]octane Scaffolds in Carbohydrate Systems
Source: J Org Chem. 2021 Sep 23;86(21):14508–52. doi: 10.1021/acs.joc.1c01376 (PMC8576821; doi:10.1021/acs.joc.1c01376)
Supplement: Supplementary file 1 — jo1c01376_si_001.pdf [file jo1c01376_si_001.pdf]

Supporting Information

1,5-Hydrogen Atom Transfer–Surzur-Tanner-rearrangement: A Radical Cascade Approach for the Synthesis of 1,6-Dioxaspiro[4.5]decane and 6,8-Dioxabicyclo[3.2.1]octane Scaffolds in Carbohydrate Systems

Elisa I. León,[a] Ángeles Martín,[a] Adrián S. Montes,[a,b] Inés Pérez-Martín,*,[a] María del Sol Rodríguez,[a] Ernesto Suárez*,[a]

[a] Síntesis de Productos Naturales, Instituto de Productos Naturales y Agrobiología del CSIC, Avda. Astrofísico Francisco Sánchez 3, 38206, La Laguna, Tenerife, Spain
[b] Doctoral and Postgraduate School, Universidad de La Laguna, Avda. Astrofísico Francisco Sánchez s/n, 38200 La Laguna, Tenerife, Spain

Table of Contents:

1. Table S1. Comparison between Experimental and Calculated Ring Coupling Constant of 3-*C*-(Glycopyranosyl)1-propoxyphthalimides. S3
2. Table S2. Comparison between Experimental and Calculated Ring Coupling Constant of *C*-(Glycopyranosyl)*N*-methoxyphthalimides. S4
3. Table S3. Comparison between Experimental and Calculated Ring Coupling Constant of 10-Deoxy-1,6-dioxaspiro[4.5]decane Compounds. S5
4. Table S4. Comparison between Experimental and Calculated Long-range $^{4}J_{w}$ Coupling Constants.. S5
5. Table S5. Selected signals of ^{1}H NMR spectra of labelled [PhCH-^{2}H]**31** and [PhCH-^{2}H]**97**. S6
6. Table S6. Selected signals of ^{13}C{H} NMR spectra of labelled [PhCH-^{2}H]**31** and [PhCH-^{2}H]**97**. S6
7. Figure S1. ^{1}H NMR spectrum of [PhCH-^{2}H]**31**. S7
8. Figure S2. ^{1}H NMR spectrum of [PhCH-^{2}H]**97**. S7
9. Figure S3. ^{13}C{H} NMR spectrum of [PhCH-^{2}H]**31.** S8
10. Figure S4. ^{13}C{H} NMR spectrum of [PhCH-^{2}H]**97**. S8
11. Table S7. Comparison between Experimental and Calculated Ring Coupling Constant of 4-Deoxy-6,8-dioxabicyclo[3.2.1]octane Compounds. S9
12. Table S8. Comparison between Experimental and Calculated Ring Coupling Constant of 3-*C*-(α-D-ribofuranosyl)1-propoxyphthalimides. S9
13. Table S9. Reactivity Differences between LGs in the 1,5-Hydrogen Atom Transfer–Surzur-Tanner Rearrangement Sequence for the Synthesis of 1,6-Dioxaspiro[4.5]decane and 6,8-Dioxabicyclo[3.2.1]octane Scaffolds. S10
14. Figures S5–S94. ^{1}H and ^{13}C{1H} NMR spectra of all new compounds.......….S11–S100

Table S1. Comparison between Experimental and Calculated Ring Coupling Constant of 3-*C*-(Glycopyranosyl)1-propoxyphthalimides.[a]

Compound	Conformation[b]	$^3J_{1,2}$ [Hz]	$^3J_{2,3}$ [Hz]	$^3J_{3,4}$ [Hz]	$^3J_{4,5}$ [Hz]
**1**	$^4C_1$ ($^3J_{calc.}$)	6.4	9.2	9.5	9.2
	$^1C_4$ ($^3J_{calc.}$)	0.9	2.5	2.8	1.2
	$^3J_{exp.}$[c]	5.5	9.0	7.8	n.o.
**2**	$^4C_1$ ($^3J_{calc.}$)	5.8	9.0	8.7	9.2
	$^1C_4$ ($^3J_{calc.}$)	1.2	2.6	3.6	1.6
	$^3J_{exp.}$	5.5	9.4	5.7	n.o.
**3**	$^4C_1$ ($^3J_{calc.}$)	8.8	9.3	9.0	9.2
	$^1C_4$ ($^3J_{calc.}$)	0.8	2.4	3.4	1.4
	$^3J_{exp.}$	9.5	9.5	n.o.	8.0
**4**	$^4C_1$ ($^3J_{calc.}$)	8.7	8.9	9.1	9.2
	$^1C_4$ ($^3J_{calc.}$)	0.9	2.9	3.8	1.5
	$^3J_{exp.}$	9.5	8.9	9.3	9.5
**5**	$^4C_1$ ($^3J_{calc.}$)	0.8	2.9	9.6	9.2
	$^1C_4$ ($^3J_{calc.}$)	8.8	2.5	3.3	1.4
	$^3J_{exp.}$	2.6	3.4	8.7	8.4
**6**	$^4C_1$ ($^3J_{calc.}$)	1.0	2.3	9.1	9.2
	$^1C_4$ ($^3J_{calc.}$)	8.7	2.6	3.3	1.5
	$^3J_{exp.}$	2.9	2.9	n.o.	n.o.
**7**	$^4C_1$ ($^3J_{calc}$)	0.4	2.6	9.5	9.2
	$^1C_4$ ($^3J_{calc}$)	6.1	3.0	3.4	1.3
	$^3J_{exp}$	1.0	3.3	9.3	9.8
**8**	$^4C_1$ ($^3J_{calc.}$)	0.1	2.2	9.1	9.1
	$^1C_4$ ($^3J_{calc.}$)	6.2	2.7	3.8	1.5
	$^3J_{exp.}$	< 1	1.9	n.o.	n.o.
**9**	$^4C_1$ ($^3J_{calc.}$)	0.3	3.0	2.6	5.3
	$^1C_4$ ($^3J_{calc.}$)	5.4	9.0	2.7	0.5
	$^3J_{exp.}$	3.0	5.9	3.2	4.5
**10**	$^4C_1$ ($^3J_{calc.}$)	0.5	2.4	2.6	5.2
	$^1C_4$ ($^3J_{calc.}$)	5.0	9.4	2.8	0.4
	$^3J_{exp.}$	3.3	6.3	3.1	4.1
**11**	$^4C_1$ ($^3J_{calc.}$)	1.2	1.7	4.1	10.0, 7.5
	$^1C_4$ ($^3J_{calc.}$)	4.9	7.7	4.4	1.6, 1.6
	$^3J_{exp.}$	2.0	3.3	5.2	7.9, 6.0
**12**	$^4C_1$ ($^3J_{calc.}$)	0.8	2.8	2.5	10.4, 6.3
	$^1C_4$ ($^3J_{calc.}$)	5.1	8.5	2.9	0.6, 2.2
	$^3J_{exp.}$	1.3	4.1	n.o.	n.o.

[a]Vicinal ring coupling constants ($^3J_{HCCH}$) were calculated from a generalization of the Karplus equation established by Haasnoot et al.[1] as implemented in Maestro version 9.0, Schrödinger, LLC, New York, NY, 2009.
[b]Minimized structure performed with AMBER* force field as implemented in MacroModel, version 9.9.013 with the GB/SA solvent model for $CHCl_3$, Schrödinger, LLC, New York, NY, 2009. The structures of compounds containing phosphorous were minimized with Chem3D 19.0.
[c]Experimental $^3J_{HH}$ extracted from simulated 1D ^{1}HNMR spectrum using the DAISY program as implemented in TOPSPIN, version 4.0.6, for Bruker.

Table S2. Comparison between Experimental and Calculated Ring Coupling Constant of *C*-(Glycopyranosyl)*N*-methoxyphthalimides.[a]

Compound	Conformation[b]	$^3J_{1,2}$ [Hz]	$^3J_{2,3}$ [Hz]	$^3J_{3,4}$ [Hz]	$^3J_{4,5}$ [Hz]	*d* O1'–H5 [Å][c]
**16**	$^4C_1$ ($^3J_{calc.}$)	5.8	9.1	9.4	9.2	2.425
	$^1C_4$ ($^3J_{calc.}$)	0.3	2.6	2.4	0.9	5.653
	$^3J_{exp.}$[d]	4.6	7.1	6.5	6.8	
**17**	$^4C_1$ ($^3J_{calc.}$)	6.6	8.2	8.6	9.1	2.411
	$^1C_4$ ($^3J_{calc.}$)	0.3	2.7	2.0	1.0	5.570
	$^3J_{exp.}$	5.3	7.4	7.0	7.0	
**18**	$^4C_1$ ($^3J_{calc.}$)	6.6	8.2	8.7	9.2	2.410
	$^1C_4$ ($^3J_{calc.}$)	0.2	2.8	2.5	1.0	5.565
	$^3J_{exp.}$	4.3	6.4	5.8	6.2	
**19**	$^4C_1$ ($^3J_{calc.}$)	6.5	8.2	8.5	9.1	2.280
	$^1C_4$ ($^3J_{calc.}$)	0.5	2.8	2.1	1.0	5.589
	$^3J_{exp.}$	4.7	7.3	7.3	7.9	
**20**	$^4C_1$ ($^3J_{calc.}$)	6.0	8.9	3.1	0.5	2.412
	$^1C_4$ ($^3J_{calc.}$)	0.1	3.2	2.9	5.8	5.644
	$^3J_{exp.}$	5.4	9.1	3.2	2.1	
**21**	$^4C_1$ ($^3J_{calc.}$)	6.1	9.0	2.1	-0.1	2.906
	$^1C_4$ ($^3J_{calc.}$)	-0.1	2.8	3.3	6.2	5.583
	$^3J_{exp.}$	5.6	8.2	2.9	2.7	
**22**	$^4C_1$ ($^3J_{calc.}$)	9.2	2.8	3.3	1.5	4.540
	$^1C_4$ ($^3J_{calc.}$)	2.0	3.7	5.4	6.2	3.013
	$^3J_{exp.}$	7.6	3.2	5.2	3.7	
**23**	$^4C_1$ ($^3J_{calc.}$)	0.2	3.1	3.1	5.7	4.674
	$^1C_4$ ($^3J_{calc.}$)	5.7	9.3	2.7	0.7	3.862
	$^3J_{exp.}$	5.9	9.3	3.4	2.0	
**24**	$^4C_1$ ($^3J_{calc}$)	0.3	2.6	2.7	5.3	4.541
	$^1C_4$ ($^3J_{calc}$)	5.6	9.3	2.7	0.1	3.031
	$^3J_{exp.}$	5.0	7.9	3.3	2.5	

[a]Vicinal ring coupling constants ($^3J_{HCCH}$) were calculated from a generalization of the Karplus equation established by Haasnoot et al.[1] as implemented in Maestro version 9.0, Schrödinger, LLC, New York, NY, 2009.
[b]Minimized structure performed with AMBER* force field as implemented in MacroModel, version 9.9.013 with the GB/SA solvent model for $CHCl_3$, Schrödinger, LLC, New York, NY, 2009. The structures of compounds containing phosphorous were minimized with Chem3D 19.0.
[c]The minimum distance O1'–H5 for each conformational isomer, was calculated performing a coordinate scan calculation of the O1'–C1'–C1–O1 dihedral from –180 to 180° in increments of 5°.
[d]Experimental $^3J_{HH}$ extracted from simulated 1D ^{1}HNMR spectrum using the DAISY program as implemented in TOPSPIN, version 4.0.6, for Bruker.

Table S3. Comparison between Experimental and Calculated Ring Coupling Constant of 10-Deoxy-1,6-dioxaspiro[4.5]decane Compounds.[a]

Compound	Conformation[b]	$^3J_{2a,3}$ [Hz]	$^3J_{2e,3}$ [Hz]	$^3J_{3,4}$ [Hz]	$^3J_{4,5}$ [Hz]	$^3J_{4,5a}$ [Hz]	$^3J_{4,5e}$ [Hz]
**25**	$^4C_1$ ($^3J_{calc.}$)	11.1	4.6	8.7	9.2	--	--
	$^1C_4$ ($^3J_{calc.}$)	2.7	3.0	3.2	1.5	--	--
	$^3J_{exp.}$[c]	11.5	5.1	8.9	9.9	--	--
**31**	$^4C_1$ ($^3J_{calc.}$)	2.4	3.3	2.8	5.2	--	--
	$^1C_4$ ($^3J_{calc.}$)	11.1	4.6	2.2	0.4	--	--
	$^3J_{exp.}$	12.1	4.5	2.7	1.7	--	--
**37**	$^4C_1$ ($^3J_{calc.}$)	3.2	2.5	4.1	--	10.2	7.1
	$^1C_4$ ($^3J_{calc.}$)	10.0	6.5	3.8	--	1.2	1.8
	$^3J_{exp.}$	8.7	6.3	5.5	--	2.4	1.0
**38**	$^4C_1$ ($^3J_{calc.}$)	2.5	3.0	3.1	--	10.3	6.7
	$^1C_4$ ($^3J_{calc.}$)	10.8	5.3	2.7	--	0.7	2.1
	$^3J_{exp.}$	11.7	5.3	3.5	--	2.3	1.6

[a]Vicinal ring coupling constants ($^3J_{HCCH}$) were calculated from a generalization of the Karplus equation established by Haasnoot et al.[1] as implemented in Maestro version 9.0, Schrödinger, LLC, New York, NY, 2009.
[b]Minimized structure performed with AMBER* force field as implemented in MacroModel, version 9.9.013 with the GB/SA solvent model for $CHCl_3$, Schrödinger, LLC, New York, NY, 2009. The structures of compounds containing phosphorous were minimized with Chem3D 19.0.
[c]Experimental $^3J_{HH}$ extracted from simulated 1D ^{1}HNMR spectrum using the DAISY program as implemented in TOPSPIN, version 4.0.6, for Bruker.

Table S4. Comparison between Experimental and Calculated Long-range ^4J_w Coupling Constants.

Compound	Conformation	Φ_1 [°][a]	Φ_2 [°][a]	^4J_W	$^4J_{calc.}$ [Hz][b]	$^4J_{exp.}$ [Hz][c]
**31**	$^1C_4$	176.2	–176.8	$^4J_{2a,4}$	1.3	1.3
[PhCH-^{2}H]**31**	$^1C_4$	173.8	–173.5	$^4J_{2a,4}$	1.3	1.0
**45**	$^4C_1$	–168.6	–160.2	$^4J_{2,1'a}$	1.1	1.1
**46**	$^1H_2$	164.1	161.9	$^4J_{2,4}$	1.1	1.5
**47**	$^1C_4$	170.4	–164.7	$^4J_{2,4}$	1.2	1.2
(5-^{2}H)**47**	$^1C_4$	170.4	–164.7	$^4J_{2,4}$	1.2	1.2
**53**	$^4C_1$	–171.8	–158.5	$^4J_{2,1'a}$	1.1	1.1

[a]Dihedral angles calculated over minimized structures using AMBER* force field as implemented in MacroModel, version 9.9.013 with the GB/SA solvent model for $CHCl_3$, Schrödinger, LLC, New York, NY, 2009.
[b]Long-range $^4J_{HH}$ were calculated from three-parameters equation ($^4J_{HH} = \cos^2 \Phi_1 + \cos^2 \Phi_2 - 0{,}7$) as established by Abraham et al.[2]
[c]All experimental $^4J_{HH}$ were extracted from simulated 1D ^{1}HNMR spectra using the DAISY program as implemented in TOPSPIN, version 4.0.6, for Bruker.

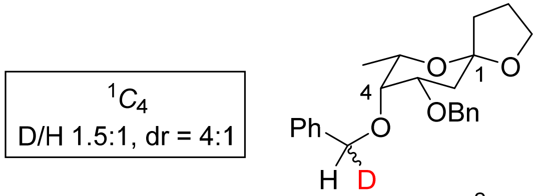

[PhCH-^{2}H]**31**

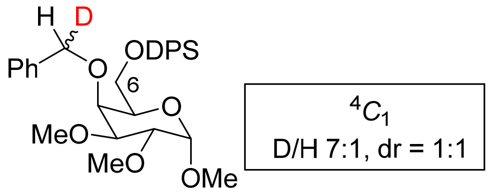

[PhCH-^{2}H]**97**

Table S5. Selected signals of ^{1}H NMR spectra of labelled [PhCH-^{2}H]**31** and [PhCH-^{2}H]**97**.

Compound	6-H [ppm] (rel. int.)		2-H_{ax} [ppm] (rel. int.)		3-H_{ax} [ppm] (rel. int.)		4-PhC*H*D-O [ppm] (rel. int.)	
	$CDCl_3$	C_6D_6	$CDCl_3$	C_6D_6	$CDCl_3$	C_6D_6	$CDCl_3$	C_6D_6
[PhCH-^{2}H]**31**	1.130 (d, 1.5H)[a]<br>1.133 (d, 0.3H)<br>1.135 (d, 1.2H)	1.276 (d, 1.5H)[a]<br>1.280 (d, 1.5H)	2.32 (dd, 1H)	2.494 (dd, 0.5H)<br>2.496 (dd, 0.5H)	3.929 (ddd, 0.5H)<br>3.931 (ddd, 0.5H)	4.02 (ddd, 1H)	4.71(br s, 0.1H)<br>4.72 (d, 0.4H)<br>4.93 (br s, 0.5H)<br>4.95 (d, 0.4H)	4.57 (br s, 0.1H)<br>4.59 (d, 0.4H)<br>5.02 (br s, 0.5H)<br>5.05 (d, 0.4H)
[PhCH-^{2}H]**97**	n. o.[b, c]	4.05 (dd, 0.5)[b]<br>4.06 (dd, 0.5)<br>4.107 (dd, 0.5)<br>4.105 (dd, 0.5)	n. o.[c]	n. o.[c]	3.56 (dd, 1H)	3.73 (dd, 1H)	4.589 (br s, 0.4H)<br>4.60 (d, 0.1H)<br>4.90 (br s, 0.4H)<br>4.91 (d, 0.1H)	4.58 (br s, 0.4H)<br>4.61 (d, 0.1H)<br>5.05 (br s, 0.4H)<br>5.07 (d, 0.1H)

[a]6-Me. [b]6-CH_2.[c]Not observed, overlapped with other signals.

Table S6. Selected signals of ^{13}C{H} NMR spectra of labelled [PhCH-^{2}H]**31** and [PhCH-^{2}H]**97**.

Compound	C4[ppm] (rel. int.)		4-PhCHD-O [ppm] (rel. int.)	
	$CDCl_3$	C_6D_6	$CDCl_3$	C_6D_6
[PhCH-^{2}H]**31**	74.97 (0.5)<br>75.02 (0.1)<br>75.09 (0.4)	76.85 (0.5)<br>76.88 (0.1)<br>76.93 (0.4)	73.59 (t, J_{CD} = 22.1 Hz) (0.6)<br>74.18 (s) (0.4)	74.85 (t, J_{CD} = 22.1 Hz) (0.6)<br>75.26 (s) (0.4)
[PhCH-^{2}H]**97**	73.56 (0.44)<br>73.59 (0.44)<br>73.64 (0.12)	75.48 (0.44)<br>75.51 (0.44)<br>75.55 (0.12)	74.35 (t, J_{CD} = 22.1 Hz) (0.88)<br>74.73 (s) (0.12)	75.05 (t, J_{CD} = 21.1 Hz) (0.44)<br>75.08 (t, J_{CD} = 22.1 Hz) (0.44)<br>75.45 (s) (0.12)

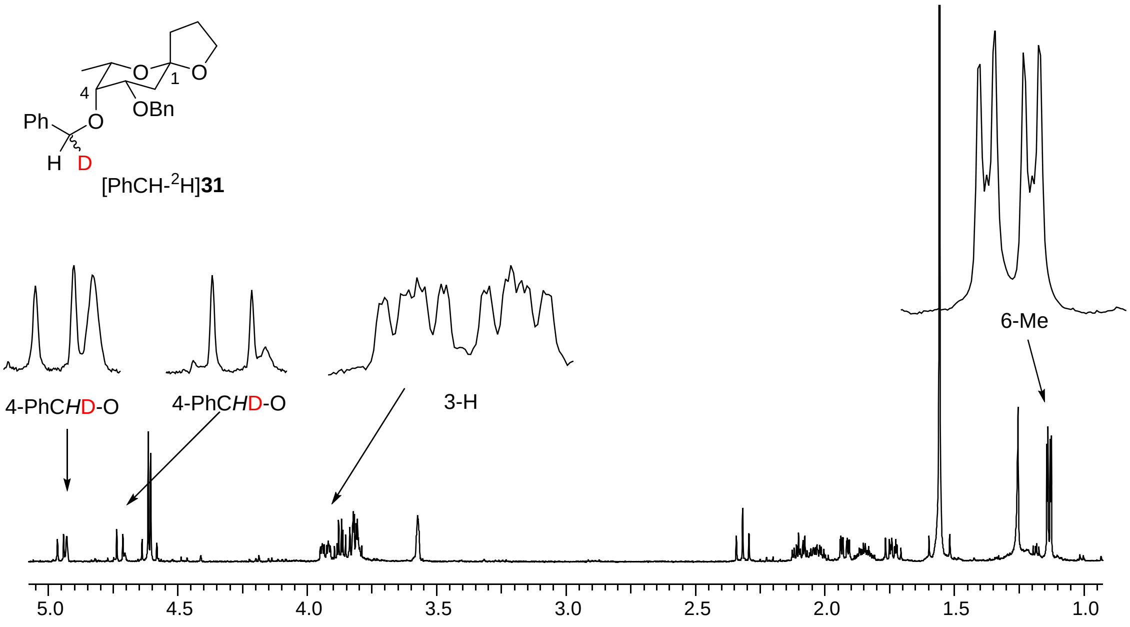

Figure S1. ^{1}H NMR (500 MHz, $CDCl_3$) of [PhCH-^{2}H]**31** (D/H 1.5:1, dr = 4:1) as a 1*R*/1*S* mixture (85:15), only major isomer shown.

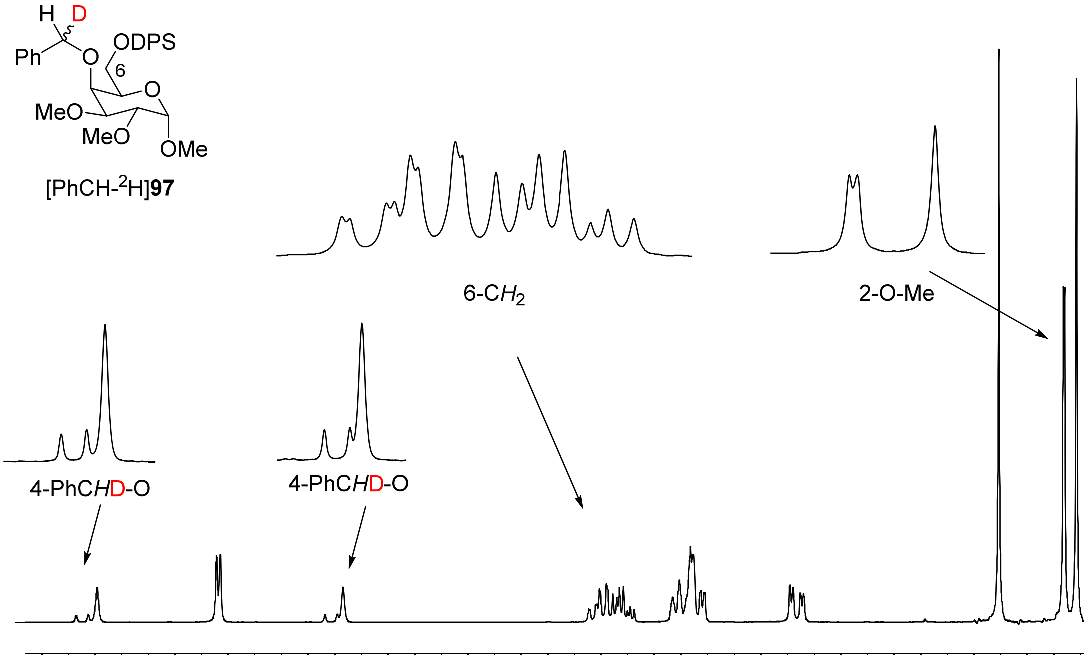

Figure S2. ^{1}H NMR (500 MHz, C_6D_6) of [PhCH-^{2}H]**97** (D/H 7:1, dr = 1:1).

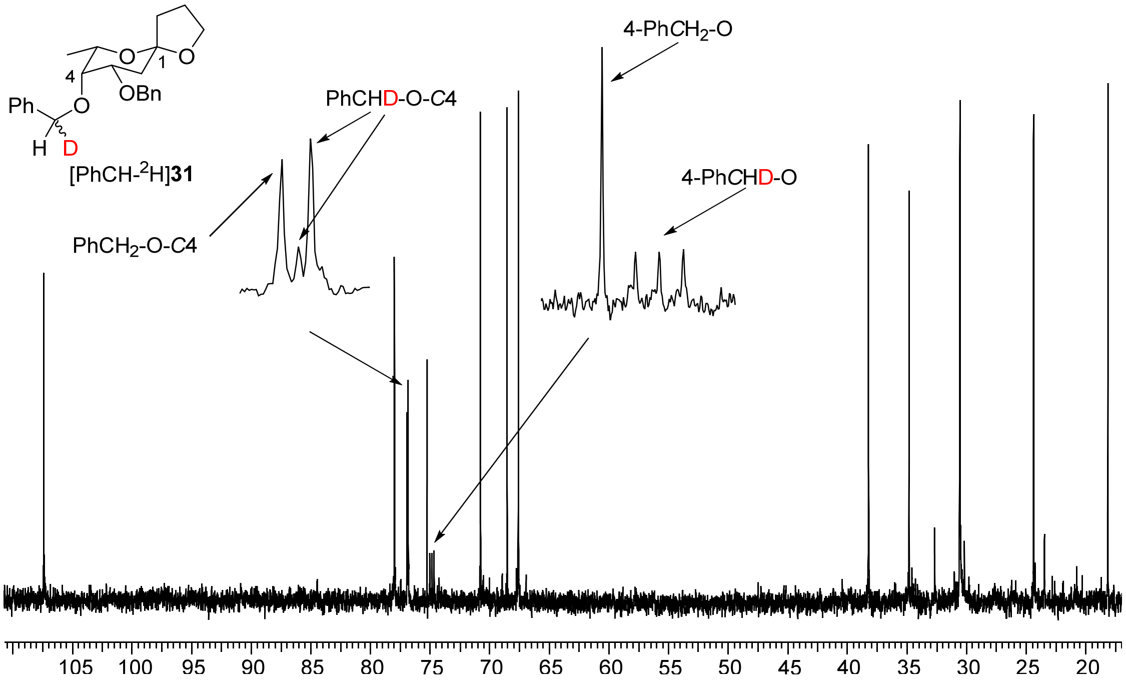

Figure S3. ^{13}C{H} NMR (125.7 MHz, C_6D_6) of [PhCH-^{2}H]**31** (D/H 1.5:1, dr = 4:1) as a 1*R*/1*S* mixture (85:15), only major isomer shown.

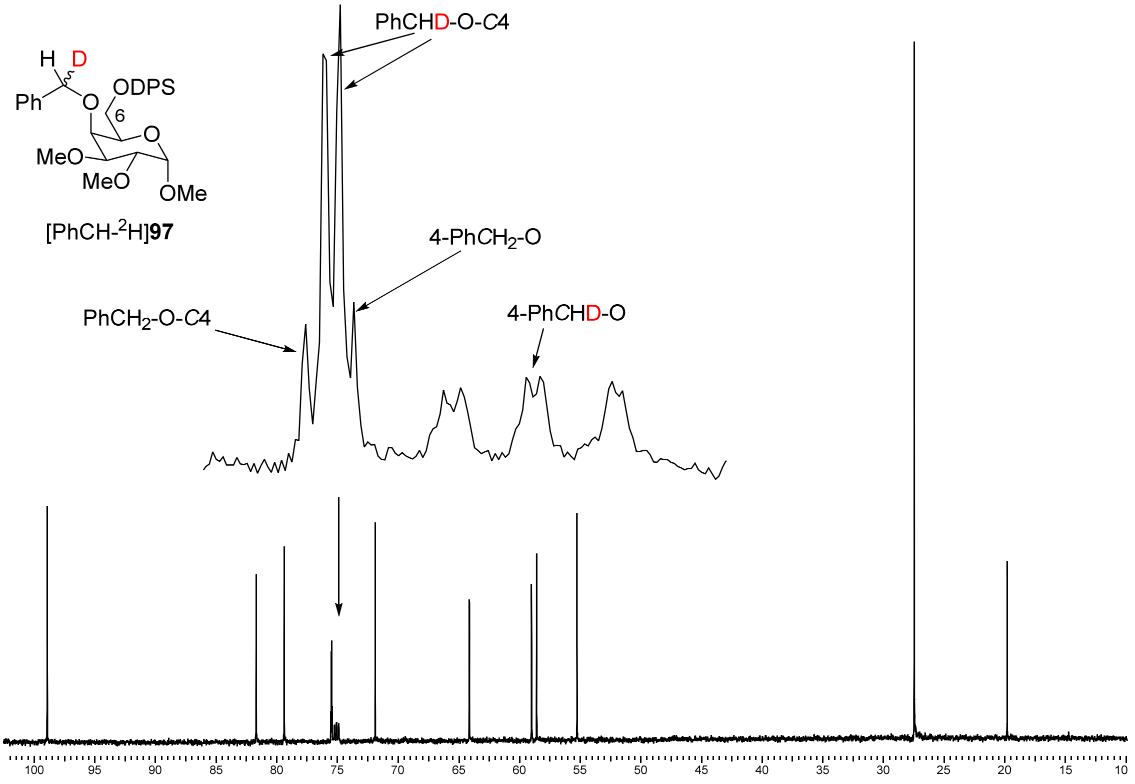

Figure S4. ^{13}C{H} NMR (125.7 MHz, C_6D_6) of [PhCH-^{2}H]**97** (D/H 7:1, dr = 1:1).

Table S7. Comparison between Experimental and Calculated Ring Coupling Constant of 4-Deoxy-6,8-dioxabicyclo[3.2.1]octane Compounds.[a]

Compound	Conformation	$^3J_{1,2}$ [Hz]	$^3J_{2,3}$ [Hz]	$^3J_{3,4a}$ [Hz]	$^3J_{3,4e}$ [Hz]	$^3J_{1,1'a}$ [Hz]	$^3J_{1,1'b}$ [Hz]
**45**	$^4C_1$ (Calc.)[b]	3.6	7.5	10.2	6.3	6.1	1.1
	Exper.[c]	4.3	8.0	10.1	6.6	5.0	0.0
**53**	$^4C_1$ (Calc.)	4.2	6.7	9.5	6.8	6.4	1.7
	Exper.	3.9	7.8	10.0	6.5	5.1	0.0
[4-^{2}H]**55**	$^1C_4$ (Calc.)	3.4	7.6	10.2	6.2	6.3	1.3
	Exper.	4.7	8.2	n.o.	6-7	4.7	0.0
**61**	$^1C_4$ (Calc.)	2.6	3.2	10.3	6.1	6.3	1.2
	Exper.	2.8	4.1	11.1	6.0	5.8	0.9

[a]Vicinal ring coupling constants ($^3J_{HCCH}$) were calculated from a generalization of the Karplus equation established by Haasnoot et al.[1] as implemented in Maestro version 9.0, Schrödinger, LLC, New York, NY, 2009.
[b]Minimized structure performed with AMBER* force field as implemented in MacroModel, version 9.9.013 with the GB/SA solvent model for $CHCl_3$, Schrödinger, LLC, New York, NY, 2009. The structures of compounds containing phosphorous were minimized with Chem3D 19.0.
[c]Experimental $^3J_{HH}$ extracted from simulated 1D ^{1}HNMR spectrum using the DAISY program as implemented in TOPSPIN, version 4.0.6, for Bruker

Table S8. Comparison between Experimental and Calculated Ring Coupling Constant of 3-*C*-(α-D-ribofuranosyl)1-propoxyphthalimides.

Compound	Conformation[a]	$^3J_{H,H}$	$^3J_{1,2}$ [Hz]	$^3J_{2,3}$ [Hz]	$^3J_{3,4}$ [Hz]
**13**	$^3T_2$ [27%, P_N = 2°, φ_m = 13°]	$^3J_{calc.}$	5.69	6.49	3.86
	$^3T_2$ [73%, P_S = 354°, φ_m = 48°]	$^3J_{calc.}$	2.56	3.99	7.70
		$^3J_{avg.}$	3.40	4.66	6.67
		$^3J_{exp.}$[b]	3.40	4.66	6.67
**14**	$^3T_2$ [55%, P_N = 358°, φ_m = 57°]	$^3J_{calc.}$	2.06	3.05	8.72
	$^2T_3$ [45%, P_S = 179°, φ_m = 3°]	$^3J_{calc.}$	6.49	6.53	2.11
		$^3J_{avg.}$	4.06	4.62	5.74
		$^3J_{exp.}$[b]	4.06	4.62	5.74
**15**	$^3T_2$ [100%, P_S = 9°, φ_m = 52°]	$^3J_{calc.}$	3.05	3.61	8.84
	$^3T_2$ [0%, P_S = 1°, φ_m = 20°]	$^3J_{calc.}$	5.20	6.24	4.70
		$^3J_{avg.}$	3.05	3.61	8.84
		$^3J_{exp.}$[b]	2.88	3.88	9.06

[a]The conformation of the five-membered ring has been established by pseudorotational analysis and designed using the Altona-Sundaralingam phase angle (*P*) and puckering amplitude (φ_m).[3]
[b]Experimental $^3J_{HH}$ extracted from simulated 1D ^{1}HNMR spectrum using the DAISY program as implemented in TOPSPIN, version 4.0.6, for Bruker.

Table S9. Reactivity Differences between LGs in the 1,5-Hydrogen Atom Transfer–Surzur-Tanner-rearrangement Sequence for the Synthesis of 1,6-Dioxaspiro[4.5]decane and 6,8-Dioxabicyclo[3.2.1]octane Scaffolds.[a]

Compound	Sugar Ring	LG	Method	Product	Yield (%)
**1**	α-D-*gluco*	Ac	B	**25**	50
**2**		$PO(OPh)_2$	D	(2-^{2}H)**25**	62
**3**	β-D-*gluco*	Ac	D	(2-^{2}H)**25**	33
**4**		$PO(OPh)_2$	D	(2-^{2}H)**25**	55
**5**	α-D-*manno*	Ac	D	--	--
**6**		$PO(OPh)_2$	D	(2-^{2}H)**25**	52
**7**	β-D-*manno*	Ac	E	[2-^{2}H]**25**	50
**8**		$PO(OPh)_2$	E	[2-^{2}H]**25**	65
**9**	α-L-*fuco*	Ac	D	[PhCH-^{2}H]**31**	53
**10**		$PO(OPh)_2$	A	**31**	52
**11**	α,β-D-*arabino*	$PO(OPh)_2$	A	**37**	60
**12**	β-D-*arabino*	$PO(OPh)_2$	D	[2-^{2}H]**38**	63
**13**	α-D-*ribo*	Ac	E	[2-^{2}H]**39**	40
**14**		Tf	A	**39**	46
**15**		$PO(OPh)_2$	A	**42** + **43**	62
**16**	α-D-*gluco*	Ac	E	[4-^{2}H]**45**	39
**17**		$PO(OPh)_2$	D	[4-^{2}H]**45**	58
**18**		Ts	D	[4-^{2}H]**45**	58
**19**		$PO(OPh)_2$	A	**53**	30
**20**	α-D-*galacto*	Ac	G	**45**	21
**21**		$PO(OPh)_2$	E	[4-^{2}H]**45**	41
**22**	α-L-*rhamno*	$PO(OPh)_2$	E	[4-^{2}H]**61**	66
**23**	α-L-*fuco*	Ac	G	**55**	23
**24**		$PO(OPh)_2$	E	[4-^{2}H]**55**	41

[a]Reagents and Conditions: Method *A*: *n*-Bu_3SnH (1 equiv), AIBN (0.1 equiv), $PhCH_3$ (0.013 M), reflux. *Method B*: *n*-Bu_3SnH (1 equiv/h), AIBN (0.1 equiv), $PhCH_3$ (0.013 M), reflux. *Method D*: *n*-Bu_3SnD (1 equiv), AIBN (0.1 equiv), $PhCH_3$ (0.013 M), reflux. *Method E*: *n*-Bu_3SnD (1 equiv), BF_3•Et_2O (0.2 equiv), AIBN (0.1 equiv), $PhCH_3$ (0.013 M), reflux. *Method G*: Hantzsch ester (0.37 equiv/h), *fac*-Ir(ppy)$_3$ (0.01 equiv), THF (0.007M), rt, blue LED.

References

(1) Haasnoot, C. A. G.; de Leeuw, F. A. A. M.; Altona, C. The Relationship between Proton-Proton NMR Coupling Constants and Substituent Electronegativities-I. An Empirical Generalization of the Karplus Equation. *Tetrahedron* **1980**, *36*, 2783–2792. https://doi.org/10.1016/0040-4020(80)80155-4.

(2) Abraham, R. J.; Gottschalck, H.; Paulsen, H.; Thomas, W. A. The Proton Magnetic Resonance Spectra and Conformations of Cyclic Compounds. Part II. The p.m.r. Spectra of the Conduritols. *J. Chem. Soc.* **1965**, 6268–6277. https://doi.org/10.1039/jr9650006268.

(3) Program Matlab GUI as described in the following: (a) Hendrickx, P. M.; Martins, J. C. A User-Friendly Matlab Program and GUI for the Pseudorotation Analysis of Saturated Five-Membered Ring Systems Based on Scalar Coupling Constants. *Chem. Cent. J.* **2008**, *2*, 20. https://doi.org/10.1186/1752-153X-2-20. For a description of the pseudorotation concept, see: (b) Altona, C.; Sundaralingam, M. Conformational Analysis of the Sugar Ring in Nucleosides and Nucleotides. A New Description Using the Concept of Pseudorotation. *J. Am. Chem. Soc.* **1972**, *94*, 8205–8212. https://doi.org/10.1021/ja00778a043. (c) Houseknecht, J. B.; Altona, C.; Hadad, C. M.; Lowary, T. L. Conformational Analysis of Furanose Rings with PSEUROT: Parametrization for Rings Possessing the Arabino, Lyxo, Ribo, and Xylo Stereochemistry and Application to Arabinofuranosides. *J. Org. Chem.* **2002**, *67*, 4647–4651. https://doi.org/10.1021/jo025635q.

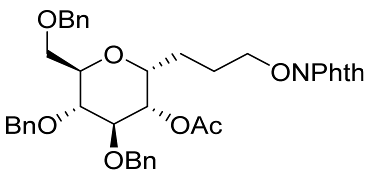

1

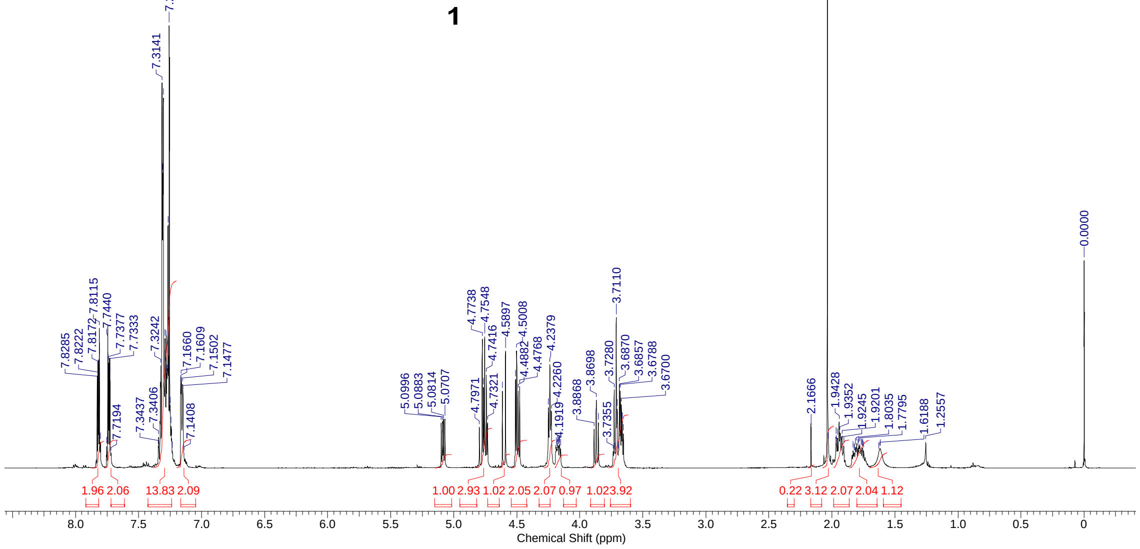

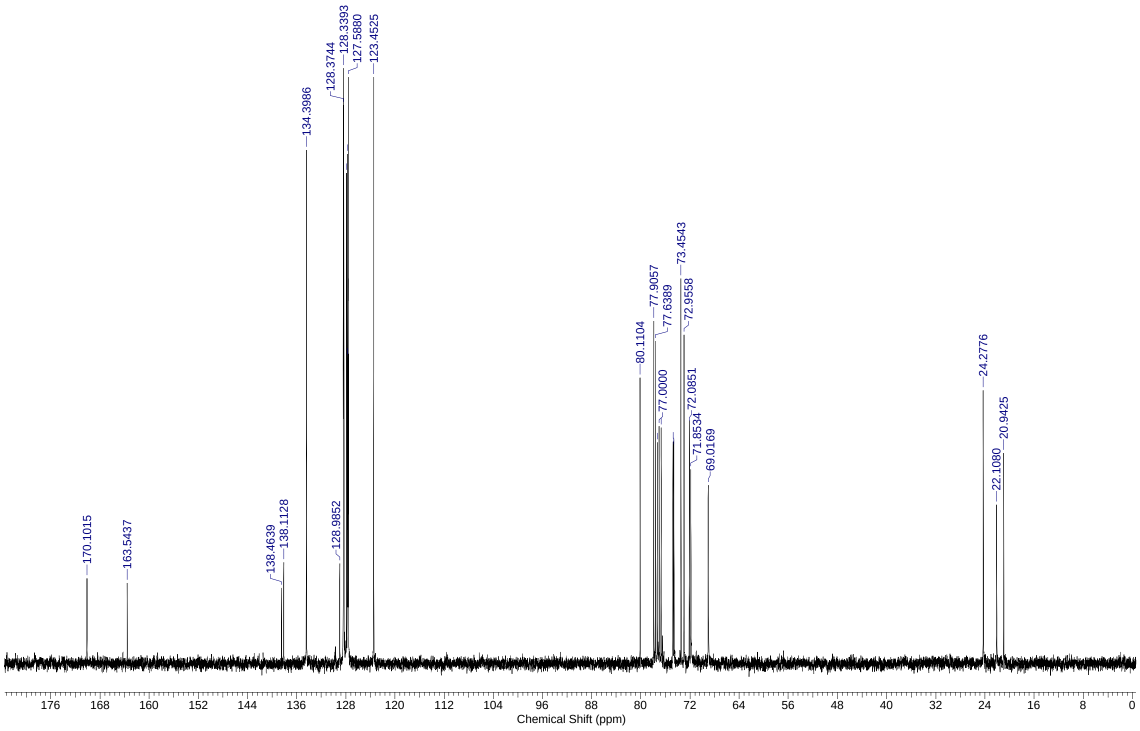

Fig. S5. ^{1}H NMR (500 MHz, $CDCl_3$) and ^{13}C{H} NMR (100.6 MHz, $CDCl_3$) of compound **1**.

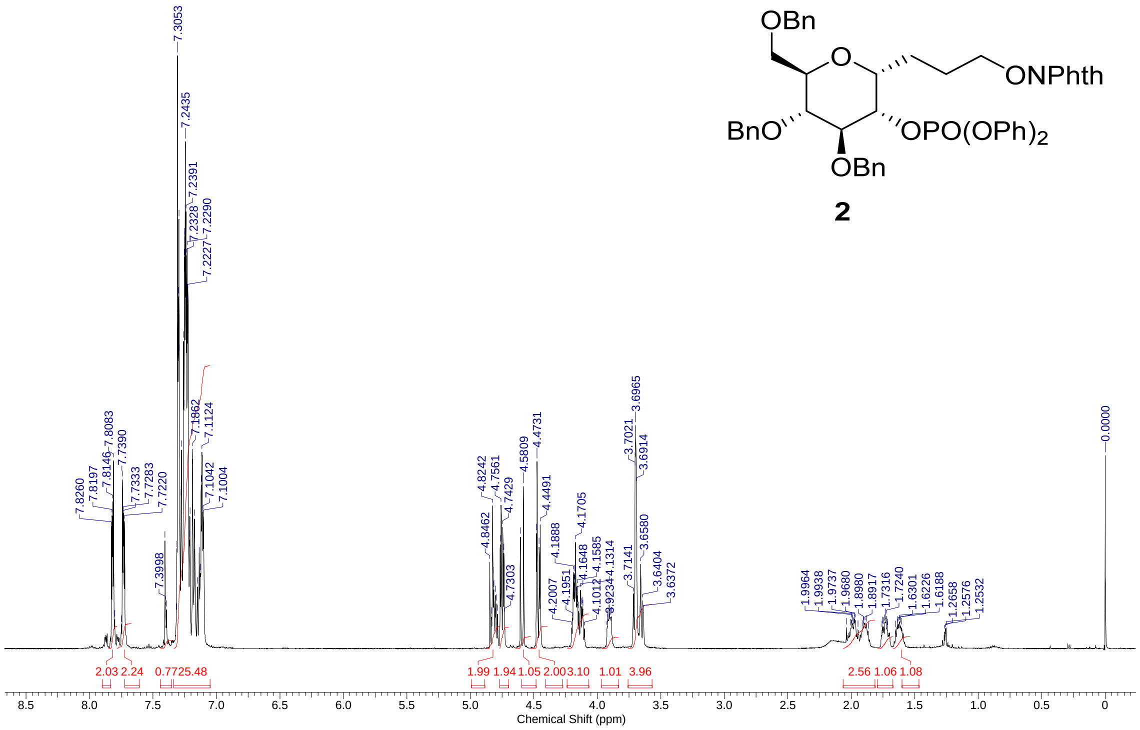

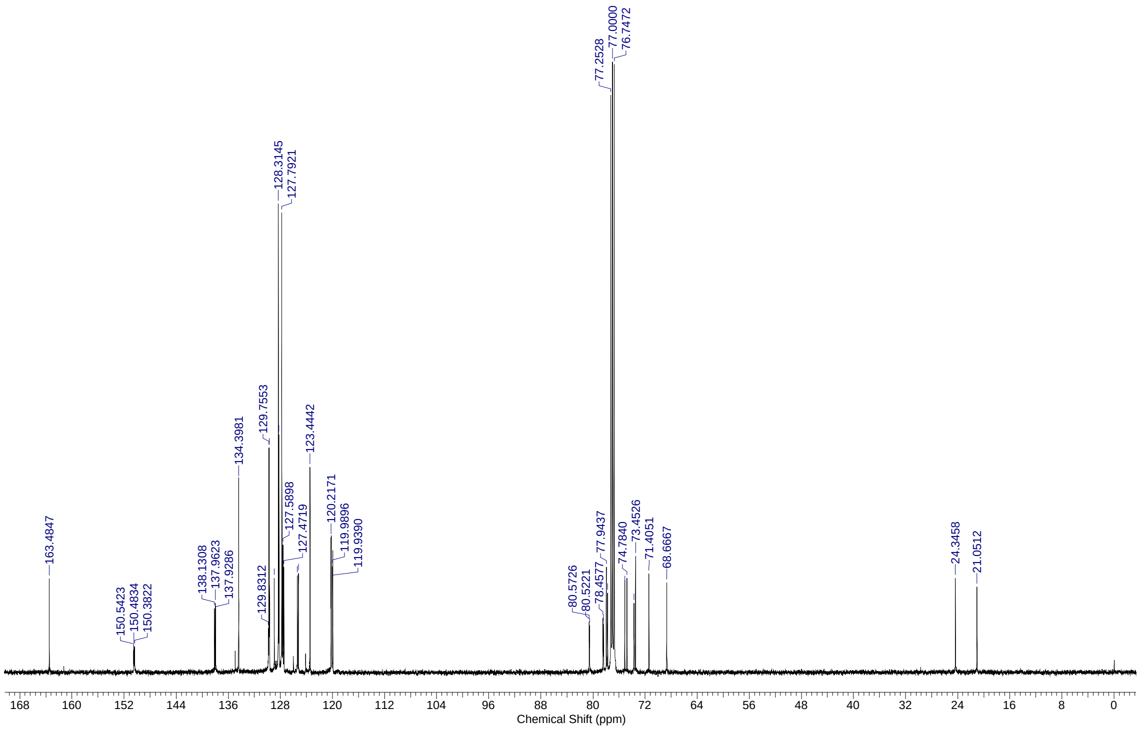

Fig. S6. ^{1}H NMR (500 MHz, $CDCl_3$) and ^{13}C{H} NMR (125.7 MHz, $CDCl_3$) of compound **2**.

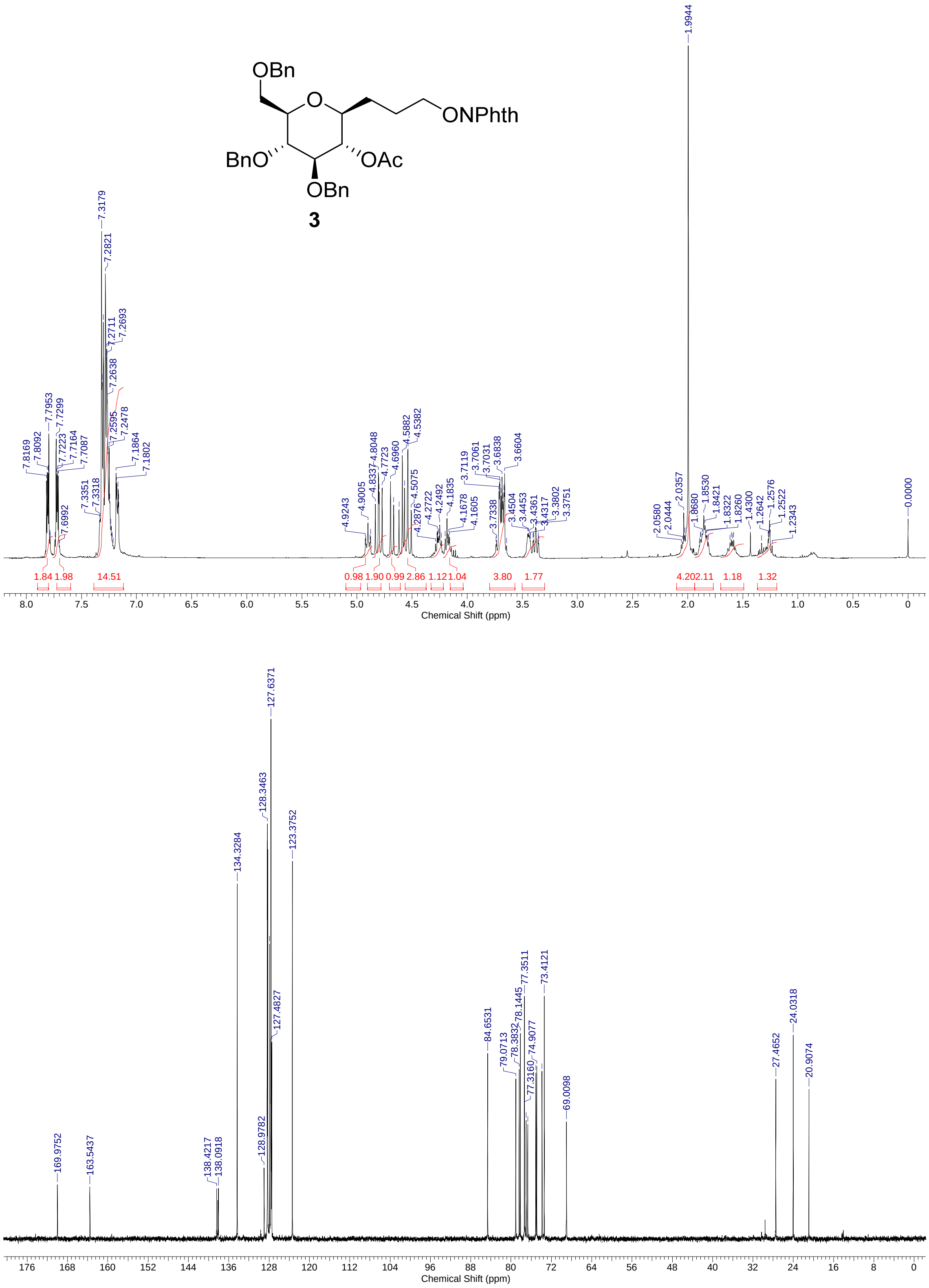

Fig. S7. ^{1}H NMR (400 MHz, $CDCl_3$) and $^{13}C\{H\}$ NMR (100.6 MHz, $CDCl_3$) of compound **3**.

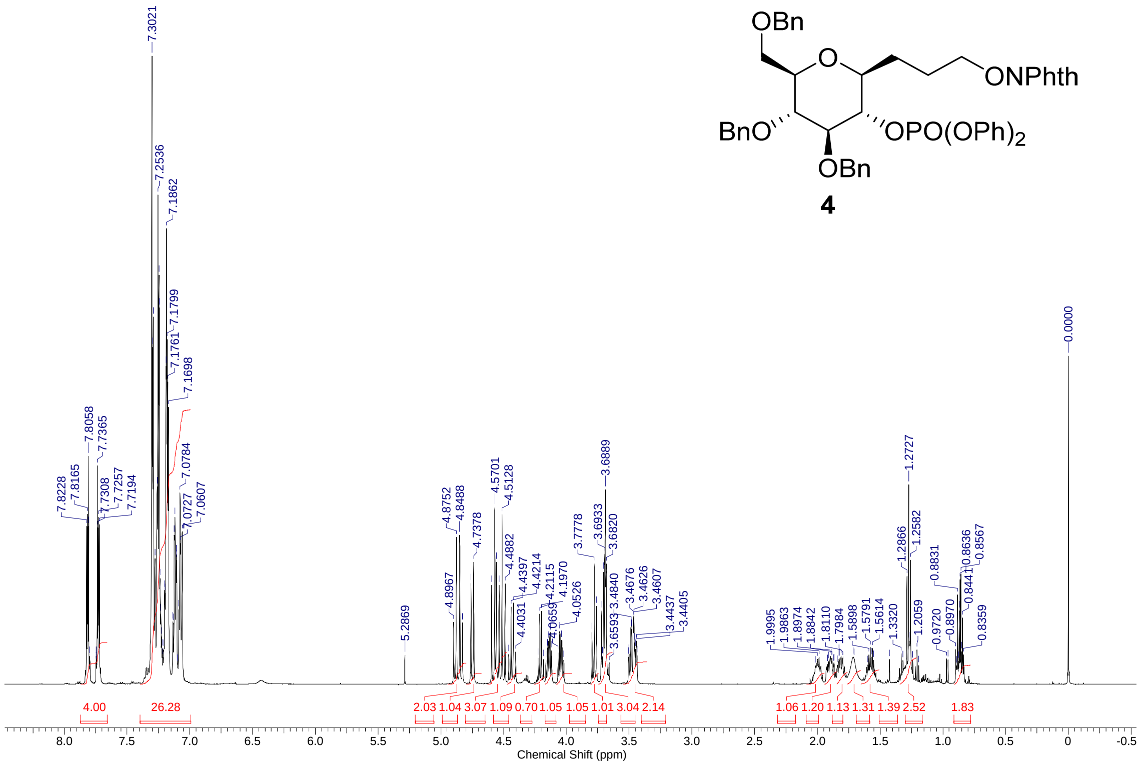

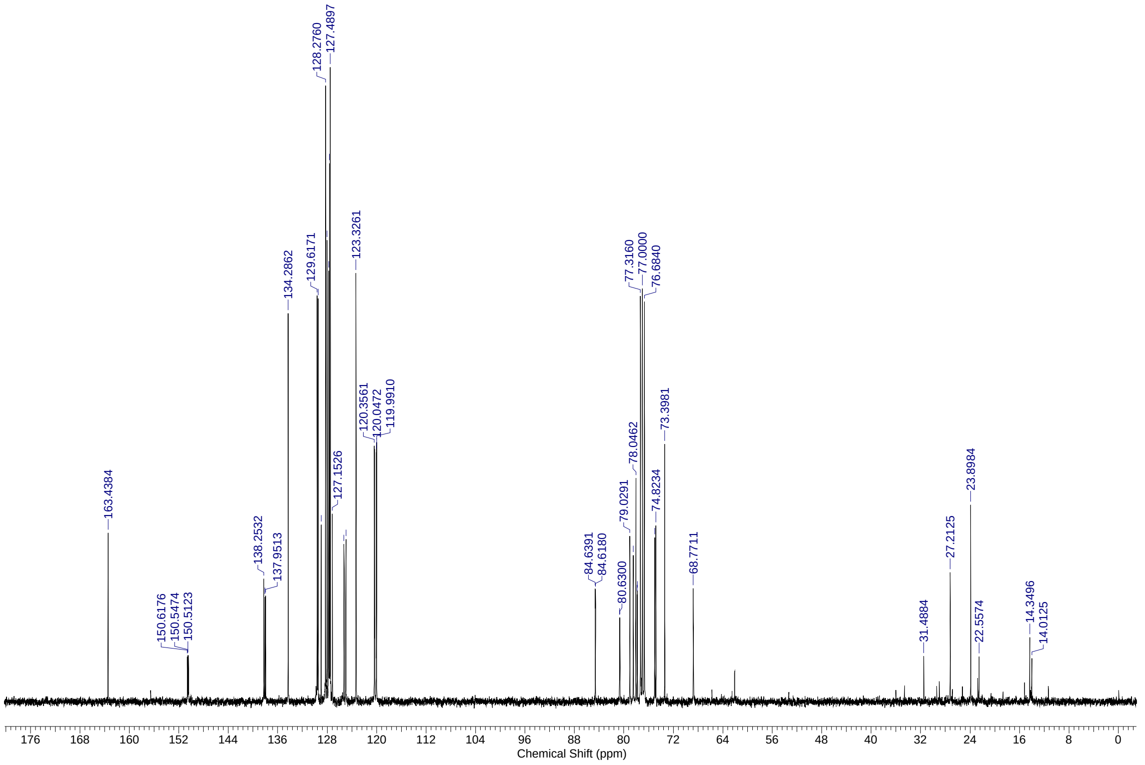

Fig. S8. ^{1}H NMR (500 MHz, $CDCl_3$) and ^{13}C{H} NMR (100.6 MHz, $CDCl_3$) of compound **4**.

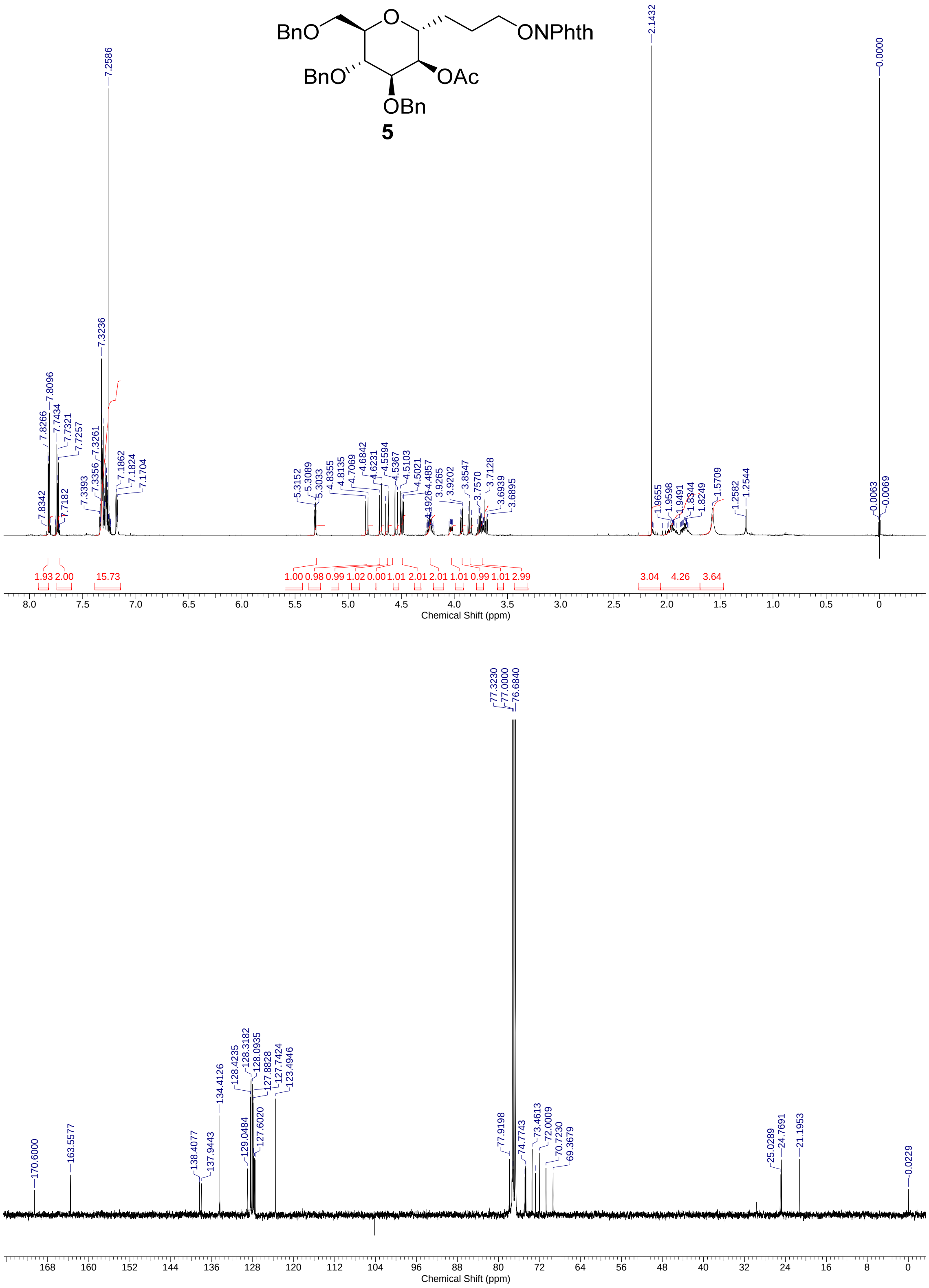

Fig. S9. ^{1}H NMR (500 MHz, $CDCl_3$) and $^{13}C\{H\}$ NMR (100.6 MHz, $CDCl_3$) of compound **5**.

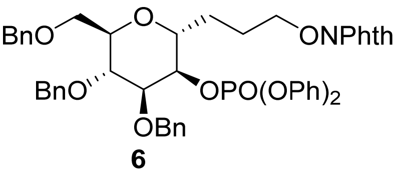

6

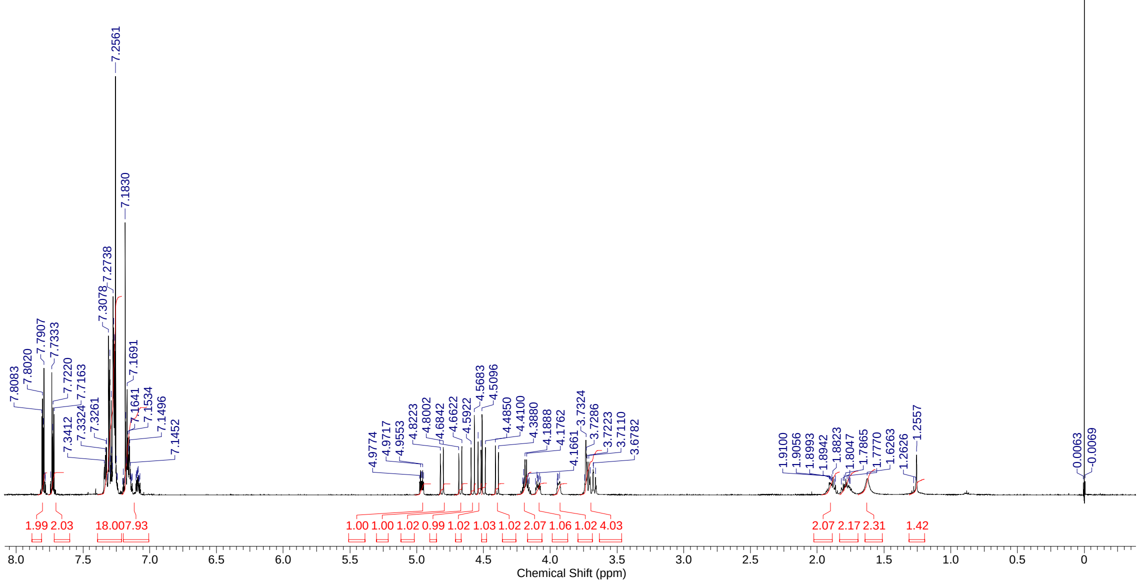

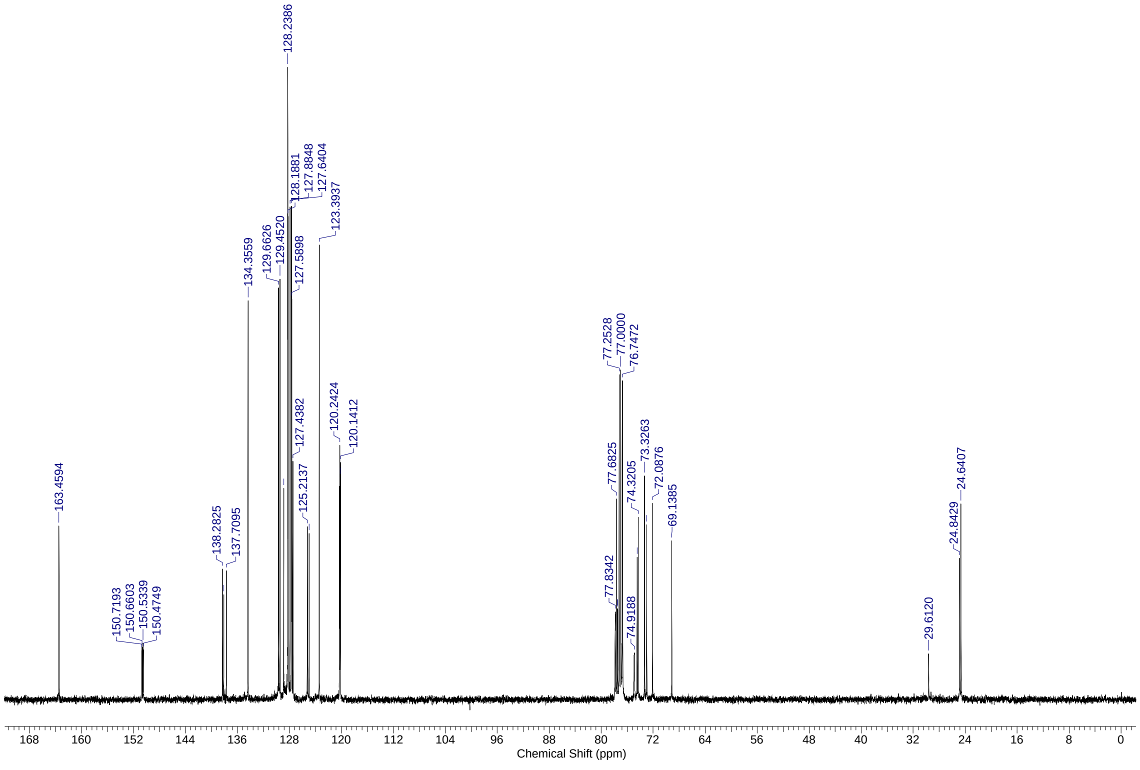

Fig. S10. ^{1}H NMR (500 MHz, $CDCl_3$) and ^{13}C{H} NMR (125.7 MHz, $CDCl_3$) of compound **6**.

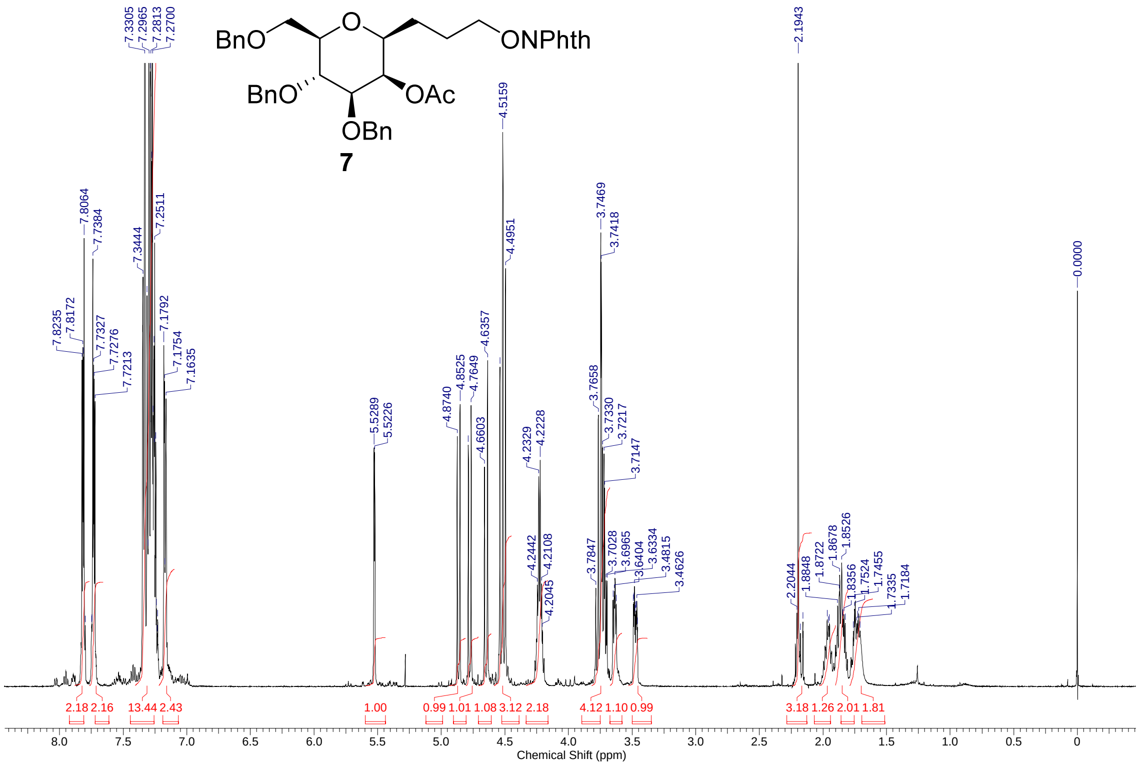

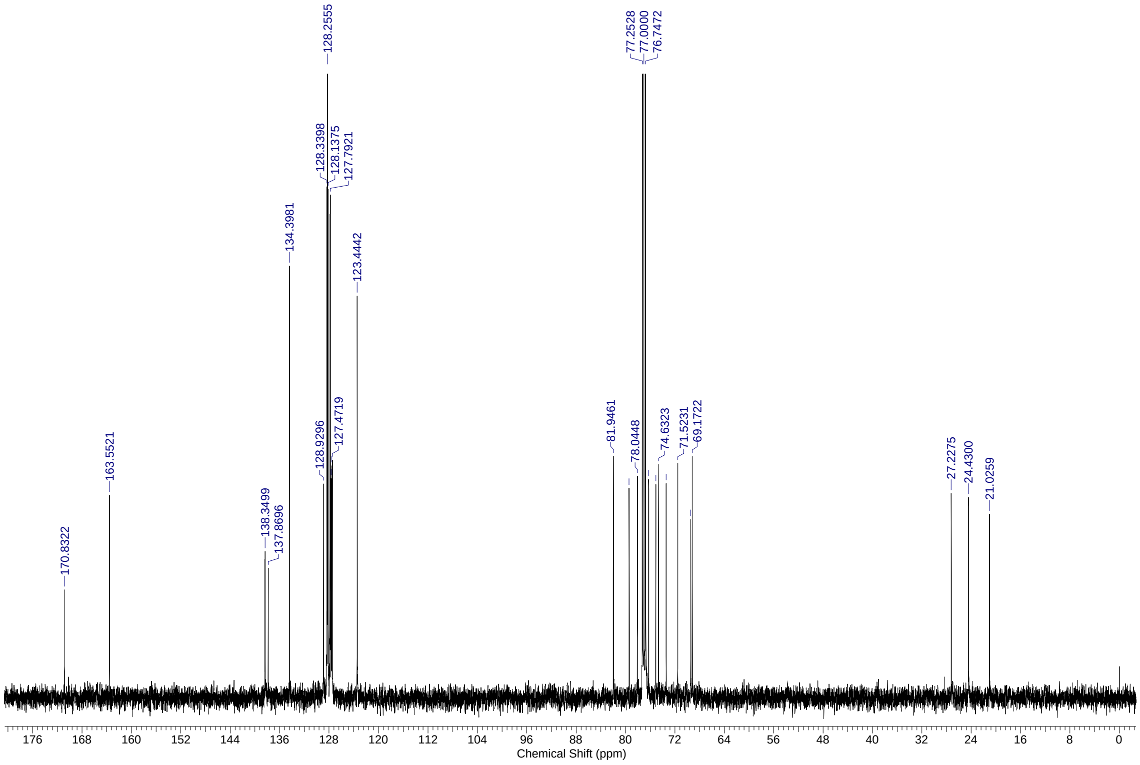

Fig. S11. ^{1}H NMR (500 MHz, $CDCl_3$) and $^{13}C\{H\}$ NMR (125.7 MHz, $CDCl_3$) of compound **7**.

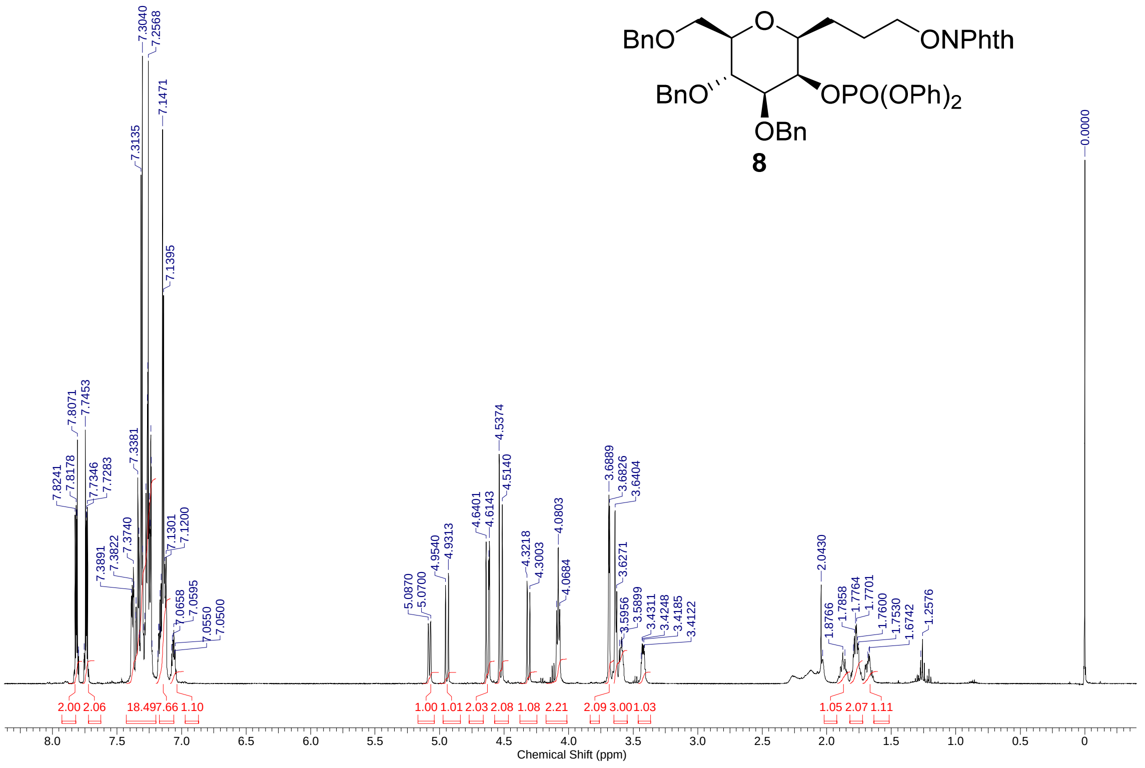

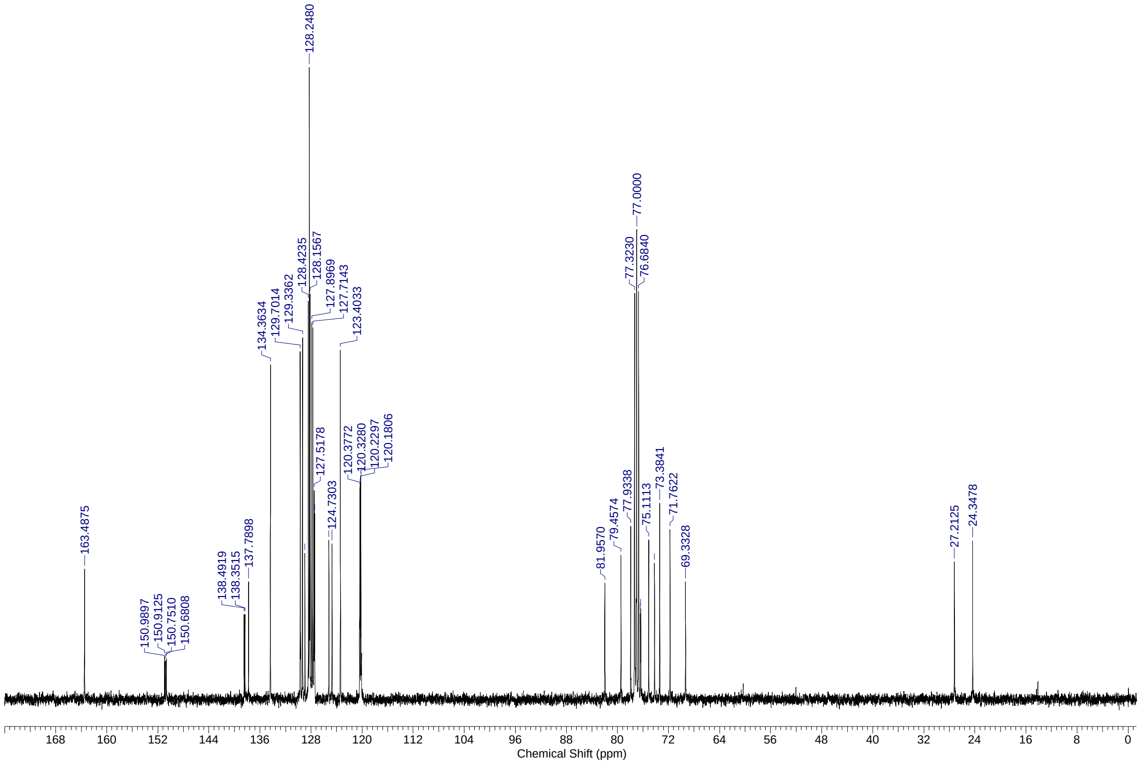

Fig. S12. ^{1}H NMR (500 MHz, $CDCl_3$) and ^{13}C{H} NMR (100.6 MHz, $CDCl_3$) of compound **8**.

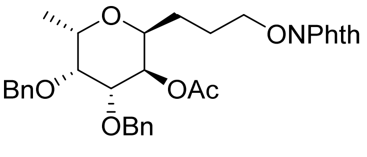

9

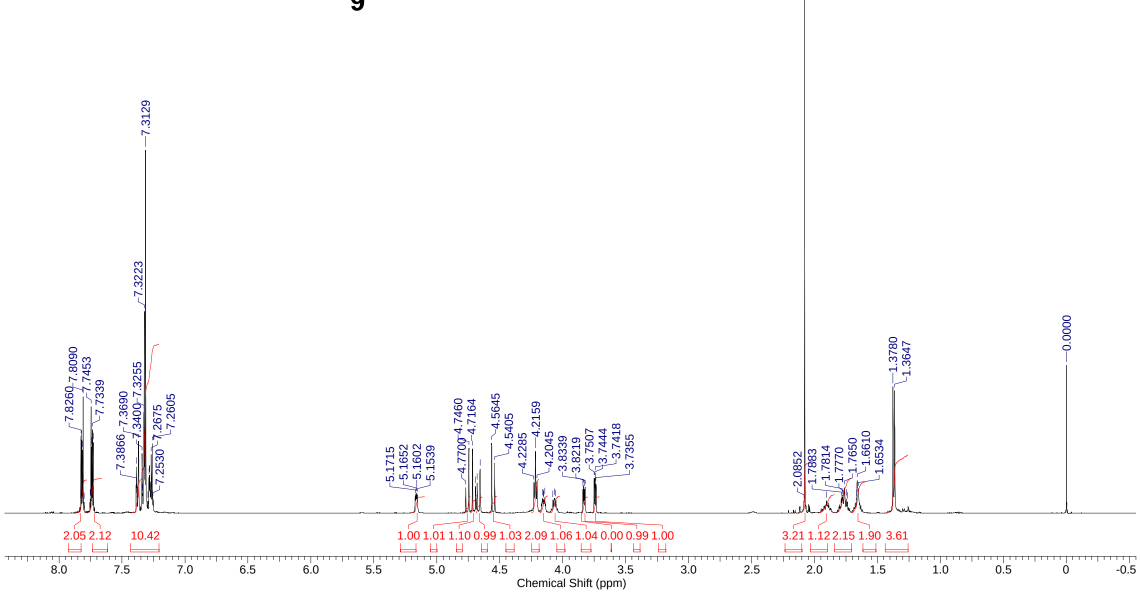

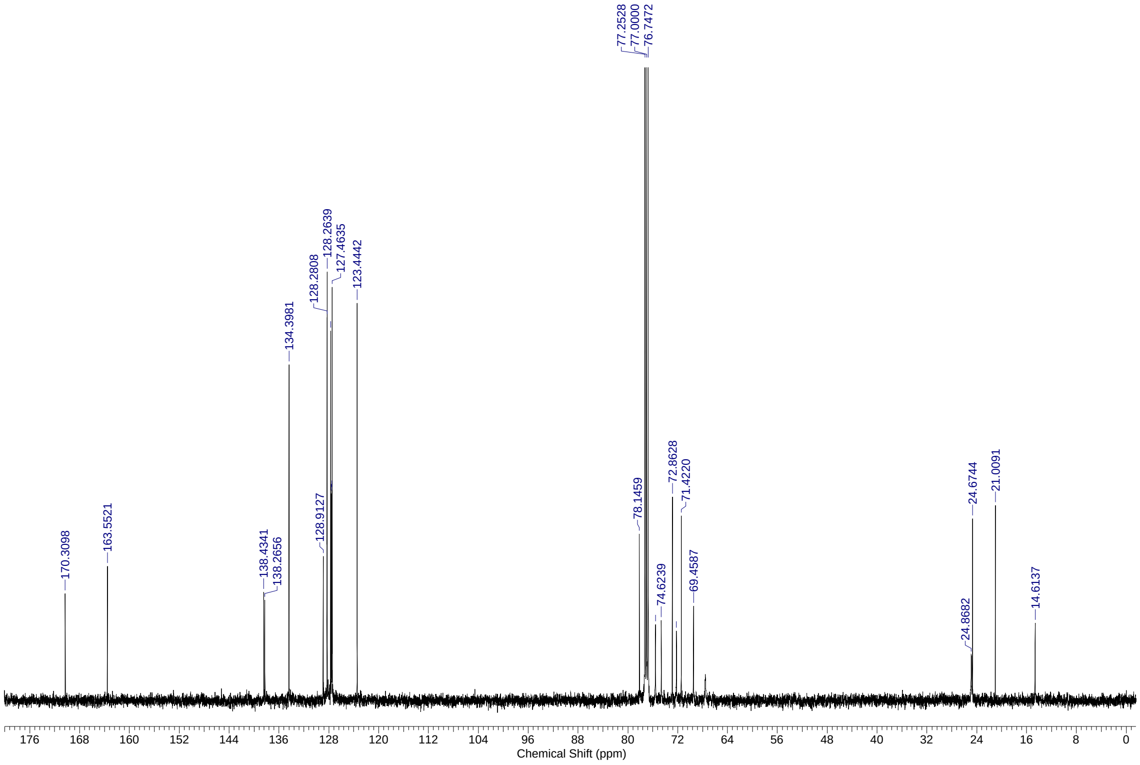

Fig. S13. ^{1}H NMR (500 MHz, $CDCl_3$) and ^{13}C{H} NMR (125.7 MHz, $CDCl_3$) of compound **9**.

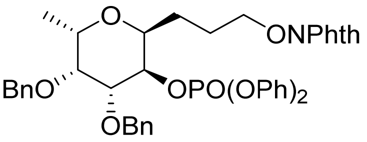

10

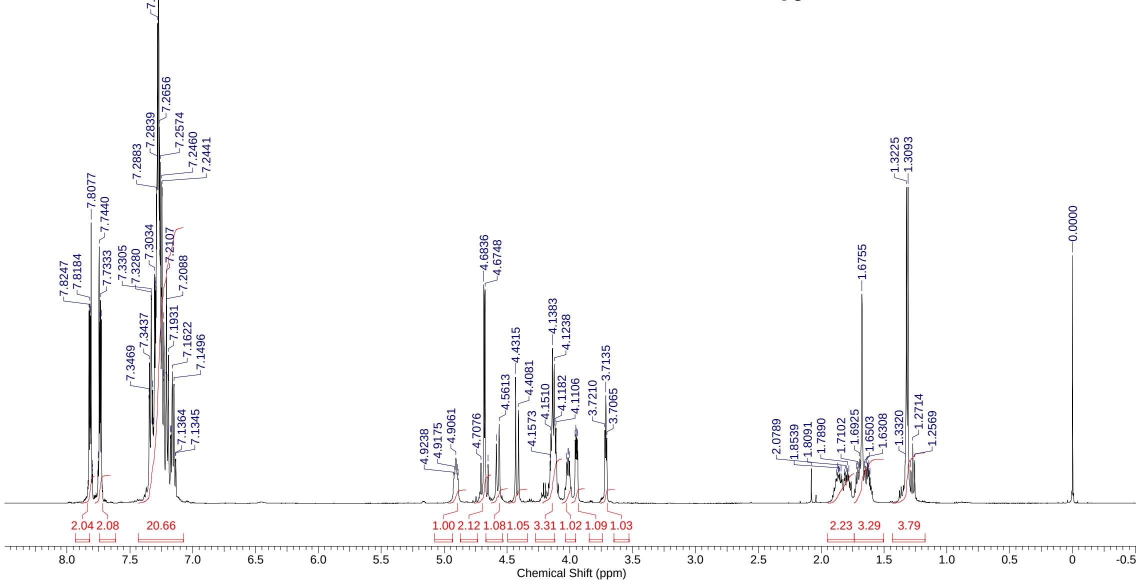

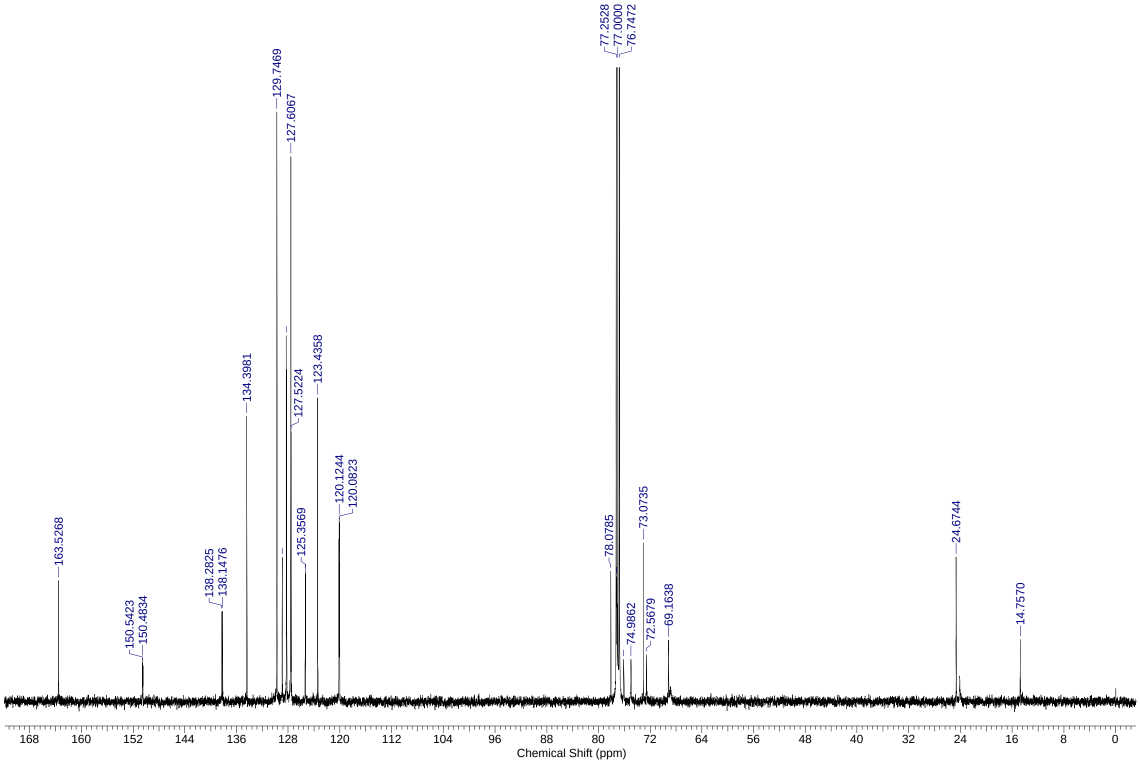

Fig. S14. ^{1}H NMR (500 MHz, $CDCl_3$) and $^{13}C\{H\}$ NMR (125.7 MHz, $CDCl_3$) of compound **10**.

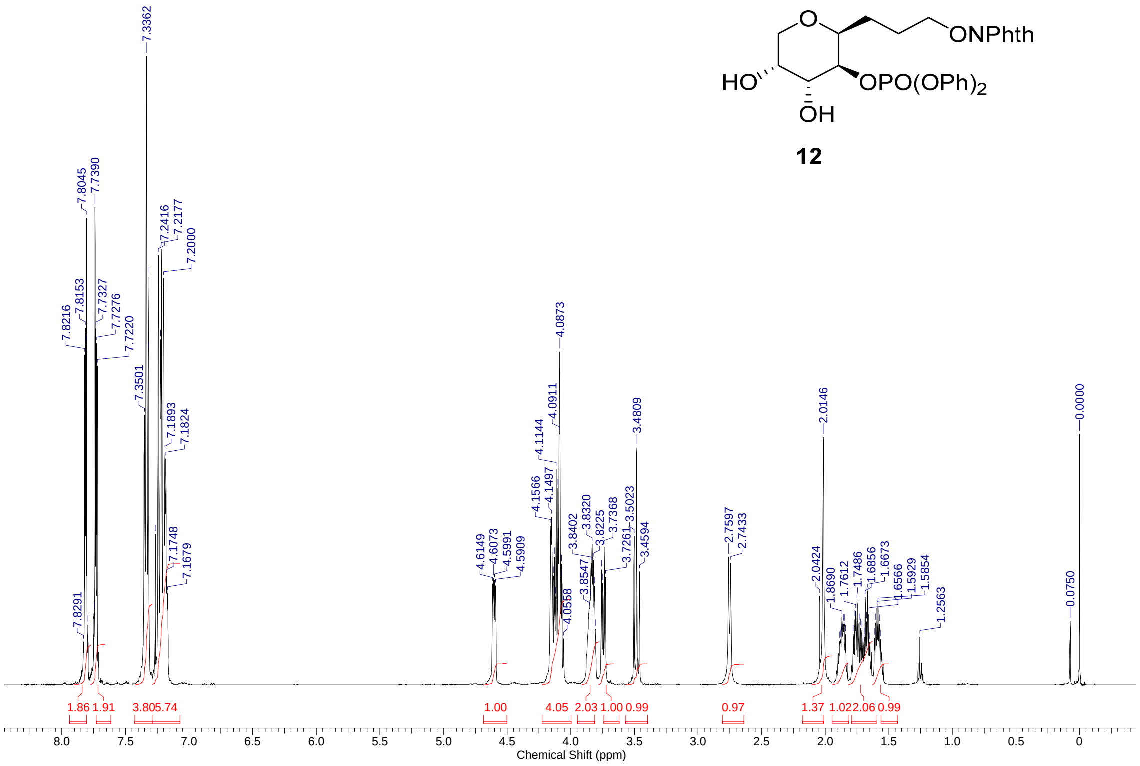

12

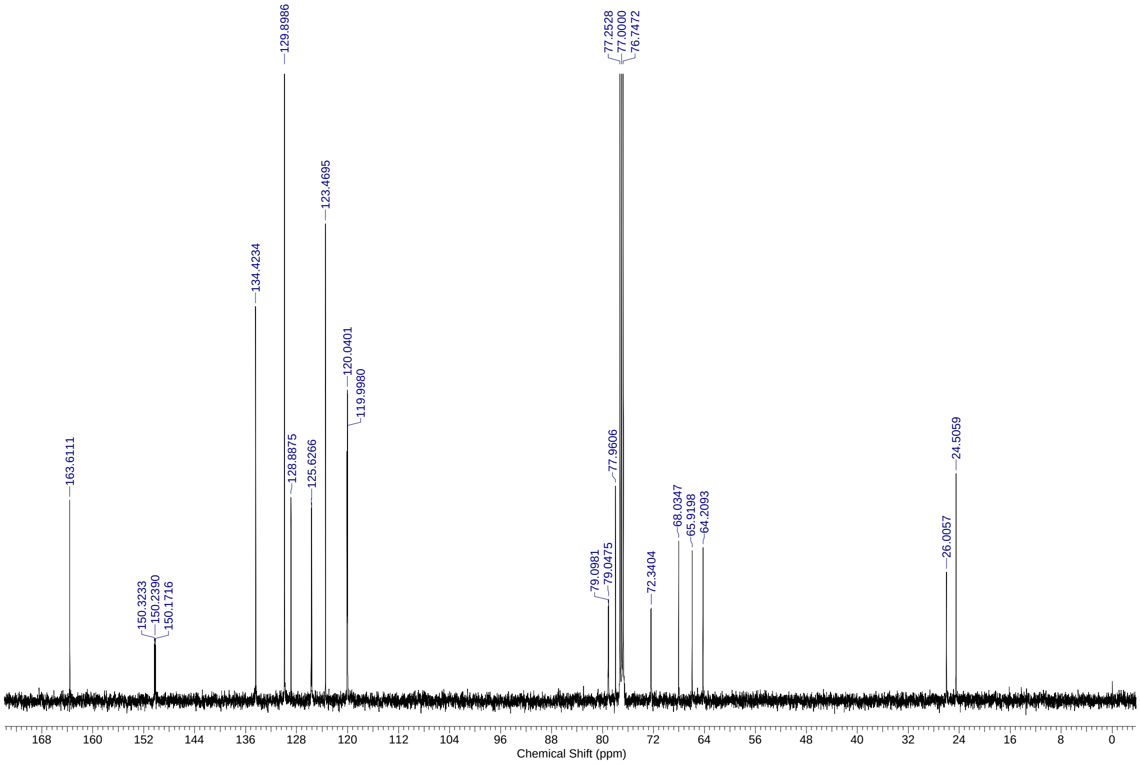

Fig. S15. ^{1}H NMR (500 MHz, $CDCl_3$) and ^{13}C{H} NMR (125.7 MHz, $CDCl_3$) of compound **12**.

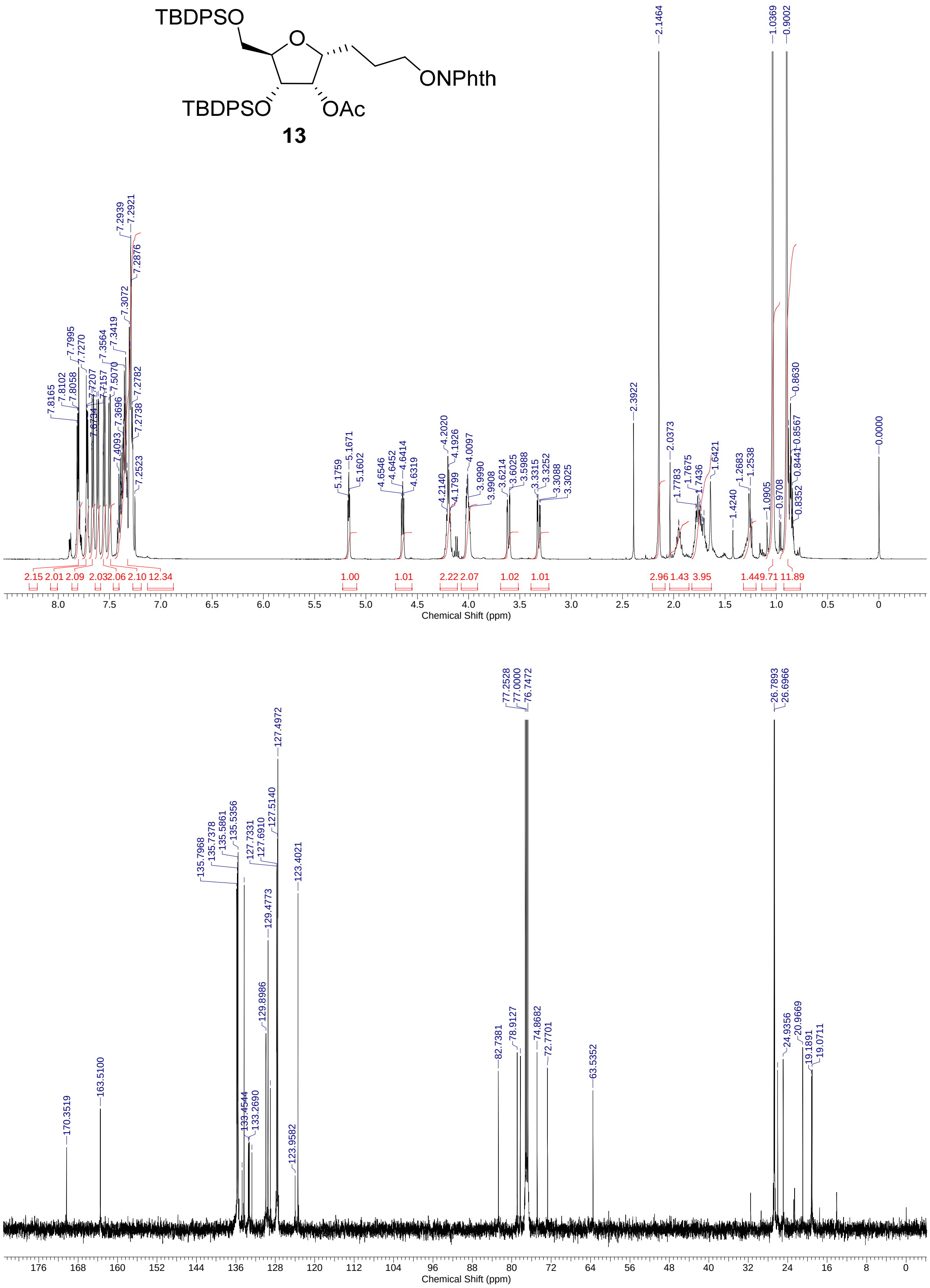

Fig. S16. ^{1}H NMR (500 MHz, $CDCl_3$) and ^{13}C{H} NMR (125.7 MHz, $CDCl_3$) of compound **13**.

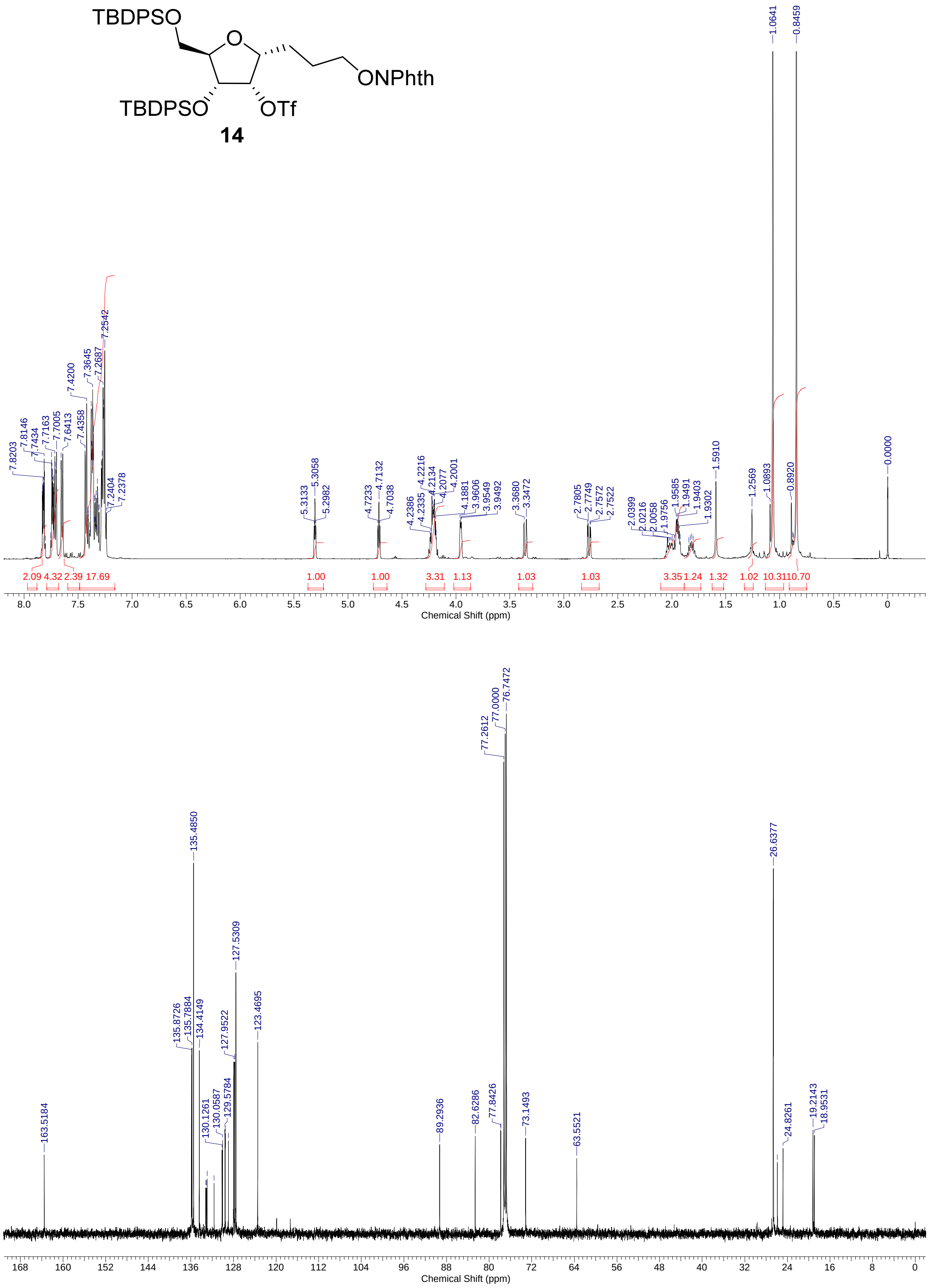

Fig. S17. ^{1}H NMR (500 MHz, $CDCl_3$) and ^{13}C{H} NMR (125.7 MHz, $CDCl_3$) of compound **14**.

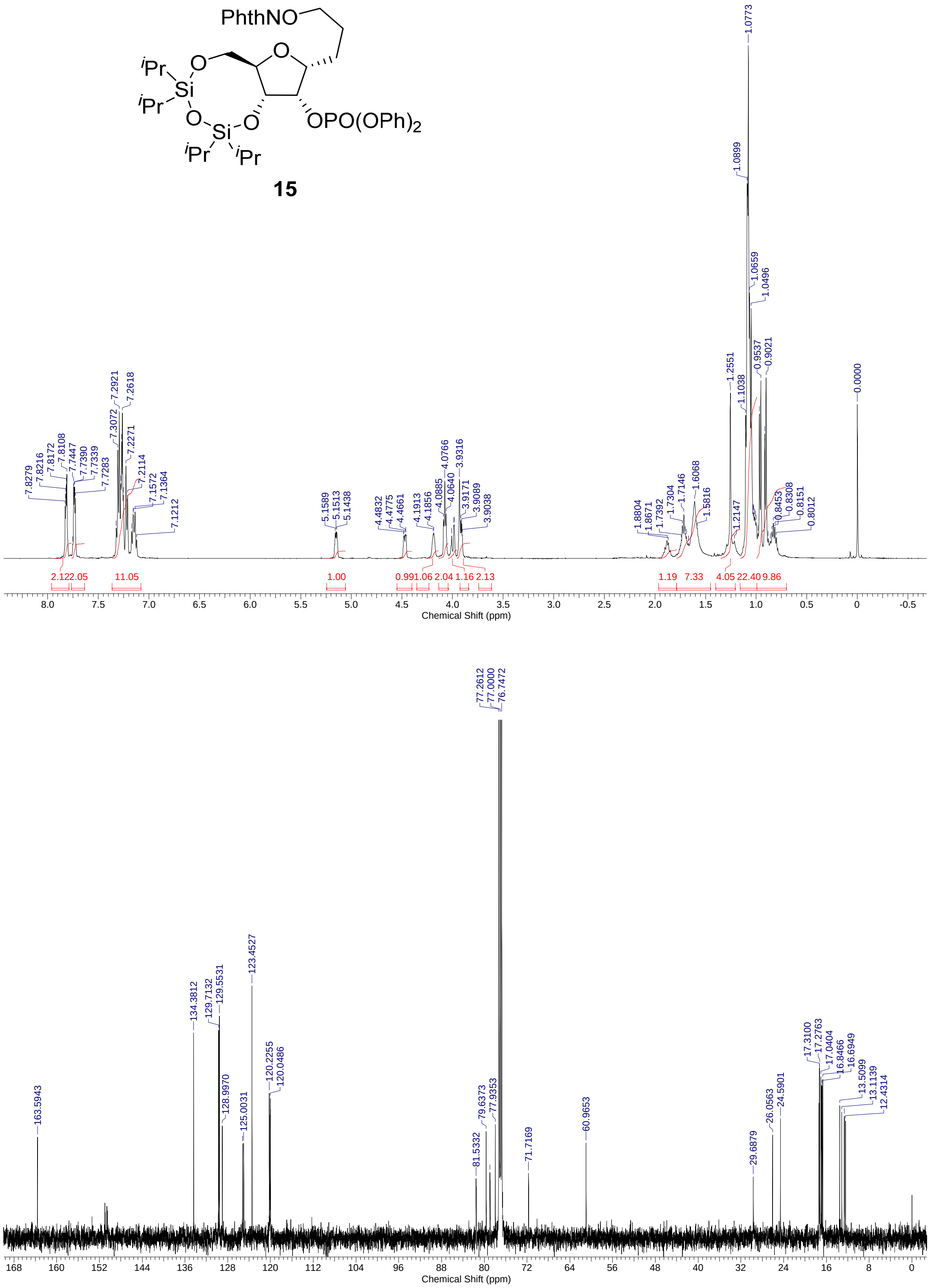

Fig. S18. ^{1}H NMR (500 MHz, $CDCl_3$) and ^{13}C{H} NMR (125.7 MHz, $CDCl_3$) of compound **15**.

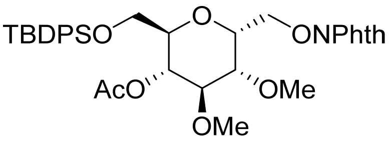

16

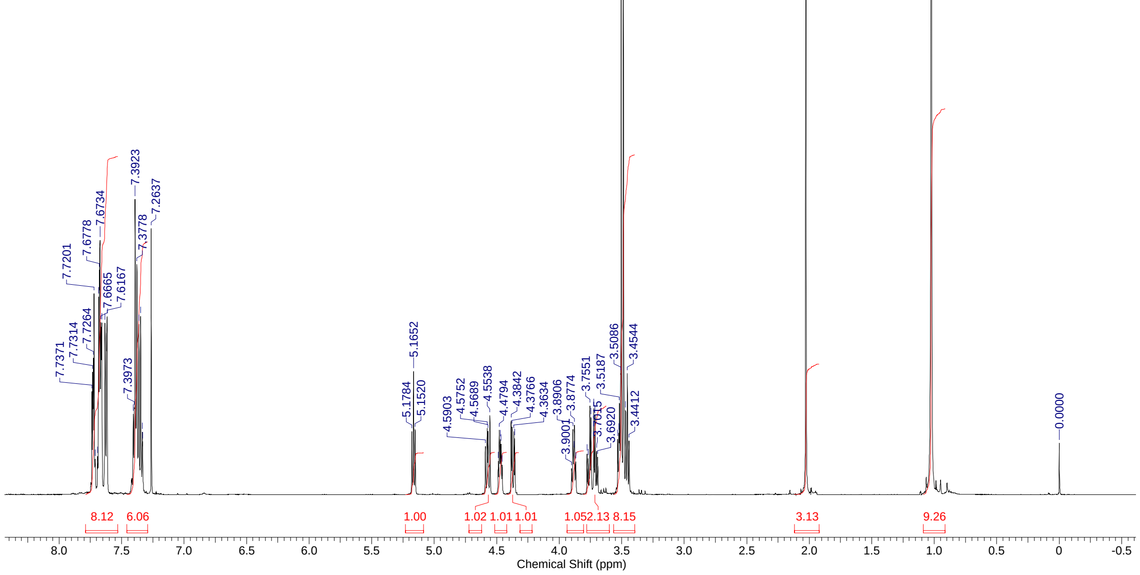

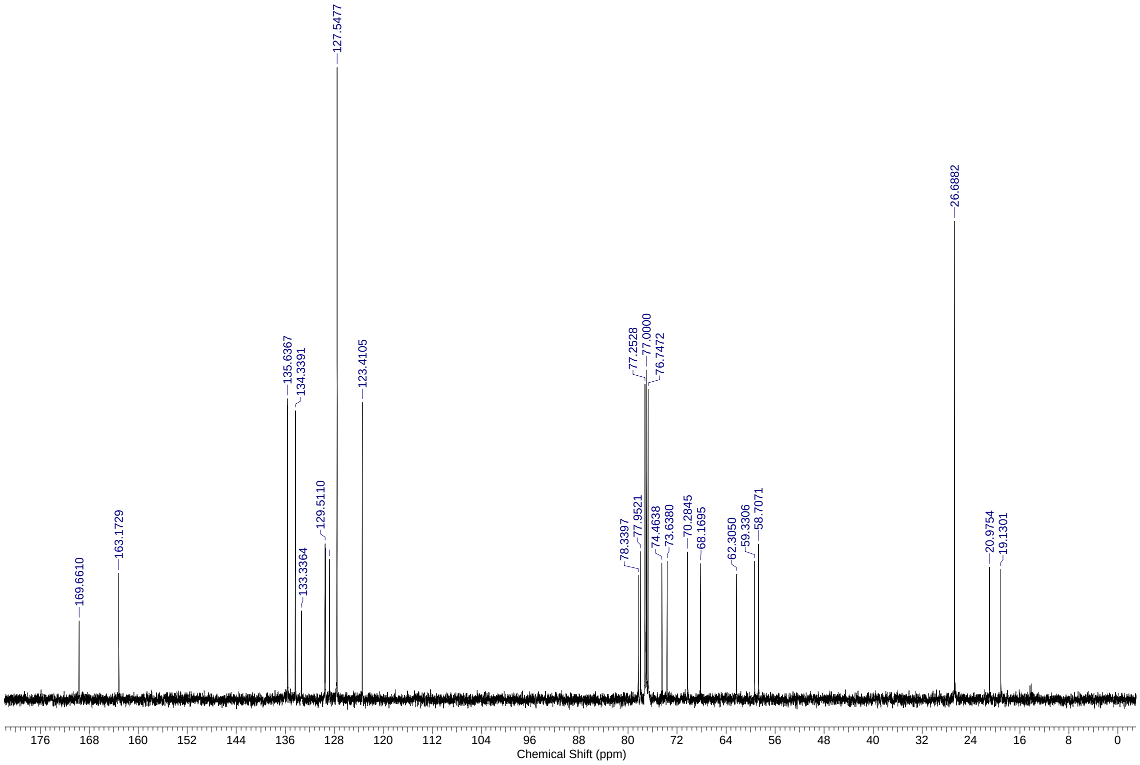

Fig. S19. ^{1}H NMR (500 MHz, $CDCl_3$) and $^{13}C\{H\}$ NMR (125.7 MHz, $CDCl_3$) of compound **16**.

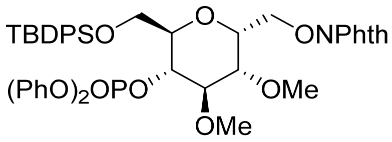

17

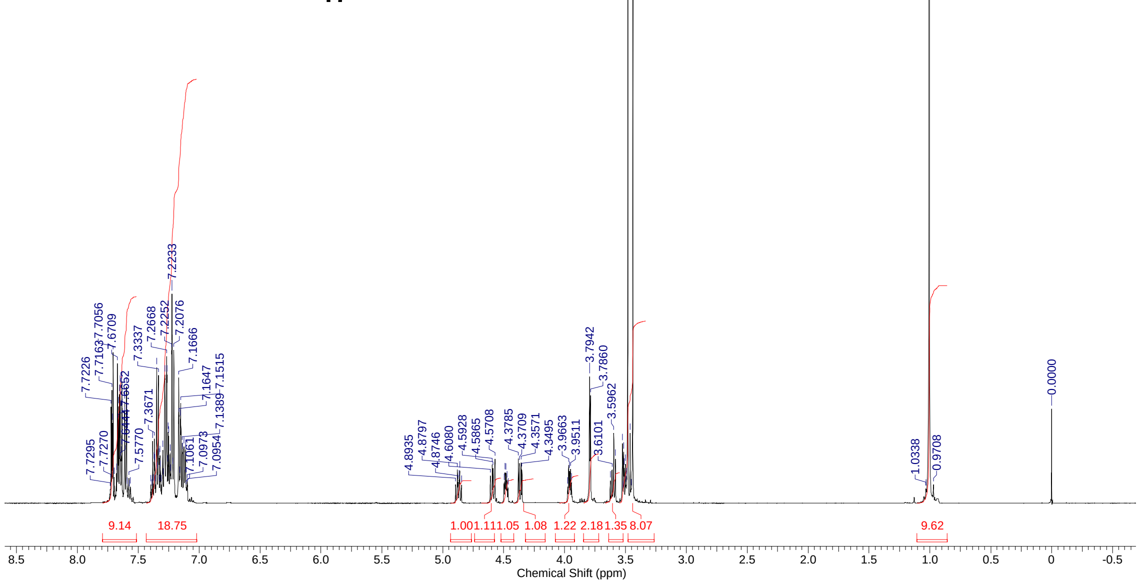

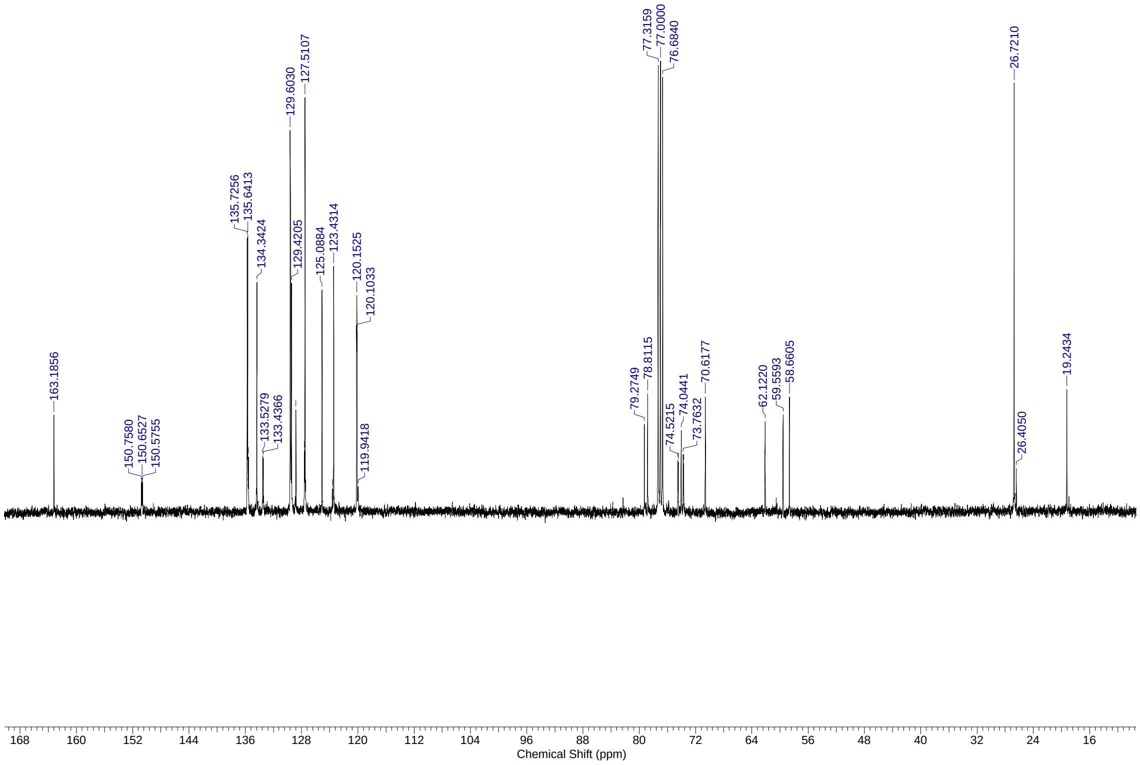

Fig. S20. ^{1}H NMR (500 MHz, $CDCl_3$) and $^{13}C\{H\}$ NMR (100.6 MHz, $CDCl_3$) of compound **17**.

18

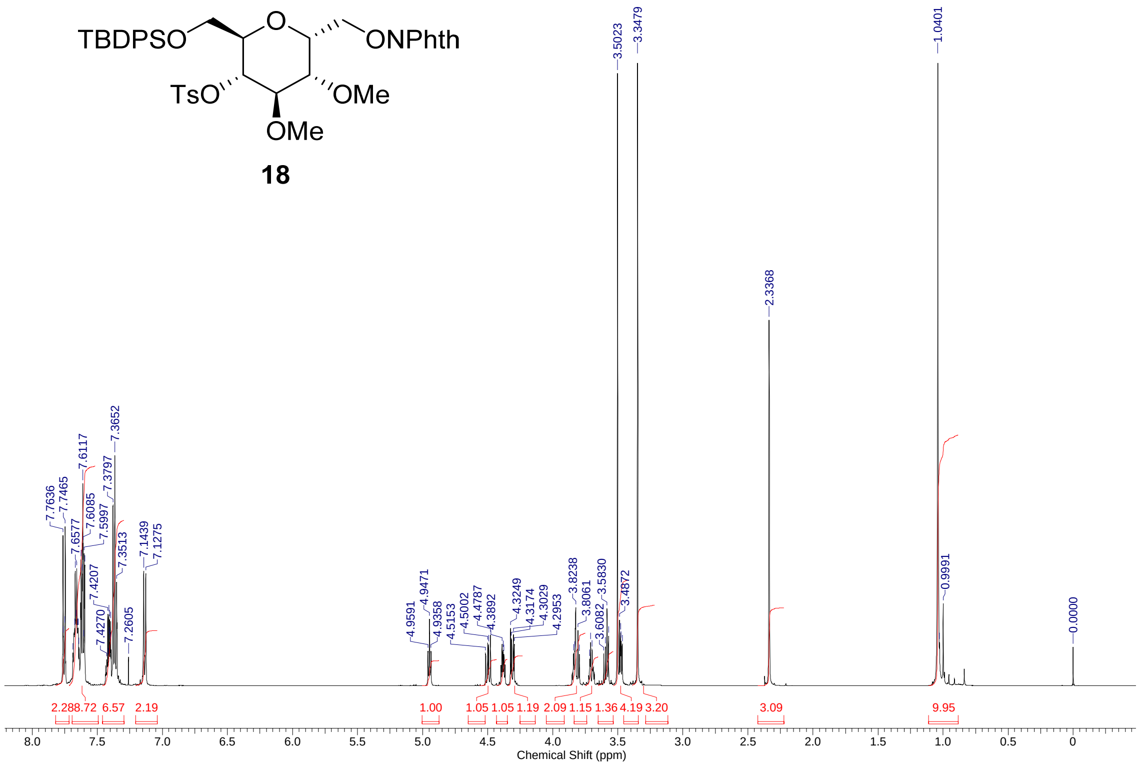

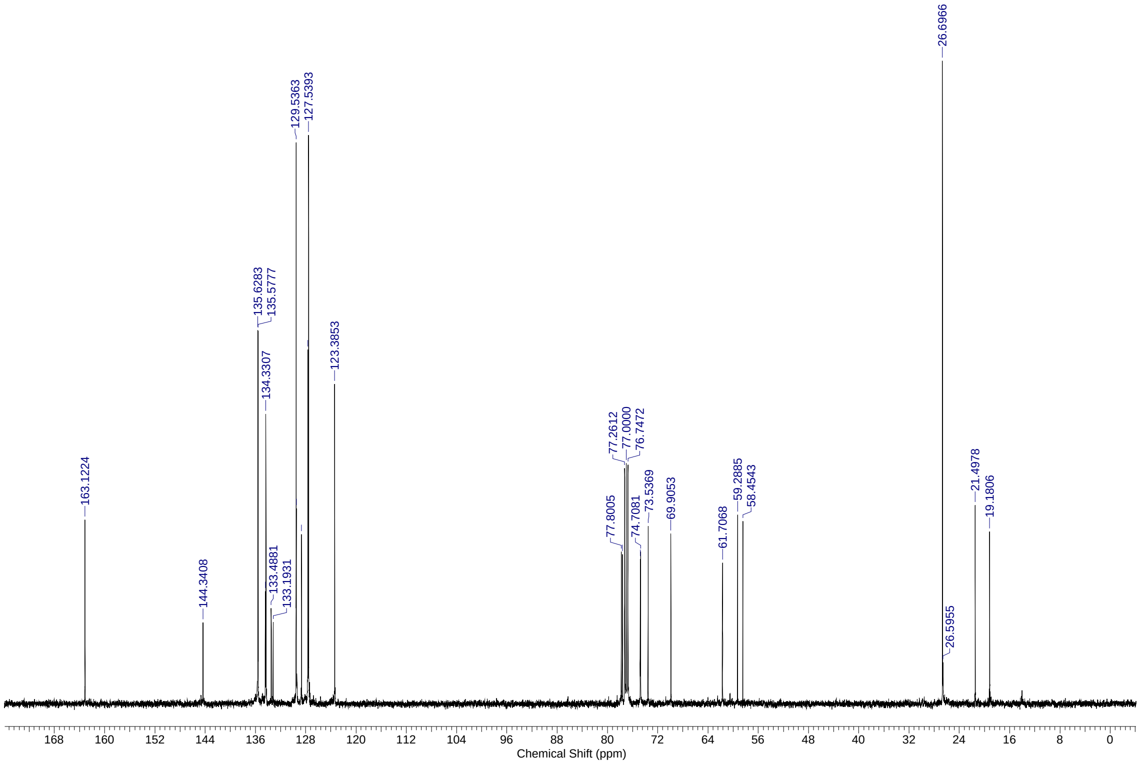

Fig. S21. ^{1}H NMR (500 MHz, $CDCl_3$) and ^{13}C{H} NMR (125.7 MHz, $CDCl_3$) of compound **18**.

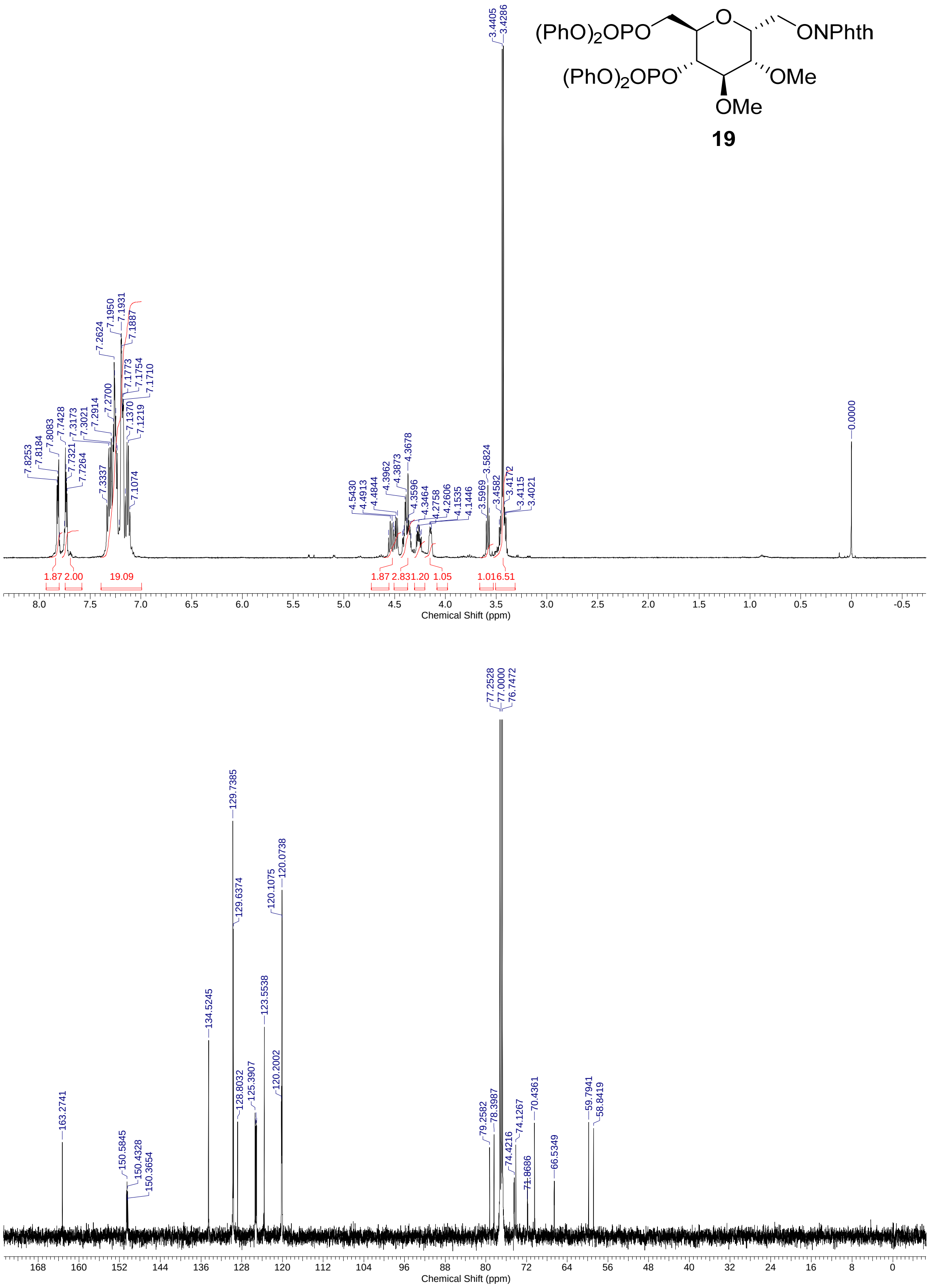

Fig. S22. ^{1}H NMR (500 MHz, $CDCl_3$) and ^{13}C{H} NMR (125.7 MHz, $CDCl_3$) of compound **19**.

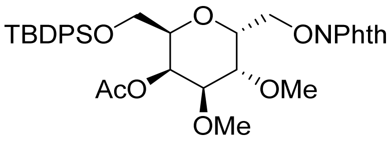

20

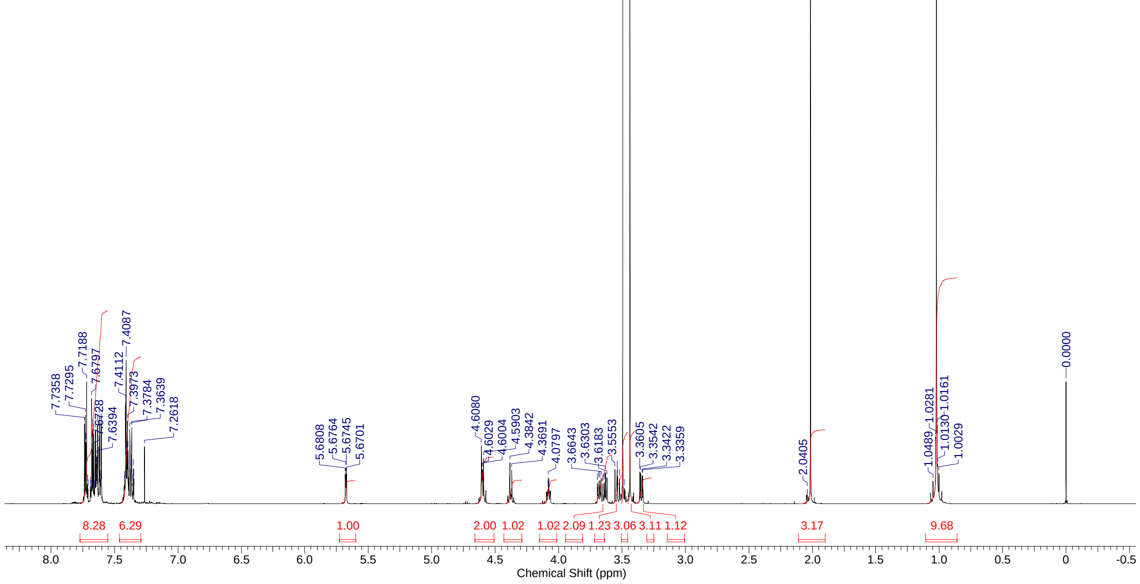

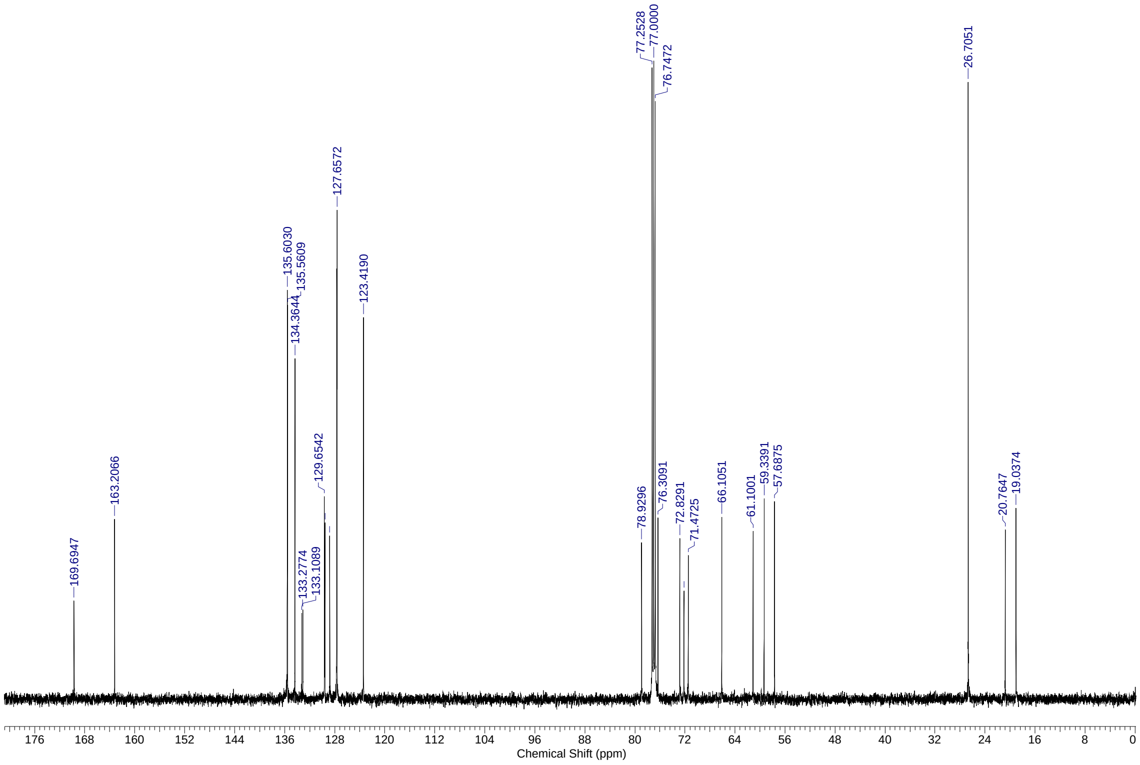

Fig. S23. ^{1}H NMR (500 MHz, $CDCl_3$) and ^{13}C{H} NMR (125.7 MHz, $CDCl_3$) of compound **20**.

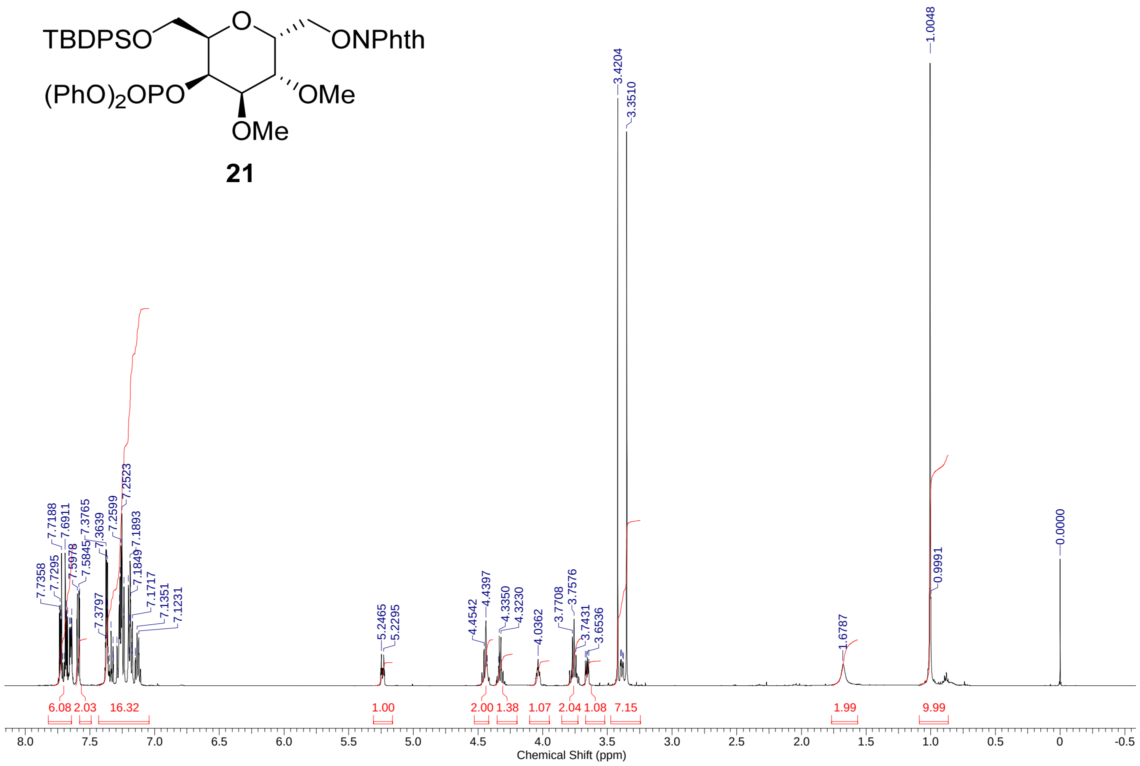

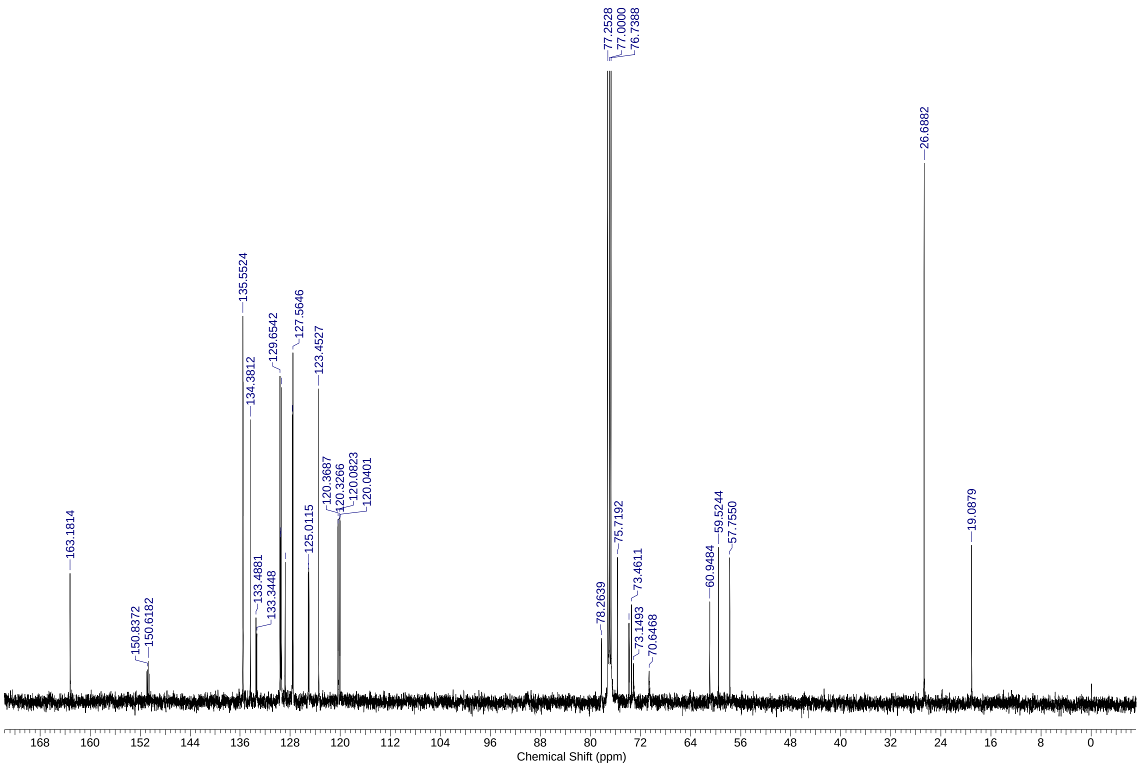

Fig. S24. ^{1}H NMR (500 MHz, $CDCl_3$) and $^{13}C\{H\}$ NMR (125.7 MHz, $CDCl_3$) of compound **21**.

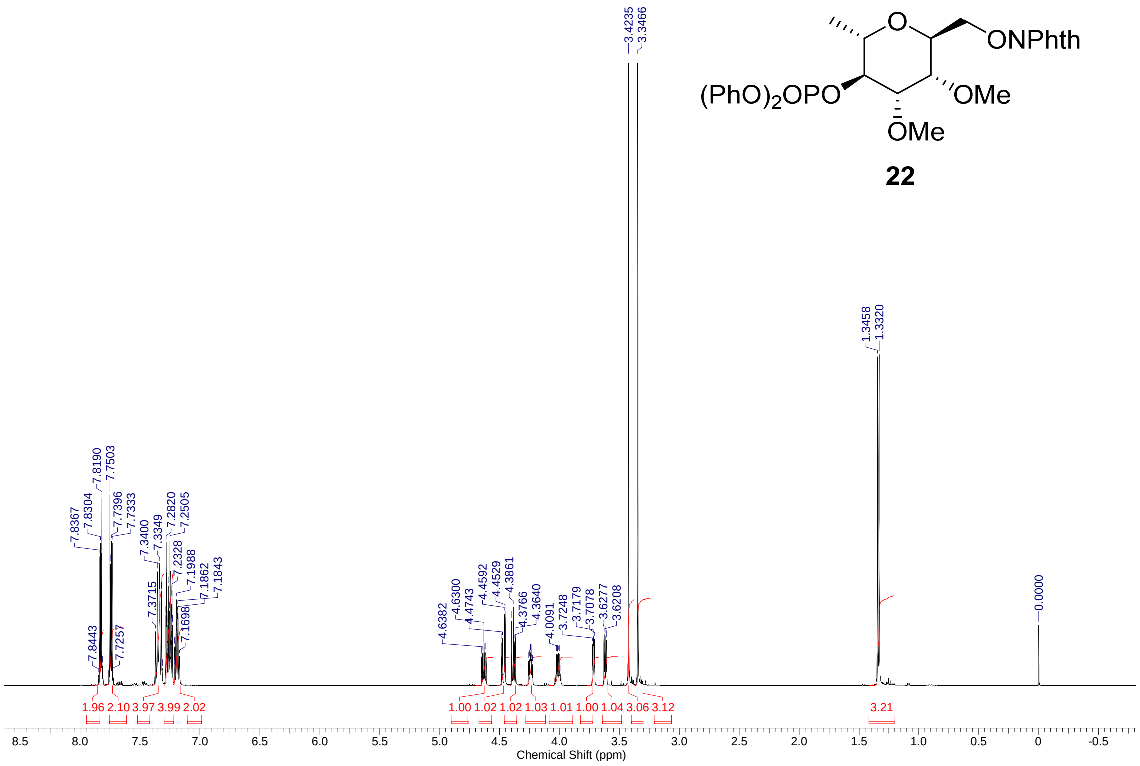

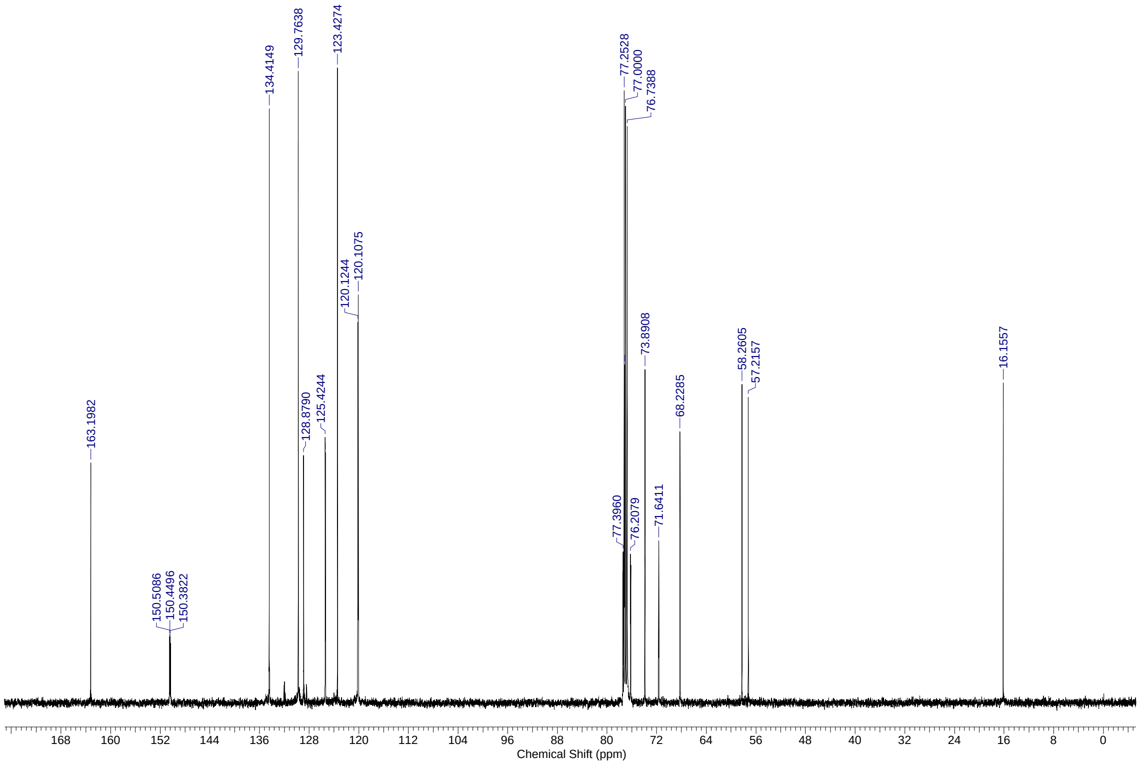

Fig. S25. ^{1}H NMR (500 MHz, $CDCl_3$) and $^{13}C\{H\}$ NMR (125.7 MHz, $CDCl_3$) of compound **22**.

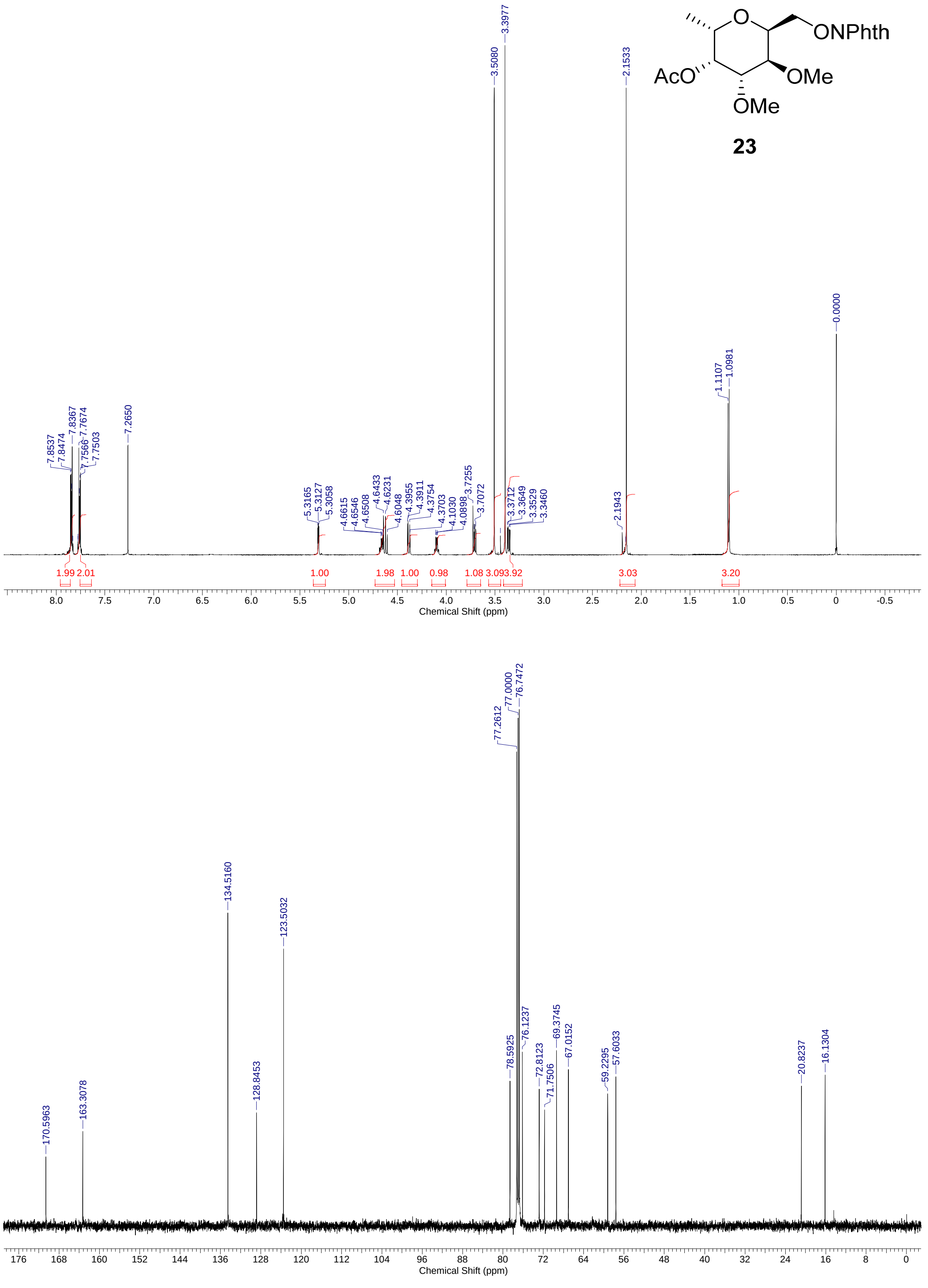

Fig. S26. ^{1}H NMR (500 MHz, $CDCl_3$) and $^{13}C\{H\}$ NMR (125.7 MHz, $CDCl_3$) of compound **23**.

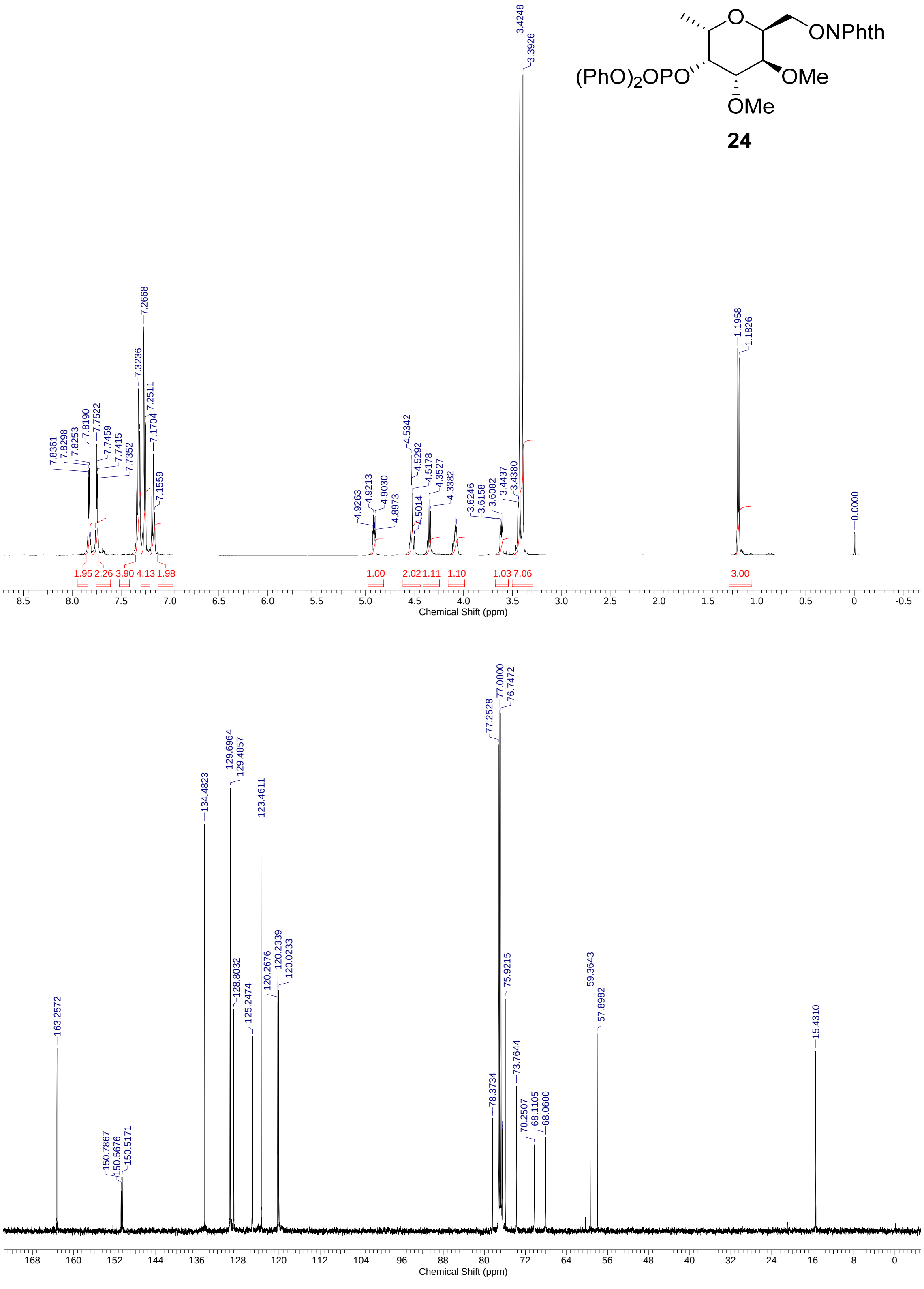

Fig. S27. ^{1}H NMR (500 MHz, $CDCl_3$) and ^{13}C{H} NMR (125.7 MHz, $CDCl_3$) of compound **24**.

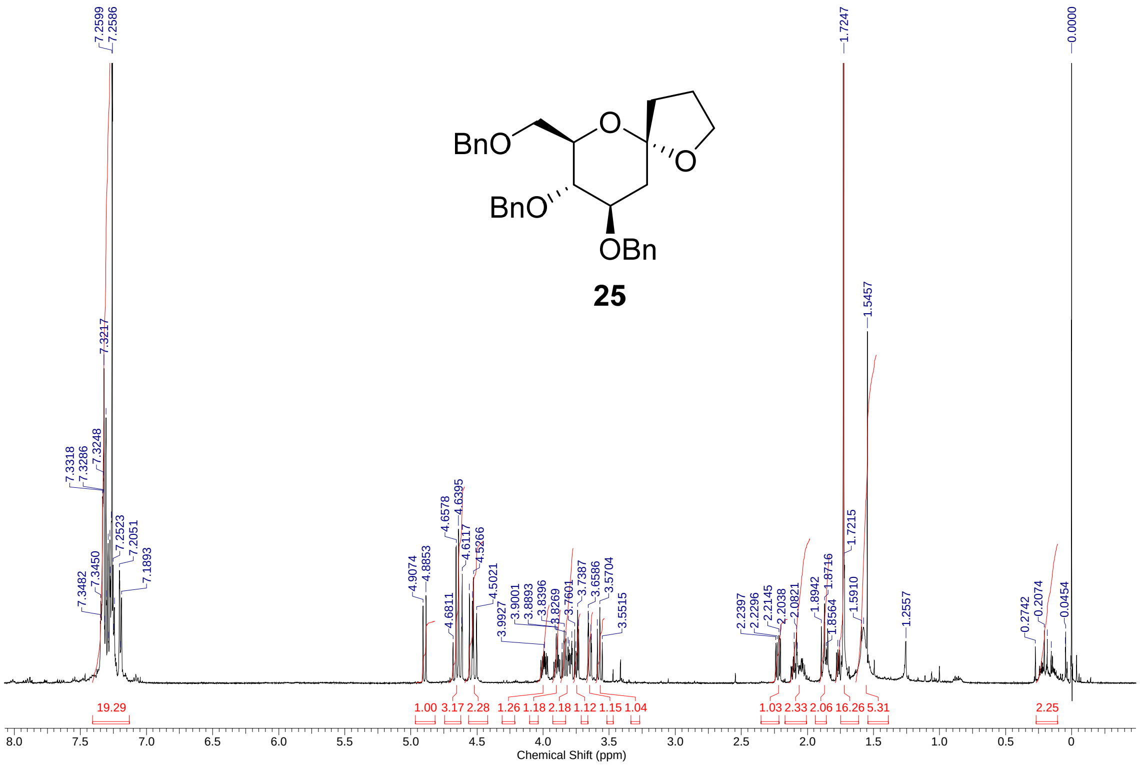

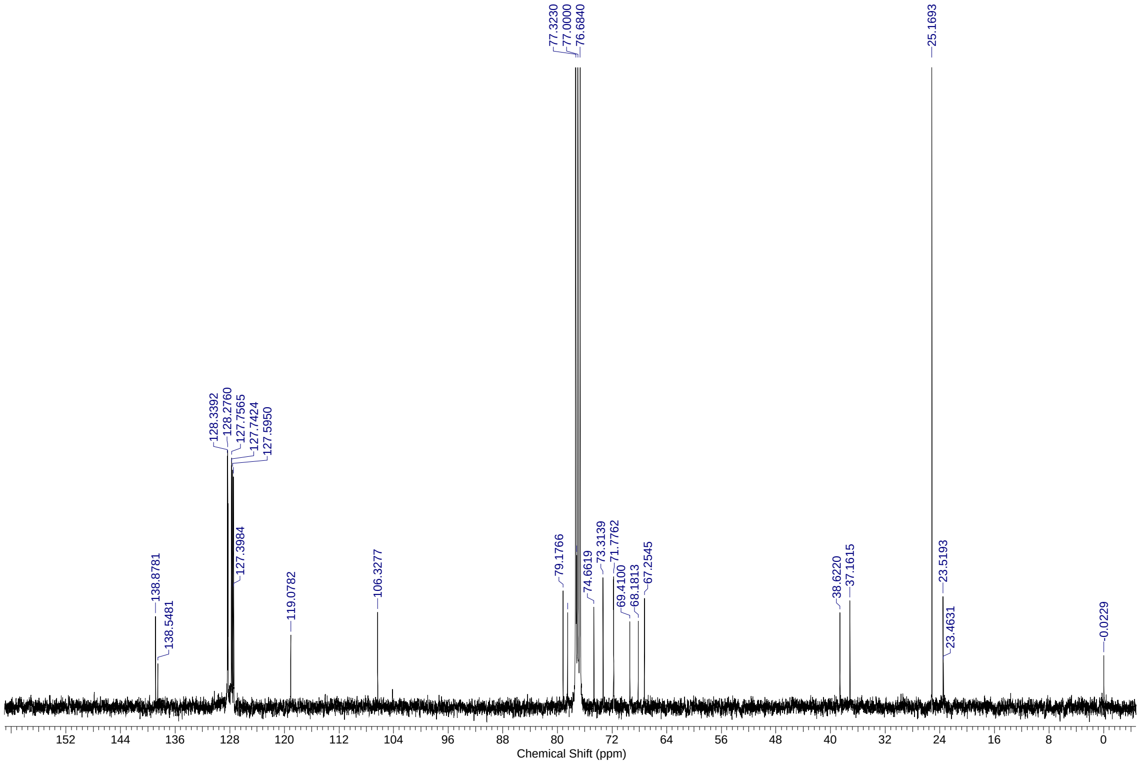

Fig. S28. ^{1}H NMR (500 MHz, $CDCl_3$) and ^{13}C{H} NMR (100.6 MHz, $CDCl_3$) of compound **25**.

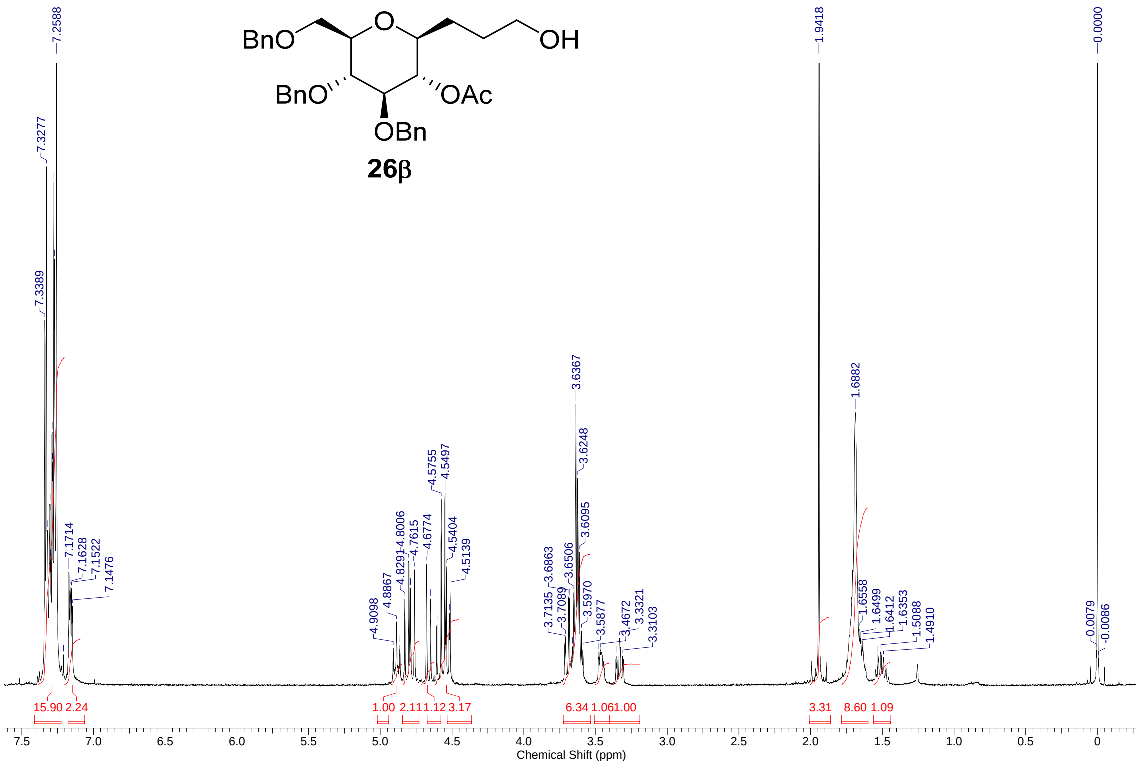

26β

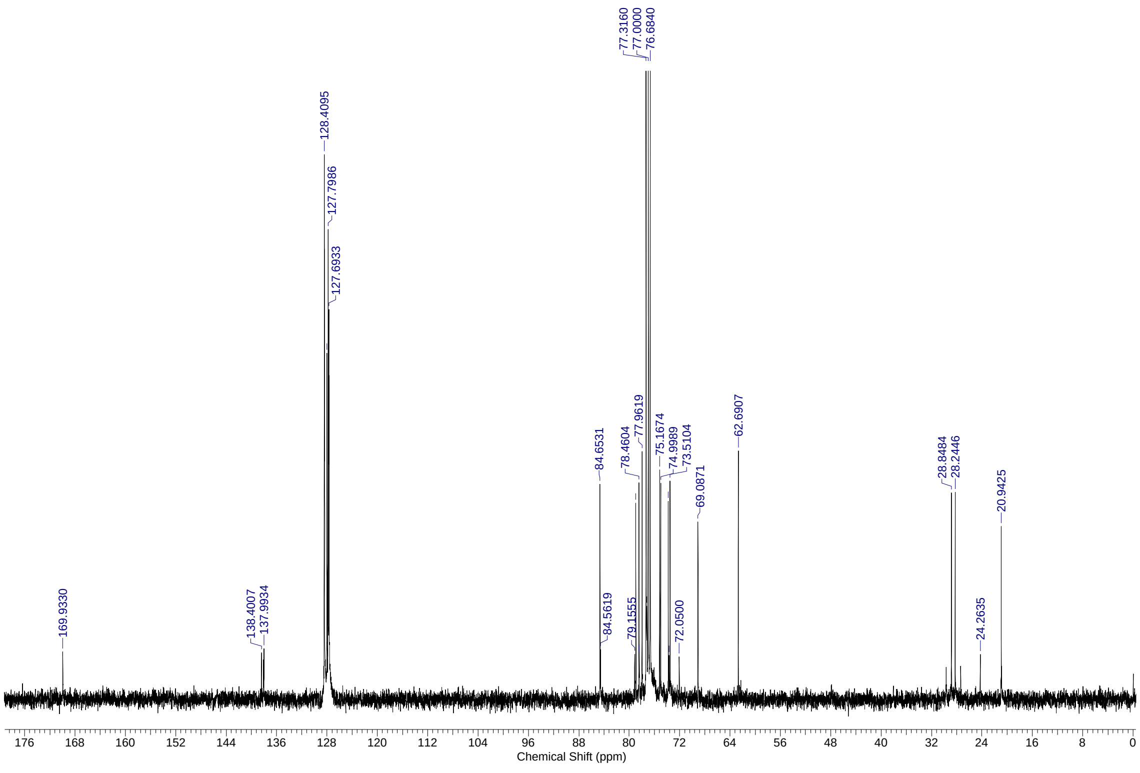

Fig. S29. ^{1}H NMR (400 MHz, $CDCl_3$) and $^{13}C\{H\}$ NMR (100.6 MHz, $CDCl_3$) of compound **26β**.

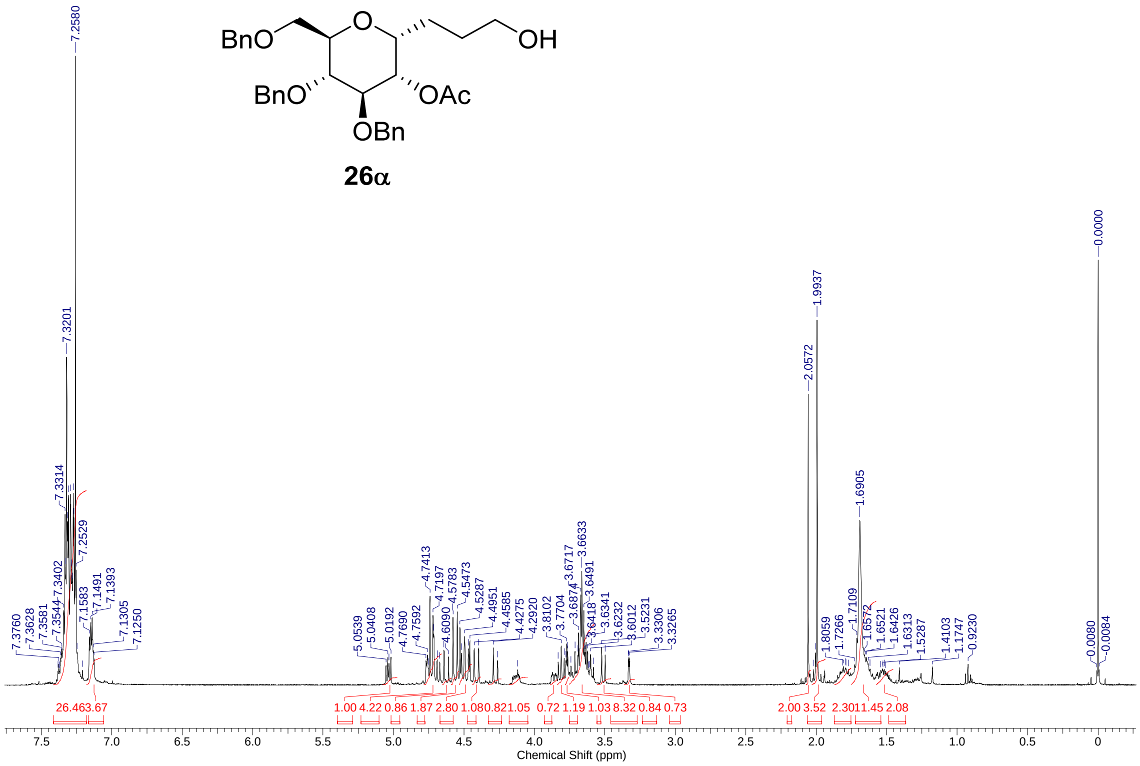

26α

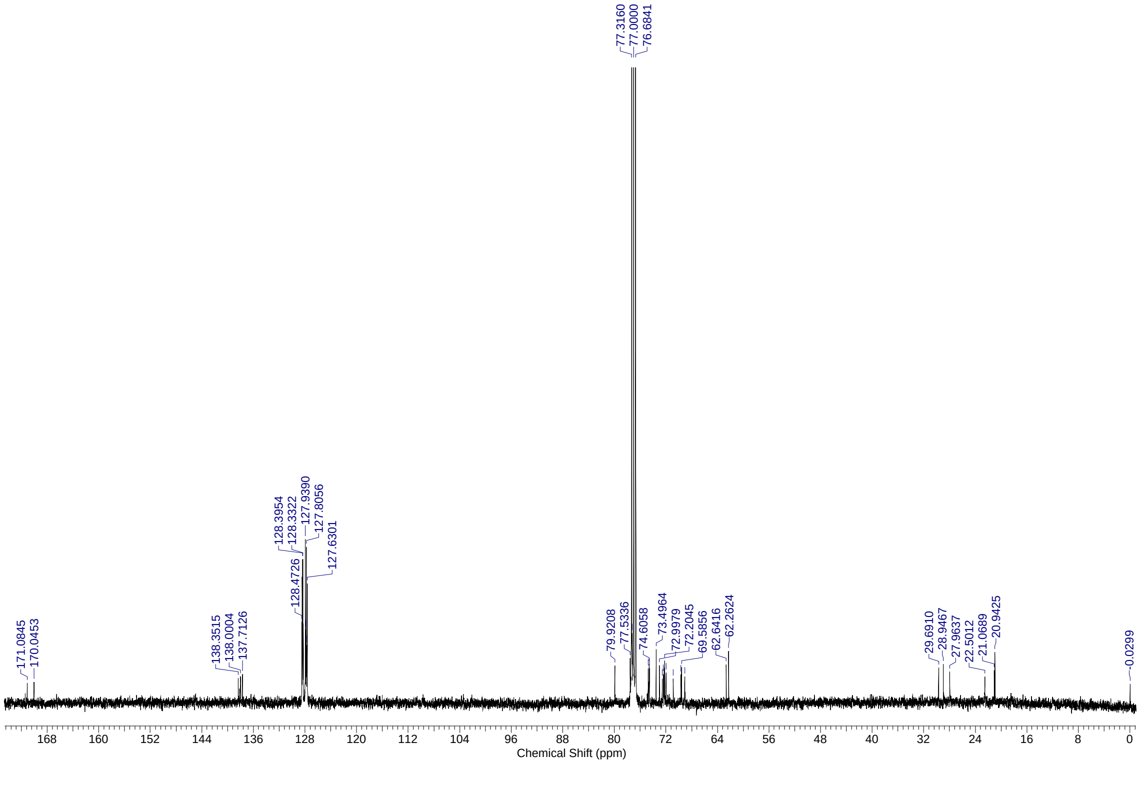

Fig. S30. ^{1}H NMR (400 MHz, $CDCl_3$) and $^{13}C\{H\}$ NMR (100.6 MHz, $CDCl_3$) of compound **26α**.

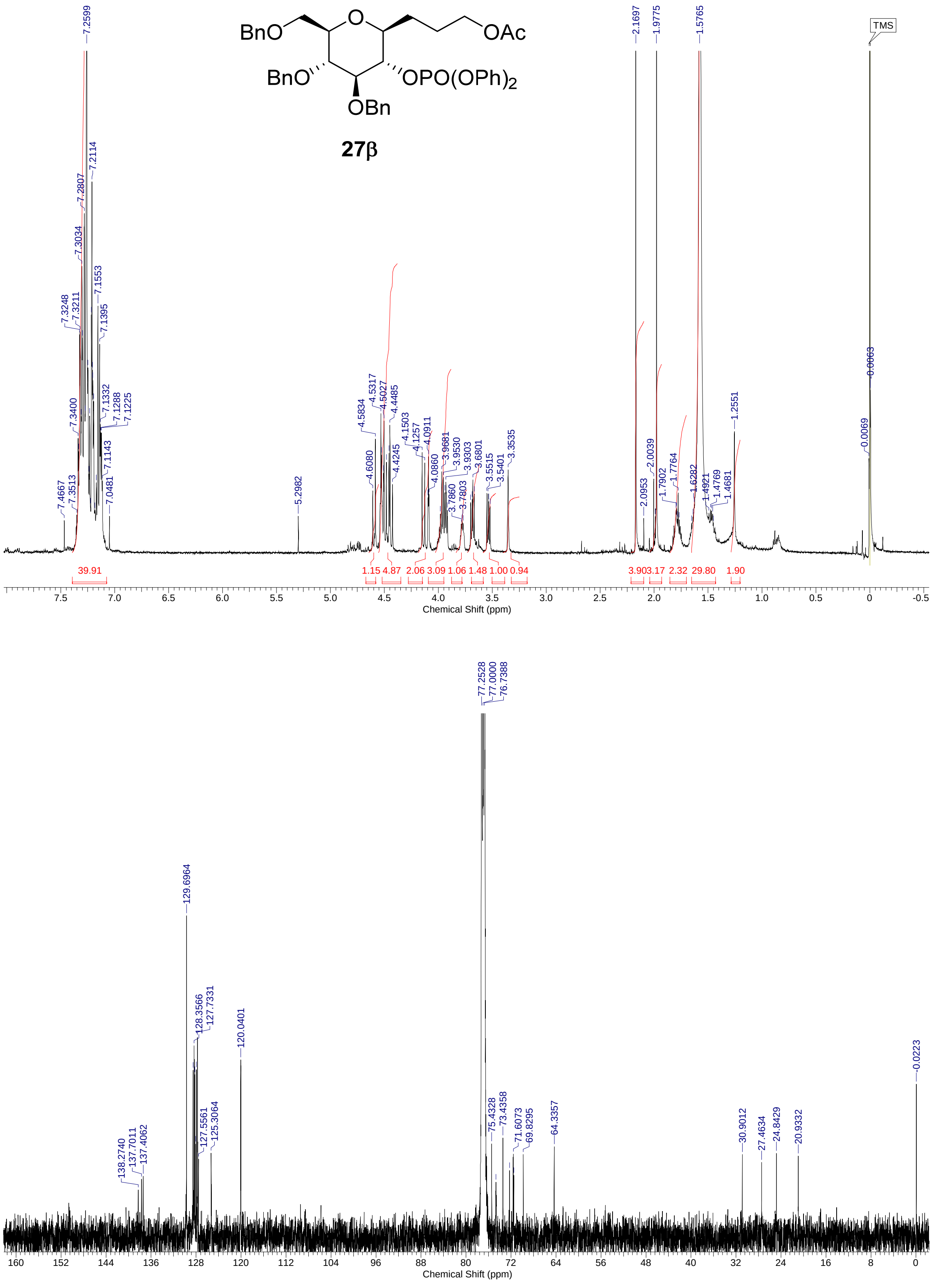

Fig. S31. ^{1}H NMR (500 MHz, $CDCl_3$) and $^{13}C\{H\}$ NMR (125.7 MHz, $CDCl_3$) of compound **27β**.

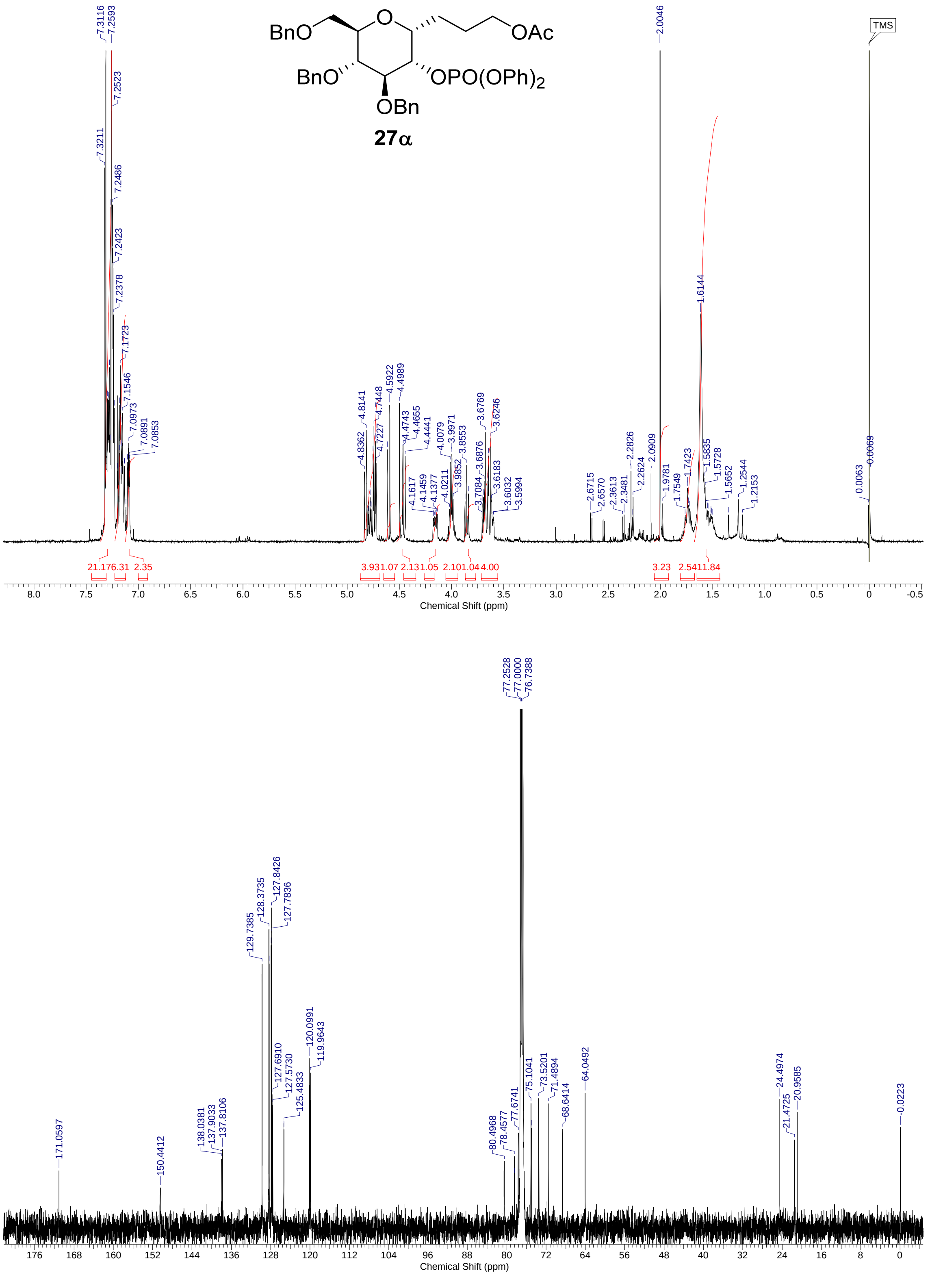

Fig. S32. ^{1}H NMR (500 MHz, $CDCl_3$) and ^{13}C{H} NMR (125.7 MHz, $CDCl_3$) of compound **27α**.

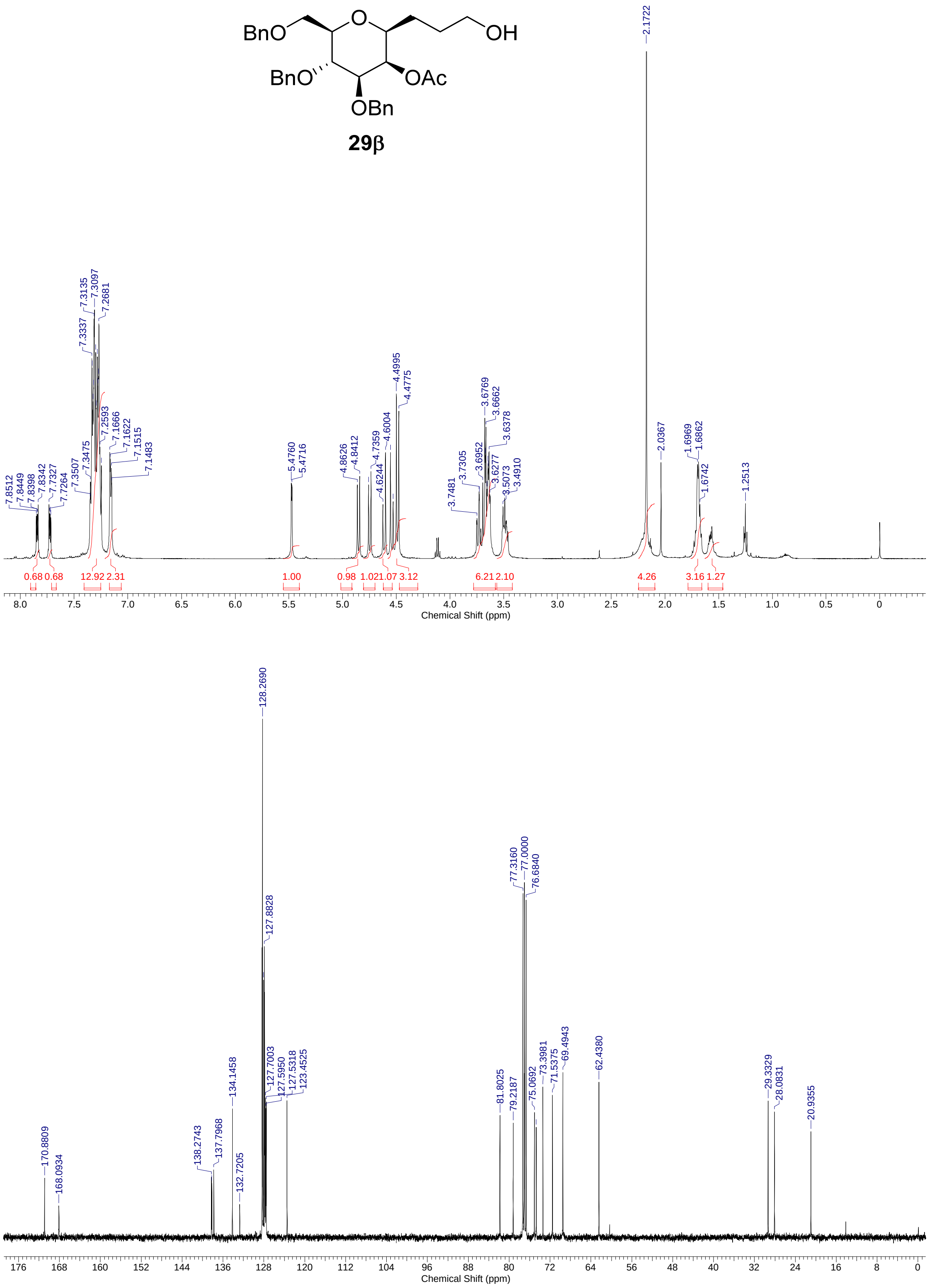

Fig. S33. ^{1}H NMR (500 MHz, $CDCl_3$) and ^{13}C{H} NMR (100.6 MHz, $CDCl_3$) of compound **29β**.

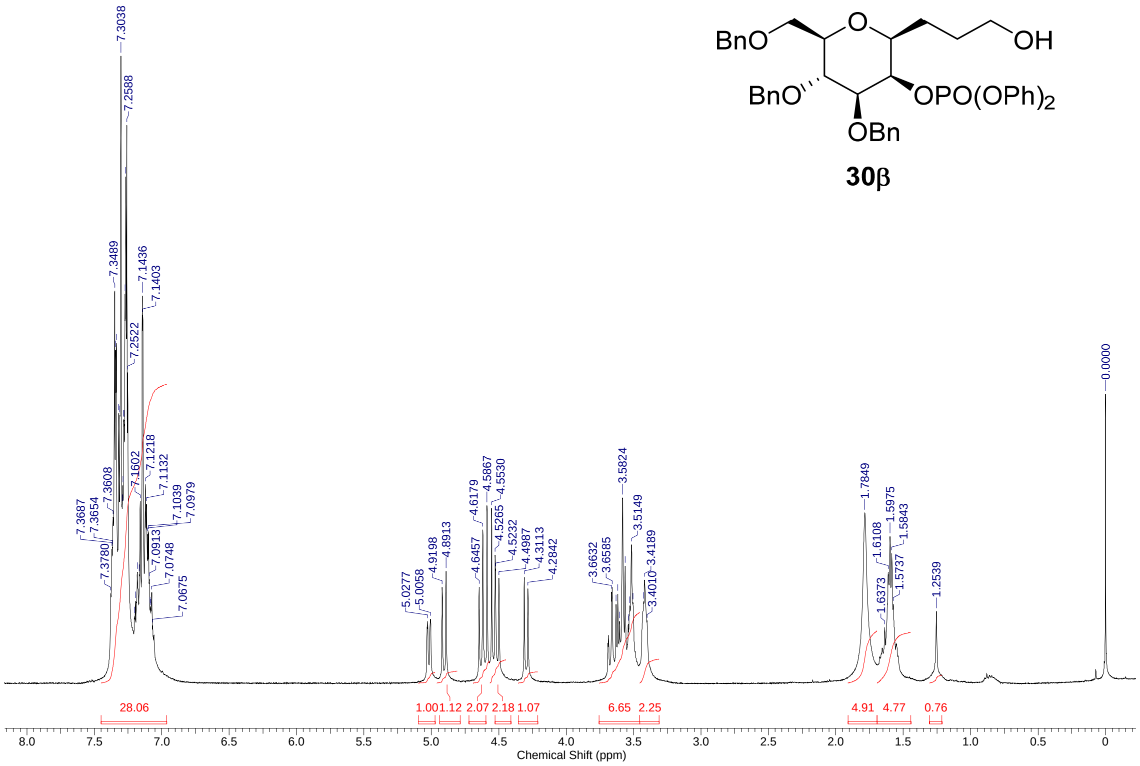

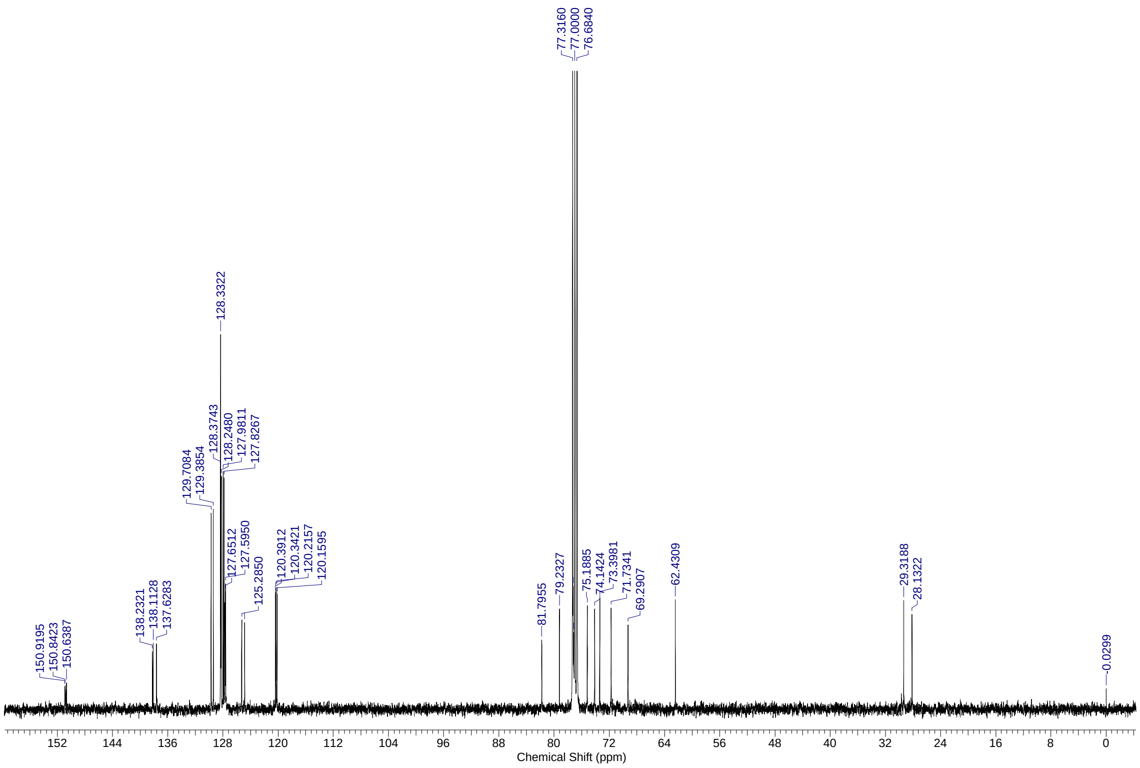

Fig. S34. ^{1}H NMR (400 MHz, $CDCl_3$) and ^{13}C{H} NMR (100.6 MHz, $CDCl_3$) of compound **30β**.

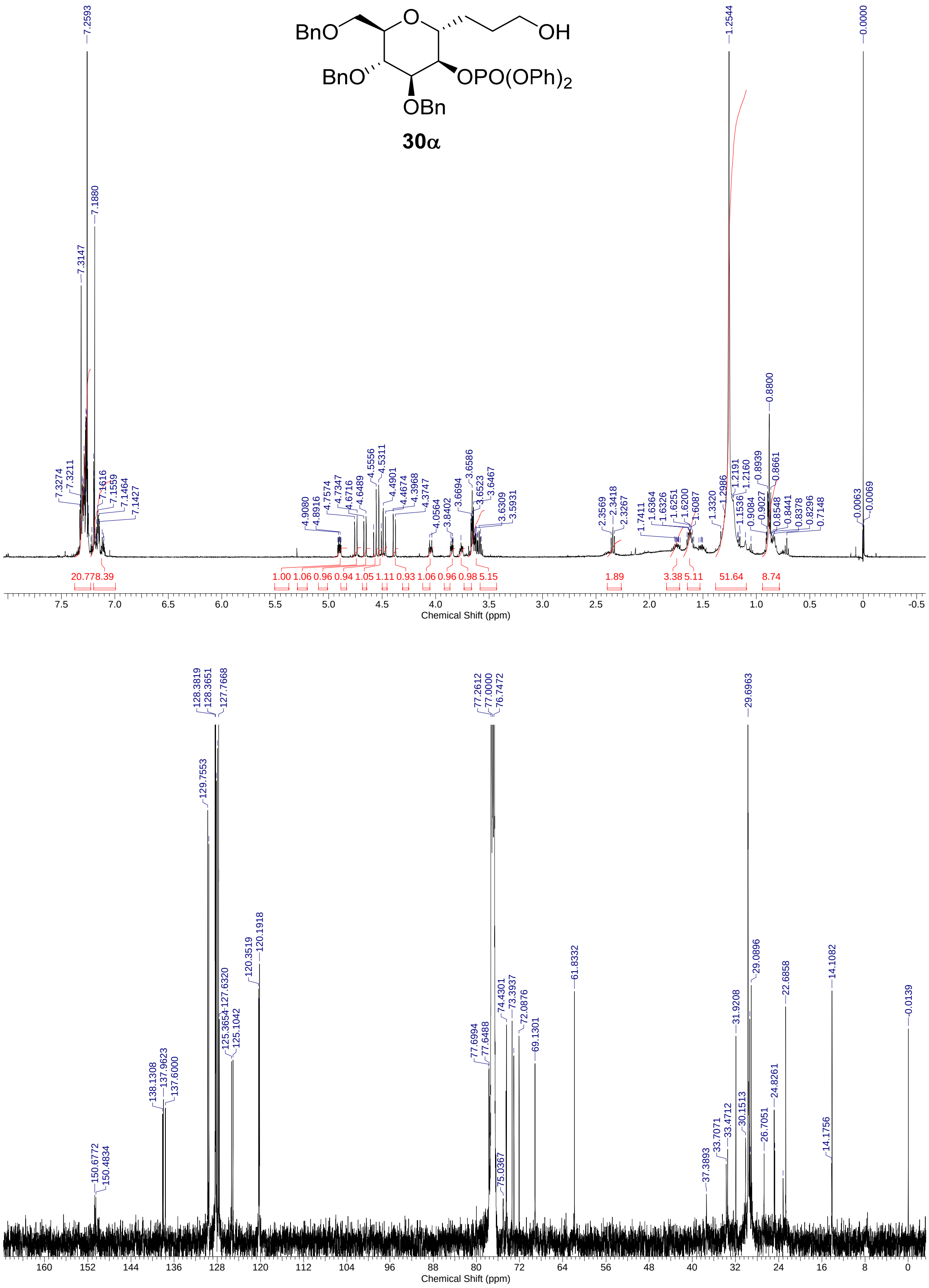

Fig. S35. ^{1}H NMR (500 MHz, $CDCl_3$) and $^{13}C\{H\}$ NMR (125.7 MHz, $CDCl_3$) of compound **30α**.

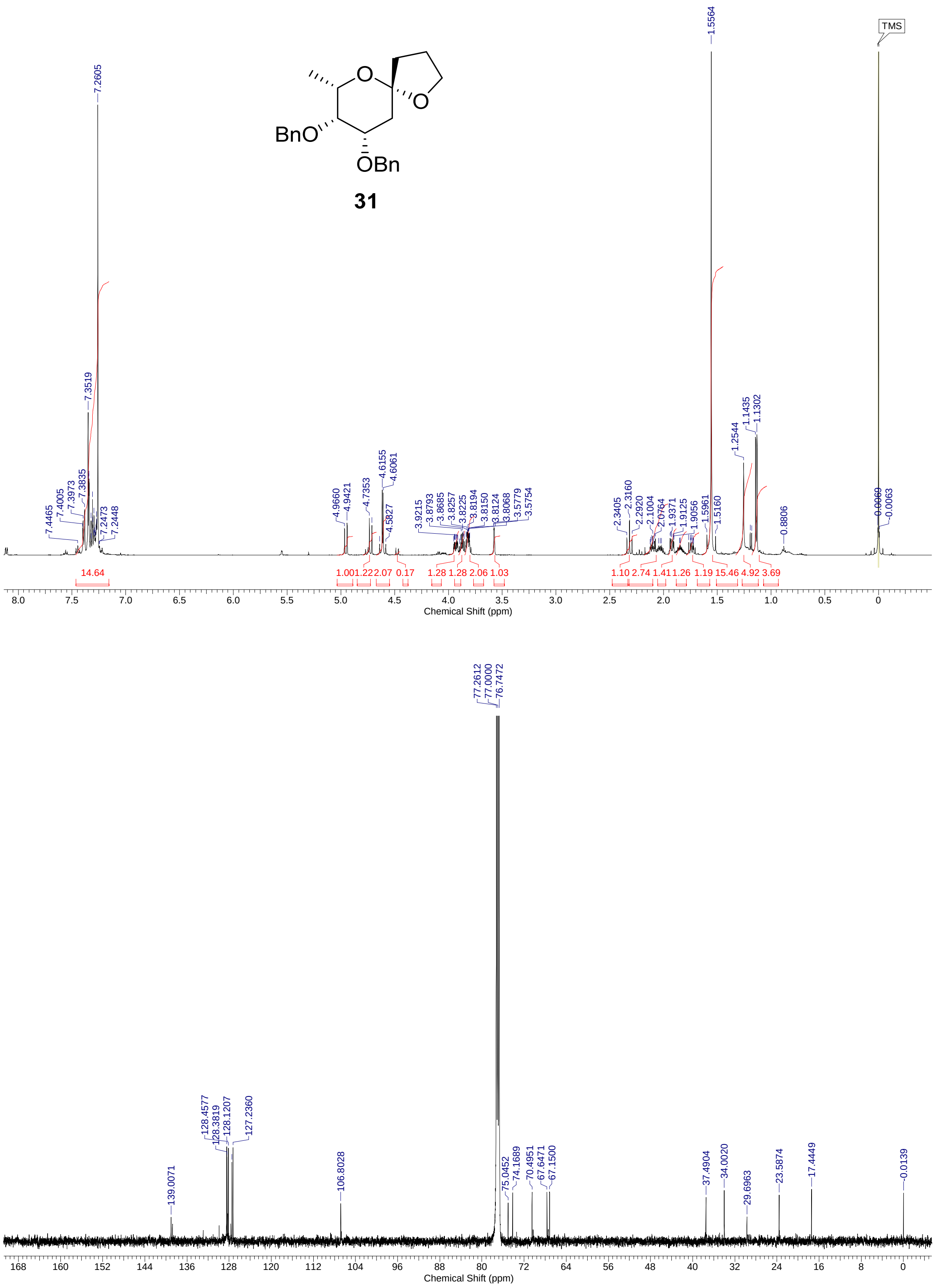

Fig. S36. ^{1}H NMR (500 MHz, $CDCl_3$) and $^{13}C\{H\}$ NMR (125.7 MHz, $CDCl_3$) of compound **31**.

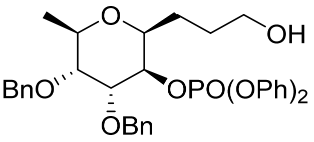

34

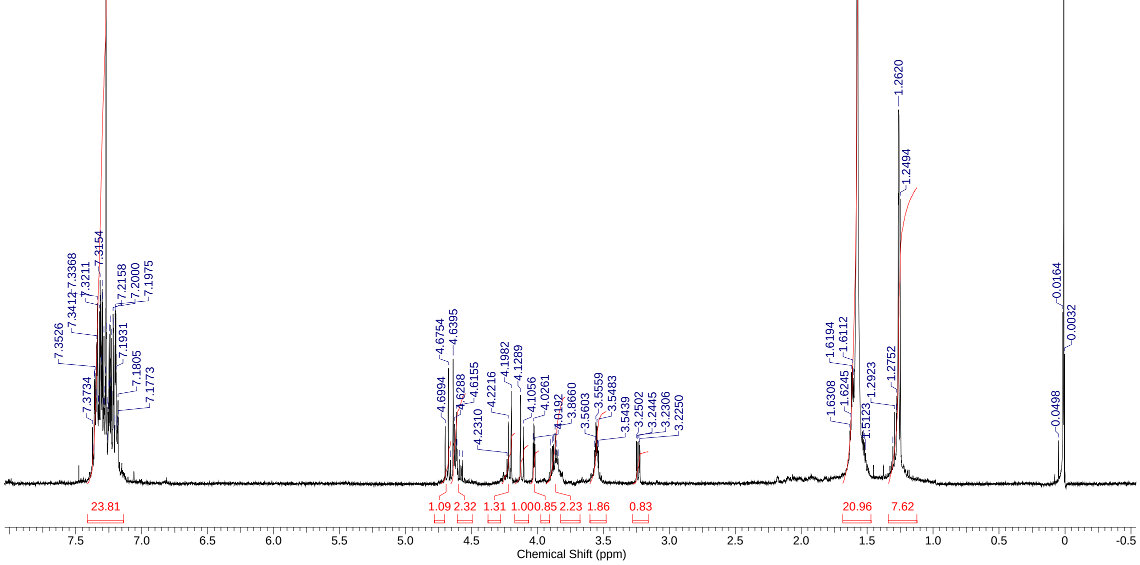

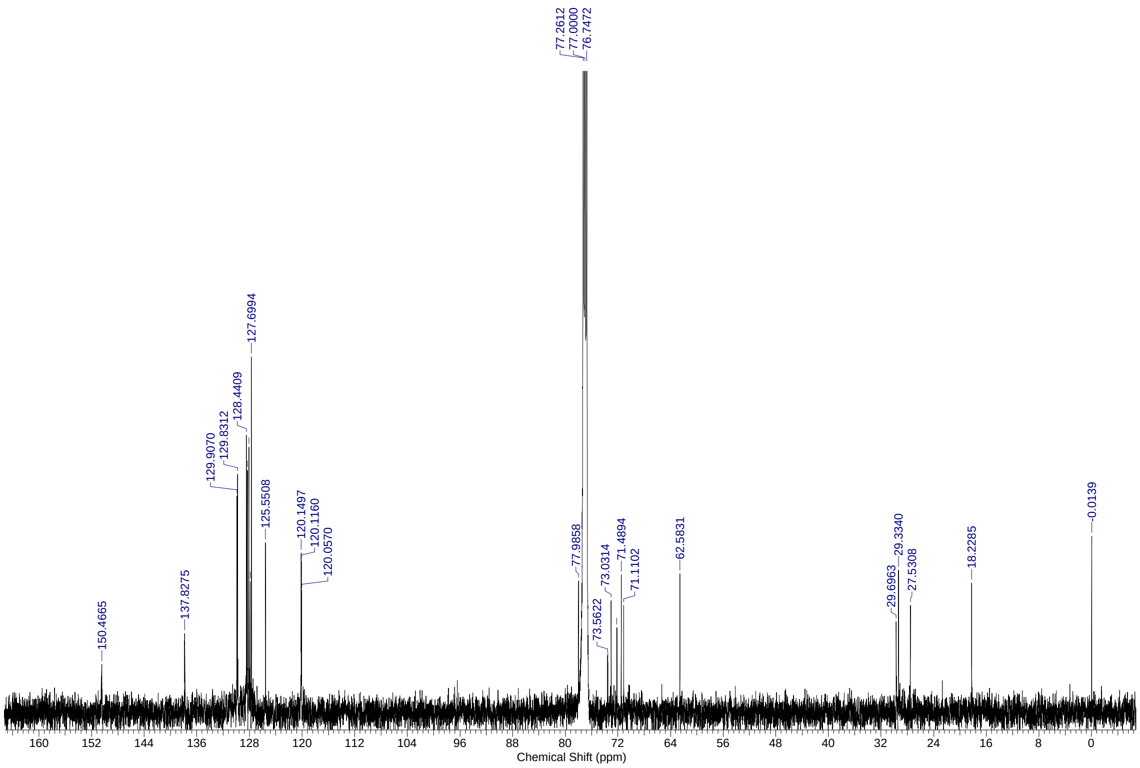

Fig. S37. ^{1}H NMR (500 MHz, $CDCl_3$) and ^{13}C{H} NMR (125.7 MHz, $CDCl_3$) of compound **34**.

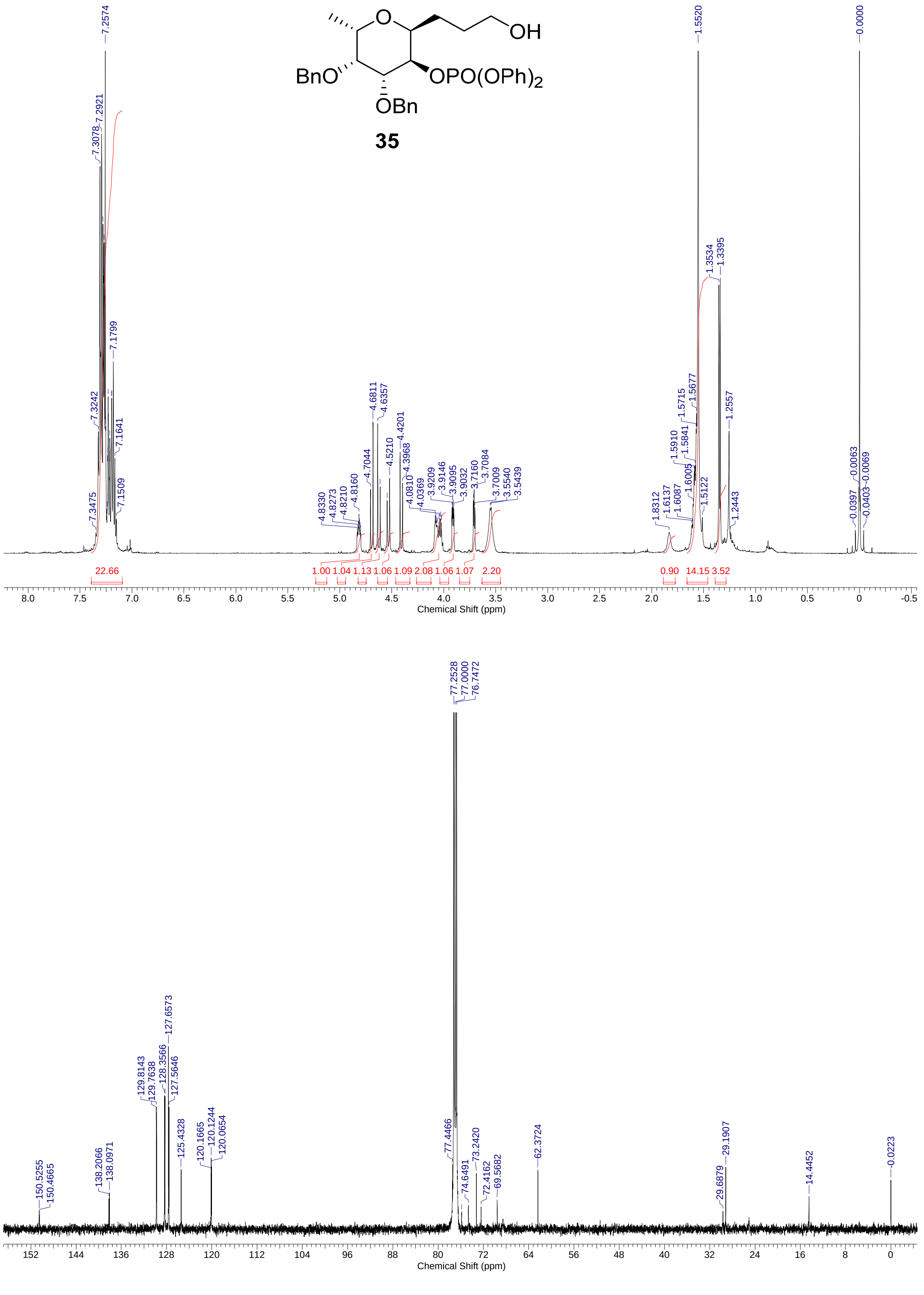

Fig. S38. ^{1}H NMR (500 MHz, $CDCl_3$) and $^{13}C\{H\}$ NMR (125.7 MHz, $CDCl_3$) of compound **35**.

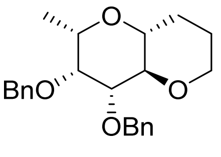

36

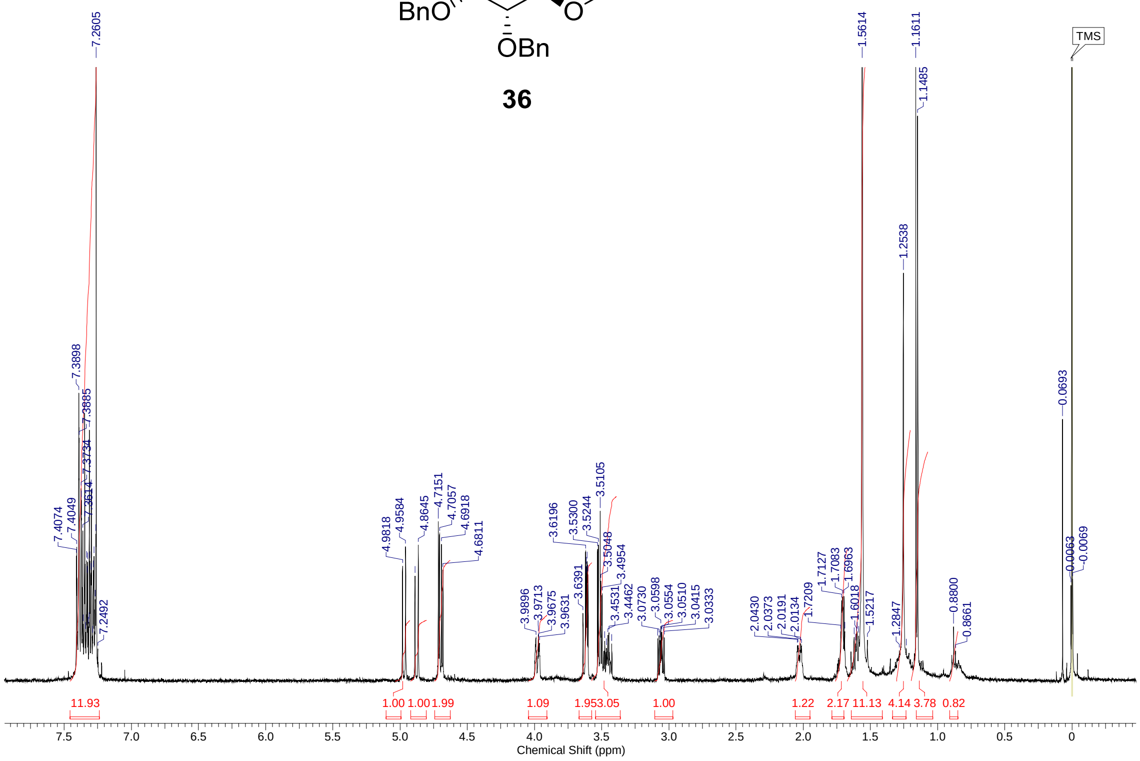

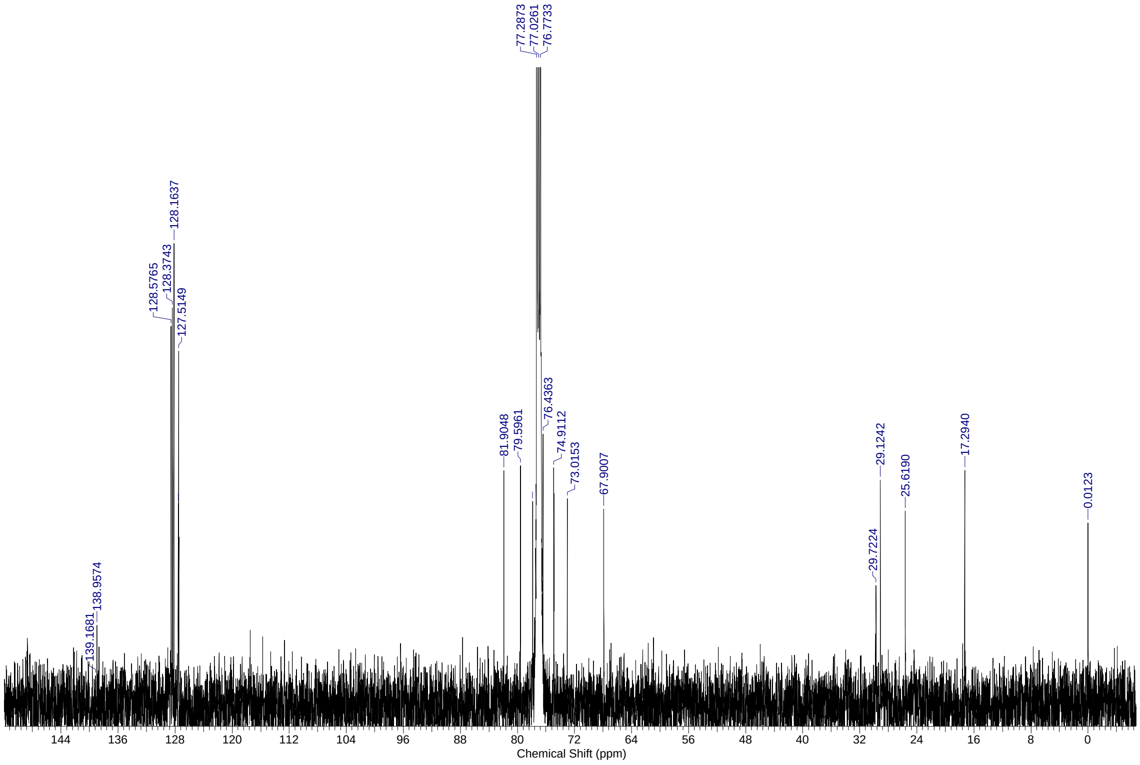

Fig. S39. ^{1}H NMR (500 MHz, $CDCl_3$) and ^{13}C{H} NMR (125.7 MHz, $CDCl_3$) of compound **36**.

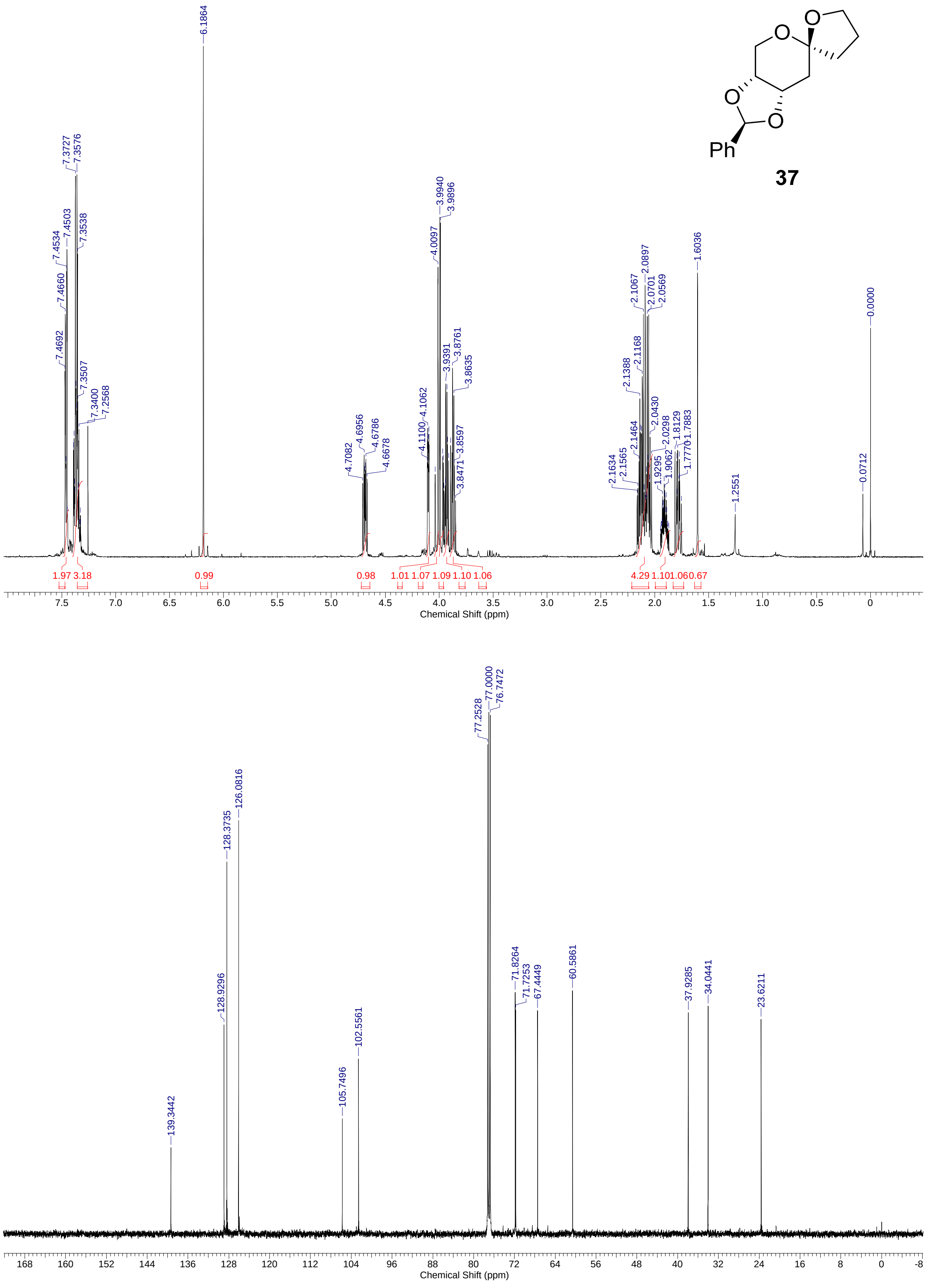

Fig. S40. ^{1}H NMR (500 MHz, $CDCl_3$) and $^{13}C\{H\}$ NMR (125.7 MHz, $CDCl_3$) of compound **37**.

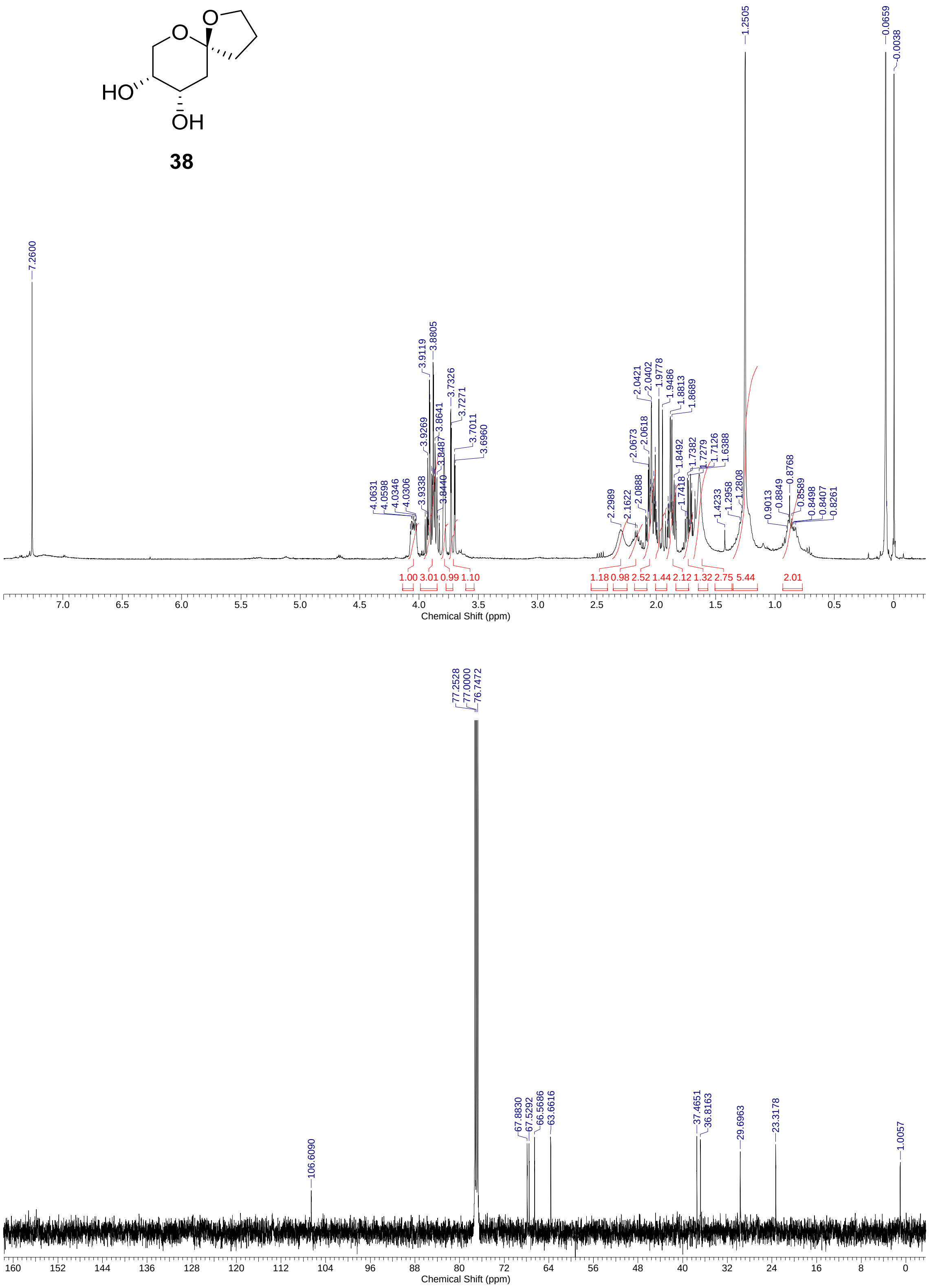

Fig. S41. ^{1}H NMR (400 MHz, $CDCl_3$) and ^{13}C{H} NMR (125.7 MHz, $CDCl_3$) of compound **38**.

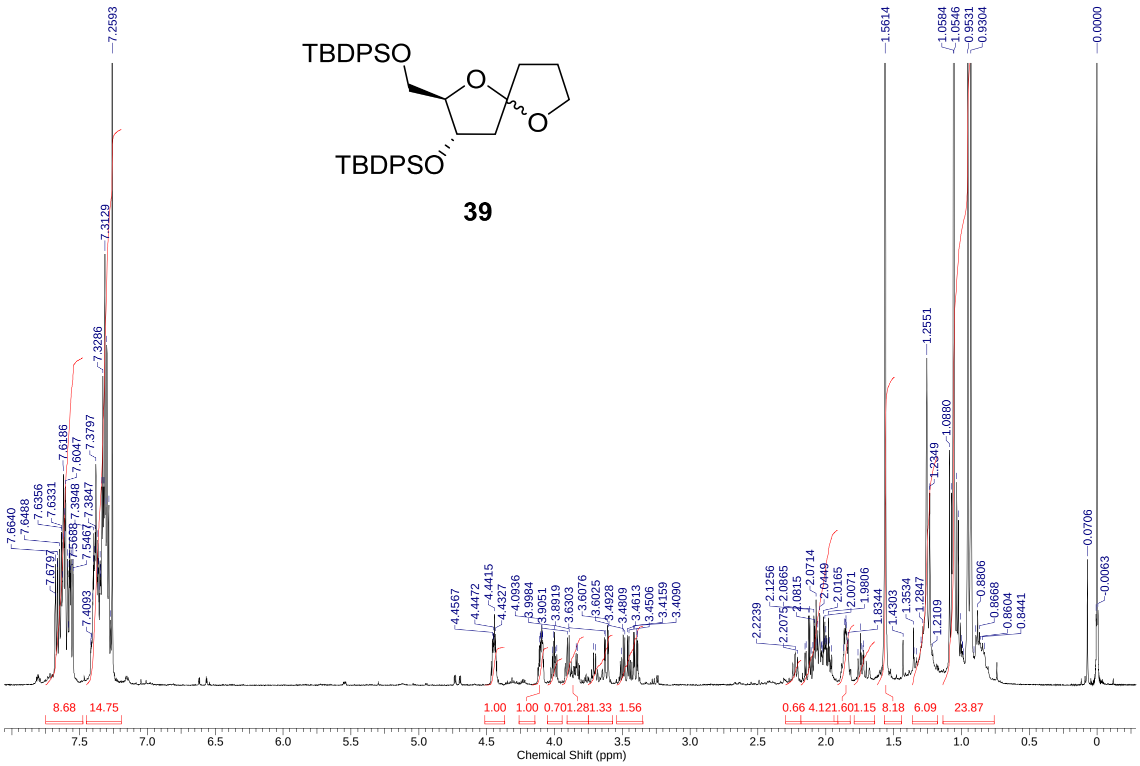

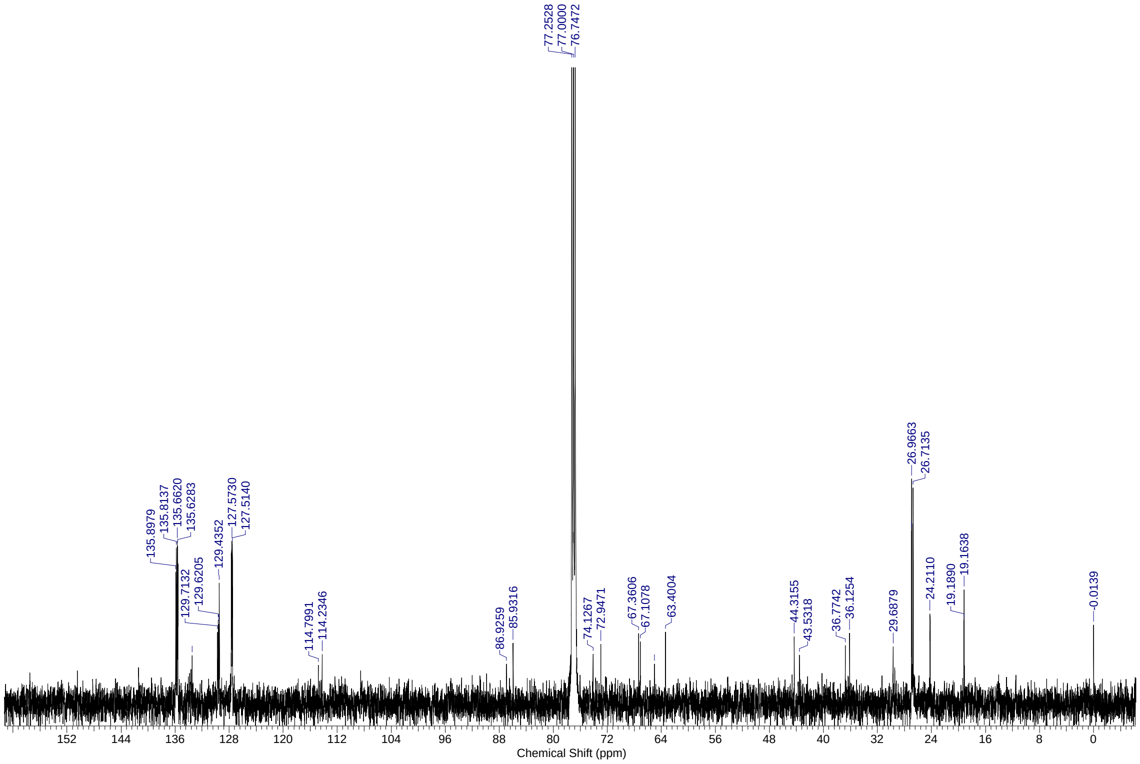

Fig. S42. ^{1}H NMR (500 MHz, $CDCl_3$) and $^{13}C\{H\}$ NMR (125.7 MHz, $CDCl_3$) of compound **39**.

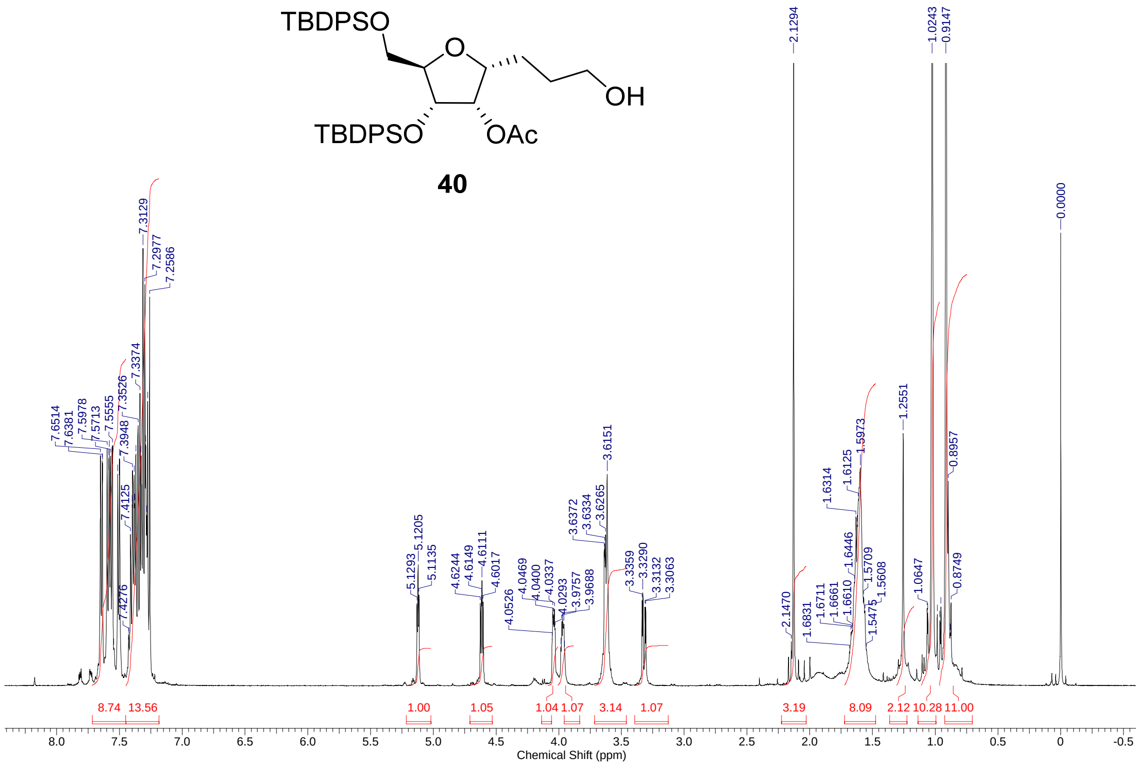

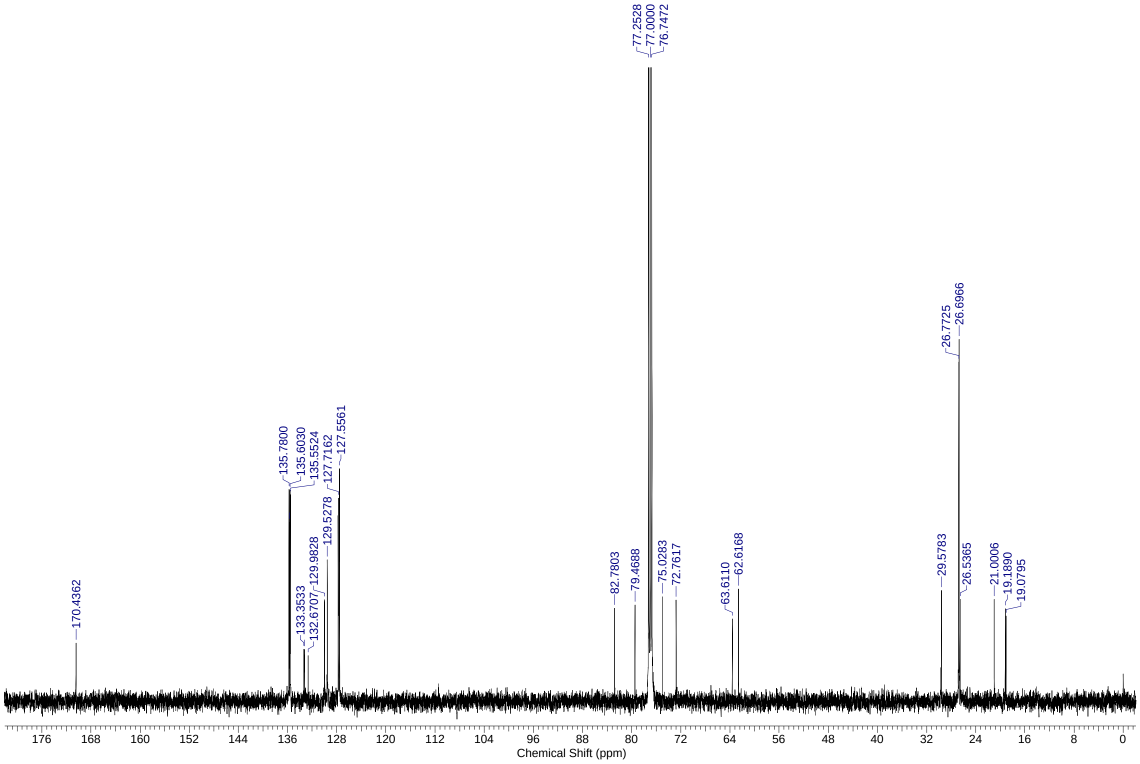

Fig. S43. ^{1}H NMR (500 MHz, $CDCl_3$) and ^{13}C{H} NMR (125.7 MHz, $CDCl_3$) of compound **40**.

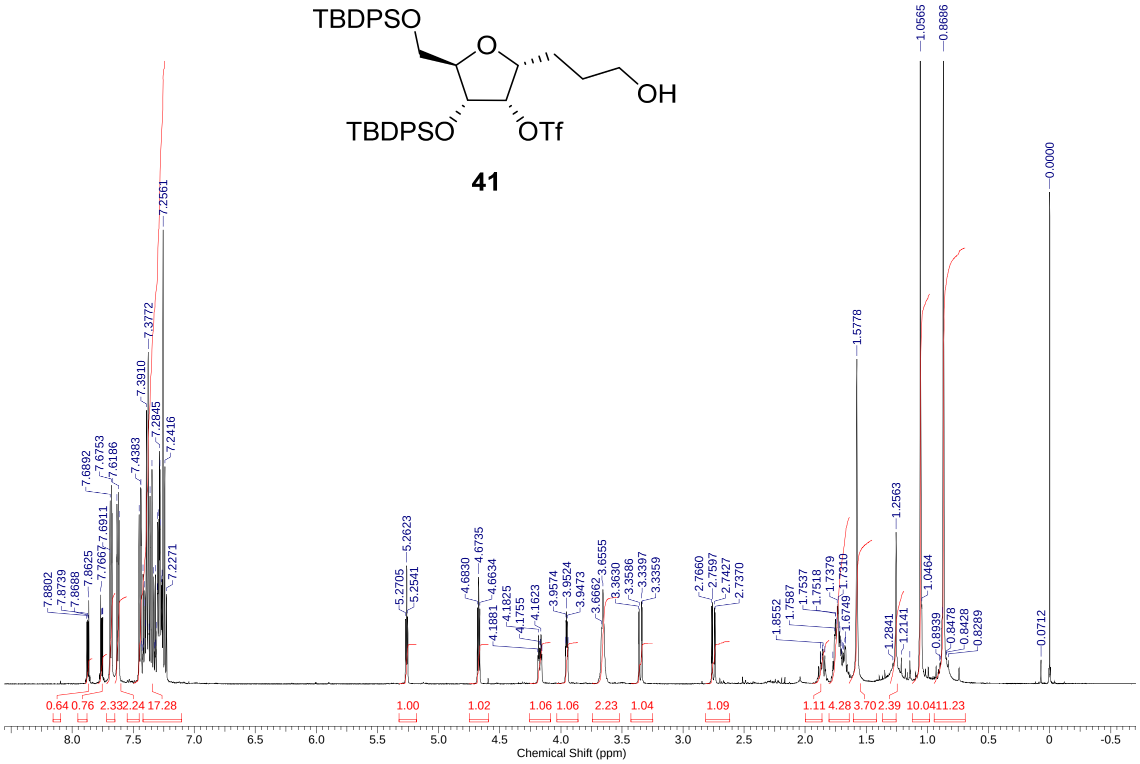

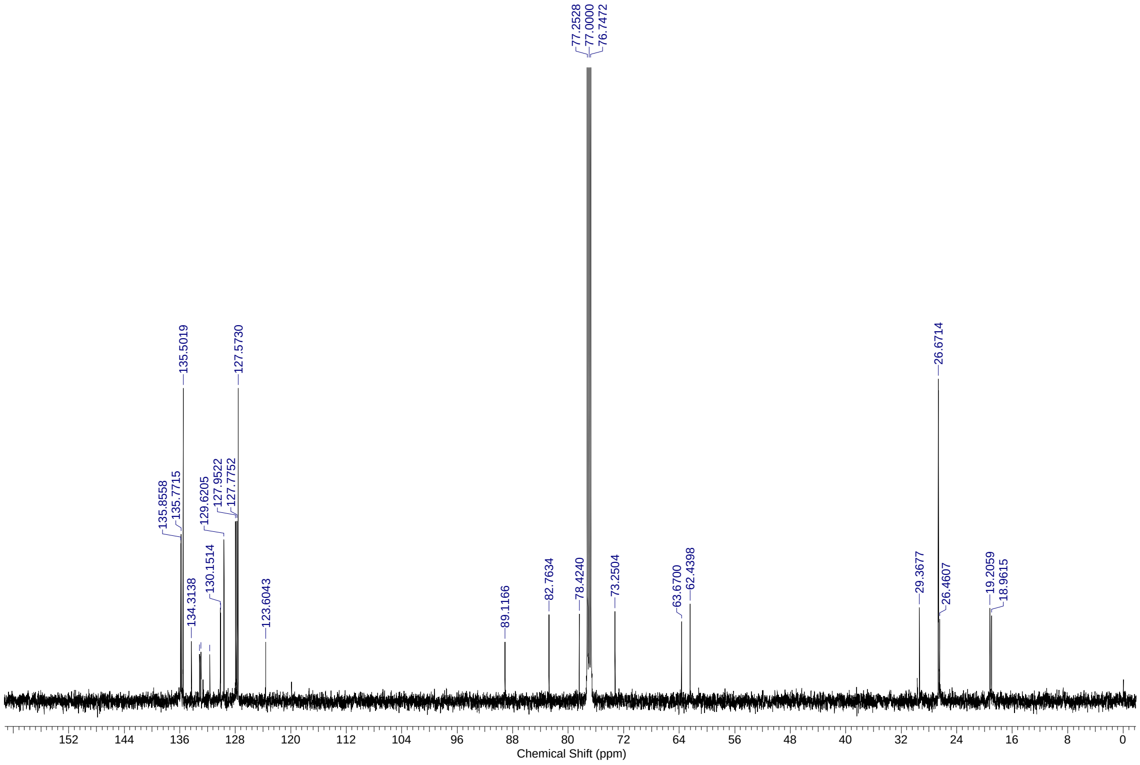

Fig. S44. ^{1}H NMR (500 MHz, $CDCl_3$) and ^{13}C{H} NMR (125.7 MHz, $CDCl_3$) of compound **41**.

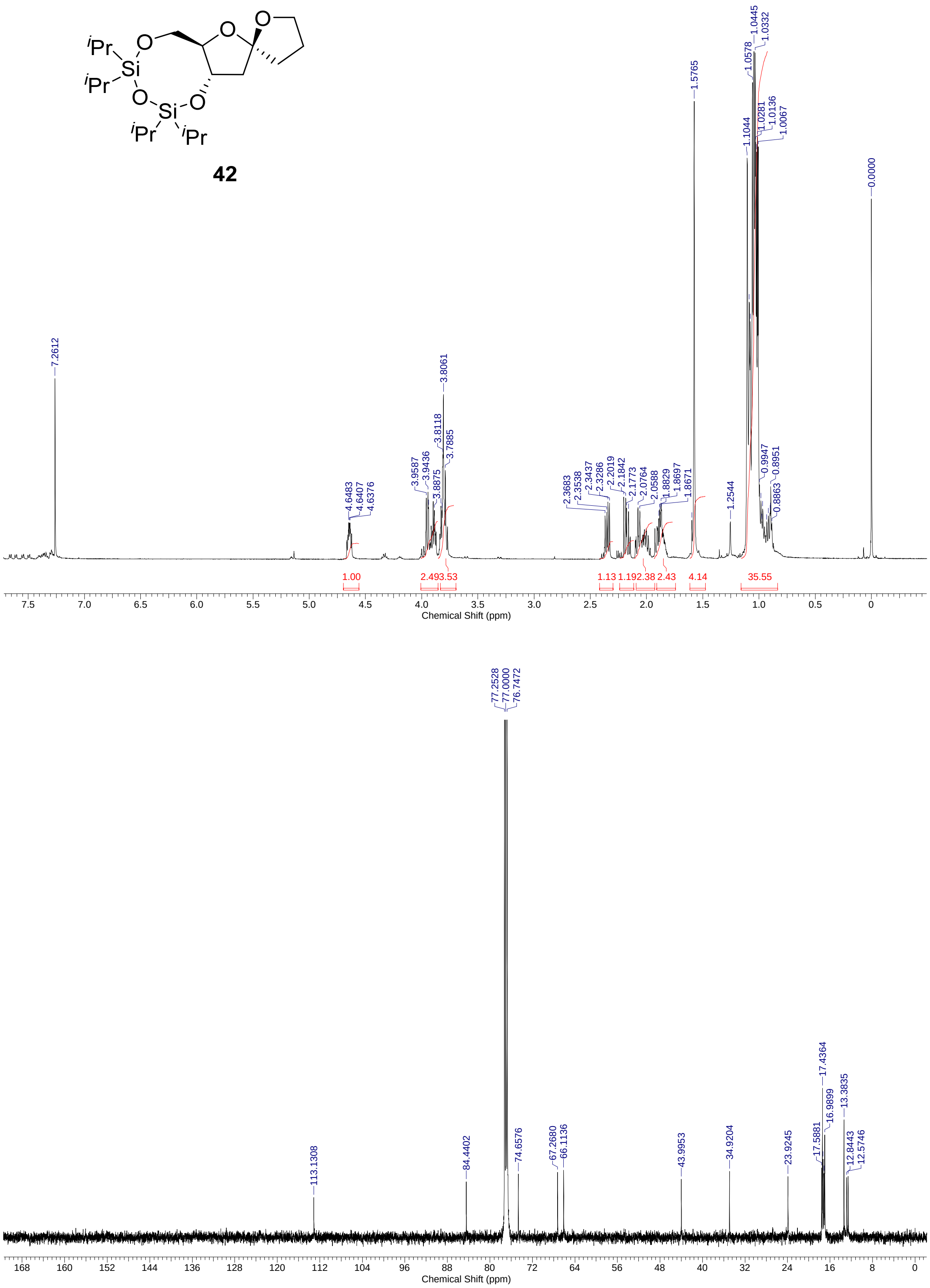

Fig. S45. ^{1}H NMR (500 MHz, $CDCl_3$) and $^{13}C\{H\}$ NMR (125.7 MHz, $CDCl_3$) of compound **42**.

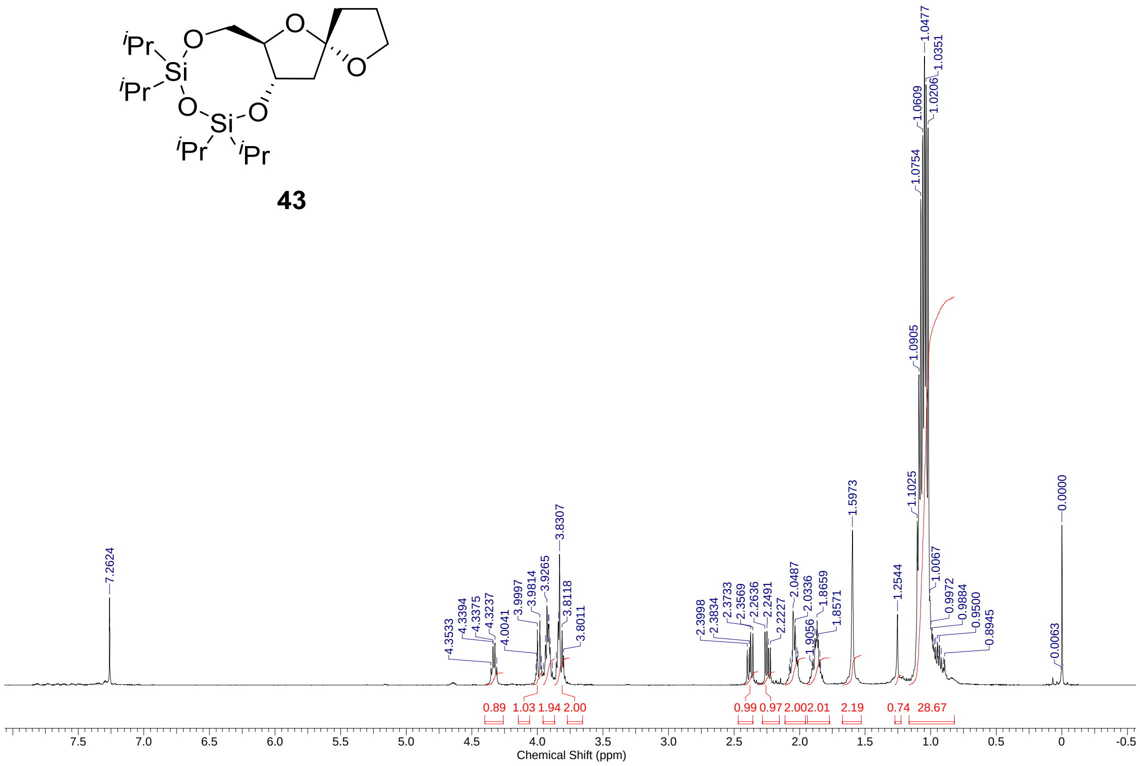

43

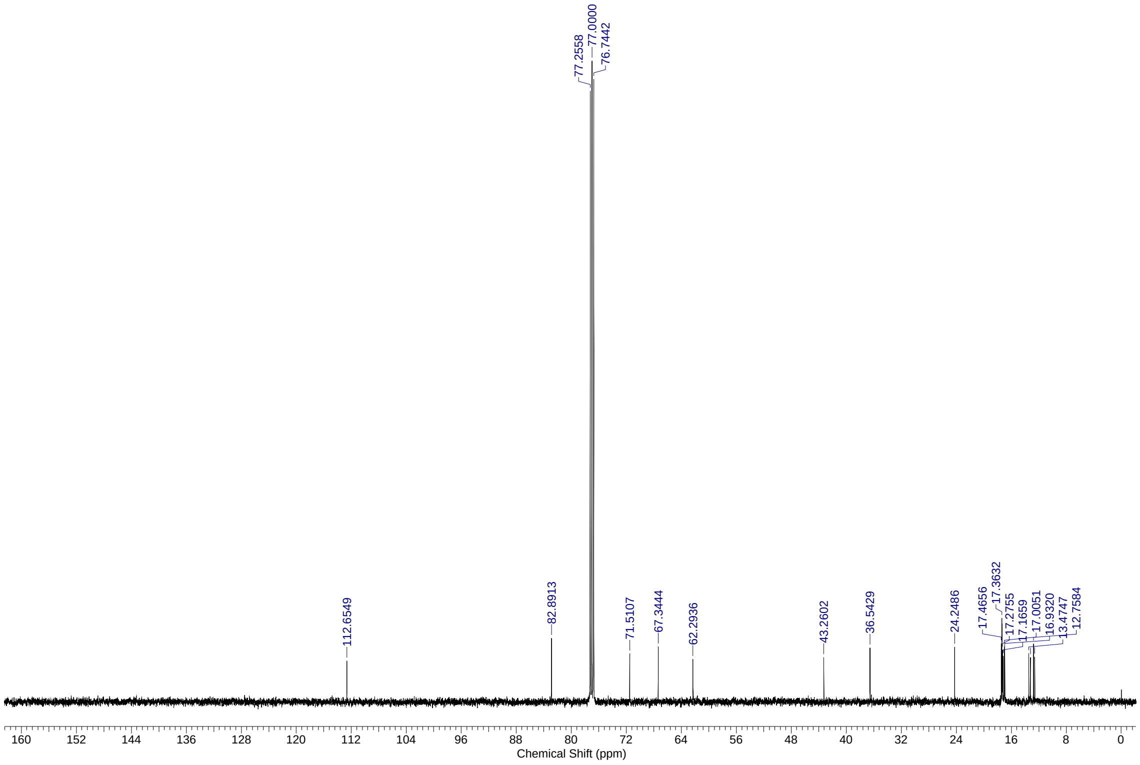

Fig. S46. ^{1}H NMR (500 MHz, $CDCl_3$) and ^{13}C{H} NMR (125.7 MHz, $CDCl_3$) of compound **43**.

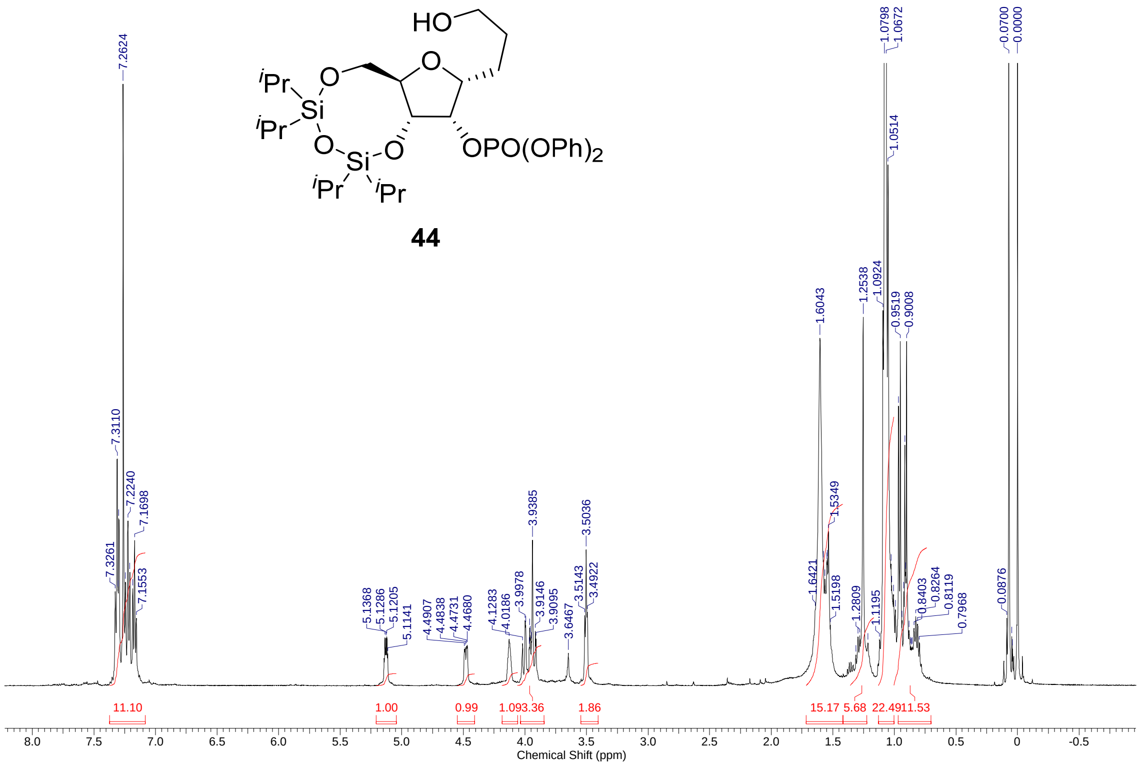

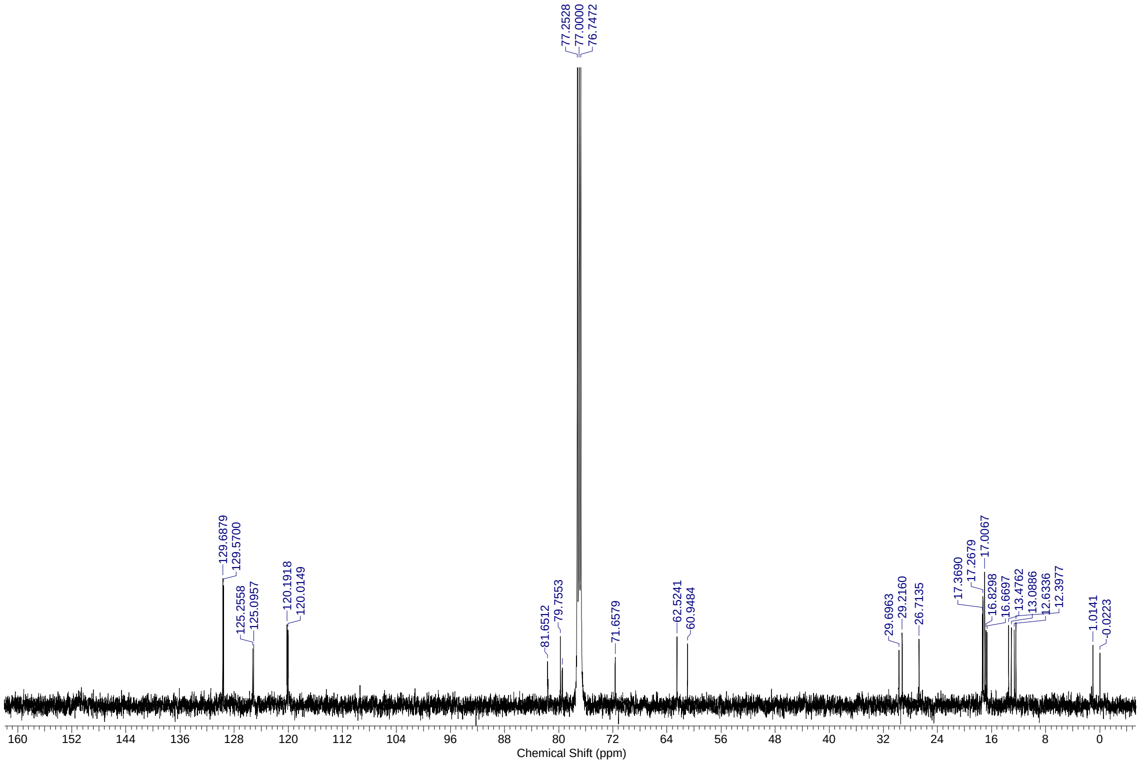

Fig. S47. ^{1}H NMR (500 MHz, $CDCl_3$) and $^{13}C\{H\}$ NMR (125.7 MHz, $CDCl_3$) of compound **44**.

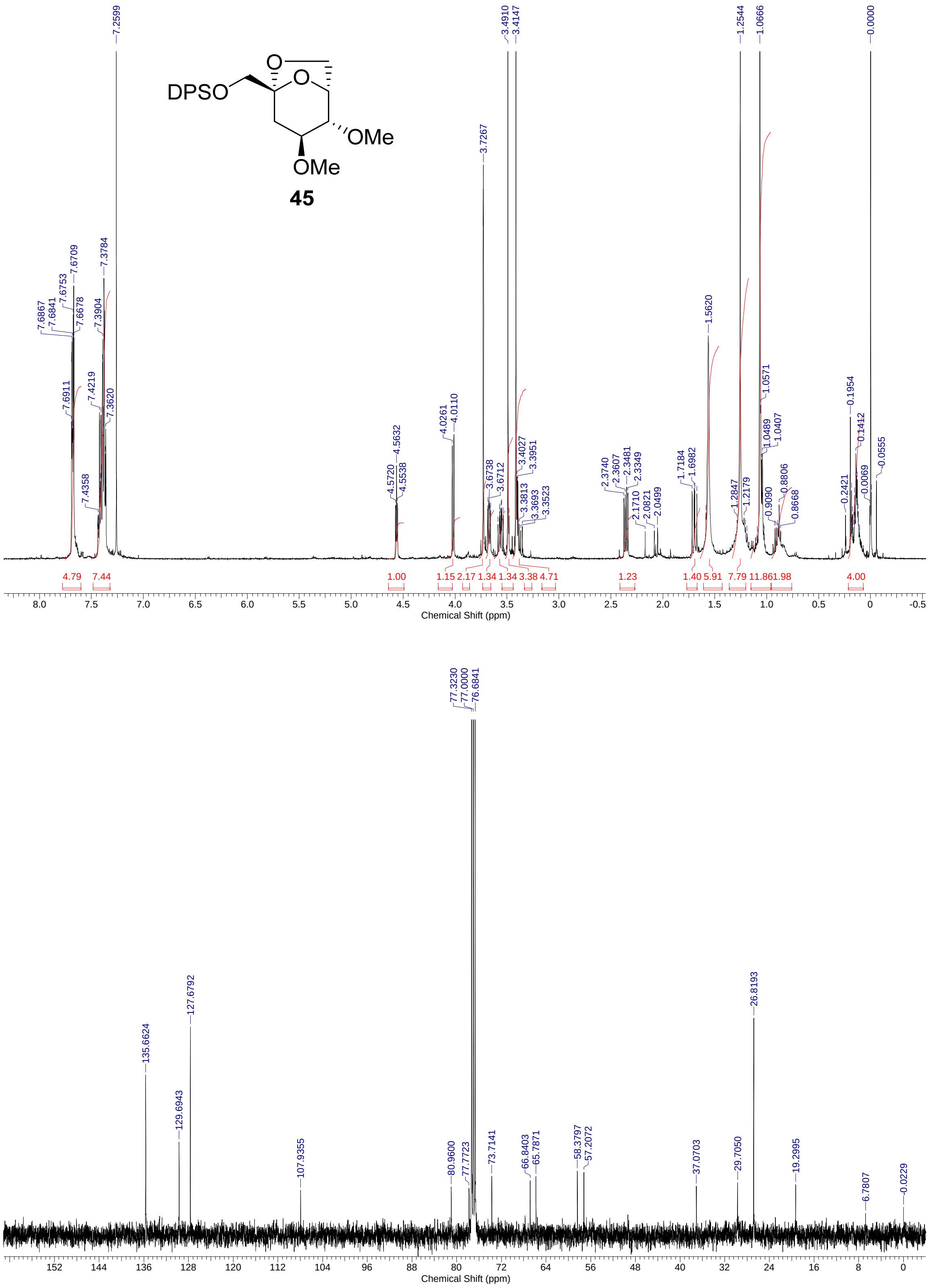

Fig. S48. ^{1}H NMR (500 MHz, $CDCl_3$) and ^{13}C{H} NMR (100.6 MHz, $CDCl_3$) of compound **45**.

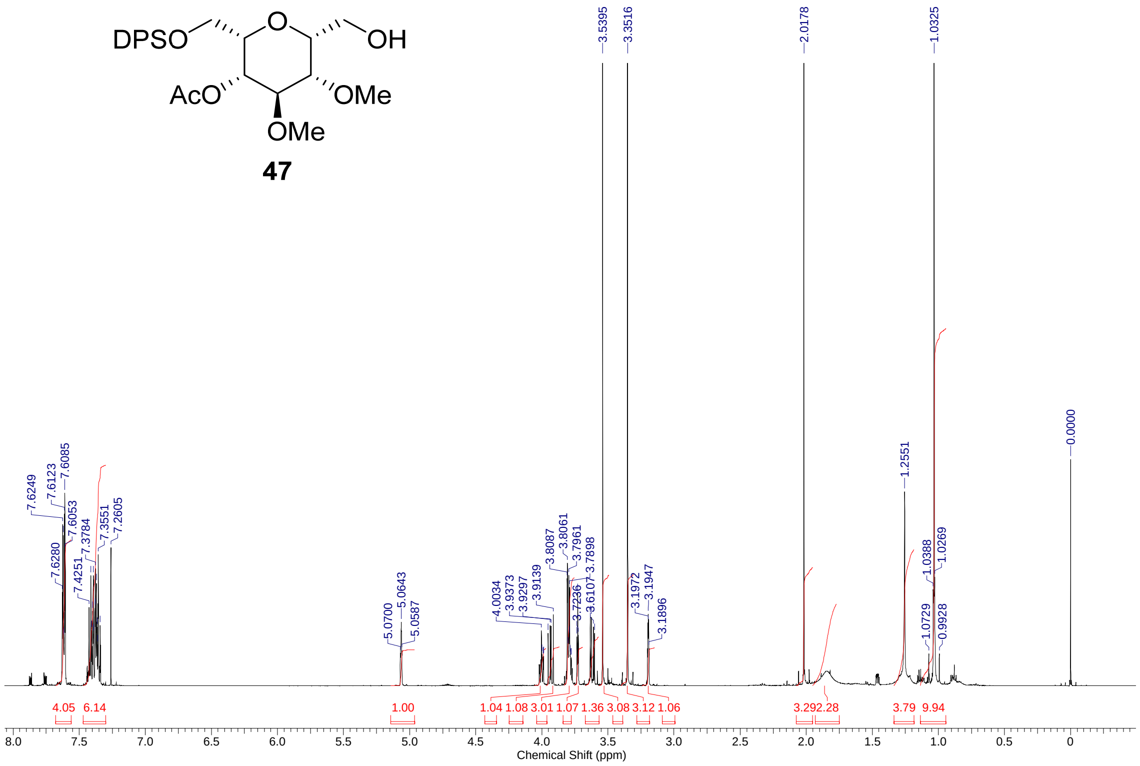

47

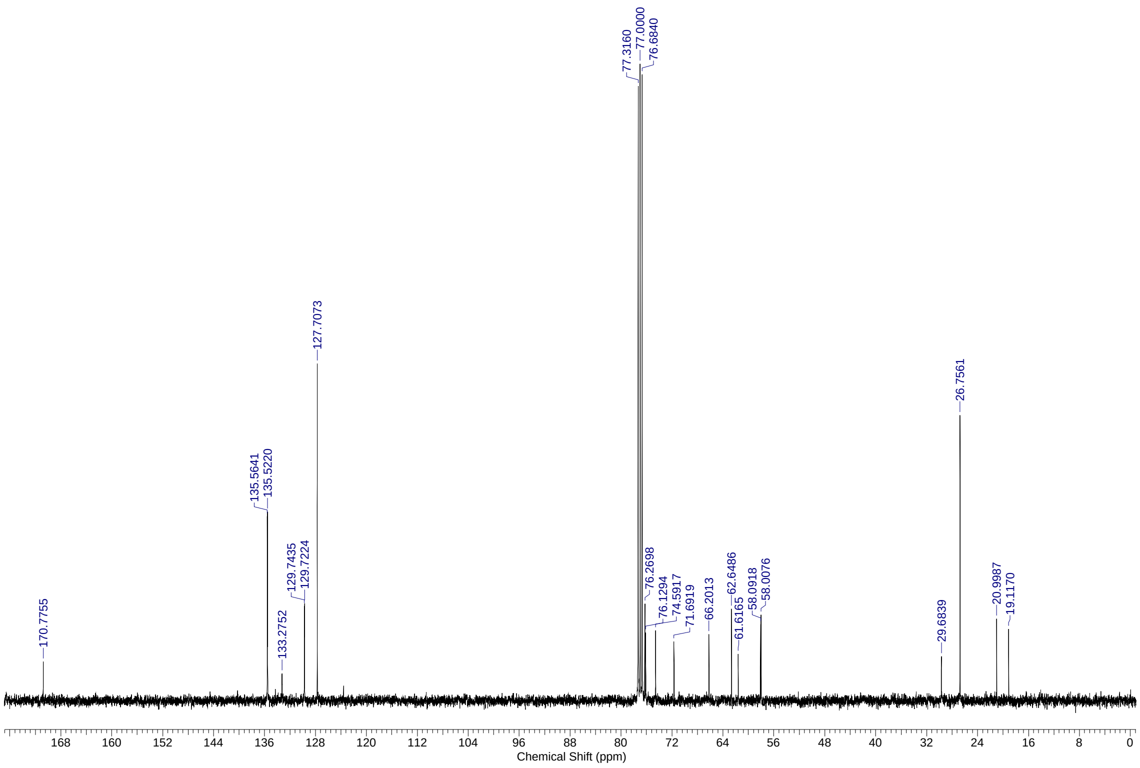

Fig. S49. ^{1}H NMR (500 MHz, $CDCl_3$) and ^{13}C{H} NMR (100.6 MHz, $CDCl_3$) of compound **47**.

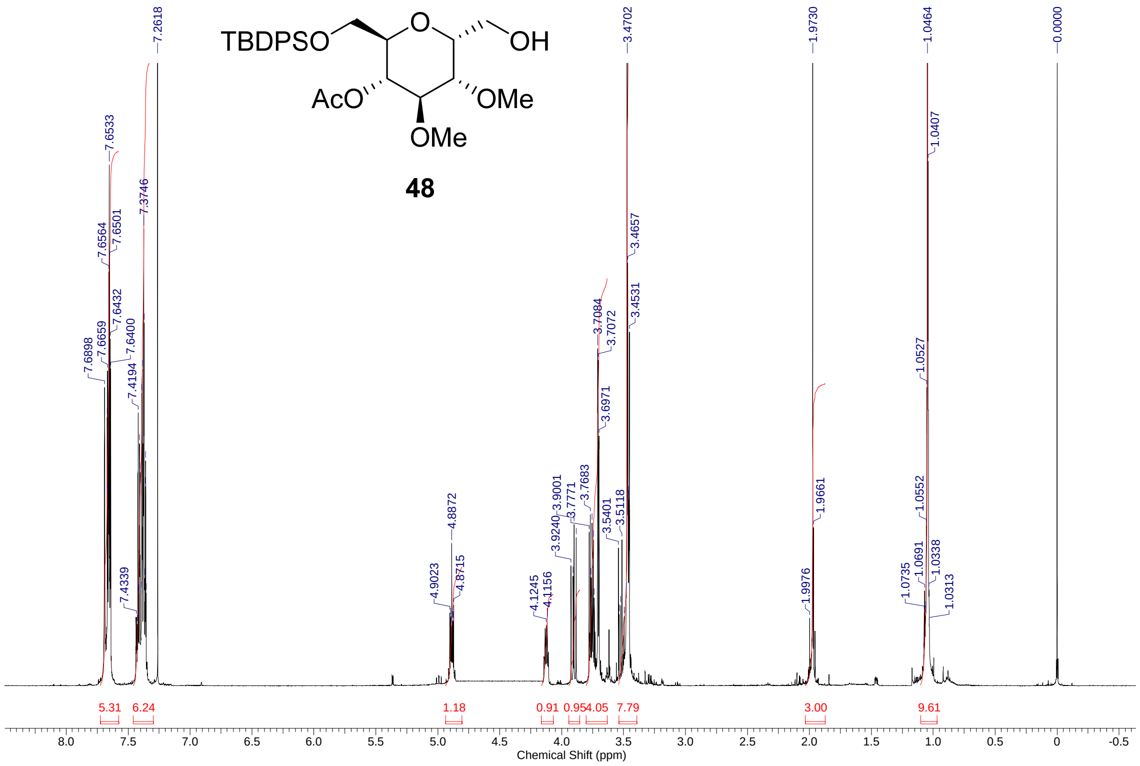

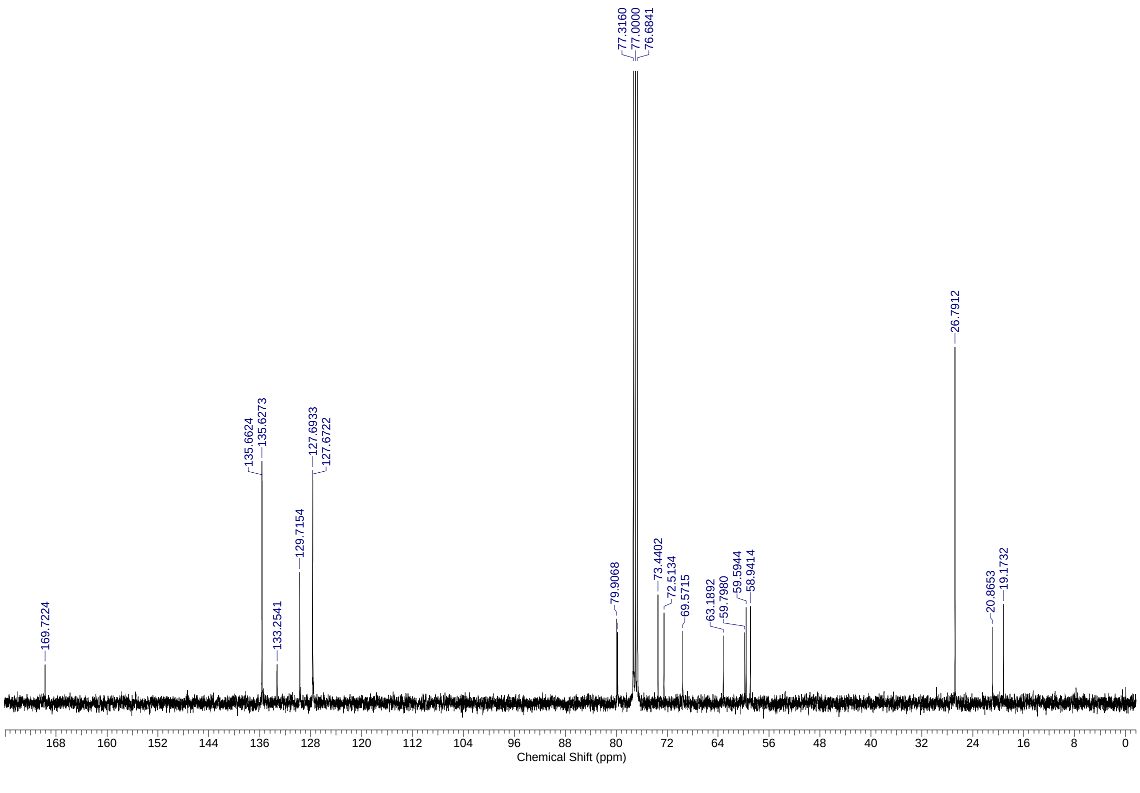

Fig. S50. ^{1}H NMR (500 MHz, $CDCl_3$) and ^{13}C{H} NMR (100.6 MHz, $CDCl_3$) of compound **48**.

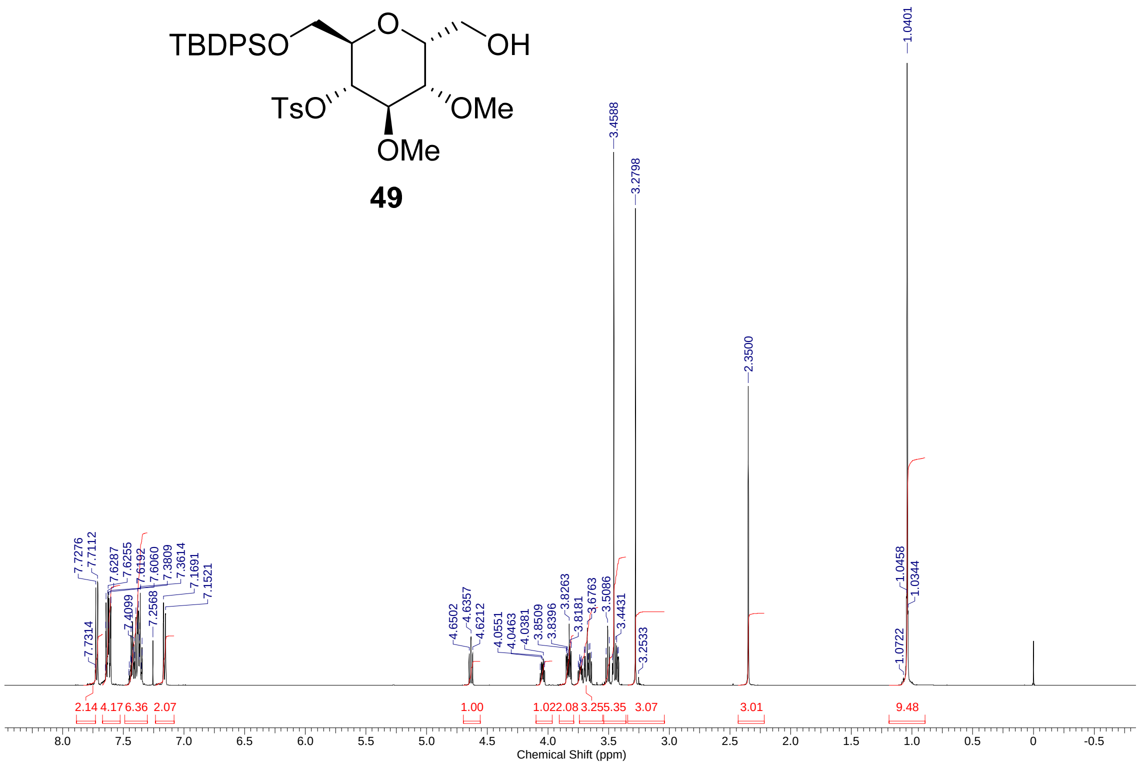

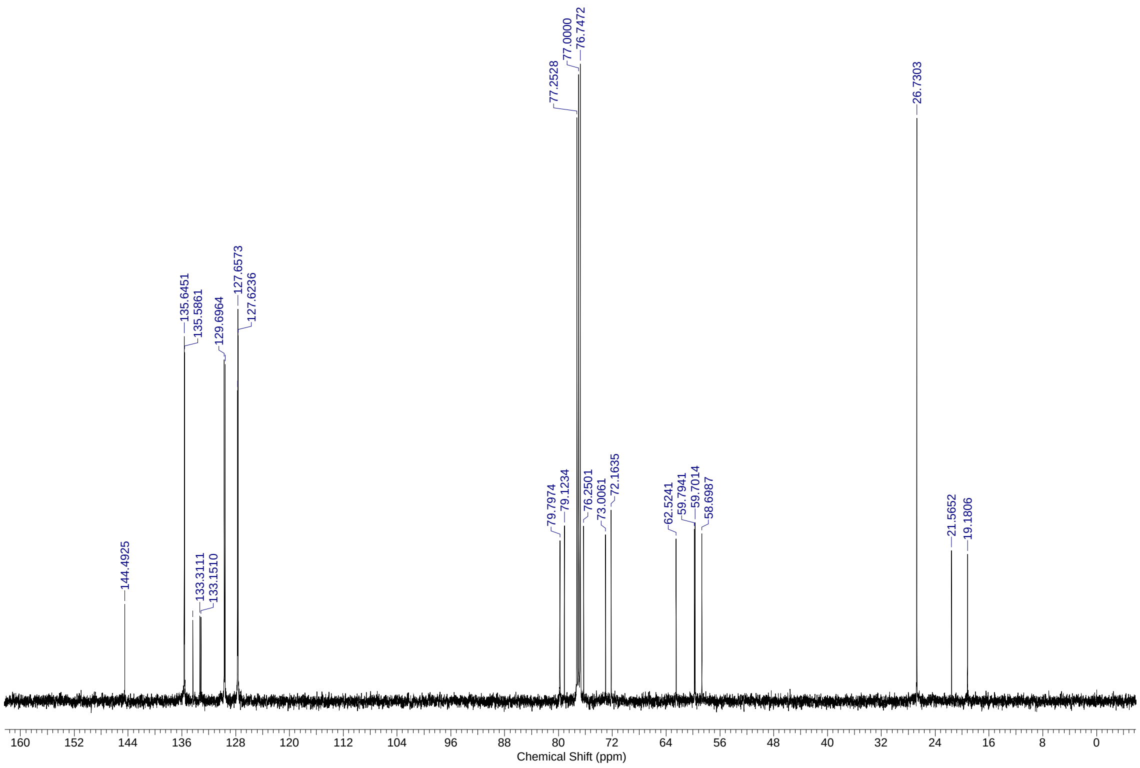

Fig. S51. ^{1}H NMR (500 MHz, $CDCl_3$) and $^{13}C\{H\}$ NMR (100.6 MHz, $CDCl_3$) of compound **49**.

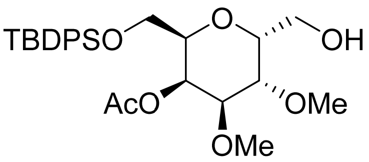

51

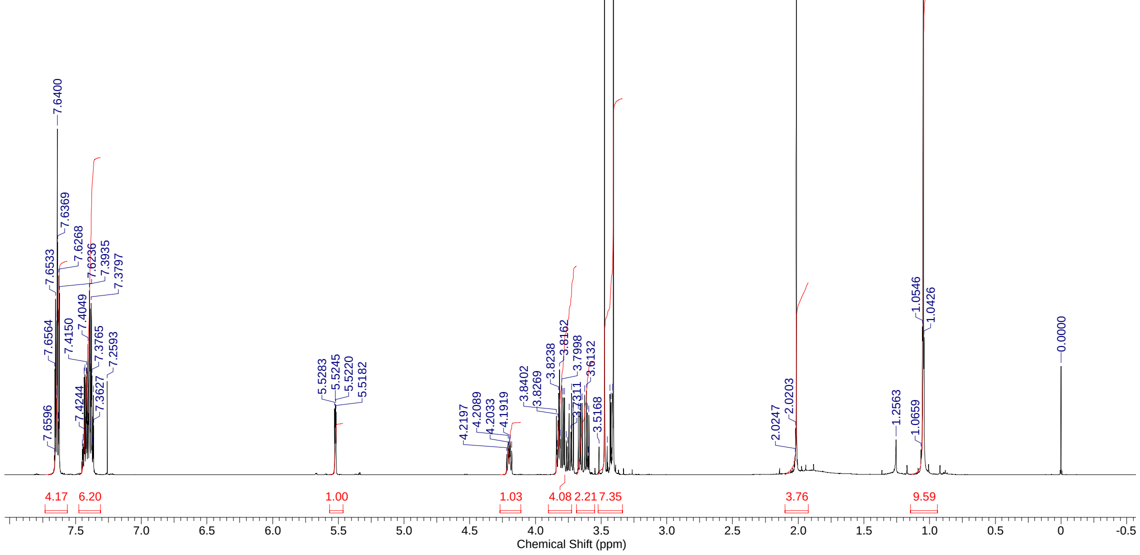

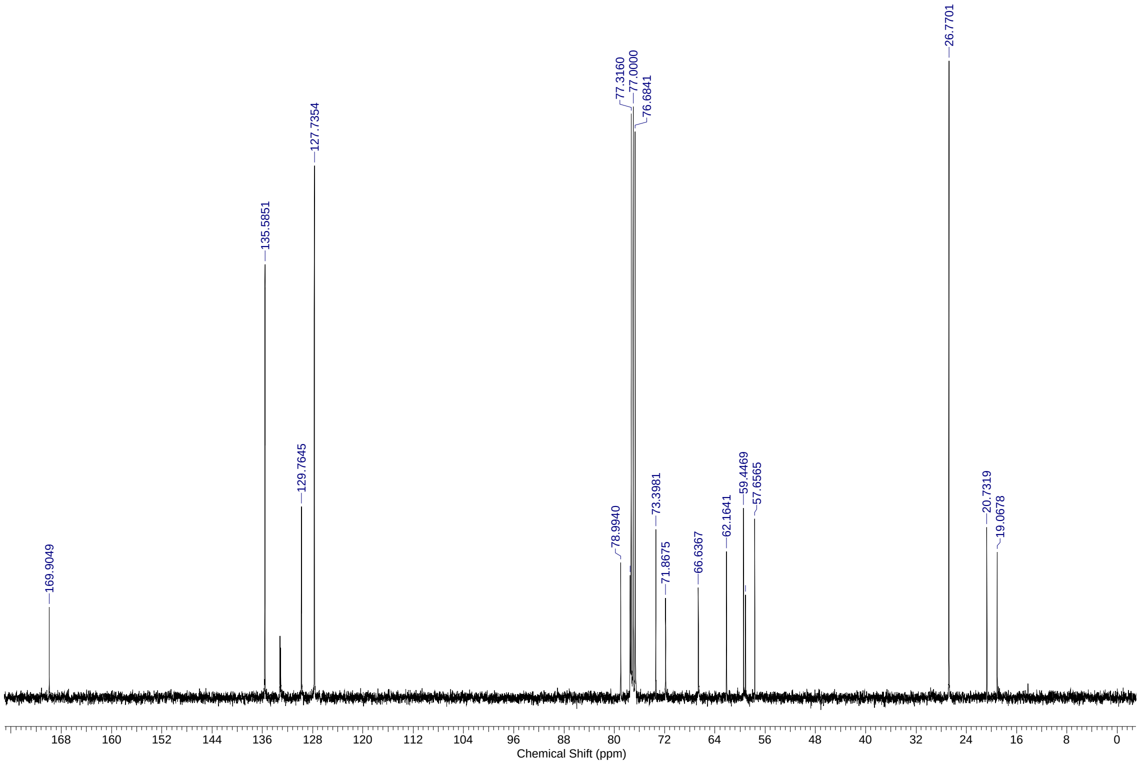

Fig. S52. ^{1}H NMR (500 MHz, $CDCl_3$) and ^{13}C{H} NMR (100.6 MHz, $CDCl_3$) of compound **51**.

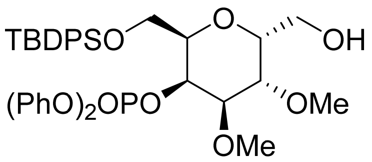

52

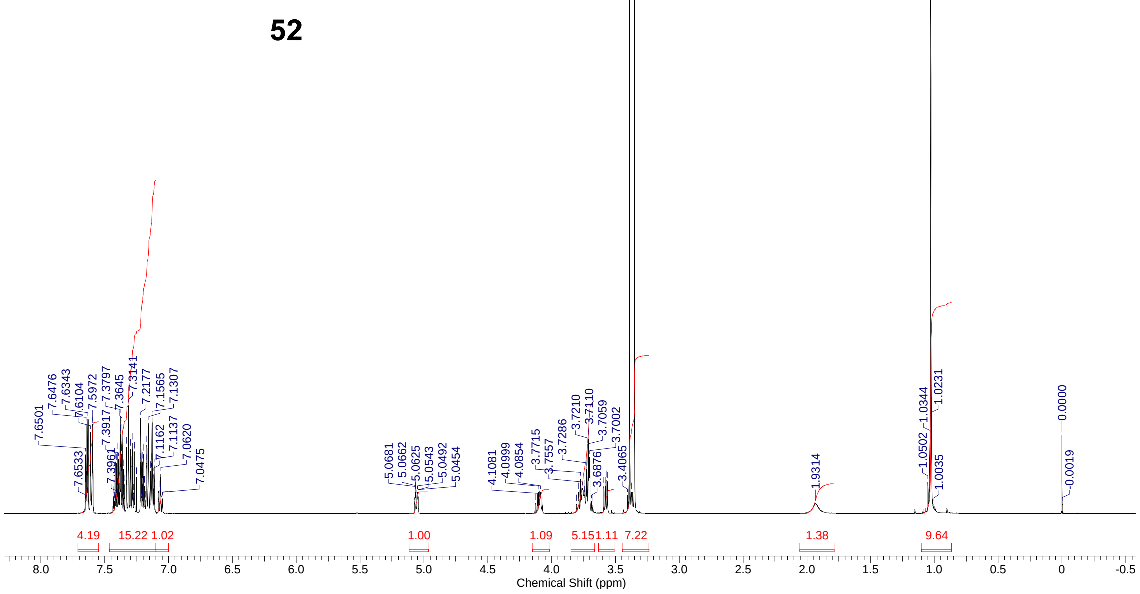

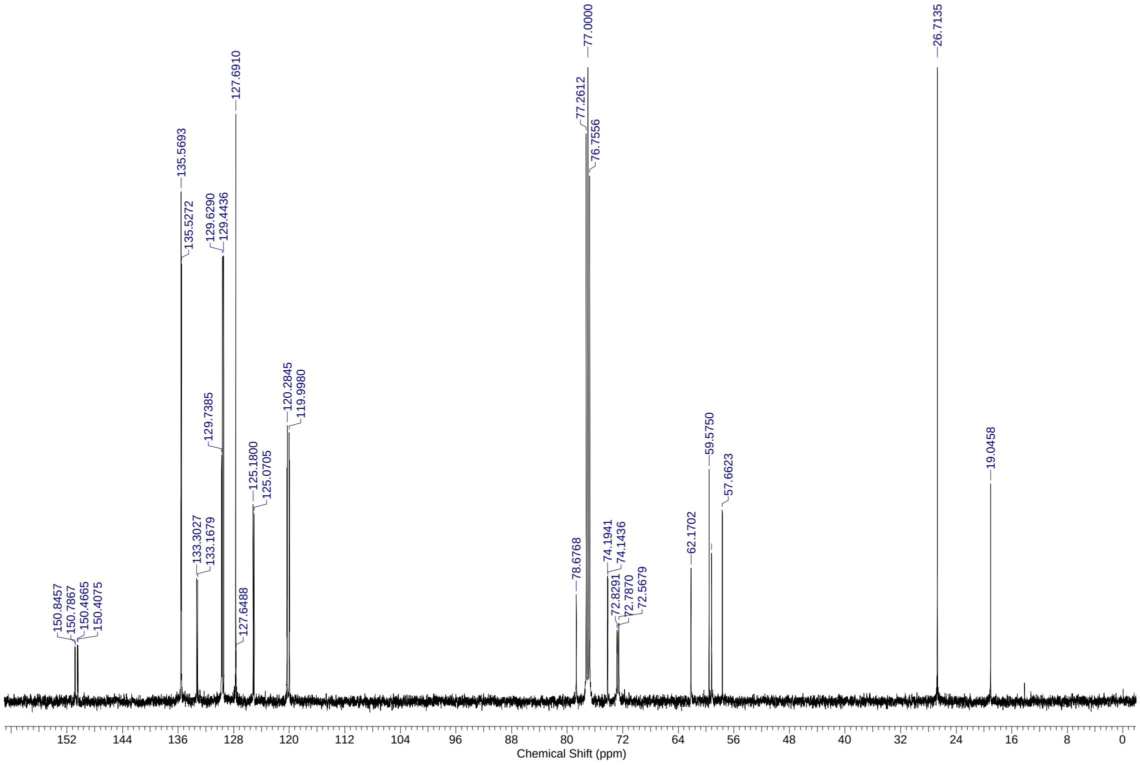

Fig. S53. ^{1}H NMR (500 MHz, $CDCl_3$) and ^{13}C{H} NMR (125.7 MHz, $CDCl_3$) of compound **52**.

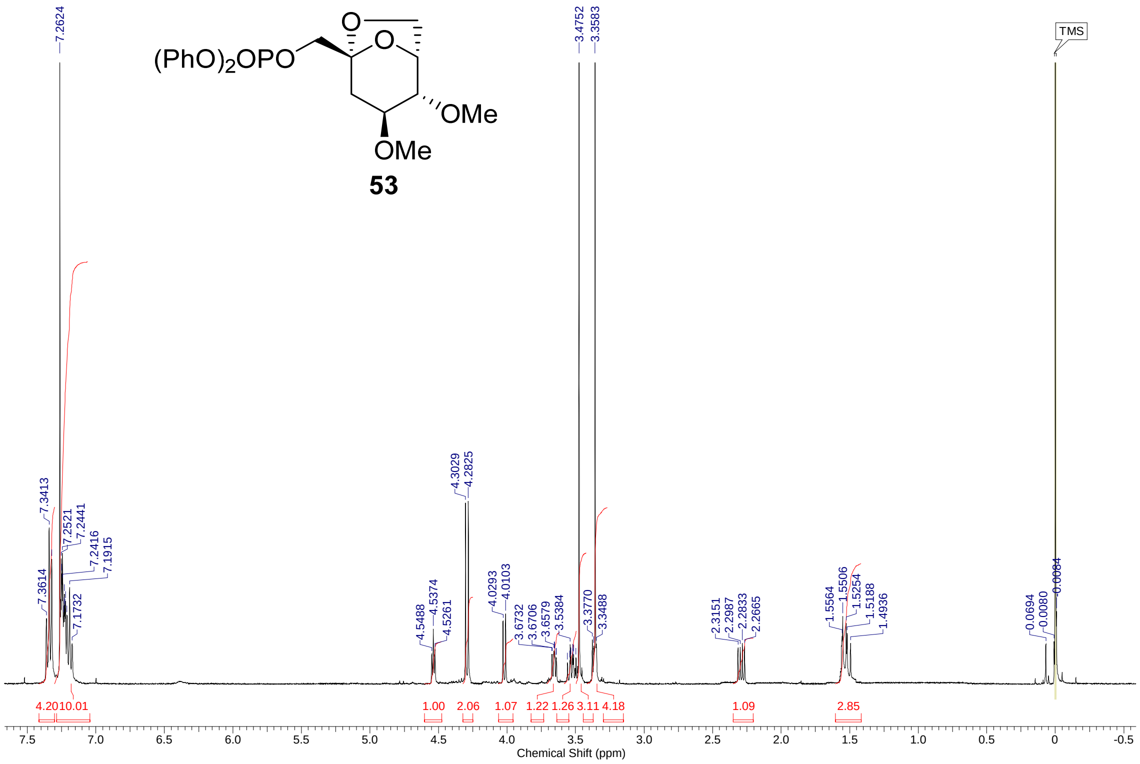

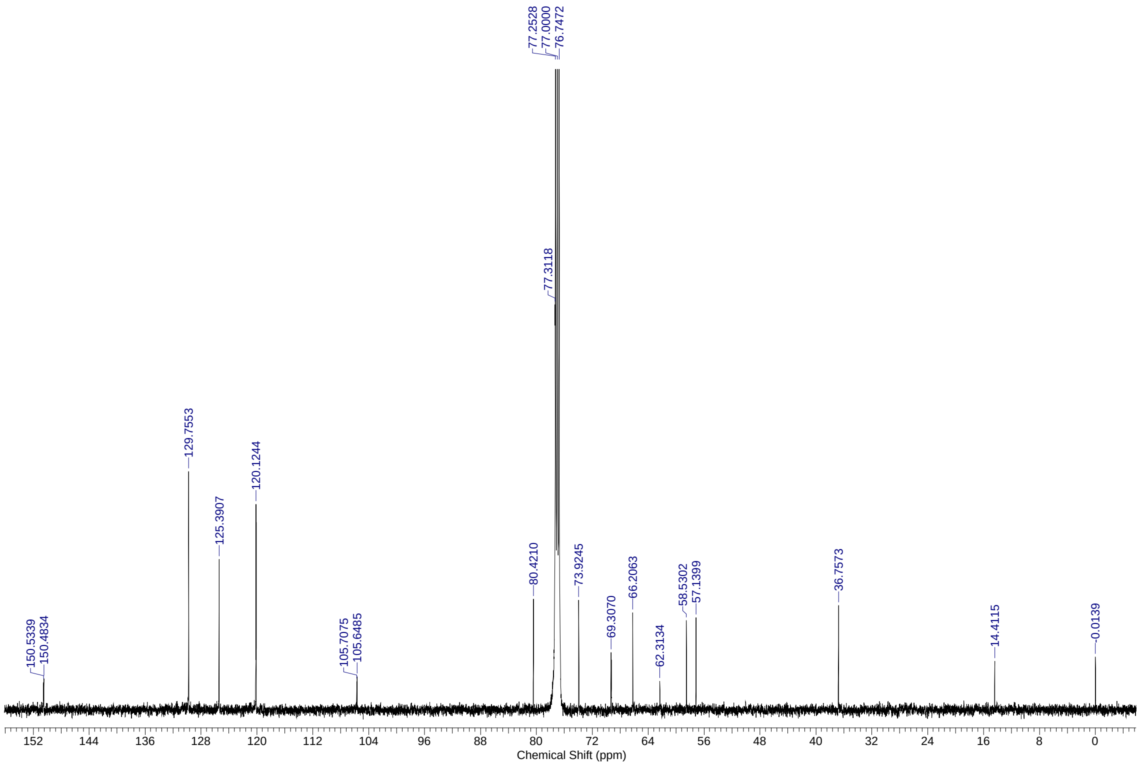

Fig. S54. ^{1}H NMR (500 MHz, $CDCl_3$) and ^{13}C{H} NMR (125.7 MHz, $CDCl_3$) of compound **53**.

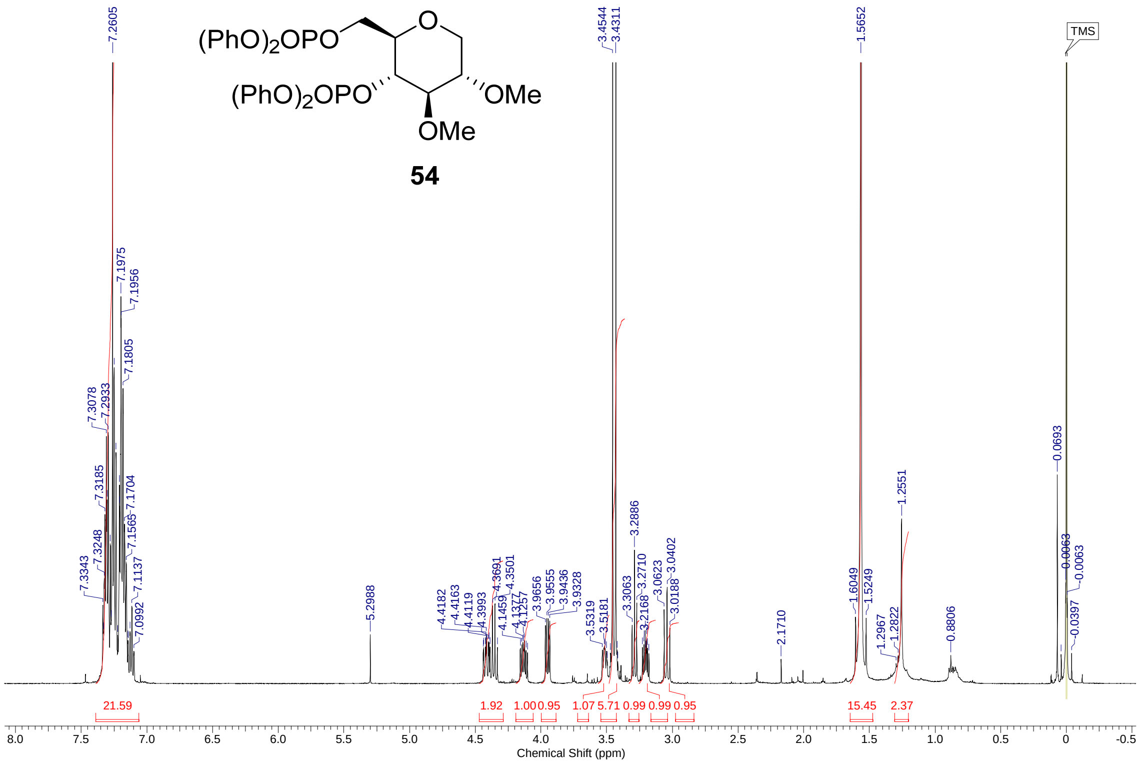

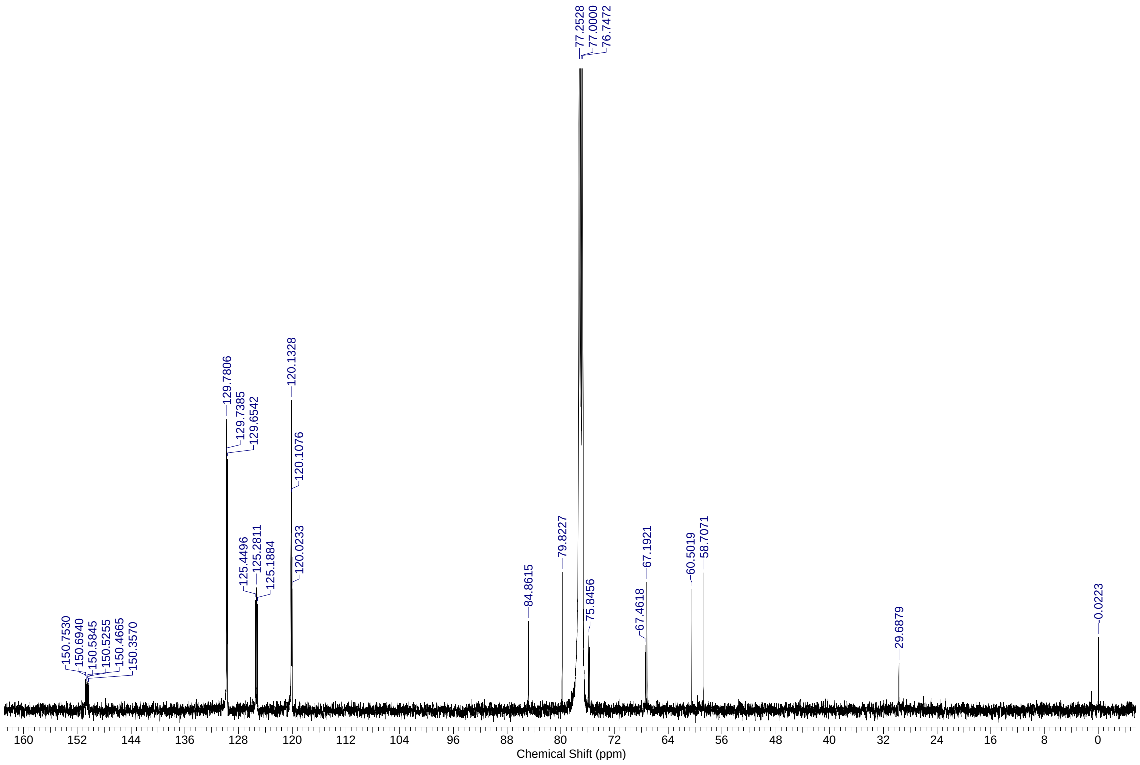

Fig. S55. ^{1}H NMR (500 MHz, $CDCl_3$) and $^{13}C\{H\}$ NMR (125.7 MHz, $CDCl_3$) of compound **54**.

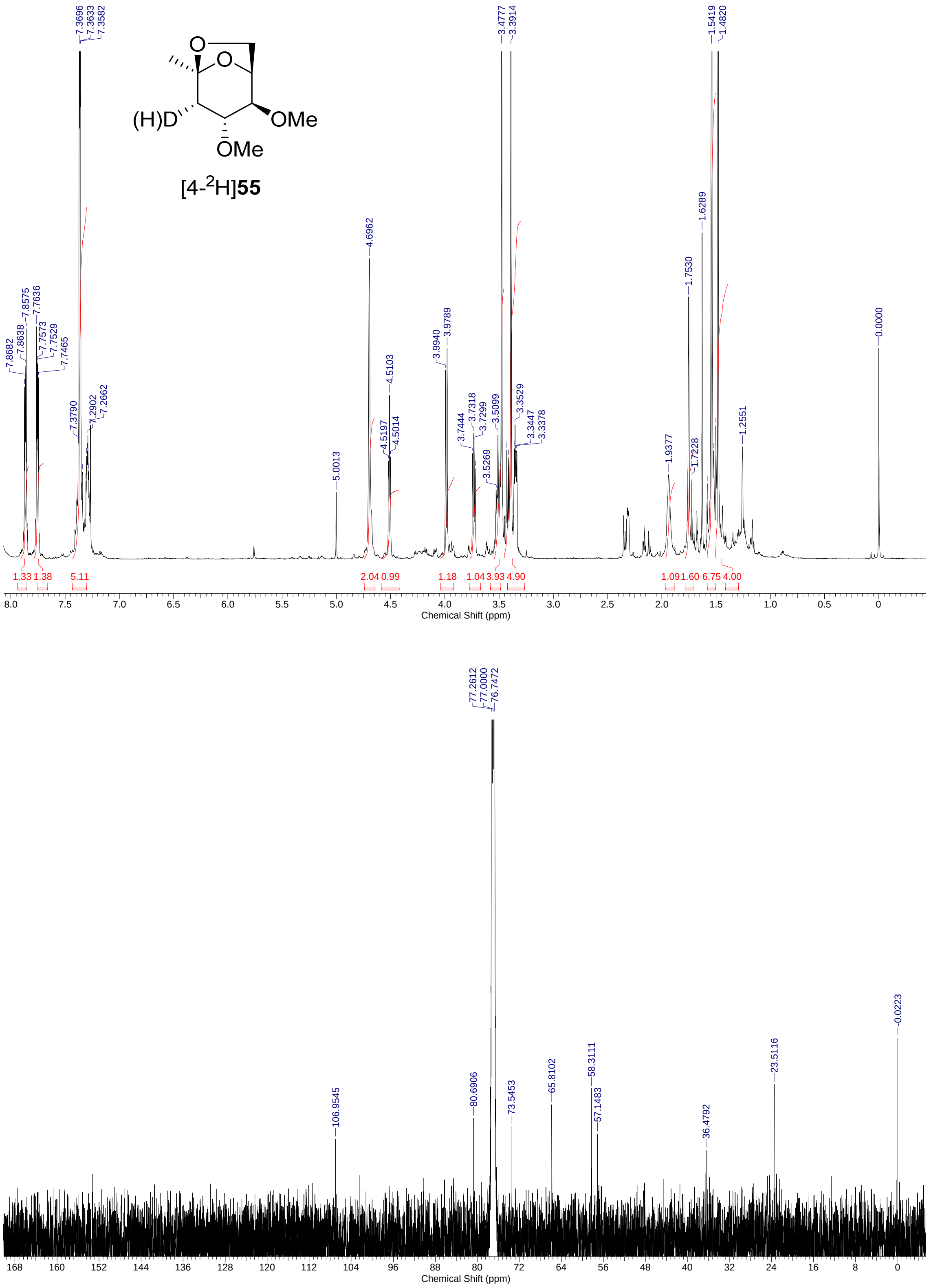

Fig. S56. ^{1}H NMR (500 MHz, $CDCl_3$) and $^{13}C\{H\}$ NMR (125.7 MHz, $CDCl_3$) of compound [4-^{2}H]**55**.

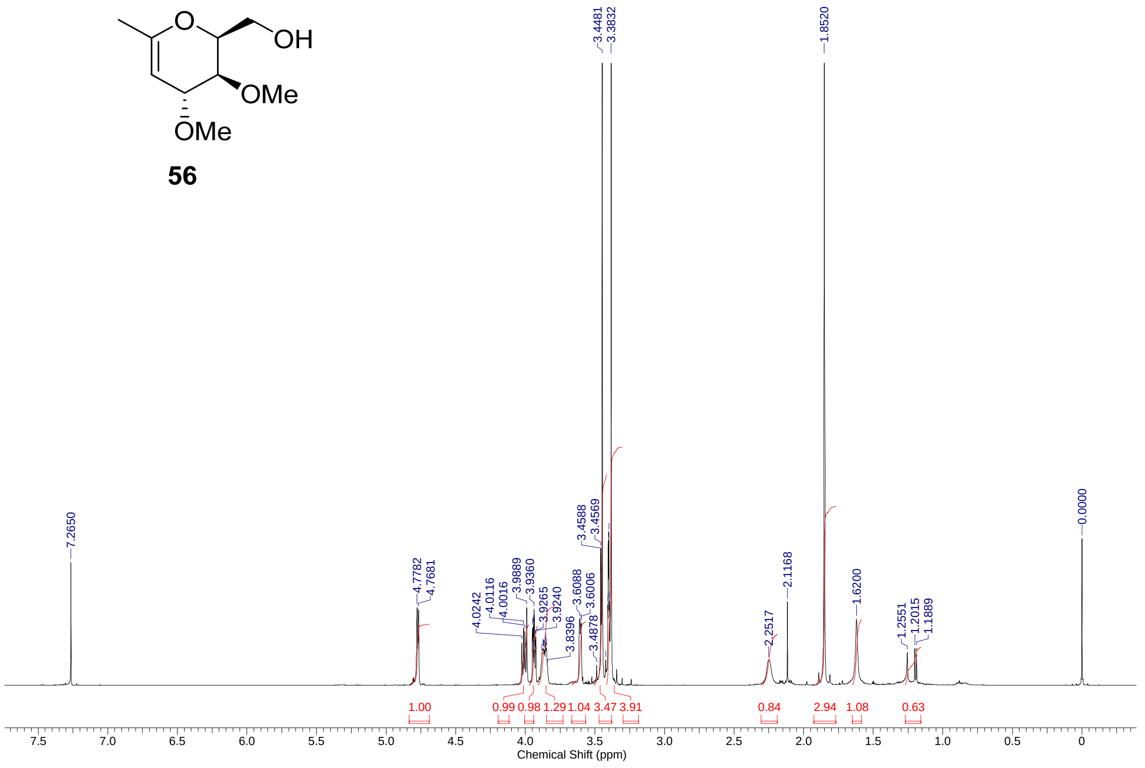

56

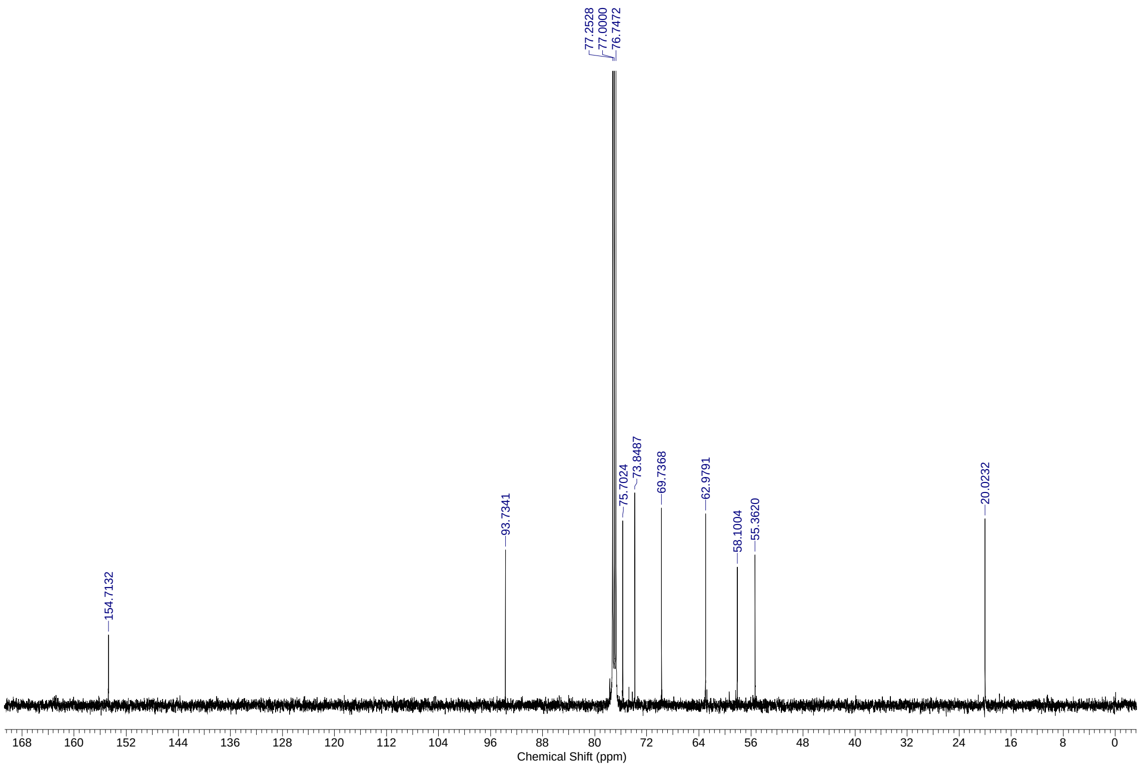

Fig. S57. ^{1}H NMR (500 MHz, $CDCl_3$) and $^{13}C\{H\}$ NMR (125.7 MHz, $CDCl_3$) of compound **56**.

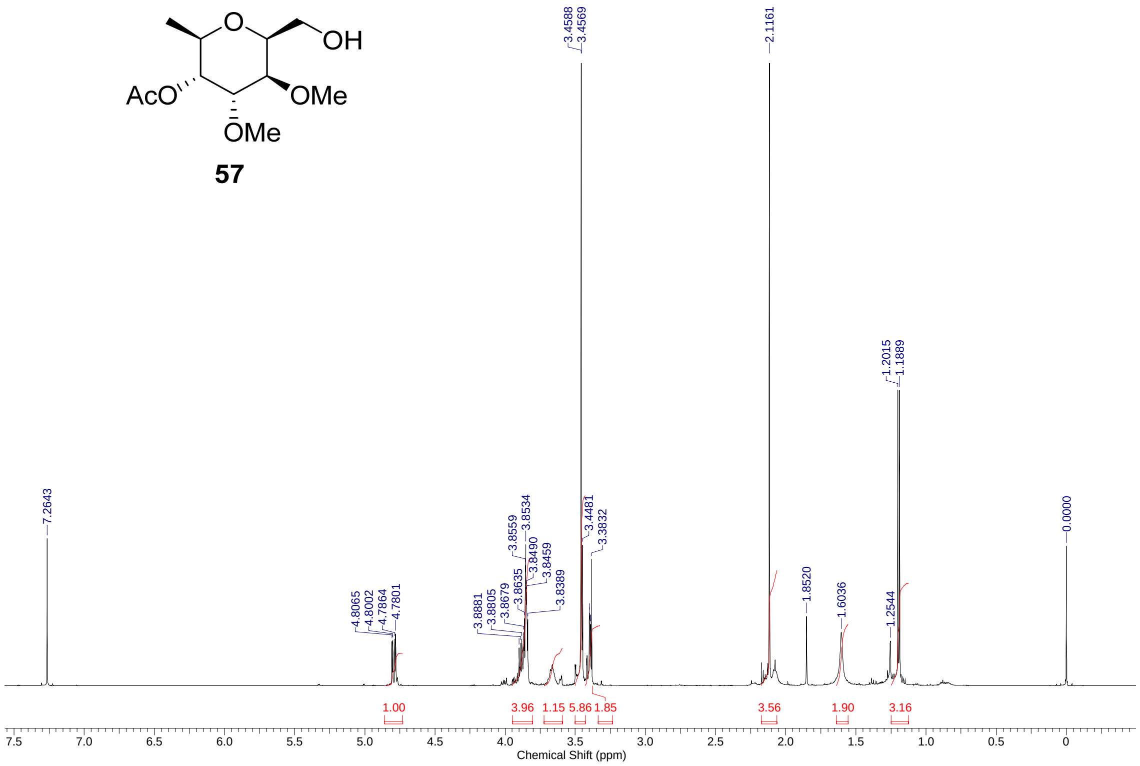

57

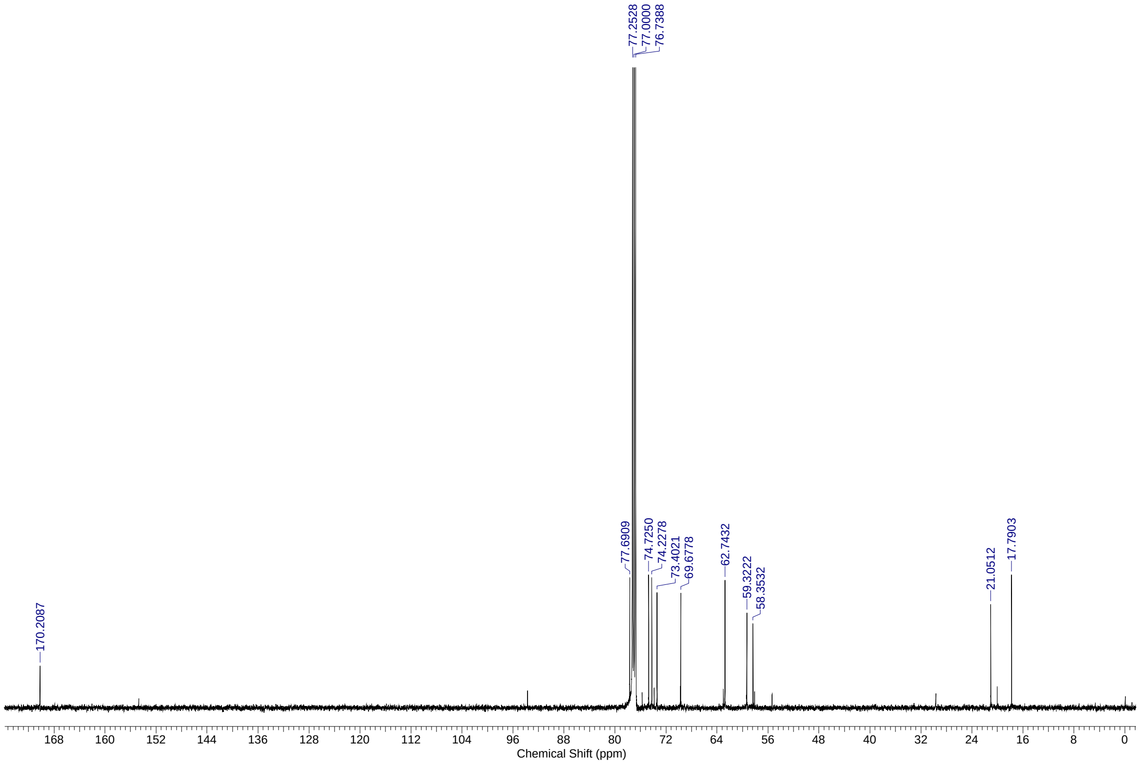

Fig. S58. ^{1}H NMR (500 MHz, $CDCl_3$) and $^{13}C\{H\}$ NMR (125.7 MHz, $CDCl_3$) of compound **57**.

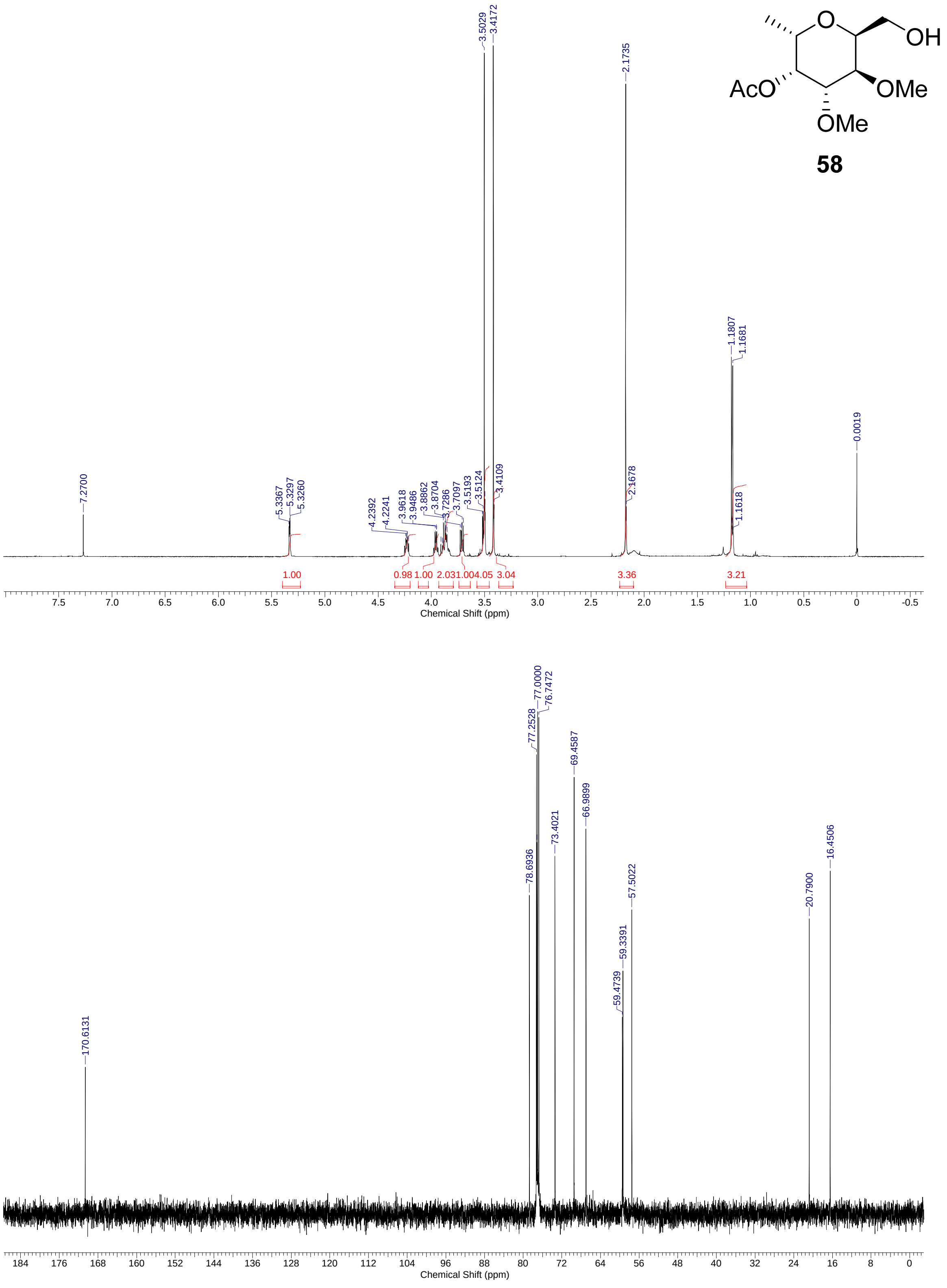

Fig. S59. ^{1}H NMR (500 MHz, $CDCl_3$) and $^{13}C\{H\}$ NMR (125.7 MHz, $CDCl_3$) of compound **58**.

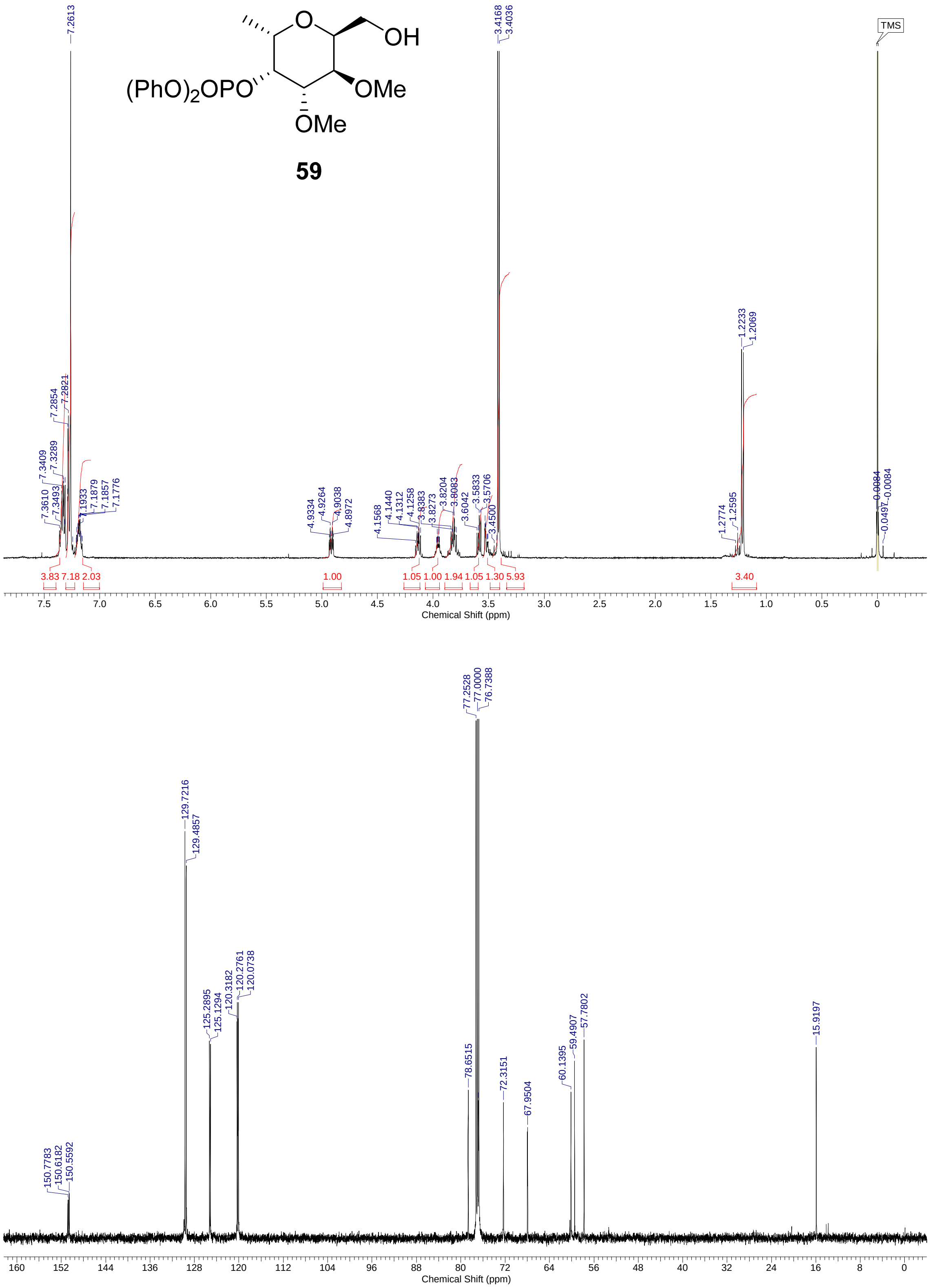

Fig. S60. ^{1}H NMR (400 MHz, $CDCl_3$) and ^{13}C{H} NMR (125.7 MHz, $CDCl_3$) of compound **59**.

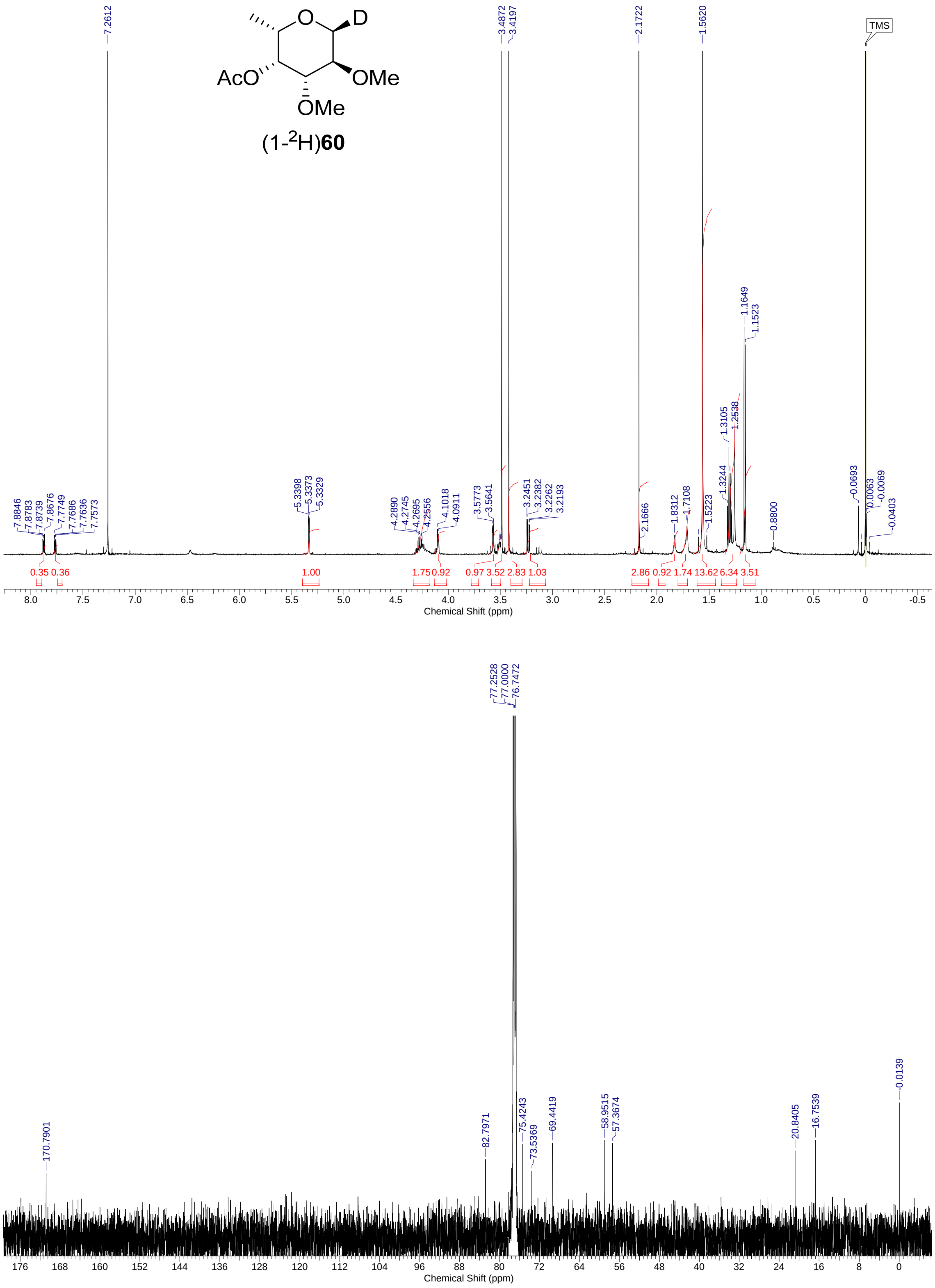

Fig. S61. ^{1}H NMR (500 MHz, $CDCl_3$) and $^{13}C\{H\}$ NMR (125.7 MHz, $CDCl_3$) of compound (1-^{2}H)**60**.

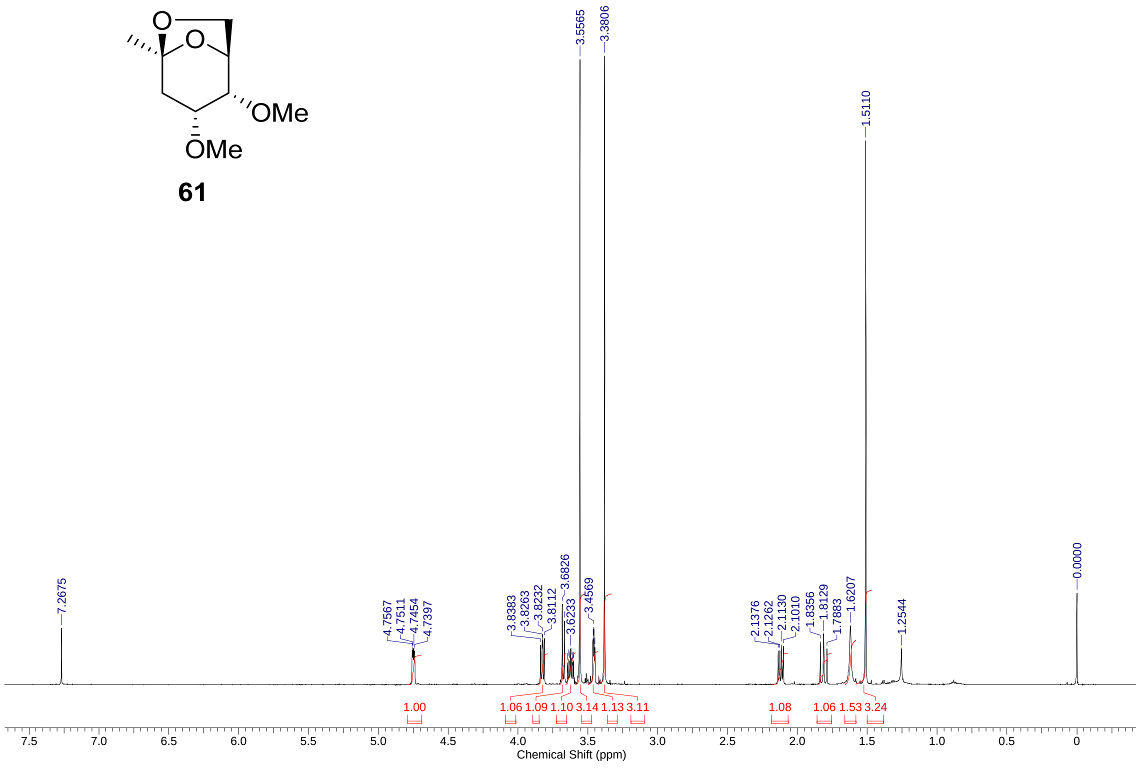

61

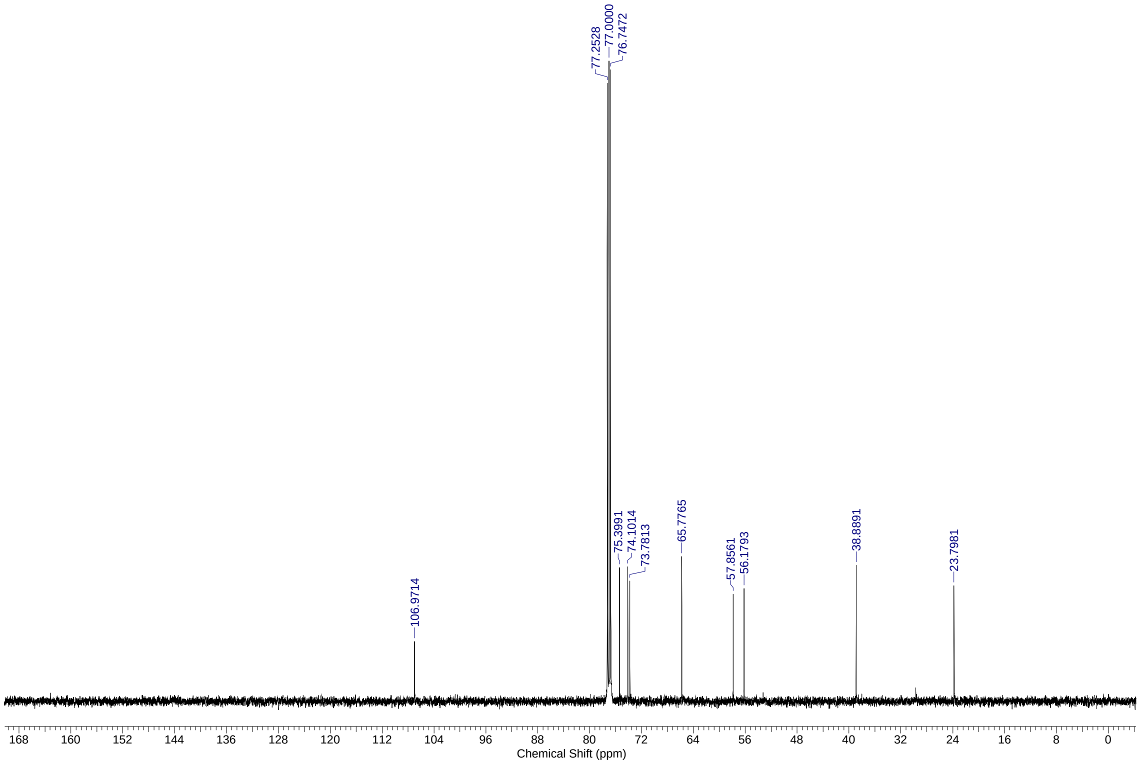

Fig. S62. ^{1}H NMR (500 MHz, $CDCl_3$) and ^{13}C{H} NMR (125.7 MHz, $CDCl_3$) of compound **61**.

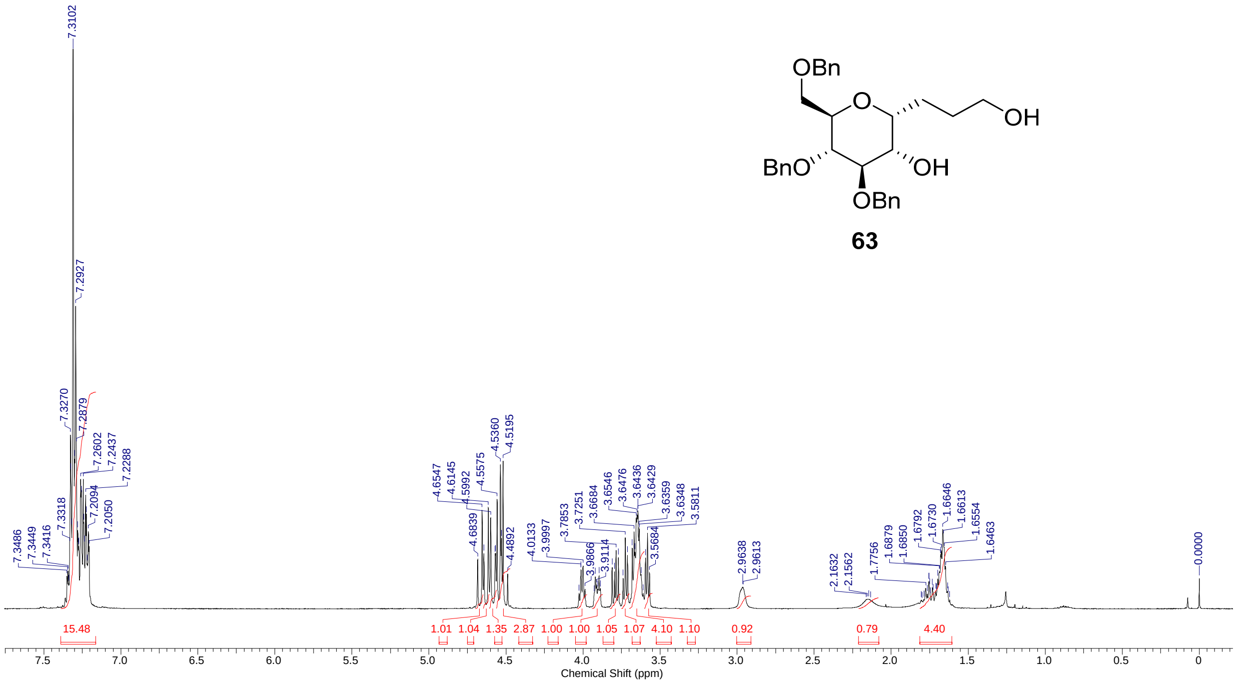

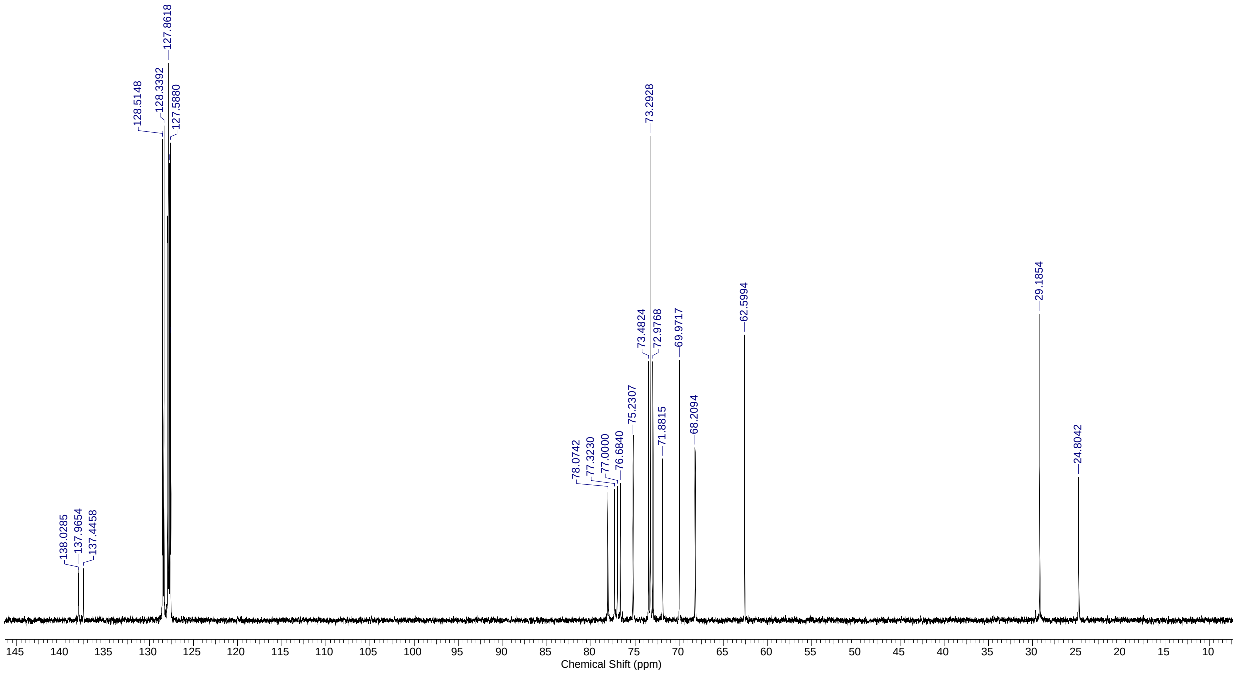

Fig. S63. ^{1}H NMR (400 MHz, $CDCl_3$) and ^{13}C{H} NMR (100.6 MHz, $CDCl_3$) of compound **63**.

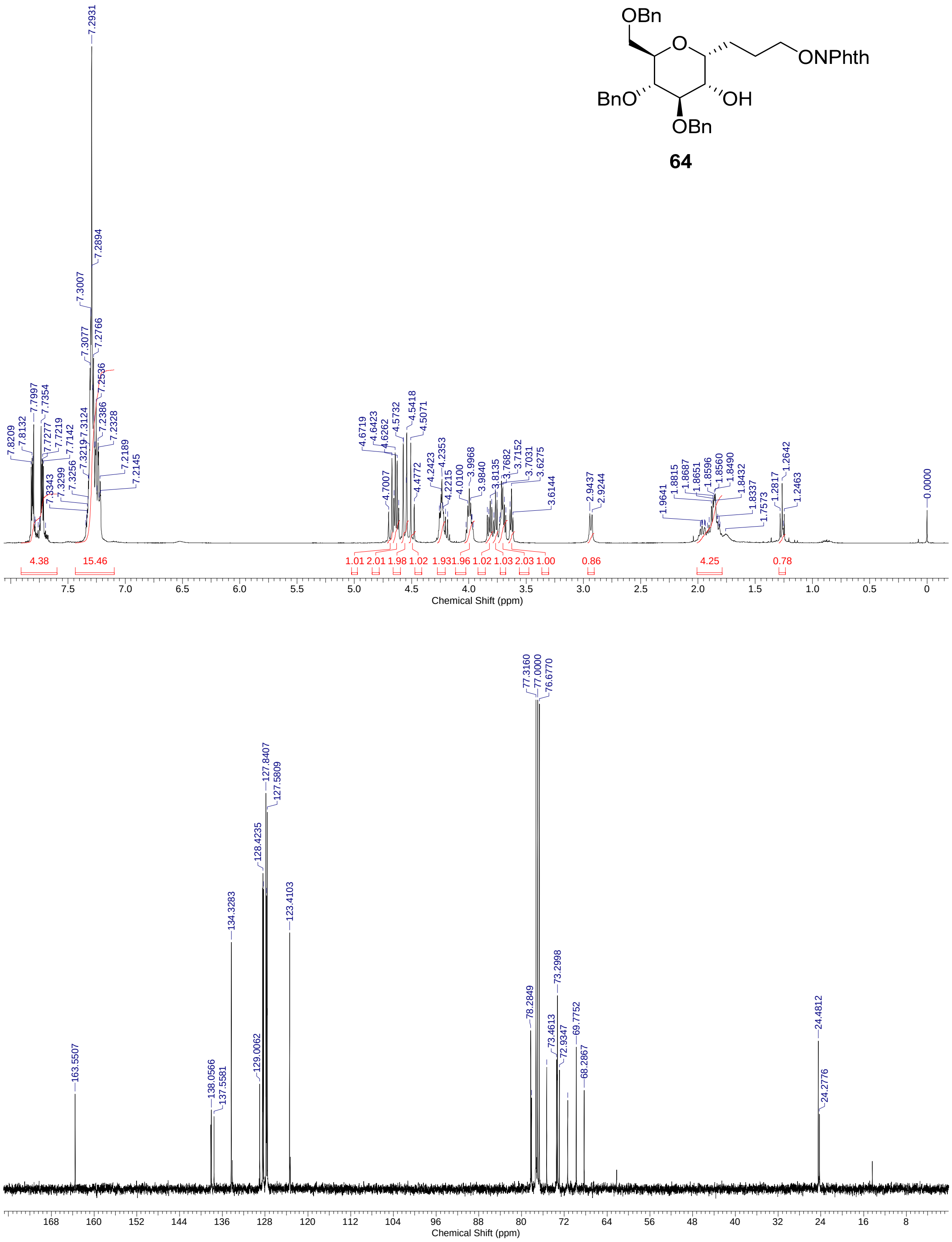

Fig. S64. ^{1}H NMR (400 MHz, $CDCl_3$) and ^{13}C{H} NMR (100.6 MHz, $CDCl_3$) of compound **64**.

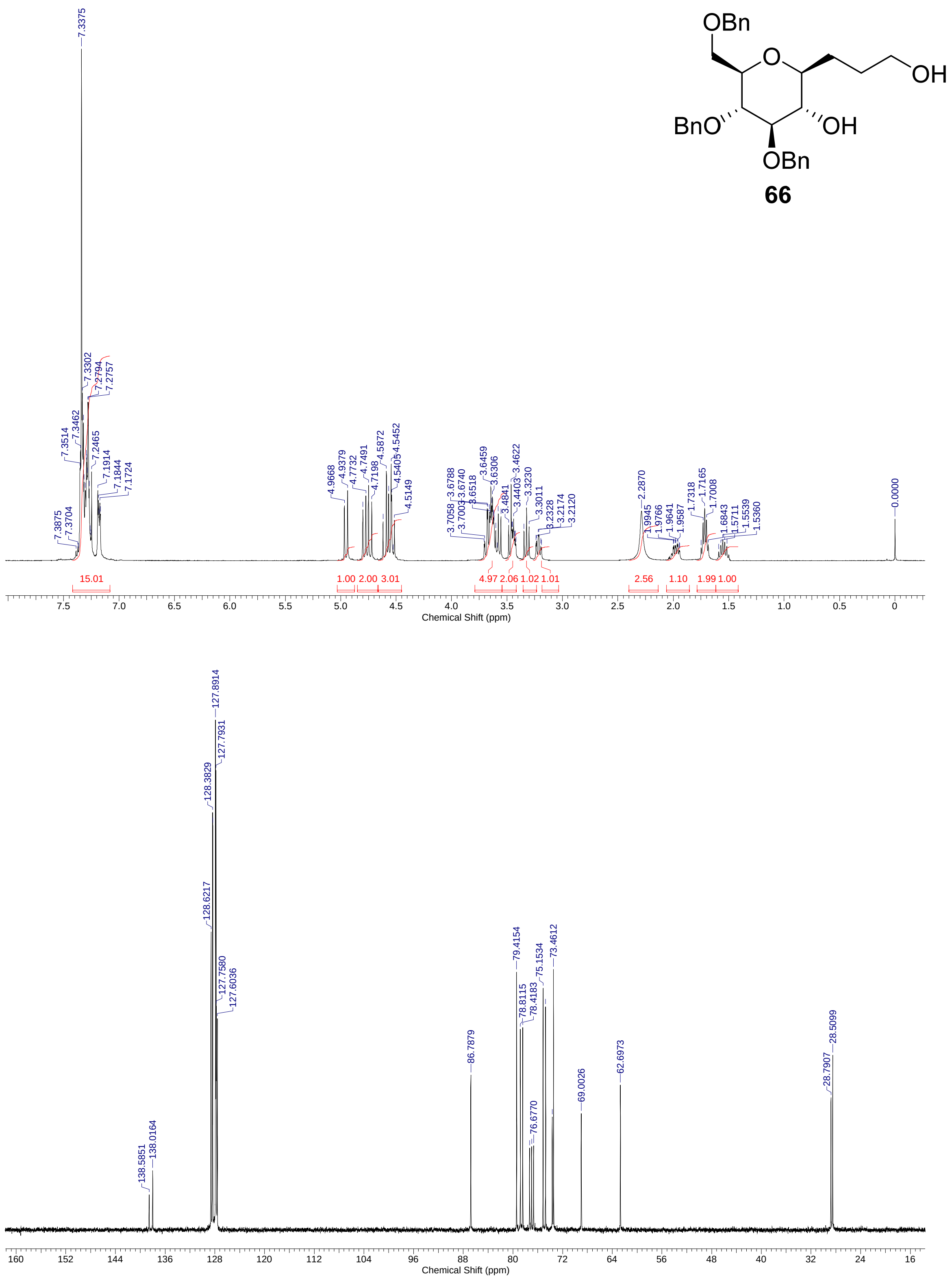

Fig. S65. ^{1}H NMR (400 MHz, $CDCl_3$) and $^{13}C\{H\}$ NMR (100.6 MHz, $CDCl_3$) of compound **66**.

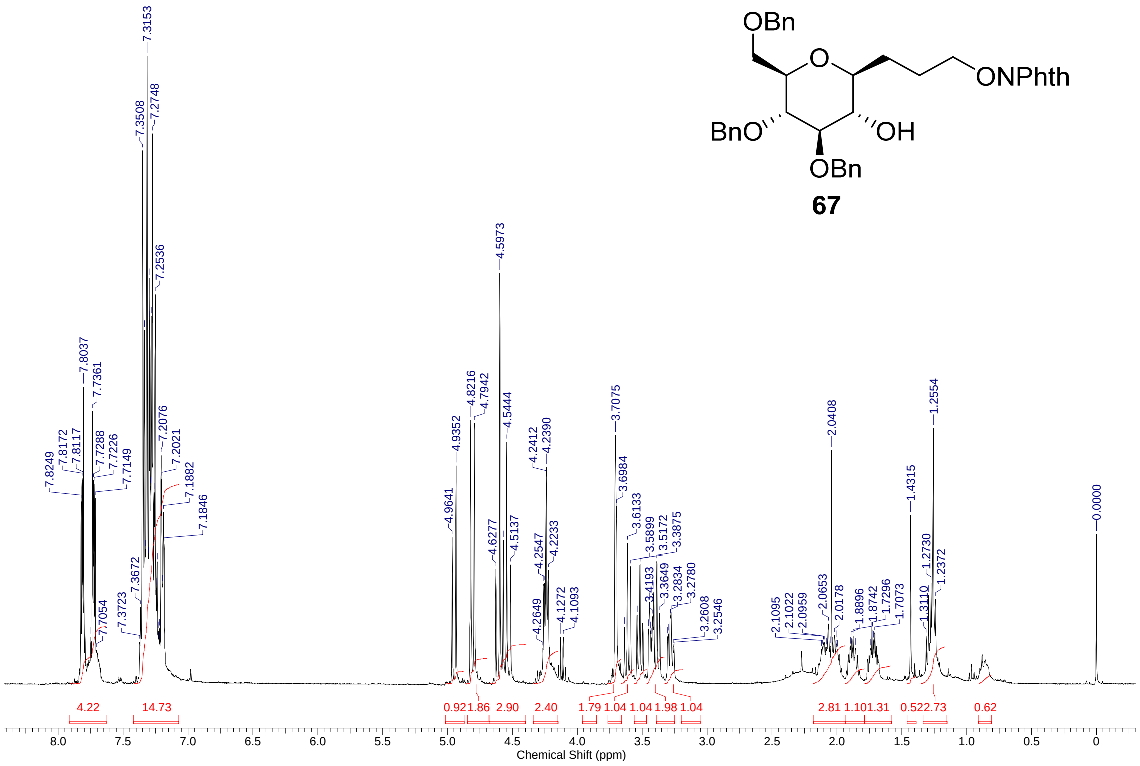

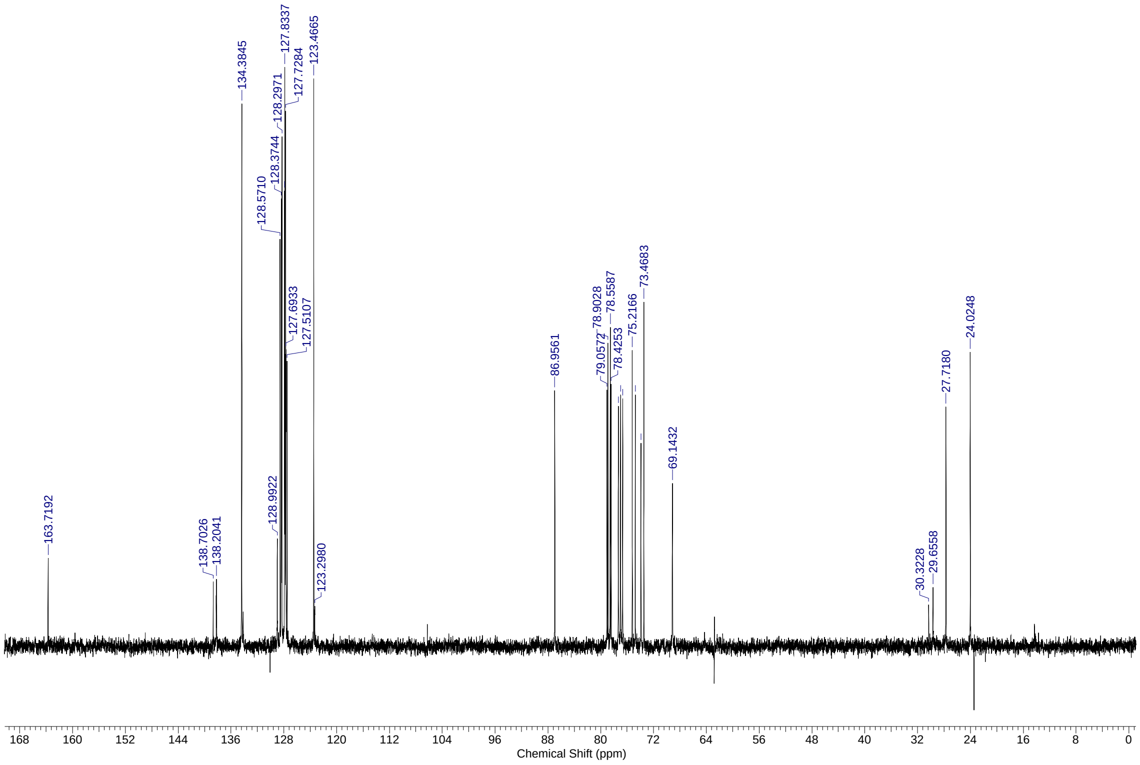

Fig. S66. ^{1}H NMR (400 MHz, $CDCl_3$) and $^{13}C\{H\}$ NMR (100.6 MHz, $CDCl_3$) of compound **67**.

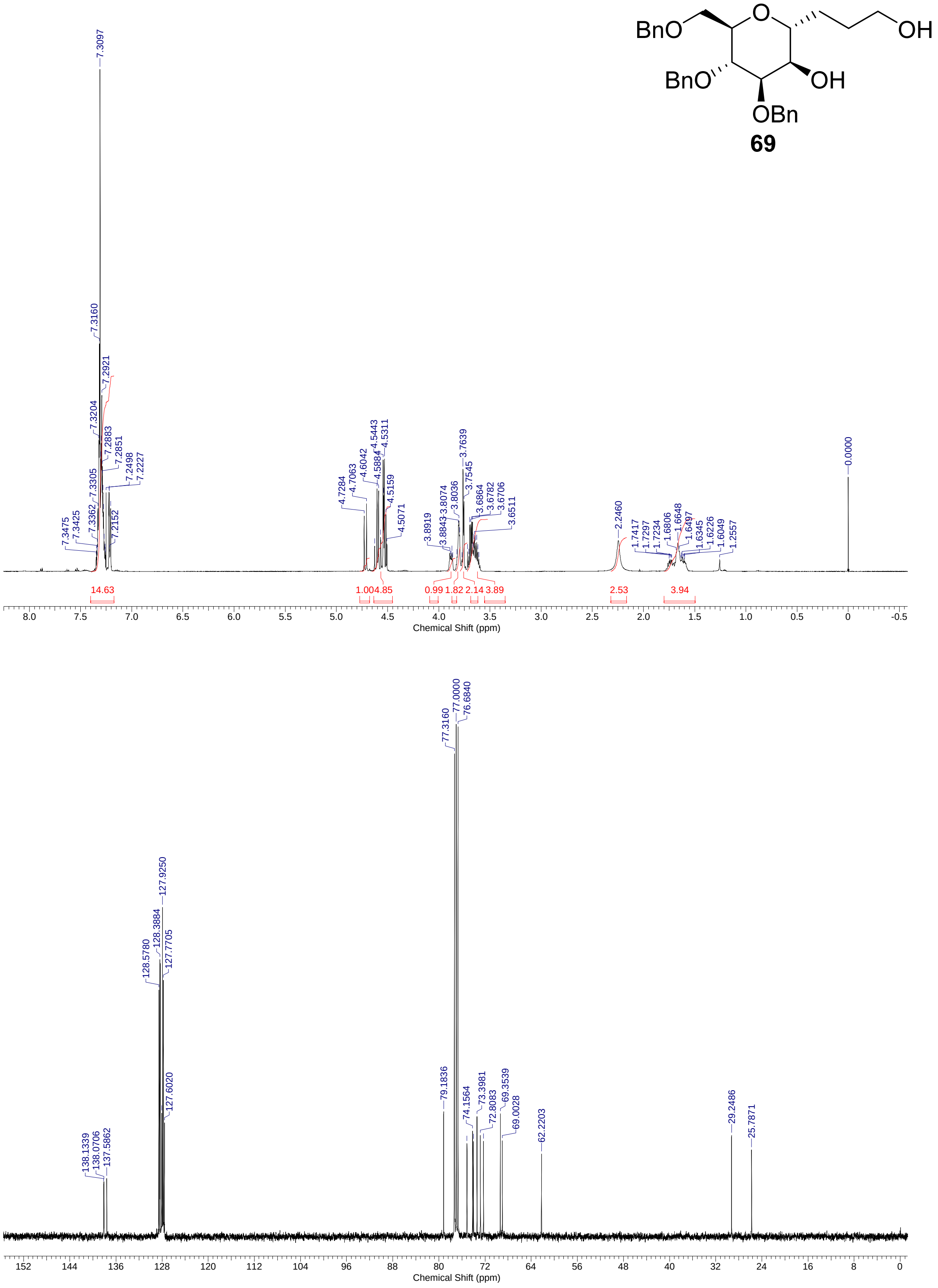

Fig. S67. ^{1}H NMR (500 MHz, $CDCl_3$) and ^{13}C{H} NMR (100.6 MHz, $CDCl_3$) of compound **69**.

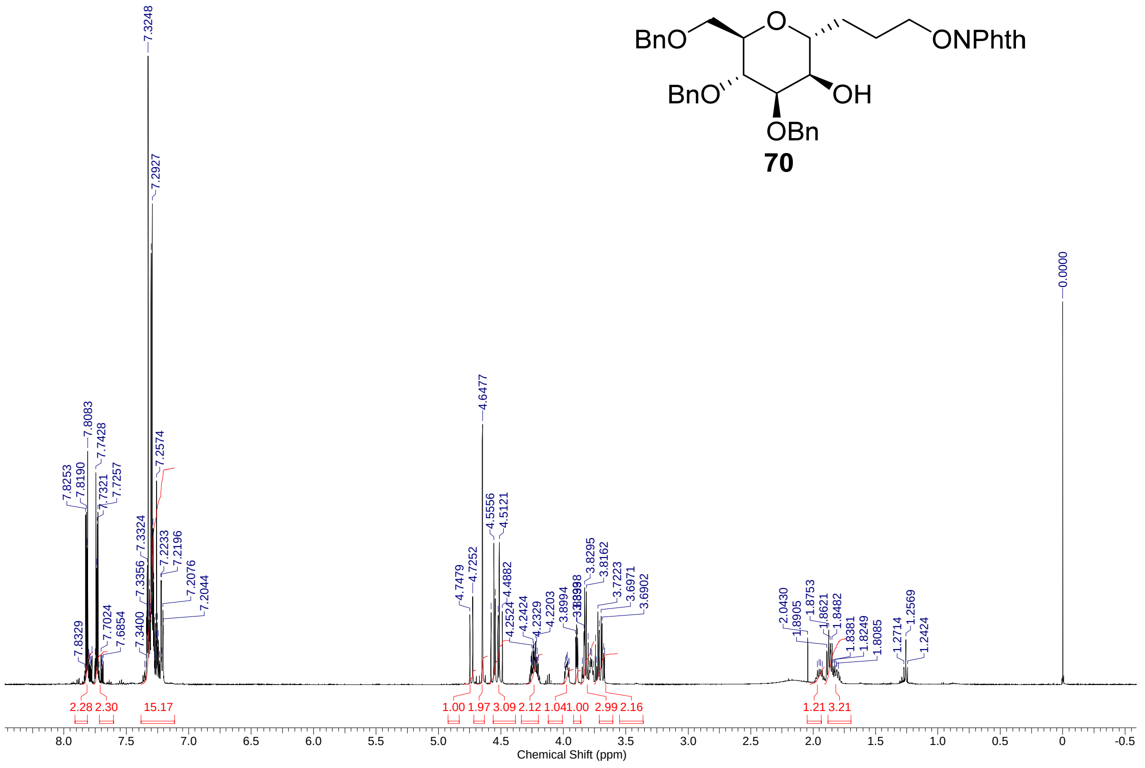

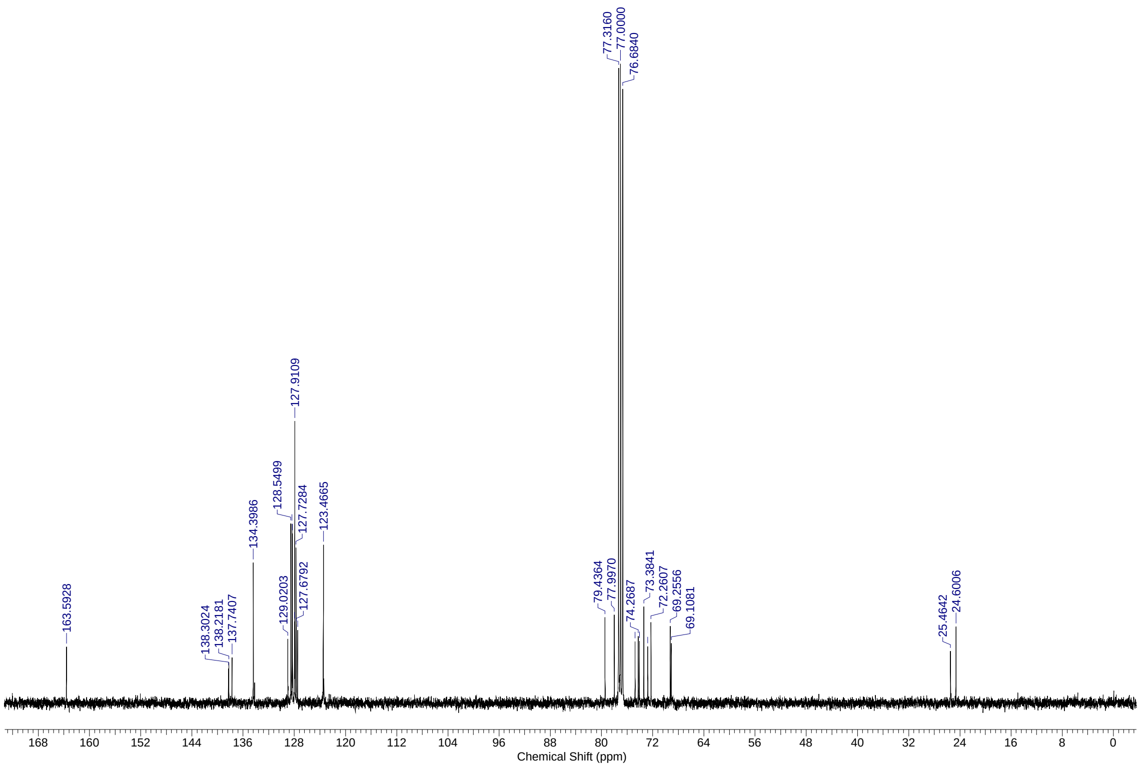

Fig. S68. ^{1}H NMR (500 MHz, $CDCl_3$) and ^{13}C{H} NMR (100.6 MHz, $CDCl_3$) of compound **70**.

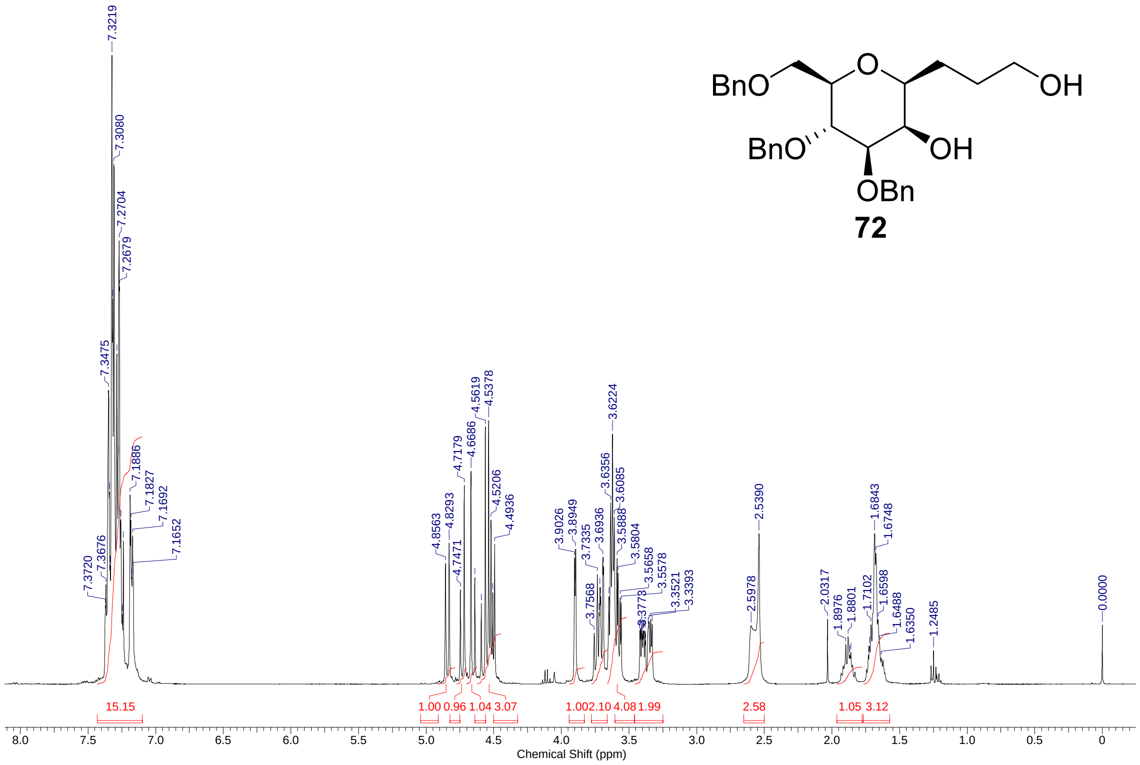

72

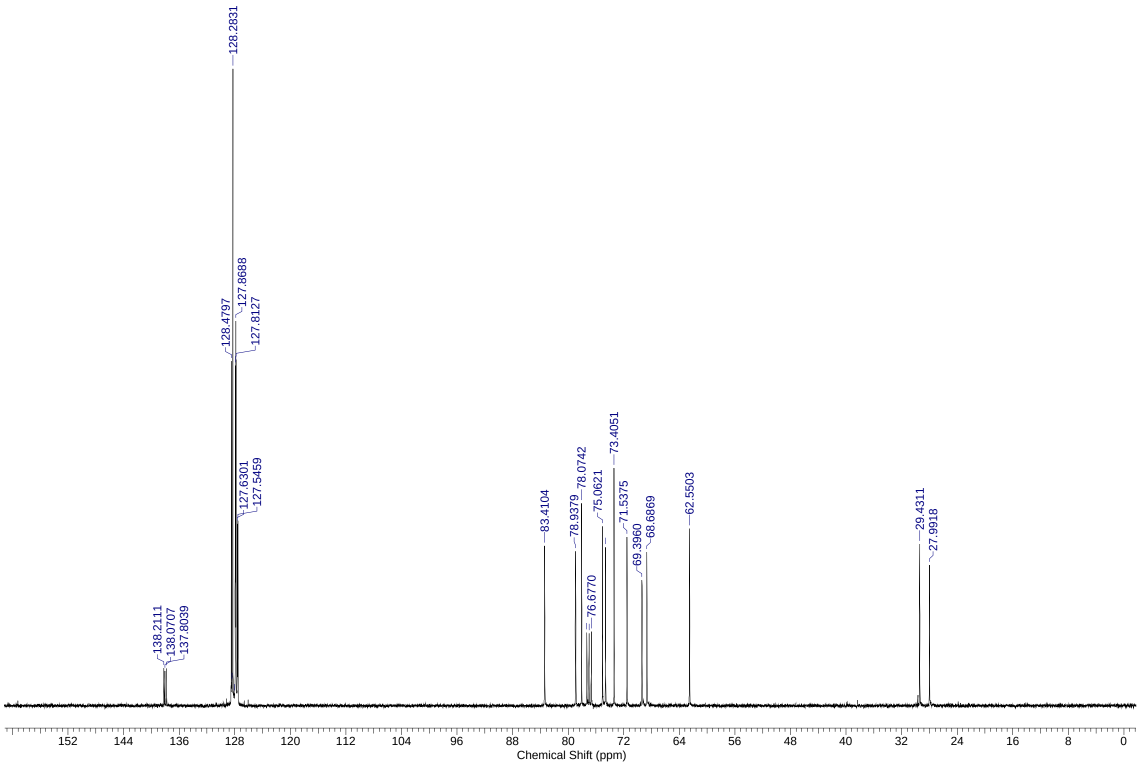

Fig. S69. ^{1}H NMR (400 MHz, $CDCl_3$) and ^{13}C{H} NMR (100.6 MHz, $CDCl_3$) of compound **72**.

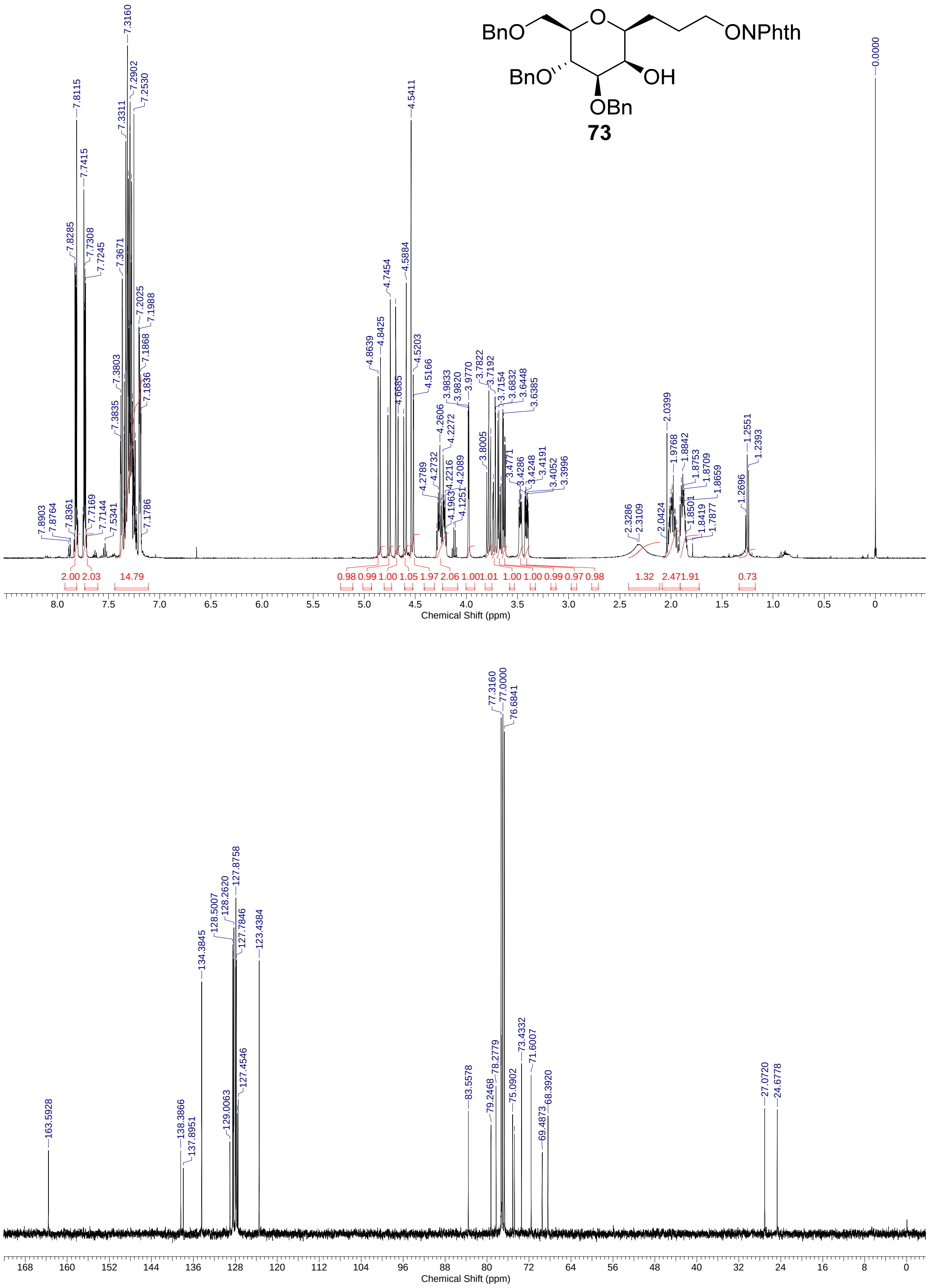

Fig. S70. ^{1}H NMR (500 MHz, $CDCl_3$) and $^{13}C\{H\}$ NMR (100.6 MHz, $CDCl_3$) of compound **73**.

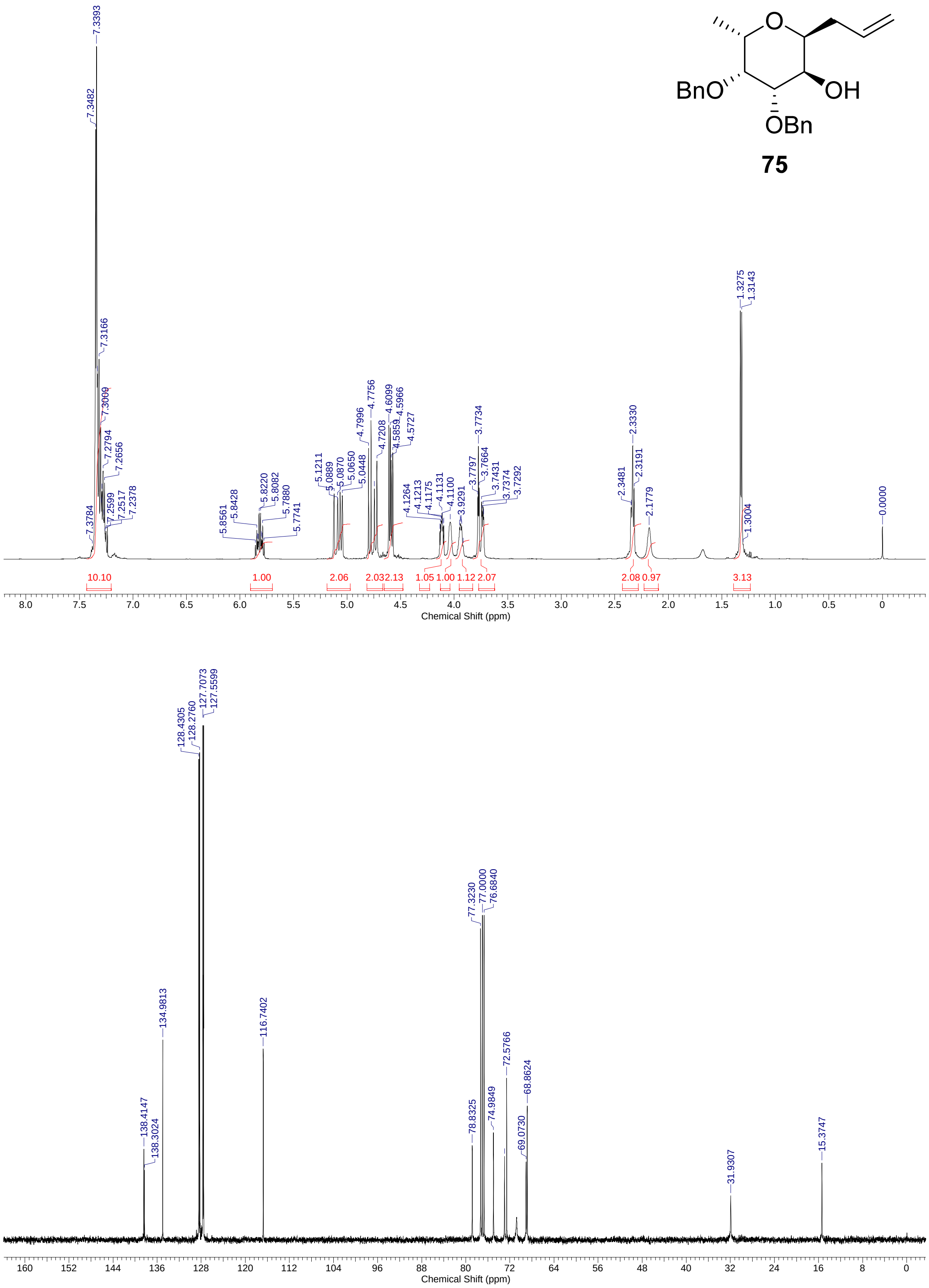

Fig. S71. ^{1}H NMR (500 MHz, $CDCl_3$) and $^{13}C\{H\}$ NMR (100.6 MHz, $CDCl_3$) of compound **75**.

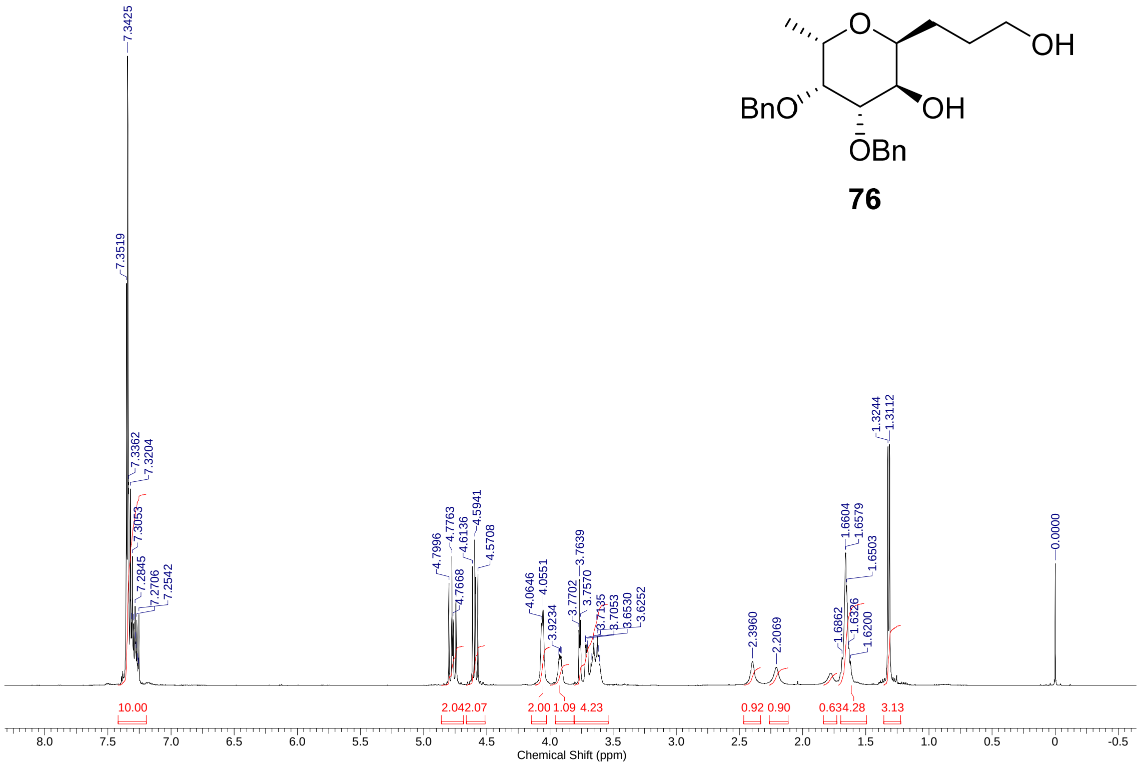

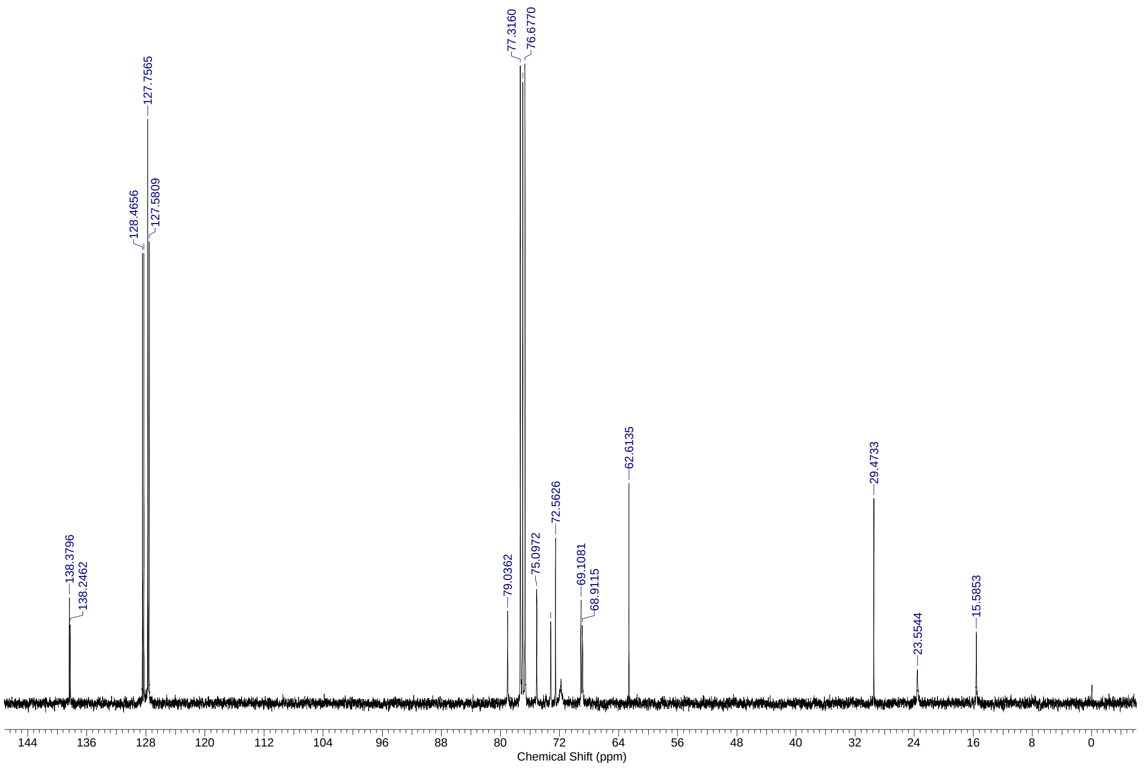

Fig. S72. ^{1}H NMR (500 MHz, $CDCl_3$) and ^{13}C{H} NMR (100.6 MHz, $CDCl_3$) of compound **76**.

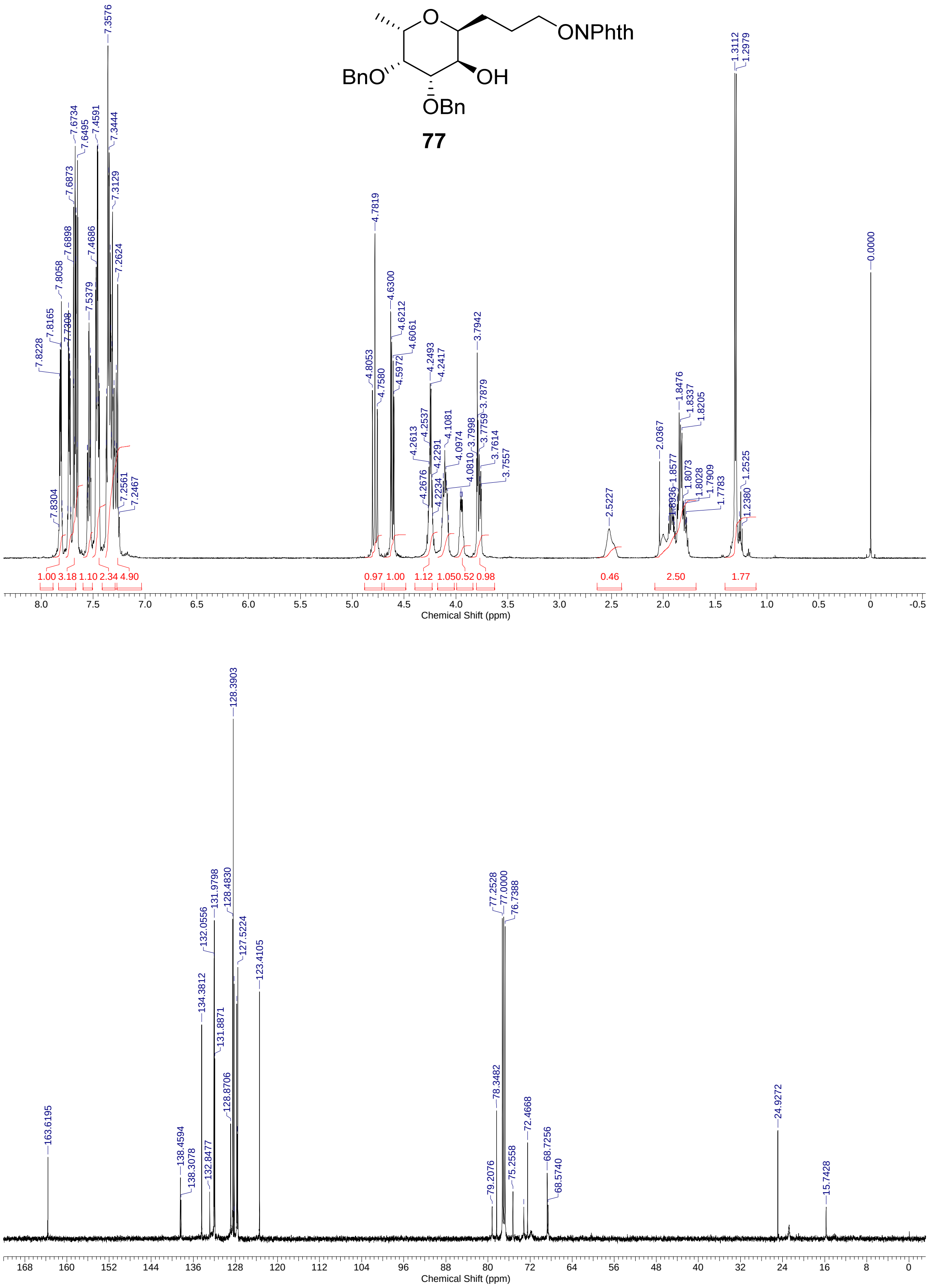

Fig. S73. ^{1}H NMR (500 MHz, $CDCl_3$) and ^{13}C{H} NMR (125.7 MHz, $CDCl_3$) of compound **77**.

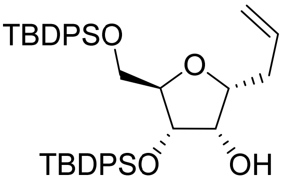

83

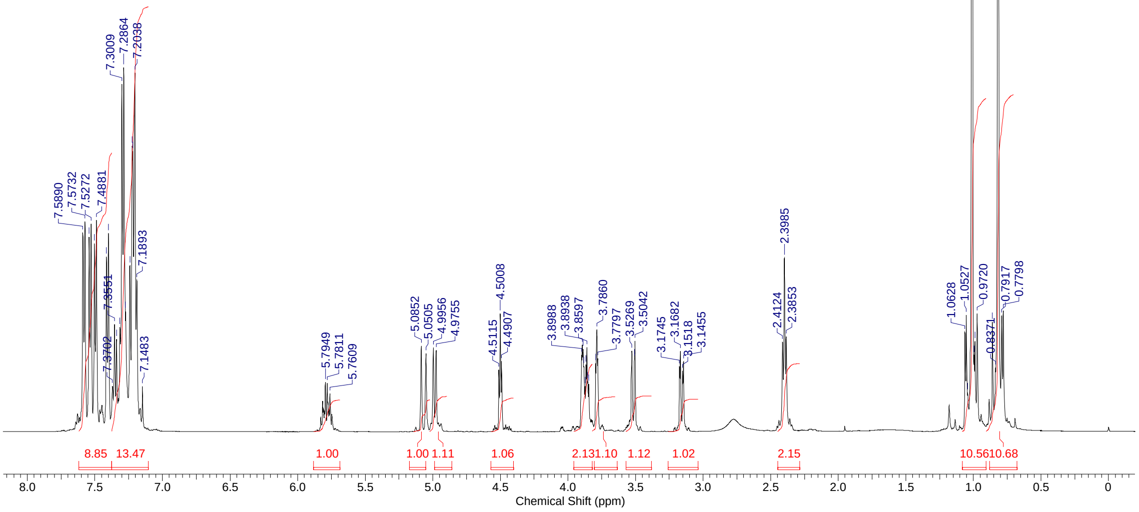

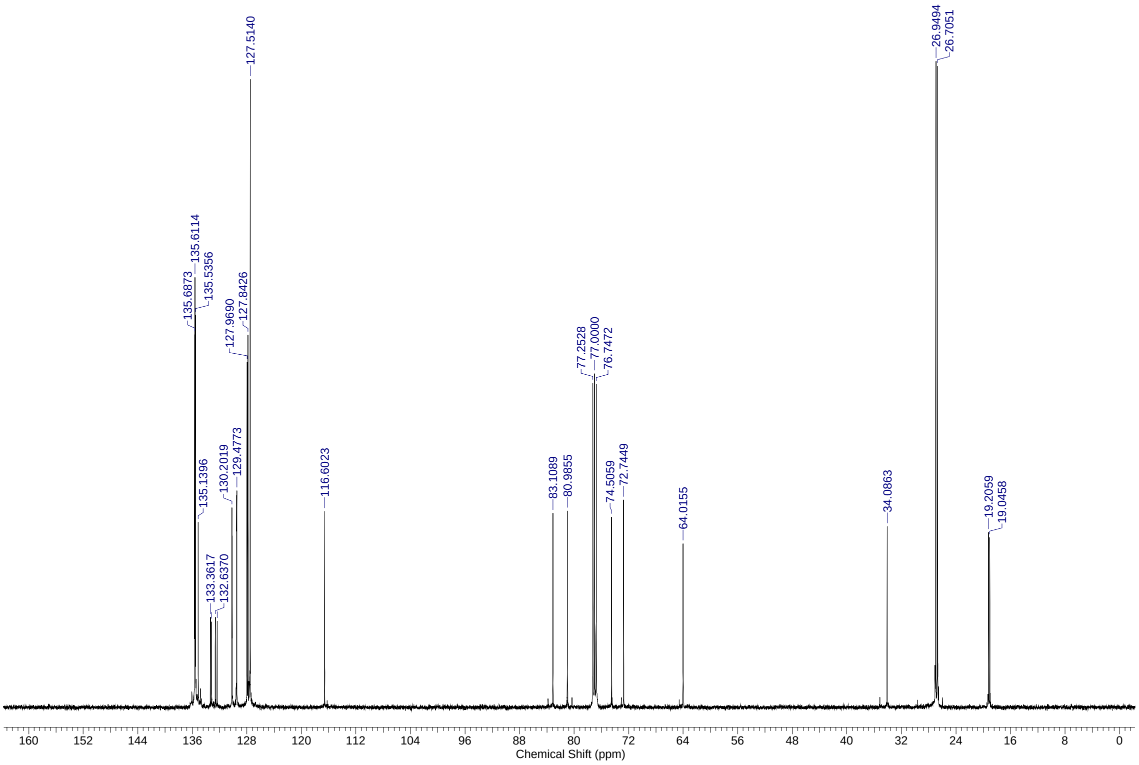

Fig. S74. ^{1}H NMR (500 MHz, $CDCl_3$) and $^{13}C\{H\}$ NMR (125.7 MHz, $CDCl_3$) of compound **83**.

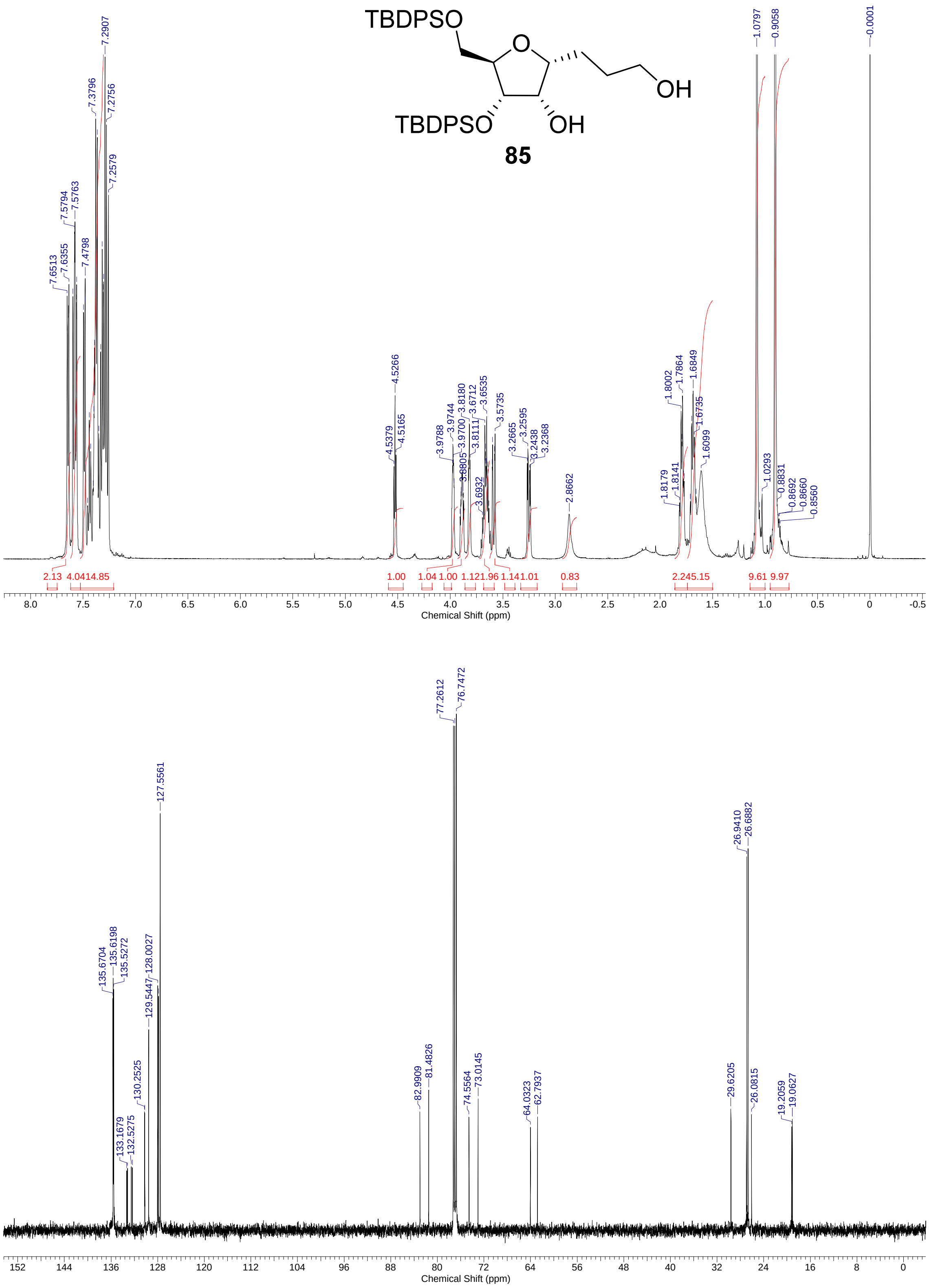

Fig. S75. ^{1}H NMR (500 MHz, $CDCl_3$) and ^{13}C{H} NMR (125.7 MHz, $CDCl_3$) of compound **85**.

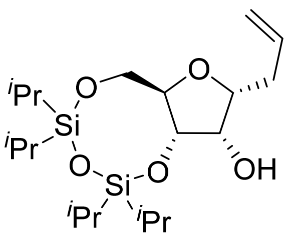

86

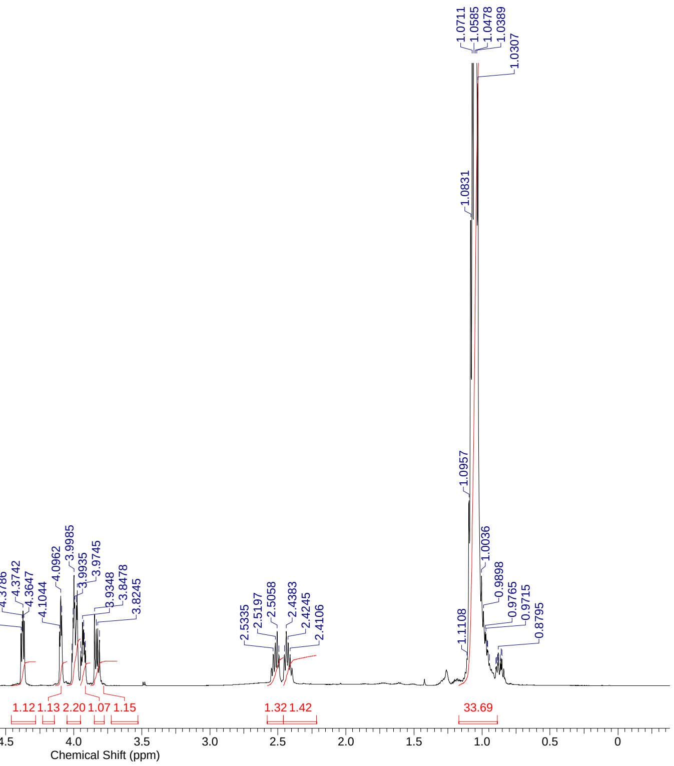

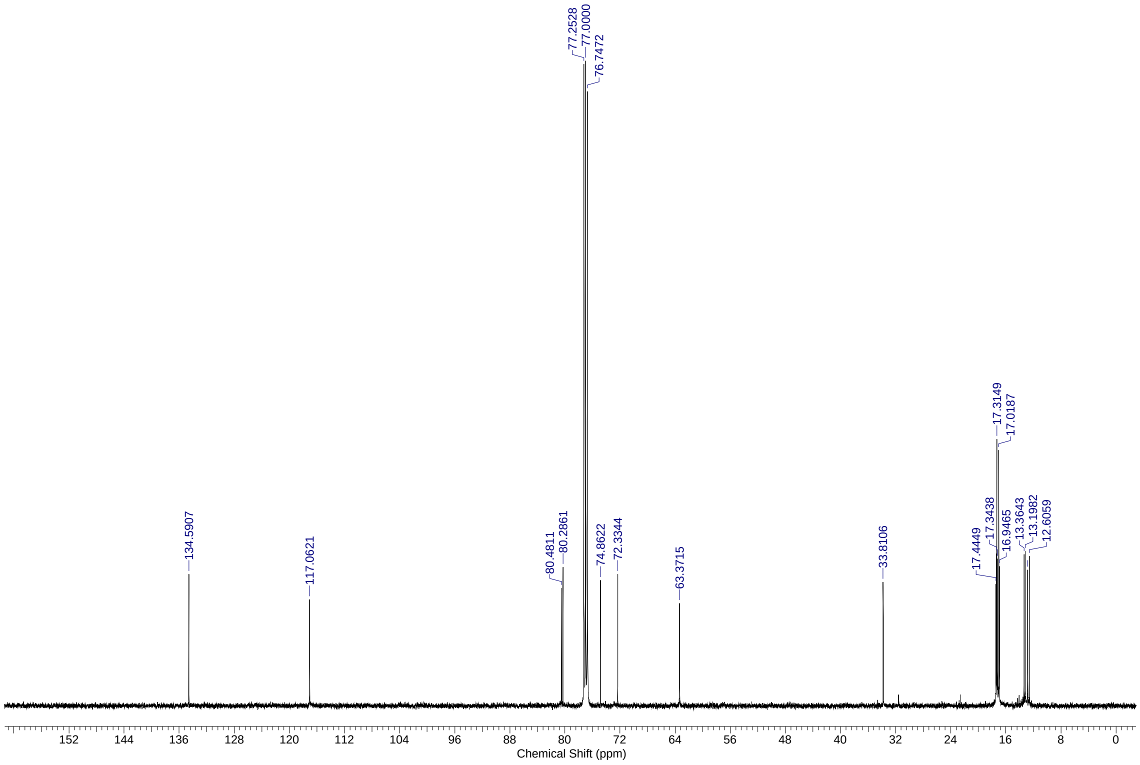

Fig. S76. ^{1}H NMR (500 MHz, $CDCl_3$) and $^{13}C\{H\}$ NMR (125.7 MHz, $CDCl_3$) of compound **86**.

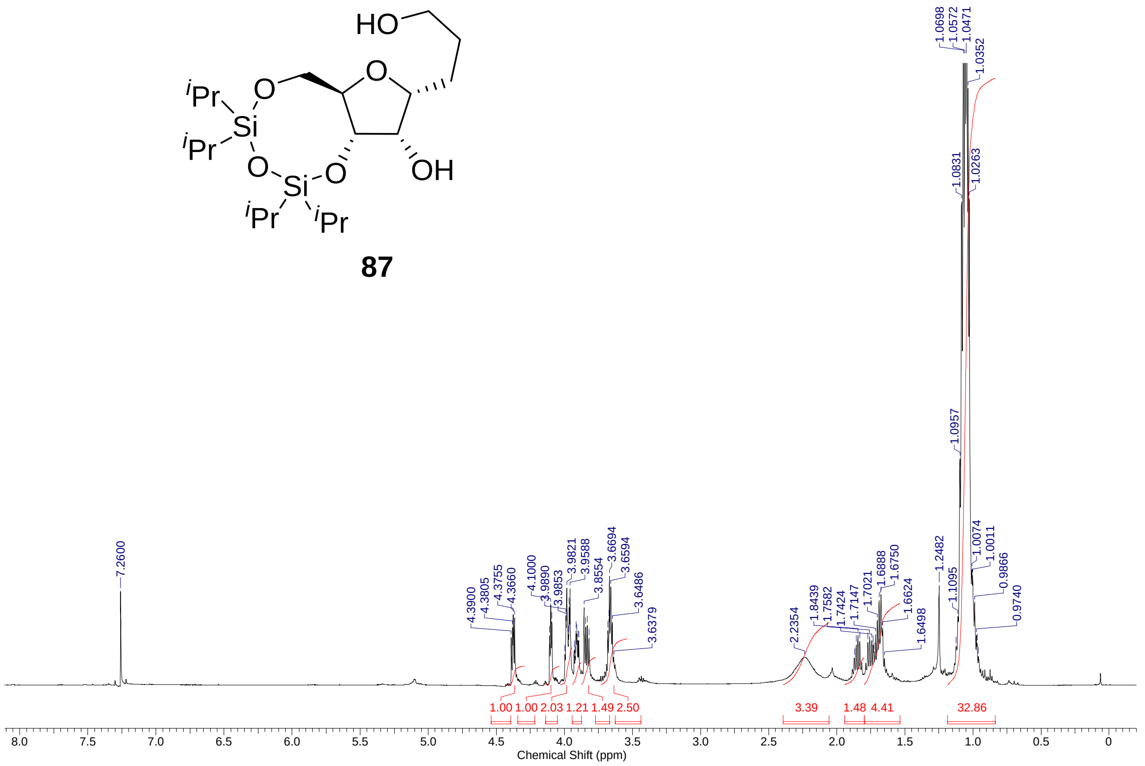

87

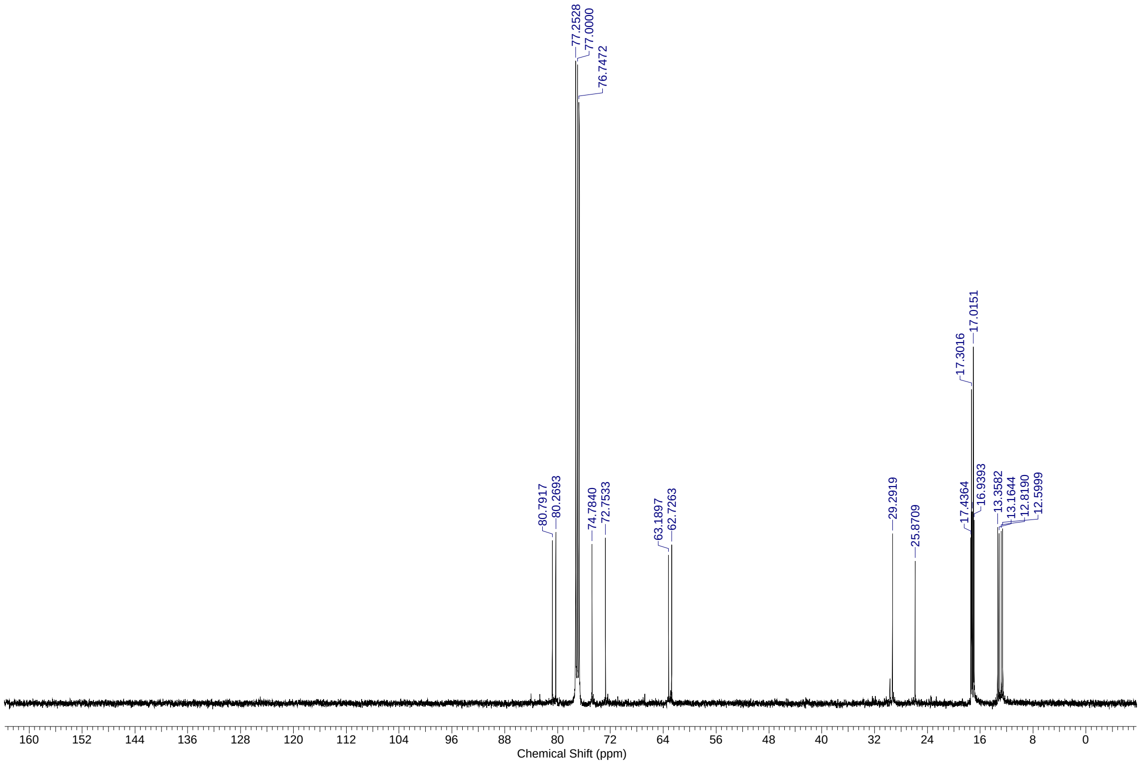

Fig. S77. ^{1}H NMR (500 MHz, $CDCl_3$) and $^{13}C\{H\}$ NMR (125.7 MHz, $CDCl_3$) of compound **87**.

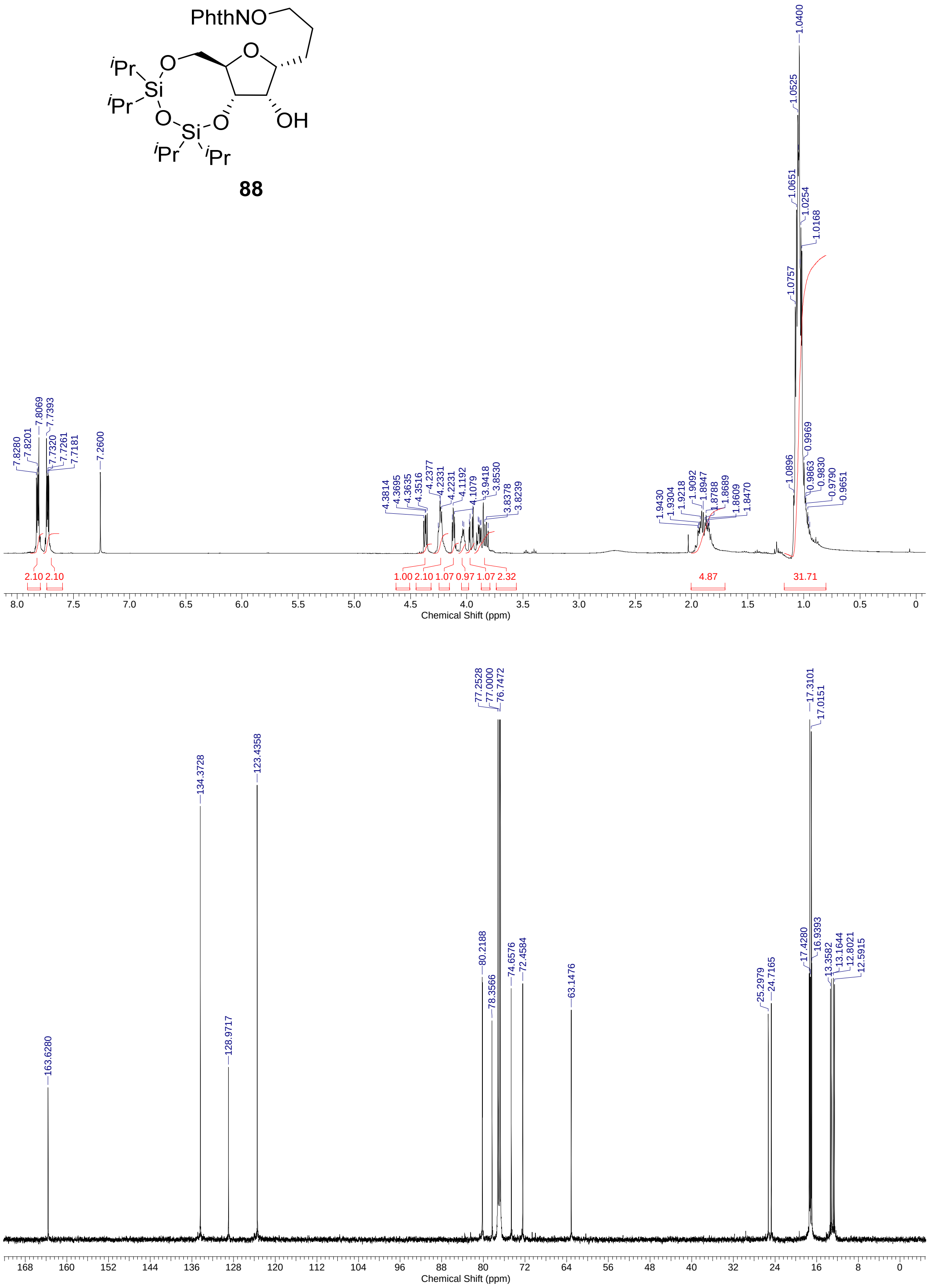

Fig. S78. ^{1}H NMR (400 MHz, $CDCl_3$) and $^{13}C\{H\}$ NMR (125.7 MHz, $CDCl_3$) of compound **88**.

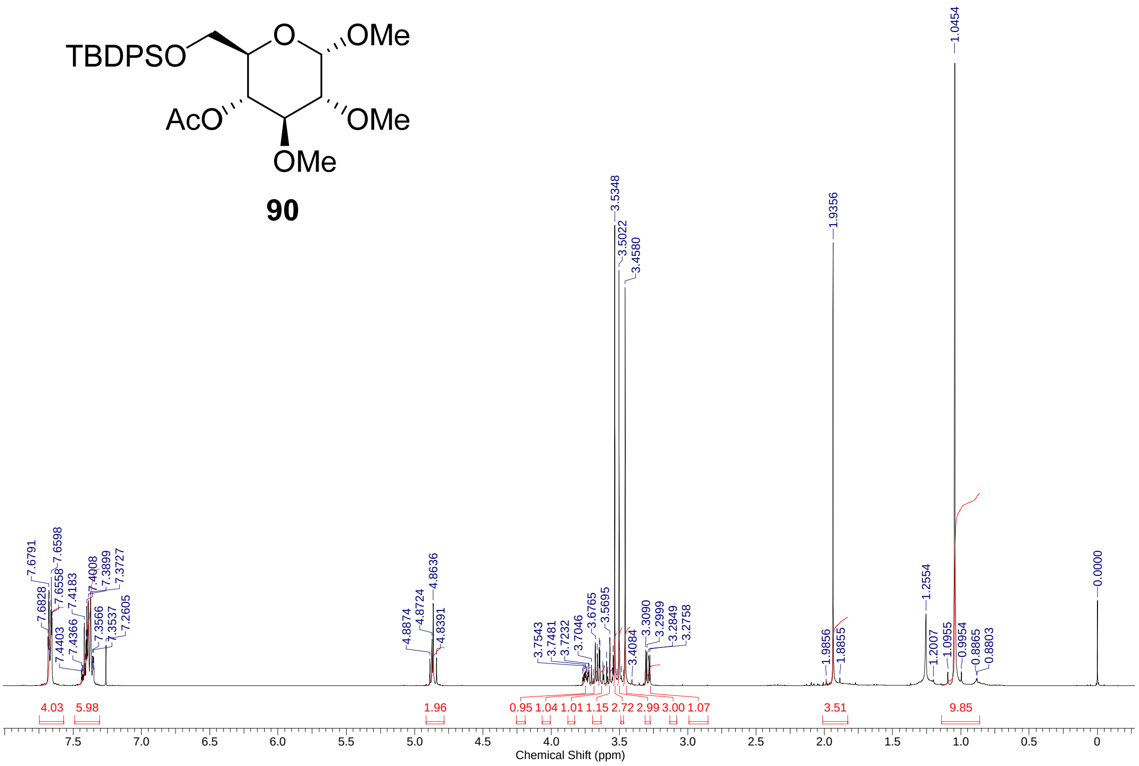

90

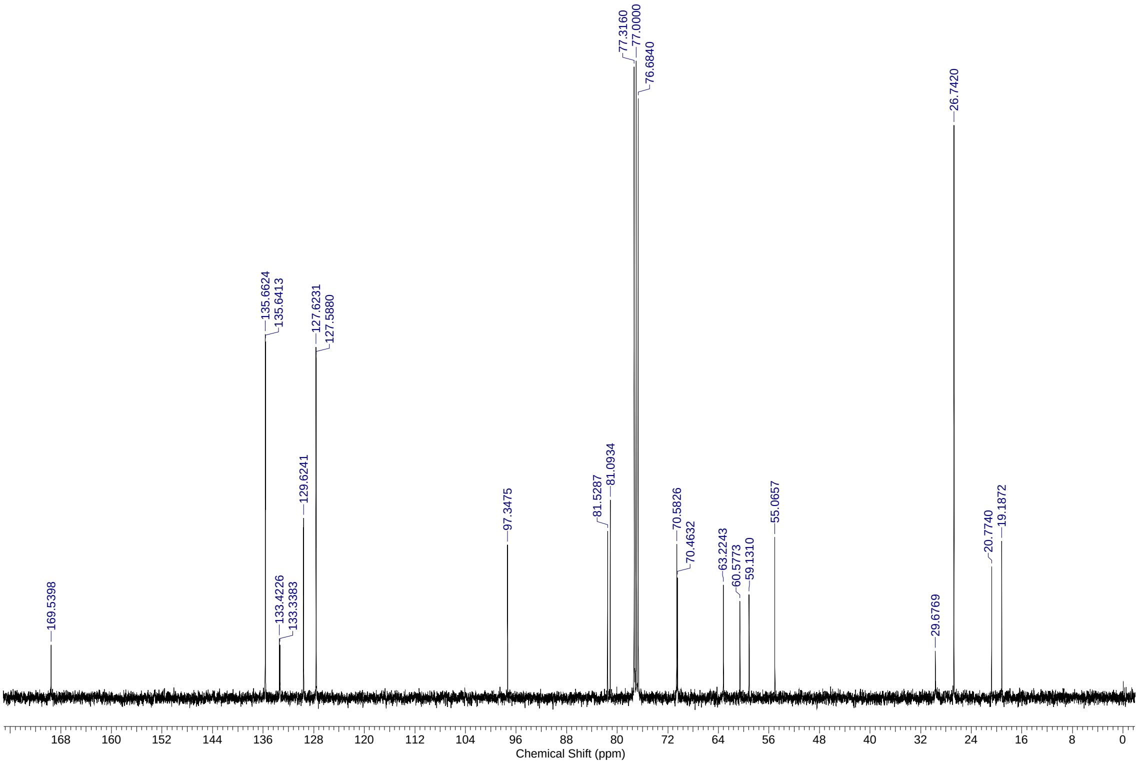

Fig. S79. ^{1}H NMR (400 MHz, $CDCl_3$) and ^{13}C{H} NMR (100.6 MHz, $CDCl_3$) of compound **90**.

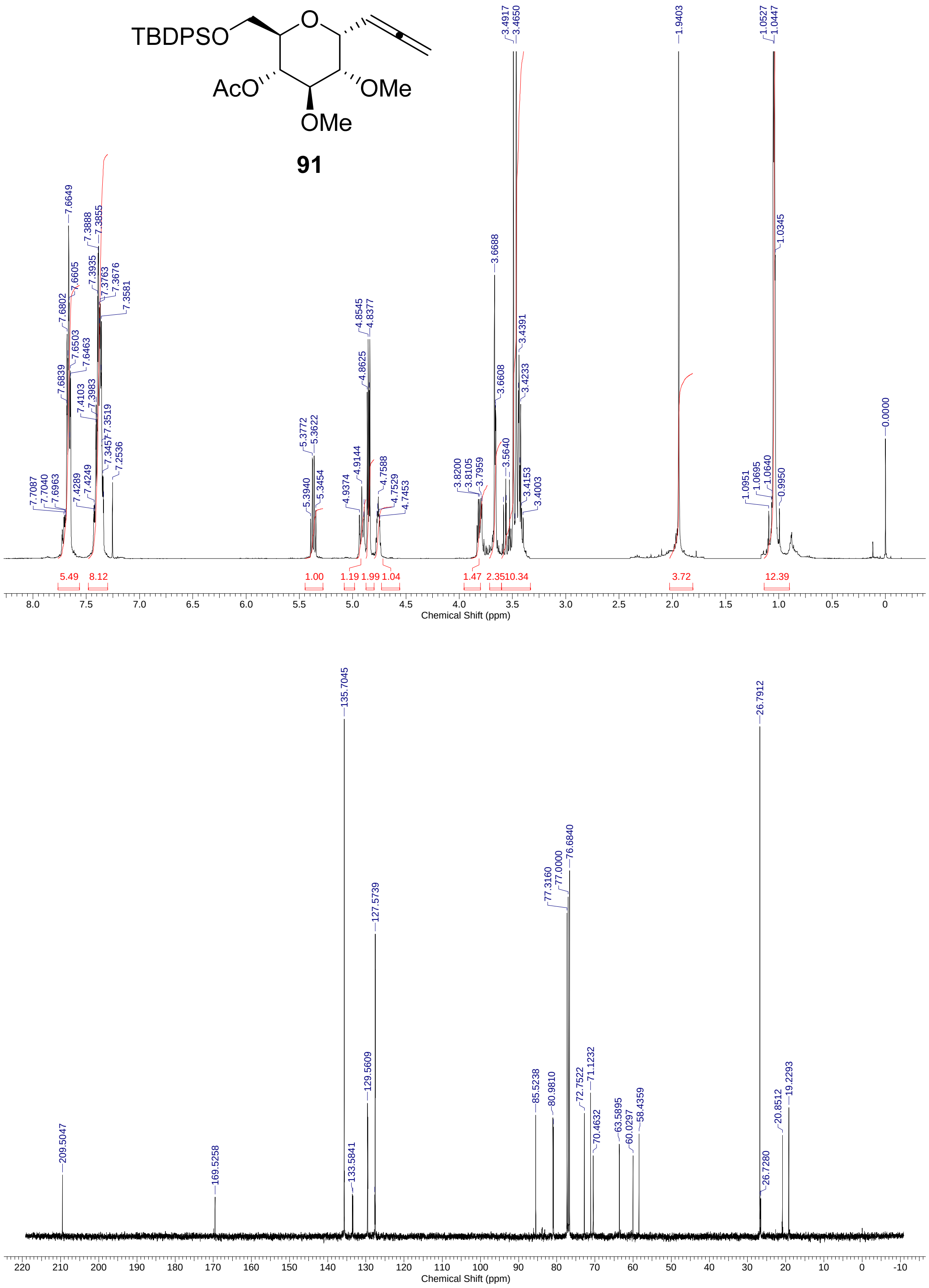

Fig. S80. ^{1}H NMR (400 MHz, $CDCl_3$) and ^{13}C{H} NMR (100.6 MHz, $CDCl_3$) of compound **91**.

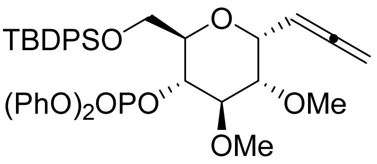

92

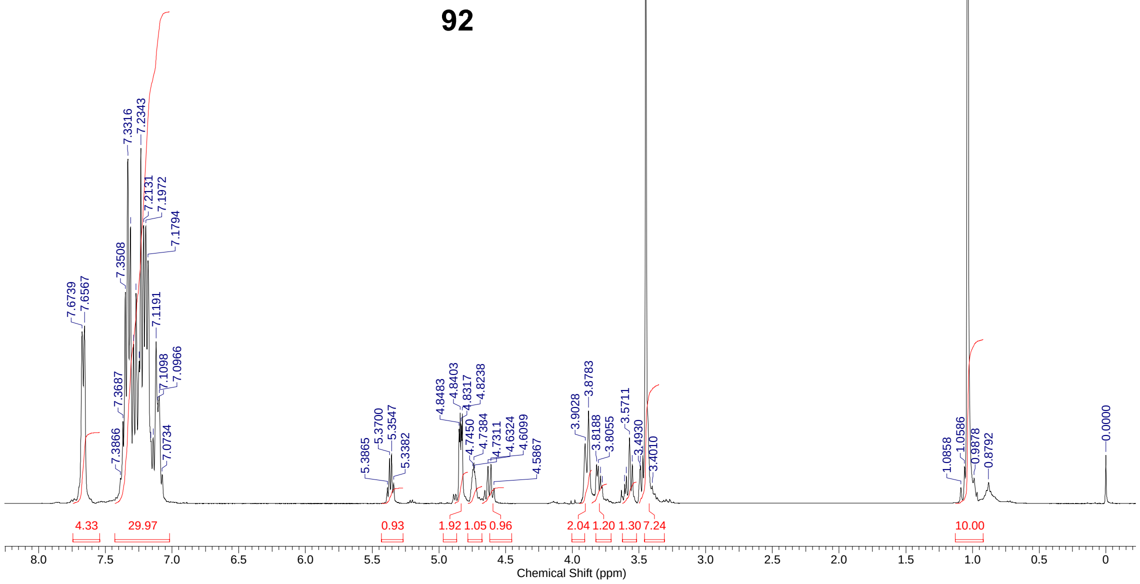

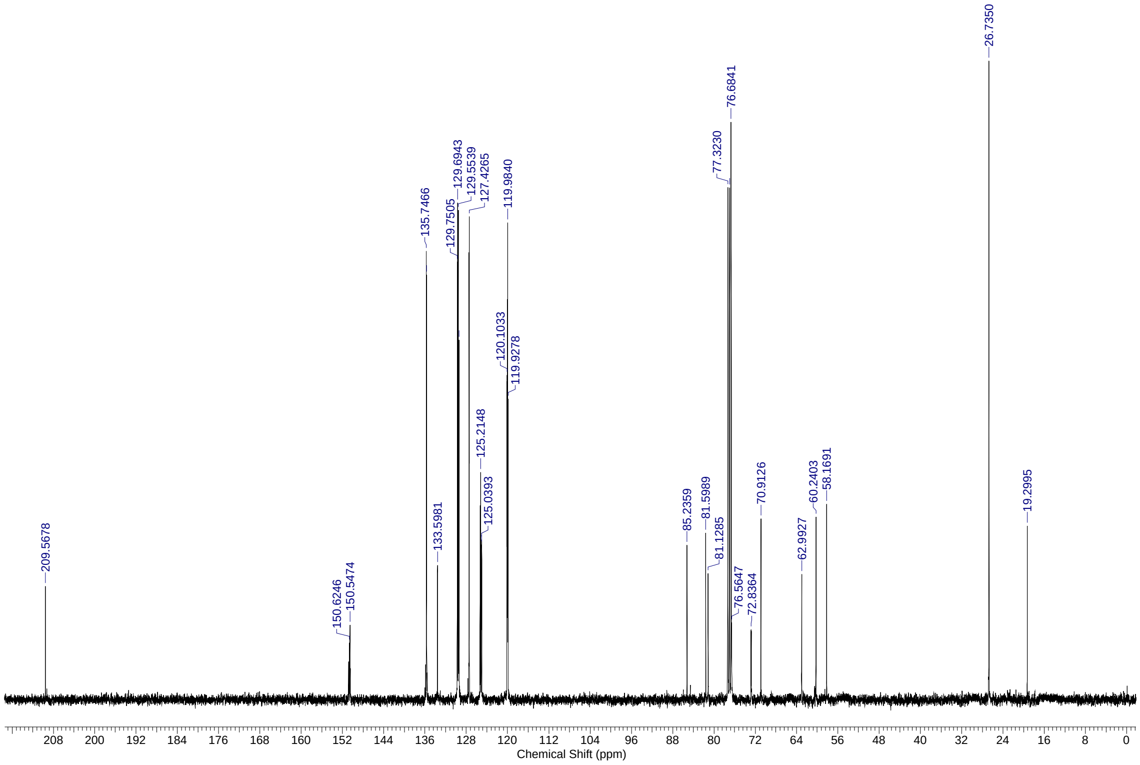

Fig. S81. ^{1}H NMR (500 MHz, $CDCl_3$) and ^{13}C{H} NMR (100.6 MHz, $CDCl_3$) of compound **92**.

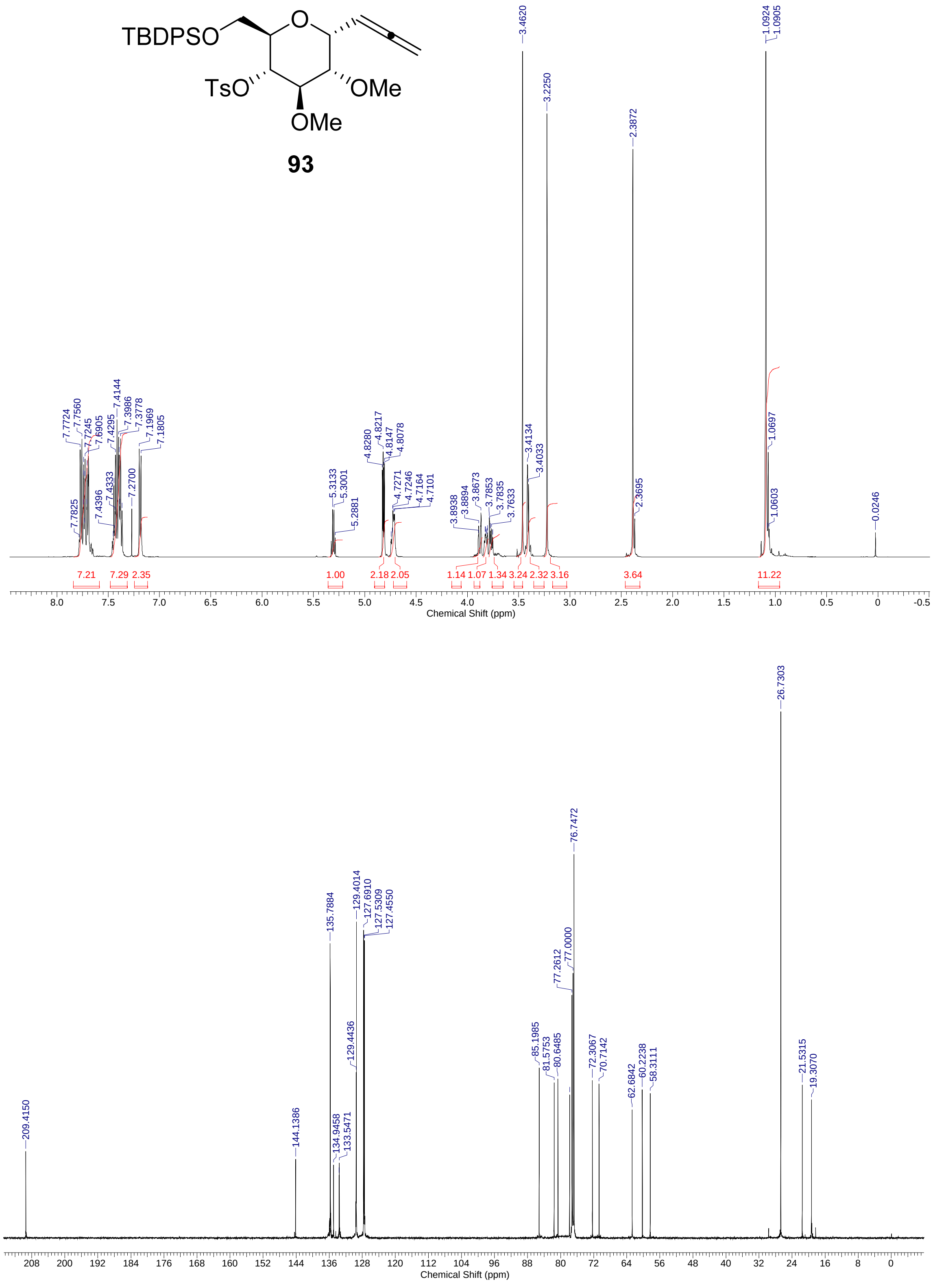

Fig. S82. ^{1}H NMR (500 MHz, $CDCl_3$) and ^{13}C{H} NMR (125.7 MHz, $CDCl_3$) of compound **93**.

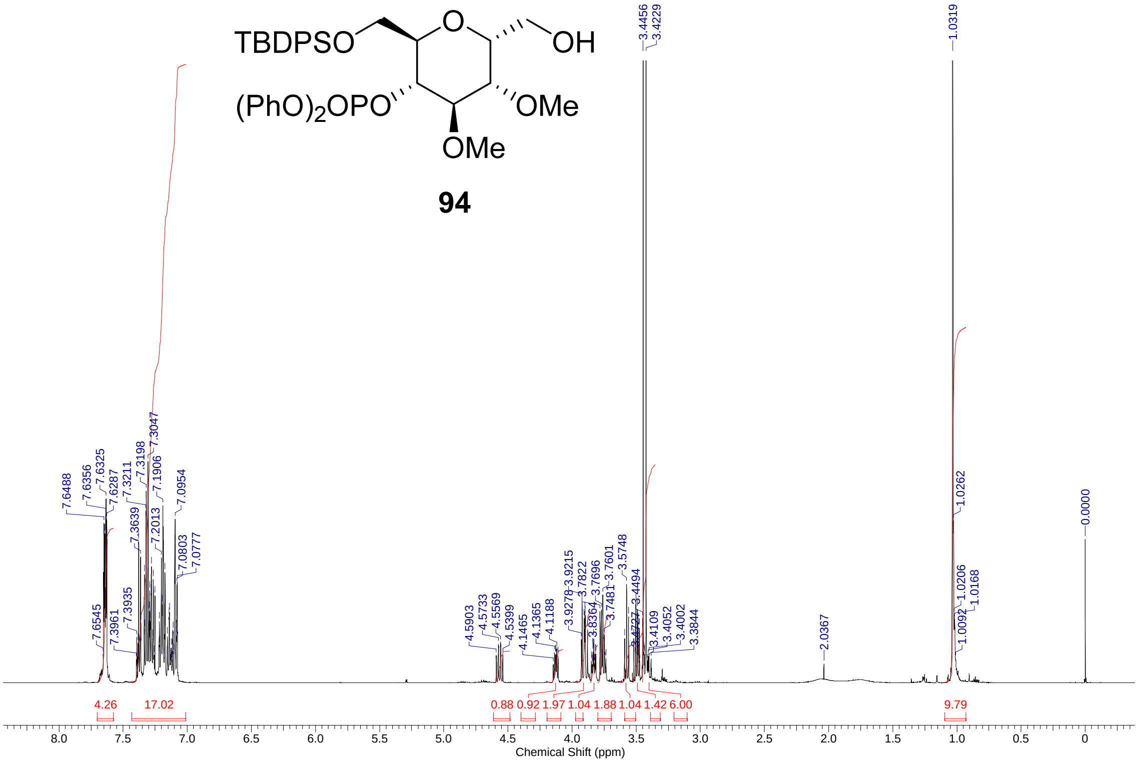

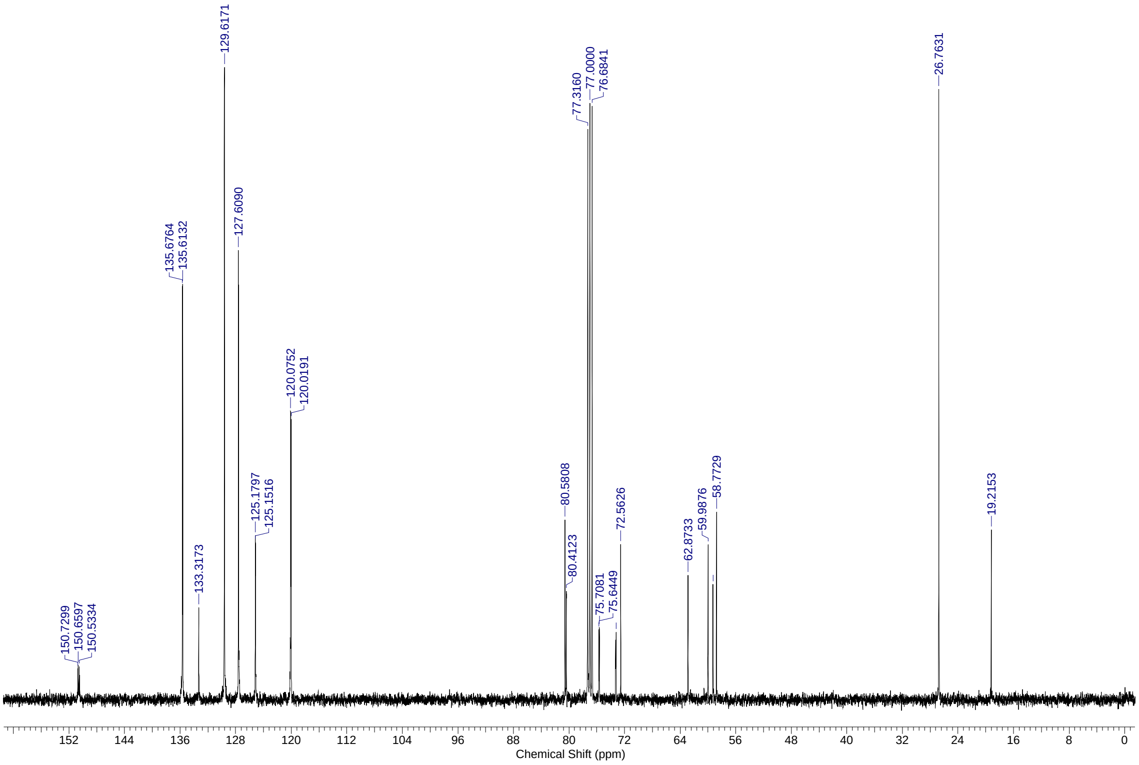

Fig. S83. ^{1}H NMR (500 MHz, $CDCl_3$) and $^{13}C\{H\}$ NMR (100.6 MHz, $CDCl_3$) of compound **94**.

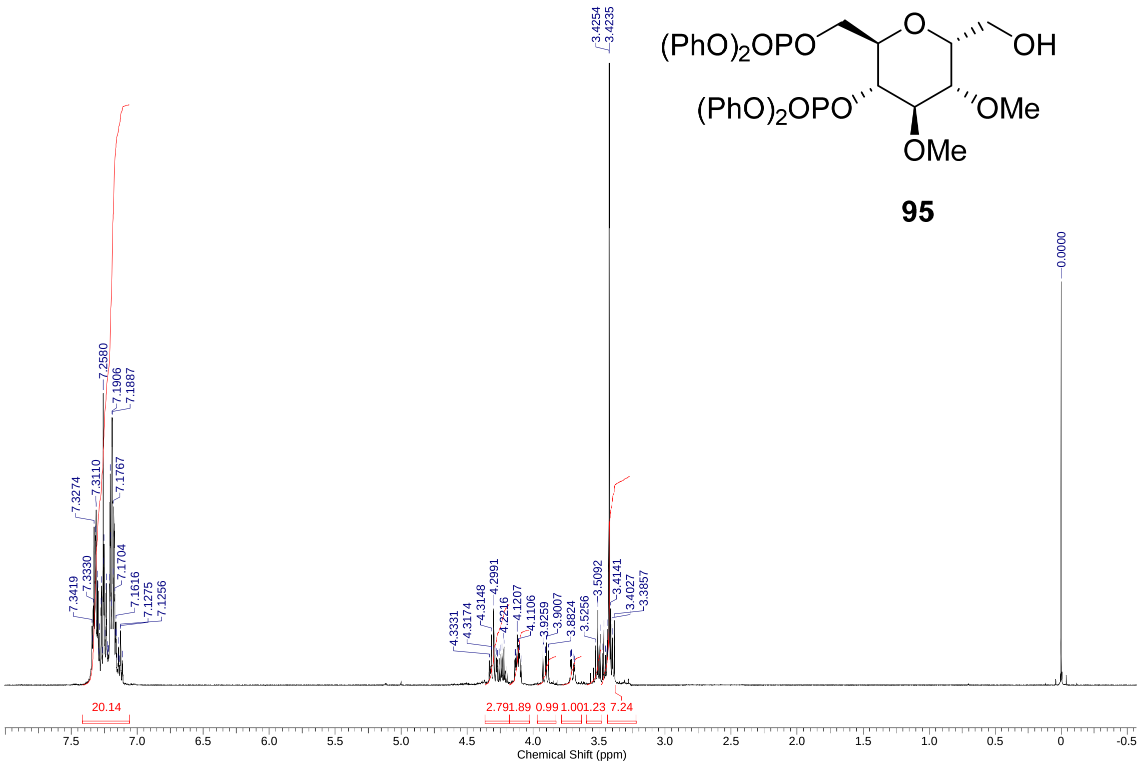

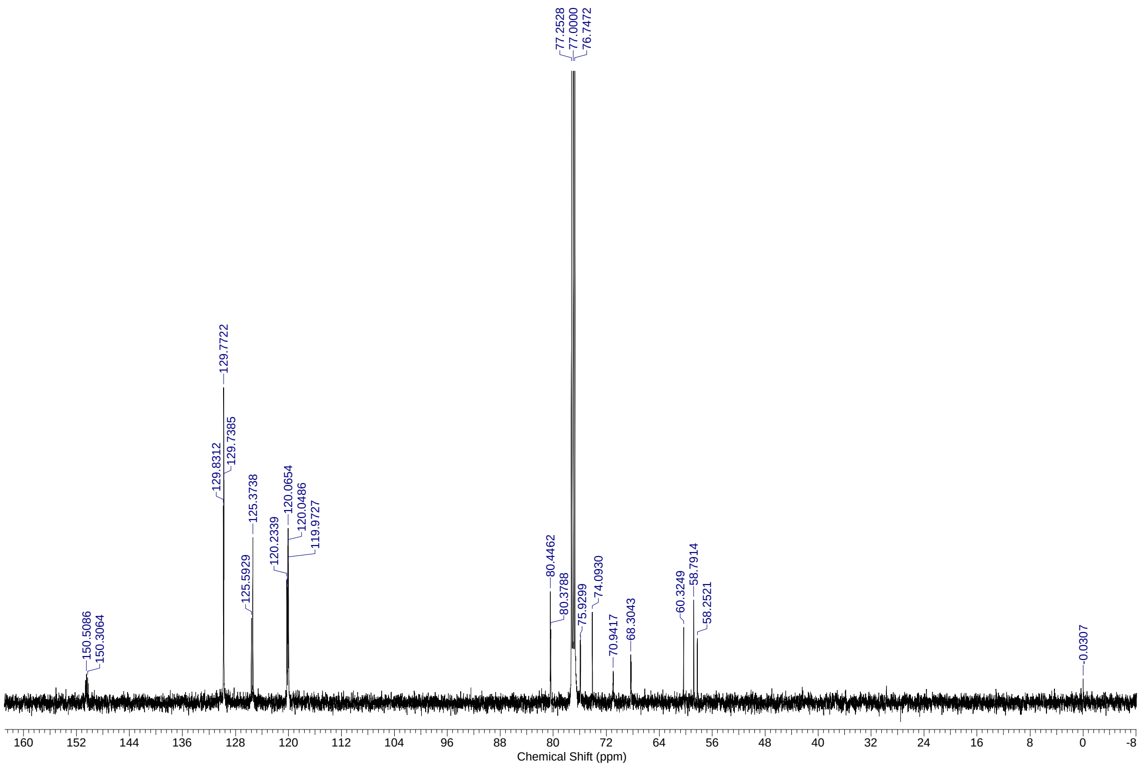

Fig. S84. ^{1}H NMR (500 MHz, $CDCl_3$) and $^{13}C\{H\}$ NMR (125.7 MHz, $CDCl_3$) of compound **95**.

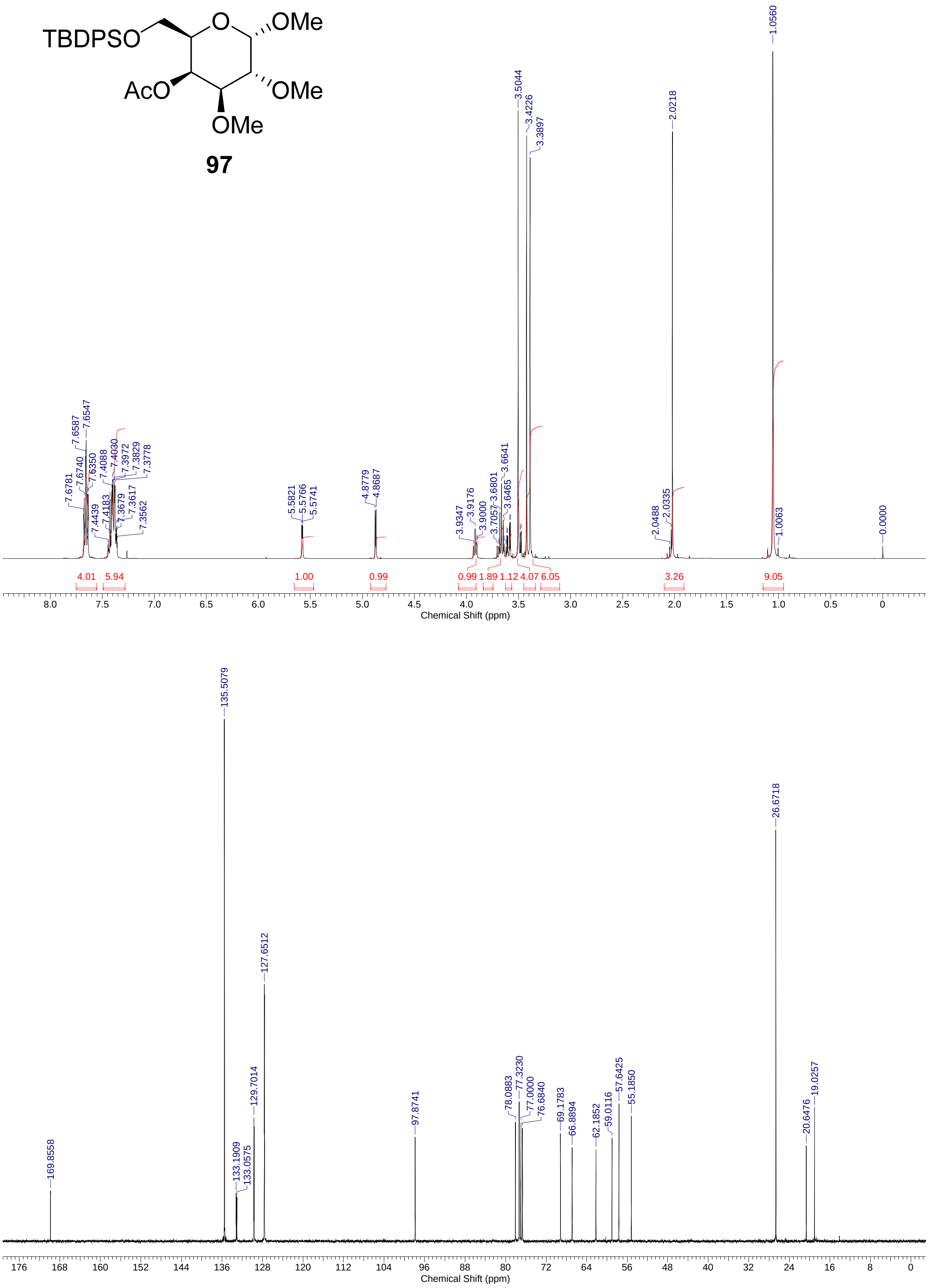

Fig. S85. ^{1}H NMR (400 MHz, $CDCl_3$) and ^{13}C{H} NMR (100.6 MHz, $CDCl_3$) of compound **97**.

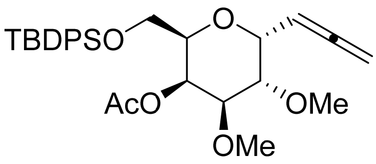

98

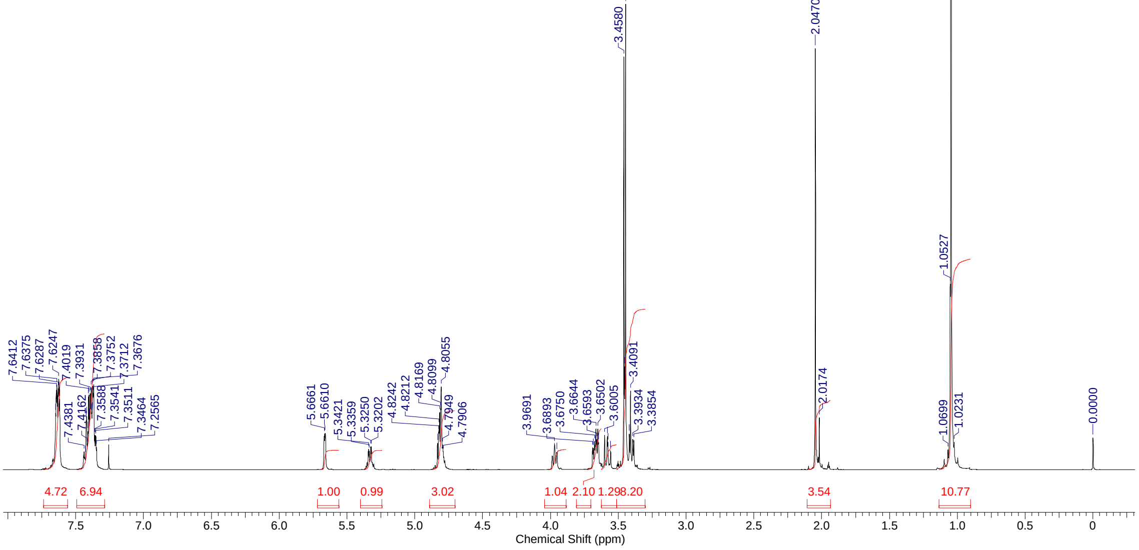

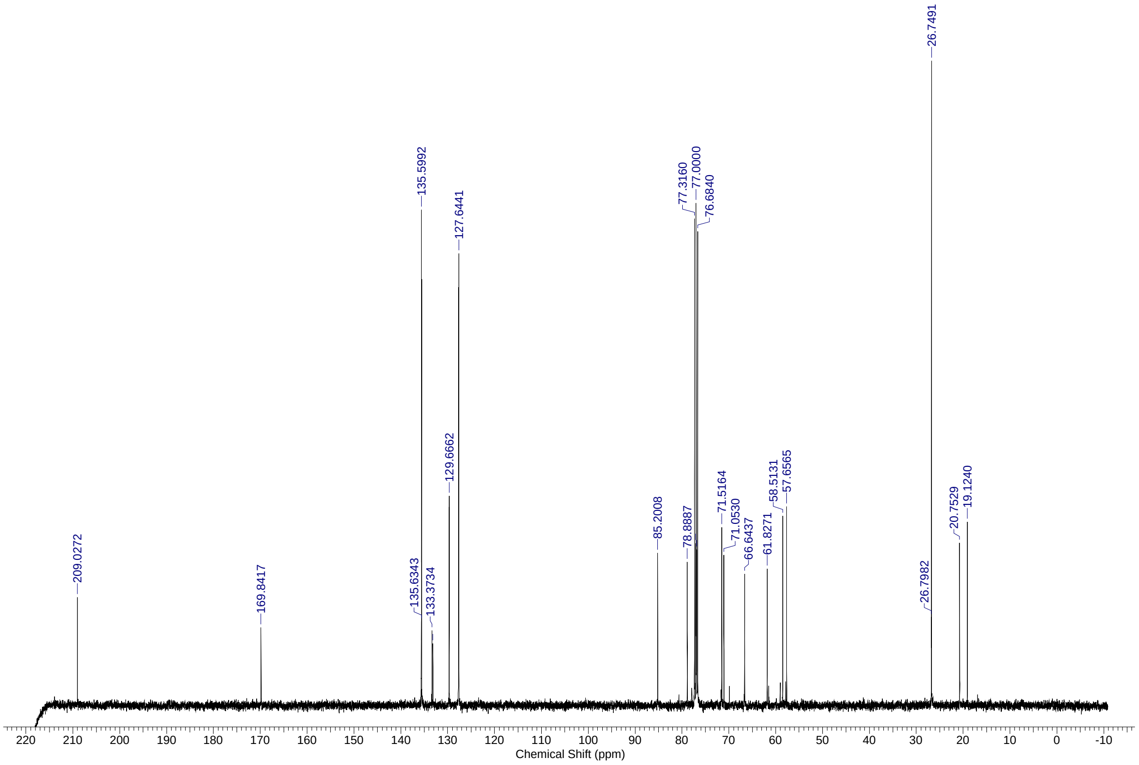

Fig. S86. ^{1}H NMR (400 MHz, $CDCl_3$) and ^{13}C{H} NMR (100.6 MHz, $CDCl_3$) of compound **98**.

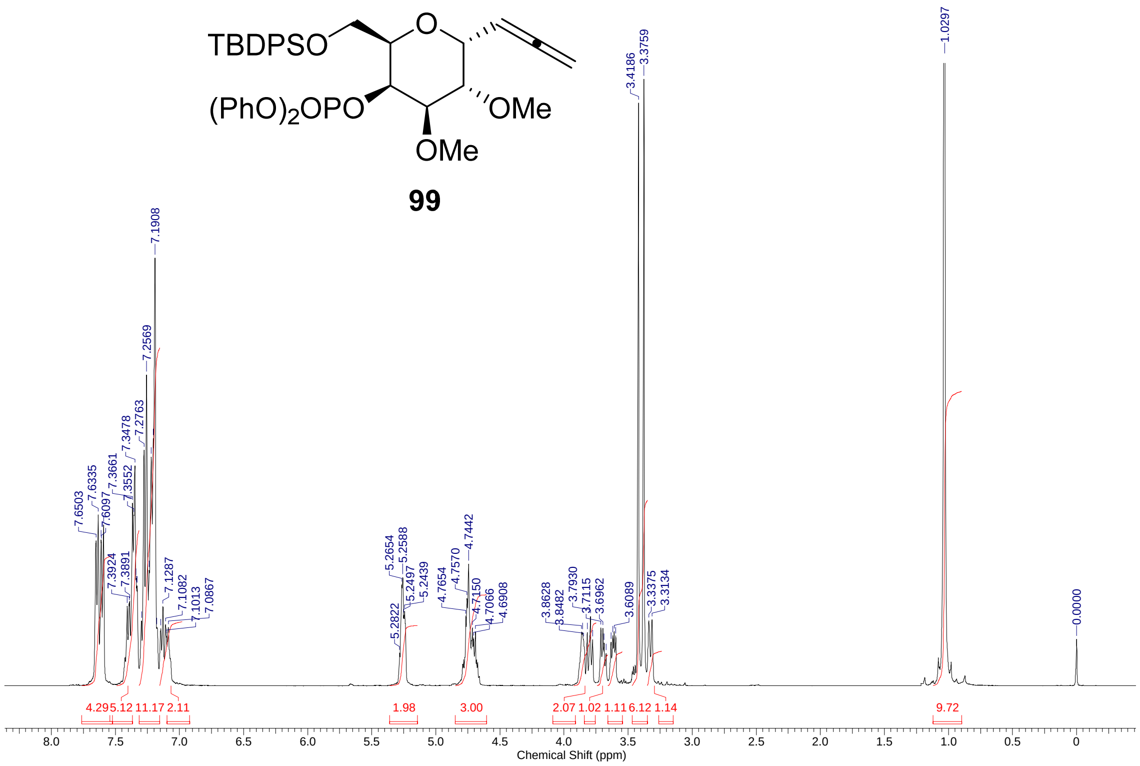

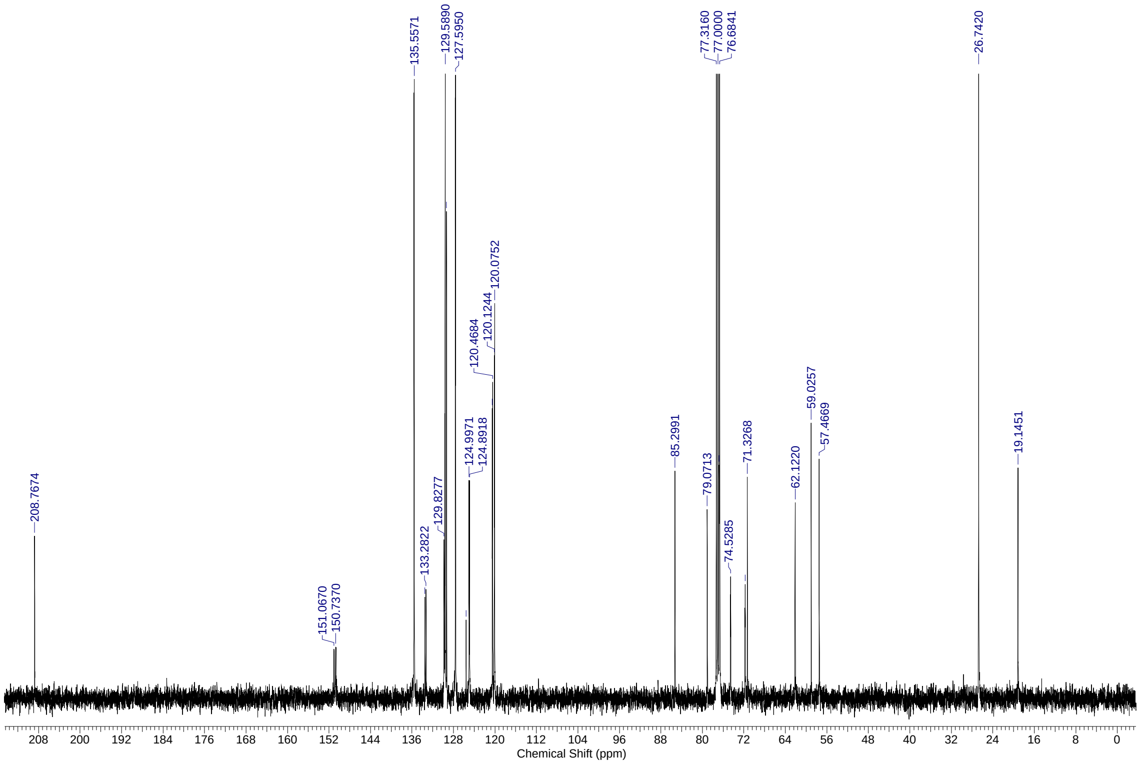

Fig. S87. ^{1}H NMR (400 MHz, $CDCl_3$) and $^{13}C\{H\}$ NMR (100.6 MHz, $CDCl_3$) of compound **99**.

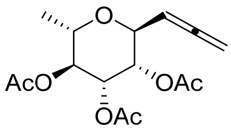

101

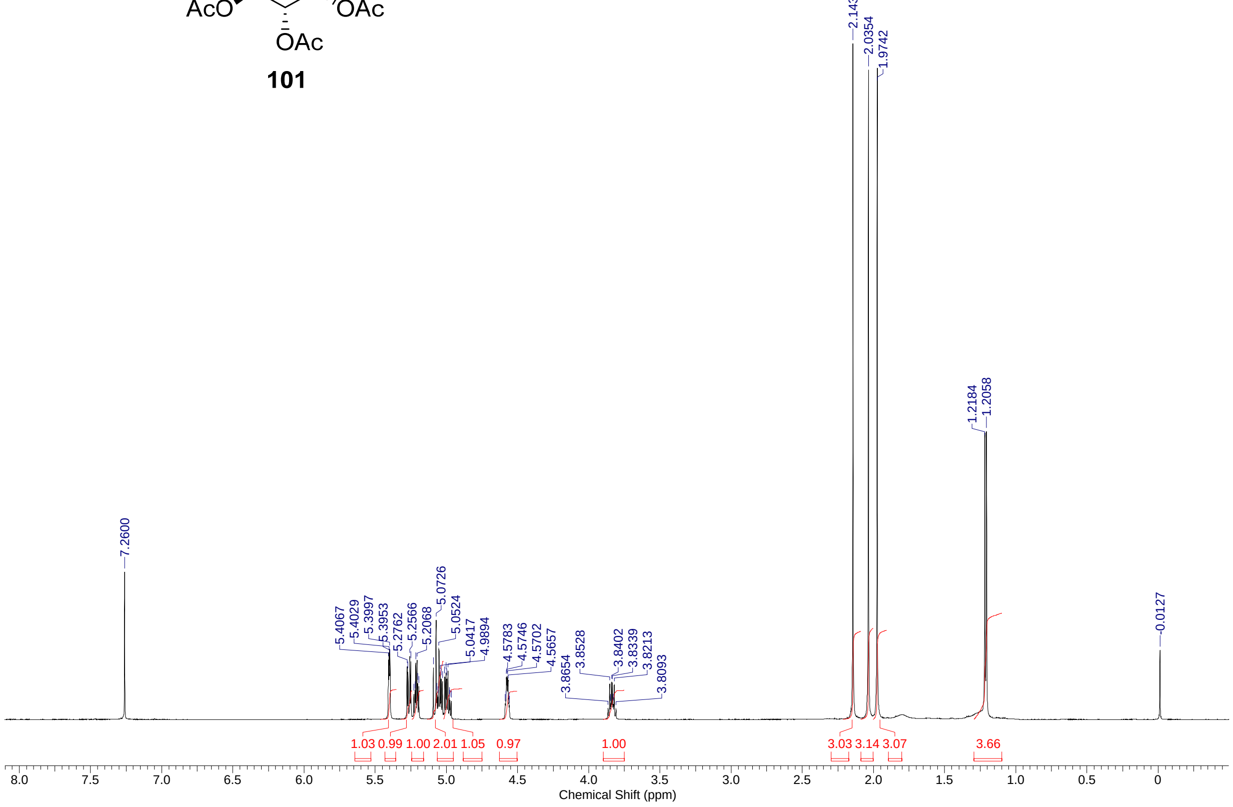

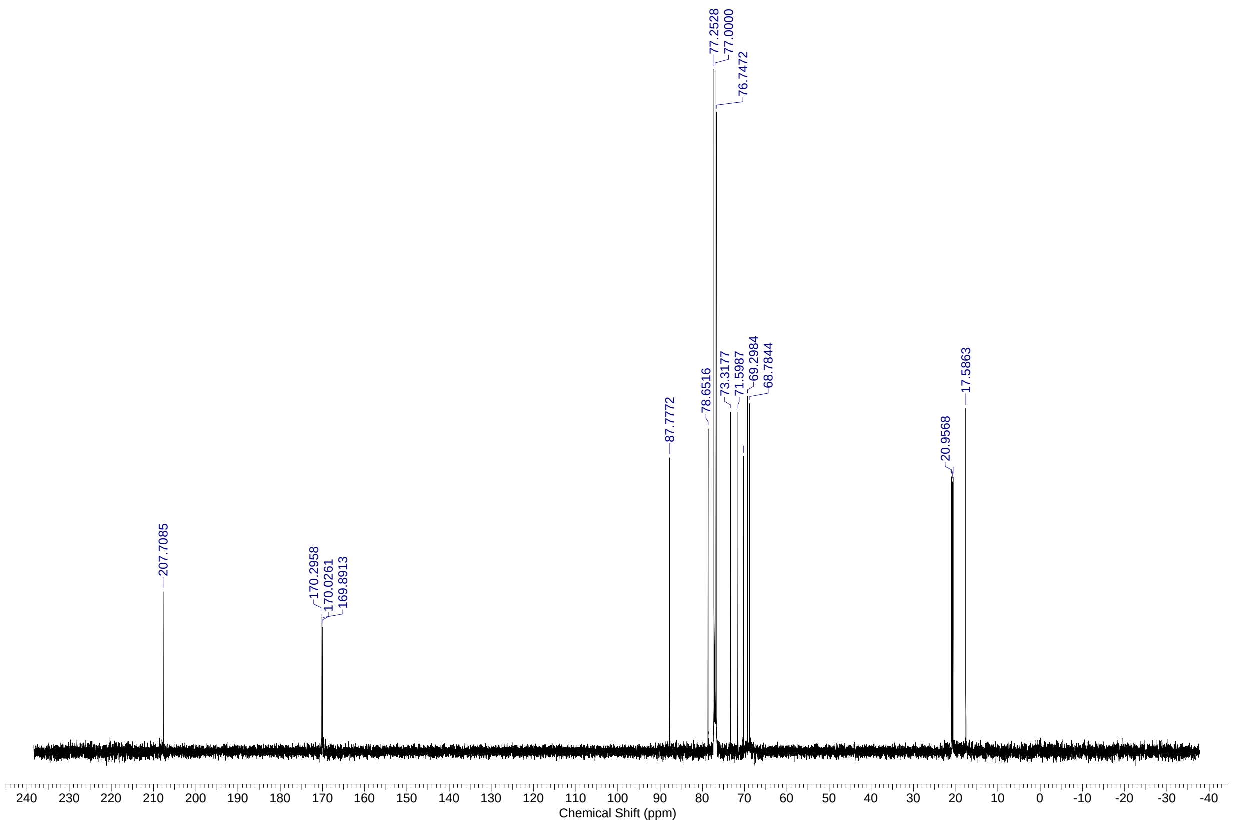

Fig. S88. ^{1}H NMR (500 MHz, $CDCl_3$) and $^{13}C\{H\}$ NMR (125.7 MHz, $CDCl_3$) of compound **101**.

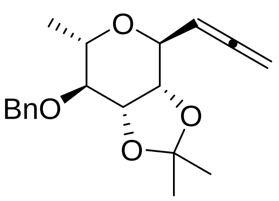

103

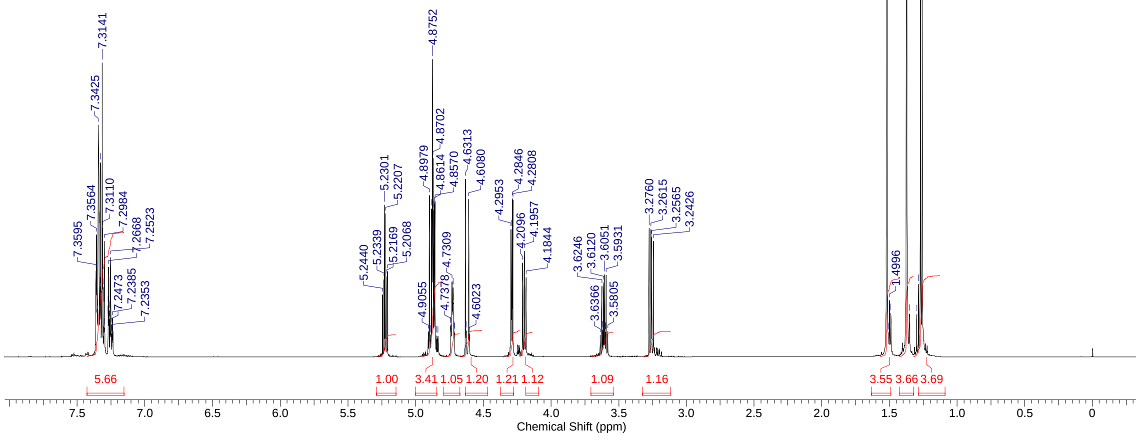

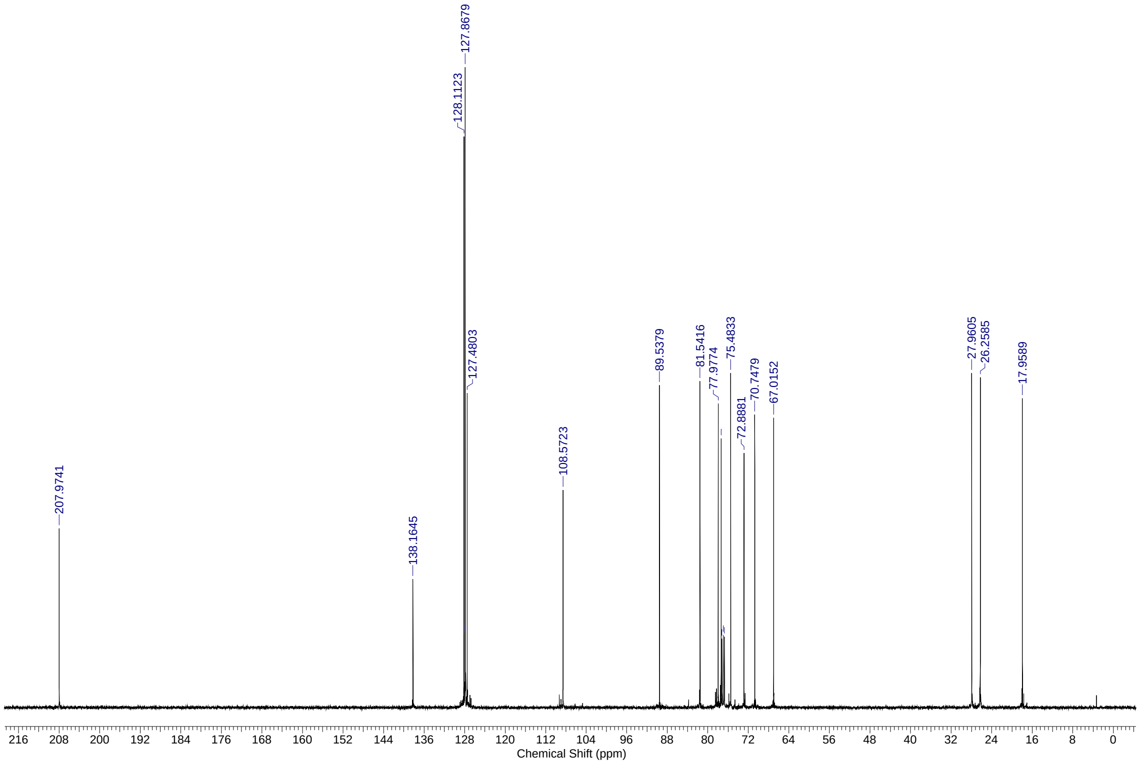

Fig. S89. ^{1}H NMR (500 MHz, $CDCl_3$) and ^{13}C{H} NMR (125.7 MHz, $CDCl_3$) of compound **103**.

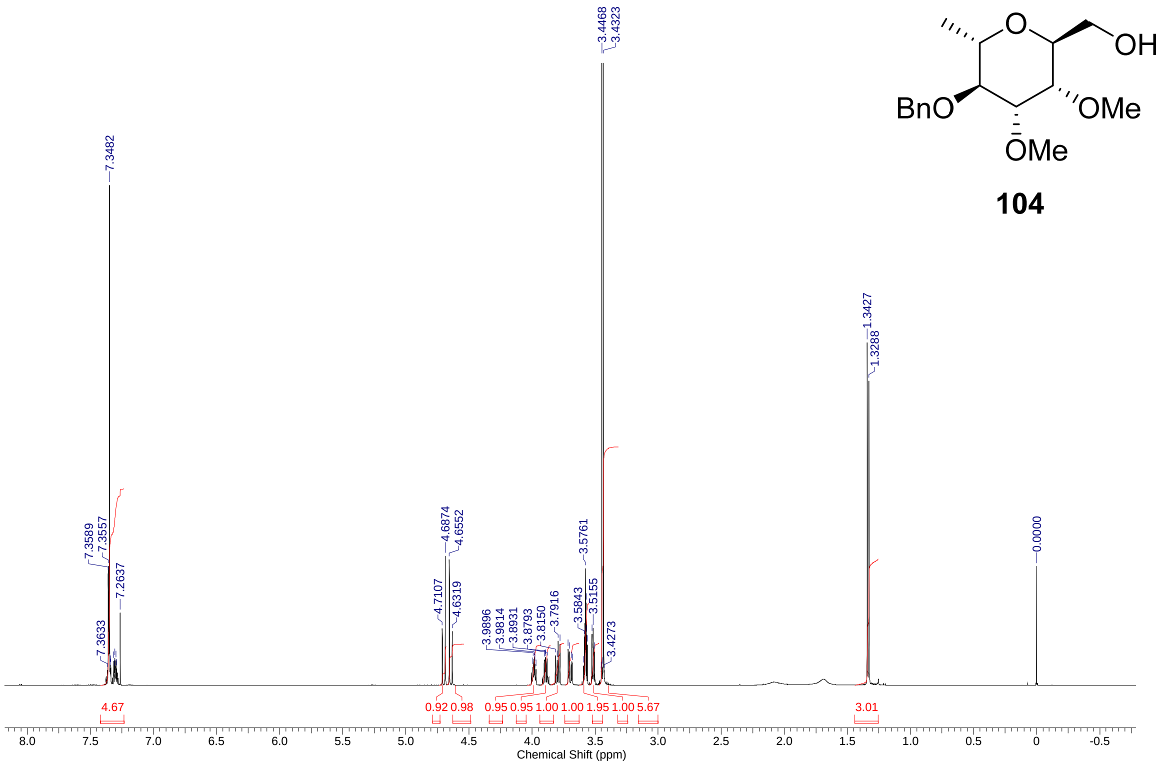

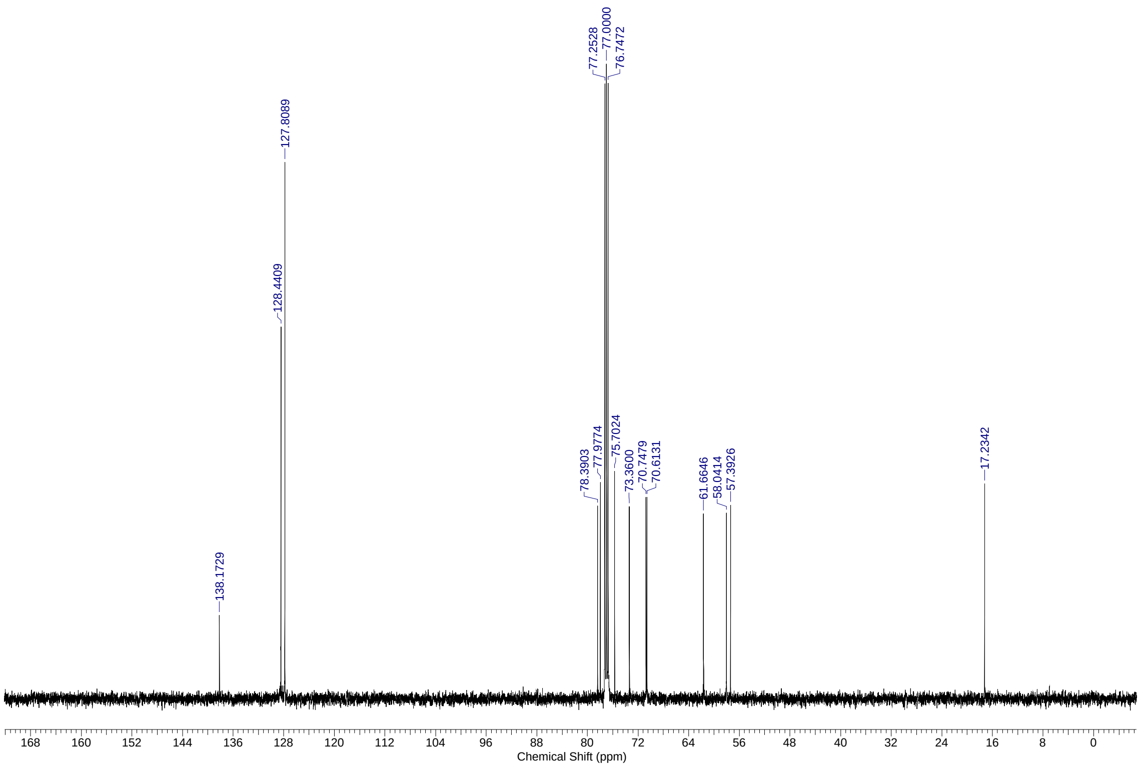

Fig. S90. ^{1}H NMR (500 MHz, $CDCl_3$) and $^{13}C\{H\}$ NMR (125.7 MHz, $CDCl_3$) of compound **104**.

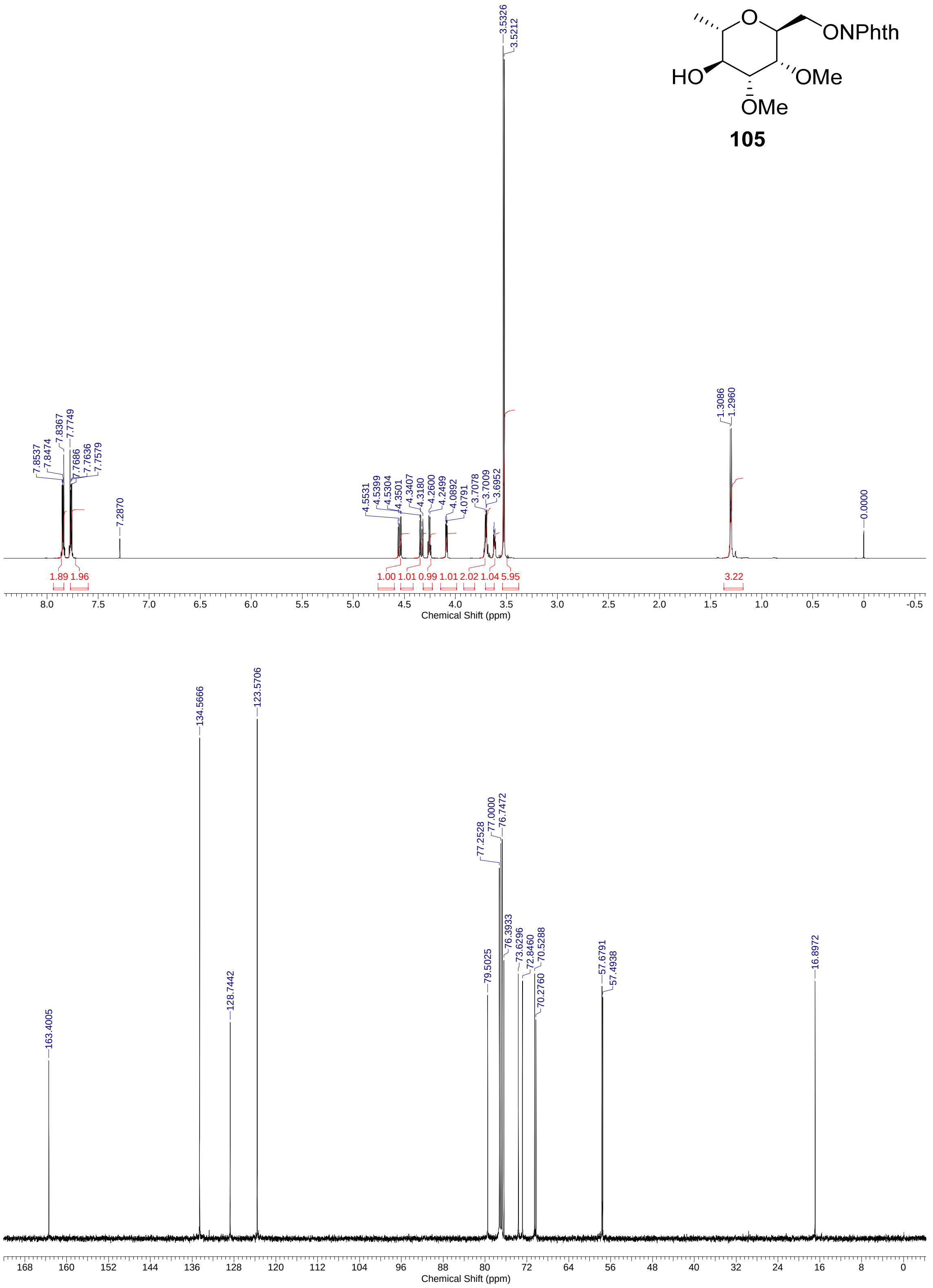

Fig. S91. ^{1}H NMR (500 MHz, $CDCl_3$) and ^{13}C{H} NMR (125.7 MHz, $CDCl_3$) of compound **105**.

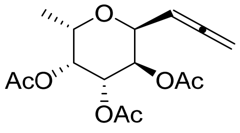

107

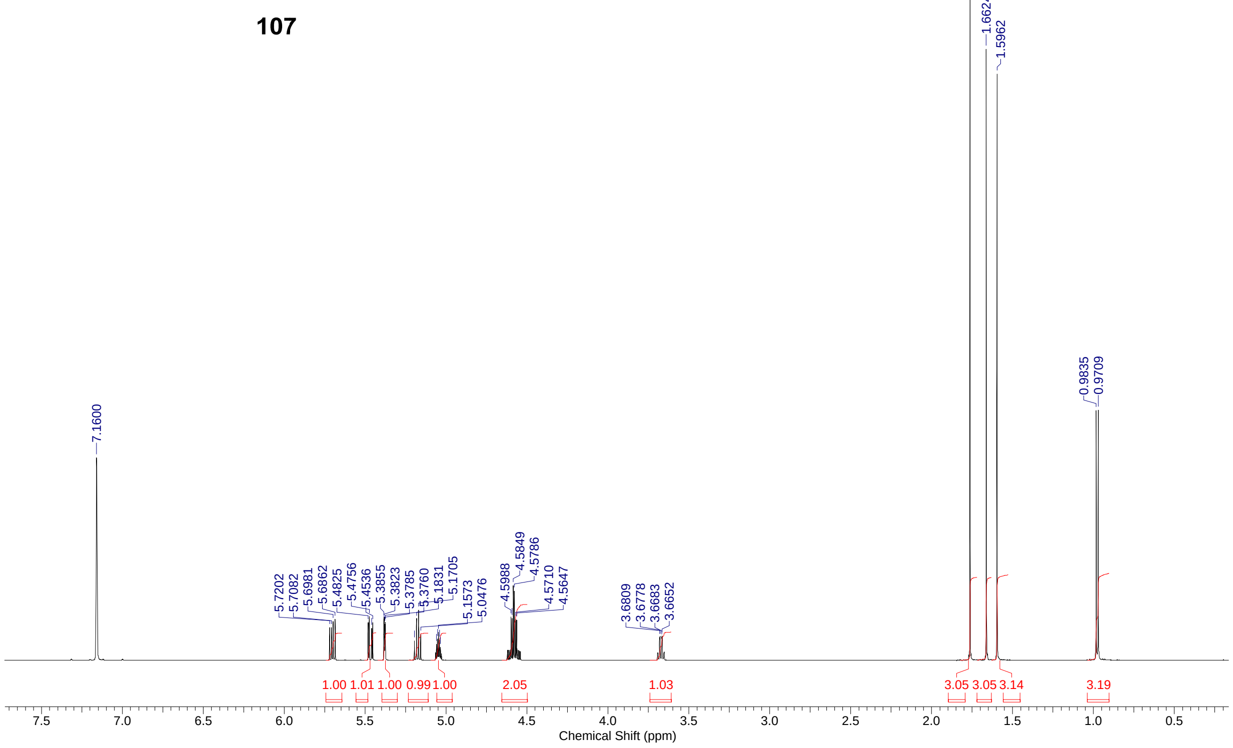

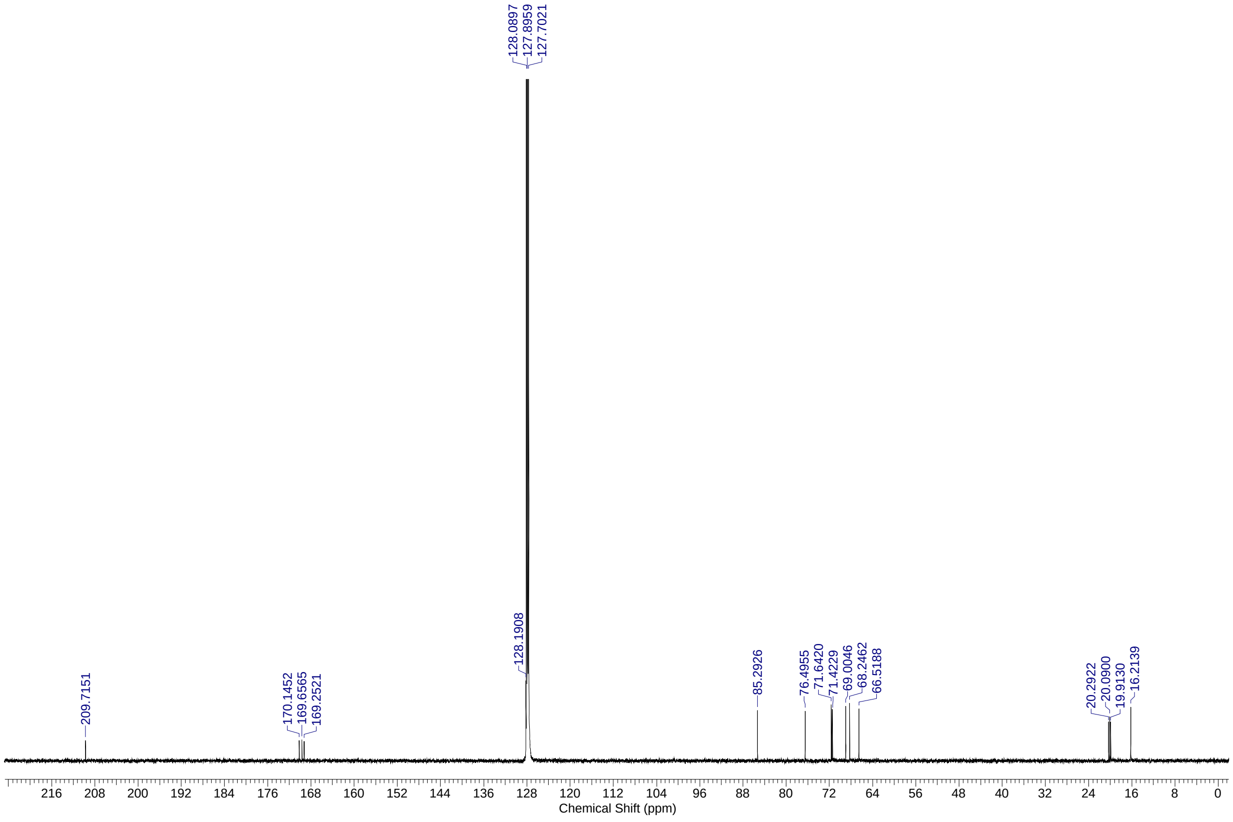

Fig. S92. ^{1}H NMR (500 MHz, C_6D_6) and ^{13}C{H} NMR (125.7 MHz, C_6D_6) of compound **107**.

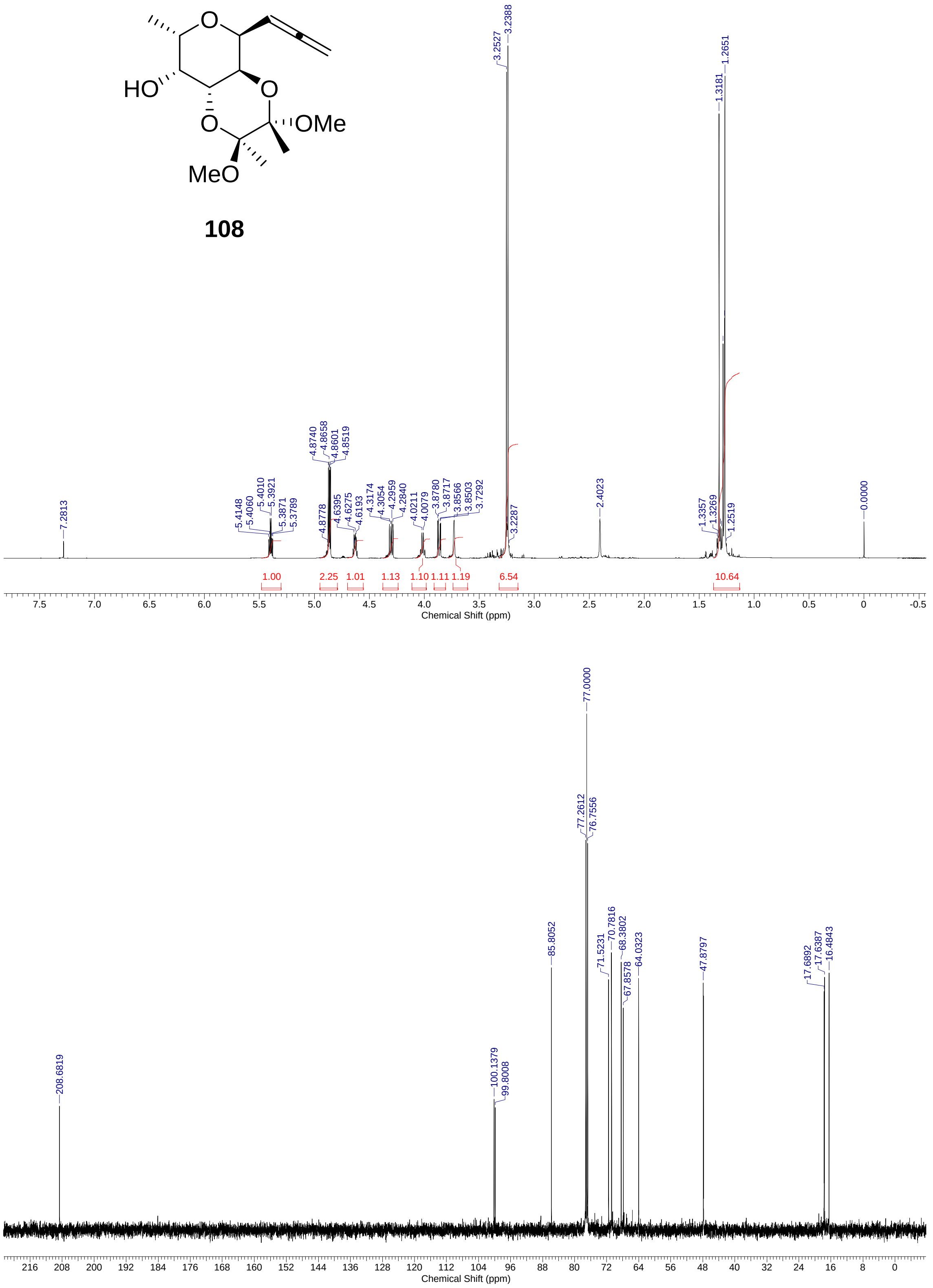

Fig. S93. ^{1}H NMR (500 MHz, $CDCl_3$) and $^{13}C\{H\}$ NMR (125.7 MHz, $CDCl_3$) of compound **108**.

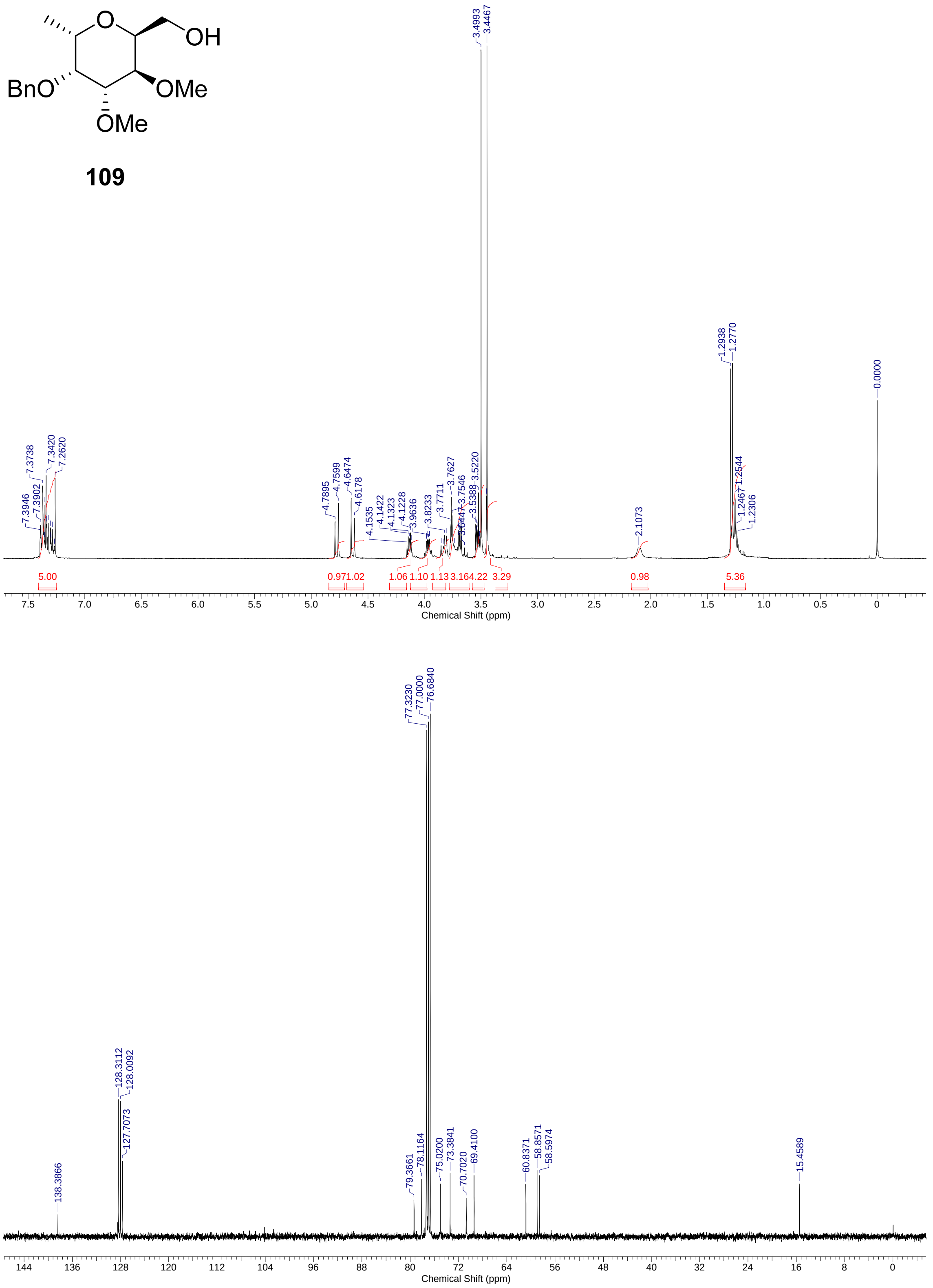

Fig. S94. ^{1}H NMR (400 MHz, $CDCl_3$) and ^{13}C{H} NMR (100.6 MHz, $CDCl_3$) of compound **109**.
